# Estrogen Receptor-Regulated Gene Signatures in Invasive Breast Cancer Cells and Aggressive Breast Tumors

**DOI:** 10.3390/cancers14122848

**Published:** 2022-06-09

**Authors:** Emily Smart, Svetlana E. Semina, Luis H. Alejo, Nidhi S. Kansara, Jonna Frasor

**Affiliations:** Department of Physiology and Biophysics, University of Illinois at Chicago, Chicago, IL 60612, USA; esmart@uic.edu (E.S.); semina@uic.edu (S.E.S.); lalejo@uic.edu (L.H.A.); nkansa2@uic.edu (N.S.K.)

**Keywords:** estrogen receptor, invasion, breast cancer

## Abstract

**Simple Summary:**

Metastatic breast cancer remains a major clinical problem, contributing to significant patient mortality, which is partly due to a lack of understanding around the early changes within the primary tumor. Tumors frequently become more aggressive and less treatable due to the activation of other signaling pathways, and, in ER+ disease, one of these pathways is NFκB. The coactivation of ER and NFκB (via IKKβ) promotes invasion and metastasis, and, here, we identify the signatures that are associated with these phenotypes. These signatures improve our understanding of how ER can drive aggressive disease, and may lead to the identification of key drivers, which could potentially be targeted with future therapies.

**Abstract:**

Most metastatic breast cancers arise from estrogen receptor α (ER)-positive disease, and yet the role of ER in promoting metastasis is unclear. Here, we used an ER+ breast cancer cell line that is highly invasive in an ER- and IKKβ-dependent manner. We defined two ER-regulated gene signatures that are specifically regulated in the subpopulations of invasive cells. The first consists of proliferation-associated genes, which is a known function of ER, which actually suppress rather than enhance invasion. The second signature consists of genes involved in essential biological processes, such as organelle assembly and vesicle trafficking. Importantly, the second subpopulation-specific signature is associated with aggressive disease and poor patient outcome, independently of proliferation. These findings indicate a complex interplay between ER-driven proliferation and invasion, and they define new ER-regulated gene signatures that are predictive of aggressive ER+ breast cancer.

## 1. Introduction

Breast cancer remains the most highly diagnosed cancer, with an estimated 276,000 cases diagnosed in the United States in 2020. Approximately 75% of breast cancers express estrogen receptor α (ER) [1], and women with ER+ tumors are typically treated with adjuvant endocrine therapies that block ER activity. Treatments include selective estrogen receptor modulators (SERMs), such as tamoxifen, and selective estrogen receptor degraders (SERDs), such as fulvestrant, both of which directly bind and target the activity of ER, and aromatase inhibitors (AIs), such as letrozole, which reduce estrogen production and thereby reduce ER activity. Despite the treatability of ER+ disease, the majority of breast-cancer-related deaths occur in patients with ER+ breast cancer, largely due to disease relapse, which can occur in up to 40% of patients [2]. Disease that has recurred is frequently more aggressive and metastatic and is often resistant to ER-targeted therapies. Current treatments for metastatic disease include endocrine therapy, chemotherapy, targeted therapies, surgery, and radiotherapy. Response is often unpredictable, which is partly due to the untargeted nature of some treatments. In addition, acquired resistance is common. Consequently, advanced metastatic ER+ disease remains a major clinical problem in need of new treatment options.

The role of ER in breast cancer has been well defined and is predominantly involved in promoting proliferation and cell survival [3,4]. However, its role in metastasis is less clear. The processes involved in metastasis can be summarized by the metastatic cascade. Tumors undergo invasion/migration out of the primary site, followed by intravasation into the lymphatic system, followed by the blood stream and circulation, before undergoing extravasation into the metastatic niche and, lastly, colonization [5]. The ER’s role in promoting metastatic processes has largely been defined by its loss of classical function, through the gain of crosstalk mechanisms with other signaling pathways, or by changes in ER coregulators [6]. ER participates in extranuclear signaling from either the cytosol or membrane, promoting the activation of signaling pathways, such as MAPK or PI3K, which leads to increased cell motility and metastasis [7,8]. A complex crosstalk mechanism between the androgen receptor (AR) and the ER has also been shown, in some instances, to promote therapy resistance. Although it has been shown that AR coexpression is widely associated with a favorable prognosis due to the repression of ER activity, there are cases where high levels of AR in ER+ tumors have been shown to promote endocrine-therapy resistance [9] and EMT [10], which ultimately lead to more aggressive disease [11].

Coactivators have also been implicated in the ER’s metastatic function. For example, the ER coregulator AIB1 has been shown to promote metastasis via the activation of the matrix metalloproteases MMP2 and MMP9 [12]. Similarly, SRC1 promotes invasion and metastasis via TWIST upregulation [13]. A recent publication from Fu et. al. reports that the overexpression of the pioneer factor FOXA1, which facilitates ER binding to chromatin, drives a genome-wide transcriptional reprogramming that promotes metastasis [14]. These studies suggest that the modification of the ER function is common in metastatic ER+ disease. This modified function contradicts the ER’s classical role in many of the processes involved in the metastatic cascade. ER has been extensively reported to suppress invasion and metastasis by suppressing EMT [15,16], increasing adhesion [17], and promoting a ‘non-invasive’ cytoskeletal architecture [18]. In addition, ESR1 mutations have been associated with therapy-resistant metastatic disease. ESR1 mutations drive the ligand-independent growth of ER+ disease. Three common mutations, Y537S, Y537N, and D538G, were more prevalent in the endocrine-refractory metastatic tumors of patients but were absent from their matched primary tumor. These findings, along with the finding that ESR1 mutations are relatively absent from endocrine-naïve patients, suggest that mutations are acquired as a result of therapy and can lead to aggressive disease [19].

Clearly, the role of ER in metastasis is complex, and its function in promoting the migration and invasion of tumorigenic cells remains unresolved. In this study, we aimed to better understand the mechanisms by which ER promotes invasion by utilizing a unique ER-regulated invasion model that was previously described by our group. We found that the coactivation of ER and IKKβ, a key upstream kinase in the NFkB pathway, promoted a highly migratory and invasive phenotype in vitro, and an elevated metastatic burden in vivo [20]. However, the mechanism by which ER promoted invasion was unclear. Here, we demonstrate that ER is only expressed in a subset of invasive cells, and that classical ER activity is reduced in the invasive population. While ER predominantly regulated genes associated with proliferation in invasive cells, this function did not contribute to the invasive process, and, in fact, it acted to prevent invasion. Further analysis revealed additional putative functions for ER in invasive cells, such as vesicle organization and intracellular trafficking, which are known to be important in the invasive process. Importantly, both the proliferative and vesicle/intracellular trafficking gene signatures were enriched in metastatic cell populations from PDX tumors and were associated with aggressive disease and poor survival in breast cancer patients. We hypothesize that ER’s multifaceted function in invasive cells promotes aggressive disease and metastasis by both retaining proliferative ability, which is important for growth at the metastatic site, while aiding in the invasion process.

## 2. Materials and Methods

### 2.1. Reagents

E2, ICI (Fulvestrant), and DOX were purchased from Sigma, and RO-3306 was purchased from Cayman Chemical Company (Ann Arbor, MI, USA). The ERα antibody was purchased from Cell Signaling (# 8644), and the Alexa Fluor 594 antibody (# R37119) was purchased from ThemoFisher Scientific (Waltham, MA, USA). siRNA for FOXM1 (5′GCGCACGGCGGAAGAUGAA3′) was a gift from Dr. Pradip Raychaudhuri at UIC.

### 2.2. Cell Culture

DOX-inducible CA-IKKβ-expressing MCF-7 cells (CA-IKKβ MCF-7) were developed in the Frasor Laboratory and cultured as previously described [20]. Briefly, constitutively active IKKβ was subcloned into a puromycin-resistant Tet-On vector, pRetroX-Tight-Pur. This construct, along with a pVSV-G viral envelope expression vector, were transfected into GP2-293 packaging cells, and retroviral particles were generated. These were used to transduce MCF-7 cells. Single-cell clones were isolated and expanded to generate multiple new cell lines. Previous work describes appropriate control studies (i.e., DOX treatment of cells expressing the Tet-On vector only) and reproducibility between clonal lines [20]. Cells routinely tested negative for mycoplasma contamination and were authenticated using short-tandem-repeat (STR) profiling.

### 2.3. Invasion Assay

Falcon transwell inserts (8.0 μm, Corning) were coated with 10µg/cm^2^ rat-tail collagen I (Corning), and 80K cells were seeded per insert and allowed to invade for 24 h. For quantification, the underside of the inserts was fixed with 4% paraformaldehyde (PFA), then stained with 0.1% crystal violet. Noninvaded cells were removed from the inner surface, and inserts were imaged using the Celigo Imaging Cytometer (Nexcelom Bioscience). Images were processed using Image J. 

### 2.4. RNA Extraction and RT-qPCR

For isolation of cells from the Falcon transwell inserts for RNA extraction (RNAseq or scRNAseq), trypsin was added to the top and bottom of the inserts so both sides of the insert were submerged, but cells on the top and bottom remained separate due to the presence of the transwell membrane. After detachment, cells on top of the inserts (noninvaded) and cells on the underside of the insert (invaded) were collected separately and then centrifuged at 300× *g* for 3 min. RNA was isolated using RNeasy Plus Micro Kit (Qiagen), according to the manufacturer’s instructions. RT-qPCR was performed and analyzed as described previously, using 36B4 gene expression as an internal control [21]. 

### 2.5. Immunofluorescence

For immunofluorescence of invaded and noninvaded cells, cells on either the underside (invaded) or topside (noninvaded) of the insert were fixed with 4% PFA, then cells on the opposite side were removed. Cells were then permeabilized using 0.2% Triton X-100, blocked with casein, and then incubated with ERα antibody for 1 h. After washing, cells were incubated with a secondary antibody and washed. Membranes were then cut off the support and mounted cell-side-up onto glass slides using ProLongTM Gold Antifade Mountant with DAPI (ThemoFisher Scientific (Waltham, MA, USA). Images were acquired on a Leica DMi8 microscope at 63x magnification using the same acquisition settings. Quantification of ER+ cells was performed on 8 fields of view from 2 independent biological replicates.

### 2.6. siRNA-Mediated Knockdown

To test the role of FOXM1 in invasion, cells were transfected with siRNA targeting FOXM1 (siFOXM1), or nontargeting control siRNA (siNEG, ThemoFisher Scientific, Waltham, MA, USA), using DharmaFECT 1 (Dharmacon, Lafayette, CO, USA), before performing qPCR analysis or transwell invasion assays. 

### 2.7. RNAseq and Bioinformatics Analysis

RNA samples were quantified using NanoDrop™ One Spectrophotometer (Thermo Scientific) and analyzed for integrity using 4200 TapeStation (Agilent, Santa Clara, CA, USA). Sequencing libraries for Illumina sequencing were prepared using 100 ng of total RNA per sample. Library prep was carried out with the Universal Plus mRNA-Seq kit (NuGen, San Carlos, CA, USA), as written in the product manual. All intermediate purification steps, and final library purification, were carried out using Agencourt AMPure XP Beads (Beckman Coulter, Brea, CA, USA). Concentration of the final library pool was confirmed by qPCR and subjected to test sequencing in order to check sequencing efficiencies and accordingly adjust proportions of individual libraries. The pool was purified with Agencourt AMPure XP Beads (Beckman Coulter), quantified by qPCR, and run on a NovaSeq6000 SP flow cell (2 × 50 nt), at approximately 30 M clusters per sample, at the University of Illinois Roy J. Carver Biotechnology Center High-Throughput Sequencing and Genotyping Unit. Raw reads were aligned to human reference genome hg38 using STAR [22]. ENSEMBL genes were quantified using FeatureCounts [23]. Differential expression statistics were computed using edgeR [24] on raw expression counts with the exactTest function. *p*-values were adjusted for multiple testing using the false-discovery-rate (FDR) correction of Benjamini and Hochberg [25]. Data are available through Gene Expression Omnibus (GSE174189). To capture the most biologically relevant genes for further analysis, all significantly ICI-regulated genes in the invasive cells (INV+) and noninvasive cells (INV−) were identified using an FDR score of 0.05 or below. This provided our initial gene lists. These gene lists were further filtered for greater fold change and significance (0.5 log2 fold change and FDR < 0.001), and the genes specifically ICI-regulated in INV+ cells only made up Signature 1. To identify Signature 2, the initial gene lists had all proliferation genes that appeared in GSEA analysis of Signature 1 removed, then a fold change and significance filter were applied (0.25 log2 fold change and FDR < 0.01). Again, an overlap with INV- list identified genes specifically regulated in INV+ cells only. 

For Gene Ontology analysis, gene set enrichment analysis (GSEA) (Broad Institute, Cambridge, MA, USA) and ingenuity pathway analysis (IPA) (Qiagen, Hilden, Germany) were performed, following standard procedures recommended by the respective user guides. For GSEA, we used the Hallmarks and GO_biological process gene sets from the Molecular Signature Database (MsigDB v. 7.2) to compute enrichment, based on normalized enrichment scores. For IPA analysis, the Disease or Function analysis was used to predict activation of a function on the basis of the gene lists generated from the RNAseq data.

### 2.8. Single-Cell RNA Sequencing (scRNA-Seq) and Data Analysis

For scRNAseq, invaded cells were collected as described above and resuspended in PBS. A total of 2 technical replicates were prepared for analysis. Single-cell encapsulation was performed using the inDrop™ System from 1CellBio. All steps, including preparation of hydrogel beads, instrument loading/shut down, preparation of rection mixes, and RT reactions, were performed according to the manufacturer’s inDrop™ Single Cell Encapsulation and Reverse Transcription Protocol, Version 2.4. After encapsulation, cells were transferred to 1CellBio UV Cleavage Device to release photocleavable oligo linkers from the hydrogel beads, and RT reaction was performed. Sequencing libraries were constructed according to the inDrop Library Preparation Protocol v2.3. Sequencing was performed using NextSeq 500 by using the High Output Kit (75 cycles). Read format was as follows: Read1 50b, Read2 36b. Total cDNA read output was approximately 400Mb. Data are available through Gene Expression Omnibus (GSE174189).

Data were processed and aligned by the UIC Research Informatics Core. Briefly, raw R2 reads were mapped to the reference transcriptome (hg38 Ensembl gene sequences, exonic only) using BWA MEM. Cell barcodes and UMIs were extracted from R1 using a custom pipeline following OneCellBio adapter design for inDrop. Unique UMI counts were summed for each gene and each unique cell barcode. Only cell barcodes with >500 counts were included in the final counts table. The output was analyzed using the Seurat package (Version 3.2) in the R programming language. Raw counts were preprocessed to eliminate cells with low gene counts (<2000 genes) and high mitochondrial gene expression (>15% of total mapped reads). This resulted in 543 cells. The data were normalized by taking the expression of a feature for each cell and dividing it by the total expression. The result was multiplied by 10^4 and log transformed. Mitochondrial genes had the variance regressed out to minimize their effect on downstream analysis. Principal component analysis (PCA) was performed to measure the distance between cells. The number of principal components was determined using the JackStraw resampling method, and only the statistically significant (*p* < 0.05) components were used to create a KNN graph. A total of eight principal components were used to identify the number of clusters using the Louvain algorithm. To visualize the data in low-dimensional space, the Uniform Manifold Approximation and Projection (UMAP) reduction technique was used. 

### 2.9. Analysis of Patient-Derived Xenograft (PDX) Model of Metastasis

Single-cell RNA sequencing data of ER+ PDX-derived breast-cancer-cell-line primary tumors vs. metastatic sites were downloaded from Gene Expression Omnibus (GSE131007). Raw counts were preprocessed to eliminate cells with low gene counts (<2000 genes) and high mitochondrial gene expression. Downstream analysis was performed as described above. PDX cell lines UCD4 (invasive ductal carcinoma (IDC), derived from a pleural effusion), and UCD65 (IDC, lymph node metastasis) were used to study metastasis, as was previously described in [26]. Briefly, NSG female mice, 6–8 weeks of age at experiment initiation, were injected into the aorta with 1 × 106 viable cells resuspended in 0.1mL of PBS. For subcutaneous tumors, cells were placed into mammary fat pads suspended in 50% DMEM/50% Cultrex. All animals were supplemented with 17β-estradiol and were monitored by IVIS imaging biweekly, starting at 10 weeks postinjection, and sacrificed at 2–3 months postinjection or when moribund. 

### 2.10. Functional Enrichment Analysis and Pathway Enrichment Analysis

Differences in pathway signatures across cell clusters were determined using functional enrichment analysis (FEA) [27]. Briefly, a pathway-signature score was first computed as the mean z-scored expression for all genes in that pathway. Signatures tested were derived from MSigDB or were custom generated from the RNAseq data. Differences in pathway-signature scores were then compared across clusters (Wilcox test, *p* < 0.01 was considered significant). In addition, ROC analysis was used to estimate the accuracy of enrichment of a pathway signature within a particular cluster on the basis of calculated pathway-signature score per cluster. An area under the curve (AUC) >0.6 was considered an enrichment.

### 2.11. Public Data Mining

Data mining of the METABRIC (Molecular Taxonomy of Breast Cancer International Consortium) cohort was performed using cBioPortal, an open-access web-based resource for providing a tool to analyze patient tumor samples. Signature 1 and 2 downregulated genes were individually analyzed in the database against samples from the METABRIC cohort. Samples were stratified into 2 groups on the basis of presence of high expression (mRNA expression above 3 standard deviations from the mean) of any gene in the signature vs. low or no expression (mRNA expression below 3 standard deviations from the mean) in any gene from the signature, named positive (+) or negative (−) for signature alterations, respectively. Overall survival, molecular subtype (PAM50 and claudin-low), neoplasm histological grade, and patient’s vital status were analyzed between the altered and unaltered groups, according to the cBioPortal’s online instructions, and statistical significance was determined by chi-squared test.

### 2.12. Statistical Analysis

Unless otherwise specified, all data presented are representative of ≥2 biological replicates, with data displayed representing ≥2 technical replicates within the experiment. Error bars represent standard error of the mean for qPCR experiments and standard deviation from the mean for other experiments. Statistical significance was determined using one-way or two-way ANOVA where appropriate.

### 2.13. Data Availability

RNA-seq and scRNA-seq datasets generated in this manuscript will be made available through Gene Expression Omnibus (GSE174189) upon publication.

## 3. Results

### 3.1. ER Is Expressed and Active in Invasive Breast Cancer Cells

**Figure 1:**
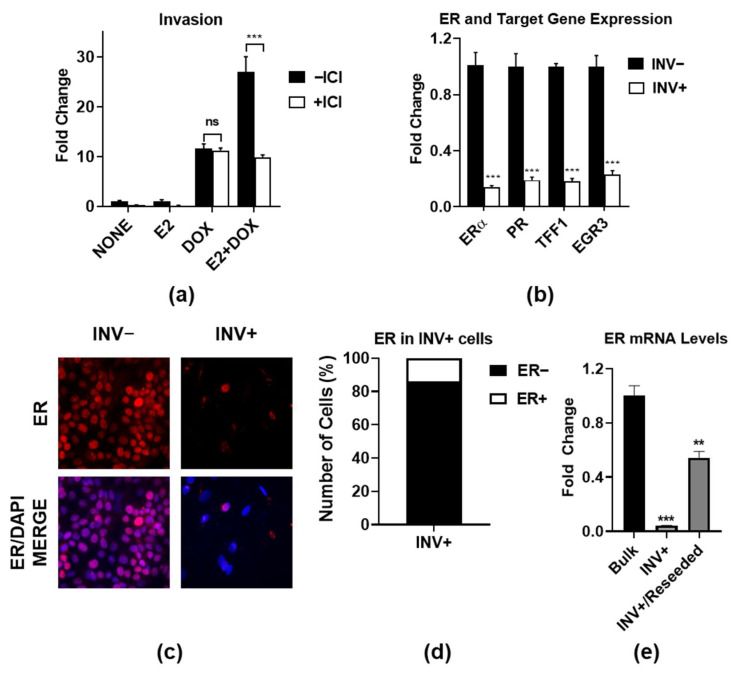
ER expression and activity in ER and IKKβ coactivated MCF-7 invasive vs. noninvasive cells. MCF-7-CA-IKKβ cells were treated with E2 (10 nM) +/− DOX (1 µg/mL) for 72 h, followed by +/− ICI 182,780 (ICI, 1 µM) for an additional 8 h. Equal numbers of cells were then seeded onto collagen-coated (10 µg/cm^2^) transwell inserts and allowed to invade for 24 h. (**a**) Cells were fixed, stained with crystal violet, and counted using Image J. Fold change vs. none was calculated. (**b**) Invaded (INV+) and noninvaded (INV−) cells were collected from the bottom and top of the insert, respectively, and RNA was extracted. ER and ER target gene mRNA expression were assessed by QPCR. Fold change in INV+ relative to INV− is shown. (**c**) ERα protein expression (red) was assessed in INV− and INV+ cells by immunofluorescence. Cell nuclei were counterstained with DAPI (blue). (**d**) The percentage of INV+ cells expressing detectable levels of ER protein was quantified from immunofluorescent images. (**e**) ER mRNA levels were determined by QPCR in INV+ cells immediately after the invasion assay or after INV+ cells were returned to standard culture conditions for 72 hr with continuous E2 + DOX treatment. Expression was compared to the bulk population of E2 + DOX-treated cells, which had not been put through the invasion assay. ** *p* < 0.01; *** *p* < 0.001; ns: not significant.

To confirm the role of ER in promoting invasion upon the constitutive activation of IKKβ (CA-IKKβ), we treated MCF-7 expressing a doxycycline (DOX)-inducible CA-IKKβ with estradiol (E2) and/or DOX, and then assessed invasion in the presence or absence of the ER antagonist, ICI 182,780 (ICI). ICI significantly reduced the invasion of E2 + DOX-treated cells, but not DOX-treated cells, suggesting that ER plays an active role in promoting invasion beyond the function of IKKβ (Figure 1a). To examine the ER expression and activity in invasive cells, we compared invaded cells (INV+) (i.e., those that have moved through the transwell insert) and noninvaded cells (INV−) (i.e., those that remain on the top of the transwell insert) following E2 + DOX treatment. We found that the expression of ER and its classical target genes, progesterone receptor (PR), trefoil factor 1 (TFF1), and early growth response protein 3 (EGR3), were all reduced in invaded cells vs. noninvaded cells (Figure 1b). An immunofluorescence analysis showed that ER expression was confined to a small percentage of invaded cells, whereas ER expression was detected in all noninvaded cells (Figure 1c,d). The reduced level of ER expression in invasive cells appears to be transient since the ER expression levels recovered after the invasive cells were reseeded in standard culture conditions and were allowed to recover (Figure 1e). These findings suggest that, despite a high degree of heterogeneity in the ER expression and reduced activity on classical target genes, ER is actively involved in promoting IKKβ-driven invasion.

### 3.2. ER-Regulated Genes Associated with Proliferation Prevent Invasion

**Figure 2:**
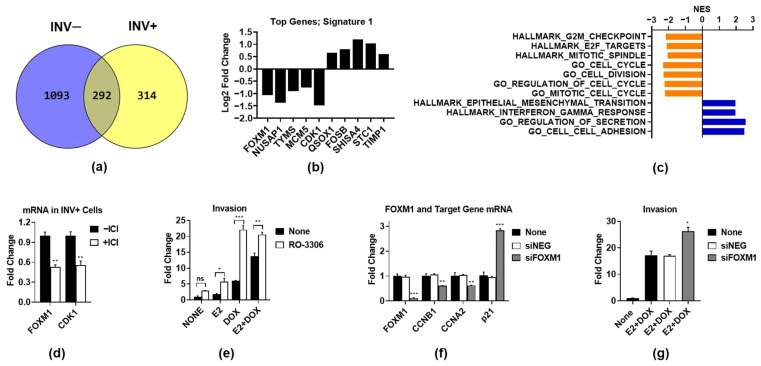
ER regulates a unique gene signature in invasive cells. RNA was isolated from INV+ and INV− cells as described in Figure 1, and RNA-seq was performed. (**a**) Genes regulated by ICI in INV− and INV+ cells were identified using a cutoff of FDR < 0.001 and log2 fold change (FC) of 0.5. Overlap of these gene lists in INV+ and INV− cells identified a 314-gene signature (Signature 1) specific to INV+ cells. (**b**) Log2 fold change expression is shown for the top up- and downregulated genes on the basis of FDR by ICI in INV+ cells only. (**c**) GSEA analysis of Signature 1 genes showing top significantly (*p* < 0.05) enriched Hallmark and GO_biological pathways and normalized enrichment scores from MsigDB v. 7.2. (**d**) FOXM1 and CDK1 mRNA was assessed by QPCR in E2 + DOX-treated INV+ cells treated with or without ICI. (**e**) MCF-7-CA-IKKβ cells were treated with E2 +/− DOX for 72 h, followed by the CDK1 inhibitor (CDKi) RO-3306 (5 µM) for a further 16 h, and invasion was assessed. (**f**,**g**) MCF-7-CA-IKKβ cells were transfected with an siRNA to FOXM1, or a scrambled negative control siRNA (siNEG), and then treated with E2 + DOX for 72 h. (**f**) QPCR for FOXM1 and FOXM1 target genes was performed, and (**g**) invasion was assessed. * *p* < 0.05; ** *p* < 0.01; *** *p* < 0.001; ns: not significant.

To identify genes that are regulated by ER during the invasion process, we performed an RNA-seq analysis of INV+ and INV− cells treated with or without ICI. In our first analysis, we identified the most biologically relevant ER-regulated genes by using a stringent cutoff for the fold change and significance (0.5 log2 fold change and FDR < 0.001). We identified a 314-gene signature, named Signature 1 (Supplemental Table S1), that was exclusively regulated by ICI in INV+ cells (Figure 2a). The top five ICI up- and downregulated genes in INV+ cells, on the basis of the FDR value, are shown (Figure 2b). The gene set enrichment analysis (GSEA) of the Signature 1 genes (Supplemental Table S2) showed that the ICI downregulated genes are associated with the cell cycle and proliferation (Figure 2c). In contrast, genes upregulated by ICI are involved in EMT and cell-adhesion and secretion processes, which was surprising, as these processes are frequently upregulated in invasive cells, and yet ICI blocks invasion. 

As the RNA-seq analyses implicate ER-regulated proliferation as playing a role in invasion, we investigated the function of cell-cycle-specific genes. As indicated in Figure 2b, FOXM1 and CDK1 are two of the top ICI downregulated genes from Signature 1, which we confirmed by QPCR in independent samples (Figure 2d). To assess the role of CDK1 in invasion, we used RO-3306, which is an inhibitor of CDK1 activity, which we found increased rather than decreased invasion in all treatment groups (Figure 2e). We then assessed the role of FOXM1 using siRNA, which efficiently knocked down FOXM1 and altered the expression of FOXM1 target genes (Figure 2f). Similar to CDK1 inhibition, the FOXM1 knockdown significantly increased the invasion of E2 + DOX-treated cells, compared to the untreated and scrambled siNEG control cells (Figure 2g). Taken together, these data suggest that, although ER drives the expression of proliferation-associated genes in invasive cells, this process does not promote invasion, but instead suppresses it. Moreover, our findings suggest that blocking ER-driven proliferation can increase invasion.

### 3.3. ER Regulates Genes Associated with Organelle Assembly and Vesicle Trafficking in Invasive Cells

**Figure 3:**
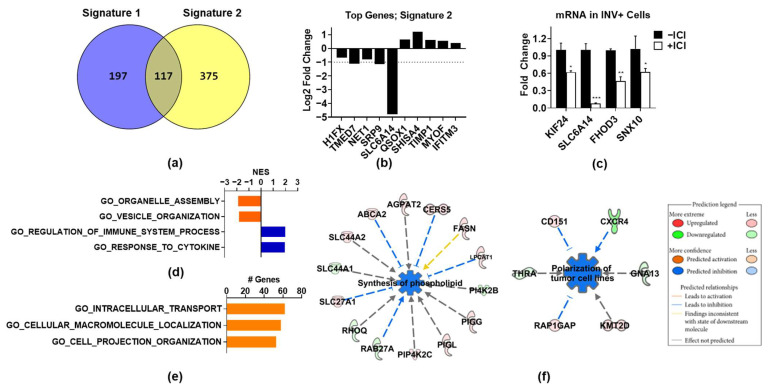
ER regulates the expression of genes associated with intracellular trafficking in invasive cells, and disruption/inhibition of vesicle formation. (**a**) Signature 2 genes were identified by applying a cutoff of 0.25 log2 fold change and FDR < 0.01 to all genes significantly regulated (FDR < 0.05) by ICI in INV− and INV+ cells, then genes exclusively regulated in INV+ cells had cell-cycle-associated genes removed. Overlap with Signature 1 is shown. (**b**) Log2 FC expression of top regulated genes. (**c**) Validation of top regulated genes by ICI in INV+ cells by QPCR. Statistical significance was determined using one-way ANOVA vs. -ICI group. * *p* < 0.05; ** *p* < 0.01; *** *p* < 0.001. (**d**) GSEA analysis of Signature 2 genes showing top enriched Hallmark and GO_biological pathways (*p* < 0.05). (**e**) Gene ontology categories with the highest number of genes present from Signature 2. (**f**) IPA-downstream-effect-analysis networks associated with cellular functions showing two highly inhibited functions by ICI in Signature 2; synthesis of phospholipid (*p* = 7.61 × 10^−3^) and polarization of tumor cell lines (*p* = 1.12 × 10^−3^). Edges and nodes are color-coded on the basis of the predicted relationship, as indicated in the legend.

Since Signature 1 is heavily dominated by proliferation-associated genes, which do not explain how ER promotes invasion, we repeated the bioinformatics analysis after removing these genes and applying a less stringent cutoff (0.25 log2 fold change and FDR < 0.01). This generated a 492-gene signature (Supplemental Table S3), named Signature 2, which contained 375 distinct genes and 117 genes overlapping with Signature 1 (Figure 3a). Since the gene set enrichment analysis gives a probability that a gene signature is enriched for a certain process but does not mean that all genes in the signature are involved in that process, we decided to include the overlapping genes not related to proliferation in Signature 2, since they may function to promote invasion. Some of the top regulated genes on the basis of FDR are shown in Figure 3b, several of which were validated by QPCR as ICI-regulated in invasive cells (Figure 3d). The GSEA analysis of Signature 2 (Supplemental Table S4) revealed that genes upregulated by ICI were related to the regulation of cytokines and immune processes (Figure 3d), which may reflect the ability of ER to suppress the NFκB pathway on classical target genes [28,29]. The genes downregulated by ICI, in contrast, were significantly associated with organelle and vesicle assembly and organization (Figure 3c). Additionally, numerous genes found in Signature 2 were related to essential biological processes, such as intracellular transport, macromolecular localization, and cell projection organization, although these signatures did not achieve significance (Figure 3e). A further interrogation of the Signature 2 genes using IPA software revealed the synthesis of phospholipids and the polarization of tumor cells as significantly downregulated by ICI (Figure 3f). While each of these ICI-regulated pathways and cellular processes can be important for cell invasion (see Discussion), the broad biological activity and essential nature of these pathways made their role in ER-promoted invasion difficult to assess.

### 3.4. Expression of Signatures 1 and 2 in Invasive Cell Subpopulations

**Figure 4:**
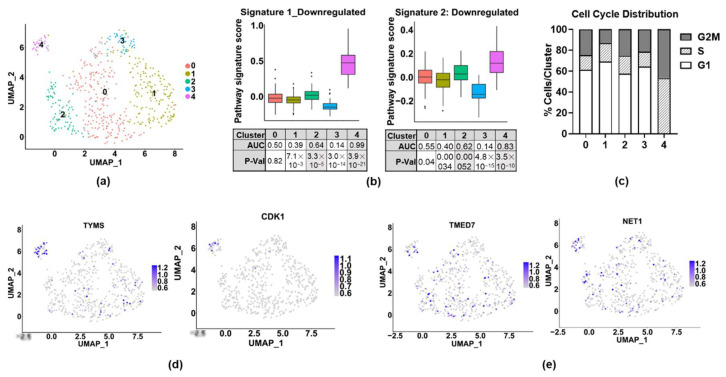
ER activity and gene signatures in invasive cell subpopulations. MCF-7-CA-IKKβ cells were treated with E2 + DOX for 72 h, then seeded onto collagen-coated (10 µg/cm^2^) transwell inserts. After 24 h, invaded (INV+) cells were collected from the bottom of the insert and sequenced using inDrop scRNA-Seq. (**a**) Bi-dimensional representation of single-cell transcriptomes of 543 MCF-7 CA-IKKβ invasive cells visualized in a UMAP plot. (**b**) Box plots to show enrichment of Signature 1 and Signature 2 genes. ROC analysis was used to generate AUC values, which represent signature enrichment in each cluster of INV+ cells. Significance of enrichment was tested by Wilcox test. AUC > 0.6 and *p* < 0.01 were considered significant. (**c**) Cell-cycle analysis of scRNA-seq data was conducted using Cell-Cycle Scoring and Regression vignette [33], provided by Seurat package v. 3.1. (**d**) Feature plots for two Signature 1 genes. (**e**) Feature plots for two Signature 2 genes.

On the basis of the heterogeneity of the ER expression, we also decided to analyze invasive cells by using single-cell RNA-sequencing (scRNA-seq). A total of 543 INV+ cells were analyzed and were found to cluster into five subpopulations on the basis of their transcriptomes (Figure 4a). The full differentially expressed gene lists for each cluster are shown in Supplemental Table S5. ER expression was found in 4% of the cells, but below the level of detection when QC cutoffs were applied to the data, which may be due to low expression or to technical issues, such as mRNA degradation after cell lysis, lower capture, variable amplification efficiency, or varied sequencing depth [30]. To assess the ER activity, we used the Hallmark Estrogen Response Early gene set from MsigDB [31,32] and found that it was not enriched in any cluster (data not shown), which supports the idea that classical ER activity is low in invasive cell populations. To examine ICI-regulated gene signatures in INV+ cell populations, we focused only on genes downregulated by ICI. This was because the RNA-seq and qPCR data (not shown) indicate that ICI upregulates (and therefore ER suppresses) some NFκB target genes and EMT-related genes. As EMT is typically associated with increased invasion, it is therefore unlikely that these genes contribute to the ER’s role in promoting invasion. We found a highly significant enrichment of both Signatures 1 and 2 in Cluster 4, and a moderate enrichment in Cluster 2 (Figure 4b). Since Signature 1 was enriched for proliferation-associated genes, we assessed the proliferative status of each cell cluster by using a cell-cycle scoring algorithm [33]. We found that the cells in Cluster 4 were in either the S (53%) or G2/M (47%) phase, suggesting that this invasive cell population is also actively proliferating (Figure 4c). We also visualized the expression of the proliferation-associated genes from Signature 1 (TYMS, CDK1) and confirmed that they were largely localized to Cluster 4 (Figure 4d). In contrast, the expression of the top genes from Signature 2 (TMED7 and NET1) appeared to be more broadly expressed, and primarily in Clusters 2 and 4 (Figure 4e). To determine how overlapping genes between Signatures 1 and 2 contribute to each signature, an analysis was run with the overlapping genes removed (Supplemental Figure S1). Our findings suggest, as expected, that the overlapping genes made no difference in the enrichment of Signature 1 in Cluster 4, as these genes are not involved in proliferation. In contrast, the overlapping genes make a strong contribution to the enrichment of Signature 2, and particularly in Cluster 2, which supports the inclusion of these genes in Signature 2, as well as their potential role in invasion. Taken together, these findings suggest that ERs may play unique roles in the subpopulations of cells to promote either proliferation or invasion. 

### 3.5. ER-Regulated Gene Signatures Are Associated with Metastasis and Poor Patient Outcome

**Figure 5:**
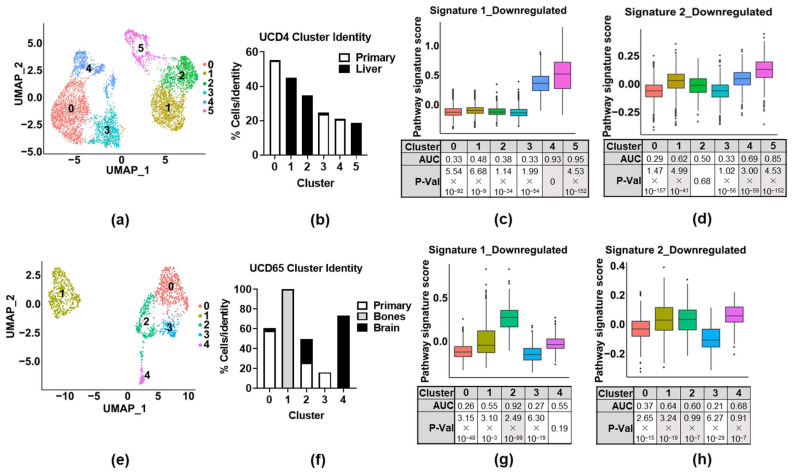
Invasive signatures are enriched in primary and metastatic PDX tumor cell populations. (**a**,**e**) UMAP plot showing bi-dimensional representation of single-cell transcriptomes of the PDX-derived breast cancer cell lines: (**a**) UCD4 and (**e**) UCD65, representing primary tumors and tumors in various sites of metastasis. (**b**,**f**) The contributions of each cell’s identity (i.e., primary or metastatic tumor) to each cluster (% of total number of cells in original identity) in each dataset. (**c**,**d**). Box plot and FEA for Signatures 1 and 2 in the UCD4 dataset. (**g**,**h**). Box plot and FEA for Signatures 1 and 2 in the UCD65 dataset.

To determine whether either Signature 1 (i.e., proliferation) or Signature 2 (i.e., organelle/vesicle assembly) are associated with aggressive disease, we first interrogated publicly available scRNA-seq datasets from metastatic-ER+-patient-derived xenograft (PDX) models [26] (GSE131007). Two PDX cell lines, UCD4 and UCD65, were used for the analysis. For each model, primary or metastatic tumors were harvested for scRNA-seq analysis, with bone and brain metastases collected from UCD65, and liver metastases collected from UCD4. The clustering of the datasets for each model was performed (Figure 5a,e), and the identity of the cells in each cluster was defined by their source, either coming from a primary tumor or from a metastatic site (Figure 5b,f). In the dataset from the PDX model UCD4, both Signatures 1 and 2 were highly enriched in Clusters 4 and 5, which consisted of cells found in the primary tumor or liver metastases, respectively (Figure 5c,d). Signature 2 was also highly enriched in Cluster 1, which was found primarily in liver metastases. In the PDX UCD65 dataset, Signature 1 was found to be enriched only in Cluster 2, which consisted of cells found in both brain and primary tumors (Figure 5g). In contrast, Signature 2 was found to be enriched in Cluster 1 (found in bone metastases), Cluster 2 (primary and brain metastases), and Cluster 4 (brain metastases). Again, overlapping genes between the two signatures played a key role for Signature 2 enrichment but not Signature 1 (Supplemental Figure S1). Thus, both signatures were found to be enriched in different cell populations of primary and metastatic PDX tumors, suggesting the potential relevance of these ER-regulated invasive gene signatures to the metastatic process, as well as supporting the concept that ER may function differently in different cell subpopulations. 

**Figure 6:**
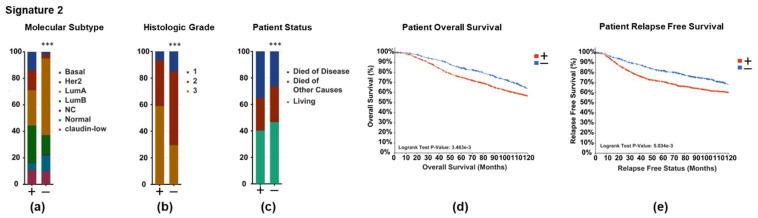
Invasive Signature 2 is associated with poor outcome in ER+ breast cancer patients. cBioPortal for Cancer Genomics was used to query 1904 patients with breast tumors from the METABRIC cohort for genes downregulated by ICI in Signature 2. Patients were stratified on the basis of high expression (+ = mRNA expression 3 standard deviations above the mean) or low expression (− = mRNA expression below 3 standard deviations below the mean) of genes downregulated by ICI from Signature 2, normalized to the reference population, where each gene is compared to the mean level of expression of all samples in the cohort. The distributions of the (**a**) molecular subtype, (**b**) histologic grade, (**c**) patient status, (**d**) patient overall survival, (**e**) and relapse-free survival between (+) and (−) groups are displayed. Statistical significance was determined using (**a**–**c**) chi-squared test or (**d**) log-rank test. *** *p* < 0.01.

Finally, to determine if ER-regulated gene signatures are associated with patient outcome, we investigated the expression of Signatures 1 and 2 in patient samples for correlations with the patient outcome and clinical parameters. By using cBioPortal for Cancer Genomics, we interrogated data from the METABRIC study containing 1904 primary breast tumors [34,35]. Each tumor was scored positive if the expression of any gene from the signature was high (above 3 standard deviations above the mean) or was scored negative if no genes in the signature were highly expressed (below 3 standard deviations above the mean). As expected, the proliferative Signature 1 was associated with more aggressive disease and poor survival (Supplementary Figure S2). Surprisingly, the non-proliferative Signature 2 was also significantly more likely to be found in the tumors of a Luminal B rather than Luminal A molecular subtype (Figure 6a), with a higher histological grade (Figure 6b), and associated with an increased risk of death from disease (Figure 6c), worse overall survival (Figure 6d), and reduced relapse-free survival (Figure 6e). Full statistical analyses for each signature are presented in Supplemental Tables S6 and S7. The same analysis was run for the signatures without overlapping genes (Supplemental Figure S3), which confirmed the importance of these genes for Signature 2′s clinical relevance. Full statistical analyses for each signature with the overlap genes removed are presented in Supplemental Tables S8 and S9. These findings suggest that a novel ER-regulated gene signature found in a subset of invasive cells is associated with more aggressive disease and poor patient outcome, independently of ER’s known role in proliferation. 

## 4. Discussion

In attempting to determine the role of ER in promoting the invasion of breast cancer cells, we found two relatively distinct gene signatures that are unique to invasive cells that were regulated by ER. Signature 1 is enriched with ER-regulated cell-cycle genes and proliferation, but this function of ER contributed to reduced rather than enhanced cell invasion. While this does not explain how ER promotes invasion when IKKβ is activated, it may explain why ER+ breast cancer cells tend to be less invasive. In contrast, Signature 2 is enriched with genes that are involved in broad cellular functions that are known to be associated with invasion, such as vesicle organization and formation, intracellular trafficking, projection formation, and cellular polarization. Importantly, both signatures were identified as enriched in PDX metastatic tumor cell populations and were correlated with worse clinicopathological features and poor patient survival. Moreover, of interest is that ER expression and function is limited to the subpopulations of invasion cells, suggesting that cellular heterogeneity may be an underlying factor in ER’s divergent functions in invasive cells. Moreover, these functions of ER appear to be uncovered only with the coactivation of IKKβ, which suggests that the NFkB pathway may reprogram the ER function to promote invasion and metastasis, which is consistent with our previous work suggesting that ER and NFkB can work together through transcriptional cooperativity to promote more aggressive disease features [20,36,37,38,39,40,41]. 

The most widely studied role of ER in driving breast tumorigenesis is a transcriptional regulator for genes associated with promoting the proliferation and inhibition of apoptosis, which leads to tumor growth. As expected, this proliferative function of ER was identified in Signature 1, but its expression was confined to a subset of invasive cells. In this invasive subpopulation of cells, ER retained the ability to control proliferation, as shown by the regulation of key players, such as FOXM1 and CDK1, but this process was actually inhibitory of invasion, and is suggestive of the widely accepted ‘go or grow’ theory [42], where cells will either proliferate or invade, but cannot perform these processes at the same time. Despite FOXM1 having a multifaceted function, including the promotion of invasion [43], in this cellular context, it appears to suppress invasion and likely has a proliferative function, alongside the majority of genes in Signature 1. The fact that a subset of invasive cells became highly proliferative may be explained by the ability of cells to dynamically switch between phenotypes, with the cells switching back to proliferation driven by ER after the invasion had occurred. Qian et. al. describe how p21/CIP1 controlled the reciprocal switching between proliferation and invasion in breast cancer cells as a mechanism that promotes metastasis [44]. Another study shows that glioblastoma cells with activated HGF/SF–Met signaling were both proliferative and invasive, and these phenotypes appeared to activate distinct downstream signaling events, independently from each other [45]. Phenotypic switching can lead to a high degree of heterogeneity in cancer cells, which is implicated in metastasis [46], which suggests that although Signature 1 genes may not promote invasion, they may still be significant in promoting metastasis.

In contrast to ER’s role in proliferation, its function in driving other features of aggressive disease, such as invasion and metastasis, are less clear. The genes in Signature 2 suggest that the role of ER in invasion is not via typical invasive gene programs, such as EMT or basal gene regulation. This is in line with the literature, which describes how ER suppresses EMT [15]. A common mechanism for this is via the suppression of NFkB [47], which we have observed in our RNA-seq data, with many IKKβ-regulated EMT genes suppressed by ER (data not shown). This is an interesting finding, as, despite EMT normally driving invasion, ER appears to drive invasion by alternate means. ER’s role in invasion points towards processes such as vesicle synthesis and trafficking. Protein trafficking via vesicles requires a number of highly regulated processes, from the initial formation of vesicles, through their transportation and delivery of cargo to specific cellular locations. Our data show that phospholipid synthesis may be inhibited by ICI, suggesting that ER may contribute to the formation of phospholipid bilayers that make up vesicles, which enhances their availability for protein trafficking. Others have linked ER to vesicle formation, including one group that identified 147 estrogen-regulated genes involved in vesicle trafficking [48], and another that showed that the formation of giant intracellular and extracellular vesicles was mediated by ER [49]. In addition to their formation, ER may also regulate proteins involved in the physical trafficking of vesicles, which is a vital process required for cell migration and invasion, as they transport cargo required at specific cell sites. For example, the transportation of MMP proteins to the membrane, and the secretion out of the cell in extracellular vesicles, are crucial for extracellular matrix degradation. Studies have shown that MT1-MMP trafficking is mediated by RaBGTPases, resulting in the vesicular delivery of MT1-MMP to invadopodia and, consequently, the degradation of ECM and increased invasion [50]. Similarly, a Rab siRNA library screen was able to identify that RAB40b was required for the secretion of MMP2/9 and regulated their trafficking within secretory vesicles during invadopodia formation, resulting in matrix degradation [51]. Several RaBGTPases appeared in Signature 2 as ER-regulated, including RAB27A and RAB23. Vesical transport is also required for other modulators of cell invasion, such as integrins, which function to anchor cells to the extracellular matrix and are rapidly cycled and transported to the cell surface, allowing cells to become motile [52]. The endocytosis of integrins has been shown to occur via Rab proteins, with Rab4 and Rab11 implicated in the trafficking of integrin containing endosomes towards the leading edge of migrating cells [53]. Clearly, both phospholipid and vesicle synthesis/trafficking proteins are implicated in promoting invasion, and we have shown that ER may contribute to both. Dissecting the role of these processes in invasion, however, has proven difficult due to their importance in cell survival and function. Interestingly, the RNAseq data show an increase in the expression levels of ‘cargo’ proteins, such as MMPs and integrins in invasive cells, which are commonly crucial for invasive processes, mediated through IKKβ activation rather than ER (data not shown). In fact, with so many typically invasive genes identified to be regulated by IKKβ alone, ER may actually function to complement how these proteins are utilized. This may suggest an interesting synergistic co-operativity between ER-regulating vesicle trafficking and IKKβ’s regulation of potential invasive cargo. 

While our studies suggest that ER can have both invasion-promoting and -suppressing functions, the scRNA-seq findings suggest a high degree of heterogeneity in the ER function in invasive cell populations. In particular, the ER-regulated genes associated with proliferation were largely confined to a proliferative subpopulation of cells, whereas the ER-regulated genes associated with intracellular trafficking, vesicle organization, phospholipid synthesis, and cellular polarization showed more broad expression across invasive cell subpopulations, suggesting a distinct heterogeneity in ER activity. The role of cellular heterogeneity has emerged as an important driver of tumor robustness [54], allowing cells to survive adverse environments, such as in circulation or the metastatic niche. To further strengthen the association of invasive cells with metastasis, we also looked for the enrichment of stemness, which is often implicated in metastasis and aggressive phenotypes. We found that multiple cancer stem signatures from MsigDB were enriched in Cluster 4 (Supplemental Table S10)**,** the same cluster as the Signature 1 enrichment. The enrichment of stemness in this subpopulation further supports the phenotypic heterogenicity in these INV+ cells, which potentially leads to a more aggressive population of cells predisposed to metastasis. Heterogeneity has been shown to promote metastasis. For example, Obradovic and colleagues demonstrated that the activation of the glucocorticoid receptor increased tumor heterogeneity and was critical for metastatic colonization [55]. Another recent study highlights the expression of vesicle-trafficking genes in heterogenous tumors, which played a role in promoting metastasis in colon cancer [56], further implicating our findings that ER may contribute to metastasis by regulating vesicle trafficking. Importantly, our data show that the enrichment of both ER-regulated gene signatures was observed in the cell populations of metastatic PDX tumors, as well as in the more aggressive ER+ breast tumors of patients, despite Signature 1 not correlating with invasive potential, which further suggests a role for heterogenic ER activity in metastasis. 

## 5. Conclusions

The mechanism by which IKKβ reprograms the ER function to promote aggressive disease, and how ER-driven heterogeneity promotes invasion and metastasis, remain unclear; however, the dissection of these functions could shed light on how ER+ tumors become aggressive, and could suggest possible therapeutic interventions. Although an absence of in vivo studies may be a limitation of this study, our previous studies have shown that IKKβ activation alone was not adequate to promote the formation of metastatic tumors in mice, despite promoting invasion in vitro, which suggests that ER’s role is crucial for promoting metastasis [20]. Here, we demonstrate the distinct heterogeneity in the ER signaling in invasive cells and suggest that this heterogeneity may underlie the differential effects of ER on invasion and increased metastasis in vivo. The correlation of both ER-regulated gene signatures with metastasis in PDX models, aggressive clinical features in human disease, and poor prognosis in breast cancer patients suggest the clinical importance of both ER-regulated gene signatures. These may be particularly relevant in tumors where NFkB activation is implicated (for example, as observed in some more aggressive Luminal B tumors). Moreover, as scRNA-seq technology begins to translate into routine disease management, we expect that the signatures that are derived from subpopulations of cells, such as the invasive populations used here, will become increasingly useful in predicting tumors predisposed to metastasis through the identification of aggressive subpopulations of cells.

## Data Availability

The RNA-seq and scRNA-seq datasets generated in this manuscript will be made available through the Gene Expression Omnibus (GSE174189) upon publication.

## Supplementary Materials

The following supporting information can be downloaded at: https://www.mdpi.com/article/10.3390/cancers14122848/s1, Figure S1: Overlapping genes between the signatures do not impact Signature 1 cluster enrichment but are important for cluster enrichment of Signature 2 in invasive cells and primary and metastatic PDX tumor cell populations; Figure S2: Signature 1 is associated with poor outcome in ER+ breast cancer patients; Figure S3: Overlapping genes between Signatures 1 and 2 do not contribute to association of Signature 1 with clinical outcome, but do contribute to the association of Signature 2 with clinical outcome; Table S1: List of ER-regulated genes forming Signature 1 in ER and IKKβ coactivated invasive MCF-7 cells; Table S2: GSEA analysis of Signature 1 genes showing enriched Hallmark and gene ontology gene sets; Table S3: List of ER-regulated genes forming Signature 2 in ER and IKKβ coactivated invasive MCF-7 cells; Table S4: GSEA analysis of Signature 2 genes showing enriched Hallmark and gene ontology gene sets; Table S5: Differentially expressed genes for each cluster identified in scRNA-seq dataset derived from invasive cells. Table S6: Statistical analysis of clinical parameters associated with Signature 1; Table S7: Statistical analysis of clinical parameters associated with Signature 2; Table S8: Statistical analysis of clinical parameters associated with Signature 1, where the genes overlapping with Signature 2 were removed; Table S9: Statistical analysis of clinical parameters associated with Signature 2, where the genes overlapping with Signature 1 were removed; Table S10: Functional enrichment analysis for stem-cell-associated gene signatures in invasive cell populations.
