# Supplementary material for: Estrogen Receptor-Regulated Gene Signatures in Invasive Breast Cancer Cells and Aggressive Breast Tumors
Source: Cancers (Basel). 2022 Jun 9;14(12):2848. doi: 10.3390/cancers14122848 (PMC9221274; doi:10.3390/cancers14122848)
Supplement: Supplementary file 1 [file cancers-14-02848-s001.zip › Supplemental Fig S1-S3_Final.pdf]

Supplementary Figure S1

A

INV+

Signature 1 _DN; Overlap Removed

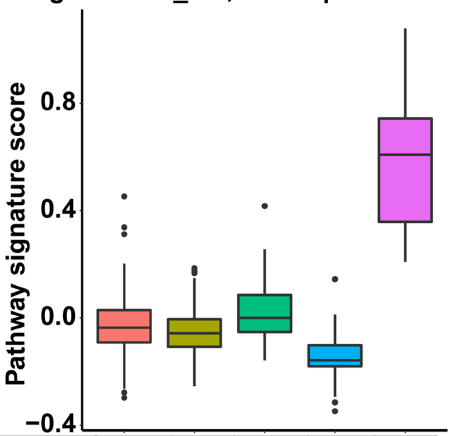

Cluster	0	1	2	3	4
AUC	0.50	0.40	0.64	0.16	0.99
P-Val	0.88	2.00×10^{-4}	3.00×10^{-5}	1.70×10^{-13}	3.30×10^{-21}

B

INV+

Signature 2_DN; Overlap Removed

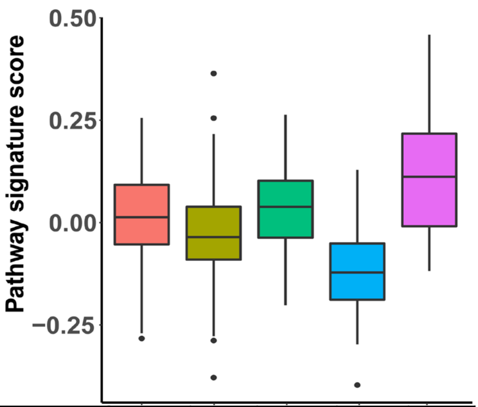

Cluster	0	1	2	3	4
AUC	0.58	0.38	0.60	0.21	0.73
P-Val	1.3×10^{-4}	1.19×10^{-5}	3.20×10^{-4}	5.05×10^{-10}	1.43×10^{-5}

C

UCD65

Siganture 1 DN_Overlap Removed

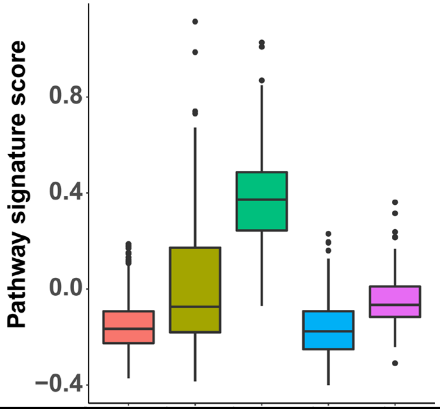

Cluster	0	1	2	3	4
AUC	0.26	0.54	0.94	0.30	0.55
P-Val	1.25×10^{-52}	9.82×10^{-3}	1.62×10^{-103}	8.80×10^{-15}	1.90×10^{-1}

D

UCD65

Siganture 2_DN_Overlap Removed

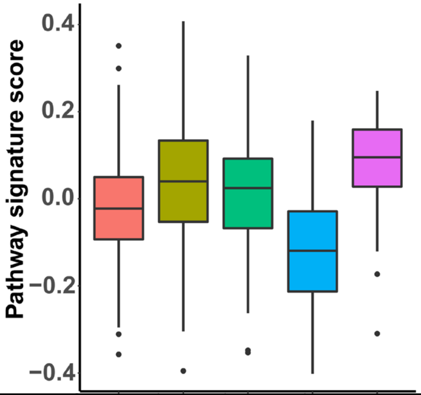

Cluster	0	1	2	3	4
AUC	0.42	0.62	0.53	0.23	0.70
P-Val	1.97×10^{-6}	7.03×10^{-15}	1.42×10^{-1}	1.06×10^{-25}	1.20×10^{-7}

E

UCD4

Signature 1_DN: Overlap Removed

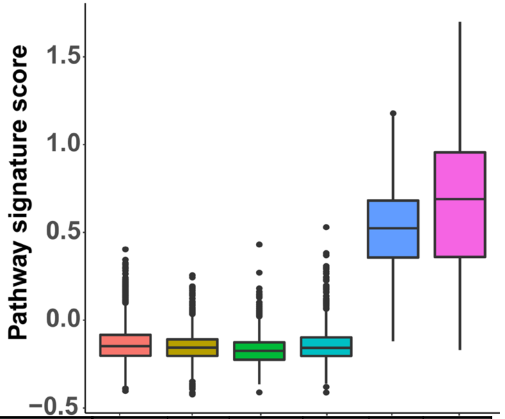

Cluster	0	1	2	3	4	5
AUC	0.40	0.37	0.32	0.40	0.93	0.95
P-Val	1.91×10^{-37}	6.15×10^{-47}	1.83×10^{-73}	2.91×10^{-22}	0.0	1.15×10^{-252}

F

UCD4

Signature 2_DN: Overlap Removed

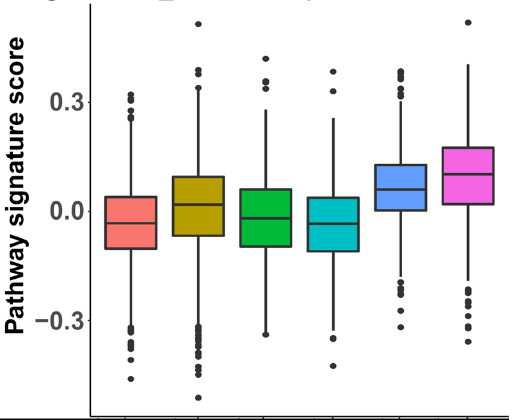

Cluster	0	1	2	3	4	5
AUC	0.39	0.54	0.44	0.40	0.67	0.74
P-Val	2.07×10^{-44}	6.81×10^{-7}	2.75×10^{-9}	2.38×10^{-21}	1.18×10^{-50}	4.25×10^{-74}

Supplementary figure S1. Overlapping genes between the signatures do not impact signature 1 cluster enrichment but are important for cluster enrichment of Signature 2 in invasive cells and primary and metastatic PDX tumor cell populations.

MCF-7-CA-IKKβ cells were treated with E2+DOX for 72 hours, then seeded onto collagen coated (10 μg/cm2) transwell inserts. After 24 hours, invaded (INV+) cells were collected from the bottom of the insert and sequenced using inDrop scRNA-Seq. Box plots to show enrichment of Signature 1 (A) without overlapping genes with signature 2, and signature 2 (B) without overlapping genes with signature 1. Enrichment of signature 1 and 2 without overlapping genes was analyzed in primary and metastatic populations of PDX derived breast cancer cell lines UCD65 and UCD4. Box plots and FEA for signature 1 without overlap (C, E) and signature 2 without overlap (D, F) in UCD65 and UCD4 respectively. ROC analysis was used to generate AUC values, which represent signature enrichment in each cluster of INV+ or PDX cells. Significance of enrichment was tested by Wilcox test. $AUC>0.6$ and $P<0.01$ was considered significant. PDX derived breast cancer

Supplementary Figure S2

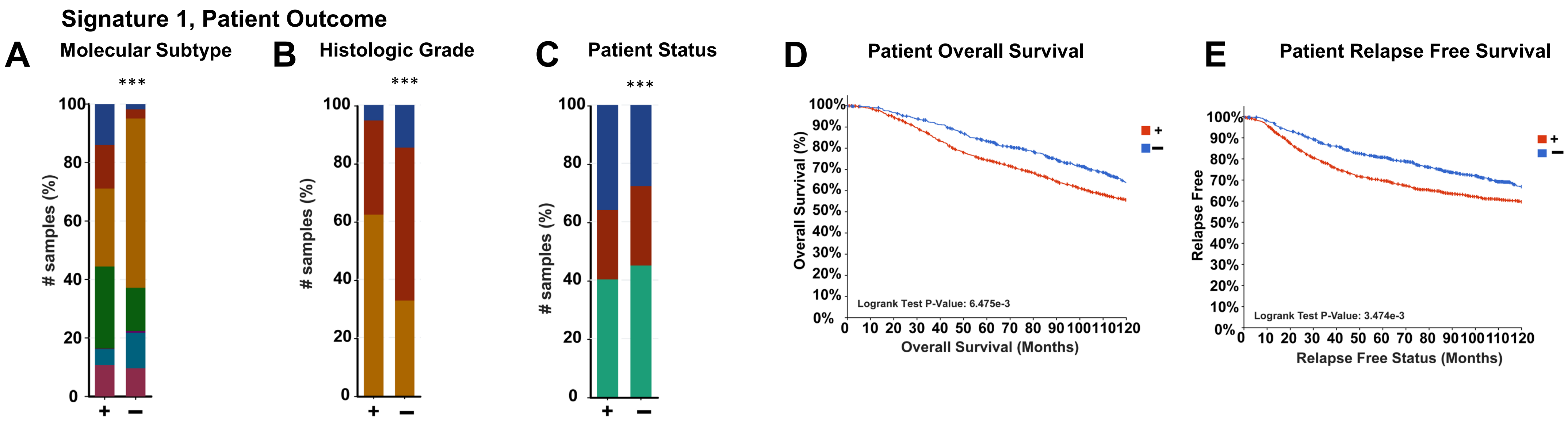

Supplementary figure S2. Signature 1 is associated with poor outcome in ER+ breast cancer patients.
cBioPortal for Cancer Genomics was used to query 1904 patients with breast tumors from the METABRIC cohort for genes downregulated by ICI in signature 1. Patients were stratified based on high expression (+ = mRNA expression 3 standard deviations above the mean) or low expression (- = mRNA expression below 3 standard deviations below the mean) of genes downregulated by ICI from signature 1, normalized to the reference population where each gene is compared to the mean level of expression of all samples in the cohort. The distribution of the (A) molecular subtype, (B) histologic grade, (C) patient status, (D) patient overall survival, and relapse free survival (E) between (+) and (-) groups were displayed. Statistical significance was determined using chi-squared test (A, B and C) or log-rank test (D). ***P<0.01.

Supplementary Figure S3

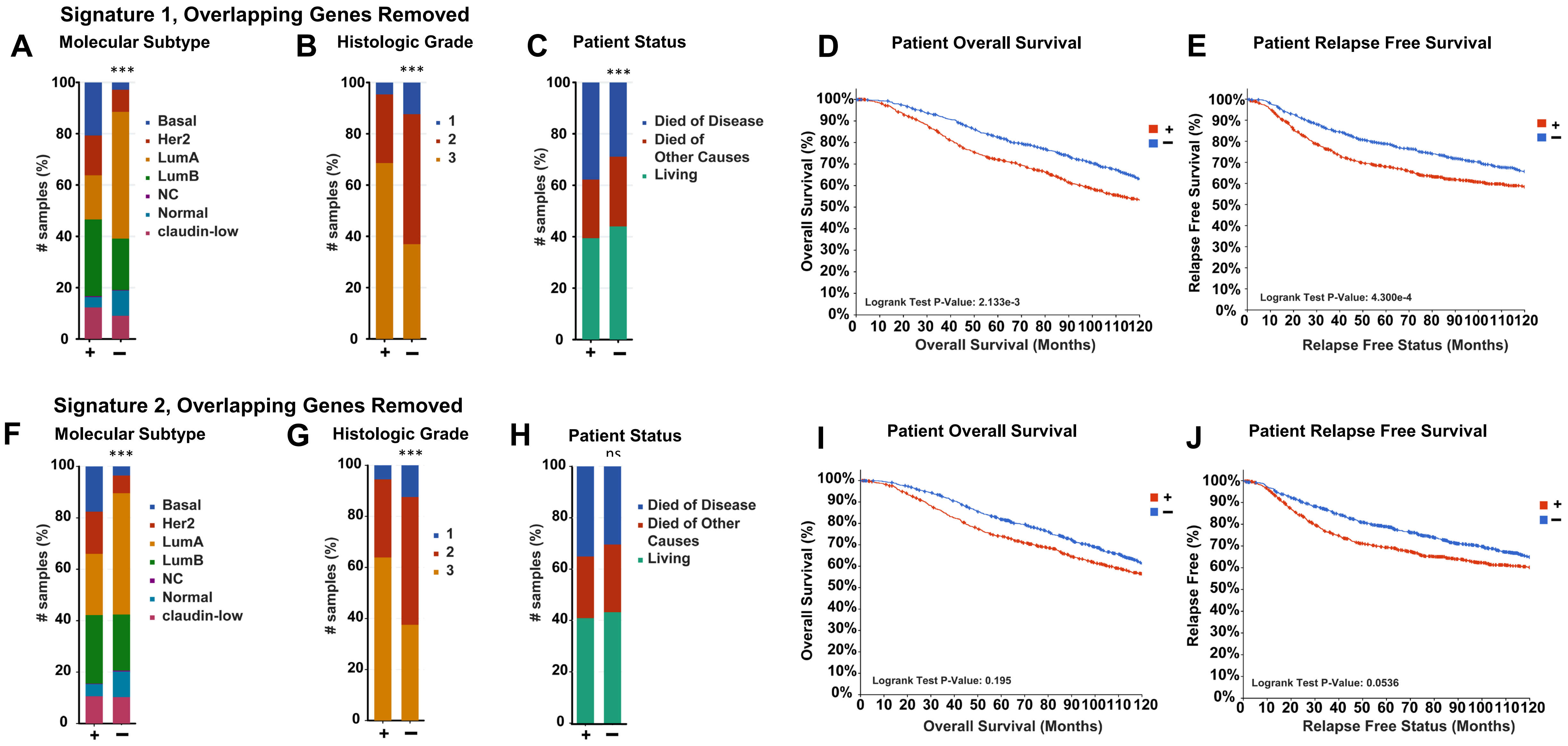

Supplementary figure S3. Overlapping genes between signature 1 and 2 do not contribute to association of signature 1 with clinical outcome, but do contribute to the association of signature 2 with clinical outcome. cBioPortal for Cancer Genomics was used to query 1904 patients with breast tumors from the METABRIC cohort for genes downregulated by ICI in signature 1 minus the genes that overlap genes with signature 2, and signature 2 minus the overlap genes with signature 1. Patients were stratified based on high expression (+ = mRNA expression 3 standard deviations above the mean) or low expression (- = mRNA expression below 3 standard deviations below the mean) of genes, normalized to the reference population where each gene is compared to the mean level of expression of all samples in the cohort. The distribution of the (A, F) molecular subtype, (B, G) histologic grade, (C, H) patient status, (D, I) patient overall survival, and (E, J) relapse free survival, between (+) and (-) groups were displayed for signature 1 without overlap genes and signature 2 without overlap genes respectively. Statistical significance was determined using chi-squared test (A, B, C, F, G, H) or log-rank test (D, E, I, J). ***P<0.01, ns; not significant.
