# Supplementary material for: Estrogen Receptor-Regulated Gene Signatures in Invasive Breast Cancer Cells and Aggressive Breast Tumors
Source: Cancers (Basel). 2022 Jun 9;14(12):2848. doi: 10.3390/cancers14122848 (PMC9221274; doi:10.3390/cancers14122848)
Supplement: Supplementary file 1 [file cancers-14-02848-s001.zip › Table S1.pdf]

Table S1: List of ER regulated genes forming signature 1 in ER and IKKβ co-activated invasive MCF-7 cells.

ENS Gene ID	Gene	FC	FDR
ENSG00000125740	FOSB	0.802471017	6.80415E-23
ENSG00000198910	L1CAM	0.508504805	1.14492E-11
ENSG00000170345	FOS	0.622883871	3.41191E-16
ENSG00000111206	FOXM1	-1.063897695	1.05714E-54
ENSG00000137804	NUSAP1	-1.366631924	4.16604E-37
ENSG00000176890	TYMS	-0.912613715	6.54869E-37
ENSG00000100297	MCM5	-0.764288987	1.00323E-29
ENSG00000136244	IL6	0.573186004	0.000419662
ENSG00000170312	CDK1	-1.47660317	2.54174E-29
ENSG00000159167	STC1	1.026099997	7.90982E-19
ENSG00000132646	PCNA	-0.818806458	7.37463E-27
ENSG00000084764	MAPRE3	0.518672303	5.18968E-05
ENSG00000131398	KCNC3	0.597844654	7.27061E-06
ENSG00000124795	DEK	-0.768031221	1.36248E-25
ENSG00000103335	PIEZO1	0.568250743	2.45165E-05
ENSG00000013810	TACC3	-1.086783039	6.29222E-23
ENSG00000138119	MYOF	0.564531856	1.52599E-16
ENSG00000076248	UNG	-0.638361168	2.76325E-21
ENSG00000152518	ZFP36L2	-0.708157204	3.3892E-21
ENSG00000184897	H1FX	-0.660757929	6.36508E-20
ENSG00000163975	MELTF	0.64106839	0.000156241
ENSG00000138182	KIF20B	-1.255871279	1.52598E-19
ENSG00000124664	SPDEF	-0.820416102	9.66345E-19
ENSG00000134970	TMED7	-1.110233166	2.67017E-16
ENSG00000137310	TCF19	-1.018443377	7.04526E-16
ENSG00000221968	FADS3	0.663169089	2.31553E-14
ENSG00000197903	HIST1H2BK	0.568797945	1.78946E-08
ENSG00000182628	SKA2	-0.755450077	3.38586E-15
ENSG00000217801	RP11-465B22.3	0.736201501	1.7387E-06
ENSG00000088305	DNMT3B	-0.804425822	4.44541E-15
ENSG00000179776	CDH5	0.847039899	7.01585E-06
ENSG00000186832	KRT16	-0.992567873	1.70154E-14
ENSG00000105419	MEIS3	0.622678334	3.11846E-09
ENSG00000043039	BARX2	-0.839883786	1.74929E-14
ENSG00000164104	HMGB2	-1.32951958	1.90755E-14
ENSG00000243279	PRAF2	0.556878611	2.54122E-11
ENSG00000092470	WDR76	-1.106628928	2.09302E-14
ENSG00000173848	NET1	-0.795268042	6.59574E-14
ENSG00000135476	ESPL1	-0.975909544	7.12462E-14
ENSG00000140525	FANCI	-0.87832029	1.22463E-13
ENSG00000178209	PLEC	0.655372468	8.68004E-11
ENSG00000135451	TROAP	-1.249115062	1.48241E-13
ENSG00000143742	SRP9	-1.126176416	5.11965E-13
ENSG00000168386	FILIP1L	-0.594970697	8.18121E-13
ENSG00000175063	UBE2C	-1.148192114	9.84542E-13

ENSG00000161800	RACGAP1	-0.868058644	1.84908E-12
ENSG00000104756	KCTD9	-0.776968149	2.81688E-12
ENSG00000126215	XRCC3	-0.539563948	4.78982E-12
ENSG00000140534	TICRR	-0.913783228	6.01351E-12
ENSG00000102034	ELF4	0.53352766	1.21698E-12
ENSG00000136367	ZFHX2	0.602819915	2.85273E-05
ENSG00000160957	RECQL4	-0.561640807	6.08508E-12
ENSG00000184985	SORCS2	0.646076152	1.07929E-14
ENSG00000268104	SLC6A14	-4.808295008	8.91747E-12
ENSG00000197045	GMFB	-0.706758618	2.03092E-11
ENSG00000127152	BCL11B	-0.781397215	2.10154E-11
ENSG00000185347	C14orf80	-0.55664418	2.22409E-11
ENSG00000117632	STMN1	-1.13165076	2.43621E-11
ENSG00000228716	DHFR	-0.652639408	4.48198E-11
ENSG00000129173	E2F8	-1.49473364	4.60472E-11
ENSG00000134363	FST	1.017327834	2.92407E-06
ENSG00000113328	CCNG1	-0.546146951	5.81984E-11
ENSG00000151239	TWF1	-0.652710364	6.91059E-11
ENSG00000170477	KRT4	-1.598067173	8.45631E-11
ENSG00000120162	MOB3B	0.98668972	9.10128E-11
ENSG00000163346	PBXIP1	0.572226253	7.43992E-05
ENSG00000177508	IRX3	-0.553473732	8.98505E-11
ENSG00000132436	FIGNL1	-0.782807628	9.21406E-11
ENSG00000091831	ESR1	-0.808827787	1.05246E-10
ENSG00000095596	CYP26A1	1.732812491	5.91454E-14
ENSG00000087258	GNAO1	1.251550622	1.05246E-10
ENSG00000116299	KIAA1324	-0.603386482	1.09047E-10
ENSG00000143401	ANP32E	-0.799000853	1.45334E-10
ENSG00000166311	SMPD1	0.592308667	2.99217E-09
ENSG00000006062	MAP3K14	0.62588981	5.93298E-10
ENSG00000205269	TMEM170B	-1.067423208	1.51507E-10
ENSG00000186298	PPP1CC	-0.683312823	1.84472E-10
ENSG00000171903	CYP4F11	0.504505926	0.00014101
ENSG00000253626	EIF5AL1	-0.584363765	1.9286E-10
ENSG00000189403	HMGB1	-0.725018854	2.14809E-10
ENSG00000126456	IRF3	0.530833344	4.374E-15
ENSG00000213639	PPP1CB	-0.630216186	2.14809E-10
ENSG00000124767	GLO1	-0.62841033	2.1656E-10
ENSG00000100744	GSKIP	-0.638600991	2.17705E-10
ENSG00000138759	FRAS1	1.356706662	0.000240049
ENSG00000196584	XRCC2	-1.13654206	2.40452E-10
ENSG00000122966	CIT	-1.024680792	3.44846E-10
ENSG00000122367	LDB3	0.703881315	0.000361636
ENSG00000142632	ARHGEF19	-0.816248837	3.56107E-10
ENSG00000183856	IQGAP3	-0.988300027	3.8541E-10
ENSG00000213390	ARHGAP19	-1.027171975	4.32384E-10
ENSG00000157500	APPL1	-0.71466548	4.53191E-10

ENSG00000169139	UBE2V2	-0.606110354	5.76679E-10
ENSG00000233198	RNF224	1.088852302	2.90989E-07
ENSG00000026652	AGPAT4	1.671212209	0.000780221
ENSG00000012171	SEMA3B	-0.809387995	6.0537E-10
ENSG00000166508	MCM7	-0.710249048	7.21192E-10
ENSG00000081154	PCNP	-0.51354678	8.98599E-10
ENSG00000143341	HMCN1	-1.100896866	1.0736E-09
ENSG00000106462	EZH2	-0.560184552	1.32607E-09
ENSG00000130304	SLC27A1	0.525166939	0.000762019
ENSG00000113810	SMC4	-1.144066799	1.49421E-09
ENSG00000119403	PHF19	-0.827736027	1.52564E-09
ENSG00000134775	FHOD3	-1.064080863	1.54479E-09
ENSG00000213347	MXD3	-0.892276926	1.94114E-09
ENSG00000112742	TTK	-1.195953963	2.32437E-09
ENSG00000147872	PLIN2	-0.898952835	3.38641E-09
ENSG00000091651	ORC6	-0.84436539	4.34528E-09
ENSG00000123374	CDK2	-0.604967387	6.21924E-09
ENSG00000076770	MBNL3	-0.845274876	6.26257E-09
ENSG00000109861	CTSC	-0.670290715	6.54286E-09
ENSG00000143578	CREB3L4	-0.82612705	6.87812E-09
ENSG00000136122	BORA	-1.200301776	6.99203E-09
ENSG00000168393	DTYMK	-0.669929265	7.28913E-09
ENSG00000165997	ARL5B	-0.64525606	8.04775E-09
ENSG00000103145	HCFC1R1	-0.564529736	1.03159E-08
ENSG00000011426	ANLN	-0.827961819	1.76309E-08
ENSG00000178295	GEN1	-0.69464974	1.82602E-08
ENSG00000138642	HERC6	0.775143782	0.000449565
ENSG00000104442	ARMC1	-0.69681742	2.41078E-08
ENSG00000171862	PTEN	-0.55861727	2.47769E-08
ENSG00000251615	RP11-774O3.3	0.640193768	0.000253695
ENSG00000105011	ASF1B	-0.761137666	2.85565E-08
ENSG00000109762	SNX25	0.586741612	2.41974E-05
ENSG00000159873	CCDC117	-0.52247901	3.03507E-08
ENSG00000157368	IL34	0.855054544	0.000496535
ENSG00000110042	DTX4	0.903977888	0.000496535
ENSG00000114346	ECT2	-0.751286846	3.33247E-08
ENSG00000136928	GABBR2	0.800375253	5.46925E-07
ENSG00000101911	PRPS2	-0.505209911	3.34873E-08
ENSG00000140451	PIF1	-1.444956865	4.28325E-08
ENSG00000157851	DPYSL5	-0.70979699	1.04222E-07
ENSG00000163513	TGFBR2	0.600172136	0.000820996
ENSG00000134049	IER3IP1	-1.151267344	1.31651E-07
ENSG00000108821	COL1A1	0.820984323	6.34395E-06
ENSG00000109685	NSD2	-0.618658952	1.50136E-07
ENSG00000196126	HLA-DRB1	3.452252334	2.8319E-05
ENSG00000076554	TPD52	-0.538122764	1.61928E-07
ENSG00000100410	PHF5A	-0.594192462	2.15436E-07

ENSG00000163703	CRELD1	0.521722211	3.65957E-05
ENSG00000010292	NCAPD2	-0.771055302	2.59619E-07
ENSG00000126458	RRAS	-0.56548532	2.98868E-07
ENSG00000198892	SHISA4	1.196525748	1.481E-21
ENSG00000159055	MIS18A	-0.680674854	4.57787E-07
ENSG00000136167	LCP1	0.700639069	7.16214E-06
ENSG00000167670	CHAF1A	-0.809932509	6.83121E-07
ENSG00000122643	NT5C3A	-0.608855561	8.56892E-07
ENSG00000104147	OIP5	-1.262364578	1.00819E-06
ENSG00000024422	EHD2	-0.590528778	1.32976E-06
ENSG00000086300	SNX10	-1.016162202	1.40875E-06
ENSG00000120992	LYPLA1	-0.515657576	1.54671E-06
ENSG00000198569	SLC34A3	1.823364429	9.46454E-05
ENSG00000137770	CTDSPL2	-0.510416062	1.64164E-06
ENSG00000073111	MCM2	-0.658316894	1.71461E-06
ENSG00000164087	POC1A	-0.815156585	1.78004E-06
ENSG00000000460	C1orf112	-0.737785333	1.79567E-06
ENSG00000123473	STIL	-0.740384179	1.93654E-06
ENSG00000162063	CCNF	-0.886812302	2.11227E-06
ENSG00000189266	PNRC2	-0.50240892	2.14191E-06
ENSG00000219200	RNASEK	0.645614661	2.88204E-06
ENSG00000165272	AQP3	1.169988697	8.25633E-14
ENSG00000196220	SRGAP3	-0.726734589	2.48968E-06
ENSG00000091622	PITPNM3	-0.52113112	2.70485E-06
ENSG00000156398	SFXN2	-0.744739273	3.09518E-06
ENSG00000111247	RAD51AP1	-1.727107081	3.29518E-06
ENSG00000254221	PCDHGB1	0.825656927	5.9223E-08
ENSG00000221829	FANCG	-0.647581463	3.51479E-06
ENSG00000151366	NDUFC2	-0.894202274	3.72034E-06
ENSG00000143815	LBR	-0.533836875	3.73029E-06
ENSG00000058085	LAMC2	0.660093757	0.00067881
ENSG00000186638	KIF24	-1.174134538	3.88186E-06
ENSG00000136527	TRA2B	-0.610827954	3.88186E-06
ENSG00000130827	PLXNA3	0.533794639	0.000477225
ENSG00000161888	SPC24	-1.127806827	4.43636E-06
ENSG00000164754	RAD21	-0.64618119	4.47079E-06
ENSG00000108602	ALDH3A1	0.86415073	1.6776E-07
ENSG00000006625	GGCT	-0.573553699	5.0323E-06
ENSG00000170270	GON7	-0.677117088	5.29316E-06
ENSG00000176208	ATAD5	-1.268817765	6.19053E-06
ENSG00000170779	CDCA4	-0.563875187	6.97792E-06
ENSG00000168496	FEN1	-0.589053303	7.11853E-06
ENSG00000197008	ZNF138	-0.501479746	7.17743E-06
ENSG00000102265	TIMP1	0.610337465	4.62968E-18
ENSG00000171320	ESCO2	-0.915304261	1.00599E-05
ENSG00000204539	CDSN	1.066607239	6.51725E-07
ENSG00000112118	MCM3	-0.570881764	1.07155E-05

ENSG00000175785	PRIMA1	-1.41419925	1.25995E-05
ENSG00000174130	TLR6	-0.820054703	1.27312E-05
ENSG00000174327	SLC16A13	0.51487829	8.53912E-08
ENSG00000180998	GPR137C	-0.885129398	1.44232E-05
ENSG00000177034	MTX3	-0.508892762	1.89526E-05
ENSG00000124574	ABCC10	0.519712542	8.73978E-06
ENSG00000065665	SEC61A2	0.57877788	2.26414E-05
ENSG00000181192	DHTKD1	-0.798432805	1.93262E-05
ENSG00000148773	MKI67	-1.215773987	1.94096E-05
ENSG00000124508	BTN2A2	0.550018897	1.56955E-06
ENSG00000122952	ZWINT	-0.814212223	1.94096E-05
ENSG00000197948	FCHSD1	0.583244857	0.000463255
ENSG00000176974	SHMT1	-0.608055324	2.12015E-05
ENSG00000186815	TPCN1	0.682396505	2.10367E-07
ENSG00000146143	PRIM2	-0.806626609	2.23089E-05
ENSG00000130600	H19	-2.164361884	2.26414E-05
ENSG00000261068	RP11-7K24.3	1.222386681	5.18307E-06
ENSG00000072864	NDE1	-0.515245648	2.43411E-05
ENSG00000064102	INTS13	-0.590054978	2.86307E-05
ENSG00000092036	HAUS4	-0.801566541	3.00018E-05
ENSG00000117748	RPA2	-0.568503515	3.50879E-05
ENSG00000149636	DSN1	-0.636494095	3.76809E-05
ENSG00000178074	C2orf69	-0.580811301	4.01523E-05
ENSG00000187951	ARHGAP11B	-1.570193643	4.14848E-05
ENSG00000188763	FZD9	0.855543212	1.43853E-13
ENSG00000184445	KNTC1	-0.717441771	4.15774E-05
ENSG00000134758	RNF138	-0.614073842	4.64611E-05
ENSG00000241399	CD302	-0.684852778	4.81485E-05
ENSG00000123219	CENPK	-1.17687323	5.17016E-05
ENSG00000271383	NBPF19	0.571033972	0.000172351
ENSG00000111581	NUP107	-0.645438771	5.25852E-05
ENSG00000173599	PC	-0.507981629	5.2771E-05
ENSG00000177917	ARL6IP6	-0.529626314	6.15208E-05
ENSG00000178695	KCTD12	1.499852827	2.85565E-08
ENSG00000189057	FAM111B	-1.043400294	6.30025E-05
ENSG00000112964	GHR	-0.540275786	6.71453E-05
ENSG00000171522	PTGER4	1.893244147	2.33406E-09
ENSG00000163743	RCHY1	-0.598250285	6.86346E-05
ENSG00000146038	DCDC2	-0.678908262	6.8646E-05
ENSG00000176912	TYMSOS	-1.378177968	6.89183E-05
ENSG00000109881	CCDC34	-0.721886533	7.79607E-05
ENSG00000205078	SYCE1L	1.191566021	0.000513052
ENSG00000129317	PUS7L	-0.612071764	8.22099E-05
ENSG00000125885	MCM8	-0.582828088	8.45128E-05
ENSG00000135245	HILPDA	-0.715441221	8.60994E-05
ENSG00000116260	QSOX1	0.656515437	1.08639E-39
ENSG00000189308	LIN54	-0.675912696	8.62636E-05

ENSG00000203668	CHML	-0.517540112	9.03372E-05
ENSG00000178966	RMI1	-1.027933159	9.93807E-05
ENSG00000100162	CENPM	-0.774431467	0.000107772
ENSG00000153130	SCOC	-0.528955388	0.000107772
ENSG00000132763	MMACHC	-0.558600671	0.000112913
ENSG00000160298	C21orf58	-0.778633783	0.000138878
ENSG00000188610	FAM72B	-1.851372145	0.000149648
ENSG00000075218	GTSE1	-1.011027249	0.000177784
ENSG00000147471	PROSC	-0.59339378	0.000181543
ENSG00000174567	GOLT1A	0.534462518	5.28358E-06
ENSG00000159216	RUNX1	0.61411024	9.12589E-11
ENSG00000144802	NFKBIZ	0.895712178	2.62108E-09
ENSG00000152455	SUV39H2	-0.577514321	0.000181973
ENSG00000166803	PCLAF	-1.230346123	0.000183619
ENSG00000168286	THAP11	-0.913283366	0.000194274
ENSG00000197472	ZNF695	-1.343755952	0.000196975
ENSG00000089050	RBBP9	-0.502740962	0.000217917
ENSG00000156374	PCGF6	-0.76370151	0.000233573
ENSG00000138767	CNOT6L	-0.548407831	0.000237097
ENSG00000182985	CADM1	0.935771995	7.86921E-10
ENSG00000133393	FOPNL	-0.576624827	0.000240049
ENSG00000152689	RASGRP3	1.442763724	0.000405294
ENSG00000180385	EMC3-AS1	-0.55375496	0.000249185
ENSG00000161692	DBF4B	-0.500567621	0.000250594
ENSG00000178177	LCORL	-0.845004682	0.000270348
ENSG00000184588	PDE4B	-0.56164204	0.000280989
ENSG00000124496	TRERF1	-0.563388534	0.000305315
ENSG00000103089	FA2H	-0.655205965	0.000310215
ENSG00000170899	GSTA4	-1.979442432	0.000337176
ENSG00000121579	NAA50	-0.580759483	0.000337361
ENSG00000135899	SP110	0.804377724	8.6474E-06
ENSG00000196372	ASB13	-0.795696426	0.000372392
ENSG00000138835	RGS3	-0.520500672	0.000409304
ENSG00000204370	SDHD	-0.833121591	0.000466215
ENSG00000117226	GBP3	2.088261943	0.000811353
ENSG00000121897	LIAS	-0.734505209	0.000495931
ENSG00000166002	SMCO4	-1.423264812	0.000502281
ENSG00000118564	FBXL5	-0.548249863	0.000510147
ENSG00000274276	CBSL	1.325027062	0.000337176
ENSG00000080986	NDC80	-1.624290707	0.00052935
ENSG00000151632	AKR1C2	-1.224241863	0.000562077
ENSG00000196678	ERI2	-0.89047743	0.000577152
ENSG00000137198	GMPR	1.015917272	0.000337361
ENSG00000175087	PDIK1L	-0.834926727	0.00058109
ENSG00000113594	LIFR	-1.213829278	0.000587337
ENSG00000138658	ZGRF1	-0.675022139	0.000595295
ENSG00000051341	POLQ	-0.969847145	0.000604425

ENSG00000164403	SHROOM1	0.712323591	0.000181973
ENSG00000158402	CDC25C	-1.455145179	0.000604425
ENSG00000204434	POTEKP	0.974137054	0.000992524
ENSG00000213186	TRIM59	-0.821742373	0.000605396
ENSG00000127129	EDN2	1.149481801	0.000154052
ENSG00000104081	BMF	0.779638915	1.6628E-07
ENSG00000213399	AC022210.2	-0.532603919	0.000625538
ENSG00000090447	TFAP4	-0.50327752	0.000637968
ENSG00000175305	CCNE2	-0.885465315	0.000678667
ENSG00000186918	ZNF395	-0.55062808	0.000687748
ENSG00000108375	RNF43	-0.515219083	0.000704548
ENSG00000165714	BORCS5	-0.573047123	0.000710012
ENSG00000147601	TERF1	-0.507621042	0.000752295
ENSG00000138587	MNS1	-1.31830575	0.000762019
ENSG00000119640	ACYP1	-0.774332301	0.000791359
ENSG00000150054	MPP7	-0.520466715	0.000807863
ENSG00000131094	C1QL1	-1.182555484	0.000866263
ENSG00000085511	MAP3K4	-0.524434341	0.000873501
ENSG00000138356	AOX1	-0.747153625	0.000901441
ENSG00000092853	CLSPN	-0.976041112	0.000913364
ENSG00000100479	POLE2	-0.898505508	0.000981483
ENSG00000163040	CCDC74A	-0.775951498	0.000988671
ENSG00000169710	FASN	0.531882913	3.2733E-08
ENSG00000145860	RNF145	0.591127082	0.000960497
ENSG00000129566	TEP1	0.600482349	2.85041E-05
ENSG00000135919	SERPINE2	0.609585789	1.81369E-05
ENSG00000127507	ADGRE2	0.620163576	5.35499E-05
ENSG00000107201	DDX58	0.631278732	7.84429E-06
ENSG00000115267	IFIH1	0.67755727	2.51293E-05
ENSG00000213462	ERV3-1	0.708718411	1.37095E-05
ENSG00000114841	DNAH1	0.891326006	6.4537E-06
ENSG00000185245	GP1BA	1.758758188	0.000348174
ENSG00000167914	GSDMA	1.764884508	4.6035E-05
ENSG00000155657	TTN	4.178594203	2.94309E-05
