# Supplementary material for: Estrogen Receptor-Regulated Gene Signatures in Invasive Breast Cancer Cells and Aggressive Breast Tumors
Source: Cancers (Basel). 2022 Jun 9;14(12):2848. doi: 10.3390/cancers14122848 (PMC9221274; doi:10.3390/cancers14122848)
Supplement: Supplementary file 1 [file cancers-14-02848-s001.zip › Table S10.pdf]

Table S10. Functional Enrichment Analysis for stem cell-associated gene signatures in invasive cell populations.

Signatures	Clusters									
	0		1		2		3		4	
	AUC	P-val	AUC	P-val	AUC	P-val	AUC	P-val	AUC	P-val
LIM_MAMMARY_STEM_CELL_UP	0.57607	0.00247	0.56995	0.00969	0.56177	0.06947	0.0465	1.34E-22	0.33782	0.00207
PECE_MAMMARY_STEM_CELL_UP	0.57285	0.00386	0.53917	0.14717	0.19223	9.11E-20	0.84997	5.36E-14	0.33179	0.00136
WONG_EMBRYONIC_STEM_CELL_CORE	0.53325	0.18579	0.3997	0.00019	0.52501	0.45294	0.26112	2.29E-07	0.98545	2.60E-20
ENGELMANN_CANCER_PROGENITORS_UP	0.53482	0.17454	0.55328	0.04803	0.58382	0.01184	0.08942	5.79E-19	0.47114	0.60648
LIM_MAMMARY_LUMINAL_PROGENITOR_UP	0.5447	0.07882	0.61872	1.05E-05	0.45099	0.14853	0.22467	3.01E-09	0.32702	0.00103
ZHANG_BREAST_CANCER_PROGENITORS_UP	0.51722	0.49801	0.47718	0.39261	0.58119	0.0162	0.05848	1.71E-21	0.88371	2.56E-13
BENPORATH_ES_1	0.48637	0.6049	0.44101	0.02865	0.66379	1.29E-06	0.04907	1.72E-22	0.96685	6.90E-19
BENPORATH_SOX2_TARGETS	0.49288	0.7803	0.55361	0.04528	0.59713	0.0041	0.04144	4.55E-23	0.68077	0.00063
BENPORATH_NANOG_TARGETS	0.49645	0.8685	0.58874	0.00096	0.55326	0.11417	0.03731	2.61E-23	0.64307	0.00656
