# Supplementary material for: Estrogen Receptor-Regulated Gene Signatures in Invasive Breast Cancer Cells and Aggressive Breast Tumors
Source: Cancers (Basel). 2022 Jun 9;14(12):2848. doi: 10.3390/cancers14122848 (PMC9221274; doi:10.3390/cancers14122848)
Supplement: Supplementary file 1 [file cancers-14-02848-s001.zip › Table S2.pdf]

Table S2: GSEA analysis of signature 1 genes showing enriched Hallmark and gene ontology gene sets.

ICI Regulation Group	Term	SIZE	NES	NOM p-val
Down Regulated Genes	HALLMARK_G2M_CHECKPOINT	29	-2.1699433	0.001579779
Down Regulated Genes	HALLMARK_E2F_TARGETS	35	-2.1103632	0
Down Regulated Genes	HALLMARK_MITOTIC_SPINDLE	14	-2.0691934	0.004885993
Down Regulated Genes	HALLMARK_SPERMATOGENESIS	3	-1.461269	0.06410257
Down Regulated Genes	HALLMARK_KRAS_SIGNALING_DN	4	-1.4591109	0.08303887
Down Regulated Genes	HALLMARK_ADIPOGENESIS	4	-1.3965453	0.10460993
Down Regulated Genes	HALLMARK_MYC_TARGETS_V1	10	-1.3158977	0.18543047
Down Regulated Genes	HALLMARK_OXIDATIVE_PHOSPHORYLATION	2	-1.2751338	0.13384321
Down Regulated Genes	HALLMARK_DNA_REPAIR	7	-1.173275	0.2852174
Down Regulated Genes	HALLMARK_PANCREAS_BETA_CELLS	1	-1.1539434	0.26070037
Down Regulated Genes	HALLMARK_PI3K_AKT_MTOR_SIGNALING	3	-1.0637633	0.37898687
Down Regulated Genes	HALLMARK_GLYCOLYSIS	5	-1.035676	0.40860215
Down Regulated Genes	HALLMARK_ANDROGEN_RESPONSE	3	-1.0308858	0.41396934
Down Regulated Genes	HALLMARK_MYC_TARGETS_V2	2	-0.8971308	0.6230469
Down Regulated Genes	HALLMARK_PROTEIN_SECRETION	3	-0.7418733	0.8357934
Down Regulated Genes	HALLMARK_APOPTOSIS	5	-0.67667055	0.8441331
Down Regulated Genes	HALLMARK_CHOLESTEROL_HOMEOSTASIS	2	-0.5331622	0.9787645
Down Regulated Genes	HALLMARK_ESTROGEN_RESPONSE_LATE	8	-0.4875066	0.9836066
Upregulated Genes	HALLMARK_EPITHELIAL_MESENCHYMAL_TRANSITION	8	1.9644825	0.007228916
Upregulated Genes	HALLMARK_INTERFERON_GAMMA_RESPONSE	7	1.9509985	0.013667426
Upregulated Genes	HALLMARK_INTERFERON_ALPHA_RESPONSE	4	1.9396011	0.002309469
Upregulated Genes	HALLMARK_TNFA_SIGNALING_VIA_NFKB	7	1.9056153	0.002398082
Upregulated Genes	HALLMARK_INFLAMMATORY_RESPONSE	5	1.6371281	0.03628118
Upregulated Genes	HALLMARK_HEME_METABOLISM	3	1.6049992	0.029850746
Upregulated Genes	HALLMARK_MYOGENESIS	7	1.5226359	0.07323232
Upregulated Genes	HALLMARK_ANGIOGENESIS	2	1.3919984	0.074235804
Upregulated Genes	HALLMARK_COAGULATION	2	1.3816751	0.09606987
Upregulated Genes	HALLMARK_UV_RESPONSE_UP	6	1.369305	0.14320388
Upregulated Genes	HALLMARK_UV_RESPONSE_DN	4	1.3195071	0.16981132
Upregulated Genes	HALLMARK_APICAL_SURFACE	1	1.3105288	0.03164557
Upregulated Genes	HALLMARK_APICAL_JUNCTION	4	1.282181	0.2106383
Upregulated Genes	HALLMARK_HYPOXIA	6	1.239377	0.20327103
Upregulated Genes	HALLMARK_NOTCH_SIGNALING	1	1.2282631	0.15843621
Upregulated Genes	HALLMARK_PEROXISOME	1	1.2153157	0.16465864
Upregulated Genes	HALLMARK_COMPLEMENT	5	1.0903863	0.34005037
Upregulated Genes	HALLMARK_IL2_STAT5_SIGNALING	4	0.9963628	0.46347606
Upregulated Genes	HALLMARK_IL6_JAK_STAT3_SIGNALING	1	0.98065597	0.5289079
Upregulated Genes	HALLMARK_XENOBIOTIC_METABOLISM	6	0.9427903	0.48364487
Upregulated Genes	HALLMARK_HEDGEHOG_SIGNALING	1	0.9405719	0.5877551
Upregulated Genes	HALLMARK_ALLOGRAFT_REJECTION	5	0.9022572	0.5549872
Upregulated Genes	HALLMARK_ESTROGEN_RESPONSE_EARLY	7	0.836061	0.63990265
Upregulated Genes	HALLMARK_MTORC1_SIGNALING	7	0.7598293	0.7330317
Upregulated Genes	HALLMARK_FATTY_ACID_METABOLISM	3	0.7368341	0.7880086
Upregulated Genes	HALLMARK_P53_PATHWAY	4	0.6226139	0.90114945
Upregulated Genes	HALLMARK_KRAS_SIGNALING_UP	2	0.55042416	0.9736842
Down Regulated Genes	GO_CELL_CYCLE	89	-2.3101294	0
Down Regulated Genes	GO_CELL_DIVISION	39	-2.2953584	0
Down Regulated Genes	GO_REGULATION_OF_CELL_CYCLE	54	-2.2494712	0
Down Regulated Genes	GO_MITOTIC_CELL_CYCLE	66	-2.217163	0
Down Regulated Genes	GO_REGULATION_OF_NUCLEAR_DIVISION	15	-2.2009432	0
Down Regulated Genes	GO_DNA_REPAIR	37	-2.176404	0
Down Regulated Genes	GO_REGULATION_OF_CELL_CYCLE_PROCESS	45	-2.1617072	0.001597444
Down Regulated Genes	GO_CELL_CYCLE_PROCESS	75	-2.1449103	0.002808989
Down Regulated Genes	GO_REGULATION_OF_MITOTIC_CELL_CYCLE	36	-2.1126451	0.001618123
Down Regulated Genes	GO_CELLULAR_RESPONSE_TO_DNA_DAMAGE_STIMULUS	45	-2.0964923	0.001589825
Down Regulated Genes	GO_DNA_METABOLIC_PROCESS	53	-2.0834868	0.001529052
Down Regulated Genes	GO_POSITIVE_REGULATION_OF_MITOTIC_CELL_CYCLE	10	-2.0726585	0.001779359
Down Regulated Genes	GO_CYTOKINESIS	9	-2.0372863	0.001712329
Down Regulated Genes	GO_POSITIVE_REGULATION_OF_CELL_CYCLE_PROCESS	23	-2.0243025	0.001618123
Down Regulated Genes	GO_MICROTUBULE_BASED_PROCESS	29	-2.0028143	0.003210273
Down Regulated Genes	GO_DNA_REPLICATION	30	-1.9470644	0.003246753
Down Regulated Genes	GO_MICROTUBULE_CYTOSKELETON_ORGANIZATION_INVOLVED_IN_MITOSIS	12	-1.9398468	0.005059022
Down Regulated Genes	GO_CELL_CYCLE_PHASE_TRANSITION	47	-1.9296776	0.00456621
Down Regulated Genes	GO_MICROTUBULE_CYTOSKELETON_ORGANIZATION	26	-1.919999	0.003273323
Down Regulated Genes	GO_MITOTIC_CYTOKINESIS	8	-1.9177812	0.001745201
Down Regulated Genes	GO_CYTOSKELETON_DEPENDENT_CYTOKINESIS	8	-1.912187	0
Down Regulated Genes	GO_POSITIVE_REGULATION_OF_CELL_CYCLE	26	-1.8812535	0.004709576
Down Regulated Genes	GO_DNA_INTEGRITY_CHECKPOINT	11	-1.8746063	0.008264462

Down Regulated Genes	GO_CELL_CYCLE_CHECKPOINT	17	-1.8720583	0.009884679
Down Regulated Genes	GO_SIGNAL_TRANSDUCTION_BY_P53_CLASS_MEDIATOR	12	-1.8712102	0.006861064
Down Regulated Genes	GO_CHROMATIN_ORGANIZATION	24	-1.8680828	0.00312989
Down Regulated Genes	GO_REGULATION_OF_SMALL_GTPASE_MEDIATED_SIGNAL_TRANSDUCTION	9	-1.8587835	0
Down Regulated Genes	GO_SPINDLE_ORGANIZATION	12	-1.8489735	0.005208334
Down Regulated Genes	GO_CELL_CYCLE_G2_M_PHASE_TRANSITION	14	-1.84758	0.005025126
Down Regulated Genes	GO_PROTEIN_LOCALIZATION_TO_CHROMOSOME_CENTROMERIC_REGION	3	-1.8454702	0
Down Regulated Genes	GO_POSITIVE_REGULATION_OF_CELL_CYCLE_PHASE_TRANSITION	7	-1.837051	0.010771993
Down Regulated Genes	GO_MITOTIC_SPINDLE_ORGANIZATION	9	-1.828083	0.008710802
Down Regulated Genes	GO_MITOTIC_CELL_CYCLE_CHECKPOINT	16	-1.8173108	0.01584786
Down Regulated Genes	GO_PROTEIN_LOCALIZATION_TO_KINETOCHORE	3	-1.8155123	0
Down Regulated Genes	GO_RESPONSE_TO_IONIZING_RADIATION	7	-1.7739451	0.012658228
Down Regulated Genes	GO_KINETOCHORE_ORGANIZATION	3	-1.7650597	0.003616637
Down Regulated Genes	GO_SIGNAL_TRANSDUCTION_IN_RESPONSE_TO_DNA_DAMAGE	10	-1.7450866	0.013605442
Down Regulated Genes	GO_MITOTIC_DNA_INTEGRITY_CHECKPOINT	10	-1.7402742	0.018932875
Down Regulated Genes	GO_NUCLEOSOME_ORGANIZATION	9	-1.7126435	0.024958402
Down Regulated Genes	GO_CHROMOSOME_SEPARATION	10	-1.7111162	0.025125628
Down Regulated Genes	GO_REGULATION_OF_CHROMOSOME_SEPARATION	8	-1.7044691	0.016891891
Down Regulated Genes	GO_METAPHASE_ANAPHASE_TRANSITION_OF_CELL_CYCLE	8	-1.6992633	0.013559322
Down Regulated Genes	GO_ORGANIC_ACID_TRANSMEMBRANE_TRANSPORT	3	-1.6934074	0.007662835
Down Regulated Genes	GO_CELL_CYCLE_ARREST	11	-1.6921991	0.030821918
Down Regulated Genes	GO_INTRACELLULAR_PROTEIN_TRANSPORT	16	-1.6914426	0.011363637
Down Regulated Genes	GO_ORGANIC_ACID_TRANSPORT	5	-1.6892133	0.017035775
Down Regulated Genes	GO_ANION_TRANSMEMBRANE_TRANSPORT	3	-1.6876768	0.005565863
Down Regulated Genes	GO_CHROMOSOME_ORGANIZATION	58	-1.6855068	0.013975156
Down Regulated Genes	GO_NEGATIVE_REGULATION_OF_CELL_CYCLE	30	-1.6818432	0.020967742
Down Regulated Genes	GO_REGULATION_OF_CELL_DIVISION	6	-1.6786938	0.019469026
Down Regulated Genes	GO_ORGANIC_ANION_TRANSPORT	5	-1.6745828	0.017211704
Down Regulated Genes	GO_RESPONSE_TO_TOXIC_SUBSTANCE	6	-1.6722492	0.020484172
Down Regulated Genes	GO_REGULATION_OF_DNA_REPLICATION	9	-1.6639681	0.032534245
Down Regulated Genes	GO_REGULATION_OF_CHROMOSOME_SEGREGATION	12	-1.6614746	0.028428094
Down Regulated Genes	GO_POSITIVE_REGULATION_OF_MITOTIC_NUCLEAR_DIVISION	3	-1.6545218	0.011173184
Down Regulated Genes	GO_DOUBLE_STRAND_BREAK_REPAIR	22	-1.6484916	0.013975156
Down Regulated Genes	GO_POSITIVE_REGULATION_OF_DNA_REPLICATION	6	-1.6463176	0.02640845
Down Regulated Genes	GO_POSITIVE_REGULATION_OF_CELLULAR_PROTEIN_LOCALIZATION	6	-1.6425235	0.028318584
Down Regulated Genes	GO_NUCLEOSOME_ASSEMBLY	8	-1.64083	0.041970804
Down Regulated Genes	GO_MEIOTIC_CHROMOSOME_SEPARATION	3	-1.6394212	0.011214953
Down Regulated Genes	GO_ORGANELLE_FISSION	29	-1.6357175	0.02134647
Down Regulated Genes	GO_POSITIVE_REGULATION_OF_NUCLEAR_DIVISION	3	-1.6337765	0.019332161
Down Regulated Genes	GO_DNA_DAMAGE_RESPONSE_SIGNAL_TRANSDUCTION_BY_P53_CLASS_MEDIATOR	9	-1.6325909	0.04152249
Down Regulated Genes	GO_MITOTIC_G2_DNA_DAMAGE_CHECKPOINT	3	-1.632384	0.015065913
Down Regulated Genes	GO_NEGATIVE_REGULATION_OF_DNA_METABOLIC_PROCESS	3	-1.6284677	0.016393442
Down Regulated Genes	GO_NEGATIVE_REGULATION_OF_RESPONSE_TO_DNA_DAMAGE_STIMULUS	2	-1.6248006	0.005703422
Down Regulated Genes	GO_REGULATION_OF_DNA_RECOMBINATION	6	-1.6243291	0.024822695
Down Regulated Genes	GO_IMPORT_ACROSS_PLASMA_MEMBRANE	2	-1.6223638	0.014925373
Down Regulated Genes	GO_PROTEIN_DNA_COMPLEX_SUBUNIT_ORGANIZATION	16	-1.6208527	0.038590603
Down Regulated Genes	GO_MITOTIC_G2_M_TRANSITION_CHECKPOINT	3	-1.6187075	0.015296367
Down Regulated Genes	GO_G2_DNA_DAMAGE_CHECKPOINT	3	-1.6100446	0.016453382
Down Regulated Genes	GO_DNA_RECOMBINATION	25	-1.6070415	0.040133778
Down Regulated Genes	GO_CELL_CYCLE_G1_S_PHASE_TRANSITION	24	-1.6044513	0.035881434
Down Regulated Genes	GO_REGULATION_OF_CELL_CYCLE_G2_M_PHASE_TRANSITION	11	-1.5984414	0.038016528
Down Regulated Genes	GO_IMPORT_INTO_CELL	2	-1.5859702	0.003759399
Down Regulated Genes	GO_REGULATION_OF_CYTOKINESIS	5	-1.5848958	0.03723404
Down Regulated Genes	GO_ESTABLISHMENT_OF_MITOTIC_SPINDLE_LOCALIZATION	4	-1.5835859	0.030852994
Down Regulated Genes	GO_PEPTIDYL_THREONINE_MODIFICATION	4	-1.5789387	0.045126352
Down Regulated Genes	GO_MITOTIC_NUCLEAR_DIVISION	24	-1.5741106	0.046698872
Down Regulated Genes	GO_PROTEIN_LOCALIZATION_TO_ORGANELLE	24	-1.5721391	0.051282052
Down Regulated Genes	GO_SPINDLE_LOCALIZATION	4	-1.5715578	0.053475935
Down Regulated Genes	GO_REGULATION_OF_CELL_CYCLE_PHASE_TRANSITION	29	-1.5695313	0.05629139
Down Regulated Genes	GO_REGULATION_OF_RESPONSE_TO_DNA_DAMAGE_STIMULUS	12	-1.5650039	0.0519263
Down Regulated Genes	GO_CELLULAR_RESPIRATION	4	-1.5616907	0.043782838
Down Regulated Genes	GO_NEGATIVE_REGULATION_OF_CELL_CYCLE_PHASE_TRANSITION	19	-1.5591422	0.045608107
Down Regulated Genes	GO_GENERATION_OF_PRECURSOR_METABOLITES_AND_ENERGY	8	-1.5574751	0.037996545
Down Regulated Genes	GO_REGULATION_OF_SISTER_CHROMATID_SEGREGATION	9	-1.556413	0.060556464
Down Regulated Genes	GO_POSITIVE_REGULATION_OF_CELL_CYCLE_G2_M_PHASE_TRANSITION	4	-1.5548344	0.043402776
Down Regulated Genes	GO_SIGNAL_TRANSDUCTION_INVOLVED_IN_CELL_CYCLE_CHECKPOINT	7	-1.5528722	0.052721087
Down Regulated Genes	GO_AMINO_ACID_TRANSPORT	2	-1.5488534	0.019455252
Down Regulated Genes	GO_REGULATION_OF_ORGANELLE_ORGANIZATION	44	-1.5387315	0.037383176
Down Regulated Genes	GO_TRANSMEMBRANE_TRANSPORT	15	-1.5287799	0.060755335
Down Regulated Genes	GO_AMINO_ACID_TRANSMEMBRANE_TRANSPORT	2	-1.5282811	0.016885553

Down Regulated Genes	GO_REGULATION_OF_CHROMOSOME_ORGANIZATION	16	-1.5255741	0.05804312
Down Regulated Genes	GO_INTRACELLULAR_TRANSPORT	23	-1.5248957	0.07431341
Down Regulated Genes	GO_POSITIVE_REGULATION_OF_CELL_CYCLE_ARREST	7	-1.5234509	0.06097561
Down Regulated Genes	GO_RESPIRATORY_ELECTRON_TRANSPORT_CHAIN	3	-1.5225847	0.04323308
Down Regulated Genes	GO_MICROTUBULE_DEPOLYMERIZATION	2	-1.5121238	0.030303031
Down Regulated Genes	GO_MICROTUBULE_ORGANIZING_CENTER_ORGANIZATION	12	-1.5105802	0.07177033
Down Regulated Genes	GO_CHROMATIN_REMODELING	9	-1.5103078	0.06238859
Down Regulated Genes	GO_COTRANSLATIONAL_PROTEIN_TARGETING_TO_MEMBRANE	2	-1.5102255	0.036968578
Down Regulated Genes	GO_NUCLEUS_ORGANIZATION	3	-1.5083345	0.03642987
Down Regulated Genes	GO_ATP_SYNTHESIS_COUPLED_ELECTRON_TRANSPORT	3	-1.5034395	0.05745063
Down Regulated Genes	GO_ESTABLISHMENT_OF_PROTEIN_LOCALIZATION_TO_ENDOPLASMIC_RETICULUM	2	-1.5022047	0.035781544
Down Regulated Genes	GO_PROTEIN_TARGETING_TO_MEMBRANE	2	-1.5020763	0.023550725
Down Regulated Genes	GO_GOLGI_ORGANIZATION	2	-1.5001818	0.034615386
Down Regulated Genes	GO_PROTEIN_TARGETING	2	-1.4979796	0.02851711
Down Regulated Genes	GO_PROTEIN_LOCALIZATION_TO_ENDOPLASMIC_RETICULUM	2	-1.4976432	0.021543985
Down Regulated Genes	GO_OXIDATIVE_PHOSPHORYLATION	3	-1.4969169	0.04964539
Down Regulated Genes	GO_MEIOTIC_CELL_CYCLE	15	-1.495482	0.09415584
Down Regulated Genes	GO_NEGATIVE_REGULATION_OF_MITOTIC_CELL_CYCLE	21	-1.493884	0.07790143
Down Regulated Genes	GO_FIBROBLAST_APOPTOTIC_PROCESS	2	-1.4928728	0.027173912
Down Regulated Genes	GO_NEGATIVE_REGULATION_OF_CELL_CYCLE_PROCESS	25	-1.4913522	0.07906977
Down Regulated Genes	GO_DNA_CONFORMATION_CHANGE	21	-1.4888328	0.092257
Down Regulated Genes	GO_REGULATION_OF_SPINDLE_ORGANIZATION	2	-1.4875897	0.02877698
Down Regulated Genes	GO_REGULATION_OF_DOUBLE_STRAND_BREAK_REPAIR_VIA_HOMOLOGOUS_RECOMBINATION	4	-1.4809521	0.0866426
Down Regulated Genes	GO_REGULATION_OF_CELLULAR_RESPONSE_TO_STRESS	17	-1.472716	0.08018868
Down Regulated Genes	GO_ATP_METABOLIC_PROCESS	6	-1.4711075	0.07067138
Down Regulated Genes	GO_SOMATIC_DIVERSIFICATION_OF_IMMUNE_RECEPTORS	7	-1.4684311	0.09075043
Down Regulated Genes	GO_MEIOTIC_CHROMOSOME_SEGREGATION	8	-1.4683483	0.082051285
Down Regulated Genes	GO_CHROMATIN_ASSEMBLY_OR_DISASSEMBLY	9	-1.468129	0.10084034
Down Regulated Genes	GO_DIGESTION	2	-1.4552907	0.05
Down Regulated Genes	GO_POSITIVE_REGULATION_OF_CELL_DIVISION	4	-1.4498014	0.08243728
Down Regulated Genes	GO_REGULATION_OF_CELL_CYCLE_ARREST	10	-1.4462839	0.10207613
Down Regulated Genes	GO_CHROMOSOME_SEGREGATION	29	-1.4402157	0.10016155
Down Regulated Genes	GO_DNA_DEPENDENT_DNA_REPLICATION	21	-1.4401351	0.09797823
Down Regulated Genes	GO_NEGATIVE_REGULATION_OF_CELL_CYCLE_G2_M_PHASE_TRANSITION	4	-1.4387081	0.088809945
Down Regulated Genes	GO_RECOMBINATIONAL_REPAIR	17	-1.4371562	0.10779436
Down Regulated Genes	GO_CYTOKINETIC_PROCESS	4	-1.4366505	0.077617325
Down Regulated Genes	GO_POSITIVE_REGULATION_OF_CYTOKINESIS	4	-1.4314071	0.08303249
Down Regulated Genes	GO_MEIOTIC_CELL_CYCLE_PROCESS	11	-1.430688	0.1127451
Down Regulated Genes	GO_MITOTIC_G1_S_TRANSITION_CHECKPOINT	8	-1.429932	0.08695652
Down Regulated Genes	GO_INORGANIC_ION_TRANSMEMBRANE_TRANSPORT	6	-1.4155298	0.11960133
Down Regulated Genes	GO_ION_TRANSMEMBRANE_TRANSPORT	9	-1.4128193	0.11960133
Down Regulated Genes	GO_CELLULAR_RESPONSE_TO_IONIZING_RADIATION	4	-1.4120873	0.09666081
Down Regulated Genes	GO_REGULATION_OF_MEIOTIC_CELL_CYCLE	2	-1.4119003	0.051823415
Down Regulated Genes	GO_ELECTRON_TRANSPORT_CHAIN	4	-1.4117447	0.10843374
Down Regulated Genes	GO_REGULATION_OF_DNA_METABOLIC_PROCESS	18	-1.409838	0.107334524
Down Regulated Genes	GO_POSITIVE_REGULATION_OF_INTRACELLULAR_PROTEIN_TRANSPORT	5	-1.4069635	0.10918544
Down Regulated Genes	GO_ANAPHASE_PROMOTING_COMPLEX_DEPENDENT_CATABOLIC_PROCESS	3	-1.4008893	0.06776557
Down Regulated Genes	GO_REGULATION_OF_DNA_REPAIR	10	-1.3971643	0.12390925
Down Regulated Genes	GO_POSITIVE_REGULATION_OF_HYDROLASE_ACTIVITY	16	-1.392291	0.1255887
Down Regulated Genes	GO_ATP_DEPENDENT_CHROMATIN_REMODELING	5	-1.3917221	0.11463845
Down Regulated Genes	GO_MEIOTIC_CELL_CYCLE_PHASE_TRANSITION	2	-1.390816	0.06859206
Down Regulated Genes	GO_SMALL_GTPASE_MEDIATED_SIGNAL_TRANSDUCTION	14	-1.3903704	0.14166667
Down Regulated Genes	GO_DNA_REPLICATION_INITIATION	10	-1.3894227	0.111692846
Down Regulated Genes	GO_DNA_BIOSYNTHETIC_PROCESS	11	-1.3891803	0.13705584
Down Regulated Genes	GO_ORGANELLE_LOCALIZATION	13	-1.3889068	0.11960133
Down Regulated Genes	GO_DNA_REPLICATION_CHECKPOINT	2	-1.384267	0.10307414
Down Regulated Genes	GO_HISTONE_EXCHANGE	5	-1.3841112	0.12982456
Down Regulated Genes	GO_DNA_REPLICATION_INDEPENDENT_NUCLEOSOME_ORGANIZATION	5	-1.3838158	0.12043796
Down Regulated Genes	GO_NEGATIVE_REGULATION_OF_ORGANELLE_ORGANIZATION	17	-1.3801322	0.13793103
Down Regulated Genes	GO_FATTY_ACID_TRANSPORT	2	-1.379438	0.08103131
Down Regulated Genes	GO_REGULATION_OF_RAS_PROTEIN_SIGNAL_TRANSDUCTION	3	-1.3789146	0.11376147
Down Regulated Genes	GO_LONG_CHAIN_FATTY_ACID_TRANSPORT	2	-1.3771712	0.07528958
Down Regulated Genes	GO_SOMATIC_DIVERSIFICATION_OF_IMMUNOGLOBULINS	4	-1.3740884	0.12523021
Down Regulated Genes	GO_PROTEIN_LOCALIZATION_TO_CYTOSKELETON	5	-1.3639209	0.13392857
Down Regulated Genes	GO_REGULATION_OF_CHROMATIN_ORGANIZATION	3	-1.3618686	0.14102565
Down Regulated Genes	GO_NEUTRAL_AMINO_ACID_TRANSPORT	1	-1.3615689	0
Down Regulated Genes	GO_POSITIVE_REGULATION_OF_CELL_POPULATION_PROLIFERATION	27	-1.3605052	0.14285715
Down Regulated Genes	GO_MITOTIC_DNA_REPLICATION_CHECKPOINT	2	-1.359679	0.09315589
Down Regulated Genes	GO_NEGATIVE_REGULATION_OF_CHROMOSOME_ORGANIZATION	10	-1.3548588	0.13986014
Down Regulated Genes	GO_MONOVALENT_INORGANIC_CATION_TRANSPORT	7	-1.3525869	0.1508772

Down Regulated Genes	GO_CATION_TRANSMEMBRANE_TRANSPORT	7	-1.352058	0.1509091
Down Regulated Genes	GO_CELL_MATURATION	2	-1.3511006	0.08365019
Down Regulated Genes	GO_NUCLEAR_CHROMOSOME_SEGREGATION	24	-1.3508544	0.12579618
Down Regulated Genes	GO_CELLULAR_COMPONENT_DISASSEMBLY	11	-1.3494095	0.15575221
Down Regulated Genes	GO_ENERGY_DERIVATION_BY_OXIDATION_OF_ORGANIC_COMPOUNDS	6	-1.3492678	0.13986014
Down Regulated Genes	GO_REGULATION_OF_PATTERN_RECOGNITION_RECEPTOR_SIGNALING_PATHWAY	4	-1.3484864	0.13105924
Down Regulated Genes	GO_CELL_AGING	3	-1.3475066	0.115517244
Down Regulated Genes	GO_CELLULAR_MACROMOLECULE_LOCALIZATION	34	-1.3442845	0.14263074
Down Regulated Genes	GO_SISTER_CHROMATID_SEGREGATION	19	-1.3432063	0.13804714
Down Regulated Genes	GO_DNA_GEOMETRIC_CHANGE	10	-1.3426895	0.14820847
Down Regulated Genes	GO_REGULATION_OF_DOUBLE_STRAND_BREAK_REPAIR	7	-1.3425673	0.16833334
Down Regulated Genes	GO_AMINO_ACID_IMPORT	1	-1.3387693	0
Down Regulated Genes	GO_ANION_TRANSPORT	8	-1.3361254	0.15555556
Down Regulated Genes	GO_PROTEIN_TRANSMEMBRANE_TRANSPORT	1	-1.3357015	0.003913894
Down Regulated Genes	GO_GLUTATHIONE_DERIVATIVE_METABOLIC_PROCESS	1	-1.3352734	0.014141414
Down Regulated Genes	GO_ESTABLISHMENT_OF_PROTEIN_LOCALIZATION_TO_ORGANELLE	8	-1.3335079	0.1524288
Down Regulated Genes	GO_POSTTRANSLATIONAL_PROTEIN_TARGETING_TO_ENDOPLASMIC_RETICULUM_MEMBRANE	1	-1.3334308	0.003802281
Down Regulated Genes	GO_ALANINE_TRANSPORT	1	-1.3327459	0
Down Regulated Genes	GO_REGULATION_OF_EMBRYONIC_DEVELOPMENT	2	-1.3282818	0.12162162
Down Regulated Genes	GO_RETROGRADE_VESICLE_MEDIATED_TRANSPORT_GOLGI_TO_ENDOPLASMIC_RETICULUM	2	-1.3264031	0.11281071
Down Regulated Genes	GO_POSTTRANSLATIONAL_PROTEIN_TARGETING_TO_MEMBRANE_TRANSLOCATION	1	-1.3216355	0.008368201
Down Regulated Genes	GO_SRP_DEPENDENT_COTRANSLATIONAL_PROTEIN_TARGETING_TO_MEMBRANE_TRANSLOCA	1	-1.3192501	0.008097166
Down Regulated Genes	GO_REGULATION_OF_TOLL_LIKE_RECEPTOR_SIGNALING_PATHWAY	4	-1.3157334	0.14901257
Down Regulated Genes	GO_MITOTIC_METAPHASE_PLATE_CONGRESSION	1	-1.314843	0.034343433
Down Regulated Genes	GO_SODIUM_ION_TRANSPORT	4	-1.3131045	0.14795008
Down Regulated Genes	GO_SULFUR_COMPOUND_TRANSPORT	2	-1.3126267	0.118421055
Down Regulated Genes	GO_METAPHASE_PLATE_CONGRESSION	1	-1.3123202	0.035433073
Down Regulated Genes	GO_PROTEIN_DEPOLYMERIZATION	3	-1.3110664	0.13953489
Down Regulated Genes	GO_SODIUM_ION_TRANSMEMBRANE_TRANSPORT	3	-1.3087078	0.14867257
Down Regulated Genes	GO_CELLULAR_RESPONSE_TO_RADIATION	6	-1.3083646	0.15734266
Down Regulated Genes	GO_DNA_ENDOREDUPLICATION	1	-1.3081899	0.04819277
Down Regulated Genes	GO_VENTRICULAR_CARDIAC_MUSCLE_CELL_DEVELOPMENT	1	-1.3076864	0.050880626
Down Regulated Genes	GO_SPROUTING_ANGIOGENESIS	1	-1.3073984	0.050301813
Down Regulated Genes	GO_CENTROMERE_COMPLEX_ASSEMBLY	4	-1.3072587	0.15277778
Down Regulated Genes	GO_POSITIVE_REGULATION_OF_OXIDATIVE_PHOSPHORYLATION	1	-1.3063097	0.0513347
Down Regulated Genes	GO_ESTABLISHMENT_OF_PROTEIN_LOCALIZATION_TO_MEMBRANE	4	-1.3052859	0.15949821
Down Regulated Genes	GO_POSITIVE_REGULATION_OF_MITOCHONDRIAL_ATP_SYNTHESIS_COUPLED_ELECTRON_TRAN	1	-1.3047434	0.054108217
Down Regulated Genes	GO_MEMBRANE_DISASSEMBLY	1	-1.3047318	0.05
Down Regulated Genes	GO_TROPHOBLAST_GIANT_CELL_DIFFERENTIATION	1	-1.3037664	0.046277665
Down Regulated Genes	GO_POSITIVE_REGULATION_OF_HEART_GROWTH	1	-1.3027269	0.050955415
Down Regulated Genes	GO_NEGATIVE_REGULATION_OF_DNA_REPAIR	1	-1.3015312	0.05222437
Down Regulated Genes	GO_REGULATION_OF_MITOCHONDRIAL_ATP_SYNTHESIS_COUPLED_ELECTRON_TRANSPORT	1	-1.3011211	0.048543688
Down Regulated Genes	GO_ATTACHMENT_OF_MITOTIC_SPINDLE_MICROTUBULES_TO_KINETOCHORE	1	-1.3006259	0.035433073
Down Regulated Genes	GO_EMBRYONIC_PLACENTA_DEVELOPMENT	1	-1.2986465	0.056420233
Down Regulated Genes	GO_SINGLE_FERTILIZATION	1	-1.2984382	0.052941177
Down Regulated Genes	GO_NEGATIVE_REGULATION_OF_DNA_RECOMBINATION	1	-1.297672	0.06534653
Down Regulated Genes	GO_POSITIVE_REGULATION_OF_MUSCLE_TISSUE_DEVELOPMENT	1	-1.2946528	0.058
Down Regulated Genes	GO_ORGANELLE_INHERITANCE	1	-1.2938364	0.057768926
Down Regulated Genes	GO_POSITIVE_REGULATION_OF_CELLULAR_RESPIRATION	1	-1.2928604	0.058939096
Down Regulated Genes	GO_EMBRYO_DEVELOPMENT_ENDING_IN_BIRTH_OR_EGG_HATCHING	8	-1.2924016	0.18612522
Down Regulated Genes	GO_POSITIVE_REGULATION_OF_CARDIAC_MUSCLE_CELL_PROLIFERATION	1	-1.291481	0.052734375
Down Regulated Genes	GO_REGULATION_OF_NEUROTRANSMITTER_LEVELS	1	-1.2912661	0.071428575
Down Regulated Genes	GO_REGULATION_OF_OXIDATIVE_PHOSPHORYLATION	1	-1.2912004	0.05719921
Down Regulated Genes	GO_RESPONSE_TO_COPPER_ION	1	-1.2908981	0.062374245
Down Regulated Genes	GO_NEGATIVE_REGULATION_OF_CELL_DIVISION	2	-1.2905668	0.12992126
Down Regulated Genes	GO_NUCLEAR_ENVELOPE_ORGANIZATION	1	-1.2902317	0.08300395
Down Regulated Genes	GO_ORGANELLE_DISASSEMBLY	1	-1.2901057	0.06138614
Down Regulated Genes	GO_POSITIVE_REGULATION_OF_CARDIAC_MUSCLE_TISSUE_DEVELOPMENT	1	-1.2895648	0.06036217
Down Regulated Genes	GO_ESTABLISHMENT_OR_MAINTENANCE_OF_CELL_POLARITY	5	-1.289395	0.1956912
Down Regulated Genes	GO_CHROMATIN_REMODELING_AT_CENTROMERE	4	-1.2893624	0.17259787
Down Regulated Genes	GO_GOLGI_DISASSEMBLY	1	-1.2892239	0.056640625
Down Regulated Genes	GO_ACTIVATION_OF_MAPK_ACTIVITY	3	-1.2890835	0.15292096
Down Regulated Genes	GO_MITOTIC_NUCLEAR_ENVELOPE_DISASSEMBLY	1	-1.287731	0.078156315
Down Regulated Genes	GO_POSITIVE_REGULATION_OF_PROTEIN_LOCALIZATION_TO_NUCLEUS	3	-1.2871898	0.16230367
Down Regulated Genes	GO_CELL_DIFFERENTIATION_INVOLVED_IN_EMBRYONIC_PLACENTA_DEVELOPMENT	1	-1.2868977	0.057377048
Down Regulated Genes	GO_ORGANELLE_FUSION	1	-1.2854016	0.065476194
Down Regulated Genes	GO_POSITIVE_REGULATION_OF_ORGAN_GROWTH	1	-1.285363	0.08282828
Down Regulated Genes	GO_HEPATOCYTE_DIFFERENTIATION	1	-1.2844226	0.04509804
Down Regulated Genes	GO_REGULATION_OF_CELLULAR_RESPIRATION	1	-1.282759	0.077922076
Down Regulated Genes	GO_NEGATIVE_REGULATION_OF_CYTOKINESIS	1	-1.2826152	0.050104383

Down Regulated Genes	GO_REGULATION_OF_PROTEIN_LOCALIZATION_TO_NUCLEUS	3	-1.2809474	0.159132
Down Regulated Genes	GO_VENTRICULAR_CARDIAC_MUSCLE_CELL_DIFFERENTIATION	1	-1.2809428	0.07723577
Down Regulated Genes	GO_MITOCHONDRIAL_GENOME_MAINTENANCE	1	-1.2809337	0.06782946
Down Regulated Genes	GO_RESPONSE_TO_AMINE	1	-1.2806962	0.06557377
Down Regulated Genes	GO_POSITIVE_REGULATION_OF_ATP_METABOLIC_PROCESS	1	-1.2793056	0.0524109
Down Regulated Genes	GO_ACTOMYOSIN_CONTRACTILE_RING_ORGANIZATION	3	-1.2789028	0.14587332
Down Regulated Genes	GO_NEGATIVE_REGULATION_OF_CHROMOSOME_SEGREGATION	6	-1.277809	0.20070423
Down Regulated Genes	GO_NEGATIVE_REGULATION_OF_DOUBLE_STRAND_BREAK_REPAIR_VIA_HOMOLOGOUS_RECOI	1	-1.2776608	0.06490872
Down Regulated Genes	GO_NEGATIVE_REGULATION_OF_SIGNAL_TRANSDUCTION_BY_P53_CLASS_MEDIATOR	1	-1.2773137	0.09689923
Down Regulated Genes	GO_CILIARY_BASAL_BODY_PLASMA_MEMBRANE_DOCKING	4	-1.2759469	0.19680852
Down Regulated Genes	GO_VITAMIN_TRANSPORT	1	-1.2746663	0.10978044
Down Regulated Genes	GO_NEUROTRANSMITTER_METABOLIC_PROCESS	1	-1.2743634	0.09022556
Down Regulated Genes	GO_DNA_PACKAGING	13	-1.2739817	0.17935702
Down Regulated Genes	GO_NUCLEAR_TRANSPORT	7	-1.2737155	0.20967741
Down Regulated Genes	GO_PHOSPHATIDYLETHANOLAMINE_BIOSYNTHETIC_PROCESS	1	-1.2731471	0.09484536
Down Regulated Genes	GO_ERYTHROCYTE_HOMEOSTASIS	1	-1.272899	0.092402466
Down Regulated Genes	GO_ESTABLISHMENT_OF_SPINDLE_ORIENTATION	2	-1.2724482	0.14044943
Down Regulated Genes	GO_POSITIVE_REGULATION_OF_LIPID_BIOSYNTHETIC_PROCESS	1	-1.2717848	0.13765182
Down Regulated Genes	GO_REGULATION_OF_TRIGLYCERIDE_BIOSYNTHETIC_PROCESS	1	-1.2710627	0.10547667
Down Regulated Genes	GO_PROTEIN_LOCALIZATION_TO_CHROMOSOME	10	-1.2696384	0.20473774
Down Regulated Genes	GO_COVALENT_CHROMATIN_MODIFICATION	10	-1.2686844	0.21018063
Down Regulated Genes	GO_POSITIVE_REGULATION_OF_MEGAKARYOCYTE_DIFFERENTIATION	1	-1.2682288	0.08853119
Down Regulated Genes	GO_REGULATION_OF_B_CELL_PROLIFERATION	1	-1.2677088	0.11608961
Down Regulated Genes	GO_NEUROTRANSMITTER_CATABOLIC_PROCESS	1	-1.2676889	0.07505071
Down Regulated Genes	GO_CELLULAR_MODIFIED_AMINO_ACID_METABOLIC_PROCESS	8	-1.2664204	0.18793103
Down Regulated Genes	GO_ATTACHMENT_OF_SPINDLE_MICROTUBULES_TO_KINETOCHORE	3	-1.2662544	0.16876122
Down Regulated Genes	GO_INTRINSIC_APOPTOTIC_SIGNALING_PATHWAY_IN_RESPONSE_TO_DNA_DAMAGE	1	-1.2658982	0.11923077
Down Regulated Genes	GO_RHYTHMIC_PROCESS	9	-1.2657638	0.2006689
Down Regulated Genes	GO_ISOTYPE_SWITCHING_TO_IGG_ISOTYPES	1	-1.2654583	0.10107527
Down Regulated Genes	GO_FATTY_ACID_TRANSMEMBRANE_TRANSPORT	1	-1.2652481	0.09919028
Down Regulated Genes	GO_NEGATIVE_REGULATION_OF_LIPID_BIOSYNTHETIC_PROCESS	1	-1.265202	0.102970295
Down Regulated Genes	GO_ESTABLISHMENT_OF_CELL_POLARITY	5	-1.265201	0.1959707
Down Regulated Genes	GO_REGULATION_OF_NEURON_MIGRATION	1	-1.2636615	0.10766046
Down Regulated Genes	GO_TRIGLYCERIDE_METABOLIC_PROCESS	1	-1.2634319	0.10778443
Down Regulated Genes	GO_PHOSPHATIDYLGLYCEROL_BIOSYNTHETIC_PROCESS	1	-1.2633667	0.092402466
Down Regulated Genes	GO_NEGATIVE_REGULATION_OF_PHOSPHOLIPID_BIOSYNTHETIC_PROCESS	1	-1.2630912	0.116
Down Regulated Genes	GO_NEGATIVE_REGULATION_OF_INTRINSIC_APOPTOTIC_SIGNALING_PATHWAY_BY_P53_CLASS	1	-1.2630403	0.1003937
Down Regulated Genes	GO_CEREBRAL_CORTEX_DEVELOPMENT	4	-1.2628237	0.19723183
Down Regulated Genes	GO_REGULATION_OF_INTRINSIC_APOPTOTIC_SIGNALING_PATHWAY_BY_P53_CLASS_MEDIATOI	1	-1.2607688	0.12601626
Down Regulated Genes	GO_POSITIVE_REGULATION_OF_TRIGLYCERIDE_BIOSYNTHETIC_PROCESS	1	-1.2596664	0.10433071
Down Regulated Genes	GO_NEGATIVE_REGULATION_OF_EXTRINSIC_APOPTOTIC_SIGNALING_PATHWAY	1	-1.2585393	0.11045365
Down Regulated Genes	GO_NEUTRAL_LIPID_METABOLIC_PROCESS	1	-1.2580628	0.1375
Down Regulated Genes	GO_INTRINSIC_APOPTOTIC_SIGNALING_PATHWAY_IN_RESPONSE_TO_DNA_DAMAGE_BY_P53_(	1	-1.2578783	0.12936345
Down Regulated Genes	GO_REGULATION_OF_PHOSPHOLIPID_BIOSYNTHETIC_PROCESS	1	-1.2569851	0.10881801
Down Regulated Genes	GO_POSITIVE_REGULATION_OF_LIPID_METABOLIC_PROCESS	1	-1.2568243	0.1237525
Down Regulated Genes	GO_NEGATIVE_REGULATION_OF_PHOSPHOLIPID_METABOLIC_PROCESS	1	-1.2566401	0.109561756
Down Regulated Genes	GO_NEUTRAL_LIPID_BIOSYNTHETIC_PROCESS	1	-1.2564881	0.12955466
Down Regulated Genes	GO_MITOTIC_CYTOKINETIC_PROCESS	1	-1.2561455	0.13061224
Down Regulated Genes	GO_PHOSPHATIDYLETHANOLAMINE_METABOLIC_PROCESS	1	-1.2560397	0.11468813
Down Regulated Genes	GO_PHOSPHATIDYLCHOLINE_BIOSYNTHETIC_PROCESS	1	-1.2558546	0.12224449
Down Regulated Genes	GO_REGULATION_OF_CELLULAR_PROTEIN_LOCALIZATION	12	-1.2554344	0.21166667
Down Regulated Genes	GO_INTESTINAL_EPITHELIAL_CELL_DIFFERENTIATION	2	-1.255331	0.1730419
Down Regulated Genes	GO_CARDIOLIPIN_METABOLIC_PROCESS	1	-1.2552166	0.12284069
Down Regulated Genes	GO_VASCULAR_TRANSPORT	1	-1.2545955	0.115913555
Down Regulated Genes	GO_LONG_CHAIN_FATTY_ACID_IMPORT_ACROSS_PLASMA_MEMBRANE	1	-1.2543828	0.11491936
Down Regulated Genes	GO_REGULATION_OF_ERYTHROCYTE_DIFFERENTIATION	1	-1.2537928	0.108949415
Down Regulated Genes	GO_MEMBRANE_DOCKING	4	-1.2533027	0.20035778
Down Regulated Genes	GO_REGULATION_OF_HEAT_GENERATION	1	-1.2530949	0.108571425
Down Regulated Genes	GO_RESPONSE_TO_RADIATION	16	-1.2530514	0.19532554
Down Regulated Genes	GO_REGULATION_OF_MITOTIC_CYTOKINESIS	1	-1.2529987	0.13450292
Down Regulated Genes	GO_POSITIVE_REGULATION_OF_TRIGLYCERIDE_METABOLIC_PROCESS	1	-1.2526376	0.13496932
Down Regulated Genes	GO_POSITIVE_REGULATION_OF_ISOTYPE_SWITCHING_TO_IGG_ISOTYPES	1	-1.2525986	0.12426036
Down Regulated Genes	GO_V_D_J_RECOMBINATION	3	-1.2525733	0.16820702
Down Regulated Genes	GO_REGULATION_OF_PHOSPHOLIPID_METABOLIC_PROCESS	1	-1.2522023	0.12355212
Down Regulated Genes	GO_NEGATIVE_REGULATION_OF_EXTRINSIC_APOPTOTIC_SIGNALING_PATHWAY_VIA_DEATH_D	1	-1.2519213	0.12266112
Down Regulated Genes	GO_PHOSPHATIDYLCHOLINE_METABOLIC_PROCESS	1	-1.2514532	0.10429448
Down Regulated Genes	GO_REGULATION_OF_TRIGLYCERIDE_METABOLIC_PROCESS	1	-1.2513726	0.11899791
Down Regulated Genes	GO_CHORIONIC_TROPHOBLAST_CELL_DIFFERENTIATION	2	-1.2512099	0.16981132
Down Regulated Genes	GO_EXPORT_ACROSS_PLASMA_MEMBRANE	1	-1.2510731	0.11740891
Down Regulated Genes	GO_REGULATION_OF_STEM_CELL_PROLIFERATION	1	-1.2509539	0.097165994

Down Regulated Genes	GO_POSITIVE_REGULATION_OF_ERYTHROCYTE_DIFFERENTIATION	1	-1.250077	0.11133603
Down Regulated Genes	GO_REGULATION_OF_INTRACELLULAR_PROTEIN_TRANSPORT	6	-1.2500662	0.21938775
Down Regulated Genes	GO_REGULATION_OF_MICROTUBULE_CYTOSKELETON_ORGANIZATION	11	-1.2488167	0.22613065
Down Regulated Genes	GO_SPERMATID_NUCLEUS_DIFFERENTIATION	1	-1.2485307	0.11610487
Down Regulated Genes	GO_VITAMIN_TRANSMEMBRANE_TRANSPORT	1	-1.2478356	0.13450292
Down Regulated Genes	GO_PHOSPHATIDYLGLYCEROL_METABOLIC_PROCESS	1	-1.2474684	0.108216435
Down Regulated Genes	GO_LIPID_TRANSPORT_ACROSS_BLOOD_BRAIN_BARRIER	1	-1.2472682	0.09542744
Down Regulated Genes	GO_HEAT_GENERATION	1	-1.247138	0.121703856
Down Regulated Genes	GO_B_CELL_PROLIFERATION	1	-1.246966	0.124223605
Down Regulated Genes	GO_REGULATION_OF_INTRINSIC_APOPTOTIC_SIGNALING_PATHWAY_IN_RESPONSE_TO_DNA_D	1	-1.2464733	0.10843374
Down Regulated Genes	GO_NEGATIVE_REGULATION_OF_INTRINSIC_APOPTOTIC_SIGNALING_PATHWAY_IN_RESPONSE_	1	-1.2457421	0.11067961
Down Regulated Genes	GO_INTRINSIC_APOPTOTIC_SIGNALING_PATHWAY_BY_P53_CLASS_MEDIATOR	1	-1.2457184	0.13207547
Down Regulated Genes	GO_NEGATIVE_REGULATION_OF_LIPID_METABOLIC_PROCESS	1	-1.2455823	0.11066398
Down Regulated Genes	GO_LIPID_IMPORT_INTO_CELL	1	-1.2451532	0.103174604
Down Regulated Genes	GO_NEURAL_TUBE_DEVELOPMENT	3	-1.2450488	0.22556391
Down Regulated Genes	GO_TRIGLYCERIDE_BIOSYNTHETIC_PROCESS	1	-1.244778	0.12948208
Down Regulated Genes	GO_POSITIVE_REGULATION_OF_HEAT_GENERATION	1	-1.24445	0.12929294
Down Regulated Genes	GO_NEURAL_TUBE_FORMATION	3	-1.2441198	0.19209039
Down Regulated Genes	GO_IMPORT_INTO_NUCLEUS	4	-1.2439637	0.20244329
Down Regulated Genes	GO_REGULATION_OF_INTRINSIC_APOPTOTIC_SIGNALING_PATHWAY_IN_RESPONSE_TO_DNA_D	1	-1.242755	0.11067194
Down Regulated Genes	GO_MITOTIC_SISTER_CHROMATID_SEGREGATION	17	-1.241903	0.21333334
Down Regulated Genes	GO_RESPONSE_TO_PROSTAGLANDIN_D	1	-1.2414957	0.14285715
Down Regulated Genes	GO_POSITIVE_REGULATION_OF_B_CELL_PROLIFERATION	1	-1.2404152	0.12185687
Down Regulated Genes	GO_POSITIVE_REGULATION_OF_NEURON_MIGRATION	1	-1.2400508	0.14020619
Down Regulated Genes	GO_CYTOSKELETON_ORGANIZATION	47	-1.2378914	0.2
Down Regulated Genes	GO_CARDIOLIPIN_BIOSYNTHETIC_PROCESS	1	-1.2365568	0.12711865
Down Regulated Genes	GO_METAL_ION_TRANSPORT	11	-1.2365359	0.22959183
Down Regulated Genes	GO_POSITIVE_REGULATION_OF_PATHWAY_RESTRICTED_SMAD_PROTEIN_PHOSPHORYLATION	1	-1.2362347	0.14728682
Down Regulated Genes	GO_POSITIVE_REGULATION_OF_DNA_REPAIR	3	-1.2357508	0.20450282
Down Regulated Genes	GO_PHOSPHATIDYLSERINE_METABOLIC_PROCESS	1	-1.2354089	0.11494253
Down Regulated Genes	GO_LEUKEMIA_INHIBITORY_FACTOR_SIGNALING_PATHWAY	1	-1.233814	0.14124294
Down Regulated Genes	GO_NEGATIVE_REGULATION_OF_EPITHELIAL_CELL_PROLIFERATION	2	-1.2333235	0.17175573
Down Regulated Genes	GO_PRIMARY_ALCOHOL_METABOLIC_PROCESS	1	-1.231271	0.14453125
Down Regulated Genes	GO_IN_UTERO_EMBRYONIC_DEVELOPMENT	5	-1.229248	0.22452505
Down Regulated Genes	GO_ONCOSTATIN_M_MEDIATED_SIGNALING_PATHWAY	1	-1.2286737	0.13452914
Down Regulated Genes	GO_INTESTINAL_EPITHELIAL_CELL_DEVELOPMENT	2	-1.228632	0.17457305
Down Regulated Genes	GO_REPRODUCTION	40	-1.2275735	0.21165644
Down Regulated Genes	GO_EXIT_FROM_MITOSIS	3	-1.2274516	0.20104896
Down Regulated Genes	GO_CORTICAL_CYTOSKELETON_ORGANIZATION	5	-1.2262783	0.20277296
Down Regulated Genes	GO_GLYCOSIDE_METABOLIC_PROCESS	1	-1.2260132	0.15106383
Down Regulated Genes	GO_TERTIARY_ALCOHOL_METABOLIC_PROCESS	1	-1.225819	0.15514019
Down Regulated Genes	GO_POSITIVE_REGULATION_OF_EPITHELIAL_CELL_PROLIFERATION	3	-1.2243757	0.19789842
Down Regulated Genes	GO_POSITIVE_REGULATION_OF_NUCLEASE_ACTIVITY	2	-1.223939	0.1879845
Down Regulated Genes	GO_OVULATION_CYCLE	2	-1.2226659	0.19360903
Down Regulated Genes	GO_POSITIVE_REGULATION_OF_RESPONSE_TO_DNA_DAMAGE_STIMULUS	3	-1.2226216	0.21195652
Down Regulated Genes	GO_PROTEIN_IMPORT	4	-1.2223015	0.2188612
Down Regulated Genes	GO_CHORION_DEVELOPMENT	2	-1.2221055	0.21731448
Down Regulated Genes	GO_FEMALE_MEIOTIC_NUCLEAR_DIVISION	1	-1.2207838	0.15555556
Down Regulated Genes	GO_MONOCARBOXYLIC_ACID_TRANSPORT	3	-1.2195065	0.21992481
Down Regulated Genes	GO_MEIOTIC_CELL_CYCLE_CHECKPOINT	1	-1.2193623	0.18218623
Down Regulated Genes	GO_PROGESTERONE_METABOLIC_PROCESS	1	-1.2192271	0.15748031
Down Regulated Genes	GO_PROTEIN_AUTOPHOSPHORYLATION	1	-1.2182407	0.15327103
Down Regulated Genes	GO_MAMMARY_GLAND_EPITHELIAL_CELL_PROLIFERATION	2	-1.2182102	0.20696326
Down Regulated Genes	GO_C21_STEROID_HORMONE_METABOLIC_PROCESS	1	-1.2176552	0.15959597
Down Regulated Genes	GO_CELLULAR_COMPONENT_MAINTENANCE	1	-1.2172272	0.18989898
Down Regulated Genes	GO_NEURON_REMODELING	1	-1.216751	0.17148362
Down Regulated Genes	GO_NEGATIVE_REGULATION_OF_METAPHASE_ANAPHASE_TRANSITION_OF_CELL_CYCLE	5	-1.2161788	0.24264705
Down Regulated Genes	GO_CILIARY_NEUROTROPHIC_FACTOR_MEDIATED_SIGNALING_PATHWAY	1	-1.2153864	0.17261904
Down Regulated Genes	GO_AMINOGLYCOSIDE_ANTIBIOTIC_METABOLIC_PROCESS	1	-1.2153718	0.16768916
Down Regulated Genes	GO_NEGATIVE_REGULATION_OF_MEIOTIC_CELL_CYCLE	1	-1.2142242	0.1820041
Down Regulated Genes	GO_CELLULAR_PROTEIN_COMPLEX_DISASSEMBLY	4	-1.213564	0.24338624
Down Regulated Genes	GO_NEGATIVE_REGULATION_OF_MEIOTIC_NUCLEAR_DIVISION	1	-1.2134372	0.168
Down Regulated Genes	GO_CELL_JUNCTION_MAINTENANCE	1	-1.21205	0.1923077
Down Regulated Genes	GO_FEMALE_MEIOSIS_CHROMOSOME_SEGREGATION	1	-1.2115418	0.14092664
Down Regulated Genes	GO_REGULATION_OF_MEIOTIC_NUCLEAR_DIVISION	1	-1.21127	0.1888668
Down Regulated Genes	GO_TRICARBOXYLIC_ACID_CYCLE	2	-1.2106141	0.20572451
Down Regulated Genes	GO_AEROBIC_RESPIRATION	2	-1.2103415	0.18773946
Down Regulated Genes	GO_EXTRAEMBRYONIC_MEMBRANE_DEVELOPMENT	2	-1.2102445	0.19480519
Down Regulated Genes	GO_MAINTENANCE_OF_SYNAPSE_STRUCTURE	1	-1.2101564	0.17450981
Down Regulated Genes	GO_PYRIMIDINE_CONTAINING_COMPOUND_METABOLIC_PROCESS	5	-1.2086533	0.2283737

Down Regulated Genes	GO_KINETOCHORE_ASSEMBLY	1	-1.2062775	0.17373738
Down Regulated Genes	GO_CATION_TRANSPORT	12	-1.2019886	0.26495728
Down Regulated Genes	GO_NEGATIVE_REGULATION_OF_NUCLEAR_DIVISION	5	-1.2018754	0.26455027
Down Regulated Genes	GO_REGULATION_OF_EXIT_FROM_MITOSIS	3	-1.2018027	0.2195572
Down Regulated Genes	GO_PROTEIN_K48_LINKED_UBIQUITINATION	1	-1.201499	0.1765835
Down Regulated Genes	GO_NEGATIVE_REGULATION_OF_TELOMERASE_ACTIVITY	2	-1.2008537	0.21072088
Down Regulated Genes	GO_HEPATOCYTE_GROWTH_FACTOR_RECEPTOR_SIGNALING_PATHWAY	1	-1.2005776	0.19672132
Down Regulated Genes	GO_MALE_SEX_DIFFERENTIATION	2	-1.2001003	0.20400728
Down Regulated Genes	GO_CELL_CYCLE_DNA_REPLICATION	13	-1.1993315	0.25460637
Down Regulated Genes	GO_CORTICAL_ACTIN_CYTOSKELETON_ORGANIZATION	5	-1.1993059	0.2508834
Down Regulated Genes	GO_POSITIVE_REGULATION_OF_BINDING	4	-1.1989973	0.25182483
Down Regulated Genes	GO_NEGATIVE_REGULATION_OF_I_KAPPAB_KINASE_NF_KAPPAB_SIGNALING	2	-1.1985255	0.20930232
Down Regulated Genes	GO_MOTOR_LEARNING	1	-1.1979431	0.1764706
Down Regulated Genes	GO_MAMMARY_GLAND_EPITHELIUM_DEVELOPMENT	2	-1.1971406	0.23597679
Down Regulated Genes	GO_NEURON_MATURATION	1	-1.1969072	0.20766129
Down Regulated Genes	GO_NON_RECOMBINATIONAL_REPAIR	5	-1.1967174	0.25183824
Down Regulated Genes	GO_PEPTIDYL_SERINE_MODIFICATION	7	-1.1957524	0.28429753
Down Regulated Genes	GO_EPITHELIAL_CELL_DIFFERENTIATION	18	-1.19364	0.25203252
Down Regulated Genes	GO_SOMITE_DEVELOPMENT	1	-1.1897223	0.20194174
Down Regulated Genes	GO_NEGATIVE_REGULATION_OF_DNA_BIOSYNTHETIC_PROCESS	2	-1.1885873	0.23571429
Down Regulated Genes	GO_RESPONSE_TO_ETHANOL	3	-1.1882851	0.24642856
Down Regulated Genes	GO_NEGATIVE_REGULATION_OF_TELOMERE_MAINTENANCE	2	-1.1880206	0.25189394
Down Regulated Genes	GO_NEGATIVE_REGULATION_OF_GUANYL_NUCLEOTIDE_EXCHANGE_FACTOR_ACTIVITY	1	-1.1869841	0.2128514
Down Regulated Genes	GO_SOMITOGENESIS	1	-1.1866728	0.21516393
Down Regulated Genes	GO_RESPONSE_TO_GAMMA_RADIATION	1	-1.1855264	0.23046093
Down Regulated Genes	GO_POSITIVE_REGULATION_OF_INTRACELLULAR_TRANSPORT	7	-1.1839339	0.27629232
Down Regulated Genes	GO_REGULATION_OF_HISTONE_MODIFICATION	2	-1.1826136	0.23376623
Down Regulated Genes	GO_REGULATION_OF_RHO_PROTEIN_SIGNAL_TRANSDUCTION	2	-1.1818899	0.2259887
Down Regulated Genes	GO_RESPONSE_TO_X_RAY	1	-1.1815888	0.2195122
Down Regulated Genes	GO_FREE_UBIQUITIN_CHAIN_POLYMERIZATION	1	-1.1815333	0.21584159
Down Regulated Genes	GO_RHO_PROTEIN_SIGNAL_TRANSDUCTION	2	-1.1808076	0.21375465
Down Regulated Genes	GO_NEGATIVE_REGULATION_OF_TELOMERE_MAINTENANCE_VIA_TELOMERE_LENGTHENING	2	-1.180634	0.21252371
Down Regulated Genes	GO_UBIQUITIN_RECYCLING	1	-1.1791596	0.21471173
Down Regulated Genes	GO_ANTERIOR_POSTERIOR_PATTERN_SPECIFICATION	1	-1.1781236	0.24260356
Down Regulated Genes	GO_NEGATIVE_REGULATION_OF_ACTIN_FILAMENT_BUNDLE_ASSEMBLY	1	-1.1779968	0.2254902
Down Regulated Genes	GO_REPLICATION_FORK_PROCESSING	3	-1.1779444	0.23191094
Down Regulated Genes	GO_REGULATION_OF_LIPID_METABOLIC_PROCESS	4	-1.1776758	0.26785713
Down Regulated Genes	GO_NEGATIVE_REGULATION_OF_TELOMERE_MAINTENANCE_VIA_TELOMERASE	2	-1.1772552	0.2368932
Down Regulated Genes	GO_NEGATIVE_REGULATION_OF_PROTEIN_BINDING	1	-1.1769874	0.23246492
Down Regulated Genes	GO_EPITHELIUM_DEVELOPMENT	30	-1.1769031	0.27831715
Down Regulated Genes	GO_PROTEIN_K11_LINKED_UBIQUITINATION	1	-1.1748472	0.2423077
Down Regulated Genes	GO_STRAND_INVASION	1	-1.1739982	0.2361111
Down Regulated Genes	GO_POSITIVE_REGULATION_OF_HISTONE_METHYLATION	2	-1.1737478	0.20039293
Down Regulated Genes	GO_POSITIVE_REGULATION_OF_CHROMATIN_ORGANIZATION	2	-1.1733348	0.2413793
Down Regulated Genes	GO_REGULATION_OF_HISTONE_METHYLATION	2	-1.1732521	0.24907748
Down Regulated Genes	GO_CHROMOSOME_LOCALIZATION	3	-1.1708015	0.24727273
Down Regulated Genes	GO_DNA_DEPENDENT_DNA_REPLICATION_MAINTENANCE_OF_FIDELITY	3	-1.1706102	0.23642732
Down Regulated Genes	GO_MEIOSIS_I_CELL_CYCLE_PROCESS	7	-1.16872	0.28621292
Down Regulated Genes	GO_REGULATION_OF_CELL_CYCLE_G1_S_PHASE_TRANSITION	12	-1.1684699	0.2944882
Down Regulated Genes	GO_PROTEIN_CONTAINING_COMPLEX_DISASSEMBLY	4	-1.1679778	0.2872928
Down Regulated Genes	GO_REGULATION_OF_WATER_LOSS_VIA_SKIN	3	-1.1666509	0.25771326
Down Regulated Genes	GO_NEGATIVE_REGULATION_OF_G_PROTEIN_COUPLED_RECEPTOR_SIGNALING_PATHWAY	1	-1.1661537	0.24489796
Down Regulated Genes	GO_NEGATIVE_REGULATION_OF_RHO_PROTEIN_SIGNAL_TRANSDUCTION	1	-1.1659952	0.26008064
Down Regulated Genes	GO_NEGATIVE_REGULATION_OF_SMALL_GTPASE_MEDIATED_SIGNAL_TRANSDUCTION	1	-1.1645864	0.26147705
Down Regulated Genes	GO_FERTILIZATION	2	-1.1639208	0.25612053
Down Regulated Genes	GO_NEGATIVE_REGULATION_OF_CANONICAL_WNT_SIGNALING_PATHWAY	1	-1.1617582	0.2689243
Down Regulated Genes	GO_GLUTATHIONE_METABOLIC_PROCESS	4	-1.1611137	0.27886322
Down Regulated Genes	GO_NEGATIVE_REGULATION_OF_INTRINSIC_APOPTOTIC_SIGNALING_PATHWAY	2	-1.1598562	0.25757575
Down Regulated Genes	GO_NEGATIVE_REGULATION_OF_TRANSLATIONAL_ELONGATION	1	-1.158737	0.2638889
Down Regulated Genes	GO_REGULATION_OF_SIGNAL_TRANSDUCTION_BY_P53_CLASS_MEDIATOR	4	-1.1551272	0.2789855
Down Regulated Genes	GO_ENDOPLASMIC_RETICULUM_TO_GOLGI_VESICLE_MEDIATED_TRANSPORT	4	-1.1514052	0.28108108
Down Regulated Genes	GO_REGULATION_OF_MICROTUBULE_BASED_PROCESS	12	-1.1511247	0.290938
Down Regulated Genes	GO_NEGATIVE_REGULATION_OF_INTRACELLULAR_SIGNAL_TRANSDUCTION	11	-1.1505368	0.31130436
Down Regulated Genes	GO_POSITIVE_REGULATION_OF_PROTEIN_KINASE_ACTIVITY	15	-1.1503057	0.2963576
Down Regulated Genes	GO_POSTREPLICATION_REPAIR	6	-1.1496897	0.29553264
Down Regulated Genes	GO_NEGATIVE_REGULATION_OF_APOPTOTIC_SIGNALING_PATHWAY	5	-1.1485327	0.28987992
Down Regulated Genes	GO_EMBRYO_DEVELOPMENT	14	-1.1483799	0.29383117
Down Regulated Genes	GO_ESTABLISHMENT_OF_ORGANELLE_LOCALIZATION	8	-1.1482508	0.295302
Down Regulated Genes	GO_NEGATIVE_REGULATION_OF_CELL_CYCLE_G1_S_PHASE_TRANSITION	11	-1.1465219	0.30831975
Down Regulated Genes	GO_PYRIMIDINE_NUCLEOTIDE_METABOLIC_PROCESS	4	-1.1458206	0.29381442

Down Regulated Genes	GO_HISTONE_PHOSPHORYLATION	2	-1.1452781	0.27737227
Down Regulated Genes	GO_REGULATION_OF_CELL_POPULATION_PROLIFERATION	36	-1.1450033	0.30171072
Down Regulated Genes	GO_SOMATIC_DIVERSIFICATION_OF_IMMUNE_RECEPTORS_VIA_SOMATIC_MUTATION	2	-1.1418227	0.29074073
Down Regulated Genes	GO_REGULATION_OF_CELL_CYCLE_CHECKPOINT	5	-1.1408238	0.32136753
Down Regulated Genes	GO_MEIOTIC_CHROMOSOME_CONDENSATION	2	-1.1406835	0.2867784
Down Regulated Genes	GO_HEPATICOBILIARY_SYSTEM_DEVELOPMENT	5	-1.1404772	0.28825623
Down Regulated Genes	GO_POSITIVE_REGULATION_OF_DOUBLE_STRAND_BREAK_REPAIR	1	-1.1403583	0.28656125
Down Regulated Genes	GO_ENDOMEMBRANE_SYSTEM_ORGANIZATION	8	-1.1402801	0.30479452
Down Regulated Genes	GO_RUFFLE_ASSEMBLY	1	-1.137421	0.29094413
Down Regulated Genes	GO_RESPONSE_TO_DIETARY_EXCESS	1	-1.1371628	0.2917505
Down Regulated Genes	GO_REGULATION_OF_LIPID_BIOSYNTHETIC_PROCESS	2	-1.1371202	0.2788104
Down Regulated Genes	GO_EATING_BEHAVIOR	1	-1.1367619	0.2997988
Down Regulated Genes	GO_REGULATION_OF_ATP_METABOLIC_PROCESS	2	-1.1354824	0.2962264
Down Regulated Genes	GO_DEOXYRIBONUCLEOTIDE_METABOLIC_PROCESS	4	-1.1331683	0.31768954
Down Regulated Genes	GO_SCHWANN_CELL_DIFFERENTIATION	2	-1.1330118	0.29608938
Down Regulated Genes	GO_REDUCTION_OF_FOOD_INTAKE_IN_RESPONSE_TO_DIETARY_EXCESS	1	-1.1329918	0.28601253
Down Regulated Genes	GO_NEGATIVE_REGULATION_OF_STRESS_ACTIVATED_PROTEIN_KINASE_SIGNALING_CASCADE	1	-1.1311218	0.31558186
Down Regulated Genes	GO_RESOLUTION_OF_MEIOTIC_RECOMBINATION_INTERMEDIATES	1	-1.128928	0.2990099
Down Regulated Genes	GO_NUCLEOSIDE_DIPHOSPHATE_METABOLIC_PROCESS	3	-1.1278931	0.27683616
Down Regulated Genes	GO_POSITIVE_REGULATION_OF_NUCLEOCYTOPLASMIC_TRANSPORT	4	-1.1265422	0.3090586
Down Regulated Genes	GO_PROTEIN_LOCALIZATION_TO_CILIUM	1	-1.1253535	0.29158512
Down Regulated Genes	GO_ACID_SECRETION	1	-1.1250901	0.32394367
Down Regulated Genes	GO_ACTIVATION_OF_PROTEIN_KINASE_ACTIVITY	11	-1.1250416	0.2923077
Down Regulated Genes	GO_DIGESTIVE_SYSTEM_PROCESS	1	-1.124864	0.32475248
Down Regulated Genes	GO_ION_TRANSPORT	17	-1.1224775	0.3392
Down Regulated Genes	GO_LONG_CHAIN_FATTY_ACID_METABOLIC_PROCESS	2	-1.121061	0.2843691
Down Regulated Genes	GO_EPITHELIAL_CELL_PROLIFERATION	9	-1.1207107	0.30511463
Down Regulated Genes	GO_ENDOSOME_ORGANIZATION	1	-1.1204549	0.32213438
Down Regulated Genes	GO_DNA_REPLICATION_DEPENDENT_NUCLEOSOME_ORGANIZATION	2	-1.120322	0.3104693
Down Regulated Genes	GO_CIRCADIAN_RHYTHM	7	-1.1199548	0.33633092
Down Regulated Genes	GO_GASTRIC_ACID_SECRETION	1	-1.119941	0.3299595
Down Regulated Genes	GO_SISTER_CHROMATID_COHESION	5	-1.1190126	0.31979695
Down Regulated Genes	GO_REGULATION_OF_CYTOSKELETON_ORGANIZATION	20	-1.1186365	0.35323384
Down Regulated Genes	GO_PYRIMIDINE_DEOXYRIBONUCLEOTIDE_METABOLIC_PROCESS	4	-1.1183608	0.3159722
Down Regulated Genes	GO_RUFFLE_ORGANIZATION	1	-1.1174222	0.3266129
Down Regulated Genes	GO_REGULATION_OF_PROTEIN_IMPORT	2	-1.1127644	0.32889733
Down Regulated Genes	GO_PROTEIN_LOCALIZATION_TO_MICROTUBULE_ORGANIZING_CENTER	2	-1.1123807	0.3056093
Down Regulated Genes	GO_MEIOTIC_SPINDLE_ORGANIZATION	1	-1.1108719	0.33460075
Down Regulated Genes	GO_NEURON_APOPTOTIC_PROCESS	3	-1.1108534	0.3232143
Down Regulated Genes	GO_POSITIVE_REGULATION_OF_MAMMARY_GLAND_EPITHELIAL_CELL_PROLIFERATION	1	-1.1098838	0.3249027
Down Regulated Genes	GO_DEFENSE_RESPONSE_TO_GRAM_NEGATIVE_BACTERIUM	2	-1.1085316	0.31850535
Down Regulated Genes	GO_PEPTIDE_METABOLIC_PROCESS	12	-1.1069713	0.34406778
Down Regulated Genes	GO_POSITIVE_REGULATION_OF_PROTEIN_IMPORT	2	-1.1061317	0.33020636
Down Regulated Genes	GO_POSITIVE_REGULATION_OF_SPINDLE_CHECKPOINT	3	-1.1059761	0.33212996
Down Regulated Genes	GO_REGULATION_OF_NUCLEOCYTOPLASMIC_TRANSPORT	5	-1.1035941	0.32490975
Down Regulated Genes	GO_REGULATION_OF_EXTRINSIC_APOPTOTIC_SIGNALING_PATHWAY	2	-1.1023349	0.34026465
Down Regulated Genes	GO_POSITIVE_REGULATION_OF_DNA_DEPENDENT_DNA_REPLICATION	3	-1.099421	0.3282313
Down Regulated Genes	GO_NUCLEIC_ACID_PHOSPHODIESTER_BOND_HYDROLYSIS	13	-1.0991343	0.35643566
Down Regulated Genes	GO_DEFENSE_RESPONSE_TO_GRAM_POSITIVE_BACTERIUM	2	-1.0988268	0.36494845
Down Regulated Genes	GO_POSITIVE_REGULATION_OF_REPRODUCTIVE_PROCESS	1	-1.0987936	0.3250478
Down Regulated Genes	GO_PROTEIN_LOCALIZATION_TO_MICROTUBULE	2	-1.0986009	0.33024117
Down Regulated Genes	GO_REGULATION_OF_EXTRINSIC_APOPTOTIC_SIGNALING_PATHWAY_VIA_DEATH_DOMAIN_RE	2	-1.0982028	0.3283859
Down Regulated Genes	GO_REGULATION_OF_ATTACHMENT_OF_SPINDLE_MICROTUBULES_TO_KINETOCHORE	2	-1.0977589	0.3371869
Down Regulated Genes	GO_NEGATIVE_REGULATION_OF_SISTER_CHROMATID_COHESION	1	-1.0951301	0.33665338
Down Regulated Genes	GO_REGULATION_OF_MAMMARY_GLAND_EPITHELIAL_CELL_PROLIFERATION	1	-1.0950232	0.3401222
Down Regulated Genes	GO_POSITIVE_REGULATION_OF_CHROMOSOME_SEPARATION	1	-1.0947142	0.34710744
Down Regulated Genes	GO_NUCLEOTIDE_PHOSPHORYLATION	3	-1.0945519	0.34558824
Down Regulated Genes	GO_REGULATION_OF_TELOMERE_MAINTENANCE	3	-1.0924431	0.34398496
Down Regulated Genes	GO_TOLL_LIKE_RECEPTOR_SIGNALING_PATHWAY	5	-1.0913624	0.35842294
Down Regulated Genes	GO_REGULATION_OF_CELL_DEATH	30	-1.0905503	0.3563403
Down Regulated Genes	GO_POSITIVE_REGULATION_OF_MEIOTIC_CELL_CYCLE	1	-1.0901719	0.37236086
Down Regulated Genes	GO_DNA_SYNTHESIS_INVOLVED_IN_DNA_REPAIR	5	-1.0899731	0.36144578
Down Regulated Genes	GO_QUINONE_METABOLIC_PROCESS	2	-1.0898157	0.35404897
Down Regulated Genes	GO_REGULATION_OF_TELOMERE_MAINTENANCE_VIA_TELOMERE_LENGTHENING	3	-1.0876509	0.33695653
Down Regulated Genes	GO_CELLULAR_RESPONSE_TO_HYDROGEN_PEROXIDE	6	-1.0875849	0.3479021
Down Regulated Genes	GO_POSITIVE_REGULATION_OF_MITOTIC_SISTER_CHROMATID_SEGREGATION	1	-1.0861255	0.36270493
Down Regulated Genes	GO_GOLGI_VESICLE_TRANSPORT	6	-1.0846181	0.3762712
Down Regulated Genes	GO_EXTRINSIC_APOPTOTIC_SIGNALING_PATHWAY_VIA_DEATH_DOMAIN_RECEPTORS	2	-1.0835984	0.3575419
Down Regulated Genes	GO_ACTIVATION_OF_GTPASE_ACTIVITY	2	-1.0829493	0.36832413
Down Regulated Genes	GO_REGULATION_OF_TELOMERASE_ACTIVITY	3	-1.079466	0.36690646

Down Regulated Genes	GO_EPITHELIAL_CELL_MATURATION	1	-1.0794214	0.39350912
Down Regulated Genes	GO_POSITIVE_REGULATION_OF_SEQUESTERING_OF_TRIGLYCERIDE	1	-1.0790845	0.37549406
Down Regulated Genes	GO_DNA_LIGATION_INVOLVED_IN_DNA_REPAIR	2	-1.0790589	0.34432235
Down Regulated Genes	GO_PYRIMIDINE_NUCLEOBASE_METABOLIC_PROCESS	1	-1.0781176	0.3600823
Down Regulated Genes	GO_POSITIVE_REGULATION_OF_DNA_BINDING	2	-1.0767876	0.3662239
Down Regulated Genes	GO_REGULATION_OF_TRANSCRIPTION_INVOLVED_IN_G1_S_TRANSITION_OF_MITOTIC_CELL_CY	3	-1.0760404	0.3833635
Down Regulated Genes	GO_CORNIFICATION	3	-1.0754768	0.37057728
Down Regulated Genes	GO_CELL_PROJECTION_ASSEMBLY	12	-1.0753794	0.36162987
Down Regulated Genes	GO_REGULATION_OF_CENTROSOME_DUPLICATION	5	-1.0736287	0.38194445
Down Regulated Genes	GO_PROTEASOMAL_PROTEIN_CATABOLIC_PROCESS	7	-1.0735604	0.34729493
Down Regulated Genes	GO_BASE_EXCISION_REPAIR	5	-1.0718856	0.36491227
Down Regulated Genes	GO_REGULATION_OF_CENTROSOME_CYCLE	5	-1.071688	0.3444056
Down Regulated Genes	GO_TELOMERE_MAINTENANCE_VIA_TELOMERE_LENGTHENING	3	-1.0711778	0.37432188
Down Regulated Genes	GO_REGULATION_OF_DNA_TEMPLATED_TRANSCRIPTION_INITIATION	2	-1.0704087	0.35227272
Down Regulated Genes	GO_REGULATION_OF_RNA_POLYMERASE_II_TRANSCRIPTION_PREINITIATION_COMPLEX_ASSEM	2	-1.0703931	0.36155203
Down Regulated Genes	GO_POSITIVE_REGULATION_OF_CELL_CYCLE_CHECKPOINT	3	-1.0703659	0.37070525
Down Regulated Genes	GO_NEGATIVE_REGULATION_OF_BIOSYNTHETIC_PROCESS	35	-1.0702882	0.39452496
Down Regulated Genes	GO_SEQUESTERING_OF_TRIGLYCERIDE	1	-1.0698972	0.39043826
Down Regulated Genes	GO_TRANSCRIPTION_PREINITIATION_COMPLEX_ASSEMBLY	2	-1.0698262	0.3833017
Down Regulated Genes	GO_REGULATION_OF_SPINDLE_CHECKPOINT	3	-1.0692797	0.38188276
Down Regulated Genes	GO_POSITIVE_REGULATION_OF_LIPID_STORAGE	2	-1.0689511	0.37453184
Down Regulated Genes	GO_DNA_TOPOLOGICAL_CHANGE	2	-1.0686558	0.38142294
Down Regulated Genes	GO_COLUMNAR_CUBOIDAL_EPITHELIAL_CELL_MATURATION	1	-1.0683235	0.38793105
Down Regulated Genes	GO_POSITIVE_REGULATION_OF_MEIOTIC_CELL_CYCLE_PHASE_TRANSITION	1	-1.0680498	0.42277992
Down Regulated Genes	GO_ENDOTHELIAL_CELL_PROLIFERATION	2	-1.0666933	0.37181997
Down Regulated Genes	GO_TORC1_SIGNALING	1	-1.0661994	0.38430583
Down Regulated Genes	GO_DUMP_METABOLIC_PROCESS	1	-1.0660441	0.40248963
Down Regulated Genes	GO_REGULATION_OF_TRANSCRIPTION_INITIATION_FROM_RNA_POLYMERASE_II_PROMOTER	2	-1.0660307	0.38533834
Down Regulated Genes	GO_REGULATION_OF_GENE_EXPRESSION_EPIGENETIC	6	-1.0655563	0.38353765
Down Regulated Genes	GO_NEGATIVE_REGULATION_OF_NUCLEOBASE_CONTAINING_COMPOUND_METABOLIC_PROCE	29	-1.0649713	0.37931034
Down Regulated Genes	GO_DNA_LIGATION	2	-1.0635774	0.3828125
Down Regulated Genes	GO_RESPONSE_TO_FOLIC_ACID	1	-1.0635238	0.4184261
Down Regulated Genes	GO_REGULATION_OF_SEQUESTERING_OF_TRIGLYCERIDE	1	-1.0629734	0.3984221
Down Regulated Genes	GO_HEART_GROWTH	3	-1.0625265	0.36229205
Down Regulated Genes	GO_RNA_3_END_PROCESSING	1	-1.0623543	0.39263803
Down Regulated Genes	GO_REGULATION_OF_ORGAN_GROWTH	3	-1.0605588	0.3448905
Down Regulated Genes	GO_NEGATIVE_REGULATION_OF_CELLULAR_COMPONENT_ORGANIZATION	22	-1.0597762	0.39660493
Down Regulated Genes	GO_MATURATION_OF_5_8S_RRNA	1	-1.0597727	0.378882
Down Regulated Genes	GO_RRNA_3_END_PROCESSING	1	-1.0597551	0.40816328
Down Regulated Genes	GO_TRANSLESION_SYNTHESIS	5	-1.0596162	0.3703041
Down Regulated Genes	GO_CARDIAC_MUSCLE_CELL_PROLIFERATION	3	-1.0591816	0.37362638
Down Regulated Genes	GO_POSITIVE_REGULATION_OF_TOR_SIGNALING	1	-1.058628	0.42519686
Down Regulated Genes	GO_NEUROGENESIS	32	-1.058231	0.38694993
Down Regulated Genes	GO_KERATINIZATION	3	-1.0581323	0.40221402
Down Regulated Genes	GO_CLEAVAGE_INVOLVED_IN_RRNA_PROCESSING	1	-1.0577993	0.4122449
Down Regulated Genes	GO_POSITIVE_REGULATION_OF_DNA_RECOMBINATION	2	-1.0577462	0.40347826
Down Regulated Genes	GO_REGULATION_OF_DEOXYRIBONUCLEASE_ACTIVITY	2	-1.0572906	0.3826923
Down Regulated Genes	GO_REGULATION_OF_TOR_SIGNALING	1	-1.0572639	0.40082645
Down Regulated Genes	GO_POSITIVE_REGULATION_OF_NIK_NF_KAPPAB_SIGNALING	2	-1.0543268	0.4050633
Down Regulated Genes	GO_POSITIVE_REGULATION_OF_LIPID_LOCALIZATION	2	-1.0532653	0.42463234
Down Regulated Genes	GO_RNA_POLYMERASE_II_PREINITIATION_COMPLEX_ASSEMBLY	2	-1.0532066	0.40699816
Down Regulated Genes	GO_REGULATION_OF_NIK_NF_KAPPAB_SIGNALING	2	-1.0520911	0.40384614
Down Regulated Genes	GO_REGULATION_OF_MUSCLE_ORGAN_DEVELOPMENT	3	-1.0520382	0.39473686
Down Regulated Genes	GO_POSITIVE_CHEMOTAXIS	2	-1.0520263	0.41847825
Down Regulated Genes	GO_POSITIVE_REGULATION_OF_B_CELL_MEDIATED_IMMUNITY	2	-1.0508863	0.42776737
Down Regulated Genes	GO_MITOCHONDRIAL_RESPIRATORY_CHAIN_COMPLEX_ASSEMBLY	1	-1.0506063	0.43505156
Down Regulated Genes	GO_POSITIVE_REGULATION_OF_ISOTYPE_SWITCHING	2	-1.0502515	0.40035906
Down Regulated Genes	GO_REGULATION_OF_GTPASE_ACTIVITY	13	-1.050196	0.41267124
Down Regulated Genes	GO_AGING	10	-1.0492953	0.39554796
Down Regulated Genes	GO_DNA_CATABOLIC_PROCESS_EXONUCLEOLYTIC	1	-1.0486035	0.4208417
Down Regulated Genes	GO_MITOCHONDRIAL_ELECTRON_TRANSPORT_NADH_TO_UBIQUINONE	1	-1.0485882	0.4396887
Down Regulated Genes	GO_NADH_DEHYDROGENASE_COMPLEX_ASSEMBLY	1	-1.0480119	0.43055555
Down Regulated Genes	GO_REGULATION_OF_TORC1_SIGNALING	1	-1.0478598	0.4311377
Down Regulated Genes	GO_NCRNA_3_END_PROCESSING	1	-1.0463765	0.43815514
Down Regulated Genes	GO_MULTICELLULAR_ORGANISM_GROWTH	4	-1.0454034	0.4051724
Down Regulated Genes	GO_MATURATION_OF_5_8S_RRNA_FROM_TRICISTRONIC_RRNA_TRANSCRIPT_SSU_RRNA_5_8S	1	-1.0451802	0.43432203
Down Regulated Genes	GO_REGULATION_OF_NUCLEASE_ACTIVITY	4	-1.0451345	0.39716312
Down Regulated Genes	GO_RRNA_METABOLIC_PROCESS	1	-1.0451125	0.44444445
Down Regulated Genes	GO_REGULATION_OF_HEART_GROWTH	3	-1.0449572	0.4442446
Down Regulated Genes	GO_REGULATION_OF_CARDIAC_MUSCLE_TISSUE_DEVELOPMENT	3	-1.0448613	0.40262172

Down Regulated Genes	GO_NEGATIVE_REGULATION_OF_CENTROSOME_CYCLE	1	-1.0432414	0.43217054
Down Regulated Genes	GO_STRIATED_MUSCLE_CELL_PROLIFERATION	3	-1.0411835	0.40733945
Down Regulated Genes	GO_POSITIVE_REGULATION_OF_ENDOTHELIAL_CELL_PROLIFERATION	2	-1.0411773	0.42447418
Down Regulated Genes	GO_LIPID_LOCALIZATION	4	-1.0402638	0.42132866
Down Regulated Genes	GO_DNA_TEMPLATED_TRANSCRIPTION_INITIATION	4	-1.0399978	0.4090909
Down Regulated Genes	GO_POSITIVE_REGULATION_OF_TORC1_SIGNALING	1	-1.0384432	0.44337812
Down Regulated Genes	GO_SULFATE_TRANSPORT	1	-1.0371633	0.42913386
Down Regulated Genes	GO_SPINDLE_MIDZONE_ASSEMBLY	1	-1.0364783	0.44128788
Down Regulated Genes	GO_TOR_SIGNALING	1	-1.0351428	0.43917525
Down Regulated Genes	GO_CELLULAR_PROTEIN_CATABOLIC_PROCESS	10	-1.0343511	0.42449665
Down Regulated Genes	GO_REGULATION_OF_ISOTYPE_SWITCHING	2	-1.0339303	0.42047533
Down Regulated Genes	GO_SPINDLE_ELONGATION	1	-1.03373	0.42479676
Down Regulated Genes	GO_RESPONSE_TO_AXON_INJURY	3	-1.0320002	0.42595977
Down Regulated Genes	GO_SOMATIC_RECOMBINATION_OF_IMMUNOGLOBULIN_GENE_SEGMENTS	3	-1.0314378	0.38963532
Down Regulated Genes	GO_REGULATION_OF_GENERATION_OF_PRECURSOR_METABOLITES_AND_ENERGY	3	-1.0309248	0.41621622
Down Regulated Genes	GO_TRANSCRIPTION_INITIATION_FROM_RNA_POLYMERASE_II_PROMOTER	4	-1.0299551	0.42031524
Down Regulated Genes	GO_RE_ENTRY_INTO_MITOTIC_CELL_CYCLE	1	-1.0291569	0.45325205
Down Regulated Genes	GO_REGULATION_OF_INTRACELLULAR_SIGNAL_TRANSDUCTION	39	-1.0270847	0.41294298
Down Regulated Genes	GO_CELLULAR_PROCESS_INVOLVED_IN_REPRODUCTION_IN_MULTICELLULAR_ORGANISM	5	-1.0263064	0.41624364
Down Regulated Genes	GO_ADIPONECTIN_ACTIVATED_SIGNALING_PATHWAY	2	-1.0257813	0.43930635
Down Regulated Genes	GO_NEGATIVE_REGULATION_OF_MYOBLAST_DIFFERENTIATION	1	-1.025758	0.4681725
Down Regulated Genes	GO_REGULATION_OF_B_CELL_MEDIATED_IMMUNITY	2	-1.0256969	0.43659043
Down Regulated Genes	GO_CELL_AGGREGATION	1	-1.024504	0.44680852
Down Regulated Genes	GO_REGULATION_OF_REPRODUCTIVE_PROCESS	5	-1.0228249	0.43654823
Down Regulated Genes	GO_PERIPHERAL_NERVOUS_SYSTEM_DEVELOPMENT	3	-1.0212256	0.41480207
Down Regulated Genes	GO_TUBE_FORMATION	4	-1.0211126	0.43106458
Down Regulated Genes	GO_DNA_CATABOLIC_PROCESS	4	-1.0210216	0.43782836
Down Regulated Genes	GO_REGULATION_OF_MYOBLAST_DIFFERENTIATION	1	-1.0202513	0.44468084
Down Regulated Genes	GO_CELLULAR_MACROMOLECULE_CATABOLIC_PROCESS	18	-1.0201812	0.43492064
Down Regulated Genes	GO_REGULATION_OF_HYDROLASE_ACTIVITY	23	-1.0200232	0.44012442
Down Regulated Genes	GO_MITOTIC_SPINDLE_ASSEMBLY	1	-1.019833	0.48490945
Down Regulated Genes	GO_PEPTIDYL_LYSINE_METHYLATION	5	-1.0196842	0.44405594
Down Regulated Genes	GO_NEGATIVE_REGULATION_OF_CELLULAR_AMIDE_METABOLIC_PROCESS	6	-1.0181655	0.44522968
Down Regulated Genes	GO_MITOTIC_SPINDLE_MIDZONE_ASSEMBLY	1	-1.0180379	0.47974414
Down Regulated Genes	GO_PTERIDINE_CONTAINING_COMPOUND_METABOLIC_PROCESS	3	-1.0171245	0.43063062
Down Regulated Genes	GO_CARBOHYDRATE_CATABOLIC_PROCESS	3	-1.0166953	0.4526316
Down Regulated Genes	GO_T_CELL_DIFFERENTIATION_IN_THYMUS	2	-1.0161828	0.44423077
Down Regulated Genes	GO_POSITIVE_REGULATION_OF_GTPASE_ACTIVITY	11	-1.0153502	0.42021278
Down Regulated Genes	GO_FOLIC_ACID_CONTAINING_COMPOUND_METABOLIC_PROCESS	3	-1.0150971	0.43726936
Down Regulated Genes	GO_EPITHELIAL_TUBE_FORMATION	4	-1.0133392	0.4432624
Down Regulated Genes	GO_REGULATION_OF_DNA_DEPENDENT_DNA_REPLICATION	6	-1.0116937	0.4460177
Down Regulated Genes	GO_CILIUM_ORGANIZATION	10	-1.0116789	0.43021345
Down Regulated Genes	GO_BLEB_ASSEMBLY	1	-1.00931	0.4770459
Down Regulated Genes	GO_CELL_PROJECTION_ORGANIZATION	25	-1.0080914	0.4869281
Down Regulated Genes	GO_NLRP3_INFLAMMASOME_COMPLEX_ASSEMBLY	1	-1.0067126	0.45550847
Down Regulated Genes	GO_NEURON_DEATH	5	-1.0062118	0.44148937
Down Regulated Genes	GO_REGULATION_OF_STRESS_ACTIVATED_PROTEIN_KINASE_SIGNALING_CASCADE	4	-1.0058131	0.4537522
Down Regulated Genes	GO_CENTROSOME_DUPLICATION	8	-1.004911	0.43316412
Down Regulated Genes	GO_SECONDARY_METABOLIC_PROCESS	2	-1.0044923	0.45131087
Down Regulated Genes	GO_MORPHOGENESIS_OF_EMBRYONIC_EPITHELIUM	4	-1.0044092	0.46494466
Down Regulated Genes	GO_ENDOPLASMIC_RETICULUM_UNFOLDED_PROTEIN_RESPONSE	1	-1.0043173	0.50392157
Down Regulated Genes	GO_DEOXYRIBOSE_PHOSPHATE_BIOSYNTHETIC_PROCESS	3	-1.0026256	0.45789474
Down Regulated Genes	GO_ANATOMICAL_STRUCTURE_MATURATION	3	-1.0025423	0.45818815
Down Regulated Genes	GO_RESPONSE_TO_TOPOLOGICALLY_INCORRECT_PROTEIN	1	-1.0023389	0.496
Down Regulated Genes	GO_FEMALE_GAMETE_GENERATION	2	-0.9995602	0.47082496
Down Regulated Genes	GO_TETRAHYDROFOLATE_METABOLIC_PROCESS	3	-0.9995075	0.43738317
Down Regulated Genes	GO_REGULATION_OF_BINDING	6	-0.9990077	0.4540636
Down Regulated Genes	GO_ONE_CARBON_METABOLIC_PROCESS	3	-0.9986698	0.44160584
Down Regulated Genes	GO_CELLULAR_RESPONSE_TO_BACTERIAL_LIPOPROTEIN	1	-0.9982545	0.48039216
Down Regulated Genes	GO_POSITIVE_REGULATION_OF_GROWTH	4	-0.997962	0.47750866
Down Regulated Genes	GO_CELLULAR_RESPONSE_TO_AMYLOID_BETA	1	-0.9958864	0.4880478
Down Regulated Genes	GO_EPITHELIAL_TUBE_MORPHOGENESIS	6	-0.9956431	0.4602076
Down Regulated Genes	GO_PYRIMIDINE_DEOXYRIBONUCLEOTIDE_BIOSYNTHETIC_PROCESS	3	-0.9955919	0.4632353
Down Regulated Genes	GO_NEGATIVE_REGULATION_OF_INTERLEUKIN_8_PRODUCTION	1	-0.9954772	0.4990099
Down Regulated Genes	GO_POSITIVE_REGULATION_OF_HISTONE_H3_K27_METHYLATION	1	-0.99543643	0.5041322
Down Regulated Genes	GO_PROTEIN_METHYLATION	5	-0.9948223	0.4622144
Down Regulated Genes	GO_CELLULAR_RESPONSE_TO_TOPOLOGICALLY_INCORRECT_PROTEIN	1	-0.99419534	0.49905124
Down Regulated Genes	GO_RESPONSE_TO_LEUKEMIA_INHIBITORY_FACTOR	3	-0.99406844	0.4721689
Down Regulated Genes	GO_RESPONSE_TO_ENDOPLASMIC_RETICULUM_STRESS	1	-0.99352974	0.5
Down Regulated Genes	GO_PYRIMIDINE_NUCLEOTIDE_BIOSYNTHETIC_PROCESS	3	-0.9935205	0.45636365

Down Regulated Genes	GO_DETECTION_OF_BACTERIAL_LIPOPROTEIN	1	-0.9926137	0.49083504
Down Regulated Genes	GO_PROTEIN_LOCALIZATION_TO_CHROMATIN	5	-0.9925546	0.48376068
Down Regulated Genes	GO_PYRIMIDINE_CONTAINING_COMPOUND_BIOSYNTHETIC_PROCESS	3	-0.99135244	0.48920864
Down Regulated Genes	GO_APOPTOTIC_PROCESS	38	-0.9910797	0.48402557
Down Regulated Genes	GO_POSITIVE_REGULATION_OF_NF_KAPPAB_TRANSCRIPTION_FACTOR_ACTIVITY	1	-0.9893969	0.5
Down Regulated Genes	GO_DEOXYRIBONUCLEOTIDE_BIOSYNTHETIC_PROCESS	3	-0.98926044	0.48758864
Down Regulated Genes	GO_POSITIVE_REGULATION_OF_NLRP3_INFLAMMASOME_COMPLEX_ASSEMBLY	1	-0.98919445	0.5233463
Down Regulated Genes	GO_REGULATION_OF_CIRCADIAN_RHYTHM	5	-0.9889762	0.46643108
Down Regulated Genes	GO_COLUMNAR_CUBOIDAL_EPITHELIAL_CELL_DIFFERENTIATION	3	-0.988925	0.4642202
Down Regulated Genes	GO_TOLL_LIKE_RECEPTOR_2_SIGNALING_PATHWAY	1	-0.98743427	0.51307845
Down Regulated Genes	GO_NEURON_DEATH_IN_RESPONSE_TO_OXIDATIVE_STRESS	1	-0.9874204	0.515748
Down Regulated Genes	GO_POSITIVE_REGULATION_OF_OXIDATIVE_STRESS_INDUCED_CELL_DEATH	1	-0.98604625	0.5189394
Down Regulated Genes	GO_POSITIVE_REGULATION_OF_CENTROSOME_CYCLE	1	-0.9846277	0.5289421
Down Regulated Genes	GO_NEGATIVE_REGULATION_OF_PROTEIN_POLYMERIZATION	4	-0.98408544	0.5
Down Regulated Genes	GO_INTERSTRAND_CROSS_LINK_REPAIR	6	-0.9840492	0.47627416
Down Regulated Genes	GO_NUCLEOSIDE_TRIPHOSPHATE_METABOLIC_PROCESS	2	-0.98381627	0.49619773
Down Regulated Genes	GO_POSITIVE_REGULATION_OF_RESPONSE_TO_OXIDATIVE_STRESS	1	-0.9836049	0.54527557
Down Regulated Genes	GO_CELLULAR_RESPONSE_TO_CHEMICAL_STRESS	12	-0.98332256	0.47202796
Down Regulated Genes	GO_LUNG_EPITHELIUM_DEVELOPMENT	1	-0.9829634	0.5
Down Regulated Genes	GO_COLUMNAR_CUBOIDAL_EPITHELIAL_CELL_DEVELOPMENT	3	-0.9827853	0.496337
Down Regulated Genes	GO_CELLULAR_MODIFIED_AMINO_ACID_BIOSYNTHETIC_PROCESS	4	-0.98276925	0.48175183
Down Regulated Genes	GO_LUNG_SECRETORY_CELL_DIFFERENTIATION	1	-0.98266095	0.5178197
Down Regulated Genes	GO_REGULATION_OF_INTRACELLULAR_TRANSPORT	9	-0.98232716	0.46205732
Down Regulated Genes	GO_HISTONE_METHYLATION	5	-0.98209745	0.4775281
Down Regulated Genes	GO_REGULATION_OF_HISTONE_H3_K27_METHYLATION	1	-0.98166144	0.56224066
Down Regulated Genes	GO_REGULATION_OF_OXIDATIVE_STRESS_INDUCED_CELL_DEATH	1	-0.98107094	0.53154874
Down Regulated Genes	GO_POSITIVE_REGULATION_OF_NITRIC_OXIDE_METABOLIC_PROCESS	1	-0.98078305	0.5160643
Down Regulated Genes	GO_RESPONSE_TO_DIACYL_BACTERIAL_LIPOPEPTIDE	1	-0.98058426	0.54008436
Down Regulated Genes	GO_NEGATIVE_REGULATION_OF_CELL_FATE_COMMITMENT	1	-0.9805174	0.51704544
Down Regulated Genes	GO_ACTIVATION_OF_NF_KAPPAB_INDUCING_KINASE_ACTIVITY	1	-0.9800518	0.5233266
Down Regulated Genes	GO_REGULATION_OF_PROTEIN_SERINE_THREONINE_KINASE_ACTIVITY	19	-0.9796426	0.48504984
Down Regulated Genes	GO_PROTEIN_POLYMERIZATION	9	-0.97952133	0.48305085
Down Regulated Genes	GO_REGULATION_OF_NITRIC_OXIDE_BIOSYNTHETIC_PROCESS	1	-0.9792849	0.53549695
Down Regulated Genes	GO_REGULATION_OF_UBIQUITIN_PROTEIN_TRANSFERASE_ACTIVITY	2	-0.9791637	0.5137255
Down Regulated Genes	GO_REGULATION_OF_REACTIVE_OXYGEN_SPECIES_BIOSYNTHETIC_PROCESS	1	-0.9786183	0.5517891
Down Regulated Genes	GO_CELLULAR_RESPONSE_TO_LIPOPROTEIN_PARTICLE_STIMULUS	1	-0.97860324	0.52129817
Down Regulated Genes	GO_REACTIVE_NITROGEN_SPECIES_METABOLIC_PROCESS	1	-0.9784946	0.52991456
Down Regulated Genes	GO_RESPONSE_TO_AMYLOID_BETA	1	-0.97790664	0.52208835
Down Regulated Genes	GO_REGULATION_OF_TOLL_LIKE_RECEPTOR_2_SIGNALING_PATHWAY	1	-0.9773695	0.5250501
Down Regulated Genes	GO_DETECTION_OF_MOLECULE_OF_BACTERIAL_ORIGIN	1	-0.9770686	0.55467194
Down Regulated Genes	GO_NEGATIVE_REGULATION_OF_BINDING	2	-0.977051	0.51717556
Down Regulated Genes	GO_POSITIVE_REGULATION_OF_OXIDATIVE_STRESS_INDUCED_NEURON_DEATH	1	-0.97696024	0.5502092
Down Regulated Genes	GO_REGULATION_OF_G_PROTEIN_COUPLED_RECEPTOR_SIGNALING_PATHWAY	2	-0.9762055	0.49304175
Down Regulated Genes	GO_POSITIVE_REGULATION_OF_REACTIVE_OXYGEN_SPECIES_BIOSYNTHETIC_PROCESS	1	-0.97618854	0.525641
Down Regulated Genes	GO_REACTIVE_OXYGEN_SPECIES_BIOSYNTHETIC_PROCESS	1	-0.97577935	0.54598826
Down Regulated Genes	GO_PRE_REPLICATIVE_COMPLEX_ASSEMBLY_INVOLVED_IN_CELL_CYCLE_DNA_REPLICATION	4	-0.97525346	0.4786642
Down Regulated Genes	GO_NON_MOTILE_CILIUM_ASSEMBLY	1	-0.97322357	0.556
Down Regulated Genes	GO_MYOBLAST_DIFFERENTIATION	2	-0.97319835	0.53144014
Down Regulated Genes	GO_NEGATIVE_REGULATION_OF_TOLL_LIKE_RECEPTOR_SIGNALING_PATHWAY	1	-0.9731515	0.54013014
Down Regulated Genes	GO_REGULATION_OF_UBIQUITIN_PROTEIN_LIGASE_ACTIVITY	2	-0.9728477	0.5196998
Down Regulated Genes	GO_NEGATIVE_REGULATION_OF_TOLL_LIKE_RECEPTOR_2_SIGNALING_PATHWAY	1	-0.9728251	0.53740156
Down Regulated Genes	GO_NEGATIVE_REGULATION_OF_CELL_DEATH	16	-0.9722146	0.48679867
Down Regulated Genes	GO_DOUBLE_STRAND_BREAK_REPAIR_VIA_BREAK_INDUCED_REPLICATION	4	-0.9710634	0.50545454
Down Regulated Genes	GO_REGULATION_OF_DNA_DIRECTED_DNA_POLYMERASE_ACTIVITY	1	-0.9709965	0.53692615
Down Regulated Genes	GO_CELLULAR_RESPONSE_TO_OXIDISED_LOW_DENSITY_LIPOPROTEIN_PARTICLE_STIMULUS	1	-0.97075385	0.55982906
Down Regulated Genes	GO_ESTROUS_CYCLE	1	-0.9704077	0.53578734
Down Regulated Genes	GO_LOBAR_BRONCHUS_DEVELOPMENT	1	-0.97039855	0.54805726
Down Regulated Genes	GO_MUSCLE_CELL_PROLIFERATION	4	-0.9702499	0.4954296
Down Regulated Genes	GO_PHOSPHATIDYLINOSITOL_METABOLIC_PROCESS	3	-0.96991676	0.49723756
Down Regulated Genes	GO_POSITIVE_REGULATION_OF_CENTROSOME_DUPLICATION	1	-0.9695351	0.53386456
Down Regulated Genes	GO_LUNG_CELL_DIFFERENTIATION	1	-0.96946657	0.53252035
Down Regulated Genes	GO_NEGATIVE_REGULATION_OF_CYTOSKELETON_ORGANIZATION	6	-0.9687875	0.48181817
Down Regulated Genes	GO_NEGATIVE_REGULATION_OF_REPRODUCTIVE_PROCESS	2	-0.9686722	0.53667957
Down Regulated Genes	GO_DEOXYRIBONUCLEOSIDE_TRIPHOSPHATE_BIOSYNTHETIC_PROCESS	2	-0.96741176	0.5414258
Down Regulated Genes	GO_PHOSPHATIDYLINOSITOL_BIOSYNTHETIC_PROCESS	3	-0.9673951	0.5046904
Down Regulated Genes	GO_PYRIMIDINE_NUCLEOSIDE_TRIPHOSPHATE_BIOSYNTHETIC_PROCESS	2	-0.9673457	0.5319549
Down Regulated Genes	GO_RESPONSE_TO_BACTERIAL_LIPOPROTEIN	1	-0.9663811	0.57228917
Down Regulated Genes	GO_ESTABLISHMENT_OF_TISSUE_POLARITY	1	-0.96597415	0.5417559
Down Regulated Genes	GO_PYRIMIDINE_DEOXYRIBONUCLEOSIDE_TRIPHOSPHATE_METABOLIC_PROCESS	2	-0.964776	0.52747256
Down Regulated Genes	GO_POSITIVE_REGULATION_OF_UBIQUITIN_PROTEIN_LIGASE_ACTIVITY	2	-0.9643826	0.54191035

Down Regulated Genes	GO_FEMALE_GENITALIA_DEVELOPMENT	1	-0.96388984	0.5300207
Down Regulated Genes	GO_MYD88_DEPENDENT_TOLL_LIKE_RECEPTOR_SIGNALING_PATHWAY	1	-0.9638702	0.57058823
Down Regulated Genes	GO_DEOXYRIBONUCLEOSIDE_TRIPHOSPHATE_METABOLIC_PROCESS	2	-0.96333146	0.5315315
Down Regulated Genes	GO_RESPONSE_TO_L_GLUTAMATE	1	-0.9632009	0.53606236
Down Regulated Genes	GO_NUCLEOBASE_CONTAINING_SMALL_MOLECULE_INTERCONVERSION	2	-0.9631431	0.5325885
Down Regulated Genes	GO_REGULATION_OF_CELL_FATE_COMMITMENT	1	-0.9626628	0.5613682
Down Regulated Genes	GO_LOBAR_BRONCHUS_EPITHELIUM_DEVELOPMENT	1	-0.96226126	0.5490982
Down Regulated Genes	GO_BASE_EXCISION_REPAIR_GAP_FILLING	1	-0.9615985	0.5681382
Down Regulated Genes	GO_PALLIUM_DEVELOPMENT	7	-0.9606674	0.48907563
Down Regulated Genes	GO_POSITIVE_REGULATION_OF_CELL_FATE_COMMITMENT	1	-0.9601264	0.575
Down Regulated Genes	GO_MORPHOGENESIS_OF_A_POLARIZED_EPITHELIUM	1	-0.96005195	0.5714286
Down Regulated Genes	GO_POSITIVE_REGULATION_OF_PROTEIN_KINASE_B_SIGNALING	3	-0.96003026	0.5313059
Down Regulated Genes	GO_POSITIVE_REGULATION_OF_DEVELOPMENTAL_GROWTH	4	-0.9598431	0.50626117
Down Regulated Genes	GO_POSITIVE_REGULATION_OF_MOLECULAR_FUNCTION	38	-0.95932484	0.53745925
Down Regulated Genes	GO_SEGMENTATION	2	-0.9576345	0.5409836
Down Regulated Genes	GO_POSITIVE_REGULATION_OF_UBIQUITIN_PROTEIN_TRANSFERASE_ACTIVITY	2	-0.9570229	0.53333336
Down Regulated Genes	GO_REGULATION_OF_SISTER_CHROMATID_COHESION	3	-0.9565534	0.5224719
Down Regulated Genes	GO_POSITIVE_REGULATION_OF_DEOXYRIBONUCLEASE_ACTIVITY	1	-0.9556359	0.55772996
Down Regulated Genes	GO_GENITALIA_DEVELOPMENT	1	-0.95529616	0.5625
Down Regulated Genes	GO_CELLULAR_RESPONSE_TO_ESTROGEN_STIMULUS	1	-0.9544565	0.57389635
Down Regulated Genes	GO_DNA_REPLICATION_SYNTHESIS_OF_RNA_PRIMER	1	-0.9529231	0.5694165
Down Regulated Genes	GO_NEURAL_CREST_CELL_DIFFERENTIATION	1	-0.952848	0.54598826
Down Regulated Genes	GO_REGULATION_OF_MORPHOGENESIS_OF_A_BRANCHING_STRUCTURE	1	-0.9524702	0.55711424
Down Regulated Genes	GO_ANTRAL_OVARIAN_FOLLICLE_GROWTH	1	-0.951308	0.5685484
Down Regulated Genes	GO_PYRIMIDINE_NUCLEOSIDE_TRIPHOSPHATE_METABOLIC_PROCESS	2	-0.95081705	0.5544933
Down Regulated Genes	GO_PROSTATE_GLANDULAR_ACINUS_DEVELOPMENT	1	-0.94997513	0.5700197
Down Regulated Genes	GO_BRANCH_ELONGATION_OF_AN_EPITHELIUM	1	-0.94991624	0.5481928
Down Regulated Genes	GO_POSITIVE_REGULATION_OF_MACROPHAGE_ACTIVATION	2	-0.9496801	0.5329341
Down Regulated Genes	GO_BRANCHING_INVOLVED_IN_PROSTATE_GLAND_MORPHOGENESIS	1	-0.94953454	0.5626283
Down Regulated Genes	GO_RNA_DEPENDENT_DNA_BIOSYNTHETIC_PROCESS	4	-0.9492425	0.5154639
Down Regulated Genes	GO_RNA_SPLICING_VIA_TRANSESTERIFICATION_REACTIONS	3	-0.94902617	0.5233813
Down Regulated Genes	GO_MODIFICATION_DEPENDENT_MACROMOLECULE_CATABOLIC_PROCESS	9	-0.94845295	0.53157896
Down Regulated Genes	GO_MULTICELLULAR_ORGANISMAL_HOMEOSTASIS	10	-0.9482015	0.5236486
Down Regulated Genes	GO_BRANCHING_INVOLVED_IN_MAMMARY_GLAND_DUCT_MORPHOGENESIS	1	-0.94781965	0.57258064
Down Regulated Genes	GO_NEGATIVE_REGULATION_OF_GENE_EXPRESSION_EPIGENETIC	4	-0.94776934	0.5190972
Down Regulated Genes	GO_POSITIVE_REGULATION_OF_FIBROBLAST_PROLIFERATION	1	-0.94608396	0.570297
Down Regulated Genes	GO_PHOSPHOLIPASE_C_ACTIVATING_G_PROTEIN_COUPLED_RECEPTOR_SIGNALING_PATHWAY	1	-0.94595116	0.5970149
Down Regulated Genes	GO_CHEMOREPULSION_OF_AXON	1	-0.9454931	0.59615386
Down Regulated Genes	GO_NEURAL_CREST_CELL_MIGRATION	1	-0.9448233	0.5908142
Down Regulated Genes	GO_REGULATION_OF_LIPASE_ACTIVITY	1	-0.94477075	0.5813008
Down Regulated Genes	GO_NUCLEOSIDE_TRIPHOSPHATE_BIOSYNTHETIC_PROCESS	2	-0.9447676	0.560396
Down Regulated Genes	GO_OVULATION_CYCLE_PROCESS	1	-0.9442096	0.590535
Down Regulated Genes	GO_MAMMARY_GLAND_BRANCHING_INVOLVED_IN_PREGNANCY	1	-0.9436149	0.5728543
Down Regulated Genes	GO_POSITIVE_REGULATION_OF_RHO_PROTEIN_SIGNAL_TRANSDUCTION	1	-0.94307214	0.5747573
Down Regulated Genes	GO_ERROR_PRONE_TRANSLESION_SYNTHESIS	3	-0.9430602	0.5502742
Down Regulated Genes	GO_PROSTATE_GLAND_MORPHOGENESIS	1	-0.9428651	0.5790514
Down Regulated Genes	GO_PROSTATE_GLANDULAR_ACINUS_MORPHOGENESIS	1	-0.9428108	0.5954825
Down Regulated Genes	GO_UTERUS_DEVELOPMENT	1	-0.94256824	0.5801527
Down Regulated Genes	GO_NEGATIVE_REGULATION_OF_HISTONE_MODIFICATION	1	-0.9425578	0.56726456
Down Regulated Genes	GO_REGULATION_OF_BRANCHING_INVOLVED_IN_PROSTATE_GLAND_MORPHOGENESIS	1	-0.9425406	0.5813449
Down Regulated Genes	GO_POSITIVE_REGULATION_OF_DNA_TEMPLATED_TRANSCRIPTION_INITIATION	1	-0.9425354	0.59081835
Down Regulated Genes	GO_DIGESTIVE_SYSTEM_DEVELOPMENT	3	-0.94235516	0.55
Down Regulated Genes	GO_PROTEIN_LOCALIZATION_TO_NUCLEUS	7	-0.9423066	0.5154265
Down Regulated Genes	GO_RNA_SPLICING	3	-0.94172007	0.530303
Down Regulated Genes	GO_AXIS_ELONGATION	1	-0.9413517	0.6026871
Down Regulated Genes	GO_REGULATION_OF_PHOSPHOLIPASE_C_ACTIVITY	1	-0.9412667	0.58627856
Down Regulated Genes	GO_MAMMARY_GLAND_DUCT_MORPHOGENESIS	1	-0.94124997	0.5872093
Down Regulated Genes	GO_POSITIVE_REGULATION_OF_RNA_POLYMERASE_II_TRANSCRIPTION_PREINITIATION_COMPL	1	-0.94108444	0.59807694
Down Regulated Genes	GO_REGULATION_OF_GTP_BINDING	2	-0.9404064	0.5894539
Down Regulated Genes	GO_ANDROGEN_METABOLIC_PROCESS	1	-0.94022816	0.5866935
Down Regulated Genes	GO_FIBROBLAST_PROLIFERATION	1	-0.94013405	0.6
Down Regulated Genes	GO_VAGINA_DEVELOPMENT	1	-0.940072	0.5699797
Down Regulated Genes	GO_POSITIVE_REGULATION_OF_TRANSCRIPTION_INITIATION_FROM_RNA_POLYMERASE_II_PRC	1	-0.9399518	0.6103647
Down Regulated Genes	GO_REGULATION_OF_HISTONE_H3_K4_METHYLATION	1	-0.9393462	0.5660377
Down Regulated Genes	GO_REGULATION_OF_CYCLIN_DEPENDENT_PROTEIN_KINASE_ACTIVITY	7	-0.9391288	0.4964539
Down Regulated Genes	GO_REGULATION_OF_TRANSLATIONAL_ELONGATION	2	-0.9382729	0.55932206
Down Regulated Genes	GO_POSITIVE_REGULATION_OF_CHROMOSOME_SEGREGATION	3	-0.9380922	0.5471698
Down Regulated Genes	GO_MAMMARY_GLAND_ALVEOLUS_DEVELOPMENT	1	-0.9380229	0.6063618
Down Regulated Genes	GO_SPINDLE_ASSEMBLY	3	-0.9375659	0.5405904
Down Regulated Genes	GO_CELLULAR_RESPONSE_TO_ESTRADIOL_STIMULUS	1	-0.93748415	0.608

Down Regulated Genes	GO_HOMOLOGOUS_RECOMBINATION	2	-0.9374149	0.5652985
Down Regulated Genes	GO_PHAGOCYTOSIS	4	-0.93541694	0.5162455
Down Regulated Genes	GO_REGULATION_OF_PHOSPHOLIPASE_ACTIVITY	1	-0.9345919	0.61473686
Down Regulated Genes	GO_CELLULAR_RESPONSE_TO_INCREASED_OXYGEN_LEVELS	1	-0.9335185	0.59196615
Down Regulated Genes	GO_POSITIVE_REGULATION_OF_CATALYTIC_ACTIVITY	35	-0.93320924	0.5547112
Down Regulated Genes	GO_REGULATION_OF_DNA_BIOSYNTHETIC_PROCESS	4	-0.9325684	0.5626102
Down Regulated Genes	GO_POSITIVE_REGULATION_OF_SMALL_GTPASE_MEDIATED_SIGNAL_TRANSDUCTION	1	-0.930771	0.60946745
Down Regulated Genes	GO_REGULATION_OF_PROTEIN_MODIFICATION_BY_SMALL_PROTEIN_CONJUGATION_OR_REM(	4	-0.93063086	0.55376345
Down Regulated Genes	GO_POSITIVE_REGULATION_OF_PHOSPHOLIPASE_ACTIVITY	1	-0.9303743	0.607438
Down Regulated Genes	GO_REGULATION_OF_GUANYL_NUCLEOTIDE_EXCHANGE_FACTOR_ACTIVITY	2	-0.92948055	0.5864078
Down Regulated Genes	GO_HISTONE_H3_K4_METHYLATION	1	-0.92865425	0.60288066
Down Regulated Genes	GO_NEGATIVE_REGULATION_OF_CHROMATIN_ORGANIZATION	1	-0.92682886	0.5800781
Down Regulated Genes	GO_DEVELOPMENTAL_GROWTH	16	-0.9250733	0.5561139
Down Regulated Genes	GO_GROWTH	18	-0.9249324	0.56333333
Down Regulated Genes	GO_PROTEIN_LOCALIZATION_TO_MEMBRANE	8	-0.92391086	0.5410122
Down Regulated Genes	GO_OVARIAN_FOLLICLE_DEVELOPMENT	2	-0.9238096	0.6076923
Down Regulated Genes	GO_POSITIVE_REGULATION_OF_LIPASE_ACTIVITY	1	-0.9235231	0.63965887
Down Regulated Genes	GO_NEGATIVE_REGULATION_OF_HISTONE_METHYLATION	1	-0.92328846	0.6
Down Regulated Genes	GO_TELOMERE_ORGANIZATION	12	-0.92218256	0.5489865
Down Regulated Genes	GO_TRANSLATIONAL_ELONGATION	2	-0.9216313	0.60600376
Down Regulated Genes	GO_CELLULAR_RESPONSE_TO_HYPEROXIA	1	-0.9208453	0.6199187
Down Regulated Genes	GO_REGULATION_OF_HISTONE_H3_K9_METHYLATION	1	-0.92051345	0.61270493
Down Regulated Genes	GO_NEGATIVE_REGULATION_OF_HISTONE_H3_K9_METHYLATION	1	-0.9203362	0.6082677
Down Regulated Genes	GO_NEGATIVE_REGULATION_OF_TRANSCRIPTION_BY_RNA_POLYMERASE_II	20	-0.91961026	0.5467128
Down Regulated Genes	GO_PROTEIN_MODIFICATION_BY_SMALL_PROTEIN_CONJUGATION_OR_REMOVAL	24	-0.919046	0.5786885
Down Regulated Genes	GO_MUSCLE_CELL_MIGRATION	1	-0.9145573	0.65940595
Down Regulated Genes	GO_POSITIVE_REGULATION_OF_PROTEIN_MODIFICATION_BY_SMALL_PROTEIN_CONJUGATION_	4	-0.91451484	0.5701439
Down Regulated Genes	GO_PEPTIDYL_LYSINE_MODIFICATION	9	-0.91363823	0.56
Down Regulated Genes	GO_DNA_METHYLATION_ON_CYTOSINE	1	-0.91348416	0.62239087
Down Regulated Genes	GO_REGULATION_OF_DNA_DAMAGE_CHECKPOINT	2	-0.9133122	0.5945946
Down Regulated Genes	GO_POSITIVE_REGULATION_OF_DNA_METABOLIC_PROCESS	8	-0.912625	0.5499154
Down Regulated Genes	GO_REGULATION_OF_RESPONSE_TO_OXIDATIVE_STRESS	2	-0.9117285	0.5954198
Down Regulated Genes	GO_DNA_CATABOLIC_PROCESS_ENDONUCLEOLYTIC	3	-0.9116904	0.60146254
Down Regulated Genes	GO_POSITIVE_REGULATION_OF_HISTONE_H3_K4_METHYLATION	1	-0.91137064	0.63773584
Down Regulated Genes	GO_REGULATION_OF_DNA_BINDING	3	-0.9109413	0.61205274
Down Regulated Genes	GO_NEGATIVE_REGULATION_OF_ACTIN_FILAMENT_POLYMERIZATION	2	-0.91048545	0.6003717
Down Regulated Genes	GO_RESPONSE_TO_DEXAMETHASONE	3	-0.91035944	0.59325045
Down Regulated Genes	GO_SULFUR_COMPOUND_BIOSYNTHETIC_PROCESS	5	-0.91018265	0.573975
Down Regulated Genes	GO_RESPONSE_TO_ACTIVITY	4	-0.9101414	0.5595238
Down Regulated Genes	GO_EXTRINSIC_APOPTOTIC_SIGNALING_PATHWAY	3	-0.90991575	0.5896488
Down Regulated Genes	GO_MYOBLAST_MIGRATION	1	-0.90990907	0.6422594
Down Regulated Genes	GO_RESPONSE_TO_CAFFEINE	1	-0.90946203	0.6659878
Down Regulated Genes	GO_REGULATION_OF_ESTABLISHMENT_OR_MAINTENANCE_OF_CELL_POLARITY	2	-0.9087204	0.6028881
Down Regulated Genes	GO_POST_EMBRYONIC_CAMERA_TYPE_EYE_DEVELOPMENT	1	-0.9084708	0.6244898
Down Regulated Genes	GO_MALE_MEIOTIC_NUCLEAR_DIVISION	1	-0.90811473	0.6340509
Down Regulated Genes	GO_EXECUTION_PHASE_OF_APOPTOSIS	3	-0.9077832	0.58201057
Down Regulated Genes	GO_GLAND_DEVELOPMENT	13	-0.9074933	0.5495208
Down Regulated Genes	GO_OSTEOBLAST_PROLIFERATION	1	-0.90720063	0.6252546
Down Regulated Genes	GO_COMMITMENT_OF_NEURONAL_CELL_TO_SPECIFIC_NEURON_TYPE_IN_FOREBRAIN	1	-0.9070459	0.6226804
Down Regulated Genes	GO_NEGATIVE_REGULATION_OF_RNA_BIOSYNTHETIC_PROCESS	25	-0.9067655	0.5659164
Down Regulated Genes	GO_REGULATION_OF_IMMUNOGLOBULIN_PRODUCTION	3	-0.9067183	0.5968858
Down Regulated Genes	GO_POSITIVE_REGULATION_OF_IMMUNOGLOBULIN_PRODUCTION	3	-0.9066562	0.5753676
Down Regulated Genes	GO_NEURON_FATE_COMMITMENT	1	-0.90658206	0.6226804
Down Regulated Genes	GO_STRIATUM_DEVELOPMENT	1	-0.90623206	0.62655604
Down Regulated Genes	GO_CYTOPLASMIC_MICROTUBULE_ORGANIZATION	1	-0.90437686	0.64705884
Down Regulated Genes	GO_DNA_DAMAGE_RESPONSE_SIGNAL_TRANSDUCTION_RESULTING_IN_TRANSCRIPTION	2	-0.90418106	0.6189591
Down Regulated Genes	GO_POST_EMBRYONIC_ANIMAL_ORGAN_DEVELOPMENT	1	-0.9038538	0.65983605
Down Regulated Genes	GO_MICROTUBULE_POLYMERIZATION_OR_DEPOLYMERIZATION	6	-0.90351355	0.5762712
Down Regulated Genes	GO_CELL_DEATH_IN_RESPONSE_TO_OXIDATIVE_STRESS	2	-0.90312797	0.64311594
Down Regulated Genes	GO_CIRCADIAN_REGULATION_OF_GENE_EXPRESSION	2	-0.90213627	0.6095764
Down Regulated Genes	GO_ANATOMICAL_STRUCTURE_HOMEOSTASIS	15	-0.9014052	0.5618729
Down Regulated Genes	GO_PEPTIDE_BIOSYNTHETIC_PROCESS	9	-0.90096724	0.5844371
Down Regulated Genes	GO_NEGATIVE_REGULATION_OF_WNT_SIGNALING_PATHWAY	2	-0.9002644	0.6433824
Down Regulated Genes	GO_FOREBRAIN_NEURON_FATE_COMMITMENT	1	-0.8997085	0.64895636
Down Regulated Genes	GO_POST_EMBRYONIC_DEVELOPMENT	1	-0.89805335	0.6502836
Down Regulated Genes	GO_REGULATION_OF_KERATINOCYTE_PROLIFERATION	1	-0.89779484	0.65286
Down Regulated Genes	GO_RESPONSE_TO_INSULIN	3	-0.89698756	0.5993031
Down Regulated Genes	GO_CELL_PART_MORPHOGENESIS	10	-0.89689505	0.5873288
Down Regulated Genes	GO_REGULATION_OF_MORPHOGENESIS_OF_AN_EPITHELIUM	3	-0.8957906	0.6267857
Down Regulated Genes	GO_REGULATION_OF_CARBOHYDRATE_CATABOLIC_PROCESS	2	-0.8948898	0.6105675

Down Regulated Genes	GO_CELLULAR_NITROGEN_COMPOUND_CATABOLIC_PROCESS	12	-0.89473844	0.57621443
Down Regulated Genes	GO_DEVELOPMENTAL_MATURATION	4	-0.8938504	0.58523726
Down Regulated Genes	GO_MICROTUBULE_BASED_MOVEMENT	7	-0.89358336	0.59090906
Down Regulated Genes	GO_MICROTUBULE_SEVERING	1	-0.89251006	0.65873015
Down Regulated Genes	GO_SOMATIC_DIVERSIFICATION_OF_T_CELL_RECEPTOR_GENES	1	-0.89197457	0.6539197
Down Regulated Genes	GO_HEMATOPOIETIC_STEM_CELL_MIGRATION	1	-0.89160055	0.67404425
Down Regulated Genes	GO_EMBRYONIC_ORGAN_DEVELOPMENT	4	-0.88980776	0.5816876
Down Regulated Genes	GO_REGULATION_OF_CARBOHYDRATE_METABOLIC_PROCESS	2	-0.88935524	0.6423358
Down Regulated Genes	GO_APOPTOTIC_DNA_FRAGMENTATION	3	-0.88904846	0.61454546
Down Regulated Genes	GO_POSITIVE_REGULATION_OF_CHROMOSOME_ORGANIZATION	7	-0.88888466	0.5877193
Down Regulated Genes	GO_PROTEIN_MODIFICATION_BY_SMALL_PROTEIN_REMOVAL	6	-0.8887834	0.5714286
Down Regulated Genes	GO_THYMUS_DEVELOPMENT	1	-0.888643	0.674
Down Regulated Genes	GO_CELLULAR_GLUCAN_METABOLIC_PROCESS	2	-0.88804734	0.62639403
Down Regulated Genes	GO_CELLULAR_COMPONENT_DISASSEMBLY_INVOLVED_IN_EXECUTION_PHASE_OF_APOPTOSIS	3	-0.8880396	0.6443203
Down Regulated Genes	GO_ORGANIC_CYCLIC_COMPOUND_CATABOLIC_PROCESS	12	-0.88753045	0.6130653
Down Regulated Genes	GO_HISTONE_MONOUBIQUITINATION	1	-0.8873532	0.6500994
Down Regulated Genes	GO_POLYSACCHARIDE_METABOLIC_PROCESS	2	-0.88677055	0.67315173
Down Regulated Genes	GO_MISMATCH_REPAIR	3	-0.8864611	0.6142322
Down Regulated Genes	GO_PIGMENT_METABOLIC_PROCESS	2	-0.88556314	0.6520147
Down Regulated Genes	GO_ERROR_FREE_TRANSLESION_SYNTHESIS	3	-0.88538677	0.63468635
Down Regulated Genes	GO_PHOTOPERIODISM	2	-0.88414055	0.6463878
Down Regulated Genes	GO_HISTONE_H2A_UBIQUITINATION	1	-0.8833243	0.6755725
Down Regulated Genes	GO_SUBPALLIUM_DEVELOPMENT	1	-0.88302195	0.6751968
Down Regulated Genes	GO_METHYLATION	8	-0.8828738	0.5982906
Down Regulated Genes	GO_CARBOHYDRATE_DERIVATIVE_METABOLIC_PROCESS	13	-0.8822779	0.584874
Down Regulated Genes	GO_PROTEIN_MONOUBIQUITINATION	1	-0.88128346	0.6653226
Down Regulated Genes	GO_DNA_STRAND_ELONGATION_INVOLVED_IN_DNA_REPLICATION	3	-0.8801268	0.6203704
Down Regulated Genes	GO_ENERGY_RESERVE_METABOLIC_PROCESS	2	-0.88009846	0.6418219
Down Regulated Genes	GO_REGULATION_OF_APOPTOTIC_SIGNALING_PATHWAY	9	-0.87833875	0.5966667
Down Regulated Genes	GO_HISTONE_H2A_MONOUBIQUITINATION	1	-0.8780089	0.6811881
Down Regulated Genes	GO_NEURON_MIGRATION	3	-0.8773053	0.6474954
Down Regulated Genes	GO_REGULATION_OF_DOUBLE_STRAND_BREAK_REPAIR_VIA_NONHOMOLOGOUS_END_JOININ	2	-0.8771781	0.6715867
Down Regulated Genes	GO_POSITIVE_REGULATION_OF_B_CELL_ACTIVATION	3	-0.8761796	0.6344086
Down Regulated Genes	GO_VIRAL_GENOME_REPLICATION	1	-0.87585545	0.6638478
Down Regulated Genes	GO_ORGANISM_EMERGENCE_FROM_PROTECTIVE_STRUCTURE	1	-0.8758505	0.6507276
Down Regulated Genes	GO_REGULATION_OF_NERVOUS_SYSTEM_DEVELOPMENT	20	-0.87531054	0.6150235
Down Regulated Genes	GO_NEGATIVE_REGULATION_OF_CELL_AGING	2	-0.8751765	0.6768953
Down Regulated Genes	GO_CELLULAR_KETONE_METABOLIC_PROCESS	3	-0.87476015	0.6579861
Down Regulated Genes	GO_RESPONSE_TO_OXIDATIVE_STRESS	14	-0.8745981	0.6
Down Regulated Genes	GO_RESPONSE_TO_CADMIUM_ION	4	-0.87448573	0.64298403
Down Regulated Genes	GO_BLASTOCYST_DEVELOPMENT	1	-0.8733793	0.7007874
Down Regulated Genes	GO_NEURON_DIFFERENTIATION	22	-0.8722931	0.63665086
Down Regulated Genes	GO_ENTRAINMENT_OF_CIRCADIAN_CLOCK	2	-0.8705381	0.64074075
Down Regulated Genes	GO_REGULATION_OF_GLIAL_CELL_DIFFERENTIATION	4	-0.8705264	0.62846583
Down Regulated Genes	GO_PIGMENT_BIOSYNTHETIC_PROCESS	2	-0.8694752	0.69104207
Down Regulated Genes	GO_DEOXYRIBONUCLEOSIDE_MONOPHOSPHATE_METABOLIC_PROCESS	2	-0.8692866	0.68515205
Down Regulated Genes	GO_DEOXYRIBONUCLEOSIDE_MONOPHOSPHATE_BIOSYNTHETIC_PROCESS	2	-0.86834306	0.67790264
Down Regulated Genes	GO_PYRIDINE_CONTAINING_COMPOUND_METABOLIC_PROCESS	1	-0.868098	0.68093383
Down Regulated Genes	GO_MRNA_PROCESSING	4	-0.8679839	0.6336283
Down Regulated Genes	GO_HISTONE_UBIQUITINATION	1	-0.8659965	0.69574034
Down Regulated Genes	GO_NUCLEOBASE_CATABOLIC_PROCESS	1	-0.86579204	0.693712
Down Regulated Genes	GO_PYRIMIDINE_NUCLEOSIDE_METABOLIC_PROCESS	2	-0.86545277	0.68101764
Down Regulated Genes	GO_REGULATION_OF_MRNA_PROCESSING	2	-0.8651258	0.68439716
Down Regulated Genes	GO_REGULATION_OF_CELL_AGING	2	-0.8649311	0.66731143
Down Regulated Genes	GO_REGULATION_OF_MRNA_METABOLIC_PROCESS	4	-0.86418855	0.6189624
Down Regulated Genes	GO_REGULATION_OF_RNA_SPLICING	2	-0.8641805	0.69590646
Down Regulated Genes	GO_HISTONE_H2A_K119_MONOUBIQUITINATION	1	-0.8631065	0.699187
Down Regulated Genes	GO_REGULATION_OF_ANIMAL_ORGAN_MORPHOGENESIS	3	-0.8622342	0.6793893
Down Regulated Genes	GO_TETRAHYDROFOLATE_INTERCONVERSION	2	-0.86217487	0.6825095
Down Regulated Genes	GO_PYRIMIDINE_DEOXYRIBONUCLEOSIDE_MONOPHOSPHATE_BIOSYNTHETIC_PROCESS	2	-0.86121553	0.6826923
Down Regulated Genes	GO_POSITIVE_REGULATION_OF_TRANSCRIPTION_BY_RNA_POLYMERASE_II	19	-0.86093426	0.6312292
Down Regulated Genes	GO_GAMETE_GENERATION	13	-0.8605389	0.62521297
Down Regulated Genes	GO_FOLIC_ACID_METABOLIC_PROCESS	2	-0.8605278	0.6864564
Down Regulated Genes	GO_FLOOR_PLATE_DEVELOPMENT	1	-0.8591819	0.6764706
Down Regulated Genes	GO_RAS_PROTEIN_SIGNAL_TRANSDUCTION	7	-0.8577381	0.625
Down Regulated Genes	GO_NEGATIVE_REGULATION_OF_SUPRAMOLECULAR_FIBER_ORGANIZATION	5	-0.8572581	0.6308725
Down Regulated Genes	GO_TELOMERE_MAINTENANCE_VIA_SEMI_CONSERVATIVE_REPLICATION	6	-0.857238	0.6122807
Down Regulated Genes	GO_REGULATION_OF_PROTEIN_EXPORT_FROM_NUCLEUS	2	-0.85586935	0.6972477
Down Regulated Genes	GO_VITAMIN_B6_METABOLIC_PROCESS	1	-0.855826	0.70083684
Down Regulated Genes	GO_DRUG_METABOLIC_PROCESS	1	-0.8557697	0.71283096

Down Regulated Genes	GO_MACROMOLECULE_CATABOLIC_PROCESS	23	-0.8545659	0.63242376
Down Regulated Genes	GO_PYRIMIDINE_NUCLEOSIDE_MONOPHOSPHATE_METABOLIC_PROCESS	2	-0.85387635	0.686907
Down Regulated Genes	GO_PLACENTA_DEVELOPMENT	4	-0.8538582	0.65924656
Down Regulated Genes	GO_PYRIMIDINE_DEOXYRIBONUCLEOSIDE_MONOPHOSPHATE_METABOLIC_PROCESS	2	-0.85228425	0.6969697
Down Regulated Genes	GO_DNA_STRAND_ELONGATION	3	-0.8506616	0.66415095
Down Regulated Genes	GO_CELLULAR_RESPONSE_TO_PEPTIDE	4	-0.8491176	0.6535581
Down Regulated Genes	GO_MITOCHONDRIAL_TRANSMEMBRANE_TRANSPORT	1	-0.84860885	0.7233202
Down Regulated Genes	GO_POSITIVE_REGULATION_OF_TRANSFERASE_ACTIVITY	19	-0.8480365	0.65384614
Down Regulated Genes	GO_DICARBOXYLIC_ACID_METABOLIC_PROCESS	2	-0.8457332	0.7306968
Down Regulated Genes	GO_NOTOCHORD_DEVELOPMENT	1	-0.84530056	0.7198364
Down Regulated Genes	GO_RESPONSE_TO_HYDROGEN_PEROXIDE	7	-0.84525126	0.6452174
Down Regulated Genes	GO_ALTERNATIVE_MRNA_SPLICING_VIA_SPLICEOSOME	2	-0.84517413	0.7037037
Down Regulated Genes	GO_POSITIVE_REGULATION_OF_PROTEIN_EXPORT_FROM_NUCLEUS	2	-0.84390604	0.71009177
Down Regulated Genes	GO_T_HELPER_1_CELL_DIFFERENTIATION	1	-0.8437193	0.70916337
Down Regulated Genes	GO_TELENCEPHALON_DEVELOPMENT	8	-0.8430316	0.65587735
Down Regulated Genes	GO_POSITIVE_REGULATION_OF_INTERLEUKIN_12_PRODUCTION	1	-0.8428522	0.7392197
Down Regulated Genes	GO_REGULATION_OF_MRNA_SPLICING_VIA_SPLICEOSOME	2	-0.8425533	0.72904485
Down Regulated Genes	GO_PYRIMIDINE_CONTAINING_COMPOUND_CATABOLIC_PROCESS	2	-0.84204626	0.7283237
Down Regulated Genes	GO_NEGATIVE_REGULATION_OF_BLOOD_VESSEL_ENDOTHELIAL_CELL_MIGRATION	1	-0.84193987	0.724
Down Regulated Genes	GO_EMBRYONIC_AXIS_SPECIFICATION	1	-0.84135	0.75
Down Regulated Genes	GO_REGULATION_OF_CENTRIOLE_REPLICATION	1	-0.8401949	0.7450593
Down Regulated Genes	GO_T_CELL_MEDIATED_IMMUNE_RESPONSE_TO_TUMOR_CELL	1	-0.8392052	0.7418033
Down Regulated Genes	GO_POSITIVE_REGULATION_OF_TOLL_LIKE_RECEPTOR_SIGNALING_PATHWAY	1	-0.8382397	0.7217899
Down Regulated Genes	GO_DENDRITIC_CELL_MIGRATION	1	-0.8379941	0.72141373
Down Regulated Genes	GO_REGULATION_OF_DENDRITIC_CELL_DIFFERENTIATION	1	-0.8363928	0.72839504
Down Regulated Genes	GO_POSITIVE_REGULATION_OF_PROTEIN_SERINE_THREONINE_KINASE_ACTIVITY	11	-0.83625114	0.66550523
Down Regulated Genes	GO_NEGATIVE_REGULATION_OF_PHAGOCYTOSIS	3	-0.8361402	0.68926555
Down Regulated Genes	GO_INTERLEUKIN_12_PRODUCTION	1	-0.8353289	0.74327123
Down Regulated Genes	GO_RESPONSE_TO_TUMOR_CELL	1	-0.8353107	0.7240664
Down Regulated Genes	GO_NEURON_DEVELOPMENT	17	-0.83526605	0.64403975
Down Regulated Genes	GO_REGULATION_OF_DNA_LIGATION	1	-0.83510345	0.7590361
Down Regulated Genes	GO_PROTEIN_LIPOYLATION	1	-0.83507687	0.73394495
Down Regulated Genes	GO_REGULATION_OF_MONOCYTE_CHEMOTAXIS	1	-0.83484775	0.709369
Down Regulated Genes	GO_REGULATION_OF_DNA_DEPENDENT_DNA_REPLICATION_INITIATION	2	-0.8343437	0.72313297
Down Regulated Genes	GO_IMMUNE_RESPONSE_TO_TUMOR_CELL	1	-0.8332449	0.7515528
Down Regulated Genes	GO_POSITIVE_REGULATION_OF_TRANSMEMBRANE_RECEPTOR_PROTEIN_SERINE_THREONINE_	2	-0.83317167	0.7552182
Down Regulated Genes	GO_REGULATION_OF_APOPTOTIC_CELL_CLEARANCE	1	-0.8331364	0.7520492
Down Regulated Genes	GO_TOLL_LIKE_RECEPTOR_9_SIGNALING_PATHWAY	1	-0.83244216	0.743487
Down Regulated Genes	GO_DENDRITIC_CELL_CHEMOTAXIS	1	-0.8317987	0.7389558
Down Regulated Genes	GO_FOREBRAIN_DEVELOPMENT	9	-0.8311921	0.6695652
Down Regulated Genes	GO_ACTIVATION_OF_INNATE_IMMUNE_RESPONSE	1	-0.831121	0.7714286
Down Regulated Genes	GO_LIPID_DROPLET_ORGANIZATION	1	-0.8310076	0.7628866
Down Regulated Genes	GO_VASCULAR_ENDOTHELIAL_CELL_PROLIFERATION	1	-0.8307904	0.74168295
Down Regulated Genes	GO_REGULATION_OF_CELL_DEVELOPMENT	21	-0.8304923	0.6644068
Down Regulated Genes	GO_REGULATION_OF_RESPONSE_TO_TUMOR_CELL	1	-0.8302606	0.7592233
Down Regulated Genes	GO_CENTRIOLE_ASSEMBLY	2	-0.83019686	0.72015655
Down Regulated Genes	GO_POSITIVE_REGULATION_OF_DNA_LIGATION	1	-0.8298425	0.7632094
Down Regulated Genes	GO_POSITIVE_REGULATION_OF_MONOCYTE_CHEMOTAXIS	1	-0.8293584	0.749004
Down Regulated Genes	GO_CELLULAR_AMIDE_METABOLIC_PROCESS	16	-0.8289595	0.65378153
Down Regulated Genes	GO_DNA_DOUBLE_STRAND_BREAK_PROCESSING	2	-0.82880265	0.707457
Down Regulated Genes	GO_APOPTOTIC_CELL_CLEARANCE	1	-0.82847315	0.75551105
Down Regulated Genes	GO_CHEMOKINE_C_X_C_MOTIF_LIGAND_2_PRODUCTION	1	-0.8283209	0.75
Down Regulated Genes	GO_NEGATIVE_REGULATION_OF_DNA_TEMPLATED_TRANSCRIPTION_INITIATION	1	-0.8270581	0.74150944
Down Regulated Genes	GO_POSITIVE_REGULATION_OF_VASCULAR_ENDOTHELIAL_CELL_PROLIFERATION	1	-0.82685983	0.7672065
Down Regulated Genes	GO_POSITIVE_REGULATION_OF_MONONUCLEAR_CELL_MIGRATION	1	-0.82603365	0.7684631
Down Regulated Genes	GO_REGULATION_OF_PROTEIN_STABILITY	3	-0.8248316	0.7321101
Down Regulated Genes	GO_REGULATION_OF_ENDODEOXYRIBONUCLEASE_ACTIVITY	1	-0.8242357	0.73619634
Down Regulated Genes	GO_REGULATION_OF_CELL_PROJECTION_ORGANIZATION	9	-0.8230175	0.6639344
Down Regulated Genes	GO_AUTOCRINE_SIGNALING	1	-0.8227157	0.7520161
Down Regulated Genes	GO_REGULATION_OF_PHAGOCYTOSIS	3	-0.8216864	0.7175141
Down Regulated Genes	GO_REGULATION_OF_T_CELL_MEDIATED_IMMUNE_RESPONSE_TO_TUMOR_CELL	1	-0.8214566	0.78483605
Down Regulated Genes	GO_CELL_FATE_COMMITMENT	3	-0.8212594	0.7324955
Down Regulated Genes	GO_NEUTROPHIL_CLEARANCE	1	-0.82044876	0.7703252
Down Regulated Genes	GO_PROTEIN_STABILIZATION	3	-0.8193631	0.714032
Down Regulated Genes	GO_POSITIVE_REGULATION_OF_ACTIVATED_T_CELL_PROLIFERATION	1	-0.81904656	0.7548387
Down Regulated Genes	GO_REGULATION_OF_CELLULAR_AMIDE_METABOLIC_PROCESS	8	-0.8188918	0.66903913
Down Regulated Genes	GO_MALE_GAMETE_GENERATION	11	-0.818823	0.6897747
Down Regulated Genes	GO_CHROMATIN_ORGANIZATION_INVOLVED_IN_REGULATION_OF_TRANSCRIPTION	3	-0.81881315	0.7063197
Down Regulated Genes	GO_SEXUAL_REPRODUCTION	17	-0.8183723	0.7006689
Down Regulated Genes	GO_POSITIVE_REGULATION_OF_CELLULAR_BIOSYNTHETIC_PROCESS	35	-0.8181658	0.69486403

Down Regulated Genes	GO_POSITIVE_REGULATION_OF_PATTERN_RECOGNITION_RECEPTOR_SIGNALING_PATHWAY	1	-0.818112	0.77642274
Down Regulated Genes	GO_POSITIVE_REGULATION_OF_CHEMOKINE_C_X_C_MOTIF_LIGAND_2_PRODUCTION	1	-0.81693864	0.7651992
Down Regulated Genes	GO_POSITIVE_REGULATION_OF_PINOCYTOSIS	1	-0.8162284	0.7581301
Down Regulated Genes	GO_POSITIVE_REGULATION_OF_TRANSMEMBRANE_TRANSPORT	1	-0.8161922	0.7728155
Down Regulated Genes	GO_REGULATION_OF_TOLL_LIKE_RECEPTOR_9_SIGNALING_PATHWAY	1	-0.81613845	0.7654321
Down Regulated Genes	GO_FATTY_ACID_METABOLIC_PROCESS	9	-0.81575745	0.6683761
Down Regulated Genes	GO_RESPONSE_TO_HEPATOCYTE_GROWTH_FACTOR	1	-0.8157531	0.7490119
Down Regulated Genes	GO_POSITIVE_REGULATION_OF_BLOOD_VESSEL_ENDOTHELIAL_CELL_MIGRATION	1	-0.8157523	0.7647059
Down Regulated Genes	GO_NEGATIVE_REGULATION_OF_TRANSCRIPTION_INITIATION_FROM_RNA_POLYMERASE_II_PR	1	-0.81506515	0.78510636
Down Regulated Genes	GO_BONE_REMODELING	2	-0.81494886	0.7196429
Down Regulated Genes	GO_POSITIVE_REGULATION_OF_INTERFERON_BETA_PRODUCTION	4	-0.8149318	0.6987522
Down Regulated Genes	GO_NEGATIVE_REGULATION_OF_IMMUNE_EFFECTOR_PROCESS	1	-0.81476367	0.7799228
Down Regulated Genes	GO_PATHWAY_RESTRICTED_SMAD_PROTEIN_PHOSPHORYLATION	3	-0.81427497	0.7202925
Down Regulated Genes	GO_TEMPERATURE_HOMEOSTASIS	2	-0.8133391	0.7598499
Down Regulated Genes	GO_GLUCOSE_IMPORT	1	-0.813057	0.79141104
Down Regulated Genes	GO_BONE_RESORPTION	2	-0.8130375	0.7607477
Down Regulated Genes	GO_INTERFERON_BETA_PRODUCTION	4	-0.81294286	0.70855147
Down Regulated Genes	GO_POSITIVE_REGULATION_OF_PROTEIN_MODIFICATION_PROCESS	27	-0.81252307	0.69871795
Down Regulated Genes	GO_SPERMATID_DIFFERENTIATION	3	-0.81131685	0.729927
Down Regulated Genes	GO_ORGANONITROGEN_COMPOUND_CATABOLIC_PROCESS	19	-0.8100554	0.7207358
Down Regulated Genes	GO_WATER_HOMEOSTASIS	4	-0.80959386	0.7033898
Down Regulated Genes	GO_NEGATIVE_REGULATION_OF_ARP2_3_COMPLEX_MEDIATED_ACTIN_NUCLEATION	1	-0.8090773	0.78154427
Down Regulated Genes	GO_KERATINOCYTE_MIGRATION	2	-0.8085161	0.7828571
Down Regulated Genes	GO_REGULATION_OF_GLUCOSE_IMPORT	1	-0.80841774	0.7733888
Down Regulated Genes	GO_REGULATION_OF_SECONDARY_METABOLIC_PROCESS	1	-0.8082429	0.7908903
Down Regulated Genes	GO_ACTIN_FILAMENT_SEVERING	1	-0.8080906	0.7807933
Down Regulated Genes	GO_FEEDING_BEHAVIOR	2	-0.80749893	0.7717391
Down Regulated Genes	GO_REGULATION_OF_ARP2_3_COMPLEX_MEDIATED_ACTIN_NUCLEATION	1	-0.8070387	0.78099173
Down Regulated Genes	GO_TOLL_LIKE_RECEPTOR_4_SIGNALING_PATHWAY	1	-0.8068796	0.7819383
Down Regulated Genes	GO_REGULATION_OF_SECONDARY_METABOLITE_BIOSYNTHETIC_PROCESS	1	-0.80649954	0.7746479
Down Regulated Genes	GO_REGULATION_OF_ACTIN_NUCLEATION	1	-0.80623066	0.79752064
Down Regulated Genes	GO_REGULATION_OF_PINOCYTOSIS	1	-0.8059156	0.81349206
Down Regulated Genes	GO_SIGNAL_TRANSDUCTION_IN_ABSENCE_OF_LIGAND	1	-0.80496705	0.80582523
Down Regulated Genes	GO_PINOCYTOSIS	1	-0.8042553	0.7920998
Down Regulated Genes	GO_POSITIVE_REGULATION_OF_SECONDARY_METABOLITE_BIOSYNTHETIC_PROCESS	1	-0.80357546	0.78543305
Down Regulated Genes	GO_DEFINITIVE_HEMOPOIESIS	1	-0.80331796	0.7900553
Down Regulated Genes	GO_POSITIVE_REGULATION_OF_ENDOCYTOSIS	1	-0.8023102	0.7629482
Down Regulated Genes	GO_REGULATION_OF_FIBROBLAST_MIGRATION	1	-0.80204886	0.8046875
Down Regulated Genes	GO_MACROPINOCYTOSIS	1	-0.80180603	0.7831094
Down Regulated Genes	GO_FIBROBLAST_MIGRATION	1	-0.8015951	0.7950664
Down Regulated Genes	GO_POSITIVE_REGULATION_OF_GLUCOSE_TRANSMEMBRANE_TRANSPORT	1	-0.8013721	0.78755367
Down Regulated Genes	GO_3_UTR_MEDIATED_MRNA_DESTABILIZATION	1	-0.8011739	0.79626167
Down Regulated Genes	GO_REGULATION_OF_ENDOCYTOSIS	1	-0.80076694	0.798419
Down Regulated Genes	GO_REGULATION_OF_TOLL_LIKE_RECEPTOR_4_SIGNALING_PATHWAY	1	-0.80048424	0.7777778
Down Regulated Genes	GO_MYELOID_CELL_HOMEOSTASIS	4	-0.80036956	0.7513812
Down Regulated Genes	GO_REGULATION_OF_NUCLEAR_TRANSCRIBED_MRNA_CATABOLIC_PROCESS_DEADENYLATION_	1	-0.79938394	0.8003992
Down Regulated Genes	GO_ARP2_3_COMPLEX_MEDIATED_ACTIN_NUCLEATION	1	-0.79932934	0.7826962
Down Regulated Genes	GO_SECONDARY_METABOLITE_BIOSYNTHETIC_PROCESS	1	-0.7991146	0.77843136
Down Regulated Genes	GO_MORPHOGENESIS_OF_AN_EPITHELIUM	11	-0.79890966	0.6881533
Down Regulated Genes	GO_PHENOL_CONTAINING_COMPOUND_METABOLIC_PROCESS	1	-0.7989016	0.7637795
Down Regulated Genes	GO_DNA_UNWINDING_INVOLVED_IN_DNA_REPLICATION	3	-0.79887575	0.74676526
Down Regulated Genes	GO_ANIMAL_ORGAN_REGENERATION	7	-0.79848176	0.6861702
Down Regulated Genes	GO_PROTEIN_ACYLATION	2	-0.7984538	0.7690972
Down Regulated Genes	GO_REGULATION_OF_FC_RECEPTOR_MEDIATED_STIMULATORY_SIGNALING_PATHWAY	1	-0.79776746	0.80340266
Down Regulated Genes	GO_REGULATION_OF_B_CELL_DIFFERENTIATION	1	-0.7974871	0.8019231
Down Regulated Genes	GO_CARBOHYDRATE_TRANSMEMBRANE_TRANSPORT	1	-0.797329	0.7968442
Down Regulated Genes	GO_INSULIN_RECEPTOR_SIGNALING_PATHWAY	1	-0.79694515	0.8121442
Down Regulated Genes	GO_POSITIVE_REGULATION_OF_TYPE_I_INTERFERON_PRODUCTION	4	-0.7966101	0.73134327
Down Regulated Genes	GO_POSITIVE_REGULATION_OF_SISTER_CHROMATID_COHESION	2	-0.7957877	0.7763401
Down Regulated Genes	GO_NEGATIVE_REGULATION_OF_ACTIN_NUCLEATION	1	-0.79507524	0.80078894
Down Regulated Genes	GO_REGULATION_OF_FC_GAMMA_RECEPTOR_SIGNALING_PATHWAY_INVOLVED_IN_PHAGOCY	1	-0.79356194	0.8254902
Down Regulated Genes	GO_SOMATIC_STEM_CELL_POPULATION_MAINTENANCE	1	-0.7933593	0.8166023
Down Regulated Genes	GO_SULFUR_COMPOUND_METABOLIC_PROCESS	9	-0.7932586	0.7198642
Down Regulated Genes	GO_NEGATIVE_REGULATION_OF_STEM_CELL_DIFFERENTIATION	1	-0.79279625	0.79752064
Down Regulated Genes	GO_GERM_CELL_DEVELOPMENT	3	-0.79272395	0.74909747
Down Regulated Genes	GO_PHENOL_CONTAINING_COMPOUND_BIOSYNTHETIC_PROCESS	1	-0.7921654	0.81346154
Down Regulated Genes	GO_RESPONSE_TO_GRANULOCYTE_MACROPHAGE_COLONY_STIMULATING_FACTOR	1	-0.79095185	0.802
Down Regulated Genes	GO_ACTIN_FILAMENT_NETWORK_FORMATION	2	-0.79090077	0.77962965
Down Regulated Genes	GO_REGULATION_OF_GLUCOSE_TRANSMEMBRANE_TRANSPORT	1	-0.7905441	0.8043912
Down Regulated Genes	GO_STEM_CELL_DIVISION	1	-0.79050666	0.8081633

Down Regulated Genes	GO_ACTIN_NUCLEATION	1	-0.7896649	0.805726
Down Regulated Genes	GO_FC_RECEPTOR_MEDIATED_STIMULATORY_SIGNALING_PATHWAY	1	-0.7891863	0.81854045
Down Regulated Genes	GO_SOMATIC_STEM_CELL_DIVISION	1	-0.78865	0.82077396
Down Regulated Genes	GO_MULTICELLULAR_ORGANISM_REPRODUCTION	18	-0.78682667	0.73311365
Down Regulated Genes	GO_POSTTRANSCRIPTIONAL_REGULATION_OF_GENE_EXPRESSION	11	-0.785489	0.70436186
Down Regulated Genes	GO_MITOTIC_SISTER_CHROMATID_COHESION	2	-0.7853018	0.78409094
Down Regulated Genes	GO_REGULATION_OF_INTRINSIC_APOPTOTIC_SIGNALING_PATHWAY	3	-0.7851865	0.75785583
Down Regulated Genes	GO_CELLULAR_DEFENSE_RESPONSE	1	-0.782255	0.8154762
Down Regulated Genes	GO_REPRODUCTIVE_SYSTEM_DEVELOPMENT	11	-0.78134954	0.7283737
Down Regulated Genes	GO_NEGATIVE_REGULATION_OF_PROTEIN_METABOLIC_PROCESS	19	-0.78000116	0.75
Down Regulated Genes	GO_NEGATIVE_REGULATION_OF_NEURON_DEATH	2	-0.77928233	0.7917415
Down Regulated Genes	GO_HISTONE_H3_K9_METHYLATION	2	-0.77823687	0.7906542
Down Regulated Genes	GO_POSITIVE_REGULATION_OF_SMOOTHENED_SIGNALING_PATHWAY	1	-0.77727437	0.78740156
Down Regulated Genes	GO_B_CELL_ACTIVATION_INVOLVED_IN_IMMUNE_RESPONSE	4	-0.7764411	0.75308645
Down Regulated Genes	GO_REGULATION_OF_PROTEIN_BINDING	3	-0.7756545	0.79787236
Down Regulated Genes	GO_HISTONE_H3_K9_MODIFICATION	2	-0.7743236	0.798893
Down Regulated Genes	GO_NEGATIVE_REGULATION_OF_MICROTUBULE_POLYMERIZATION	2	-0.77425057	0.79515827
Down Regulated Genes	GO_RESPONSE_TO_COLD	3	-0.7741346	0.7858473
Down Regulated Genes	GO_POSITIVE_REGULATION_OF_PRODUCTION_OF_MOLECULAR_MEDIATOR_OF_IMMUNE_RESI	4	-0.7731212	0.7837838
Down Regulated Genes	GO_PEPTIDYL_TYROSINE_DEPHOSPHORYLATION	2	-0.7728432	0.7967626
Down Regulated Genes	GO_PROTEIN_ACETYLATION	2	-0.7722571	0.8168498
Down Regulated Genes	GO_NEGATIVE_REGULATION_OF_NEURON_APOPTOTIC_PROCESS	2	-0.7673147	0.81602913
Down Regulated Genes	GO_DEVELOPMENT_OF_PRIMARY_SEXUAL_CHARACTERISTICS	5	-0.7669468	0.76021314
Down Regulated Genes	GO_REGULATION_OF_DNA_METHYLATION	1	-0.7659556	0.85582256
Down Regulated Genes	GO_SEX_DIFFERENTIATION	5	-0.7657487	0.7749141
Down Regulated Genes	GO_GLYCOSYL_COMPOUND_BIOSYNTHETIC_PROCESS	1	-0.76482594	0.8425197
Down Regulated Genes	GO_PYRIMIDINE_NUCLEOSIDE_BIOSYNTHETIC_PROCESS	1	-0.7641751	0.84931505
Down Regulated Genes	GO_ACTIN_POLYMERIZATION_OR_DEPOLYMERIZATION	4	-0.7637623	0.7690941
Down Regulated Genes	GO_REGULATION_OF_ACTIN_FILAMENT_LENGTH	4	-0.7624173	0.7785844
Down Regulated Genes	GO_NUCLEOTIDE_EXCISION_REPAIR_DNA_GAP_FILLING	2	-0.7611397	0.81474483
Down Regulated Genes	GO_TUBE_MORPHOGENESIS	13	-0.760904	0.7441472
Down Regulated Genes	GO_PYRIMIDINE_DEOXYRIBONUCLEOSIDE_METABOLIC_PROCESS	1	-0.76040155	0.868472
Down Regulated Genes	GO_REGULATION_OF_B_CELL_ACTIVATION	4	-0.7594156	0.7705224
Down Regulated Genes	GO_NUCLEOSIDE_MONOPHOSPHATE_PHOSPHORYLATION	1	-0.7592111	0.84765625
Down Regulated Genes	GO_NEGATIVE_REGULATION_OF_MICROTUBULE_POLYMERIZATION_OR_DEPOLYMERIZATION	2	-0.7589143	0.8293135
Down Regulated Genes	GO_REGULATION_OF_PRODUCTION_OF_MOLECULAR_MEDIATOR_OF_IMMUNE_RESPONSE	4	-0.7567782	0.77614677
Down Regulated Genes	GO_PROSTANOID_METABOLIC_PROCESS	2	-0.75639856	0.81040895
Down Regulated Genes	GO_NUCLEOSIDE_DIPHOSPHATE_BIOSYNTHETIC_PROCESS	1	-0.7561918	0.8667992
Down Regulated Genes	GO_GLYCOSPHINGOLIPID_BIOSYNTHETIC_PROCESS	1	-0.7546752	0.860735
Down Regulated Genes	GO_GLYCOSYLCERAMIDE_BIOSYNTHETIC_PROCESS	1	-0.75421816	0.84817815
Down Regulated Genes	GO_TRANSCRIPTION_COUPLED_NUCLEOTIDE_EXCISION_REPAIR	2	-0.7536207	0.8457143
Down Regulated Genes	GO_REACTIVE_OXYGEN_SPECIES_METABOLIC_PROCESS	4	-0.75313044	0.7855856
Down Regulated Genes	GO_HORMONE_MEDIATED_SIGNALING_PATHWAY	5	-0.7528423	0.7848537
Down Regulated Genes	GO_MRNA_METABOLIC_PROCESS	6	-0.7528193	0.7842105
Down Regulated Genes	GO_DEOXYRIBONUCLEOSIDE_DIPHOSPHATE_METABOLIC_PROCESS	1	-0.7522463	0.8602362
Down Regulated Genes	GO_LEARNING	3	-0.7517216	0.7894737
Down Regulated Genes	GO_DEOXYRIBONUCLEOSIDE_METABOLIC_PROCESS	1	-0.75161475	0.8757515
Down Regulated Genes	GO_RNA_PROCESSING	7	-0.751547	0.77374786
Down Regulated Genes	GO_DNA_DAMAGE_RESPONSE_DETECTION_OF_DNA_DAMAGE	2	-0.751482	0.82129276
Down Regulated Genes	GO_NUCLEOTIDE_EXCISION_REPAIR_DNA_INCISION	2	-0.75112236	0.83778626
Down Regulated Genes	GO_REGULATION_OF_REACTIVE_OXYGEN_SPECIES_METABOLIC_PROCESS	4	-0.7508789	0.77110696
Down Regulated Genes	GO_VESICLE_BUDDING_FROM_MEMBRANE	1	-0.7507838	0.8614458
Down Regulated Genes	GO_ORGAN_GROWTH	6	-0.7500016	0.77983195
Down Regulated Genes	GO_REGULATION_OF_CELL_PROJECTION_ASSEMBLY	4	-0.74918544	0.7787611
Down Regulated Genes	GO_T_CELL_MEDIATED_CYTOTOXICITY	1	-0.74907184	0.8665377
Down Regulated Genes	GO_COPII_COATED_VESICLE_BUDDING	1	-0.74892986	0.8722555
Down Regulated Genes	GO_REGULATION_OF_DNA_HELICASE_ACTIVITY	1	-0.7480877	0.8670757
Down Regulated Genes	GO_EAR_DEVELOPMENT	1	-0.7478922	0.88846153
Down Regulated Genes	GO_GOLGI_VESICLE_BUDDING	1	-0.74646354	0.86470586
Down Regulated Genes	GO_GALACTOLIPID_BIOSYNTHETIC_PROCESS	1	-0.7464139	0.8747346
Down Regulated Genes	GO_GLYCOLIPID_BIOSYNTHETIC_PROCESS	1	-0.7461199	0.86585367
Down Regulated Genes	GO_VESICLE_TARGETING_TO_FROM_OR_WITHIN_GOLGI	1	-0.7460092	0.865019
Down Regulated Genes	GO_GLUCOSYLCERAMIDE_METABOLIC_PROCESS	1	-0.7458606	0.8949495
Down Regulated Genes	GO_REGULATION_OF_DNA_DUPLEX_UNWINDING	1	-0.74452245	0.8648649
Down Regulated Genes	GO_MEMBRANE_RAFT_ORGANIZATION	1	-0.743883	0.88709676
Down Regulated Genes	GO_SCHWANN_CELL_DEVELOPMENT	1	-0.7437094	0.8687259
Down Regulated Genes	GO_POSITIVE_REGULATION_OF_ENDOTHELIAL_CELL_MIGRATION	2	-0.74366915	0.83460075
Down Regulated Genes	GO_PERIPHERAL_NERVOUS_SYSTEM_MYELIN_MAINTENANCE	1	-0.7423833	0.8646465
Down Regulated Genes	GO_GLYCOSYLCERAMIDE_METABOLIC_PROCESS	1	-0.74235415	0.8562874
Down Regulated Genes	GO_COCHLEA_DEVELOPMENT	1	-0.7421865	0.8767677

Down Regulated Genes	GO_PLASMA_MEMBRANE_RAFT_ORGANIZATION	1	-0.74179924	0.8802178
Down Regulated Genes	GO_NEGATIVE_REGULATION_OF_ATPASE_ACTIVITY	1	-0.74123126	0.8811881
Down Regulated Genes	GO_POSITIVE_REGULATION_OF_JNK_CASCADE	3	-0.74097395	0.81461674
Down Regulated Genes	GO_REGULATION_OF_ATPASE_ACTIVITY	1	-0.7407154	0.8869732
Down Regulated Genes	GO_SENSORY_PERCEPTION_OF_LIGHT_STIMULUS	2	-0.74071497	0.84220535
Down Regulated Genes	GO_GALACTOLIPID_METABOLIC_PROCESS	1	-0.7406173	0.87401575
Down Regulated Genes	GO_NEGATIVE_REGULATION_OF_HELICASE_ACTIVITY	1	-0.73984843	0.892057
Down Regulated Genes	GO_SEBACEOUS_GLAND_DEVELOPMENT	1	-0.7395136	0.881323
Down Regulated Genes	GO_VESICLE_TARGETING	1	-0.7391054	0.86460346
Down Regulated Genes	GO_POSITIVE_REGULATION_OF_MICROGLIAL_CELL_ACTIVATION	1	-0.73808336	0.8712871
Down Regulated Genes	GO_INTERMEDIATE_FILAMENT_BASED_PROCESS	2	-0.7378119	0.82342005
Down Regulated Genes	GO_NCRNA_EXPORT_FROM_NUCLEUS	1	-0.73718697	0.8962076
Down Regulated Genes	GO_GLYCOSYL_COMPOUND_METABOLIC_PROCESS	4	-0.73704696	0.78205127
Down Regulated Genes	GO_BLOOD_VESSEL_MORPHOGENESIS	8	-0.73668724	0.7766143
Down Regulated Genes	GO_REGULATION_OF_HELICASE_ACTIVITY	1	-0.73536414	0.8898129
Down Regulated Genes	GO_NUCLEOTIDE_EXCISION_REPAIR	2	-0.734556	0.8350877
Down Regulated Genes	GO_TRNA_TRANSPORT	1	-0.73365265	0.8847656
Down Regulated Genes	GO_RNA_EXPORT_FROM_NUCLEUS	1	-0.7331714	0.8899804
Down Regulated Genes	GO_POSITIVE_REGULATION_OF_PHOSPHORUS_METABOLIC_PROCESS	22	-0.7330492	0.80414015
Down Regulated Genes	GO_GLIAL_CELL_DIFFERENTIATION	6	-0.7320228	0.78929186
Down Regulated Genes	GO_MEIOSIS_II_CELL_CYCLE_PROCESS	1	-0.7314906	0.902834
Down Regulated Genes	GO_CELLULAR_RESPONSE_TO_REACTIVE_OXYGEN_SPECIES	9	-0.73095065	0.80322003
Down Regulated Genes	GO_MRNA_EXPORT_FROM_NUCLEUS	1	-0.72930545	0.8836735
Down Regulated Genes	GO_TUBE_DEVELOPMENT	17	-0.72913146	0.7804054
Down Regulated Genes	GO_STEM_CELL_DIFFERENTIATION	5	-0.7289659	0.7823009
Down Regulated Genes	GO_TRANSCRIPTION_DEPENDENT_TETHERING_OF_RNA_POLYMERASE_II_GENE_DNA_AT_NUCL	1	-0.72874653	0.8658777
Down Regulated Genes	GO_MULTI_ORGANISM_LOCALIZATION	1	-0.72739005	0.9001957
Down Regulated Genes	GO_NEGATIVE_REGULATION_OF_ACTIN_FILAMENT_DEPOLYMERIZATION	1	-0.72722757	0.91188526
Down Regulated Genes	GO_REGULATION_OF_NUCLEOTIDE_METABOLIC_PROCESS	1	-0.7264863	0.926
Down Regulated Genes	GO_SEQUESTERING_OF_ACTIN_MONOMERS	1	-0.72610855	0.9346405
Down Regulated Genes	GO_REPLICATION_BORN_DOUBLE_STRAND_BREAK_REPAIR_VIA_SISTER_CHROMATID_EXCHAN(	1	-0.725879	0.90082645
Down Regulated Genes	GO_HOMEOSTASIS_OF_NUMBER_OF_CELLS	5	-0.7258472	0.82123893
Down Regulated Genes	GO_NUCLEAR_PORE_ORGANIZATION	1	-0.72575676	0.8997996
Down Regulated Genes	GO_AMIDE_BIOSYNTHETIC_PROCESS	12	-0.7256576	0.7790894
Down Regulated Genes	GO_POSITIVE_REGULATION_OF_STRESS_ACTIVATED_PROTEIN_KINASE_SIGNALING_CASCADE	3	-0.72534096	0.84398496
Down Regulated Genes	GO_POSITIVE_REGULATION_OF_MAP_KINASE_ACTIVITY	8	-0.72520274	0.78655463
Down Regulated Genes	GO_APOPTOTIC_SIGNALING_PATHWAY	12	-0.7246884	0.79111844
Down Regulated Genes	GO_LIVER_REGENERATION	4	-0.7243366	0.8348457
Down Regulated Genes	GO_REGULATION_OF_DEVELOPMENTAL_GROWTH	7	-0.7240538	0.79115045
Down Regulated Genes	GO_NUCLEAR_PORE_COMPLEX_ASSEMBLY	1	-0.7240109	0.9007937
Down Regulated Genes	GO_PORE_COMPLEX_ASSEMBLY	1	-0.7235506	0.8967611
Down Regulated Genes	GO_REGULATION_OF_SUPEROXIDE_METABOLIC_PROCESS	1	-0.7230889	0.91935486
Down Regulated Genes	GO_REGULATION_OF_PROTEIN_DEPOLYMERIZATION	1	-0.7223357	0.90041494
Down Regulated Genes	GO_NEGATIVE_REGULATION_OF_PROTEIN_CONTAINING_COMPLEX_DISASSEMBLY	1	-0.7219858	0.92927307
Down Regulated Genes	GO_PTERIDINE_CONTAINING_COMPOUND_BIOSYNTHETIC_PROCESS	1	-0.7208274	0.9083503
Down Regulated Genes	GO_PYRUVATE_METABOLIC_PROCESS	4	-0.71977997	0.82851636
Down Regulated Genes	GO_BARBED_END_ACTIN_FILAMENT_CAPPING	1	-0.7188785	0.9386503
Down Regulated Genes	GO_REGULATION_OF_LIPID_LOCALIZATION	3	-0.7188169	0.859375
Down Regulated Genes	GO_MAINTENANCE_OF_PROTEIN_LOCATION_IN_CELL	1	-0.7184583	0.9294606
Down Regulated Genes	GO_PURINE_CONTAINING_COMPOUND_CATABOLIC_PROCESS	2	-0.7183623	0.85315984
Down Regulated Genes	GO_REGULATION_OF_REMOVAL_OF_SUPEROXIDE_RADICALS	1	-0.7179732	0.9143969
Down Regulated Genes	GO_PROTEIN_LOCALIZATION_TO_GOLGI_APPARATUS	1	-0.7179121	0.9230769
Down Regulated Genes	GO_RESPONSE_TO_METHOTREXATE	1	-0.71755433	0.92753625
Down Regulated Genes	GO_LIPID_STORAGE	3	-0.71750826	0.84615386
Down Regulated Genes	GO_REGULATION_OF_GLIOGENESIS	6	-0.7173537	0.81754386
Down Regulated Genes	GO_CELLULAR_RESPONSE_TO_OXYGEN_RADICAL	1	-0.7169734	0.92561984
Down Regulated Genes	GO_POSITIVE_REGULATION_OF_GENE_EXPRESSION_EPIGENETIC	2	-0.71622473	0.8515901
Down Regulated Genes	GO_REGULATION_OF_LAMELLIPODIUM_ORGANIZATION	1	-0.71562374	0.9219331
Down Regulated Genes	GO_ACTIN_MODIFICATION	1	-0.7150341	0.9257028
Down Regulated Genes	GO_RESPONSE_TO_OXYGEN_RADICAL	1	-0.7149865	0.9301397
Down Regulated Genes	GO_MAINTENANCE_OF_PROTEIN_LOCATION	1	-0.7135648	0.91170824
Down Regulated Genes	GO_POSITIVE_REGULATION_OF_NITRIC_OXIDE_SYNTHASE_ACTIVITY	1	-0.71313345	0.92121214
Down Regulated Genes	GO_CELLULAR_RESPONSE_TO_TOXIC_SUBSTANCE	1	-0.7129726	0.9302326
Down Regulated Genes	GO_POSITIVE_REGULATION_OF_RESPONSE_TO_BIOTIC_STIMULUS	4	-0.71297204	0.875
Down Regulated Genes	GO_ACTIN_FILAMENT_DEPOLYMERIZATION	1	-0.71262676	0.92899406
Down Regulated Genes	GO_POSITIVE_REGULATION_OF_INNATE_IMMUNE_RESPONSE	4	-0.7123561	0.8460145
Down Regulated Genes	GO_RIBOSOME_BIOGENESIS	2	-0.7117838	0.86811024
Down Regulated Genes	GO_DEPYRIMIDINATION	1	-0.71120924	0.94789577
Down Regulated Genes	GO_TETRAHYDROBIOPTERIN_METABOLIC_PROCESS	1	-0.71031946	0.9188641
Down Regulated Genes	GO_TETRAHYDROFOLATE_BIOSYNTHETIC_PROCESS	1	-0.71001697	0.9354839

Down Regulated Genes	GO_POSITIVE_REGULATION_OF_MONOOXYGENASE_ACTIVITY	1	-0.70995307	0.9329389
Down Regulated Genes	GO_PROTEIN_POLYUBIQUITINATION	5	-0.7097197	0.8165785
Down Regulated Genes	GO_DIOL_METABOLIC_PROCESS	1	-0.7093343	0.918
Down Regulated Genes	GO_POSITIVE_REGULATION_OF_CELL_PROJECTION_ORGANIZATION	6	-0.7090267	0.81296295
Down Regulated Genes	GO_PROSTATE_GLAND_GROWTH	2	-0.70899767	0.87758946
Down Regulated Genes	GO_REGULATION_OF_CELLULAR_COMPONENT_SIZE	9	-0.70834523	0.8067227
Down Regulated Genes	GO_SUPEROXIDE_METABOLIC_PROCESS	1	-0.7082533	0.9409449
Down Regulated Genes	GO_DETOXIFICATION	1	-0.7082321	0.94466406
Down Regulated Genes	GO_HISTONE_H3_K27_METHYLATION	2	-0.7081047	0.8754717
Down Regulated Genes	GO_MAMMARY_GLAND_DEVELOPMENT	4	-0.70773786	0.8373702
Down Regulated Genes	GO_REGULATION_OF_RESPONSE_TO_REACTIVE_OXYGEN_SPECIES	1	-0.70745796	0.91050583
Down Regulated Genes	GO_INTERLEUKIN_1_PRODUCTION	3	-0.70720774	0.84734136
Down Regulated Genes	GO_DIOL_BIOSYNTHETIC_PROCESS	1	-0.7070797	0.9312377
Down Regulated Genes	GO_PROSTATE_GLAND_DEVELOPMENT	2	-0.7069834	0.875226
Down Regulated Genes	GO_LAMELLIPODIUM_ORGANIZATION	1	-0.70693034	0.9395161
Down Regulated Genes	GO_RIBONUCLEOPROTEIN_COMPLEX_BIOGENESIS	2	-0.7069151	0.8764045
Down Regulated Genes	GO_CELLULAR_OXIDANT_DETOXIFICATION	1	-0.70652354	0.93890023
Down Regulated Genes	GO_FOLIC_ACID_CONTAINING_COMPOUND_BIOSYNTHETIC_PROCESS	1	-0.7063634	0.93737376
Down Regulated Genes	GO_PYRIMIDINE_NUCLEOTIDE_CATABOLIC_PROCESS	1	-0.70489377	0.95714283
Down Regulated Genes	GO_BASE_EXCISION_REPAIR_AP_SITE_FORMATION	1	-0.70331544	0.9551657
Down Regulated Genes	GO_REGULATION_OF_LIPID_STORAGE	3	-0.70324415	0.862254
Down Regulated Genes	GO_INTRINSIC_APOPTOTIC_SIGNALING_PATHWAY_IN_RESPONSE_TO_OXIDATIVE_STRESS	1	-0.7025616	0.91129035
Down Regulated Genes	GO_REGULATION_OF_LAMELLIPODIUM_ASSEMBLY	1	-0.7020092	0.9525617
Down Regulated Genes	GO_LAMELLIPODIUM_ASSEMBLY	1	-0.70167226	0.93560606
Down Regulated Genes	GO_POLYOL_BIOSYNTHETIC_PROCESS	1	-0.7012847	0.9328063
Down Regulated Genes	GO_REGULATION_OF_SIGNALING_RECEPTOR_ACTIVITY	2	-0.7008335	0.89373815
Down Regulated Genes	GO_CELLULAR_POLYSACCHARIDE_CATABOLIC_PROCESS	1	-0.70065045	0.95564514
Down Regulated Genes	GO_NEGATIVE_REGULATION_OF_DNA_BINDING_TRANSCRIPTION_FACTOR_ACTIVITY	2	-0.699363	0.88846153
Down Regulated Genes	GO_POSITIVE_REGULATION_OF_ADAPTIVE_IMMUNE_RESPONSE	4	-0.69931096	0.85557586
Down Regulated Genes	GO_NEGATIVE_REGULATION_OF_PROTEIN_MODIFICATION_PROCESS	10	-0.6988887	0.8101933
Down Regulated Genes	GO_POLYSACCHARIDE_CATABOLIC_PROCESS	1	-0.6986348	0.94949496
Down Regulated Genes	GO_RNA_PHOSPHODIESTER_BOND_HYDROLYSIS_EXONUCLEOLYTIC	2	-0.69860846	0.8752363
Down Regulated Genes	GO_SCF_DEPENDENT_PROTEASOMAL_UBIQUITIN_DEPENDENT_PROTEIN_CATABOLIC_PROCESS	2	-0.6965542	0.8953271
Down Regulated Genes	GO_MAINTENANCE_OF_CELL_NUMBER	2	-0.69572973	0.8766355
Down Regulated Genes	GO_CHROMATIN_SILENCING	2	-0.6952374	0.87265915
Down Regulated Genes	GO_REGULATION_OF_PROTEIN_POLYMERIZATION	7	-0.6949727	0.82867134
Down Regulated Genes	GO_HETEROPHILIC_CELL_CELL_ADHESION_VIA_PLASMA_MEMBRANE_CELL_ADHESION_MOLEC	2	-0.69454736	0.8687259
Down Regulated Genes	GO_DEOXYRIBOSE_PHOSPHATE_CATABOLIC_PROCESS	1	-0.6944791	0.9525692
Down Regulated Genes	GO_CARBOHYDRATE_DERIVATIVE_BIOSYNTHETIC_PROCESS	6	-0.6937932	0.8607383
Down Regulated Genes	GO_CELLULAR_RESPONSE_TO_INSULIN_STIMULUS	2	-0.69341743	0.8738574
Down Regulated Genes	GO_NUCLEOSIDE_MONOPHOSPHATE_METABOLIC_PROCESS	4	-0.69300085	0.8660551
Down Regulated Genes	GO_REGULATION_OF_GLYCOGEN_CATABOLIC_PROCESS	1	-0.69230175	0.96007985
Down Regulated Genes	GO_PROTEIN_CATABOLIC_PROCESS	13	-0.6922381	0.8357964
Down Regulated Genes	GO_SEPTUM_PRIMUM_DEVELOPMENT	1	-0.69181025	0.9762376
Down Regulated Genes	GO_G0_TO_G1_TRANSITION	2	-0.69168204	0.86778396
Down Regulated Genes	GO_CELLULAR_CARBOHYDRATE_BIOSYNTHETIC_PROCESS	1	-0.69128937	0.9719439
Down Regulated Genes	GO_METHYLGLYOXAL_METABOLIC_PROCESS	1	-0.6912084	0.9576108
Down Regulated Genes	GO_REGULATION_OF_CELLULAR_CARBOHYDRATE_CATABOLIC_PROCESS	1	-0.69005334	0.97604793
Down Regulated Genes	GO_GLYCOGEN_BIOSYNTHETIC_PROCESS	1	-0.6886637	0.95866144
Down Regulated Genes	GO_POLYSACCHARIDE_BIOSYNTHETIC_PROCESS	1	-0.68845636	0.9577735
Down Regulated Genes	GO_REGULATION_OF_GLUCAN_BIOSYNTHETIC_PROCESS	1	-0.6872841	0.96868885
Down Regulated Genes	GO_ICOSANOID_METABOLIC_PROCESS	3	-0.68657225	0.86174244
Down Regulated Genes	GO_HISTONE_H4_K20_METHYLATION	1	-0.68653077	0.957935
Down Regulated Genes	GO_TOOTH_ERUPTION	2	-0.6861006	0.8773946
Down Regulated Genes	GO_REGULATION_OF_GLYCOGEN_METABOLIC_PROCESS	1	-0.6859958	0.9727463
Down Regulated Genes	GO_CELLULAR_CARBOHYDRATE_CATABOLIC_PROCESS	1	-0.68515295	0.953629
Down Regulated Genes	GO_POSITIVE_REGULATION_OF_LYMPHOCYTE_MEDIATED_IMMUNITY	4	-0.68460226	0.87603307
Down Regulated Genes	GO_REGULATION_OF_POLYSACCHARIDE_METABOLIC_PROCESS	1	-0.6844734	0.9573643
Down Regulated Genes	GO_PSEUDOURIDINE_SYNTHESIS	1	-0.6836491	0.96755725
Down Regulated Genes	GO_REGULATION_OF_GLUCOSE_METABOLIC_PROCESS	1	-0.68358856	0.9596929
Down Regulated Genes	GO_POSITIVE_REGULATION_OF_ISOTYPE_SWITCHING_TO_IGA_ISOTYPES	1	-0.6833225	0.9775051
Down Regulated Genes	GO_ATRIAL_SEPTUM_DEVELOPMENT	1	-0.68267363	0.97119343
Down Regulated Genes	GO_NCRNA_PROCESSING	2	-0.68228525	0.90590405
Down Regulated Genes	GO_REGULATION_OF_CARBOHYDRATE_BIOSYNTHETIC_PROCESS	1	-0.6822486	0.97556007
Down Regulated Genes	GO_CARDIAC_ATRIUM_MORPHOGENESIS	1	-0.6822473	0.978308
Down Regulated Genes	GO_DOUBLE_STRAND_BREAK_REPAIR_VIA_SINGLE_STRAND_ANNEALING	1	-0.68217164	0.9791667
Down Regulated Genes	GO_REGULATION_OF_CELLULAR_CARBOHYDRATE_METABOLIC_PROCESS	1	-0.68134683	0.97318006
Down Regulated Genes	GO_ATRIAL_SEPTUM_PRIMUM_MORPHOGENESIS	1	-0.6810669	0.96525097
Down Regulated Genes	GO_ISOTYPE_SWITCHING_TO_IGA_ISOTYPES	1	-0.6802211	0.95991564
Down Regulated Genes	GO_CARDIAC_ATRIUM_DEVELOPMENT	1	-0.6789834	0.9717172

Down Regulated Genes	GO_ATRIAL_SEPTUM_MORPHOGENESIS	1	-0.67828727	0.9561753
Down Regulated Genes	GO_CELL_MORPHOGENESIS_INVOLVED_IN_NEURON_DIFFERENTIATION	9	-0.6775789	0.8400703
Down Regulated Genes	GO_PROTEIN_HOMOTETRAMERIZATION	1	-0.67752427	0.9881188
Down Regulated Genes	GO_RNA_MODIFICATION	1	-0.6769472	0.969112
Down Regulated Genes	GO_EMBRYONIC_BRAIN_DEVELOPMENT	1	-0.6766589	0.9898374
Down Regulated Genes	GO_HISTONE_H3_K36_METHYLATION	1	-0.67653376	0.9838384
Down Regulated Genes	GO_ORGANONITROGEN_COMPOUND_BIOSYNTHETIC_PROCESS	18	-0.6751962	0.8559871
Down Regulated Genes	GO_CARNITINE_BIOSYNTHETIC_PROCESS	1	-0.67469	0.9893162
Down Regulated Genes	GO_DEVELOPMENTAL_PROCESS_INVOLVED_IN_REPRODUCTION	18	-0.67351395	0.8390411
Down Regulated Genes	GO_TELENCEPHALON_REGIONALIZATION	1	-0.6729473	0.9895833
Down Regulated Genes	GO_SERINE_FAMILY_AMINO_ACID_CATABOLIC_PROCESS	1	-0.6728335	0.98630136
Down Regulated Genes	GO_NEGATIVE_REGULATION_OF_PHOSPHORUS_METABOLIC_PROCESS	10	-0.67266124	0.8388704
Down Regulated Genes	GO_GLYCINE_METABOLIC_PROCESS	1	-0.6719685	0.9829424
Down Regulated Genes	GO_ALPHA_AMINO_ACID_CATABOLIC_PROCESS	1	-0.6716502	0.9800399
Down Regulated Genes	GO_POSITIVE_REGULATION_OF_RNA_SPLICING	1	-0.67100316	0.99201596
Down Regulated Genes	GO_CARNITINE_METABOLIC_PROCESS	1	-0.67048395	0.9840319
Down Regulated Genes	GO_FOREBRAIN_REGIONALIZATION	1	-0.67003566	0.99418604
Down Regulated Genes	GO_POSITIVE_REGULATION_OF_CYTOKINE_PRODUCTION_INVOLVED_IN_INFLAMMATORY_RESI	3	-0.6698374	0.88729876
Down Regulated Genes	GO_POSITIVE_REGULATION_OF_MRNA_SPLICING_VIA_SPLICEOSOME	1	-0.6691889	0.9864341
Down Regulated Genes	GO_INTRINSIC_APOPTOTIC_SIGNALING_PATHWAY	4	-0.66823363	0.8687392
Down Regulated Genes	GO_CYTOKINE_PRODUCTION_INVOLVED_IN_INFLAMMATORY_RESPONSE	3	-0.66809815	0.8802947
Down Regulated Genes	GO_HEAD_DEVELOPMENT	16	-0.6672901	0.8622449
Down Regulated Genes	GO_POSITIVE_REGULATION_OF_MRNA_PROCESSING	1	-0.66722393	0.9941292
Down Regulated Genes	GO_NEGATIVE_REGULATION_OF_PHOSPHORYLATION	9	-0.66717345	0.838488
Down Regulated Genes	GO_NUCLEOBASE_BIOSYNTHETIC_PROCESS	1	-0.6664854	0.9941406
Down Regulated Genes	GO_CEREBRAL_CORTEX_REGIONALIZATION	1	-0.665957	0.9940711
Down Regulated Genes	GO_CELLULAR_RESPONSE_TO_MOLECULE_OF_BACTERIAL_ORIGIN	6	-0.6659377	0.8669951
Down Regulated Genes	GO_EMBRYONIC_MORPHOGENESIS	6	-0.6656353	0.88173914
Down Regulated Genes	GO_VASCULOGENESIS	3	-0.665237	0.90613717
Down Regulated Genes	GO_AMINO_ACID_BETAINE_METABOLIC_PROCESS	1	-0.6647267	0.9776876
Down Regulated Genes	GO_PURINE_NUCLEOBASE_BIOSYNTHETIC_PROCESS	1	-0.66460687	0.99201596
Down Regulated Genes	GO_NEGATIVE_REGULATION_OF_TRANSFERASE_ACTIVITY	8	-0.6639799	0.8475712
Down Regulated Genes	GO_NIK_NF_KAPPAB_SIGNALING	3	-0.66362965	0.89721256
Down Regulated Genes	GO_NCRNA_METABOLIC_PROCESS	2	-0.6632667	0.9056261
Down Regulated Genes	GO_REGULATION_OF_MICROTUBULE_POLYMERIZATION	3	-0.66237867	0.9064748
Down Regulated Genes	GO_AMINO_ACID_BETAINE_BIOSYNTHETIC_PROCESS	1	-0.6619396	0.984127
Down Regulated Genes	GO_CELLULAR_RESPONSE_TO_BIOTIC_STIMULUS	6	-0.65998864	0.863388
Down Regulated Genes	GO_CELLULAR_AMINO_ACID_CATABOLIC_PROCESS	1	-0.657073	0.9941973
Down Regulated Genes	GO_PRODUCTION_OF_MOLECULAR_MEDIATOR_INVOLVED_IN_INFLAMMATORY_RESPONSE	3	-0.6517284	0.9148936
Down Regulated Genes	GO_REGULATION_OF_CELL_SIZE	5	-0.6508112	0.8946459
Down Regulated Genes	GO_BONE_MINERALIZATION	2	-0.6470563	0.92897195
Down Regulated Genes	GO_RESPONSE_TO_DRUG	15	-0.64362913	0.87603307
Down Regulated Genes	GO_NUCLEOSIDE_PHOSPHATE_BIOSYNTHETIC_PROCESS	5	-0.63546616	0.8934281
Down Regulated Genes	GO_REGIONALIZATION	4	-0.6339634	0.9130435
Down Regulated Genes	GO_NUCLEAR_EXPORT	3	-0.6309261	0.9334501
Down Regulated Genes	GO_NUCLEOSIDE_MONOPHOSPHATE_BIOSYNTHETIC_PROCESS	3	-0.6287639	0.9323583
Down Regulated Genes	GO_STEM_CELL_PROLIFERATION	6	-0.6258974	0.9172297
Down Regulated Genes	GO_POSITIVE_REGULATION_OF_PROTEIN_METABOLIC_PROCESS	32	-0.6250622	0.89398736
Down Regulated Genes	GO_GLIOGENESIS	8	-0.62414354	0.9177102
Down Regulated Genes	GO_REGULATION_OF_INNATE_IMMUNE_RESPONSE	5	-0.62280315	0.91803277
Down Regulated Genes	GO_PROTEOLYSIS	20	-0.62213796	0.9021207
Down Regulated Genes	GO_CENTRAL_NERVOUS_SYSTEM_DEVELOPMENT	17	-0.61585325	0.9076175
Down Regulated Genes	GO_POSITIVE_REGULATION_OF_JUN_KINASE_ACTIVITY	2	-0.6143808	0.9461967
Down Regulated Genes	GO_REGULATION_OF_GROWTH	9	-0.6133676	0.89045936
Down Regulated Genes	GO_POSITIVE_REGULATION_OF_ORGANELLE_ORGANIZATION	17	-0.612729	0.89361703
Down Regulated Genes	GO_REGULATION_OF_JUN_KINASE_ACTIVITY	2	-0.61025786	0.9501845
Down Regulated Genes	GO_TISSUE_REMODELING	3	-0.60843146	0.9352381
Down Regulated Genes	GO_POSITIVE_REGULATION_OF_PLASMA_MEMBRANE_BOUNDED_CELL_PROJECTION_ASSEMBL	2	-0.6073663	0.9581749
Down Regulated Genes	GO_REGULATION_OF_ADAPTIVE_IMMUNE_RESPONSE	5	-0.601334	0.9312268
Down Regulated Genes	GO_ALCOHOL_METABOLIC_PROCESS	7	-0.5996195	0.932409
Down Regulated Genes	GO_POSITIVE_REGULATION_OF_DNA_BIOSYNTHETIC_PROCESS	2	-0.598308	0.9669118
Down Regulated Genes	GO_REGULATION_OF_LYMPHOCYTE_MEDIATED_IMMUNITY	5	-0.59777784	0.92633516
Down Regulated Genes	GO_MITOCHONDRION_ORGANIZATION	7	-0.5974669	0.94188035
Down Regulated Genes	GO_VESICLE_ORGANIZATION	3	-0.5954915	0.9461806
Down Regulated Genes	GO_RESPONSE_TO_UV	5	-0.5888496	0.9477352
Down Regulated Genes	GO_GLANDULAR_EPITHELIAL_CELL_DIFFERENTIATION	2	-0.587872	0.9682836
Down Regulated Genes	GO_RESPONSE_TO_PEPTIDE_HORMONE	6	-0.5835595	0.9390681
Down Regulated Genes	GO_RESPONSE_TO_REACTIVE_OXYGEN_SPECIES	11	-0.583536	0.9196581
Down Regulated Genes	GO_HOMOLOGOUS_CHROMOSOME_SEGREGATION	5	-0.58175486	0.9540636
Down Regulated Genes	GO_MYD88_INDEPENDENT_TOLL_LIKE_RECEPTOR_SIGNALING_PATHWAY	2	-0.5805267	0.97321427

Down Regulated Genes	GO_OSTEOCLAST_DIFFERENTIATION	3	-0.5765999	0.9556377
Down Regulated Genes	GO_GLIAL_CELL_ACTIVATION	3	-0.568989	0.965642
Down Regulated Genes	GO_RESPONSE_TO_TEMPERATURE_STIMULUS	6	-0.56647474	0.9444444
Down Regulated Genes	GO_LEUKOCYTE_ACTIVATION_INVOLVED_IN_INFLAMMATORY_RESPONSE	3	-0.5639212	0.9680451
Down Regulated Genes	GO_INTERLEUKIN_1_BETA_PRODUCTION	2	-0.5632936	0.9734345
Down Regulated Genes	GO_REGULATION_OF_RESPONSE_TO_BIOTIC_STIMULUS	6	-0.56151175	0.9370861
Down Regulated Genes	GO_NEUROINFLAMMATORY_RESPONSE	3	-0.5611718	0.9643494
Down Regulated Genes	GO_REGULATION_OF_MACROPHAGE_ACTIVATION	3	-0.56002825	0.9680451
Down Regulated Genes	GO_CHROMOSOME_ORGANIZATION_INVOLVED_IN_MEIOTIC_CELL_CYCLE	6	-0.5539295	0.9699647
Down Regulated Genes	GO_NEGATIVE_CHEMOTAXIS	2	-0.54184985	0.9833024
Down Regulated Genes	GO_REGULATION_OF_MAP_KINASE_ACTIVITY	10	-0.54097915	0.96127945
Down Regulated Genes	GO_REGULATION_OF_AXON_GUIDANCE	2	-0.54015493	0.9839858
Down Regulated Genes	GO_CHONDROCYTE_DEVELOPMENT	2	-0.53808254	0.99248123
Down Regulated Genes	GO_POSITIVE_REGULATION_OF_CELLULAR_COMPONENT_ORGANIZATION	32	-0.5378683	0.9611198
Down Regulated Genes	GO_NEGATIVE_REGULATION_OF_AXON_EXTENSION	2	-0.5375467	0.9909091
Down Regulated Genes	GO_POSITIVE_REGULATION_OF_REACTIVE_OXYGEN_SPECIES_METABOLIC_PROCESS	2	-0.5375179	0.9811321
Down Regulated Genes	GO_SEMAPHORIN_PLEXIN_SIGNALING_PATHWAY	2	-0.5369248	0.987156
Down Regulated Genes	GO_NEURON_PROJECTION_EXTENSION_INVOLVED_IN_NEURON_PROJECTION_GUIDANCE	2	-0.53556377	0.99298245
Down Regulated Genes	GO_AXON_DEVELOPMENT	9	-0.5348694	0.94789916
Down Regulated Genes	GO_CHONDROCYTE_DEVELOPMENT_INVOLVED_IN_ENDOCHONDRAL_BONE_MORPHOGENESIS	2	-0.5309592	0.9944238
Down Regulated Genes	GO_GROWTH_PLATE_CARTILAGE_CHONDROCYTE_DEVELOPMENT	2	-0.5307903	0.98892987
Down Regulated Genes	GO_CHONDROCYTE_DIFFERENTIATION_INVOLVED_IN_ENDOCHONDRAL_BONE_MORPHOGENES	2	-0.5300902	0.9920319
Down Regulated Genes	GO_NEGATIVE_REGULATION_OF_AXON_GUIDANCE	2	-0.52945304	1
Down Regulated Genes	GO_NEGATIVE_REGULATION_OF_CHEMOTAXIS	2	-0.5278797	0.9878472
Down Regulated Genes	GO_GROWTH_PLATE_CARTILAGE_CHONDROCYTE_DIFFERENTIATION	2	-0.52417034	0.9891501
Down Regulated Genes	GO_RESPONSE_TO_PEPTIDE	7	-0.5207185	0.96644294
Down Regulated Genes	GO_ENDOCYTOSIS	5	-0.5198954	0.9654577
Down Regulated Genes	GO_PRODUCTION_OF_SMALL_RNA_INVOLVED_IN_GENE_SILENCING_BY_RNA	2	-0.51674604	0.9980392
Down Regulated Genes	GO_PROTEIN_KINASE_B_SIGNALING	6	-0.51501626	0.97333336
Down Regulated Genes	GO_POSITIVE_REGULATION_OF_NEURON_DEATH	2	-0.51413155	0.9981132
Down Regulated Genes	GO_BRONCHUS_DEVELOPMENT	2	-0.51238227	0.99622643
Down Regulated Genes	GO_NEGATIVE_REGULATION_OF_GENE_SILENCING	2	-0.50671375	0.99626863
Down Regulated Genes	GO_MYOTUBE_DIFFERENTIATION	3	-0.5054688	0.9892857
Down Regulated Genes	GO_NEGATIVE_REGULATION_OF_POSTTRANSCRIPTIONAL_GENE_SILENCING	2	-0.5054582	0.99613154
Down Regulated Genes	GO_NEGATIVE_REGULATION_OF_PRODUCTION_OF_MIRNAS_INVOLVED_IN_GENE_SILENCING_I	2	-0.5037351	0.99810606
Down Regulated Genes	GO_REGULATION_OF_PRODUCTION_OF_SMALL_RNA_INVOLVED_IN_GENE_SILENCING_BY_RNA	2	-0.50362056	0.99809885
Down Regulated Genes	GO_REGULATION_OF_MICROTUBULE_POLYMERIZATION_OR_DEPOLYMERIZATION	4	-0.5031827	0.9831933
Down Regulated Genes	GO_B_CELL_ACTIVATION	7	-0.50131834	0.9767857
Down Regulated Genes	GO_REGENERATION	10	-0.50102895	0.9831366
Down Regulated Genes	GO_NEGATIVE_REGULATION_OF_MOLECULAR_FUNCTION	16	-0.47546995	0.9934211
Down Regulated Genes	GO_POSITIVE_REGULATION_OF_NERVOUS_SYSTEM_DEVELOPMENT	14	-0.4397043	0.98811543
Down Regulated Genes	GO_COGNITION	6	-0.4324661	0.99647886
Down Regulated Genes	GO_RESPONSE_TO_LIGHT_STIMULUS	8	-0.41995645	0.99832773
Down Regulated Genes	GO_POSITIVE_REGULATION_OF_CELL_DEVELOPMENT	15	-0.38481286	0.99833333
Upregulated Genes	GO_REGULATION_OF_SECRETION	12	2.5624917	0
Upregulated Genes	GO_CELL_CELL_ADHESION	16	2.4787588	0
Upregulated Genes	GO_RESPONSE_TO_MECHANICAL_STIMULUS	9	2.461543	0
Upregulated Genes	GO_BIOLOGICAL_ADHESION	25	2.4460537	0
Upregulated Genes	GO_SECRETION	22	2.4443712	0
Upregulated Genes	GO_REGULATION_OF_PEPTIDE_SECRETION	10	2.3193183	0
Upregulated Genes	GO_NEGATIVE_REGULATION_OF_MULTICELLULAR_ORGANISMAL_PROCESS	22	2.302808	0
Upregulated Genes	GO_POSITIVE_REGULATION_OF_PEPTIDE_SECRETION	8	2.2983627	0
Upregulated Genes	GO_REGULATION_OF_CELL_CELL_ADHESION	10	2.2954426	0.0025
Upregulated Genes	GO_POSITIVE_REGULATION_OF_SECRETION	8	2.2805402	0
Upregulated Genes	GO_REGULATION_OF_IMMUNE_SYSTEM_PROCESS	26	2.2667837	0
Upregulated Genes	GO_CELLULAR_ION_HOMEOSTASIS	10	2.258418	0.002421308
Upregulated Genes	GO_PEPTIDE_SECRETION	11	2.2197447	0
Upregulated Genes	GO_REGULATION_OF_CELL_ADHESION	16	2.2084398	0
Upregulated Genes	GO_HEART_PROCESS	5	2.1840303	0
Upregulated Genes	GO_MUSCLE_CONTRACTION	6	2.1776643	0
Upregulated Genes	GO_CELL_ACTIVATION	28	2.158036	0
Upregulated Genes	GO_MUSCLE_SYSTEM_PROCESS	7	2.1565936	0
Upregulated Genes	GO_DEFENSE_RESPONSE	29	2.1551282	0
Upregulated Genes	GO_EXTRACELLULAR_STRUCTURE_ORGANIZATION	7	2.1303735	0
Upregulated Genes	GO_CHEMICAL_HOMEOSTASIS	20	2.1145518	0
Upregulated Genes	GO_ION_HOMEOSTASIS	12	2.080079	0
Upregulated Genes	GO_CIRCULATORY_SYSTEM_PROCESS	8	2.075372	0
Upregulated Genes	GO_CELLULAR_RESPONSE_TO_EXTERNAL_STIMULUS	6	2.069569	0
Upregulated Genes	GO_REGULATION_OF_RESPONSE_TO_EXTERNAL_STIMULUS	22	2.0623224	0.002754821
Upregulated Genes	GO_POSITIVE_REGULATION_OF_LEUKOCYTE_DIFFERENTIATION	8	2.0498497	0

Upregulated Genes	GO_IMMUNE_EFFECTOR_PROCESS	22	2.043452	0
Upregulated Genes	GO_POSITIVE_REGULATION_OF_CYTOKINE_SECRETION	6	2.0399504	0.002439024
Upregulated Genes	GO_RESPONSE_TO_CAMP	5	2.0368145	0.002304148
Upregulated Genes	GO_SIGNAL_RELEASE	10	2.0064836	0.009950249
Upregulated Genes	GO_NEGATIVE_REGULATION_OF_CELL_ADHESION	10	2.0048513	0.007281554
Upregulated Genes	GO_NEGATIVE_REGULATION_OF_RESPONSE_TO_EXTERNAL_STIMULUS	8	2.0038705	0.004739337
Upregulated Genes	GO_T_CELL_ACTIVATION	12	1.9782112	0.007194245
Upregulated Genes	GO_POSITIVE_REGULATION_OF_TRANSPORT	17	1.9657526	0.002610966
Upregulated Genes	GO_CELLULAR_HOMEOSTASIS	20	1.9317569	0.005235602
Upregulated Genes	GO_REGULATION_OF_TRANSPORT	30	1.9289541	0
Upregulated Genes	GO_CELL_CELL_SIGNALING	28	1.9257648	0.005208334
Upregulated Genes	GO_POSITIVE_REGULATION_OF_ESTABLISHMENT_OF_PROTEIN_LOCALIZATION	13	1.9208581	0.007614213
Upregulated Genes	GO_NEGATIVE_REGULATION_OF_CYTOKINE_PRODUCTION	9	1.9117993	0.014492754
Upregulated Genes	GO_DETECTION_OF_STIMULUS	8	1.9039578	0.014319809
Upregulated Genes	GO_REGULATION_OF_MYELOID_LEUKOCYTE_DIFFERENTIATION	4	1.8968259	0.007263923
Upregulated Genes	GO_POSITIVE_REGULATION_OF_IMMUNE_SYSTEM_PROCESS	22	1.8958136	0.012820513
Upregulated Genes	GO_REGULATION_OF_LEUKOCYTE_DIFFERENTIATION	9	1.8948238	0.01
Upregulated Genes	GO_DEFENSE_RESPONSE_TO_VIRUS	7	1.8937421	0.013986014
Upregulated Genes	GO_REGULATION_OF_PEPTIDE_TRANSPORT	17	1.8826314	0.007731959
Upregulated Genes	GO_REGULATION_OF_T_CELL_ACTIVATION	7	1.8708473	0.007109005
Upregulated Genes	GO_RESPONSE_TO_CYTOKINE	25	1.8598802	0.01344086
Upregulated Genes	GO_LEUKOCYTE_CELL_CELL_ADHESION	7	1.8565634	0.01438849
Upregulated Genes	GO_METAL_ION_HOMEOSTASIS	11	1.856369	0.014423077
Upregulated Genes	GO_RESPONSE_TO_BIOTIC_STIMULUS	25	1.8544415	0.002570694
Upregulated Genes	GO_RESPONSE_TO_INTERFERON_GAMMA	3	1.8530601	0.004807693
Upregulated Genes	GO_REGULATION_OF_CELLULAR_RESPONSE_TO_GROWTH_FACTOR_STIMULUS	6	1.8486053	0.011933174
Upregulated Genes	GO_MULTI_MULTICELLULAR_ORGANISM_PROCESS	7	1.8480357	0.007211539
Upregulated Genes	GO_POSITIVE_REGULATION_OF_MYELOID_LEUKOCYTE_DIFFERENTIATION	4	1.8467594	0.008928572
Upregulated Genes	GO_NEGATIVE_REGULATION_OF_INFLAMMATORY_RESPONSE	3	1.8350719	0.002277904
Upregulated Genes	GO_DEFENSE_RESPONSE_TO_OTHER_ORGANISM	18	1.8236932	0.025252525
Upregulated Genes	GO_PLATELET_DEGRANULATION	4	1.8217903	0.002247191
Upregulated Genes	GO_REGULATION_OF_HEMOPOIESIS	11	1.8183793	0.007317073
Upregulated Genes	GO_NEGATIVE_REGULATION_OF_DEFENSE_RESPONSE	3	1.8168101	0.006976744
Upregulated Genes	GO_DIVALENT_INORGANIC_CATION_HOMEOSTASIS	9	1.814068	0.02676399
Upregulated Genes	GO_LEUKOCYTE_DIFFERENTIATION	19	1.8081862	0.020671835
Upregulated Genes	GO_RESPONSE_TO_RETINOIC_ACID	3	1.8065903	0
Upregulated Genes	GO_CELLULAR_RESPONSE_TO_MECHANICAL_STIMULUS	4	1.8049812	0.014285714
Upregulated Genes	GO_NEGATIVE_REGULATION_OF_CATABOLIC_PROCESS	4	1.8027525	0.008492569
Upregulated Genes	GO_REGULATION_OF_BLOOD_CIRCULATION	4	1.7961054	0.016908212
Upregulated Genes	GO_CYTOKINE_MEDIATED_SIGNALING_PATHWAY	14	1.795572	0.007894737
Upregulated Genes	GO_CYTOKINE_PRODUCTION	16	1.7935883	0.012406948
Upregulated Genes	GO_REGULATION_OF_MYELOID_CELL_DIFFERENTIATION	6	1.7883337	0.017391304
Upregulated Genes	GO_RESPONSE_TO_CALCIUM_ION	5	1.7812297	0.009153318
Upregulated Genes	GO_GRANULOCYTE_MIGRATION	5	1.7743248	0.009174312
Upregulated Genes	GO_SUPRAMOLECULAR_FIBER_ORGANIZATION	16	1.7666565	0.018469658
Upregulated Genes	GO_RESPONSE_TO_NUTRIENT	9	1.763759	0.022222223
Upregulated Genes	GO_REGULATION_OF_INFLAMMATORY_RESPONSE	10	1.7623216	0.024390243
Upregulated Genes	GO_NEGATIVE_REGULATION_OF_IMMUNE_SYSTEM_PROCESS	6	1.7507937	0.017902814
Upregulated Genes	GO_LEUKOCYTE_MIGRATION	9	1.7479843	0.027295286
Upregulated Genes	GO_REGULATION_OF_EPIDERMIS_DEVELOPMENT	5	1.7421463	0.029345373
Upregulated Genes	GO_LYMPHOCYTE_ACTIVATION	17	1.7398418	0.02764977
Upregulated Genes	GO_RESPONSE_TO_VITAMIN	6	1.7360581	0.013793103
Upregulated Genes	GO_EXTRACELLULAR_MATRIX_DISASSEMBLY	4	1.734988	0.013215859
Upregulated Genes	GO_PLATELET_ACTIVATION	4	1.7339362	0.013215859
Upregulated Genes	GO_POSITIVE_REGULATION_OF_LYMPHOCYTE_DIFFERENTIATION	4	1.7300917	0.009195402
Upregulated Genes	GO_WOUND_HEALING	12	1.7253228	0.025287356
Upregulated Genes	GO_POSITIVE_REGULATION_OF_HEMOPOIESIS	9	1.7244102	0.022988506
Upregulated Genes	GO_T_CELL_PROLIFERATION	6	1.7138191	0.016666668
Upregulated Genes	GO_NEGATIVE_REGULATION_OF_CELL_CELL_ADHESION	6	1.7131805	0.016091954
Upregulated Genes	GO_ACTIN_MEDIATED_CELL_CONTRACTION	3	1.7100035	0.008547009
Upregulated Genes	GO_CELLULAR_RESPONSE_TO_VIRUS	4	1.7023865	0.028571429
Upregulated Genes	GO_REGULATION_OF_IMMUNE_RESPONSE	17	1.6995517	0.025839793
Upregulated Genes	GO_RESPONSE_TO_BACTERIUM	13	1.6980312	0.016786572
Upregulated Genes	GO_CYTOKINE_SECRETION	9	1.6916919	0.027713627
Upregulated Genes	GO_ACTIN_FILAMENT_BASED_MOVEMENT	3	1.6854085	0.010752688
Upregulated Genes	GO_POSITIVE_REGULATION_OF_CELL_CELL_ADHESION	7	1.6790338	0.021951219
Upregulated Genes	GO_OSSIFICATION	9	1.6774817	0.034324944
Upregulated Genes	GO_IMMUNE_RESPONSE_REGULATING_SIGNALING_PATHWAY	7	1.6773586	0.039408866
Upregulated Genes	GO_CD4_POSITIVE_ALPHA_BETA_T_CELL_ACTIVATION	6	1.6742119	0.028235294
Upregulated Genes	GO_ERK1_AND_ERK2_CASCADE	8	1.667179	0.043902438

Upregulated Genes	GO_CARDIAC_MUSCLE_CONTRACTION	3	1.6669488	0.01826484
Upregulated Genes	GO_ANTIGEN_RECEPTOR_MEDIATED_SIGNALING_PATHWAY	5	1.6601208	0.032581452
Upregulated Genes	GO_STRIATED_MUSCLE_CONTRACTION	3	1.655233	0.010706638
Upregulated Genes	GO_CELLULAR_RESPONSE_TO_EXTRACELLULAR_STIMULUS	3	1.6532891	0.016091954
Upregulated Genes	GO_G_PROTEIN_COUPLED_RECEPTOR_SIGNALING_PATHWAY_COUPLED_TO_CYCLIC_NUCLEOTI	3	1.6509378	0.024742268
Upregulated Genes	GO_RESPONSE_TO_PURINE_CONTAINING_COMPOUND	7	1.6485622	0.035799522
Upregulated Genes	GO_REGULATION_OF_DEFENSE_RESPONSE	14	1.6455053	0.042755343
Upregulated Genes	GO_MYELOID_LEUKOCYTE_MIGRATION	7	1.641133	0.050691243
Upregulated Genes	GO_REGULATION_OF_LEUKOCYTE_MIGRATION	5	1.6368831	0.026252983
Upregulated Genes	GO_ACTIN_FILAMENT_ORGANIZATION	9	1.6347222	0.04819277
Upregulated Genes	GO_REGULATION_OF_EPITHELIAL_CELL_DIFFERENTIATION	5	1.6338049	0.03678161
Upregulated Genes	GO_EXOCYTOSIS	9	1.630457	0.05147059
Upregulated Genes	GO_RESPONSE_TO_VITAMIN_D	2	1.6288081	0.016427105
Upregulated Genes	GO_COAGULATION	6	1.628278	0.034313727
Upregulated Genes	GO_REGULATION_OF_CYTOSOLIC_CALCIUM_ION_CONCENTRATION	6	1.6273147	0.038554218
Upregulated Genes	GO_MYELOID_LEUKOCYTE_DIFFERENTIATION	9	1.6262435	0.041062802
Upregulated Genes	GO_REGULATION_OF_KERATINOCYTE_DIFFERENTIATION	3	1.6231697	0.02232143
Upregulated Genes	GO_EPIDERMIS_DEVELOPMENT	11	1.6193359	0.04411765
Upregulated Genes	GO_RESPONSE_TO_EXTRACELLULAR_STIMULUS	11	1.6187078	0.046798028
Upregulated Genes	GO_RESPONSE_TO_ORGANOPHOSPHORUS	7	1.6179403	0.04522613
Upregulated Genes	GO_REGULATION_OF_HORMONE_LEVELS	6	1.6174742	0.032258064
Upregulated Genes	GO_SECOND_MESSENGER_MEDIATED_SIGNALING	3	1.6154498	0.019027485
Upregulated Genes	GO_SKELETAL_SYSTEM_DEVELOPMENT	11	1.6126907	0.05580357
Upregulated Genes	GO_NEGATIVE_REGULATION_OF_CELLULAR_CATABOLIC_PROCESS	3	1.6105145	0.028761063
Upregulated Genes	GO_REGULATION_OF_PROTEIN_LOCALIZATION	24	1.6089842	0.038860105
Upregulated Genes	GO_ANIMAL_ORGAN_MORPHOGENESIS	21	1.6051942	0.05370844
Upregulated Genes	GO_G_PROTEIN_COUPLED_RECEPTOR_SIGNALING_PATHWAY	16	1.5998062	0.042606518
Upregulated Genes	GO_POSITIVE_REGULATION_OF_ALPHA_BETA_T_CELL_DIFFERENTIATION	3	1.5983646	0.03426124
Upregulated Genes	GO_LEUKOCYTE_PROLIFERATION	8	1.596601	0.040572792
Upregulated Genes	GO_MONONUCLEAR_CELL_DIFFERENTIATION	3	1.5922463	0.042035397
Upregulated Genes	GO_RENAL_SYSTEM_PROCESS	2	1.5895128	0.004282655
Upregulated Genes	GO_MYELOID_CELL_DIFFERENTIATION	11	1.5883778	0.046116505
Upregulated Genes	GO_POSITIVE_REGULATION_OF_ALPHA_BETA_T_CELL_ACTIVATION	3	1.5867697	0.03076923
Upregulated Genes	GO_ACTIN_FILAMENT_BASED_PROCESS	20	1.5841734	0.05357143
Upregulated Genes	GO_ALPHA_BETA_T_CELL_ACTIVATION	8	1.5810437	0.042056076
Upregulated Genes	GO_DETECTION_OF_ABIOTIC_STIMULUS	3	1.5807753	0.033039648
Upregulated Genes	GO_POSITIVE_REGULATION_OF_RESPONSE_TO_EXTERNAL_STIMULUS	12	1.5805598	0.057142857
Upregulated Genes	GO_T_CELL_RECEPTOR_SIGNALING_PATHWAY	4	1.5775671	0.039719626
Upregulated Genes	GO_REGULATION_OF_MONOCYTE_DIFFERENTIATION	2	1.5756377	0.021008404
Upregulated Genes	GO_REGULATION_OF_ION_TRANSPORT	6	1.573425	0.0520362
Upregulated Genes	GO_MACROPHAGE_DIFFERENTIATION	2	1.5553447	0.023404256
Upregulated Genes	GO_MACROPHAGE_CHEMOTAXIS	2	1.553282	0.017660044
Upregulated Genes	GO_EMBRYO_IMPLANTATION	3	1.553086	0.040339705
Upregulated Genes	GO_INNATE_IMMUNE_RESPONSE	14	1.5502458	0.06053269
Upregulated Genes	GO_NEGATIVE_REGULATION_OF_CELL_ACTIVATION	5	1.5502216	0.053240743
Upregulated Genes	GO_POSITIVE_REGULATION_OF_TUMOR_NECROSIS_FACTOR_SECRETION	3	1.54991	0.06712963
Upregulated Genes	GO_DETECTION_OF_MECHANICAL_STIMULUS	3	1.5486697	0.037037037
Upregulated Genes	GO_EPITHELIAL_CELL_APOPTOTIC_PROCESS	3	1.5450785	0.0520362
Upregulated Genes	GO_POSITIVE_REGULATION_OF_LEUKOCYTE_CELL_CELL_ADHESION	6	1.5413643	0.06666667
Upregulated Genes	GO_POSITIVE_REGULATION_OF_MONOCYTE_DIFFERENTIATION	2	1.5405729	0.021505376
Upregulated Genes	GO_NEGATIVE_REGULATION_OF_TRANSMEMBRANE_RECEPTOR_PROTEIN_SERINE_THREONINE_	4	1.5395477	0.068432674
Upregulated Genes	GO_HEART_MORPHOGENESIS	4	1.5379772	0.05882353
Upregulated Genes	GO_REGULATION_OF_CATABOLIC_PROCESS	16	1.5320865	0.07349081
Upregulated Genes	GO_SYNAPTIC_SIGNALING	5	1.5316597	0.060096152
Upregulated Genes	GO_POSITIVE_REGULATION_OF_VIRAL_PROCESS	3	1.530631	0.077922076
Upregulated Genes	GO_CYTOPLASMIC_PATTERN_RECOGNITION_RECEPTOR_SIGNALING_PATHWAY_IN_RESPONSE_	3	1.5292754	0.058956917
Upregulated Genes	GO_REGULATION_OF_SYSTEM_PROCESS	7	1.527422	0.08924485
Upregulated Genes	GO_POSITIVE_REGULATION_OF_MYELOID_CELL_DIFFERENTIATION	5	1.5267528	0.060185187
Upregulated Genes	GO_LYMPHOCYTE_DIFFERENTIATION	11	1.5266567	0.05940594
Upregulated Genes	GO_MACROPHAGE_MIGRATION	2	1.525492	0.016563147
Upregulated Genes	GO_NEGATIVE_REGULATION_OF_TYPE_I_INTERFERON_PRODUCTION	3	1.5251341	0.044345897
Upregulated Genes	GO_ACTOMYOSIN_STRUCTURE_ORGANIZATION	9	1.5235851	0.083526686
Upregulated Genes	GO_POSITIVE_REGULATION_OF_INTERFERON_ALPHA_PRODUCTION	3	1.5222682	0.055913977
Upregulated Genes	GO_REGULATION_OF_SYMBIOTIC_PROCESS	3	1.5222285	0.07349666
Upregulated Genes	GO_T_CELL_ACTIVATION_INVOLVED_IN_IMMUNE_RESPONSE	5	1.5215377	0.07538803
Upregulated Genes	GO_RESPONSE_TO_DSRNA	3	1.5215038	0.04347826
Upregulated Genes	GO_CELLULAR_RESPONSE_TO_DSRNA	3	1.5154929	0.052747253
Upregulated Genes	GO_ADHERENS_JUNCTION_ORGANIZATION	2	1.515341	0.02008032
Upregulated Genes	GO_MUSCLE_FIBER_DEVELOPMENT	2	1.5148841	0.02972399
Upregulated Genes	GO_CELLULAR_RESPONSE_TO_EXOGENOUS_DSRNA	3	1.5142614	0.06896552

Upregulated Genes	GO_POSITIVE_REGULATION_OF_CELL_ADHESION	8	1.5114956	0.08168317
Upregulated Genes	GO_INTERFERON_GAMMA_MEDIATED_SIGNALING_PATHWAY	2	1.5089791	0.04585153
Upregulated Genes	GO_POSITIVE_REGULATION_OF_RESPONSE_TO_CYTOKINE_STIMULUS	3	1.5078082	0.054545455
Upregulated Genes	GO_REGULATION_OF_CELL_DIFFERENTIATION	38	1.5071731	0.050938338
Upregulated Genes	GO_POSITIVE_REGULATION_OF_MEMBRANE_PERMEABILITY	2	1.5057502	0.038617887
Upregulated Genes	GO_NEGATIVE_REGULATION_OF_ESTABLISHMENT_OF_PROTEIN_LOCALIZATION	4	1.5054642	0.070938215
Upregulated Genes	GO_REGULATION_OF_MEMBRANE_PERMEABILITY	2	1.5053588	0.02783726
Upregulated Genes	GO_REGULATION_OF_MITOCHONDRIAL_MEMBRANE_PERMEABILITY_INVOLVED_IN_APOPTOTI(	2	1.5018151	0.039045554
Upregulated Genes	GO_SKIN_MORPHOGENESIS	2	1.501043	0.04121475
Upregulated Genes	GO_REGULATION_OF_OSSIFICATION	3	1.4989043	0.06323185
Upregulated Genes	GO_REGULATION_OF_VIRAL_LIFE_CYCLE	2	1.4977825	0.044025157
Upregulated Genes	GO_MITOCHONDRIAL_OUTER_MEMBRANE_PERMEABILIZATION	2	1.4965897	0.03757829
Upregulated Genes	GO_CELL_JUNCTION_ORGANIZATION	12	1.4958215	0.08080808
Upregulated Genes	GO_REGULATION_OF_EPITHELIAL_TO_MESENCHYMAL_TRANSITION	5	1.4954871	0.08237986
Upregulated Genes	GO_RESPONSE_TO_VIRUS	8	1.4952842	0.09873418
Upregulated Genes	GO_PROTEIN_KINASE_A_SIGNALING	2	1.4902409	0.037698414
Upregulated Genes	GO_RESPONSE_TO_MUSCLE_STRETCH	2	1.4901693	0.05394191
Upregulated Genes	GO_RESPONSE_TO_TUMOR_NECROSIS_FACTOR	4	1.4896349	0.0861678
Upregulated Genes	GO_REGULATION_OF_MITOCHONDRION_ORGANIZATION	2	1.4889381	0.032119915
Upregulated Genes	GO_RESPONSE_TO_GROWTH_FACTOR	13	1.4862074	0.092731826
Upregulated Genes	GO_CELLULAR_RESPONSE_TO_CARBOHYDRATE_STIMULUS	2	1.482249	0.04883227
Upregulated Genes	GO_ENDOTHELIUM_DEVELOPMENT	3	1.4817102	0.07439825
Upregulated Genes	GO_CYTOPLASMIC_PATTERN_RECOGNITION_RECEPTOR_SIGNALING_PATHWAY	3	1.4815841	0.07158837
Upregulated Genes	GO_BONE_DEVELOPMENT	6	1.480755	0.085510686
Upregulated Genes	GO_INTERFERON_ALPHA_PRODUCTION	3	1.4806752	0.062068965
Upregulated Genes	GO_MUSCLE_HYPERTROPHY	2	1.4779779	0.027600849
Upregulated Genes	GO_REGULATION_OF_VASCULATURE_DEVELOPMENT	5	1.4762634	0.075
Upregulated Genes	GO_NEGATIVE_REGULATION_OF_T_CELL_PROLIFERATION	2	1.4761486	0.04761905
Upregulated Genes	GO_ALPHA_BETA_T_CELL_DIFFERENTIATION	7	1.4754851	0.08037825
Upregulated Genes	GO_PROTEIN_TETRAMERIZATION	3	1.473306	0.07594936
Upregulated Genes	GO_HORMONE_TRANSPORT	2	1.4730911	0.058956917
Upregulated Genes	GO_ENDOTHELIAL_CELL_DEVELOPMENT	3	1.4726218	0.07748184
Upregulated Genes	GO_STEROL_METABOLIC_PROCESS	4	1.4716489	0.08390023
Upregulated Genes	GO_INFLAMMATORY_RESPONSE	19	1.4714717	0.10443864
Upregulated Genes	GO_REGULATION_OF_EPIDERMAL_CELL_DIFFERENTIATION	4	1.4707642	0.07061503
Upregulated Genes	GO_POSITIVE_REGULATION_OF_VIRAL_LIFE_CYCLE	2	1.4703627	0.03501094
Upregulated Genes	GO_CELLULAR_GLUCOSE_HOMEOSTASIS	2	1.4699483	0.056155507
Upregulated Genes	GO_REGULATION_OF_CELLULAR_CATABOLIC_PROCESS	14	1.4694297	0.07816712
Upregulated Genes	GO_INSULIN_SECRETION	2	1.4684335	0.0625
Upregulated Genes	GO_NEGATIVE_REGULATION_OF_LEUKOCYTE_CELL_CELL_ADHESION	4	1.4671117	0.08944954
Upregulated Genes	GO_SMALL_MOLECULE_BIOSYNTHETIC_PROCESS	14	1.4654897	0.09756097
Upregulated Genes	GO_REGULATION_OF_MUSCLE_CONTRACTION	3	1.4648619	0.07484408
Upregulated Genes	GO_IMMUNE_SYSTEM_DEVELOPMENT	30	1.4646351	0.07880435
Upregulated Genes	GO_PEPTIDE_HORMONE_SECRETION	2	1.4637629	0.05720339
Upregulated Genes	GO_HUMORAL_IMMUNE_RESPONSE	2	1.462569	0.05636743
Upregulated Genes	GO_PROTEIN_FOLDING	2	1.4612904	0.047916666
Upregulated Genes	GO_REGULATION_OF_PEPTIDE_HORMONE_SECRETION	2	1.4603225	0.04897959
Upregulated Genes	GO_REGULATION_OF_MUSCLE_SYSTEM_PROCESS	3	1.4599471	0.08695652
Upregulated Genes	GO_CELLULAR_LIPID_CATABOLIC_PROCESS	3	1.4577539	0.08436214
Upregulated Genes	GO_EPITHELIAL_TO_MESENCHYMAL_TRANSITION	5	1.4573262	0.09047619
Upregulated Genes	GO_POSITIVE_REGULATION_OF_SIGNALING	33	1.4561026	0.08421053
Upregulated Genes	GO_INTERLEUKIN_10_PRODUCTION	3	1.455993	0.09048724
Upregulated Genes	GO_CELL_SUBSTRATE_ADHESION	5	1.4525584	0.10599078
Upregulated Genes	GO_CD4_POSITIVE_ALPHA_BETA_T_CELL_DIFFERENTIATION	5	1.4499915	0.10909091
Upregulated Genes	GO_T_CELL_DIFFERENTIATION	9	1.4487103	0.08495145
Upregulated Genes	GO_NEGATIVE_REGULATION_OF_LEUKOCYTE_PROLIFERATION	2	1.4479821	0.046709128
Upregulated Genes	GO_UNSATURATED_FATTY_ACID_BIOSYNTHETIC_PROCESS	2	1.4475205	0.060215052
Upregulated Genes	GO_INTERFERON_GAMMA_PRODUCTION	3	1.4467541	0.089171976
Upregulated Genes	GO_REGULATION_OF_INSULIN_SECRETION	2	1.4432093	0.072210066
Upregulated Genes	GO_SIGNAL_TRANSDUCTION_BY_PROTEIN_PHOSPHORYLATION	20	1.4420865	0.10501193
Upregulated Genes	GO_NEGATIVE_REGULATION_OF_AUTOPHAGY	2	1.441013	0.06458797
Upregulated Genes	GO_GRANULOCYTE_CHEMOTAXIS	4	1.4407431	0.08232445
Upregulated Genes	GO_NEGATIVE_REGULATION_OF_LYMPHOCYTE_ACTIVATION	4	1.4403956	0.10633484
Upregulated Genes	GO_REGULATION_OF_HORMONE_SECRETION	2	1.4386497	0.06833713
Upregulated Genes	GO_HEMIDESMOSOME_ASSEMBLY	2	1.4340426	0.051282052
Upregulated Genes	GO_PROTEIN_COMPLEX_OLIGOMERIZATION	7	1.4306207	0.11298077
Upregulated Genes	GO_RESPONSE_TO_ABIOTIC_STIMULUS	35	1.4283155	0.10298103
Upregulated Genes	GO_PEPTIDYL_TYROSINE_MODIFICATION	6	1.4251984	0.1294964
Upregulated Genes	GO_REGULATION_OF_LEUKOCYTE_PROLIFERATION	7	1.4245987	0.114754096
Upregulated Genes	GO_MYOFIBRIL_ASSEMBLY	3	1.4237918	0.092307694

Upregulated Genes	GO_POSITIVE_REGULATION_OF_ERK1_AND_ERK2_CASCADE	3	1.4232477	0.10775862
Upregulated Genes	GO_LIPID_CATABOLIC_PROCESS	3	1.4199725	0.081497796
Upregulated Genes	GO_TYPE_III_INTERFERON_PRODUCTION	2	1.4176755	0.07625272
Upregulated Genes	GO_RESPONSE_TO_TRANSFORMING_GROWTH_FACTOR_BETA	7	1.4176246	0.11600928
Upregulated Genes	GO_ZYMOGEN_ACTIVATION	2	1.4147993	0.08137045
Upregulated Genes	GO_MUSCLE_ORGAN_DEVELOPMENT	5	1.4115582	0.1291866
Upregulated Genes	GO_TRANSFORMING_GROWTH_FACTOR_BETA_RECEPTOR_SIGNALING_PATHWAY	5	1.4092578	0.11448598
Upregulated Genes	GO_RESPONSE_TO_METAL_ION	9	1.4085113	0.0948718
Upregulated Genes	GO_CELLULAR_COMPONENT_ASSEMBLY_INVOLVED_IN_MORPHOGENESIS	3	1.4080893	0.10940919
Upregulated Genes	GO_RESPONSE_TO_MINERALOCORTICOID	2	1.4077296	0.08172043
Upregulated Genes	GO_CARBOHYDRATE_HOMEOSTASIS	4	1.4069089	0.12641084
Upregulated Genes	GO_RESPONSE_TO_CORTICOSTERONE	2	1.4066089	0.08240534
Upregulated Genes	GO_DETECTION_OF_OTHER_ORGANISM	2	1.4026808	0.08260869
Upregulated Genes	GO_SARCOMERE_ORGANIZATION	3	1.4022985	0.116071425
Upregulated Genes	GO_ADENYLATE_CYCLASE_ACTIVATING_G_PROTEIN_COUPLED_RECEPTOR_SIGNALING_PATHW/	2	1.4012837	0.097345136
Upregulated Genes	GO_REGULATION_OF_MYELOID_LEUKOCYTE_MEDIATED_IMMUNITY	2	1.4003091	0.09663866
Upregulated Genes	GO_DETECTION_OF_VIRUS	2	1.4000591	0.09690721
Upregulated Genes	GO_T_HELPER_1_TYPE_IMMUNE_RESPONSE	2	1.3989532	0.07966457
Upregulated Genes	GO_POSITIVE_REGULATION_OF_I_KAPPAB_KINASE_NF_KAPPAB_SIGNALING	5	1.3986337	0.13839285
Upregulated Genes	GO_PROTEIN_PROCESSING	2	1.398166	0.0969163
Upregulated Genes	GO_TISSUE_MORPHOGENESIS	13	1.3976802	0.1313364
Upregulated Genes	GO_REGULATION_OF_PLASMINOGEN_ACTIVATION	2	1.3960344	0.08350731
Upregulated Genes	GO_RESPONSE_TO_OXYGEN_LEVELS	9	1.3939668	0.11165048
Upregulated Genes	GO_PLASMINOGEN_ACTIVATION	2	1.3913404	0.08
Upregulated Genes	GO_ALPHA_BETA_T_CELL_ACTIVATION_INVOLVED_IN_IMMUNE_RESPONSE	4	1.3911119	0.11378556
Upregulated Genes	GO_NEGATIVE_REGULATION_OF_RESPONSE_TO_STIMULUS	32	1.3910058	0.09972299
Upregulated Genes	GO_POSITIVE_REGULATION_OF_MULTICELLULAR_ORGANISMAL_PROCESS	43	1.3906213	0.074498564
Upregulated Genes	GO_RESPONSE_TO_CARBOHYDRATE	5	1.3899845	0.13163972
Upregulated Genes	GO_REGULATION_OF_COAGULATION	2	1.388091	0.094420604
Upregulated Genes	GO_NEGATIVE_REGULATION_OF_INTERFERON_GAMMA_PRODUCTION	2	1.388034	0.10666667
Upregulated Genes	GO_HOMOTYPIC_CELL_CELL_ADHESION	2	1.3873147	0.09586056
Upregulated Genes	GO_PLATELET_AGGREGATION	2	1.3857533	0.08939709
Upregulated Genes	GO_POSITIVE_REGULATION_OF_EPITHELIAL_TO_MESENCHYMAL_TRANSITION	4	1.3842341	0.1286031
Upregulated Genes	GO_CYCLIC_NUCLEOTIDE_MEDIATED_SIGNALING	2	1.383014	0.08370044
Upregulated Genes	GO_REGULATION_OF_CELL_ACTIVATION	14	1.3822019	0.13176471
Upregulated Genes	GO_RESPONSE_TO_BMP	3	1.3786918	0.12307692
Upregulated Genes	GO_POSITIVE_REGULATION_OF_DEPHOSPHORYLATION	2	1.3776954	0.094339624
Upregulated Genes	GO_ACTIVATION_OF_IMMUNE_RESPONSE	7	1.3746387	0.14541388
Upregulated Genes	GO_RESPONSE_TO_OXYGEN_CONTAINING_COMPOUND	40	1.3744427	0.14327486
Upregulated Genes	GO_NEGATIVE_REGULATION_OF_PEPTIDASE_ACTIVITY	2	1.3735952	0.09684685
Upregulated Genes	GO_REGULATION_OF_LYMPHOCYTE_ACTIVATION	10	1.3731579	0.13383839
Upregulated Genes	GO_REGULATION_OF_BMP_SIGNALING_PATHWAY	3	1.372485	0.14318182
Upregulated Genes	GO_DETECTION_OF_EXTERNAL_BIOTIC_STIMULUS	2	1.372473	0.11344538
Upregulated Genes	GO_REGULATION_OF_DEPHOSPHORYLATION	2	1.3724681	0.08547009
Upregulated Genes	GO_NEGATIVE_REGULATION_OF_CELLULAR_RESPONSE_TO_TRANSFORMING_GROWTH_FACTOI	2	1.3723615	0.11228813
Upregulated Genes	GO_NEGATIVE_REGULATION_OF_COAGULATION	2	1.3720304	0.10300429
Upregulated Genes	GO_NEGATIVE_REGULATION_OF_PROTEIN_CATABOLIC_PROCESS	2	1.3713055	0.10042735
Upregulated Genes	GO_NEGATIVE_REGULATION_OF_CELL_POPULATION_PROLIFERATION	10	1.3702246	0.1285347
Upregulated Genes	GO_CARDIAC_MYOFIBRIL_ASSEMBLY	2	1.3691732	0.11752137
Upregulated Genes	GO_MITOCHONDRIAL_MEMBRANE_ORGANIZATION	3	1.3675216	0.12866817
Upregulated Genes	GO_ACTIN_FILAMENT_BUNDLE_ORGANIZATION	4	1.3659749	0.15207373
Upregulated Genes	GO_ANTIGEN_PROCESSING_AND_PRESENTATION_OF_PEPTIDE_ANTIGEN	2	1.365088	0.12096774
Upregulated Genes	GO_MODULATION_BY_SYMBIONT_OF_ENTRY_INTO_HOST	1	1.3632549	0.008368201
Upregulated Genes	GO_NEGATIVE_REGULATION_OF_PROTEOLYSIS	2	1.3632064	0.10367171
Upregulated Genes	GO_NOTCH_SIGNALING_PATHWAY	2	1.3618419	0.104477614
Upregulated Genes	GO_REGULATION_OF_PROTEIN_MATURATION	2	1.3602029	0.11353712
Upregulated Genes	GO_DETECTION_OF_BIOTIC_STIMULUS	2	1.3598595	0.1012931
Upregulated Genes	GO_ACTIVIN_RECEPTOR_SIGNALING_PATHWAY	2	1.3589952	0.10745614
Upregulated Genes	GO_REGULATION_OF_PROTEIN_DEPHOSPHORYLATION	2	1.3589436	0.1010989
Upregulated Genes	GO_RESPONSE_TO_MONOSACCHARIDE	5	1.3573825	0.15521064
Upregulated Genes	GO_NEGATIVE_REGULATION_OF_CELLULAR_PROTEIN_LOCALIZATION	3	1.3573315	0.15333334
Upregulated Genes	GO_NEGATIVE_REGULATION_OF_PROTEIN_SERINE_THREONINE_KINASE_ACTIVITY	4	1.3570067	0.16
Upregulated Genes	GO_EMBRYONIC_SKELETAL_SYSTEM_DEVELOPMENT	2	1.3569481	0.10714286
Upregulated Genes	GO_POSITIVE_REGULATION_OF_INSULIN_SECRETION	1	1.3549588	0.006122449
Upregulated Genes	GO_RESPONSE_TO_INORGANIC_SUBSTANCE	15	1.3529471	0.1373057
Upregulated Genes	GO_RESPONSE_TO_INTERLEUKIN_1	2	1.3523874	0.124223605
Upregulated Genes	GO_POSITIVE_REGULATION_OF_PEPTIDE_HORMONE_SECRETION	1	1.3522542	0.005791506
Upregulated Genes	GO_ANTIGEN_PROCESSING_AND_PRESENTATION_OF_PEPTIDE_OR_POLYSACCHARIDE_ANTIGEN	2	1.3521461	0.101321585
Upregulated Genes	GO_POSITIVE_REGULATION_OF_CD4_POSITIVE_ALPHA_BETA_T_CELL_ACTIVATION	2	1.3486886	0.10169491
Upregulated Genes	GO_INTRACELLULAR_RECEPTOR_SIGNALING_PATHWAY	8	1.3479112	0.15201901

Upregulated Genes	GO_CARDIAC_MUSCLE_TISSUE_MORPHOGENESIS	1	1.3477318	0
Upregulated Genes	GO_ANTIGEN_PROCESSING_AND_PRESENTATION	2	1.3469604	0.1064257
Upregulated Genes	GO_POSITIVE_REGULATION_OF_GLIAL_CELL_DIFFERENTIATION	2	1.3462633	0.12268519
Upregulated Genes	GO_REGULATION_OF_METAL_ION_TRANSPORT	4	1.3460705	0.12727273
Upregulated Genes	GO_MYOSIN_FILAMENT_ORGANIZATION	1	1.344385	0
Upregulated Genes	GO_MUSCLE_ORGAN_MORPHOGENESIS	1	1.3416315	0
Upregulated Genes	GO_POSTSYNAPSE_ORGANIZATION	3	1.3413234	0.14252874
Upregulated Genes	GO_CELLULAR_RESPONSE_TO_NITROGEN_COMPOUND	14	1.339891	0.13902439
Upregulated Genes	GO_NEGATIVE_REGULATION_OF_CELLULAR_RESPONSE_TO_GROWTH_FACTOR_STIMULUS	3	1.3380953	0.15349887
Upregulated Genes	GO_RESPONSE_TO_PROSTAGLANDIN_E	1	1.3373648	0.010683761
Upregulated Genes	GO_HUMORAL_IMMUNE_RESPONSE_MEDIATED_BY_CIRCULATING_IMMUNOGLOBULIN	1	1.3370919	0.006147541
Upregulated Genes	GO_POSITIVE_REGULATION_OF_PROTEIN_DEPHOSPHORYLATION	2	1.3360125	0.11885246
Upregulated Genes	GO_INTERLEUKIN_4_PRODUCTION	1	1.3347046	0.004040404
Upregulated Genes	GO_REGULATION_OF_CELLULAR_RESPONSE_TO_TRANSFORMING_GROWTH_FACTOR_BETA_STI	2	1.3344913	0.12108559
Upregulated Genes	GO_CARDIAC_MUSCLE_FIBER_DEVELOPMENT	1	1.3327442	0
Upregulated Genes	GO_SKELETAL_MYOFIBRIL_ASSEMBLY	1	1.3319805	0
Upregulated Genes	GO_T_CELL_DIFFERENTIATION_INVOLVED_IN_IMMUNE_RESPONSE	4	1.331255	0.17257683
Upregulated Genes	GO_REGULATION_OF_VIRAL_ENTRY_INTO_HOST_CELL	1	1.3311118	0.007889546
Upregulated Genes	GO_NEGATIVE_REGULATION_OF_SECRETION	2	1.3307675	0.118303575
Upregulated Genes	GO_MUSCLE_FILAMENT_SLIDING	1	1.3307203	0
Upregulated Genes	GO_MUSCLE_TISSUE_DEVELOPMENT	6	1.3303707	0.15315315
Upregulated Genes	GO_REGULATION_OF_EOSINOPHIL_MIGRATION	1	1.3292044	0.01984127
Upregulated Genes	GO_EOSINOPHIL_MIGRATION	1	1.3288069	0.016
Upregulated Genes	GO_NEGATIVE_REGULATION_OF_PEPTIDE_SECRETION	2	1.3286471	0.11504425
Upregulated Genes	GO_REGULATION_OF_INFLAMMATORY_RESPONSE_TO_ANTIGENIC_STIMULUS	1	1.3283705	0.00998004
Upregulated Genes	GO_POSITIVE_REGULATION_OF_IMMUNE_RESPONSE	14	1.3275563	0.15776698
Upregulated Genes	GO_SKELETAL_MUSCLE_THIN_FILAMENT_ASSEMBLY	1	1.3272065	0
Upregulated Genes	GO_PYROPTOSIS	1	1.3257443	0.03305785
Upregulated Genes	GO_NEGATIVE_REGULATION_OF_IMMUNE_RESPONSE	1	1.3246688	0.01183432
Upregulated Genes	GO_CELLULAR_ANION_HOMEOSTASIS	1	1.3240367	0.021611001
Upregulated Genes	GO_MONOVALENT_INORGANIC_ANION_HOMEOSTASIS	1	1.3239552	0.014736842
Upregulated Genes	GO_CELLULAR_RESPONSE_TO_PROSTAGLANDIN_E_STIMULUS	1	1.3232768	0.013539651
Upregulated Genes	GO_CARDIAC_MUSCLE_TISSUE_DEVELOPMENT	5	1.3227288	0.15647922
Upregulated Genes	GO_POSITIVE_REGULATION_OF_HORMONE_SECRETION	1	1.3220905	0.006060606
Upregulated Genes	GO_NEGATIVE_REGULATION_OF_CELLULAR_EXTRAVASATION	1	1.3219292	0.01532567
Upregulated Genes	GO_POSITIVE_REGULATION_OF_INSULIN_SECRETION_INVOLVED_IN_CELLULAR_RESPONSE_TO_	1	1.3219016	0.005905512
Upregulated Genes	GO_IMMUNOGLOBULIN_PRODUCTION_INVOLVED_IN_IMMUNOGLOBULIN_MEDIATED_IMMUN	4	1.3206254	0.1825
Upregulated Genes	GO_NEGATIVE_REGULATION_OF_INFLAMMATORY_RESPONSE_TO_ANTIGENIC_STIMULUS	1	1.318915	0.008298756
Upregulated Genes	GO_POSITIVE_REGULATION_OF_VASCULATURE_DEVELOPMENT	4	1.3185958	0.15764706
Upregulated Genes	GO_RETINOIC_ACID_CATABOLIC_PROCESS	1	1.3184708	0.032388665
Upregulated Genes	GO_SODIUM_DEPENDENT_PHOSPHATE_TRANSPORT	1	1.3178838	0.041493777
Upregulated Genes	GO_REGULATION_OF_ASTROCYTE_DIFFERENTIATION	2	1.3176403	0.15257733
Upregulated Genes	GO_POSITIVE_REGULATION_BY_SYMBIONT_OF_ENTRY_INTO_HOST	1	1.3168527	0.006122449
Upregulated Genes	GO_REGULATION_OF_CELLULAR_EXTRAVASATION	1	1.3159814	0.019305019
Upregulated Genes	GO_TERPENOID_CATABOLIC_PROCESS	1	1.3154149	0.043052837
Upregulated Genes	GO_INSULIN_SECRETION_INVOLVED_IN_CELLULAR_RESPONSE_TO_GLUCOSE_STIMULUS	1	1.3153269	0.006355932
Upregulated Genes	GO_REGULATION_OF_T_CELL_DIFFERENTIATION	5	1.3143426	0.18468468
Upregulated Genes	GO_ANION_HOMEOSTASIS	1	1.3143039	0.028248588
Upregulated Genes	GO_PROTEIN_ACTIVATION_CASCADE	1	1.312463	0.045360826
Upregulated Genes	GO_CYTOKINE_PRODUCTION_INVOLVED_IN_IMMUNE_RESPONSE	2	1.3122574	0.12916666
Upregulated Genes	GO_ASTROCYTE_DIFFERENTIATION	2	1.3114455	0.15611814
Upregulated Genes	GO_REGULATION_OF_EXTRACELLULAR_MATRIX_ORGANIZATION	2	1.3111706	0.13953489
Upregulated Genes	GO_NECROTIC_CELL_DEATH	2	1.3108793	0.17173913
Upregulated Genes	GO_NEGATIVE_REGULATION_OF_LEUKOCYTE_MIGRATION	1	1.3100868	0.020920502
Upregulated Genes	GO_CELLULAR_DIVALENT_INORGANIC_ANION_HOMEOSTASIS	1	1.3093113	0.03125
Upregulated Genes	GO_MHC_PROTEIN_COMPLEX_ASSEMBLY	1	1.308904	0.012244898
Upregulated Genes	GO_CELL_CELL_ADHESION_VIA_PLASMA_MEMBRANE_ADHESION_MOLECULES	6	1.3088878	0.18409091
Upregulated Genes	GO_PHOSPHATE_ION_TRANSPORT	1	1.307905	0.027027028
Upregulated Genes	GO_POSITIVE_REGULATION_OF_EXTRACELLULAR_MATRIX_DISASSEMBLY	2	1.3061434	0.16317992
Upregulated Genes	GO_HOMOPHILIC_CELL_ADHESION_VIA_PLASMA_MEMBRANE_ADHESION_MOLECULES	5	1.3059275	0.18594104
Upregulated Genes	GO_HOMEOSTATIC_PROCESS	38	1.3054726	0.1396648
Upregulated Genes	GO_CELLULAR_EXTRAVASATION	1	1.3051344	0.028513238
Upregulated Genes	GO_ODONTOGENESIS	5	1.304652	0.1748879
Upregulated Genes	GO_REGULATION_OF_INTEGRIN_ACTIVATION	2	1.3043358	0.15221988
Upregulated Genes	GO_T_HELPER_17_TYPE_IMMUNE_RESPONSE	2	1.3036917	0.15833333
Upregulated Genes	GO_COLLAGEN_METABOLIC_PROCESS	2	1.3035022	0.14750542
Upregulated Genes	GO_ISOPRENOID_CATABOLIC_PROCESS	1	1.302815	0.05284553
Upregulated Genes	GO_PROGRAMMED_NECROTIC_CELL_DEATH	2	1.3026979	0.16488223
Upregulated Genes	GO_CDP_DIACYLGLYCEROL_METABOLIC_PROCESS	1	1.3018645	0.048582997
Upregulated Genes	GO_DIVALENT_INORGANIC_ANION_HOMEOSTASIS	1	1.3009669	0.035490606

Upregulated Genes	GO_BLOOD_COAGULATION_INTRINSIC_PATHWAY	1	1.3008922	0.046747968
Upregulated Genes	GO_NEGATIVE_REGULATION_OF_TRANSPORT	9	1.2997915	0.16435185
Upregulated Genes	GO_FIBRINOLYSIS	1	1.2992647	0.0375
Upregulated Genes	GO_REGULATION_OF_EPITHELIAL_CELL_APOPTOTIC_PROCESS	2	1.2984117	0.15517241
Upregulated Genes	GO_MOVEMENT_IN_HOST_ENVIRONMENT	3	1.2982609	0.1750547
Upregulated Genes	GO_NEGATIVE_REGULATION_OF_CYTOKINE_SECRETION	2	1.2982526	0.15932915
Upregulated Genes	GO_ISOPRENOID_METABOLIC_PROCESS	1	1.2981772	0.036821704
Upregulated Genes	GO_CARDIAC_CELL_DEVELOPMENT	3	1.2979482	0.19392523
Upregulated Genes	GO_REGULATION_OF_ION_TRANSMEMBRANE_TRANSPORT	4	1.296322	0.19058296
Upregulated Genes	GO_B_CELL_MEDIATED_IMMUNITY	4	1.2958347	0.15461347
Upregulated Genes	GO_INTEGRIN_ACTIVATION	2	1.2954918	0.14959016
Upregulated Genes	GO_GRANULOCYTE_DIFFERENTIATION	3	1.2942282	0.2
Upregulated Genes	GO_I_KAPPAB_KINASE_NF_KAPPAB_SIGNALING	7	1.293988	0.1847575
Upregulated Genes	GO_RETINOIC_ACID_METABOLIC_PROCESS	1	1.2931414	0.054435484
Upregulated Genes	GO_RESPONSE_TO_DOPAMINE	1	1.2929605	0.09072978
Upregulated Genes	GO_TERPENOID_METABOLIC_PROCESS	1	1.2905157	0.049079753
Upregulated Genes	GO_REGULATORY_T_CELL_DIFFERENTIATION	2	1.2884786	0.175
Upregulated Genes	GO_REGULATION_OF_CELL_SUBSTRATE_ADHESION	4	1.2881042	0.19642857
Upregulated Genes	GO_HYDROGEN_SULFIDE_METABOLIC_PROCESS	1	1.2868574	0.05928854
Upregulated Genes	GO_REGULATION_OF_CYTOKINE_PRODUCTION_INVOLVED_IN_IMMUNE_RESPONSE	2	1.2846195	0.14
Upregulated Genes	GO_SULFUR_AMINO_ACID_METABOLIC_PROCESS	1	1.2842499	0.06097561
Upregulated Genes	GO_REGULATION_OF_CELLULAR_COMPONENT_MOVEMENT	25	1.2838001	0.16795866
Upregulated Genes	GO_POSITIVE_REGULATION_OF_CATABOLIC_PROCESS	10	1.2833178	0.18554217
Upregulated Genes	GO_RESPONSE_TO_LIPID	26	1.282672	0.16842106
Upregulated Genes	GO_COLLAGEN_BIOSYNTHETIC_PROCESS	2	1.2823367	0.15584415
Upregulated Genes	GO_REGULATION_OF_EXTRACELLULAR_MATRIX_DISASSEMBLY	2	1.282306	0.18103448
Upregulated Genes	GO_INFLAMMATORY_RESPONSE_TO_ANTIGENIC_STIMULUS	3	1.2822598	0.1780822
Upregulated Genes	GO_HOMOCYSTEINE_METABOLIC_PROCESS	1	1.2819939	0.08695652
Upregulated Genes	GO_CARDIOCYTE_DIFFERENTIATION	3	1.2818866	0.18082789
Upregulated Genes	GO_WNT_SIGNALING_PATHWAY_CALCIUM_MODULATING_PATHWAY	1	1.2818195	0.08266129
Upregulated Genes	GO_POSITIVE_REGULATION_OF_CYTOKINE_PRODUCTION_INVOLVED_IN_IMMUNE_RESPONSE	2	1.2808499	0.18125
Upregulated Genes	GO_NERVE_DEVELOPMENT	2	1.2808167	0.14935064
Upregulated Genes	GO_SULFUR_AMINO_ACID_BIOSYNTHETIC_PROCESS	1	1.2801838	0.084337346
Upregulated Genes	GO_MONOCARBOXYLIC_ACID_CATABOLIC_PROCESS	2	1.2800264	0.17094018
Upregulated Genes	GO_MUSCLE_CELL_DEVELOPMENT	5	1.2798951	0.20092379
Upregulated Genes	GO_NEGATIVE_REGULATION_OF_RETINOIC_ACID_RECEPTOR_SIGNALING_PATHWAY	2	1.2793015	0.1788793
Upregulated Genes	GO_ORGANIC_HYDROXY_COMPOUND_TRANSPORT	1	1.2792714	0.105367795
Upregulated Genes	GO_REGULATION_OF_RETINOIC_ACID_RECEPTOR_SIGNALING_PATHWAY	2	1.2790698	0.17190775
Upregulated Genes	GO_POSITIVE_REGULATION_OF_SMOOTH_MUSCLE_CELL_PROLIFERATION	2	1.2777436	0.17083333
Upregulated Genes	GO_ESTABLISHMENT_OF_ENDOTHELIAL_BARRIER	2	1.2762775	0.19438446
Upregulated Genes	GO_REGULATION_OF_RESPONSE_TO_CYTOKINE_STIMULUS	6	1.2757657	0.21307506
Upregulated Genes	GO_MUSCLE_CELL_DIFFERENTIATION	8	1.2750603	0.19689119
Upregulated Genes	GO_CYSTEINE_METABOLIC_PROCESS	1	1.2733009	0.09836066
Upregulated Genes	GO_CELLULAR_RESPONSE_TO_OXYGEN_GLUCOSE_DEPRIVATION	1	1.2732915	0.11462451
Upregulated Genes	GO_POSITIVE_REGULATION_OF_HEART_CONTRACTION	1	1.2732412	0.10980392
Upregulated Genes	GO_REGULATION_OF_SYSTEMIC_ARTERIAL_BLOOD_PRESSURE_BY_HORMONE	1	1.2727194	0.090361446
Upregulated Genes	GO_DOPAMINE_RECEPTOR_SIGNALING_PATHWAY	1	1.2714887	0.07543521
Upregulated Genes	GO_HOMOSERINE_METABOLIC_PROCESS	1	1.2713917	0.064449064
Upregulated Genes	GO_REGULATION_OF_SYSTEMIC_ARTERIAL_BLOOD_PRESSURE	1	1.271348	0.09393346
Upregulated Genes	GO_FATTY_ACID_DERIVATIVE_BIOSYNTHETIC_PROCESS	2	1.2708296	0.1594203
Upregulated Genes	GO_INOSITOL_PHOSPHATE_MEDIATED_SIGNALING	1	1.2705147	0.09664694
Upregulated Genes	GO_APOPTOTIC_MITOCHONDRIAL_CHANGES	3	1.2703935	0.1632653
Upregulated Genes	GO_DELAMINATION	1	1.2691919	0.11851852
Upregulated Genes	GO_EXCRETION	1	1.2691771	0.117870726
Upregulated Genes	GO_INTERACTION_WITH_HOST	3	1.2688429	0.19908467
Upregulated Genes	GO_UREA_TRANSPORT	1	1.2686656	0.096
Upregulated Genes	GO_MAINTENANCE_OF_BLOOD_BRAIN_BARRIER	2	1.2686	0.17248908
Upregulated Genes	GO_POSITIVE_REGULATION_OF_OSSIFICATION	2	1.267949	0.15966387
Upregulated Genes	GO_CALCIUM_MEDIATED_SIGNALING	1	1.2671362	0.10344828
Upregulated Genes	GO_POLYOL_TRANSPORT	1	1.2670599	0.13279678
Upregulated Genes	GO_VITAMIN_CATABOLIC_PROCESS	2	1.2670412	0.17372881
Upregulated Genes	GO_SMOOTH_MUSCLE_CONTRACTION	1	1.2664155	0.11023622
Upregulated Genes	GO_HYDROGEN_SULFIDE_BIOSYNTHETIC_PROCESS	1	1.2658796	0.07952286
Upregulated Genes	GO_ADAPTIVE_IMMUNE_RESPONSE_BASED_ON_SOMATIC_RECOMBINATION_OF_IMMUNE_RE	8	1.2658666	0.2028302
Upregulated Genes	GO_REGULATION_OF_SYSTEMIC_ARTERIAL_BLOOD_PRESSURE_MEDIATED_BY_A_CHEMICAL_SI	1	1.2650777	0.109561756
Upregulated Genes	GO_WATER_TRANSPORT	1	1.2646968	0.10843374
Upregulated Genes	GO_RENAL_WATER_TRANSPORT	1	1.2644293	0.12068965
Upregulated Genes	GO_MOTOR_NEURON_AXON_GUIDANCE	2	1.2634522	0.17371938
Upregulated Genes	GO_PROSTANOID_BIOSYNTHETIC_PROCESS	1	1.2629162	0.11044177
Upregulated Genes	GO_POSITIVE_REGULATION_OF_BLOOD_CIRCULATION	1	1.2626176	0.09306931

Upregulated Genes	GO_ENDOCRINE_PROCESS	1	1.2617831	0.11067961
Upregulated Genes	GO_LEUKOCYTE_CHEMOTAXIS	6	1.2617201	0.21113689
Upregulated Genes	GO_FAT_SOLUBLE_VITAMIN_METABOLIC_PROCESS	2	1.2615677	0.19753087
Upregulated Genes	GO_ALPHA_BETA_T_CELL_PROLIFERATION	2	1.2613761	0.18683651
Upregulated Genes	GO_ONE_CARBON_COMPOUND_TRANSPORT	1	1.261251	0.08722109
Upregulated Genes	GO_RENAL_ABSORPTION	1	1.2600222	0.11691023
Upregulated Genes	GO_REGULATION_OF_THE_FORCE_OF_HEART_CONTRACTION	1	1.2600147	0.1002004
Upregulated Genes	GO_REGULATION_OF_THE_FORCE_OF_HEART_CONTRACTION_BY_CHEMICAL_SIGNAL	1	1.2600051	0.12601626
Upregulated Genes	GO_NEGATIVE_REGULATION_OF_BLOOD_VESSEL_DIAMETER	1	1.2599733	0.10309278
Upregulated Genes	GO_REGULATION_OF_BLOOD_PRESSURE	1	1.259881	0.10139165
Upregulated Genes	GO_MDA_5_SIGNALING_PATHWAY	2	1.2598139	0.20353982
Upregulated Genes	GO_POSITIVE_REGULATION_OF_ION_TRANSPORT	1	1.259343	0.11491936
Upregulated Genes	GO_FLUID_TRANSPORT	1	1.2577835	0.1
Upregulated Genes	GO_POSITIVE_REGULATION_OF_HEART_RATE	1	1.2577635	0.14502165
Upregulated Genes	GO_CARDIAC_MUSCLE_CELL_DIFFERENTIATION	3	1.257239	0.20096852
Upregulated Genes	GO_CALCIUM_ION_IMPORT	1	1.2563839	0.11067194
Upregulated Genes	GO_REGULATION_OF_TUBE_SIZE	1	1.2563493	0.121703856
Upregulated Genes	GO_POSITIVE_REGULATION_OF_AXONOGENESIS	2	1.2555795	0.18238993
Upregulated Genes	GO_TONIC_SMOOTH_MUSCLE_CONTRACTION	1	1.2551951	0.10831721
Upregulated Genes	GO_CELLULAR_RESPONSE_TO_ENDOGENOUS_STIMULUS	27	1.255105	0.18941504
Upregulated Genes	GO_POSITIVE_REGULATION_OF_EXTRACELLULAR_MATRIX_ORGANIZATION	2	1.2550554	0.18924731
Upregulated Genes	GO_ENDOCARDIAL_CUSHION_DEVELOPMENT	2	1.2537093	0.18709677
Upregulated Genes	GO_MATERNAL_PLACENTA_DEVELOPMENT	1	1.2536227	0.111561865
Upregulated Genes	GO_REGULATION_OF_ANTIGEN_RECEPTOR_MEDIATED_SIGNALING_PATHWAY	2	1.2524784	0.2030905
Upregulated Genes	GO_RESPONSE_TO_OXYGEN_GLUCOSE_DEPRIVATION	1	1.2522727	0.11468813
Upregulated Genes	GO_PHASIC_SMOOTH_MUSCLE_CONTRACTION	1	1.2522559	0.1257485
Upregulated Genes	GO_REGULATION_OF_SYSTEMIC_ARTERIAL_BLOOD_PRESSURE_BY_ENDOTHELIN	1	1.2519063	0.109375
Upregulated Genes	GO_RELEASE_OF_CYTOCHROME_C_FROM_MITOCHONDRIA	3	1.2518328	0.2033097
Upregulated Genes	GO_REGULATION_OF_WOUND_HEALING	3	1.251709	0.18859649
Upregulated Genes	GO_REGULATION_OF_VASOCONSTRICTION	1	1.2512802	0.12877263
Upregulated Genes	GO_ARTERY_SMOOTH_MUSCLE_CONTRACTION	1	1.2510376	0.11914894
Upregulated Genes	GO_REGULATION_OF_RESPONSE_TO_WOUNDING	3	1.2509667	0.1977528
Upregulated Genes	GO_ICOSANOID_BIOSYNTHETIC_PROCESS	1	1.2509395	0.11597374
Upregulated Genes	GO_REGULATION_OF_EXCRETION	1	1.2503881	0.15071283
Upregulated Genes	GO_POSITIVE_REGULATION_OF_SMOOTH_MUSCLE_CONTRACTION	1	1.2501562	0.11198428
Upregulated Genes	GO_ENDOTHELIAL_CELL_MORPHOGENESIS	1	1.2493843	0.12984496
Upregulated Genes	GO_INFLAMMATORY_CELL_APOPTOTIC_PROCESS	2	1.2482127	0.20390455
Upregulated Genes	GO_VASCULAR_ASSOCIATED_SMOOTH_MUSCLE_CONTRACTION	1	1.247621	0.112
Upregulated Genes	GO_STRIATED_MUSCLE_CELL_DIFFERENTIATION	8	1.2475948	0.2271605
Upregulated Genes	GO_NON_CANONICAL_WNT_SIGNALING_PATHWAY	3	1.2475154	0.21956521
Upregulated Genes	GO_POSITIVE_REGULATION_OF_PEPTIDYL_TYROSINE_PHOSPHORYLATION	3	1.247461	0.19318181
Upregulated Genes	GO_POSITIVE_REGULATION_OF_HAIR_CYCLE	1	1.2471923	0.12648222
Upregulated Genes	GO_RETINOIC_ACID_RECEPTOR_SIGNALING_PATHWAY	2	1.2471184	0.1959596
Upregulated Genes	GO_GLYCEROL_TRANSPORT	1	1.2471135	0.1167002
Upregulated Genes	GO_CELL_JUNCTION_ASSEMBLY	7	1.2469474	0.22222222
Upregulated Genes	GO_REGULATION_OF_HAIR_FOLLICLE_DEVELOPMENT	1	1.246286	0.1125
Upregulated Genes	GO_POSITIVE_REGULATION_OF_MUSCLE_CONTRACTION	1	1.2453963	0.13179916
Upregulated Genes	GO_EPIDERMIS_MORPHOGENESIS	1	1.2452263	0.16527197
Upregulated Genes	GO_RENAL_WATER_HOMEOSTASIS	1	1.2449474	0.124497995
Upregulated Genes	GO_SKIN_EPIDERMIS_DEVELOPMENT	1	1.2439582	0.15445544
Upregulated Genes	GO_POSITIVE_REGULATION_OF_CELL_MORPHOGENESIS_INVOLVED_IN_DIFFERENTIATION	2	1.243552	0.1838843
Upregulated Genes	GO_DECIDUALIZATION	1	1.2435393	0.118236475
Upregulated Genes	GO_BONE_MORPHOGENESIS	4	1.243324	0.21132898
Upregulated Genes	GO_REGULATION_OF_ACTIVIN_RECEPTOR_SIGNALING_PATHWAY	1	1.2431233	0.13740458
Upregulated Genes	GO_VIRAL_LIFE_CYCLE	5	1.2428073	0.23690204
Upregulated Genes	GO_NEGATIVE_REGULATION_OF_KERATINOCYTE_DIFFERENTIATION	1	1.242032	0.14341846
Upregulated Genes	GO_PURINE_CONTAINING_COMPOUND_SALVAGE	1	1.2419538	0.14401622
Upregulated Genes	GO_REGULATION_OF_SMOOTH_MUSCLE_CONTRACTION	1	1.2419533	0.1124031
Upregulated Genes	GO_CELLULAR_RESPONSE_TO_CAMP	1	1.2417313	0.12929294
Upregulated Genes	GO_REGULATION_OF_CALCIUM_ION_IMPORT	1	1.2416848	0.14851485
Upregulated Genes	GO_CHONDROCYTE_PROLIFERATION	1	1.2414613	0.15866388
Upregulated Genes	GO_POSITIVE_REGULATION_OF_CALCIUM_ION_IMPORT	1	1.2413064	0.13250518
Upregulated Genes	GO_REGULATION_OF_CELL_KILLING	1	1.2413038	0.1417004
Upregulated Genes	GO_POSITIVE_REGULATION_OF_LEUKOCYTE_CHEMOTAXIS	4	1.241021	0.21300448
Upregulated Genes	GO_CARTILAGE_MORPHOGENESIS	1	1.2398183	0.11632653
Upregulated Genes	GO_REGULATION_OF_HEART_RATE	1	1.2391063	0.12795275
Upregulated Genes	GO_LUNG_ALVEOLUS_DEVELOPMENT	1	1.238909	0.13987474
Upregulated Genes	GO_POSITIVE_REGULATION_OF_LEUKOCYTE_MIGRATION	4	1.2389022	0.22365591
Upregulated Genes	GO_NEGATIVE_REGULATION_OF_RESPONSE_TO_WOUNDING	3	1.2388711	0.21315193
Upregulated Genes	GO_NEGATIVE_REGULATION_OF_WOUND_HEALING	3	1.2376243	0.20177384

Upregulated Genes	GO_POSITIVE_REGULATION_OF_CALCIUM_ION_TRANSPORT	1	1.2374761	0.14143427
Upregulated Genes	GO_CELLULAR_METABOLIC_COMPOUND_SALVAGE	1	1.2371116	0.15587045
Upregulated Genes	GO_MITOTIC_CHROMOSOME_CONDENSATION	4	1.2370799	0.24541284
Upregulated Genes	GO_NATURAL_KILLER_CELL_MEDIATED_IMMUNITY	1	1.2364837	0.14509805
Upregulated Genes	GO_REGULATION_OF_MEMBRANE_DEPOLARIZATION	1	1.2358831	0.16135459
Upregulated Genes	GO_REGULATION_OF_TRANSFERASE_ACTIVITY	31	1.2348485	0.1891892
Upregulated Genes	GO_REGULATION_OF_HIPPO_SIGNALING	1	1.234424	0.14910537
Upregulated Genes	GO_REGULATION_OF_PEPTIDYL_TYROSINE_PHOSPHORYLATION	3	1.233496	0.21212122
Upregulated Genes	GO_POSITIVE_REGULATION_OF_WNT_SIGNALING_PATHWAY	3	1.232859	0.19870411
Upregulated Genes	GO_REGULATION_OF_RENAL_SYSTEM_PROCESS	1	1.2313197	0.1495327
Upregulated Genes	GO_NEGATIVE_REGULATION_OF_ACTIVIN_RECEPTOR_SIGNALING_PATHWAY	1	1.230495	0.14705883
Upregulated Genes	GO_POSITIVE_REGULATION_OF_ORGANELLE_ASSEMBLY	3	1.2302697	0.22685185
Upregulated Genes	GO_REGULATION_OF_LEUKOCYTE_MEDIATED_CYTOTOXICITY	1	1.2291602	0.14078675
Upregulated Genes	GO_REGULATION_OF_ANION_TRANSPORT	1	1.2281629	0.15506959
Upregulated Genes	GO_POSITIVE_REGULATION_OF_NATURAL_KILLER_CELL_MEDIATED_IMMUNITY	1	1.225803	0.1694215
Upregulated Genes	GO_REGULATION_OF_BONE_MINERALIZATION	1	1.2245402	0.15966387
Upregulated Genes	GO_POSITIVE_REGULATION_OF_CHEMOTAXIS	4	1.2238017	0.2347826
Upregulated Genes	GO_CARTILAGE_DEVELOPMENT_INVOLVED_IN_ENDOCHONDRAL_BONE_MORPHOGENESIS	4	1.2232388	0.2448037
Upregulated Genes	GO_REGULATION_OF_ALPHA_BETA_T_CELL_DIFFERENTIATION	4	1.2231054	0.21445782
Upregulated Genes	GO_LYMPHOCYTE_MEDIATED_IMMUNITY	8	1.2213229	0.24770643
Upregulated Genes	GO_POSITIVE_REGULATION_OF_T_HELPER_17_TYPE_IMMUNE_RESPONSE	1	1.2213035	0.20309478
Upregulated Genes	GO_POSITIVE_REGULATION_OF_EPIDERMIS_DEVELOPMENT	1	1.2206169	0.17373738
Upregulated Genes	GO_NEGATIVE_REGULATION_OF_CALCIUM_ION_TRANSPORT	1	1.2202202	0.17263158
Upregulated Genes	GO_POSITIVE_REGULATION_OF_T_HELPER_17_CELL_DIFFERENTIATION	1	1.2200471	0.15568863
Upregulated Genes	GO_POSITIVE_REGULATION_OF_CELL_KILLING	1	1.219997	0.16969697
Upregulated Genes	GO_POSTSYNAPTIC_CYTOSKELETON_ORGANIZATION	1	1.2199911	0.1588785
Upregulated Genes	GO_REGULATION_OF_SKELETAL_MUSCLE_ACETYLCHOLINE_GATED_CHANNEL_CLUSTERING	1	1.2185935	0.17087379
Upregulated Genes	GO_REGULATION_OF_NECROTIC_CELL_DEATH	1	1.2184719	0.17399618
Upregulated Genes	GO_REGULATION_OF_NATURAL_KILLER_CELL_MEDIATED_IMMUNITY	1	1.2179182	0.192
Upregulated Genes	GO_ORGANIC_ACID_BIOSYNTHETIC_PROCESS	9	1.2174747	0.23774509
Upregulated Genes	GO_POSITIVE_REGULATION_OF_NATURAL_KILLER_CELL_MEDIATED_CYTOTOXICITY	1	1.2172352	0.15725806
Upregulated Genes	GO_REGULATION_OF_MITOCHONDRIAL_OUTER_MEMBRANE_PERMEABILIZATION_INVOLVED_I	1	1.2160748	0.2003891
Upregulated Genes	GO_CELLULAR_PROTEIN_CONTAINING_COMPLEX_ASSEMBLY	31	1.2150453	0.2334294
Upregulated Genes	GO_AXONEME_ASSEMBLY	1	1.2133397	0.18039216
Upregulated Genes	GO_POSITIVE_REGULATION_OF_CD4_POSITIVE_ALPHA_BETA_T_CELL_DIFFERENTIATION	1	1.2126852	0.17219917
Upregulated Genes	GO_REGULATION_OF_CD4_POSITIVE_ALPHA_BETA_T_CELL_ACTIVATION	4	1.2121923	0.25119618
Upregulated Genes	GO_POSITIVE_REGULATION_OF_BMP_SIGNALING_PATHWAY	1	1.2119412	0.18857142
Upregulated Genes	GO_DIVALENT_INORGANIC_CATION_TRANSPORT	3	1.2114873	0.24498886
Upregulated Genes	GO_REGULATION_OF_T_HELPER_17_TYPE_IMMUNE_RESPONSE	1	1.2110455	0.17234042
Upregulated Genes	GO_REGULATION_OF_RECEPTOR_CLUSTERING	1	1.2108443	0.17777778
Upregulated Genes	GO_REGULATION_OF_OLIGODENDROCYTE_DIFFERENTIATION	1	1.2106966	0.16367266
Upregulated Genes	GO_REGULATION_OF_PHOSPHATIDYLINOSITOL_3_KINASE_SIGNALING	3	1.2106125	0.2080537
Upregulated Genes	GO_INNER_DYNEIN_ARM_ASSEMBLY	1	1.2105837	0.18495935
Upregulated Genes	GO_IMMUNOGLOBULIN_PRODUCTION	6	1.2104752	0.24264705
Upregulated Genes	GO_SPERM_AXONEME_ASSEMBLY	1	1.2102168	0.16666667
Upregulated Genes	GO_REGULATION_OF_POSTSYNAPTIC_MEMBRANE_ORGANIZATION	1	1.2100934	0.16872428
Upregulated Genes	GO_POSITIVE_REGULATION_OF_IMMUNE_EFFECTOR_PROCESS	7	1.209661	0.24941725
Upregulated Genes	GO_NEGATIVE_REGULATION_OF_ION_TRANSPORT	3	1.2095469	0.22453703
Upregulated Genes	GO_REGULATION_OF_NEURAL_PRECURSOR_CELL_PROLIFERATION	1	1.2090424	0.18813907
Upregulated Genes	GO_CELL_RECOGNITION	1	1.2084315	0.16938776
Upregulated Genes	GO_POSITIVE_REGULATION_OF_BONE_MINERALIZATION	1	1.2077565	0.19791667
Upregulated Genes	GO_SKELETAL_MUSCLE_ACETYLCHOLINE_GATED_CHANNEL_CLUSTERING	1	1.2070282	0.18326694
Upregulated Genes	GO_PROTEIN_LOCALIZATION_TO_BICELLULAR_TIGHT_JUNCTION	1	1.2064598	0.206
Upregulated Genes	GO_EXTRACELLULAR_TRANSPORT	1	1.2064489	0.17693837
Upregulated Genes	GO_NEGATIVE_REGULATION_OF_CELL_SUBSTRATE_ADHESION	3	1.2064213	0.23820224
Upregulated Genes	GO_ENDOCHONDRAL_BONE_MORPHOGENESIS	4	1.2052605	0.24458875
Upregulated Genes	GO_SMALL_MOLECULE_CATABOLIC_PROCESS	5	1.2048063	0.2589928
Upregulated Genes	GO_REGULATION_OF_PROGRAMMED_NECROTIC_CELL_DEATH	1	1.2047889	0.19387755
Upregulated Genes	GO_REGULATION_OF_NEUROMUSCULAR_JUNCTION_DEVELOPMENT	1	1.204584	0.19038077
Upregulated Genes	GO_MACROPHAGE_PROLIFERATION	1	1.2044189	0.19037656
Upregulated Genes	GO_PROTEIN_CONTAINING_COMPLEX_SUBUNIT_ORGANIZATION	49	1.2043567	0.19382022
Upregulated Genes	GO_POSITIVE_REGULATION_OF_BIOMINERALIZATION	1	1.2026713	0.20238096
Upregulated Genes	GO_CELL_CELL_ADHESION_MEDIATED_BY_CADHERIN	1	1.2025994	0.19315895
Upregulated Genes	GO_POSTSYNAPTIC_MEMBRANE_ORGANIZATION	1	1.202348	0.19132149
Upregulated Genes	GO_REGULATION_OF_LEUKOCYTE_CHEMOTAXIS	4	1.201825	0.23529412
Upregulated Genes	GO_TELOMERIC_LOOP_DISASSEMBLY	3	1.2017043	0.24263039
Upregulated Genes	GO_TRANSMEMBRANE_RECEPTOR_PROTEIN_SERINE_THREONINE_KINASE_SIGNALING_PATHW/	8	1.201341	0.2511737
Upregulated Genes	GO_POSITIVE_REGULATION_OF_MACROPHAGE_CHEMOTAXIS	1	1.2012953	0.19161677
Upregulated Genes	GO_CELLULAR_RESPONSE_TO_ORGANIC_CYCLIC_COMPOUND	15	1.2012386	0.26419753
Upregulated Genes	GO_LOCALIZATION_WITHIN_MEMBRANE	1	1.2008246	0.18145162

Upregulated Genes	GO_REGULATION_OF_T_HELPER_CELL_DIFFERENTIATION	1	1.200772	0.1970339
Upregulated Genes	GO_HEART_DEVELOPMENT	10	1.2006155	0.2691358
Upregulated Genes	GO_REGULATION_OF_PROTEIN_TYROSINE_KINASE_ACTIVITY	1	1.2005972	0.19076306
Upregulated Genes	GO_NEGATIVE_REGULATION_OF_PHOSPHATIDYLINOSITOL_3_KINASE_SIGNALING	3	1.1995329	0.2473573
Upregulated Genes	GO_REGULATION_OF_MACROPHAGE_PROLIFERATION	1	1.1992564	0.18711019
Upregulated Genes	GO_REGULATION_OF_MITOCHONDRIAL_MEMBRANE_POTENTIAL	1	1.19907	0.20081136
Upregulated Genes	GO_ADAPTIVE_IMMUNE_RESPONSE	8	1.1989918	0.24825986
Upregulated Genes	GO_AXONEMAL_DYNEIN_COMPLEX_ASSEMBLY	1	1.1988742	0.22417153
Upregulated Genes	GO_CALCIUM_DEPENDENT_CELL_CELL_ADHESION_VIA_PLASMA_MEMBRANE_CELL_ADHESION	1	1.1988701	0.20250522
Upregulated Genes	GO_REGULATION_OF_MACROPHAGE_DIFFERENTIATION	1	1.1986712	0.21212122
Upregulated Genes	GO_REGULATION_OF_MEMBRANE_POTENTIAL	3	1.1986464	0.25221238
Upregulated Genes	GO_REGULATION_OF_POSTSYNAPTIC_CYTOSOLIC_CALCIUM_ION_CONCENTRATION	1	1.198613	0.19038077
Upregulated Genes	GO_POSITIVE_REGULATION_OF_T_HELPER_CELL_DIFFERENTIATION	1	1.1985096	0.20083682
Upregulated Genes	GO_POSITIVE_REGULATION_OF_ENDOTHELIAL_CELL_DEVELOPMENT	1	1.1982915	0.20703125
Upregulated Genes	GO_MICROTUBULE_BUNDLE_FORMATION	1	1.197868	0.17773438
Upregulated Genes	GO_REGULATION_OF_TRANS_SYNAPTIC_SIGNALING	3	1.1976364	0.2290749
Upregulated Genes	GO_POSITIVE_REGULATION_OF_NEURAL_PRECURSOR_CELL_PROLIFERATION	1	1.1972673	0.19624217
Upregulated Genes	GO_NEGATIVE_REGULATION_OF_ENDOTHELIAL_CELL_APOPTOTIC_PROCESS	1	1.19705	0.20318726
Upregulated Genes	GO_POSITIVE_REGULATION_OF_OLIGODENDROCYTE_DIFFERENTIATION	1	1.1967633	0.20816326
Upregulated Genes	GO_ENTRY_INTO_HOST	2	1.1967162	0.24590164
Upregulated Genes	GO_NEGATIVE_REGULATION_OF_MITOCHONDRION_ORGANIZATION	1	1.1953934	0.20866142
Upregulated Genes	GO_REGULATION_OF_PROTEIN_CATABOLIC_PROCESS	6	1.1946585	0.25675675
Upregulated Genes	GO_NEGATIVE_REGULATION_OF_NECROTIC_CELL_DEATH	1	1.1941533	0.17234468
Upregulated Genes	GO_HIPPO_SIGNALING	1	1.1938695	0.176
Upregulated Genes	GO_REGULATION_OF_ENDOTHELIAL_CELL_DIFFERENTIATION	1	1.1930994	0.19844358
Upregulated Genes	GO_ENDOTHELIAL_CELL_APOPTOTIC_PROCESS	1	1.1927633	0.20481928
Upregulated Genes	GO_COLLAGEN_FIBRIL_ORGANIZATION	1	1.1925275	0.22519083
Upregulated Genes	GO_POSITIVE_REGULATION_OF_CANONICAL_WNT_SIGNALING_PATHWAY	3	1.1925042	0.23194748
Upregulated Genes	GO_REGULATION_OF_GRANULOCYTE_CHEMOTAXIS	1	1.1922224	0.23274162
Upregulated Genes	GO_REGULATION_OF_MACROPHAGE_MIGRATION	1	1.1920867	0.20119523
Upregulated Genes	GO_NEGATIVE_REGULATION_OF_MEMBRANE_DEPOLARIZATION	1	1.1915538	0.18837675
Upregulated Genes	GO_MEMBRANE_DEPOLARIZATION	1	1.1915042	0.2
Upregulated Genes	GO_REGULATION_OF_CYCLASE_ACTIVITY	1	1.1910456	0.20833333
Upregulated Genes	GO_BONE_TRABECULA_MORPHOGENESIS	1	1.1904823	0.20990099
Upregulated Genes	GO_REGULATION_OF_ALPHA_BETA_T_CELL_ACTIVATION	4	1.1902167	0.2585034
Upregulated Genes	GO_NEGATIVE_REGULATION_OF_MITOCHONDRIAL_OUTER_MEMBRANE_PERMEABILIZATION_II	1	1.1900275	0.21966527
Upregulated Genes	GO_POSITIVE_REGULATION_OF_EPITHELIAL_CELL_DIFFERENTIATION	1	1.1894059	0.20790021
Upregulated Genes	GO_ORGANIC_ACID_CATABOLIC_PROCESS	3	1.1889467	0.25238097
Upregulated Genes	GO_CELLULAR_RESPONSE_TO_RETINOIC_ACID	1	1.1887883	0.203125
Upregulated Genes	GO_NEGATIVE_REGULATION_OF_ADENYLATE_CYCLASE_ACTIVITY	1	1.1881535	0.22524272
Upregulated Genes	GO_BLOOD_VESSEL_MATURATION	1	1.1878462	0.22264151
Upregulated Genes	GO_CELL_SUBSTRATE_JUNCTION_ORGANIZATION	3	1.1876138	0.25413224
Upregulated Genes	GO_POSITIVE_REGULATION_OF_MACROPHAGE_MIGRATION	1	1.1868238	0.22779922
Upregulated Genes	GO_REGULATION_OF_BIOMINERALIZATION	1	1.1867579	0.20925553
Upregulated Genes	GO_CELLULAR_RESPONSE_TO_ACID_CHEMICAL	1	1.1866758	0.20436507
Upregulated Genes	GO_TRABECULA_MORPHOGENESIS	1	1.186281	0.22291666
Upregulated Genes	GO_POSITIVE_REGULATION_OF_ENDOTHELIAL_CELL_DIFFERENTIATION	1	1.1859219	0.23481782
Upregulated Genes	GO_TRABECULA_FORMATION	1	1.1854699	0.22839506
Upregulated Genes	GO_REGULATION_OF_SYNAPTIC_PLASTICITY	3	1.1854526	0.23076923
Upregulated Genes	GO_MITOCHONDRIAL_DEPOLARIZATION	1	1.1853844	0.2055336
Upregulated Genes	GO_REGULATION_OF_MACROPHAGE_CHEMOTAXIS	1	1.1852756	0.19631901
Upregulated Genes	GO_NEUROMUSCULAR_JUNCTION_DEVELOPMENT	1	1.1851499	0.21359223
Upregulated Genes	GO_CELLULAR_RESPONSE_TO_NUTRIENT	1	1.184918	0.20647773
Upregulated Genes	GO_POSITIVE_REGULATION_OF_PROTEIN_TYROSINE_KINASE_ACTIVITY	1	1.1847985	0.19175258
Upregulated Genes	GO_MODULATION_OF_PROCESS_OF_OTHER_ORGANISM	2	1.184784	0.26875
Upregulated Genes	GO_MICROGLIAL_CELL_PROLIFERATION	1	1.1842655	0.21722114
Upregulated Genes	GO_REPLACEMENT_OSSIFICATION	1	1.1841476	0.24561404
Upregulated Genes	GO_REGULATION_OF_ADENYLATE_CYCLASE_ACTIVITY	1	1.183857	0.23246492
Upregulated Genes	GO_LIMBIC_SYSTEM_DEVELOPMENT	3	1.1836152	0.26576576
Upregulated Genes	GO_COLLAGEN_ACTIVATED_SIGNALING_PATHWAY	1	1.1832063	0.22986248
Upregulated Genes	GO_COLLAGEN_ACTIVATED_TYROSINE_KINASE_RECEPTOR_SIGNALING_PATHWAY	1	1.1823407	0.25155926
Upregulated Genes	GO_INOSITOL_LIPID_MEDIATED_SIGNALING	3	1.181641	0.27494457
Upregulated Genes	GO_HIPPOCAMPUS_DEVELOPMENT	3	1.1814727	0.24557522
Upregulated Genes	GO_TOOTH_MINERALIZATION	1	1.1814452	0.22222222
Upregulated Genes	GO_REGULATION_OF_ENDOTHELIAL_CELL_DEVELOPMENT	1	1.1807142	0.23493975
Upregulated Genes	GO_RECEPTOR_CLUSTERING	1	1.1802276	0.24273859
Upregulated Genes	GO_REGULATION_OF_VASCULAR_PERMEABILITY	1	1.1799035	0.21428572
Upregulated Genes	GO_POSITIVE_REGULATION_OF_MACROPHAGE_DIFFERENTIATION	1	1.1781172	0.2165992
Upregulated Genes	GO_TISSUE_REGENERATION	3	1.1779302	0.2780269
Upregulated Genes	GO_PROTEIN_LOCALIZATION_TO_MITOCHONDRION	1	1.1769252	0.2275574

Upregulated Genes	GO_MESENCHYME_DEVELOPMENT	7	1.1756096	0.27505827
Upregulated Genes	GO_CELLULAR_LIPID_METABOLIC_PROCESS	14	1.1753331	0.29473683
Upregulated Genes	GO_REGULATION_OF_PHOSPHORUS_METABOLIC_PROCESS	38	1.1749277	0.2630058
Upregulated Genes	GO_PROCESS_UTILIZING_AUTOPHAGIC_MECHANISM	5	1.1737504	0.28538814
Upregulated Genes	GO_DIRECT_OSSIFICATION	1	1.1737119	0.24899599
Upregulated Genes	GO_PHOSPHATIDYLINOSITOL_3_KINASE_SIGNALING	3	1.1732812	0.292517
Upregulated Genes	GO_BONE_TRABECULA_FORMATION	1	1.1729285	0.2368932
Upregulated Genes	GO_REGULATION_OF_PHOSPHORYLATION	38	1.1727563	0.2650273
Upregulated Genes	GO_RESPONSE_TO_ORGANIC_CYCLIC_COMPOUND	29	1.171989	0.26598465
Upregulated Genes	GO_GAMMA_AMINOBUTYRIC_ACID_SIGNALING_PATHWAY	1	1.171787	0.23217922
Upregulated Genes	GO_RESPONSE_TO_VITAMIN_E	1	1.1717256	0.2193676
Upregulated Genes	GO_CELL_MOTILITY	35	1.1712985	0.2679558
Upregulated Genes	GO_SMOOTH_MUSCLE_CELL_PROLIFERATION	3	1.1712626	0.26211452
Upregulated Genes	GO_PROTEIN_INSERTION_INTO_MEMBRANE	1	1.1712055	0.24621212
Upregulated Genes	GO_ANOIKIS	1	1.1710993	0.22862823
Upregulated Genes	GO_REGULATION_OF_TRANSMEMBRANE_RECEPTOR_PROTEIN_SERINE_THREONINE_KINASE_SI	6	1.1709524	0.26697892
Upregulated Genes	GO_CELL_MATRIX_ADHESION	3	1.1694584	0.28418803
Upregulated Genes	GO_NEGATIVE_REGULATION_OF_LYASE_ACTIVITY	1	1.16918	0.24746451
Upregulated Genes	GO_RESPONSE_TO_FLUORIDE	1	1.168047	0.23188406
Upregulated Genes	GO_PURINE_CONTAINING_COMPOUND_BIOSYNTHETIC_PROCESS	4	1.1677481	0.2639225
Upregulated Genes	GO_CELLULAR_RESPONSE_TO_VITAMIN	1	1.1658901	0.23809524
Upregulated Genes	GO_RESPONSE_TO_ISOQUINOLINE_ALKALOID	1	1.1650566	0.24536082
Upregulated Genes	GO_NEGATIVE_REGULATION_OF_EPITHELIAL_CELL_APOPTOTIC_PROCESS	1	1.1649239	0.25110132
Upregulated Genes	GO_POSITIVE_REGULATION_OF_RELEASE_OF_CYTOCHROME_C_FROM_MITOCHONDRIA	1	1.1645133	0.24550898
Upregulated Genes	GO_MODULATION_BY_HOST_OF_SYMBIONT_PROCESS	2	1.1632996	0.29273504
Upregulated Genes	GO_LEUKOCYTE_MEDIATED_IMMUNITY	13	1.1630256	0.2800926
Upregulated Genes	GO_LEUKOCYTE_APOPTOTIC_PROCESS	3	1.1621685	0.26760563
Upregulated Genes	GO_PROTEIN_INSERTION_INTO_MITOCHONDRIAL_MEMBRANE_INVOLVED_IN_APOPTOTIC_SIGI	1	1.1619394	0.24421053
Upregulated Genes	GO_MYELOID_CELL_APOPTOTIC_PROCESS	3	1.1618544	0.29359823
Upregulated Genes	GO_REGULATION_OF_LYASE_ACTIVITY	1	1.1616853	0.26060605
Upregulated Genes	GO_ESTABLISHMENT_OF_PROTEIN_LOCALIZATION_TO_MITOCHONDRIAL_MEMBRANE	1	1.1605862	0.25865582
Upregulated Genes	GO_CHROMOSOME_CONDENSATION	5	1.1603881	0.2837838
Upregulated Genes	GO_REGULATION_OF_RELEASE_OF_CYTOCHROME_C_FROM_MITOCHONDRIA	1	1.159512	0.2555781
Upregulated Genes	GO_REGULATION_OF_PODOSOME_ASSEMBLY	1	1.1585383	0.2615694
Upregulated Genes	GO_POSITIVE_REGULATION_OF_MITOCHONDRION_ORGANIZATION	1	1.1557719	0.25838265
Upregulated Genes	GO_REGULATION_OF_RESPONSE_TO_STRESS	31	1.1554611	0.2805195
Upregulated Genes	GO_REGULATION_OF_PROTEOLYSIS	8	1.155431	0.31542057
Upregulated Genes	GO_RIBOSE_PHOSPHATE_BIOSYNTHETIC_PROCESS	2	1.1550988	0.28654972
Upregulated Genes	GO_POSITIVE_REGULATION_OF_INTRINSIC_APOPTOTIC_SIGNALING_PATHWAY	1	1.1545827	0.26459143
Upregulated Genes	GO_REGULATION_OF_AUTOPHAGY	5	1.153516	0.31625834
Upregulated Genes	GO_POSITIVE_REGULATION_OF_PODOSOME_ASSEMBLY	1	1.15146	0.28962818
Upregulated Genes	GO_EXTRACELLULAR_MATRIX_ASSEMBLY	1	1.1494586	0.26968503
Upregulated Genes	GO_REGULATION_OF_NUCLEAR_CELL_CYCLE_DNA_REPLICATION	2	1.149396	0.32054177
Upregulated Genes	GO_RESPONSE_TO_WOUNDING	15	1.14867	0.27970296
Upregulated Genes	GO_NEGATIVE_REGULATION_OF_CELL_CYCLE_ARREST	2	1.1477447	0.3059548
Upregulated Genes	GO_INTERACTION_WITH_SYMBIONT	2	1.1465409	0.28794643
Upregulated Genes	GO_NEGATIVE_REGULATION_OF_MACROAUTOPHAGY	1	1.1456136	0.27439025
Upregulated Genes	GO_NEGATIVE_REGULATION_OF_PROTEIN_LOCALIZATION_TO_MEMBRANE	2	1.1453363	0.30322582
Upregulated Genes	GO_MICROTUBULE_BASED_TRANSPORT	3	1.1452587	0.28395063
Upregulated Genes	GO_MITOCHONDRIAL_TRANSPORT	3	1.1442056	0.32093024
Upregulated Genes	GO_MUSCLE_STRUCTURE_DEVELOPMENT	13	1.1439714	0.3041237
Upregulated Genes	GO_CARTILAGE_DEVELOPMENT	8	1.1433647	0.3106796
Upregulated Genes	GO_PRODUCTION_OF_MOLECULAR_MEDIATOR_OF_IMMUNE_RESPONSE	7	1.1425723	0.29820627
Upregulated Genes	GO_PODOSOME_ASSEMBLY	1	1.1409917	0.2874016
Upregulated Genes	GO_CYTOSOLIC_CALCIUM_ION_TRANSPORT	1	1.1371727	0.3007968
Upregulated Genes	GO_CALCIUM_ION_TRANSMEMBRANE_IMPORT_INTO_CYTOSOL	1	1.1363839	0.2951807
Upregulated Genes	GO_CONNECTIVE_TISSUE_DEVELOPMENT	8	1.1348228	0.3278302
Upregulated Genes	GO_CELL_CELL_JUNCTION_ORGANIZATION	5	1.1343176	0.31264368
Upregulated Genes	GO_CELLULAR_AMINO_ACID_METABOLIC_PROCESS	2	1.1334802	0.28923768
Upregulated Genes	GO_POSITIVE_REGULATION_OF_INFLAMMATORY_RESPONSE	6	1.1330217	0.32843137
Upregulated Genes	GO_RESPONSE_TO_ENDOGENOUS_STIMULUS	34	1.1327732	0.31016043
Upregulated Genes	GO_POSITIVE_REGULATION_OF_GLIOGENESIS	4	1.1312006	0.30997878
Upregulated Genes	GO_RESPONSE_TO_INTERLEUKIN_12	1	1.1306131	0.308
Upregulated Genes	GO_CELLULAR_RESPONSE_TO_OXYGEN_CONTAINING_COMPOUND	29	1.1282378	0.29461756
Upregulated Genes	GO_CELLULAR_COMPONENT_MORPHOGENESIS	13	1.1274633	0.32692307
Upregulated Genes	GO_SEQUESTERING_OF_CALCIUM_ION	1	1.1261537	0.31663325
Upregulated Genes	GO_POSITIVE_REGULATION_OF_CELL_DIFFERENTIATION	27	1.1248225	0.30578512
Upregulated Genes	GO_SERINE_FAMILY_AMINO_ACID_BIOSYNTHETIC_PROCESS	2	1.1245819	0.32765958
Upregulated Genes	GO_INORGANIC_ANION_TRANSPORT	2	1.1231227	0.32683983
Upregulated Genes	GO_REGULATION_OF_PROTEIN_MODIFICATION_PROCESS	41	1.1192257	0.34202898

Upregulated Genes	GO_L_SERINE_METABOLIC_PROCESS	2	1.1175947	0.34446764
Upregulated Genes	GO_ACTIVATION_OF_MAPKK_ACTIVITY	2	1.117578	0.318872
Upregulated Genes	GO_SYMBIOTIC_PROCESS	15	1.1164547	0.3166227
Upregulated Genes	GO_STEROL_BIOSYNTHETIC_PROCESS	2	1.1149981	0.34468085
Upregulated Genes	GO_NEGATIVE_REGULATION_OF_MAP_KINASE_ACTIVITY	2	1.1134237	0.33261803
Upregulated Genes	GO_SERINE_FAMILY_AMINO_ACID_METABOLIC_PROCESS	2	1.1132933	0.34623656
Upregulated Genes	GO_RECEPTOR_CATABOLIC_PROCESS	2	1.1128817	0.3263158
Upregulated Genes	GO_RESPONSE_TO_CATECHOLAMINE	2	1.1122216	0.33829787
Upregulated Genes	GO_ANATOMICAL_STRUCTURE_FORMATION_INVOLVED_IN_MORPHOGENESIS	18	1.1114819	0.30079156
Upregulated Genes	GO_CELLULAR_AMINO_ACID_BIOSYNTHETIC_PROCESS	2	1.1106645	0.34303534
Upregulated Genes	GO_PROTEIN_LOCALIZATION_TO_CELL_CELL_JUNCTION	2	1.1091931	0.33402923
Upregulated Genes	GO_ALPHA_AMINO_ACID_BIOSYNTHETIC_PROCESS	2	1.1081127	0.3549107
Upregulated Genes	GO_ALPHA_AMINO_ACID_METABOLIC_PROCESS	2	1.1074883	0.34591195
Upregulated Genes	GO_PROTEIN_LOCALIZATION_TO_CELL_JUNCTION	2	1.1068938	0.32985386
Upregulated Genes	GO_TELOMERE_CAPPING	2	1.1060207	0.3612245
Upregulated Genes	GO_SUBSTRATE_ADHESION_DEPENDENT_CELL_SPREADING	1	1.1058962	0.3394309
Upregulated Genes	GO_REGULATION_OF_MACROAUTOPHAGY	2	1.103771	0.33333334
Upregulated Genes	GO_POSITIVE_REGULATION_OF_DENDRITIC_CELL_CYTOKINE_PRODUCTION	1	1.1017816	0.31132075
Upregulated Genes	GO_IRON_ION_TRANSPORT	1	1.1014129	0.32897604
Upregulated Genes	GO_NEGATIVE_REGULATION_OF_SUBSTRATE_ADHESION_DEPENDENT_CELL_SPREADING	1	1.1004486	0.3446215
Upregulated Genes	GO_POSITIVE_REGULATION_OF_GRANULOCYTE_MACROPHAGE_COLONY_STIMULATING_FACTC	1	1.0985981	0.37373737
Upregulated Genes	GO_ORGANOPHOSPHATE_CATABOLIC_PROCESS	4	1.0985861	0.33846155
Upregulated Genes	GO_RECEPTOR_METABOLIC_PROCESS	2	1.0979431	0.3618421
Upregulated Genes	GO_POSITIVE_REGULATION_OF_PLASMINOGEN_ACTIVATION	1	1.0977824	0.3613963
Upregulated Genes	GO_POSITIVE_REGULATION_OF_MYELOID_LEUKOCYTE_MEDIATED_IMMUNITY	1	1.0975251	0.3533058
Upregulated Genes	GO_ASSOCIATIVE_LEARNING	1	1.0972639	0.33584905
Upregulated Genes	GO_GRANULOCYTE_MACROPHAGE_COLONY_STIMULATING_FACTOR_PRODUCTION	1	1.0969046	0.34697855
Upregulated Genes	GO_POSITIVE_REGULATION_OF_DEFENSE_RESPONSE_TO_VIRUS_BY_HOST	1	1.0962415	0.36434108
Upregulated Genes	GO_POSITIVE_REGULATION_OF_PROTEIN_MATURATION	1	1.0961968	0.3493014
Upregulated Genes	GO_TUMOR_NECROSIS_FACTOR_MEDIATED_SIGNALING_PATHWAY	1	1.0961553	0.37166324
Upregulated Genes	GO_REGULATION_OF_SUBSTRATE_ADHESION_DEPENDENT_CELL_SPREADING	1	1.0955743	0.36511156
Upregulated Genes	GO_REGULATION_OF_OSTEOCLAST_DIFFERENTIATION	1	1.0949687	0.3250478
Upregulated Genes	GO_POSITIVE_REGULATION_OF_TUMOR_NECROSIS_FACTOR_SUPERFAMILY_CYTOKINE_PRODU	4	1.0947351	0.3489933
Upregulated Genes	GO_TRANSITION_METAL_ION_TRANSPORT	1	1.094465	0.3507014
Upregulated Genes	GO_NEUROPEPTIDE_SIGNALING_PATHWAY	1	1.0942984	0.35928145
Upregulated Genes	GO_POSITIVE_REGULATION_OF_CYTOKINE_PRODUCTION	13	1.0936238	0.35915494
Upregulated Genes	GO_SMAD_PROTEIN_SIGNAL_TRANSDUCTION	1	1.0933182	0.35550934
Upregulated Genes	GO_RIG_I_SIGNALING_PATHWAY	1	1.0931488	0.34949496
Upregulated Genes	GO_POSITIVE_REGULATION_OF_OSTEOCLAST_DIFFERENTIATION	1	1.0922751	0.3407258
Upregulated Genes	GO_MYELOID_DENDRITIC_CELL_CYTOKINE_PRODUCTION	1	1.0888293	0.38104838
Upregulated Genes	GO_NEUTROPHIL_DIFFERENTIATION	2	1.0888104	0.36717063
Upregulated Genes	GO_DENDRITIC_CELL_CYTOKINE_PRODUCTION	1	1.0880125	0.3253493
Upregulated Genes	GO_STEROID_BIOSYNTHETIC_PROCESS	2	1.0871574	0.37229437
Upregulated Genes	GO_REGULATION_OF_REGULATED_SECRETORY_PATHWAY	1	1.0866814	0.35185185
Upregulated Genes	GO_TUMOR_NECROSIS_FACTOR_SECRETION	4	1.0853438	0.3611738
Upregulated Genes	GO_SKELETAL_MUSCLE_ORGAN_DEVELOPMENT	1	1.0846087	0.37058824
Upregulated Genes	GO_CELLULAR_RESPONSE_TO_CADMIUM_ION	1	1.0845617	0.3747412
Upregulated Genes	GO_FC_EPSILON_RECEPTOR_SIGNALING_PATHWAY	1	1.0842377	0.36610878
Upregulated Genes	GO_RESPONSE_TO_GRAVITY	1	1.0841428	0.3628692
Upregulated Genes	GO_RESPONSE_TO_IMMOBILIZATION_STRESS	1	1.0832393	0.38372093
Upregulated Genes	GO_REGULATION_OF_DEFENSE_RESPONSE_TO_VIRUS_BY_HOST	1	1.0830214	0.37373737
Upregulated Genes	GO_TUMOR_NECROSIS_FACTOR_SUPERFAMILY_CYTOKINE_PRODUCTION	4	1.082429	0.34684685
Upregulated Genes	GO_NEGATIVE_REGULATION_OF_METALLOPEPTIDASE_ACTIVITY	1	1.0822144	0.36681223
Upregulated Genes	GO_REGULATION_OF_BODY_FLUID_LEVELS	10	1.0816762	0.3686747
Upregulated Genes	GO_HEMATOPOIETIC_STEM_CELL_DIFFERENTIATION	1	1.0813581	0.37051794
Upregulated Genes	GO_PRI_MIRNA_TRANSCRIPTION_BY_RNA_POLYMERASE_II	1	1.0813576	0.4054054
Upregulated Genes	GO_REGULATION_OF_EXOCYTOSIS	1	1.0809299	0.36082473
Upregulated Genes	GO_NEGATIVE_REGULATION_OF_SODIUM_ION_TRANSPORT	1	1.0805404	0.37396693
Upregulated Genes	GO_POSITIVE_REGULATION_OF_RECEPTOR_SIGNALING_PATHWAY_VIA_STAT	2	1.0802952	0.3877551
Upregulated Genes	GO_SLEEP	1	1.080059	0.3787575
Upregulated Genes	GO_POSITIVE_REGULATION_OF_MYELOID_LEUKOCYTE_CYTOKINE_PRODUCTION_INVOLVED_IN	1	1.0795038	0.378
Upregulated Genes	GO_REGULATION_OF_RECEPTOR_SIGNALING_PATHWAY_VIA_STAT	2	1.079311	0.35758835
Upregulated Genes	GO_POSITIVE_REGULATION_OF_PRI_MIRNA_TRANSCRIPTION_BY_RNA_POLYMERASE_II	1	1.0791593	0.35841584
Upregulated Genes	GO_REGULATION_OF_MAST_CELL_ACTIVATION_INVOLVED_IN_IMMUNE_RESPONSE	1	1.0789905	0.36862746
Upregulated Genes	GO_NEGATIVE_REGULATION_OF_PLATELET_ACTIVATION	1	1.0786914	0.37090164
Upregulated Genes	GO_FORMATION_OF_EXTRACHROMOSOMAL_CIRCULAR_DNA	2	1.0786427	0.38274336
Upregulated Genes	GO_LIPID_BIOSYNTHETIC_PROCESS	12	1.0782541	0.36989796
Upregulated Genes	GO_MYELOID_LEUKOCYTE_CYTOKINE_PRODUCTION	1	1.0780978	0.39494163
Upregulated Genes	GO_MAST_CELL_ACTIVATION	1	1.0780079	0.38430583
Upregulated Genes	GO_REGULATION_OF_CHEMOTAXIS	6	1.0773137	0.35514018

Upregulated Genes	GO_TROPHOBLAST_CELL_MIGRATION	1	1.0769594	0.37278107
Upregulated Genes	GO_REGULATION_OF_LYMPHOCYTE_DIFFERENTIATION	6	1.0761379	0.32666665
Upregulated Genes	GO_CD8_POSITIVE_ALPHA_BETA_T_CELL_ACTIVATION	1	1.0759195	0.3884462
Upregulated Genes	GO_REGULATION_OF_DEFENSE_RESPONSE_TO_VIRUS	1	1.0751638	0.3987854
Upregulated Genes	GO_NEGATIVE_REGULATION_OF_KINASE_ACTIVITY	6	1.0751199	0.37697518
Upregulated Genes	GO_REGULATION_OF_SENSORY_PERCEPTION	1	1.0747031	0.37938145
Upregulated Genes	GO_MEMBRANE_PROTEIN_PROTEOLYSIS	1	1.0746591	0.3782696
Upregulated Genes	GO_POSITIVE_REGULATION_OF_TYROSINE_PHOSPHORYLATION_OF_STAT_PROTEIN	2	1.0745656	0.37339056
Upregulated Genes	GO_REGULATION_OF_METALLOPEPTIDASE_ACTIVITY	1	1.0744394	0.37724552
Upregulated Genes	GO_WOUND_HEALING_INVOLVED_IN_INFLAMMATORY_RESPONSE	1	1.074231	0.38065845
Upregulated Genes	GO_NEGATIVE_REGULATION_OF_TROPHOBLAST_CELL_MIGRATION	1	1.0740432	0.39019608
Upregulated Genes	GO_REGULATION_OF_MAST_CELL_ACTIVATION	1	1.0734553	0.36666667
Upregulated Genes	GO_SKELETAL_MUSCLE_CELL_DIFFERENTIATION	1	1.0731322	0.3963039
Upregulated Genes	GO_REGULATION_OF_SYNAPTIC_TRANSMISSION_GLUTAMATERGIC	1	1.0731279	0.37550202
Upregulated Genes	GO_REGULATION_OF_HOMOTYPIC_CELL_CELL_ADHESION	1	1.0730929	0.38367346
Upregulated Genes	GO_CONNECTIVE_TISSUE_REPLACEMENT	1	1.0730472	0.3910387
Upregulated Genes	GO_REGULATION_OF_CD8_POSITIVE_ALPHA_BETA_T_CELL_ACTIVATION	1	1.0726944	0.37181997
Upregulated Genes	GO_NEGATIVE_REGULATION_OF_SMOOTHENED_SIGNALING_PATHWAY	1	1.0719166	0.37250996
Upregulated Genes	GO_MACROAUTOPHAGY	2	1.071458	0.39578947
Upregulated Genes	GO_INSEMINATION	1	1.0713248	0.38771185
Upregulated Genes	GO_CONDITIONED_TASTE_AVERSION	1	1.0710899	0.3960396
Upregulated Genes	GO_INTEGRIN_MEDIATED_SIGNALING_PATHWAY	1	1.0708747	0.3958763
Upregulated Genes	GO_SECONDARY_PALATE_DEVELOPMENT	1	1.0706898	0.37029704
Upregulated Genes	GO_INNERVATION	1	1.0700449	0.39793813
Upregulated Genes	GO_DETECTION_OF_MECHANICAL_STIMULUS_INVOLVED_IN_SENSORY_PERCEPTION	1	1.0700212	0.40425533
Upregulated Genes	GO_POSITIVE_REGULATION_OF_DEFENSE_RESPONSE	10	1.0697211	0.3564815
Upregulated Genes	GO_NEGATIVE_REGULATION_OF_HOMOTYPIC_CELL_CELL_ADHESION	1	1.0696192	0.38075313
Upregulated Genes	GO_REGULATION_OF_GRANULOCYTE_DIFFERENTIATION	1	1.0696138	0.37950665
Upregulated Genes	GO_BIOMINERALIZATION	3	1.0694315	0.3568376
Upregulated Genes	GO_HEMATOPOIETIC_STEM_CELL_PROLIFERATION	1	1.068896	0.3852459
Upregulated Genes	GO_MAST_CELL_MEDIATED_IMMUNITY	1	1.0688562	0.41532257
Upregulated Genes	GO_NEGATIVE_REGULATION_OF_MEMBRANE_PROTEIN_ECTODOMAIN_PROTEOLYSIS	1	1.0686772	0.40674603
Upregulated Genes	GO_REGULATION_OF_MEMBRANE_PROTEIN_ECTODOMAIN_PROTEOLYSIS	1	1.0685023	0.39676112
Upregulated Genes	GO_REGULATION_OF_LEUKOCYTE_DEGRANULATION	1	1.0683727	0.39501038
Upregulated Genes	GO_NEGATIVE_REGULATION_OF_MULTI_ORGANISM_PROCESS	1	1.0678415	0.42147118
Upregulated Genes	GO_REGULATION_OF_PLATELET_ACTIVATION	1	1.0677371	0.37869823
Upregulated Genes	GO_B_CELL_RECEPTOR_SIGNALING_PATHWAY	1	1.0672826	0.41359222
Upregulated Genes	GO_REGULATION_OF_HEMATOPOIETIC_STEM_CELL_DIFFERENTIATION	1	1.0668048	0.3846154
Upregulated Genes	GO_COPULATION	1	1.0663381	0.3726708
Upregulated Genes	GO_RESPONSE_TO_CORTICOSTEROID	11	1.0663104	0.36633664
Upregulated Genes	GO_RECEPTOR_SIGNALING_PATHWAY_VIA_STAT	2	1.0662302	0.38723403
Upregulated Genes	GO_POSITIVE_REGULATION_OF_CD8_POSITIVE_ALPHA_BETA_T_CELL_ACTIVATION	1	1.0659677	0.39839035
Upregulated Genes	GO_CD4_POSITIVE_ALPHA_BETA_T_CELL_PROLIFERATION	1	1.0655187	0.37959182
Upregulated Genes	GO_POSITIVE_REGULATION_OF_ASTROCYTE_DIFFERENTIATION	1	1.0652113	0.41035858
Upregulated Genes	GO_T_CELL_TOLERANCE_INDUCTION	1	1.0650746	0.4032258
Upregulated Genes	GO_SPHINGOMYELIN_CATABOLIC_PROCESS	1	1.0645134	0.37708333
Upregulated Genes	GO_CELLULAR_RESPONSE_TO_OXYGEN_LEVELS	6	1.0640699	0.36130536
Upregulated Genes	GO_REGULATION_OF_SODIUM_ION_TRANSPORT	1	1.064029	0.418
Upregulated Genes	GO_POSITIVE_REGULATION_OF_GRANULOCYTE_DIFFERENTIATION	1	1.0639471	0.412
Upregulated Genes	GO_REGULATION_OF_BICELLULAR_TIGHT_JUNCTION_ASSEMBLY	1	1.0638665	0.41482967
Upregulated Genes	GO_NEGATIVE_REGULATION_OF_PLASMINOGEN_ACTIVATION	1	1.0636404	0.41434264
Upregulated Genes	GO_TELOMERE_MAINTENANCE_VIA_RECOMBINATION	2	1.0633105	0.39092872
Upregulated Genes	GO_CARDIAC_VENTRICLE_MORPHOGENESIS	1	1.0632718	0.40152964
Upregulated Genes	GO_CD8_POSITIVE_ALPHA_BETA_T_CELL_DIFFERENTIATION	1	1.0632514	0.3984375
Upregulated Genes	GO_PERIPHERAL_NERVOUS_SYSTEM_NEURON_DIFFERENTIATION	1	1.0628407	0.42553192
Upregulated Genes	GO_CEREBELLAR_GRANULAR_LAYER_DEVELOPMENT	1	1.0622417	0.3970894
Upregulated Genes	GO_SECRETORY_GRANULE_ORGANIZATION	1	1.0620328	0.39363816
Upregulated Genes	GO_REGULATION_OF_INTEGRIN_MEDIATED_SIGNALING_PATHWAY	1	1.0618342	0.40447155
Upregulated Genes	GO_REGULATION_OF_HEMATOPOIETIC_PROGENITOR_CELL_DIFFERENTIATION	1	1.0617722	0.4110672
Upregulated Genes	GO_NEGATIVE_REGULATION_OF_PROTEIN_MATURATION	1	1.0616516	0.39087301
Upregulated Genes	GO_MEMBRANE_PROTEIN_ECTODOMAIN_PROTEOLYSIS	1	1.0616404	0.4069098
Upregulated Genes	GO_REGULATION_OF_CD8_POSITIVE_ALPHA_BETA_T_CELL_DIFFERENTIATION	1	1.0612422	0.4052953
Upregulated Genes	GO_ENDOCARDIAL_CUSHION_MORPHOGENESIS	1	1.0611328	0.3980198
Upregulated Genes	GO_POSITIVE_REGULATION_OF_CELL_DEATH	14	1.060894	0.375
Upregulated Genes	GO_NEGATIVE_REGULATION_OF_PATHWAY_RESTRICTED_SMAD_PROTEIN_PHOSPHORYLATION	1	1.0596339	0.40613025
Upregulated Genes	GO_INFLAMMATORY_RESPONSE_TO_WOUNDING	1	1.0594082	0.41101694
Upregulated Genes	GO_TRACHEA_MORPHOGENESIS	1	1.0588397	0.4106383
Upregulated Genes	GO_SYNAPTIC_TRANSMISSION_GLUTAMATERGIC	1	1.0583624	0.41
Upregulated Genes	GO_HINDBRAIN_MORPHOGENESIS	1	1.0576415	0.4180328
Upregulated Genes	GO_REGULATION_OF_B_CELL_RECEPTOR_SIGNALING_PATHWAY	1	1.0570114	0.41910332

Upregulated Genes	GO_NK_T_CELL_DIFFERENTIATION	1	1.0564482	0.42682928
Upregulated Genes	GO_JNK_CASCADE	4	1.0558385	0.37588653
Upregulated Genes	GO_REGULATION_OF_T_CELL_TOLERANCE_INDUCTION	1	1.0558116	0.41439688
Upregulated Genes	GO_REGULATION_OF_ANATOMICAL_STRUCTURE_MORPHOGENESIS	13	1.0546861	0.37704918
Upregulated Genes	GO_CEREBELLAR_GRANULAR_LAYER_MORPHOGENESIS	1	1.0546013	0.4319066
Upregulated Genes	GO_POSITIVE_REGULATION_OF_NEURON_PROJECTION_DEVELOPMENT	4	1.0535916	0.3546911
Upregulated Genes	GO_REGULATION_OF_DEVELOPMENT_HETEROCHRONIC	1	1.0534486	0.41894737
Upregulated Genes	GO_SENSORY_PERCEPTION_OF_PAIN	1	1.0533749	0.42607003
Upregulated Genes	GO_EMBRYONIC_SKELETAL_SYSTEM_MORPHOGENESIS	1	1.0531721	0.4012474
Upregulated Genes	GO_HINDBRAIN_DEVELOPMENT	2	1.0527575	0.4038055
Upregulated Genes	GO_CELLULAR_RESPONSE_TO_PROSTAGLANDIN_STIMULUS	2	1.0524412	0.40638298
Upregulated Genes	GO_REGULATION_OF_EPITHELIAL_TO_MESENCHYMAL_TRANSITION_INVOLVED_IN_ENDOCARD	1	1.0523502	0.4125
Upregulated Genes	GO_VITAMIN_METABOLIC_PROCESS	7	1.0522943	0.39819005
Upregulated Genes	GO_MESENCHYMAL_CELL_PROLIFERATION	1	1.0520072	0.40120968
Upregulated Genes	GO_BRANCHING_INVOLVED_IN_BLOOD_VESSEL_MORPHOGENESIS	1	1.0517533	0.42138365
Upregulated Genes	GO_CEREBELLAR_CORTEX_MORPHOGENESIS	1	1.0516776	0.4248497
Upregulated Genes	GO_POSITIVE_REGULATION_OF_CD4_POSITIVE_ALPHA_BETA_T_CELL_PROLIFERATION	1	1.0510788	0.42798355
Upregulated Genes	GO_DETECTION_OF_STIMULUS_INVOLVED_IN_SENSORY_PERCEPTION	1	1.0506735	0.4180328
Upregulated Genes	GO_REGULATION_OF_HEART_MORPHOGENESIS	1	1.0504985	0.40246406
Upregulated Genes	GO_COMMON_PARTNER_SMAD_PROTEIN_PHOSPHORYLATION	1	1.0503111	0.42694497
Upregulated Genes	GO_TYROSINE_PHOSPHORYLATION_OF_STAT_PROTEIN	2	1.0502458	0.4069264
Upregulated Genes	GO_ENDOCARDIAL_CUSHION_FORMATION	1	1.0501834	0.4107884
Upregulated Genes	GO_MEMBRANE_LIPID_CATABOLIC_PROCESS	1	1.0501168	0.41393444
Upregulated Genes	GO_CRANIAL_SKELETAL_SYSTEM_DEVELOPMENT	1	1.0501045	0.42975205
Upregulated Genes	GO_RESPONSE_TO_STEROL	1	1.0498316	0.41566265
Upregulated Genes	GO_NEGATIVE_REGULATION_OF_PLATELET_AGGREGATION	1	1.0497965	0.40368852
Upregulated Genes	GO_REGULATION_OF_SKELETAL_MUSCLE_TISSUE_REGENERATION	1	1.0496181	0.40725806
Upregulated Genes	GO_EMBRYONIC_HEMOPOIESIS	1	1.0485334	0.42406312
Upregulated Genes	GO_ENERGY_HOMEOSTASIS	2	1.0482186	0.43695652
Upregulated Genes	GO_OUTFLOW_TRACT_MORPHOGENESIS	1	1.0481895	0.4288703
Upregulated Genes	GO_REGULATION_OF_MESENCHYMAL_CELL_PROLIFERATION	1	1.0481387	0.4269006
Upregulated Genes	GO_STRESS_FIBER_ASSEMBLY	2	1.0480541	0.40829694
Upregulated Genes	GO_REGULATION_OF_ACTIN_FILAMENT_BUNDLE_ASSEMBLY	2	1.0480139	0.41870824
Upregulated Genes	GO_POSITIVE_REGULATION_OF_EPITHELIAL_TO_MESENCHYMAL_TRANSITION_INVOLVED_IN_E	1	1.0479233	0.42436975
Upregulated Genes	GO_REGULATION_OF_PLATELET_AGGREGATION	1	1.0475361	0.44153225
Upregulated Genes	GO_CELLULAR_RESPONSE_TO_ALCOHOL	3	1.0468367	0.3955056
Upregulated Genes	GO_MYELOID_DENDRITIC_CELL_DIFFERENTIATION	1	1.0463775	0.40524194
Upregulated Genes	GO_ATRIOVENTRICULAR_VALVE_DEVELOPMENT	1	1.0444226	0.41263157
Upregulated Genes	GO_POSITIVE_REGULATION_OF_T_CELL_TOLERANCE_INDUCTION	1	1.0442772	0.42203742
Upregulated Genes	GO_POSITIVE_REGULATION_OF_PROTEOLYSIS	6	1.0441046	0.39784947
Upregulated Genes	GO_TRACHEA_FORMATION	1	1.044097	0.42886597
Upregulated Genes	GO_NEUTROPHIL_CHEMOTAXIS	2	1.0431648	0.41613588
Upregulated Genes	GO_TYPE_I_INTERFERON_PRODUCTION	5	1.0430447	0.41002277
Upregulated Genes	GO_HEART_VALVE_DEVELOPMENT	1	1.042822	0.47095436
Upregulated Genes	GO_POSITIVE_REGULATION_OF_ALPHA_BETA_T_CELL_PROLIFERATION	1	1.042614	0.41910332
Upregulated Genes	GO_POSITIVE_REGULATION_OF_MESENCHYMAL_CELL_PROLIFERATION	1	1.0423899	0.43953934
Upregulated Genes	GO_REGULATION_OF_NK_T_CELL_DIFFERENTIATION	1	1.0420814	0.4245283
Upregulated Genes	GO_OUTFLOW_TRACT_SEPTUM_MORPHOGENESIS	1	1.0419649	0.43942505
Upregulated Genes	GO_TRICUSPID_VALVE_DEVELOPMENT	1	1.0418131	0.42944786
Upregulated Genes	GO_POSITIVE_REGULATION_OF_TOLERANCE_INDUCTION	1	1.0417663	0.43763214
Upregulated Genes	GO_LUNG_MORPHOGENESIS	1	1.0416119	0.44612068
Upregulated Genes	GO_TRICUSPID_VALVE_MORPHOGENESIS	1	1.0414433	0.4288425
Upregulated Genes	GO_TOLERANCE_INDUCTION_TO_SELF_ANTIGEN	1	1.041145	0.4413519
Upregulated Genes	GO_ESTABLISHMENT_OF_RNA_LOCALIZATION	4	1.0403105	0.38938054
Upregulated Genes	GO_IRON_ION_HOMEOSTASIS	2	1.0394615	0.42459396
Upregulated Genes	GO_SPHINGOMYELIN_METABOLIC_PROCESS	1	1.0393149	0.4330544
Upregulated Genes	GO_NEGATIVE_REGULATION_OF_SIGNALING	27	1.0389205	0.3899204
Upregulated Genes	GO_NEGATIVE_REGULATION_OF_EPIDERMAL_CELL_DIFFERENTIATION	2	1.0388353	0.4088983
Upregulated Genes	GO_CELL_ADHESION_INVOLVED_IN_HEART_MORPHOGENESIS	1	1.0385914	0.4520256
Upregulated Genes	GO_EPITHELIAL_TO_MESENCHYMAL_TRANSITION_INVOLVED_IN_ENDOCARDIAL_CUSHION_FOF	1	1.0384836	0.44946235
Upregulated Genes	GO_CARDIAC_LEFT_VENTRICLE_MORPHOGENESIS	1	1.0384206	0.444227
Upregulated Genes	GO_TRANSITION_METAL_ION_HOMEOSTASIS	2	1.037978	0.41163793
Upregulated Genes	GO_REGULATION_OF_CARDIAC_EPITHELIAL_TO_MESENCHYMAL_TRANSITION	1	1.0377585	0.436214
Upregulated Genes	GO_VASCULAR_PROCESS_IN_CIRCULATORY_SYSTEM	3	1.0375156	0.4022472
Upregulated Genes	GO_ANIMAL_ORGAN_FORMATION	1	1.0373505	0.46387833
Upregulated Genes	GO_CHONDROCYTE_HYPERTROPHY	1	1.0368571	0.43960395
Upregulated Genes	GO_LENS_DEVELOPMENT_IN_CAMERA_TYPE_EYE	1	1.0368044	0.44444445
Upregulated Genes	GO_POSITIVE_REGULATION_OF_SKELETAL_MUSCLE_TISSUE_REGENERATION	1	1.036565	0.4563107
Upregulated Genes	GO_RECEPTOR_MEDIATED_ENDOCYTOSIS	1	1.0364457	0.44360903
Upregulated Genes	GO_POSITIVE_REGULATION_OF_ANIMAL_ORGAN_MORPHOGENESIS	1	1.0356587	0.45067698

Upregulated Genes	GO_LUNG_LOBE_DEVELOPMENT	1	1.0355636	0.4442231
Upregulated Genes	GO_CELLULAR_RESPONSE_TO_FATTY_ACID	2	1.0348902	0.43076923
Upregulated Genes	GO_OSTEOBLAST_DIFFERENTIATION	4	1.0344775	0.3954023
Upregulated Genes	GO_TRACHEA_DEVELOPMENT	1	1.0335875	0.46307385
Upregulated Genes	GO_MESENCHYME_MORPHOGENESIS	1	1.0334532	0.4621359
Upregulated Genes	GO_NEUTROPHIL_MIGRATION	2	1.032877	0.43037975
Upregulated Genes	GO_NEUROMUSCULAR_SYNAPTIC_TRANSMISSION	1	1.0327811	0.44158417
Upregulated Genes	GO_EMBRYONIC_CRANIAL_SKELETON_MORPHOGENESIS	1	1.032779	0.46761134
Upregulated Genes	GO_MULTI_ORGANISM_CELLULAR_PROCESS	1	1.0317538	0.44639376
Upregulated Genes	GO_GASTRULATION	1	1.0314509	0.46060607
Upregulated Genes	GO_RNA_LOCALIZATION	4	1.0311389	0.4036281
Upregulated Genes	GO_REGULATION_OF_CELL_JUNCTION_ASSEMBLY	2	1.0309654	0.43683085
Upregulated Genes	GO_PHOSPHOLIPID_CATABOLIC_PROCESS	1	1.0308334	0.44145873
Upregulated Genes	GO_MESENCHYMAL_CELL_DIFFERENTIATION	6	1.030514	0.39650872
Upregulated Genes	GO_CELL_CELL_ADHESION_MEDIATED_BY_INTEGRIN	1	1.0297377	0.4244898
Upregulated Genes	GO_MONOCARBOXYLIC_ACID_BIOSYNTHETIC_PROCESS	6	1.0294442	0.41826922
Upregulated Genes	GO_CARDIAC_EPITHELIAL_TO_MESENCHYMAL_TRANSITION	1	1.0293548	0.45489442
Upregulated Genes	GO_SYNAPTONEMAL_COMPLEX_ORGANIZATION	2	1.0291966	0.44175825
Upregulated Genes	GO_REGULATION_OF_VESICLE_MEDIATED_TRANSPORT	6	1.0289323	0.41007194
Upregulated Genes	GO_TELOMERIC_D_LOOP_DISASSEMBLY	2	1.0279133	0.44376278
Upregulated Genes	GO_FATTY_ACID_BIOSYNTHETIC_PROCESS	6	1.0278723	0.3846154
Upregulated Genes	GO_THROMBIN_ACTIVATED_RECEPTOR_SIGNALING_PATHWAY	2	1.0269516	0.4273859
Upregulated Genes	GO_RESPONSE_TO_PROSTAGLANDIN	2	1.0262114	0.44421488
Upregulated Genes	GO_POSITIVE_REGULATION_OF_MAPK_CASCADE	12	1.0260158	0.4219949
Upregulated Genes	GO_POTASSIUM_ION_TRANSPORT	3	1.0244515	0.4202586
Upregulated Genes	GO_CELL_MORPHOGENESIS	16	1.0236909	0.42631578
Upregulated Genes	GO_PLASMA_MEMBRANE_ORGANIZATION	4	1.0232916	0.41784036
Upregulated Genes	GO_NEGATIVE_REGULATION_OF_SYNAPSE_ORGANIZATION	2	1.0225732	0.4524313
Upregulated Genes	GO_RESPONSE_TO_FATTY_ACID	2	1.0218377	0.43524417
Upregulated Genes	GO_TYPE_B_PANCREATIC_CELL_APOPTOTIC_PROCESS	1	1.0210804	0.44510978
Upregulated Genes	GO_LIVER_MORPHOGENESIS	1	1.0202405	0.464
Upregulated Genes	GO_NEGATIVE_REGULATION_OF_EPITHELIAL_CELL_DIFFERENTIATION	2	1.020078	0.42548597
Upregulated Genes	GO_CD4_POSITIVE_ALPHA_BETA_T_CELL_CYTOKINE_PRODUCTION	1	1.0200427	0.46185568
Upregulated Genes	GO_CEREBELLAR_CORTEX_DEVELOPMENT	2	1.0189629	0.4371134
Upregulated Genes	GO_IMMUNOGLOBULIN_SECRETION	1	1.0184618	0.434
Upregulated Genes	GO_POSITIVE_REGULATION_OF_CYTOKINE_BIOSYNTHETIC_PROCESS	1	1.0173515	0.43243244
Upregulated Genes	GO_GERMINAL_CENTER_B_CELL_DIFFERENTIATION	1	1.0168761	0.46705428
Upregulated Genes	GO_CARDIAC_MUSCLE_CELL_CONTRACTION	2	1.0157908	0.42857143
Upregulated Genes	GO_VESICLE_LOCALIZATION	4	1.0145054	0.43119267
Upregulated Genes	GO_POSITIVE_REGULATION_OF_CELL_CELL_ADHESION_MEDIATED_BY_INTEGRIN	1	1.0144727	0.47188756
Upregulated Genes	GO_POSITIVE_REGULATION_OF_APOPTOTIC_SIGNALING_PATHWAY	4	1.0141425	0.42191142
Upregulated Genes	GO_CHEMOKINE_BIOSYNTHETIC_PROCESS	1	1.0138979	0.46361747
Upregulated Genes	GO_MULTI_ORGANISM_MEMBRANE_ORGANIZATION	1	1.0138428	0.48897797
Upregulated Genes	GO_MATING	2	1.0138143	0.45196506
Upregulated Genes	GO_REGULATION_OF_OSTEOBLAST_DIFFERENTIATION	1	1.0137162	0.47011954
Upregulated Genes	GO_POSITIVE_REGULATION_OF_SYNAPTIC_TRANSMISSION	2	1.0137159	0.45755693
Upregulated Genes	GO_REGULATION_OF_DENDRITE_DEVELOPMENT	2	1.0135671	0.45806453
Upregulated Genes	GO_POSITIVE_REGULATION_OF_INTEGRIN_ACTIVATION	1	1.0128667	0.48897797
Upregulated Genes	GO_REGULATION_OF_PEPTIDYL_SERINE_PHOSPHORYLATION	2	1.0123641	0.44251627
Upregulated Genes	GO_REGULATION_OF_CELL_ADHESION_MEDIATED_BY_INTEGRIN	1	1.0122979	0.47683397
Upregulated Genes	GO_REGULATION_OF_STRIATED_MUSCLE_CONTRACTION	2	1.0121615	0.44642857
Upregulated Genes	GO_LONG_TERM_SYNAPTIC_DEPRESSION	2	1.0120925	0.4576659
Upregulated Genes	GO_CENTRAL_NERVOUS_SYSTEM_NEURON_DEVELOPMENT	2	1.0120896	0.47098213
Upregulated Genes	GO_SKELETAL_MUSCLE_TISSUE_REGENERATION	2	1.0118268	0.4348786
Upregulated Genes	GO_NEGATIVE_REGULATION_OF_CARDIAC_MUSCLE_TISSUE_GROWTH	2	1.0114229	0.46966293
Upregulated Genes	GO_LYMPHOCYTE_ACTIVATION_INVOLVED_IN_IMMUNE_RESPONSE	8	1.011174	0.4400978
Upregulated Genes	GO_NEGATIVE_REGULATION_OF_CYTOKINE_BIOSYNTHETIC_PROCESS	1	1.0104038	0.47
Upregulated Genes	GO_ADULT_BEHAVIOR	2	1.0103385	0.45341614
Upregulated Genes	GO_ENDOCRINE_PANCREAS_DEVELOPMENT	1	1.0102674	0.47817838
Upregulated Genes	GO_NEGATIVE_REGULATION_OF_CELL_DEVELOPMENT	6	1.010103	0.3966346
Upregulated Genes	GO_REGULATION_OF_GLUCAGON_SECRETION	1	1.0100656	0.47826087
Upregulated Genes	GO_POSITIVE_REGULATION_OF_T_HELPER_2_CELL_CYTOKINE_PRODUCTION	1	1.0098996	0.48041236
Upregulated Genes	GO_NEGATIVE_REGULATION_OF_CYCLIN_DEPENDENT_PROTEIN_KINASE_ACTIVITY	2	1.0098904	0.4638298
Upregulated Genes	GO_TUMOR_NECROSIS_FACTOR_BIOSYNTHETIC_PROCESS	1	1.0097787	0.4810379
Upregulated Genes	GO_NEGATIVE_REGULATION_OF_TISSUE_REMODELING	1	1.0091966	0.47907948
Upregulated Genes	GO_NEGATIVE_REGULATION_OF_CHEMOKINE_PRODUCTION	1	1.0089028	0.48526523
Upregulated Genes	GO_NEGATIVE_REGULATION_OF_CARDIAC_MUSCLE_CELL_PROLIFERATION	2	1.0088714	0.46781117
Upregulated Genes	GO_NEGATIVE_REGULATION_OF_EPIDERMIS_DEVELOPMENT	2	1.0087932	0.44791666
Upregulated Genes	GO_CELLULAR_CARBOHYDRATE_METABOLIC_PROCESS	4	1.0087198	0.45396146
Upregulated Genes	GO_POSITIVE_REGULATION_OF_INTERLEUKIN_17_PRODUCTION	1	1.0087191	0.4750499

Upregulated Genes	GO_NEGATIVE_REGULATION_OF_INTERLEUKIN_1_MEDIATED_SIGNALING_PATHWAY	1	1.0082799	0.486
Upregulated Genes	GO_NEURON_CELLULAR_HOMEOSTASIS	1	1.008211	0.452
Upregulated Genes	GO_CYTOKINE_METABOLIC_PROCESS	1	1.0077602	0.47151276
Upregulated Genes	GO_POSITIVE_REGULATION_OF_ACUTE_INFLAMMATORY_RESPONSE	1	1.0076754	0.45891783
Upregulated Genes	GO_POSITIVE_REGULATION_OF_VASCULAR_ENDOTHELIAL_GROWTH_FACTOR_PRODUCTION	1	1.0074373	0.49278352
Upregulated Genes	GO_ACUTE_INFLAMMATORY_RESPONSE	1	1.0073513	0.48659003
Upregulated Genes	GO_ACUTE_PHASE_RESPONSE	1	1.0071589	0.482684
Upregulated Genes	GO_LONG_TERM_SYNAPTIC_POTENTIATION	2	1.006901	0.46943232
Upregulated Genes	GO_REGULATION_OF_LEUKOCYTE_ADHESION_TO_VASCULAR_ENDOTHELIAL_CELL	1	1.0068259	0.4875
Upregulated Genes	GO_NEGATIVE_REGULATION_OF_MUSCLE_TISSUE_DEVELOPMENT	2	1.0067326	0.45322245
Upregulated Genes	GO_POSITIVE_REGULATION_OF_PROTEIN_CATABOLIC_PROCESS	4	1.0065575	0.45190156
Upregulated Genes	GO_POSITIVE_REGULATION_OF_POSTTRANSCRIPTIONAL_GENE_SILENCING	1	1.006533	0.4894027
Upregulated Genes	GO_NEGATIVE_REGULATION_OF_RESPONSE_TO_CYTOKINE_STIMULUS	1	1.00652	0.4731801
Upregulated Genes	GO_POSITIVE_REGULATION_OF_PRODUCTION_OF_MIRNAS_INVOLVED_IN_GENE_SILENCING_B'	1	1.0063841	0.47173488
Upregulated Genes	GO_EPITHELIAL_CELL_DEVELOPMENT	8	1.0061795	0.4506024
Upregulated Genes	GO_NEGATIVE_REGULATION_OF_T_CELL_RECEPTOR_SIGNALING_PATHWAY	1	1.0061243	0.4861996
Upregulated Genes	GO_CELL_ADHESION_MEDIATED_BY_INTEGRIN	1	1.0057714	0.5
Upregulated Genes	GO_POSITIVE_REGULATION_OF_T_CELL_CYTOKINE_PRODUCTION	1	1.0055975	0.48676172
Upregulated Genes	GO_REGULATION_OF_VASCULAR_ENDOTHELIAL_GROWTH_FACTOR_RECEPTOR_SIGNALING_PA	1	1.0054936	0.48559672
Upregulated Genes	GO_ORGAN_OR_TISSUE_SPECIFIC_IMMUNE_RESPONSE	1	1.0052111	0.4967462
Upregulated Genes	GO_REGULATION_OF_INTERLEUKIN_1_MEDIATED_SIGNALING_PATHWAY	1	1.0050508	0.472
Upregulated Genes	GO_POSITIVE_REGULATION_OF_OSTEOBLAST_DIFFERENTIATION	1	1.0049953	0.51307845
Upregulated Genes	GO_NEGATIVE_REGULATION_OF_COLLAGEN_METABOLIC_PROCESS	1	1.0046958	0.45858586
Upregulated Genes	GO_ASTROCYTE_ACTIVATION	1	1.0045073	0.4990177
Upregulated Genes	GO_T_FOLLICULAR_HELPER_CELL_DIFFERENTIATION	1	1.0041221	0.49122807
Upregulated Genes	GO_MEMBRANE_FUSION	1	1.0041015	0.4942748
Upregulated Genes	GO_INTERLEUKIN_17_PRODUCTION	1	1.0039836	0.48362234
Upregulated Genes	GO_NEGATIVE_REGULATION_OF_ERK1_AND_ERK2_CASCADE	2	1.0038674	0.4845815
Upregulated Genes	GO_CD4_POSITIVE_OR_CD8_POSITIVE_ALPHA_BETA_T_CELL_LINEAGE_COMMITMENT	1	1.0037926	0.49516442
Upregulated Genes	GO_VASCULAR_ENDOTHELIAL_GROWTH_FACTOR_RECEPTOR_SIGNALING_PATHWAY	1	1.0037428	0.48041236
Upregulated Genes	GO_REGULATION_OF_T_HELPER_2_CELL_CYTOKINE_PRODUCTION	1	1.0036767	0.5057252
Upregulated Genes	GO_PANCREAS_DEVELOPMENT	1	1.0032939	0.4896694
Upregulated Genes	GO_POSITIVE_REGULATION_OF_TYPE_2_IMMUNE_RESPONSE	1	1.0030347	0.5
Upregulated Genes	GO_POSITIVE_REGULATION_OF_T_CELL_MEDIATED_IMMUNITY	1	1.0027076	0.49499
Upregulated Genes	GO_REGULATION_OF_SYNAPSE_STRUCTURE_OR_ACTIVITY	2	1.00264	0.4580777
Upregulated Genes	GO_NEGATIVE_REGULATION_OF_HYDROLASE_ACTIVITY	4	1.0025535	0.44103774
Upregulated Genes	GO_POSITIVE_REGULATION_OF_CELL_ADHESION_MEDIATED_BY_INTEGRIN	1	1.0023955	0.48732942
Upregulated Genes	GO_REGULATION_OF_NEURON_DIFFERENTIATION	11	1.0021319	0.44031832
Upregulated Genes	GO_POSITIVE_REGULATION_OF_LEUKOCYTE_ADHESION_TO_VASCULAR_ENDOTHELIAL_CELL	1	1.0020353	0.50751877
Upregulated Genes	GO_LIPID_METABOLIC_PROCESS	19	1.0020261	0.45776567
Upregulated Genes	GO_T_HELPER_CELL_LINEAGE_COMMITMENT	1	1.0020144	0.49284253
Upregulated Genes	GO_POSITIVE_REGULATION_OF_IMMUNOGLOBULIN_SECRETION	1	1.0019894	0.4827586
Upregulated Genes	GO_RESPONSE_TO_INTERLEUKIN_6	1	1.001751	0.4875
Upregulated Genes	GO_NEGATIVE_REGULATION_OF_ORGAN_GROWTH	2	1.0017334	0.4755102
Upregulated Genes	GO_ORGANIC_HYDROXY_COMPOUND_CATABOLIC_PROCESS	2	1.0016195	0.46680942
Upregulated Genes	GO_REGULATION_OF_TYPE_2_IMMUNE_RESPONSE	1	1.001616	0.4707113
Upregulated Genes	GO_T_HELPER_17_CELL_LINEAGE_COMMITMENT	1	1.00158	0.4970297
Upregulated Genes	GO_L_AMINO_ACID_TRANSPORT	1	1.0014547	0.5
Upregulated Genes	GO_LEUKOCYTE_ADHESION_TO_VASCULAR_ENDOTHELIAL_CELL	1	1.0013528	0.5049505
Upregulated Genes	GO_POSITIVE_REGULATION_OF_TUMOR_NECROSIS_FACTOR_BIOSYNTHETIC_PROCESS	1	1.0012835	0.47398844
Upregulated Genes	GO_METENCEPHALON_DEVELOPMENT	2	1.0010904	0.47239265
Upregulated Genes	GO_REGULATION_OF_TISSUE_REMODELING	1	1.0008582	0.49705306
Upregulated Genes	GO_NEGATIVE_REGULATION_OF_CARDIAC_MUSCLE_TISSUE_DEVELOPMENT	2	1.0007868	0.47204968
Upregulated Genes	GO_REGULATION_OF_ASTROCYTE_ACTIVATION	1	1.0007282	0.5010101
Upregulated Genes	GO_NEGATIVE_REGULATION_OF_PROTEIN_KINASE_B_SIGNALING	2	1.0006748	0.46136865
Upregulated Genes	GO_REGULATION_OF_T_CELL_RECEPTOR_SIGNALING_PATHWAY	1	1.0006158	0.49330783
Upregulated Genes	GO_TYPE_2_IMMUNE_RESPONSE	1	1.000416	0.49686846
Upregulated Genes	GO_INTERLEUKIN_1_MEDIATED_SIGNALING_PATHWAY	1	1.0002772	0.4846154
Upregulated Genes	GO_CARDIAC_CHAMBER_DEVELOPMENT	3	0.99994105	0.44920993
Upregulated Genes	GO_INTERLEUKIN_6_MEDIATED_SIGNALING_PATHWAY	1	0.9998994	0.5108911
Upregulated Genes	GO_INTERLEUKIN_21_PRODUCTION	1	0.99986446	0.514
Upregulated Genes	GO_REGULATION_OF_EXECUTION_PHASE_OF_APOPTOSIS	1	0.99981326	0.51703405
Upregulated Genes	GO_NEGATIVE_REGULATION_OF_LIPID_LOCALIZATION	1	0.99953514	0.50609756
Upregulated Genes	GO_INTERLEUKIN_2_PRODUCTION	2	0.9994909	0.47173914
Upregulated Genes	GO_POSITIVE_REGULATION_OF_APOPTOTIC_DNA_FRAGMENTATION	1	0.9991583	0.5089463
Upregulated Genes	GO_VASCULAR_ENDOTHELIAL_GROWTH_FACTOR_PRODUCTION	1	0.99913853	0.49224806
Upregulated Genes	GO_POSITIVE_REGULATION_OF_INTERLEUKIN_2_PRODUCTION	2	0.9989939	0.48240167
Upregulated Genes	GO_REGULATION_OF_ACTIN_FILAMENT_BASED_MOVEMENT	2	0.99893665	0.45852536
Upregulated Genes	GO_REGULATION_OF_BONE_REMODELING	1	0.9985646	0.5009862
Upregulated Genes	GO_SKIN_DEVELOPMENT	11	0.9985356	0.4292453

Upregulated Genes	GO_POSITIVE_REGULATION_OF_CELLULAR_COMPONENT_BIOGENESIS	14	0.998319	0.4368687
Upregulated Genes	GO_POSITIVE_REGULATION_OF_DEVELOPMENTAL_PROCESS	34	0.9983041	0.44094488
Upregulated Genes	GO_REGULATION_OF_T_CELL_CYTOKINE_PRODUCTION	1	0.99827546	0.5070994
Upregulated Genes	GO_T_TUBULE_ORGANIZATION	1	0.99803054	0.5114504
Upregulated Genes	GO_MATURE_B_CELL_DIFFERENTIATION	1	0.9977419	0.5140562
Upregulated Genes	GO_NEGATIVE_REGULATION_OF_ANTIGEN_RECEPTOR_MEDIATED_SIGNALING_PATHWAY	1	0.9973933	0.5
Upregulated Genes	GO_RESPONSE_TO_PEPTIDOGLYCAN	1	0.99722075	0.5216495
Upregulated Genes	GO_CARDIAC_SEPTUM_DEVELOPMENT	3	0.9971236	0.45054945
Upregulated Genes	GO_POSITIVE_REGULATION_OF_PEPTIDYL_SERINE_PHOSPHORYLATION	1	0.99649304	0.519337
Upregulated Genes	GO_ASTROCYTE_DEVELOPMENT	1	0.9964368	0.51129365
Upregulated Genes	GO_ALDITOL_METABOLIC_PROCESS	1	0.9960364	0.5041841
Upregulated Genes	GO_FACE_DEVELOPMENT	2	0.9956217	0.45969498
Upregulated Genes	GO_REGULATION_OF_POSTSYNAPSE_ORGANIZATION	2	0.99561536	0.48063782
Upregulated Genes	GO_REGULATION_OF_CELL_CELL_ADHESION_MEDIATED_BY_INTEGRIN	1	0.9955422	0.5048733
Upregulated Genes	GO_POSITIVE_REGULATION_OF_EPITHELIAL_CELL_APOPTOTIC_PROCESS	1	0.99553454	0.5131313
Upregulated Genes	GO_NEGATIVE_REGULATION_OF_LIPID_STORAGE	1	0.9954403	0.5079051
Upregulated Genes	GO_ANATOMICAL_STRUCTURE_ARRANGEMENT	1	0.995246	0.50363636
Upregulated Genes	GO_NEUTROPHIL_APOPTOTIC_PROCESS	1	0.9950136	0.49807692
Upregulated Genes	GO_REGULATION_OF_CATION_TRANSMEMBRANE_TRANSPORT	2	0.99492544	0.4859611
Upregulated Genes	GO_POSITIVE_REGULATION_OF_DNA_CATABOLIC_PROCESS	1	0.9943049	0.5194274
Upregulated Genes	GO_REGULATION_OF_TYPE_I_INTERFERON_MEDIATED_SIGNALING_PATHWAY	1	0.99414307	0.5102459
Upregulated Genes	GO_DICARBOXYLIC_ACID_TRANSPORT	1	0.9941315	0.48832685
Upregulated Genes	GO_NEGATIVE_REGULATION_OF_ACTIVATED_T_CELL_PROLIFERATION	1	0.99369913	0.4950884
Upregulated Genes	GO_MATURE_B_CELL_DIFFERENTIATION_INVOLVED_IN_IMMUNE_RESPONSE	1	0.9935176	0.53386456
Upregulated Genes	GO_ACETYL_COA_METABOLIC_PROCESS	1	0.99337023	0.5019455
Upregulated Genes	GO_REGULATION_OF_COLLAGEN_METABOLIC_PROCESS	1	0.993169	0.50509167
Upregulated Genes	GO_T_CELL_LINEAGE_COMMITMENT	1	0.9923403	0.53306615
Upregulated Genes	GO_NUCLEOSIDE_BISPHOSPHATE_BIOSYNTHETIC_PROCESS	1	0.992143	0.49007937
Upregulated Genes	GO_PRIMARY_MIRNA_PROCESSING	1	0.9916046	0.49333334
Upregulated Genes	GO_NUCLEOSIDE_BISPHOSPHATE_METABOLIC_PROCESS	1	0.99155414	0.5030675
Upregulated Genes	GO_T_HELPER_2_CELL_CYTOKINE_PRODUCTION	1	0.9914542	0.53521127
Upregulated Genes	GO_ACIDIC_AMINO_ACID_TRANSPORT	1	0.99126345	0.51975054
Upregulated Genes	GO_NEGATIVE_REGULATION_OF_SYNAPTIC_TRANSMISSION	2	0.991177	0.49353448
Upregulated Genes	GO_L_GLUTAMATE_TRANSMEMBRANE_TRANSPORT	1	0.9907034	0.51140684
Upregulated Genes	GO_T_CELL_CYTOKINE_PRODUCTION	1	0.9906368	0.5324948
Upregulated Genes	GO_PLASMA_MEMBRANE_REPAIR	1	0.99041235	0.51195216
Upregulated Genes	GO_LIPOPOLYSACCHARIDE_MEDIATED_SIGNALING_PATHWAY	1	0.9902793	0.5080972
Upregulated Genes	GO_POSITIVE_REGULATION_OF_EXECUTION_PHASE_OF_APOPTOSIS	1	0.99003875	0.53333336
Upregulated Genes	GO_NEGATIVE_REGULATION_OF_BONE_REMODELING	1	0.9893188	0.53091687
Upregulated Genes	GO_FACIAL_NERVE_STRUCTURAL_ORGANIZATION	1	0.98919725	0.49596775
Upregulated Genes	GO_REGULATION_OF_CELLULAR_PROTEIN_CATABOLIC_PROCESS	4	0.9889682	0.4568182
Upregulated Genes	GO_NK_T_CELL_PROLIFERATION	1	0.9877825	0.5056391
Upregulated Genes	GO_POSITIVE_REGULATION_OF_TYPE_I_INTERFERON_MEDIATED_SIGNALING_PATHWAY	1	0.98665243	0.5237192
Upregulated Genes	GO_MACROPHAGE_APOPTOTIC_PROCESS	1	0.98609036	0.5254902
Upregulated Genes	GO_NATURAL_KILLER_CELL_ACTIVATION	1	0.98601437	0.52182955
Upregulated Genes	GO_REGULATION_OF_CALCIUM_ION_TRANSPORT	2	0.98599327	0.48893806
Upregulated Genes	GO_NATURAL_KILLER_CELL_PROLIFERATION	1	0.9853195	0.5214724
Upregulated Genes	GO_NK_T_CELL_ACTIVATION	1	0.9852395	0.50619835
Upregulated Genes	GO_FATTY_ACYL_COA_BIOSYNTHETIC_PROCESS	1	0.98503697	0.5103735
Upregulated Genes	GO_REGULATION_OF_DNA_CATABOLIC_PROCESS	1	0.98482114	0.545809
Upregulated Genes	GO_BRANCHIOMOTOR_NEURON_AXON_GUIDANCE	1	0.98416984	0.52191234
Upregulated Genes	GO_POSITIVE_REGULATION_OF_OXIDOREDUCTASE_ACTIVITY	2	0.9837075	0.50209206
Upregulated Genes	GO_TRIGEMINAL_NERVE_MORPHOGENESIS	1	0.98365295	0.49609375
Upregulated Genes	GO_ENDOCRINE_SYSTEM_DEVELOPMENT	1	0.9835829	0.5481336
Upregulated Genes	GO_REGULATION_OF_ACUTE_INFLAMMATORY_RESPONSE	1	0.98293984	0.5294118
Upregulated Genes	GO_L_ALPHA_AMINO_ACID_TRANSMEMBRANE_TRANSPORT	1	0.9828436	0.5483871
Upregulated Genes	GO_REGULATION_OF_TRANSMEMBRANE_TRANSPORT	5	0.9818821	0.44097996
Upregulated Genes	GO_REGULATION_OF_CARDIAC_MUSCLE_CONTRACTION	2	0.9813433	0.4845815
Upregulated Genes	GO_THIOESTER_METABOLIC_PROCESS	1	0.9807156	0.52674896
Upregulated Genes	GO_POSITIVE_REGULATION_OF_CILIUM_ASSEMBLY	1	0.980442	0.51008064
Upregulated Genes	GO_POSITIVE_REGULATION_OF_REGULATORY_T_CELL_DIFFERENTIATION	1	0.98034555	0.5557769
Upregulated Genes	GO_CRANIAL_NERVE_MORPHOGENESIS	1	0.9786187	0.5298969
Upregulated Genes	GO_BODY_MORPHOGENESIS	2	0.978432	0.49896908
Upregulated Genes	GO_MACROMOLECULE_DEACYLATION	2	0.9783002	0.5075922
Upregulated Genes	GO_REGULATION_OF_CHOLESTEROL_METABOLIC_PROCESS	1	0.97812766	0.5248509
Upregulated Genes	GO_REGULATION_OF_STEROID_METABOLIC_PROCESS	1	0.9771029	0.53740156
Upregulated Genes	GO_DENDRITE_DEVELOPMENT	4	0.976735	0.45934066
Upregulated Genes	GO_RESPONSE_TO_NITROGEN_COMPOUND	26	0.97627026	0.48
Upregulated Genes	GO_ETHER_BIOSYNTHETIC_PROCESS	1	0.97625333	0.55212355
Upregulated Genes	GO_LYSOSOME_LOCALIZATION	2	0.9760215	0.5076253

Upregulated Genes	GO_FACIAL_NERVE_MORPHOGENESIS	1	0.97558224	0.5289421
Upregulated Genes	GO_SEMAPHORIN_PLEXIN_SIGNALING_PATHWAY_INVOLVED_IN_NEURON_PROJECTION_GUIDA	1	0.97503734	0.5251938
Upregulated Genes	GO_REGULATION_OF_TRANSPORTER_ACTIVITY	2	0.9749934	0.5105042
Upregulated Genes	GO_LEFT_RIGHT_PATTERN_FORMATION	1	0.97472703	0.5102041
Upregulated Genes	GO_AUTONOMIC_NERVOUS_SYSTEM_DEVELOPMENT	1	0.9741517	0.5498008
Upregulated Genes	GO_CIRCULATORY_SYSTEM_DEVELOPMENT	17	0.97376406	0.48704663
Upregulated Genes	GO_POSITIVE_REGULATION_OF_RNA_BIOSYNTHETIC_PROCESS	26	0.97322816	0.48324022
Upregulated Genes	GO_ETHER_METABOLIC_PROCESS	1	0.973182	0.53688526
Upregulated Genes	GO_HEAD_MORPHOGENESIS	2	0.9728313	0.5264271
Upregulated Genes	GO_RESPONSE_TO_TYPE_I_INTERFERON	1	0.97279644	0.545082
Upregulated Genes	GO_THIOESTER_BIOSYNTHETIC_PROCESS	1	0.9727069	0.5313175
Upregulated Genes	GO_POSITIVE_REGULATION_OF_NEURON_DIFFERENTIATION	8	0.97234094	0.46135265
Upregulated Genes	GO_REGULATION_OF_STEROID_BIOSYNTHETIC_PROCESS	1	0.972166	0.5479452
Upregulated Genes	GO_RESPONSE_TO_STEROID_HORMONE	16	0.97182316	0.4691689
Upregulated Genes	GO_POSITIVE_REGULATION_OF_CELLULAR_PROTEIN_CATABOLIC_PROCESS	4	0.9712677	0.46818182
Upregulated Genes	GO_SYNAPTIC_VESICLE_LOCALIZATION	2	0.97112286	0.49772727
Upregulated Genes	GO_REGULATION_OF_ALCOHOL_BIOSYNTHETIC_PROCESS	1	0.9709597	0.546371
Upregulated Genes	GO_ESTABLISHMENT_OF_ENDOTHELIAL_INTESTINAL_BARRIER	1	0.97061133	0.5489022
Upregulated Genes	GO_SKELETAL_SYSTEM_MORPHOGENESIS	5	0.97010946	0.49289098
Upregulated Genes	GO_ENDOTHELIAL_CELL_MIGRATION	5	0.9696159	0.4701835
Upregulated Genes	GO_REGULATION_OF_CHOLESTEROL_BIOSYNTHETIC_PROCESS	1	0.9691424	0.5616162
Upregulated Genes	GO_FATTY_ACYL_COA_METABOLIC_PROCESS	1	0.9686197	0.5487078
Upregulated Genes	GO_CALCIUM_ION_TRANSMEMBRANE_TRANSPORT	2	0.96842045	0.52025586
Upregulated Genes	GO_REGULATION_OF_CELL_SHAPE	1	0.9679733	0.5436893
Upregulated Genes	GO_REGULATION_OF_CELL_MATRIX_ADHESION	2	0.9671338	0.51077586
Upregulated Genes	GO_CARBOHYDRATE_TRANSPORT	2	0.9663197	0.50109893
Upregulated Genes	GO_REGULATION_OF_ACTOMYOSIN_STRUCTURE_ORGANIZATION	3	0.96575934	0.50440526
Upregulated Genes	GO_VESICLE_CYTOSKELETAL_TRAFFICKING	2	0.9655734	0.49895614
Upregulated Genes	GO_TRIGEMINAL_NERVE_DEVELOPMENT	1	0.9653778	0.52469134
Upregulated Genes	GO_GLYCEROL_ETHER_METABOLIC_PROCESS	1	0.9639981	0.5690722
Upregulated Genes	GO_GLANDULAR_EPITHELIAL_CELL_DEVELOPMENT	1	0.9639264	0.5495868
Upregulated Genes	GO_CRANIAL_NERVE_DEVELOPMENT	1	0.9633803	0.54747474
Upregulated Genes	GO_PARASYMPATHETIC_NERVOUS_SYSTEM_DEVELOPMENT	1	0.9628913	0.54780877
Upregulated Genes	GO_RESPONSE_TO_VITAMIN_A	3	0.9628815	0.4885321
Upregulated Genes	GO_CRANIAL_NERVE_STRUCTURAL_ORGANIZATION	1	0.9624297	0.560166
Upregulated Genes	GO_PEPTIDYL_AMINO_ACID_MODIFICATION	21	0.96188796	0.5027174
Upregulated Genes	GO_VESICLE_TRANSPORT_ALONG_MICROTUBULE	2	0.9618333	0.53594774
Upregulated Genes	GO_TRANSPORT_ALONG_MICROTUBULE	2	0.96179575	0.51875
Upregulated Genes	GO_CYTOSKELETON_DEPENDENT_INTRACELLULAR_TRANSPORT	2	0.9617782	0.50840336
Upregulated Genes	GO_REGULATION_OF_OXIDOREDUCTASE_ACTIVITY	3	0.95827985	0.5125285
Upregulated Genes	GO_PROTEIN_LOCALIZATION_TO_MICROTUBULE_END	1	0.9581148	0.5681342
Upregulated Genes	GO_LEFT_RIGHT_AXIS_SPECIFICATION	1	0.9576527	0.58483034
Upregulated Genes	GO_POSITIVE_REGULATION_OF_MICROTUBULE_BINDING	1	0.95744735	0.54844964
Upregulated Genes	GO_CARBOHYDRATE_DERIVATIVE_CATABOLIC_PROCESS	3	0.9572164	0.49779737
Upregulated Genes	GO_POSITIVE_REGULATION_OF_PROTEIN_BINDING	1	0.95641965	0.56626505
Upregulated Genes	GO_SENSORY_PERCEPTION_OF_MECHANICAL_STIMULUS	3	0.9560229	0.5127021
Upregulated Genes	GO_REGULATION_OF_MAPK_CASCADE	17	0.9554058	0.50377834
Upregulated Genes	GO_POSITIVE_REGULATION_OF_PROTEIN_CONTAINING_COMPLEX_ASSEMBLY	11	0.9548043	0.5194805
Upregulated Genes	GO_POSITIVE_REGULATION_OF_CYCLIN_DEPENDENT_PROTEIN_KINASE_ACTIVITY	1	0.9520233	0.55452865
Upregulated Genes	GO_POSITIVE_REGULATION_OF_AXON_EXTENSION	1	0.9514474	0.58449304
Upregulated Genes	GO_PYRAMIDAL_NEURON_DIFFERENTIATION	1	0.9514415	0.59574467
Upregulated Genes	GO_FATTY_ACID_DERIVATIVE_CATABOLIC_PROCESS	1	0.94989586	0.5626283
Upregulated Genes	GO_CELLULAR_RESPONSE_TO_CALCIUM_ION	3	0.9498479	0.5146067
Upregulated Genes	GO_PHYLLOQUINONE_METABOLIC_PROCESS	1	0.94744647	0.5836502
Upregulated Genes	GO_FOREBRAIN_NEURON_DEVELOPMENT	1	0.94725776	0.59839356
Upregulated Genes	GO_NUCLEAR_TRANSCRIBED_MRNA_CATABOLIC_PROCESS_NONSENSE_MEDIATED_DECAY	1	0.9463572	0.55419225
Upregulated Genes	GO_ORGANELLE_TRANSPORT_ALONG_MICROTUBULE	2	0.9461359	0.55179703
Upregulated Genes	GO_POSITIVE_REGULATION_OF_TRANSLATION	2	0.94607615	0.5828092
Upregulated Genes	GO_REGULATION_OF_MICROTUBULE_BINDING	1	0.9460137	0.5779093
Upregulated Genes	GO_LEUKOTRIENE_B4_METABOLIC_PROCESS	1	0.9453619	0.56875
Upregulated Genes	GO_MENAQUINONE_METABOLIC_PROCESS	1	0.9440559	0.5860656
Upregulated Genes	GO_POSITIVE_REGULATION_OF_CELLULAR_AMIDE_METABOLIC_PROCESS	2	0.9424408	0.5545657
Upregulated Genes	GO_RNA_DECAPPING	1	0.9419056	0.5753138
Upregulated Genes	GO_REGULATION_OF_CD4_POSITIVE_ALPHA_BETA_T_CELL_DIFFERENTIATION	3	0.94173825	0.5295405
Upregulated Genes	GO_POSITIVE_REGULATION_OF_CELL_GROWTH	1	0.9408343	0.58185405
Upregulated Genes	GO_KIDNEY_MORPHOGENESIS	2	0.9407857	0.5543478
Upregulated Genes	GO_REGULATION_OF_EPITHELIAL_CELL_MIGRATION	5	0.9402633	0.49403343
Upregulated Genes	GO_ARACHIDONIC_ACID_METABOLIC_PROCESS	1	0.93961096	0.5945946
Upregulated Genes	GO_KETONE_CATABOLIC_PROCESS	1	0.93913305	0.6230159
Upregulated Genes	GO_POLYOL_METABOLIC_PROCESS	3	0.9387584	0.53555554

Upregulated Genes	GO_ICOSANOID_CATABOLIC_PROCESS	1	0.938734	0.5805169
Upregulated Genes	GO_LEUKOTRIENE_METABOLIC_PROCESS	1	0.93784374	0.58773786
Upregulated Genes	GO_NUCLEAR_TRANSCRIBED_MRNA_CATABOLIC_PROCESS_DEADENYLATION_INDEPENDENT_D	1	0.93778217	0.5683168
Upregulated Genes	GO_RNA_CATABOLIC_PROCESS	4	0.9358815	0.556582
Upregulated Genes	GO_RESPIRATORY_SYSTEM_DEVELOPMENT	3	0.93584394	0.5404762
Upregulated Genes	GO_NEGATIVE_REGULATION_OF_CELLULAR_COMPONENT_MOVEMENT	10	0.9353717	0.52955663
Upregulated Genes	GO_FATTY_ACID_CATABOLIC_PROCESS	1	0.93458265	0.6023857
Upregulated Genes	GO_CARBOHYDRATE_METABOLIC_PROCESS	8	0.9339295	0.5155131
Upregulated Genes	GO_ORGANIC_ACID_METABOLIC_PROCESS	19	0.9334437	0.5186104
Upregulated Genes	GO_VITAMIN_K_METABOLIC_PROCESS	1	0.9329242	0.60749507
Upregulated Genes	GO_REGULATION_OF_HAIR_CYCLE	2	0.93197334	0.52609605
Upregulated Genes	GO_POSITIVE_REGULATION_OF_NUCLEAR_CELL_CYCLE_DNA_REPLICATION	1	0.9302393	0.5797101
Upregulated Genes	GO_METANEPHROS_DEVELOPMENT	2	0.92946017	0.55402297
Upregulated Genes	GO_LONG_CHAIN_FATTY_ACID_CATABOLIC_PROCESS	1	0.9279388	0.61054766
Upregulated Genes	GO_NUCLEOBASE_CONTAINING_COMPOUND_TRANSPORT	3	0.9270676	0.5465587
Upregulated Genes	GO_PATTERN_SPECIFICATION_PROCESS	9	0.92699075	0.5177665
Upregulated Genes	GO_REGULATION_OF_VIRAL_TRANSCRIPTION	1	0.92559963	0.60115606
Upregulated Genes	GO_B_CELL_DIFFERENTIATION	4	0.9250858	0.5406032
Upregulated Genes	GO_NEGATIVE_REGULATION_OF_VIRAL_TRANSCRIPTION	1	0.9250617	0.6136364
Upregulated Genes	GO_REGULATION_OF_CYSTEINE_TYPE_ENDOPEPTIDASE_ACTIVITY_INVOLVED_IN_APOPTOTIC_S	1	0.92464375	0.6189555
Upregulated Genes	GO_REGULATION_OF_PEPTIDASE_ACTIVITY	4	0.92441183	0.54588234
Upregulated Genes	GO_REGULATION_OF_MEGAKARYOCYTE_DIFFERENTIATION	3	0.9237518	0.5508475
Upregulated Genes	GO_PURINE_CONTAINING_COMPOUND_METABOLIC_PROCESS	8	0.92265445	0.51860464
Upregulated Genes	GO_CELL_ACTIVATION_INVOLVED_IN_IMMUNE_RESPONSE	13	0.9223062	0.535109
Upregulated Genes	GO_ROOF_OF_MOUTH_DEVELOPMENT	2	0.92065215	0.5560254
Upregulated Genes	GO_ALCOHOL_BIOSYNTHETIC_PROCESS	3	0.9204128	0.57939917
Upregulated Genes	GO_NEGATIVE_REGULATION_OF_VIRAL_PROCESS	1	0.92008334	0.60946745
Upregulated Genes	GO_RESPONSE_TO_HEAT	3	0.9200689	0.53775746
Upregulated Genes	GO_REGULATION_OF_MUSCLE_CELL_DIFFERENTIATION	2	0.919319	0.56057495
Upregulated Genes	GO_REGULATION_OF_SMALL_MOLECULE_METABOLIC_PROCESS	3	0.9181342	0.5273109
Upregulated Genes	GO_POSITIVE_REGULATION_OF_CYSTEINE_TYPE_ENDOPEPTIDASE_ACTIVITY_INVOLVED_IN_APO	1	0.91768837	0.61811024
Upregulated Genes	GO_CELLULAR_RESPONSE_TO_KETONE	5	0.9175473	0.5407925
Upregulated Genes	GO_POSITIVE_REGULATION_OF_VIRAL_TRANSCRIPTION	1	0.9162034	0.6012024
Upregulated Genes	GO_GENE_SILENCING	8	0.915638	0.5353774
Upregulated Genes	GO_NEGATIVE_REGULATION_OF_DNA_BINDING	1	0.9156315	0.640873
Upregulated Genes	GO_NEGATIVE_REGULATION_OF_CELL_DIFFERENTIATION	14	0.9154105	0.5561224
Upregulated Genes	GO_RIBONUCLEOSIDE_MONOPHOSPHATE_METABOLIC_PROCESS	1	0.9152878	0.60078275
Upregulated Genes	GO_REGULATION_OF_STRIATED_MUSCLE_CELL_DIFFERENTIATION	2	0.9146745	0.554371
Upregulated Genes	GO_5_PHOSPHORIBOSE_1_DIPHOSPHATE_METABOLIC_PROCESS	1	0.9144533	0.6392157
Upregulated Genes	GO_NEGATIVE_REGULATION_BY_HOST_OF_VIRAL_TRANSCRIPTION	1	0.9138241	0.6227898
Upregulated Genes	GO_POSITIVE_REGULATION_BY_HOST_OF_VIRAL_TRANSCRIPTION	1	0.9127673	0.61935484
Upregulated Genes	GO_REGULATION_OF_PROTEIN_LOCALIZATION_TO_CHROMOSOME_TELOMERIC_REGION	1	0.91099447	0.60488796
Upregulated Genes	GO_RIBONUCLEOSIDE_MONOPHOSPHATE_BIOSYNTHETIC_PROCESS	1	0.91016984	0.63508064
Upregulated Genes	GO_POSITIVE_REGULATION_OF_CELLULAR_COMPONENT_MOVEMENT	14	0.9083076	0.57608694
Upregulated Genes	GO_STRESS_ACTIVATED_PROTEIN_KINASE_SIGNALING_CASCADE	5	0.9081354	0.53186274
Upregulated Genes	GO_NEGATIVE_REGULATION_OF_BMP_SIGNALING_PATHWAY	1	0.90784043	0.6389452
Upregulated Genes	GO_REGULATION_OF_ORGANELLE_ASSEMBLY	5	0.90776217	0.53391683
Upregulated Genes	GO_BIOTIN_METABOLIC_PROCESS	1	0.9072436	0.6307054
Upregulated Genes	GO_POSITIVE_REGULATION_BY_HOST_OF_VIRAL_PROCESS	1	0.9068589	0.63582677
Upregulated Genes	GO_GLIAL_CELL_DEVELOPMENT	3	0.9061413	0.5916473
Upregulated Genes	GO_ORGANIC_HYDROXY_COMPOUND_BIOSYNTHETIC_PROCESS	4	0.9061139	0.55452436
Upregulated Genes	GO_LIPOPROTEIN_CATABOLIC_PROCESS	1	0.90608454	0.6316794
Upregulated Genes	GO_NEGATIVE_REGULATION_OF_GOLGI_TO_PLASMA_MEMBRANE_PROTEIN_TRANSPORT	1	0.9058109	0.6385542
Upregulated Genes	GO_MONOSACCHARIDE_BIOSYNTHETIC_PROCESS	1	0.9049751	0.6452282
Upregulated Genes	GO_BONE_GROWTH	3	0.9045391	0.58099353
Upregulated Genes	GO_REGULATION_OF_NERVOUS_SYSTEM_PROCESS	3	0.90447277	0.588
Upregulated Genes	GO_MEGAKARYOCYTE_DIFFERENTIATION	3	0.9042488	0.59151787
Upregulated Genes	GO_INNER_MITOCHONDRIAL_MEMBRANE_ORGANIZATION	1	0.90080947	0.6392405
Upregulated Genes	GO_POSITIVE_REGULATION_OF_PROTEOLYSIS_INVOLVED_IN_CELLULAR_PROTEIN_CATABOLIC_	3	0.9006562	0.57589287
Upregulated Genes	GO_NEGATIVE_REGULATION_OF_DNA_DEPENDENT_DNA_REPLICATION	1	0.89998686	0.67346936
Upregulated Genes	GO_SYNCYTIUM_FORMATION	2	0.89981717	0.6103093
Upregulated Genes	GO_PROTEIN_LOCALIZATION_TO_CHROMOSOME_TELOMERIC_REGION	1	0.89931226	0.61960787
Upregulated Genes	GO_MODULATION_BY_HOST_OF_VIRAL_PROCESS	1	0.8991428	0.6306483
Upregulated Genes	GO_GROWTH_PLATE_CARTILAGE_DEVELOPMENT	3	0.89899576	0.5912088
Upregulated Genes	GO_VIRION_ASSEMBLY	1	0.8983614	0.63178295
Upregulated Genes	GO_PHOSPHATIDIC_ACID_METABOLIC_PROCESS	2	0.89822686	0.62255967
Upregulated Genes	GO_REGULATION_OF_IMMUNE_EFFECTOR_PROCESS	9	0.8981426	0.5798526
Upregulated Genes	GO_REGULATION_OF_ESTABLISHMENT_OF_PROTEIN_LOCALIZATION_TO_CHROMOSOME	1	0.8973013	0.6569201
Upregulated Genes	GO_MYOBLAST_FUSION	2	0.8971874	0.6145833
Upregulated Genes	GO_GOLGI_TO_PLASMA_MEMBRANE_TRANSPORT	1	0.89681256	0.6547619

Upregulated Genes	GO_RNA_PHOSPHODIESTER_BOND_HYDROLYSIS_ENDONUCLEOLYTIC	2	0.89679223	0.6132723
Upregulated Genes	GO_MICROTUBULE_ORGANIZING_CENTER_LOCALIZATION	2	0.89493644	0.6345733
Upregulated Genes	GO_ENSHEATHMENT_OF_NEURONS	3	0.8947402	0.6004619
Upregulated Genes	GO_VIRAL_RELEASE_FROM_HOST_CELL	1	0.89433044	0.64356434
Upregulated Genes	GO_CHROMOSOME_LOCALIZATION_TO_NUCLEAR_ENVELOPE_INVOLVED_IN_HOMOLOGOUS_C	1	0.8942512	0.6686747
Upregulated Genes	GO_SPERM_MOTILITY	2	0.8941584	0.61027837
Upregulated Genes	GO_CELLULAR_RESPONSE_TO_HEAT	3	0.89367926	0.60126585
Upregulated Genes	GO_CILIUM_OR_FLAGELLUM_DEPENDENT_CELL_MOTILITY	2	0.89340067	0.5975104
Upregulated Genes	GO_NEGATIVE_REGULATION_OF_NUCLEASE_ACTIVITY	1	0.8931307	0.6515748
Upregulated Genes	GO_GLIAL_CELL_PROLIFERATION	3	0.8930186	0.589404
Upregulated Genes	GO_POSITIVE_REGULATION_OF_UBIQUITIN_DEPENDENT_PROTEIN_CATABOLIC_PROCESS	2	0.8912123	0.59381896
Upregulated Genes	GO_MEMORY	2	0.8898854	0.6066667
Upregulated Genes	GO_POSITIVE_REGULATION_OF_VIRAL_RELEASE_FROM_HOST_CELL	1	0.8894673	0.6639676
Upregulated Genes	GO_NEGATIVE_REGULATION_OF_PROTEIN_CONTAINING_COMPLEX_ASSEMBLY	6	0.8891377	0.5690867
Upregulated Genes	GO_POST_GOLGI_VESICLE_MEDIATED_TRANSPORT	1	0.8889926	0.6608187
Upregulated Genes	GO_ESTABLISHMENT_OF_PROTEIN_LOCALIZATION_TO_CHROMOSOME	1	0.88865465	0.67657995
Upregulated Genes	GO_ESTABLISHMENT_OF_PROTEIN_LOCALIZATION_TO_TELOMERE	1	0.8885327	0.6533066
Upregulated Genes	GO_OLIGODENDROCYTE_DIFFERENTIATION	3	0.8875527	0.6084071
Upregulated Genes	GO_LIPOPROTEIN_METABOLIC_PROCESS	1	0.8867477	0.66260165
Upregulated Genes	GO_WATER_SOLUBLE_VITAMIN_METABOLIC_PROCESS	5	0.88618594	0.55803573
Upregulated Genes	GO_NEGATIVE_REGULATION_OF_DNA_REPLICATION	1	0.88616985	0.6757322
Upregulated Genes	GO_CELL_CHEMOTAXIS	7	0.8856719	0.53579676
Upregulated Genes	GO_MICROTUBULE_NUCLEATION	1	0.8849284	0.6817326
Upregulated Genes	GO_MACROMOLECULE_DEPALMITOYLATION	1	0.8845458	0.64796907
Upregulated Genes	GO_GOLGI_TO_PLASMA_MEMBRANE_PROTEIN_TRANSPORT	1	0.88422245	0.6632017
Upregulated Genes	GO_KERATINOCYTE_DEVELOPMENT	2	0.8838619	0.6130435
Upregulated Genes	GO_POSITIVE_REGULATION_OF_LYMPHOCYTE_ACTIVATION	8	0.88326603	0.6051402
Upregulated Genes	GO_ORGANELLE_ASSEMBLY	22	0.8825747	0.6296296
Upregulated Genes	GO_PRENYLATION	1	0.88209504	0.6666667
Upregulated Genes	GO_RESPONSE_TO_HORMONE	24	0.88173866	0.6124031
Upregulated Genes	GO_VESICLE_MEDIATED_TRANSPORT_TO_THE_PLASMA_MEMBRANE	1	0.8804412	0.6877395
Upregulated Genes	GO_NEGATIVE_REGULATION_OF_PROTEIN_LOCALIZATION_TO_CELL_PERIPHERY	1	0.8801931	0.69201523
Upregulated Genes	GO_PROTEIN_DEPALMITOYLATION	1	0.87993526	0.6814516
Upregulated Genes	GO_CILIUM_MOVEMENT	2	0.87986857	0.61388284
Upregulated Genes	GO_CENTROSOME_SEPARATION	1	0.87969035	0.687251
Upregulated Genes	GO_REGULATION_OF_GOLGI_TO_PLASMA_MEMBRANE_PROTEIN_TRANSPORT	1	0.8795673	0.6807229
Upregulated Genes	GO_REGULATION_OF_UBIQUITIN_DEPENDENT_PROTEIN_CATABOLIC_PROCESS	2	0.87935597	0.639485
Upregulated Genes	GO_ESTABLISHMENT_OF_PROTEIN_LOCALIZATION_TO_PLASMA_MEMBRANE	1	0.87858534	0.6826923
Upregulated Genes	GO_DEVELOPMENTAL_CELL_GROWTH	4	0.8777408	0.6331878
Upregulated Genes	GO_REGULATION_OF_MULTI_ORGANISM_PROCESS	2	0.8773345	0.67157894
Upregulated Genes	GO_INACTIVATION_OF_MAPK_ACTIVITY	1	0.8769982	0.6679612
Upregulated Genes	GO_NUCLEOBASE_CONTAINING_SMALL_MOLECULE_BIOSYNTHETIC_PROCESS	3	0.8760474	0.64801866
Upregulated Genes	GO_REGULATION_OF_MICROTUBULE_MOTOR_ACTIVITY	1	0.8757126	0.6860707
Upregulated Genes	GO_PROTEIN_GERANYLGERANYLATION	1	0.8744106	0.6781818
Upregulated Genes	GO_PHOSPHOLIPID_BIOSYNTHETIC_PROCESS	4	0.8708677	0.60045147
Upregulated Genes	GO_CELLULAR_HORMONE_METABOLIC_PROCESS	3	0.87065667	0.6316964
Upregulated Genes	GO_GLYCEROPHOSPHOLIPID_METABOLIC_PROCESS	4	0.8705617	0.61587983
Upregulated Genes	GO_PROTEIN_LOCALIZATION_TO_ADHERENS_JUNCTION	1	0.87003285	0.6843177
Upregulated Genes	GO_UROGENITAL_SYSTEM_DEVELOPMENT	6	0.86940247	0.6019656
Upregulated Genes	GO_EPITHELIAL_CELL_MORPHOGENESIS	2	0.8680738	0.6361746
Upregulated Genes	GO_POSITIVE_REGULATION_OF_TELOMERE_MAINTENANCE_VIA_TELOMERE_LENGTHENING	1	0.8666767	0.6776699
Upregulated Genes	GO_KERATINOCYTE_PROLIFERATION	2	0.8651258	0.6551724
Upregulated Genes	GO_GLYCEROLIPID_METABOLIC_PROCESS	4	0.86385244	0.64047617
Upregulated Genes	GO_SYNAPSE_ORGANIZATION	5	0.8630562	0.61725664
Upregulated Genes	GO_LEUKOCYTE_MEDIATED_CYTOTOXICITY	2	0.8622434	0.6204691
Upregulated Genes	GO_GLYCEROLIPID_BIOSYNTHETIC_PROCESS	4	0.86195385	0.6244444
Upregulated Genes	GO_CELL_CELL_JUNCTION_ASSEMBLY	4	0.8614955	0.64568764
Upregulated Genes	GO_P38MAPK_CASCADE	1	0.861466	0.68821293
Upregulated Genes	GO_HORMONE_METABOLIC_PROCESS	4	0.8611956	0.6355353
Upregulated Genes	GO_SMOOTHENED_SIGNALING_PATHWAY	4	0.8602895	0.5995526
Upregulated Genes	GO_NUCLEOSIDE_METABOLIC_PROCESS	3	0.8596774	0.61171365
Upregulated Genes	GO_POSITIVE_REGULATION_OF_TELOMERASE_ACTIVITY	1	0.8596758	0.6988189
Upregulated Genes	GO_CELL_KILLING	2	0.8572028	0.65296805
Upregulated Genes	GO_DEFENSE_RESPONSE_TO_BACTERIUM	4	0.8571367	0.62053573
Upregulated Genes	GO_REGULATION_OF_TELOMERE_CAPPING	1	0.85598934	0.734
Upregulated Genes	GO_RESPONSE_TO_UV_C	1	0.85587436	0.70731705
Upregulated Genes	GO_MALE_SEX_DETERMINATION	1	0.85531914	0.68421054
Upregulated Genes	GO_POSITIVE_REGULATION_OF_TELOMERE_MAINTENANCE	1	0.855232	0.71057886
Upregulated Genes	GO_MEMBRANOUS_SEPTUM_MORPHOGENESIS	2	0.8544708	0.6688453
Upregulated Genes	GO_SEX_DETERMINATION	1	0.8535105	0.71659917

Upregulated Genes	GO_MATERNAL_PROCESS_INVOLVED_IN_FEMALE_PREGNANCY	2	0.84909546	0.66875
Upregulated Genes	GO_CARBOHYDRATE_BIOSYNTHETIC_PROCESS	2	0.84893245	0.6696231
Upregulated Genes	GO_POSITIVE_REGULATION_OF_MACROAUTOPHAGY	1	0.84881127	0.7271062
Upregulated Genes	GO_POSITIVE_REGULATION_OF_P38MAPK_CASCADE	1	0.8462546	0.7538168
Upregulated Genes	GO_VENTRICULAR_SEPTUM_MORPHOGENESIS	2	0.84542716	0.6952191
Upregulated Genes	GO_ENZYME_LINKED_RECEPTOR_PROTEIN_SIGNALING_PATHWAY	14	0.84451157	0.6467662
Upregulated Genes	GO_POSITIVE_REGULATION_OF_TELOMERE_CAPPING	1	0.8434862	0.7475915
Upregulated Genes	GO_CARDIAC_VENTRICLE_DEVELOPMENT	2	0.8418896	0.6876356
Upregulated Genes	GO_ODONTOGENESIS_OF_DENTIN_CONTAINING_TOOTH	2	0.8418453	0.69753087
Upregulated Genes	GO_REGULATION_OF_PROTEIN_KINASE_ACTIVITY	26	0.8418022	0.6861702
Upregulated Genes	GO_MITOTIC_RECOMBINATION	3	0.83929837	0.639413
Upregulated Genes	GO_NEGATIVE_REGULATION_OF_GROWTH	5	0.8389684	0.6273148
Upregulated Genes	GO_PHOSPHOLIPID_METABOLIC_PROCESS	5	0.8385458	0.6635294
Upregulated Genes	GO_MONOSACCHARIDE_METABOLIC_PROCESS	2	0.8383944	0.6788793
Upregulated Genes	GO_CELLULAR_RESPONSE_TO_STEROID_HORMONE_STIMULUS	7	0.83794427	0.61800486
Upregulated Genes	GO_UNSATURATED_FATTY_ACID_METABOLIC_PROCESS	4	0.8370991	0.65011287
Upregulated Genes	GO_MONONUCLEAR_CELL_MIGRATION	3	0.83534694	0.6579521
Upregulated Genes	GO_GROWTH_HORMONE_RECEPTOR_SIGNALING_PATHWAY_VIA_JAK_STAT	1	0.83487874	0.7360825
Upregulated Genes	GO_PROTEIN_MATURATION	4	0.834191	0.6773455
Upregulated Genes	GO_REGULATION_OF_PROTEIN_COMPLEX_STABILITY	1	0.833709	0.7292929
Upregulated Genes	GO_NEPHRON_TUBULE_FORMATION	1	0.8328328	0.73964494
Upregulated Genes	GO_CARDIAC_CHAMBER_MORPHOGENESIS	2	0.83238316	0.68546635
Upregulated Genes	GO_DOUBLE_STRAND_BREAK_REPAIR_VIA_SYNTHESIS_DEPENDENT_STRAND_ANNEALING	1	0.82975113	0.7490119
Upregulated Genes	GO_ACTIVATION_OF_JANUS_KINASE_ACTIVITY	1	0.828118	0.7560484
Upregulated Genes	GO_SEGMENT_SPECIFICATION	1	0.8272333	0.74432987
Upregulated Genes	GO_TAURINE_METABOLIC_PROCESS	1	0.8264256	0.7768595
Upregulated Genes	GO_RENAL_TUBULE_DEVELOPMENT	1	0.8261113	0.7495069
Upregulated Genes	GO_ORGANIC_HYDROXY_COMPOUND_METABOLIC_PROCESS	10	0.8252821	0.6674757
Upregulated Genes	GO_POSITIVE_REGULATION_OF_MULTICELLULAR_ORGANISM_GROWTH	1	0.825193	0.76254827
Upregulated Genes	GO_GLUCOSE_METABOLIC_PROCESS	2	0.8251155	0.71276593
Upregulated Genes	GO_CELLULAR_ALDEHYDE_METABOLIC_PROCESS	2	0.82481545	0.7237113
Upregulated Genes	GO_CELLULAR_RESPONSE_TO_GROWTH_HORMONE_STIMULUS	1	0.8244241	0.7556391
Upregulated Genes	GO_VENTRICULAR_SEPTUM_DEVELOPMENT	2	0.82387275	0.7027601
Upregulated Genes	GO_NUCLEOSIDE_PHOSPHATE_CATABOLIC_PROCESS	2	0.8228609	0.6974249
Upregulated Genes	GO_INSULIN_LIKE_GROWTH_FACTOR_RECEPTOR_SIGNALING_PATHWAY	1	0.82270455	0.74549097
Upregulated Genes	GO_REGULATION_OF_CYTOPLASMIC_MRNA_PROCESSING_BODY_ASSEMBLY	1	0.821247	0.7715431
Upregulated Genes	GO_SENSORY_PERCEPTION	5	0.8211931	0.69124424
Upregulated Genes	GO_POSITIVE_REGULATION_OF_INTRACELLULAR_SIGNAL_TRANSDUCTION	20	0.81997347	0.6946565
Upregulated Genes	GO_NEPHRON_EPITHELIUM_DEVELOPMENT	1	0.81988066	0.7447217
Upregulated Genes	GO_LOOP_OF_HENLE_DEVELOPMENT	1	0.8197506	0.7624751
Upregulated Genes	GO_NEPHRON_MORPHOGENESIS	1	0.81948113	0.76152307
Upregulated Genes	GO_PROXIMAL_DISTAL_PATTERN_FORMATION	1	0.81939995	0.74493927
Upregulated Genes	GO_RESPONSE_TO_GROWTH_HORMONE	1	0.81916016	0.77732795
Upregulated Genes	GO_APICAL_JUNCTION_ASSEMBLY	4	0.8186505	0.6912752
Upregulated Genes	GO_CELLULAR_TRANSITION_METAL_ION_HOMEOSTASIS	1	0.8169059	0.7745098
Upregulated Genes	GO_RESPONSE_TO_ALCOHOL	8	0.8163777	0.6317073
Upregulated Genes	GO_MYELOID_LEUKOCYTE_ACTIVATION	10	0.8146472	0.6481481
Upregulated Genes	GO_CARDIAC_SEPTUM_MORPHOGENESIS	2	0.81433713	0.71548116
Upregulated Genes	GO_REGULATION_OF_MULTICELLULAR_ORGANISM_GROWTH	1	0.8142401	0.7928287
Upregulated Genes	GO_MEMBRANE_ORGANIZATION	10	0.8127115	0.66584766
Upregulated Genes	GO_POSITIVE_REGULATION_OF_CYTOPLASMIC_MRNA_PROCESSING_BODY_ASSEMBLY	1	0.812385	0.74693877
Upregulated Genes	GO_MESODERM_DEVELOPMENT	1	0.8111344	0.795501
Upregulated Genes	GO_TAXIS	13	0.81057984	0.6917098
Upregulated Genes	GO_P_BODY_ASSEMBLY	1	0.8101969	0.78185326
Upregulated Genes	GO_AXIS_SPECIFICATION	3	0.80901897	0.70412844
Upregulated Genes	GO_STEROID_METABOLIC_PROCESS	6	0.80895805	0.68550366
Upregulated Genes	GO_REGULATION_OF_CELL_MORPHOGENESIS	5	0.8078048	0.6884058
Upregulated Genes	GO_RESPONSE_TO_ALKALOID	3	0.8075779	0.6588785
Upregulated Genes	GO_CELLULAR_IRON_ION_HOMEOSTASIS	1	0.80751675	0.7847358
Upregulated Genes	GO_NUCLEAR_TRANSCRIBED_MRNA_CATABOLIC_PROCESS	3	0.806811	0.7096774
Upregulated Genes	GO_CELL_GROWTH	6	0.80642605	0.6882217
Upregulated Genes	GO_PROTEIN_LOCALIZATION_TO_PLASMA_MEMBRANE	3	0.80597275	0.6926503
Upregulated Genes	GO_POSITIVE_REGULATION_OF_MRNA_METABOLIC_PROCESS	3	0.8057229	0.71710527
Upregulated Genes	GO_KERATINOCYTE_DIFFERENTIATION	6	0.8055273	0.71981776
Upregulated Genes	GO_PATTERN_SPECIFICATION_INVOLVED_IN_KIDNEY_DEVELOPMENT	1	0.805001	0.79358715
Upregulated Genes	GO_RESPONSE_TO_EPIDERMAL_GROWTH_FACTOR	3	0.8031155	0.7316017
Upregulated Genes	GO_CELLULAR_RESPONSE_TO_INORGANIC_SUBSTANCE	4	0.8026341	0.6962617
Upregulated Genes	GO_MAINTENANCE_OF_LOCATION_IN_CELL	2	0.80248564	0.7488987
Upregulated Genes	GO_NUCLEAR_TRANSCRIBED_MRNA_POLY_A_TAIL_SHORTENING	1	0.801789	0.78672034
Upregulated Genes	GO_REGULATION_OF_PROTEIN_CONTAINING_COMPLEX_DISASSEMBLY	2	0.8016795	0.73319757

Upregulated Genes	GO_REGULATION_OF_ACTIN_FILAMENT_ORGANIZATION	6	0.80073005	0.6882494
Upregulated Genes	GO_KIDNEY_EPITHELIUM_DEVELOPMENT	1	0.8006518	0.80042017
Upregulated Genes	GO_CELLULAR_RESPONSE_TO_PEPTIDE_HORMONE_STIMULUS	3	0.80051345	0.7136752
Upregulated Genes	GO_NEGATIVE_REGULATION_OF_DNA_TEMPLATED_TRANSCRIPTION_ELONGATION	1	0.79979056	0.8074534
Upregulated Genes	GO_REGULATION_OF_PROTEIN_LOCALIZATION_TO_MEMBRANE	3	0.7985076	0.72460496
Upregulated Genes	GO_NEURON_PROJECTION_REGENERATION	2	0.797886	0.73100615
Upregulated Genes	GO_REGULATION_OF_MONOOXYGENASE_ACTIVITY	2	0.7973173	0.75
Upregulated Genes	GO_POSITIVE_REGULATION_OF_DENDRITE_DEVELOPMENT	1	0.7969422	0.8114754
Upregulated Genes	GO_REGULATION_OF_CELL_MORPHOGENESIS_INVOLVED_IN_DIFFERENTIATION	5	0.79635996	0.7242991
Upregulated Genes	GO_SENSORY_SYSTEM_DEVELOPMENT	3	0.79417306	0.72602737
Upregulated Genes	GO_BEHAVIOR	9	0.79411656	0.6981132
Upregulated Genes	GO_PROTEIN_LOCALIZATION_TO_CELL_PERIPHERY	3	0.79372734	0.71011233
Upregulated Genes	GO_RESPONSE_TO_XENOBIOTIC_STIMULUS	5	0.79358137	0.70288247
Upregulated Genes	GO_NEGATIVE_REGULATION_OF_SIGNALING_RECEPTOR_ACTIVITY	1	0.7925396	0.8004115
Upregulated Genes	GO_REGULATION_OF_NITRIC_OXIDE_SYNTHASE_ACTIVITY	2	0.79222715	0.743083
Upregulated Genes	GO_TRANSCRIPTION_ELONGATION_FROM_RNA_POLYMERASE_II_PROMOTER	1	0.79140806	0.8028169
Upregulated Genes	GO_NEGATIVE_REGULATION_OF_RIBOSOME_BIOGENESIS	1	0.79071605	0.8168421
Upregulated Genes	GO_MULTICELLULAR_ORGANISMAL_RESPONSE_TO_STRESS	1	0.7902631	0.80038023
Upregulated Genes	GO_STRIATED_MUSCLE_ADAPTATION	1	0.79018563	0.790224
Upregulated Genes	GO_PROTEIN_SUMOYLATION	2	0.7901388	0.76391983
Upregulated Genes	GO_DENDRITIC_SPINE_DEVELOPMENT	1	0.78951985	0.8043912
Upregulated Genes	GO_POSITIVE_REGULATION_OF_EXTRINSIC_APOPTOTIC_SIGNALING_PATHWAY_VIA_DEATH_DC	1	0.7891377	0.79923517
Upregulated Genes	GO_RESPONSE_TO_ARSENIC_CONTAINING_SUBSTANCE	1	0.7889203	0.8212058
Upregulated Genes	GO_CELLULAR_RESPONSE_TO_ANTIBIOTIC	1	0.78891593	0.8143712
Upregulated Genes	GO_RESPONSE_TO_ANTIBIOTIC	1	0.7888374	0.8089431
Upregulated Genes	GO_POSTSYNAPTIC_SPECIALIZATION_ORGANIZATION	1	0.7885776	0.805726
Upregulated Genes	GO_FOCAL_ADHESION_ASSEMBLY	1	0.7883829	0.804829
Upregulated Genes	GO_NEGATIVE_REGULATION_OF_CELL_SIZE	1	0.78814137	0.81836736
Upregulated Genes	GO_CENTRAL_NERVOUS_SYSTEM_NEURON_AXONOGENESIS	1	0.7880666	0.81584156
Upregulated Genes	GO_MUSCLE_HYPERTROPHY_IN_RESPONSE_TO_STRESS	1	0.7880043	0.81584156
Upregulated Genes	GO_CHROMATIN_SILENCING_AT_TELOMERE	1	0.7875116	0.8121212
Upregulated Genes	GO_PRESYNAPTIC_MEMBRANE_ORGANIZATION	1	0.78741336	0.8109756
Upregulated Genes	GO_NEGATIVE_REGULATION_OF_CELL_SUBSTRATE_JUNCTION_ORGANIZATION	1	0.78731614	0.827853
Upregulated Genes	GO_STARTLE_RESPONSE	1	0.7871523	0.8053279
Upregulated Genes	GO_STRIATED_MUSCLE_CELL_APOPTOTIC_PROCESS	1	0.7861664	0.8244576
Upregulated Genes	GO_MUSCLE_ADAPTATION	1	0.78612125	0.822314
Upregulated Genes	GO_POSITIVE_REGULATION_OF_EXCITATORY_POSTSYNAPTIC_POTENTIAL	1	0.7858165	0.82222223
Upregulated Genes	GO_FOREBRAIN_MORPHOGENESIS	1	0.78556097	0.8201581
Upregulated Genes	GO_POSITIVE_REGULATION_OF_STRIATED_MUSCLE_CELL_APOPTOTIC_PROCESS	1	0.7854734	0.80701756
Upregulated Genes	GO_EXCITATORY_SYNAPSE_ASSEMBLY	1	0.7850655	0.82421875
Upregulated Genes	GO_CHEMICAL_SYNAPTIC_TRANSMISSION_POSTSYNAPTIC	1	0.784777	0.8286853
Upregulated Genes	GO_CHONDROCYTE_DIFFERENTIATION	3	0.78456086	0.7113164
Upregulated Genes	GO_MALE_MATING_BEHAVIOR	1	0.78450704	0.80658436
Upregulated Genes	GO_CELLULAR_RESPONSE_TO_ETHANOL	1	0.7843039	0.8126273
Upregulated Genes	GO_DNA_MODIFICATION	5	0.7841946	0.6921296
Upregulated Genes	GO_NEGATIVE_REGULATION_OF_DENDRITE_MORPHOGENESIS	1	0.7840776	0.8102767
Upregulated Genes	GO_NEGATIVE_REGULATION_OF_DENDRITE_DEVELOPMENT	1	0.78404224	0.825
Upregulated Genes	GO_NEGATIVE_REGULATION_OF_NEURON_PROJECTION_REGENERATION	1	0.78399384	0.8060606
Upregulated Genes	GO_LYMPHOCYTE_APOPTOTIC_PROCESS	1	0.7837225	0.81237525
Upregulated Genes	GO_POSTSYNAPTIC_SPECIALIZATION_ASSEMBLY	1	0.7835973	0.8408644
Upregulated Genes	GO_CELLULAR_RESPONSE_TO_ELECTRICAL_STIMULUS	1	0.78356683	0.8104449
Upregulated Genes	GO_HISTONE_H3_K27_TRIMETHYLATION	1	0.7835533	0.8352941
Upregulated Genes	GO_PRESYNAPSE_ORGANIZATION	1	0.783509	0.8060837
Upregulated Genes	GO_DENTATE_GYRUS_DEVELOPMENT	1	0.7834642	0.81
Upregulated Genes	GO_PHOSPHOLIPID_DEPHOSPHORYLATION	1	0.78333724	0.819802
Upregulated Genes	GO_REGULATION_OF_NEURON_PROJECTION_REGENERATION	1	0.7829988	0.7920792
Upregulated Genes	GO_REGULATION_OF_SYNAPTIC_VESICLE_CYCLE	1	0.7829108	0.8241107
Upregulated Genes	GO_REGULATION_OF_DENDRITIC_SPINE_DEVELOPMENT	1	0.782838	0.8283898
Upregulated Genes	GO_SYNAPSE_MATURATION	1	0.7827016	0.81837606
Upregulated Genes	GO_MUSCLE_CELL_APOPTOTIC_PROCESS	1	0.78261834	0.83044314
Upregulated Genes	GO_RESPONSE_TO_ELECTRICAL_STIMULUS	1	0.7824095	0.8214286
Upregulated Genes	GO_REGULATION_OF_WOUND_HEALING_SPREADING_OF_EPIDERMAL_CELLS	1	0.78237957	0.8216433
Upregulated Genes	GO_CELLULAR_RESPONSE_TO_INSULIN_LIKE_GROWTH_FACTOR_STIMULUS	1	0.7823715	0.83524907
Upregulated Genes	GO_RESPONSE_TO_ATP	1	0.782272	0.8368932
Upregulated Genes	GO_REGULATION_OF_SYNAPTIC_VESICLE_CLUSTERING	1	0.78216356	0.8181818
Upregulated Genes	GO_MRNA_TRANSPORT	2	0.78184986	0.7617021
Upregulated Genes	GO_MATING_BEHAVIOR	1	0.781804	0.808
Upregulated Genes	GO_NEGATIVE_REGULATION_OF_POTASSIUM_ION_TRANSMEMBRANE_TRANSPORT	1	0.78173745	0.8297456
Upregulated Genes	GO_REGULATION_OF_TRANSCRIPTION_ELONGATION_FROM_RNA_POLYMERASE_II_PROMOTER	1	0.7816923	0.8172888
Upregulated Genes	GO_REGULATION_OF_POSTSYNAPTIC_MEMBRANE_POTENTIAL	1	0.78167397	0.8377823

Upregulated Genes	GO_ORGANOPHOSPHATE_METABOLIC_PROCESS	16	0.78165746	0.7315271
Upregulated Genes	GO_NEGATIVE_REGULATION_OF_MUSCLE_CELL_DIFFERENTIATION	1	0.78160334	0.8215768
Upregulated Genes	GO_RHYTHMIC_BEHAVIOR	1	0.7815567	0.8346154
Upregulated Genes	GO_VESICLE_MEDIATED_TRANSPORT_IN_SYNAPSE	1	0.78143287	0.8265107
Upregulated Genes	GO_WOUND_HEALING_SPREADING_OF_EPIDERMAL_CELLS	1	0.7813167	0.838
Upregulated Genes	GO_EPIBOLY	1	0.7812404	0.82857144
Upregulated Genes	GO_G1_TO_G0_TRANSITION	1	0.78120136	0.8
Upregulated Genes	GO_REGULATION_OF_CELLULAR_LOCALIZATION	19	0.781138	0.75
Upregulated Genes	GO_CANONICAL_WNT_SIGNALING_PATHWAY	5	0.78105956	0.7258427
Upregulated Genes	GO_MORPHOGENESIS_OF_AN_EPITHELIAL_SHEET	1	0.7809835	0.8450704
Upregulated Genes	GO_INOSITOL_PHOSPHATE_CATABOLIC_PROCESS	1	0.78090197	0.8215768
Upregulated Genes	GO_NEGATIVE_REGULATION_OF_DENDRITIC_SPINE_DEVELOPMENT	1	0.7807128	0.8300395
Upregulated Genes	GO_REGULATION_OF_SUPRAMOLECULAR_FIBER_ORGANIZATION	8	0.7806081	0.7051887
Upregulated Genes	GO_NEURON_NEURON_SYNAPTIC_TRANSMISSION	1	0.7803871	0.8141414
Upregulated Genes	GO_POSITIVE_REGULATION_OF_NERVOUS_SYSTEM_PROCESS	1	0.7803771	0.812749
Upregulated Genes	GO_NEGATIVE_REGULATION_OF_NERVOUS_SYSTEM_DEVELOPMENT	6	0.78034204	0.7245119
Upregulated Genes	GO_NEGATIVE_REGULATION_OF_CELL_MATRIX_ADHESION	1	0.780313	0.83829784
Upregulated Genes	GO_PARENTAL_BEHAVIOR	1	0.78028435	0.83044314
Upregulated Genes	GO_DEPHOSPHORYLATION	8	0.7799733	0.7237443
Upregulated Genes	GO_DENDRITIC_SPINE_MORPHOGENESIS	1	0.7796568	0.82509506
Upregulated Genes	GO_DNA_TEMPLATED_TRANSCRIPTION_ELONGATION	1	0.7796156	0.83716077
Upregulated Genes	GO_REGULATION_OF_POTASSIUM_ION_TRANSPORT	1	0.7795787	0.828125
Upregulated Genes	GO_REGULATION_OF_DNA_TEMPLATED_TRANSCRIPTION_ELONGATION	1	0.77909887	0.84136546
Upregulated Genes	GO_POSITIVE_REGULATION_OF_CELL_CYCLE_G1_S_PHASE_TRANSITION	1	0.77898014	0.848
Upregulated Genes	GO_REGULATION_OF_DENDRITE_MORPHOGENESIS	1	0.77889645	0.8333333
Upregulated Genes	GO_NEGATIVE_REGULATION_OF_TRANSPORTER_ACTIVITY	1	0.7787438	0.8480493
Upregulated Genes	GO_NEGATIVE_REGULATION_OF_VASCULAR_ASSOCIATED_SMOOTH_MUSCLE_CELL_PROLIFERA	1	0.77857614	0.8484849
Upregulated Genes	GO_REGULATION_OF_NEURON_PROJECTION_DEVELOPMENT	6	0.77854437	0.69902915
Upregulated Genes	GO_LOCOMOTOR_RHYTHM	1	0.7783161	0.82258064
Upregulated Genes	GO_NEGATIVE_REGULATION_OF_POTASSIUM_ION_TRANSPORT	1	0.7782836	0.8390342
Upregulated Genes	GO_NEGATIVE_REGULATION_OF_EPITHELIAL_TO_MESENCHYMAL_TRANSITION	1	0.7781694	0.8403042
Upregulated Genes	GO_REGULATION_OF_VASCULAR_ASSOCIATED_SMOOTH_MUSCLE_CELL_PROLIFERATION	1	0.7780217	0.8246628
Upregulated Genes	GO_NEUROMUSCULAR_PROCESS	1	0.77793753	0.84033614
Upregulated Genes	GO_HEMATOPOIETIC_PROGENITOR_CELL_DIFFERENTIATION	6	0.7778147	0.7223529
Upregulated Genes	GO_PHOSPHORYLATED_CARBOHYDRATE_DEPHOSPHORYLATION	1	0.77775604	0.8308977
Upregulated Genes	GO_NEGATIVE_REGULATION_OF_CELL_JUNCTION_ASSEMBLY	1	0.7776892	0.8125
Upregulated Genes	GO_B_CELL_APOPTOTIC_PROCESS	1	0.77768147	0.829703
Upregulated Genes	GO_REGULATION_OF_RIBOSOME_BIOGENESIS	1	0.7775762	0.83567137
Upregulated Genes	GO_CAMP_METABOLIC_PROCESS	1	0.7775535	0.8411215
Upregulated Genes	GO_DNA_STRAND_RENATURATION	1	0.7775369	0.8302658
Upregulated Genes	GO_REGULATION_OF_LYMPHOCYTE_APOPTOTIC_PROCESS	1	0.7771675	0.8333333
Upregulated Genes	GO_RESPONSE_TO_LEPTIN	1	0.7771467	0.8181818
Upregulated Genes	GO_NEGATIVE_REGULATION_OF_DENDRITIC_SPINE_MORPHOGENESIS	1	0.77698183	0.83168316
Upregulated Genes	GO_REGULATION_OF_TRAIL_ACTIVATED_APOPTOTIC_SIGNALING_PATHWAY	1	0.7766748	0.8426966
Upregulated Genes	GO_REGULATION_OF_LEUKOCYTE_MEDIATED_IMMUNITY	7	0.77655447	0.74004686
Upregulated Genes	GO_SYNAPSE_ASSEMBLY	1	0.77626824	0.83011585
Upregulated Genes	GO_MODULATION_OF_EXCITATORY_POSTSYNAPTIC_POTENTIAL	1	0.7762239	0.8218391
Upregulated Genes	GO_NEURON_PROJECTION_GUIDANCE	6	0.776194	0.72897196
Upregulated Genes	GO_CELL_CYCLE_DNA_REPLICATION_INITIATION	2	0.7759217	0.76890755
Upregulated Genes	GO_REGULATION_OF_POTASSIUM_ION_TRANSMEMBRANE_TRANSPORTER_ACTIVITY	1	0.7758395	0.8221344
Upregulated Genes	GO_POLYOL_CATABOLIC_PROCESS	1	0.7754237	0.8258197
Upregulated Genes	GO_TRAIL_ACTIVATED_APOPTOTIC_SIGNALING_PATHWAY	1	0.77535963	0.8479532
Upregulated Genes	GO_RESPONSE_TO_NERVE_GROWTH_FACTOR	1	0.77514035	0.8666667
Upregulated Genes	GO_POSITIVE_REGULATION_OF_EXTRINSIC_APOPTOTIC_SIGNALING_PATHWAY	1	0.7750254	0.84468085
Upregulated Genes	GO_NEGATIVE_REGULATION_OF_TRANSMEMBRANE_TRANSPORT	1	0.7749028	0.82730925
Upregulated Genes	GO_REPRODUCTIVE_BEHAVIOR	1	0.77473027	0.83914727
Upregulated Genes	GO_RELAXATION_OF_CARDIAC_MUSCLE	1	0.77467036	0.8327138
Upregulated Genes	GO_BRAIN_MORPHOGENESIS	1	0.7746667	0.8449304
Upregulated Genes	GO_REGULATION_OF_CELL_SUBSTRATE_JUNCTION_ORGANIZATION	1	0.77458066	0.84313726
Upregulated Genes	GO_PHOSPHATIDYLINOSITOL_DEPHOSPHORYLATION	1	0.77441245	0.83265305
Upregulated Genes	GO_POSITIVE_REGULATION_OF_MUSCLE_CELL_APOPTOTIC_PROCESS	1	0.7742868	0.829703
Upregulated Genes	GO_NEGATIVE_REGULATION_OF_STRIATED_MUSCLE_CELL_DIFFERENTIATION	1	0.77427834	0.8372093
Upregulated Genes	GO_RESPONSE_TO_ZINC_ION	1	0.7740872	0.8212058
Upregulated Genes	GO_PLATELET_DERIVED_GROWTH_FACTOR_RECEPTOR_SIGNALING_PATHWAY	1	0.77406037	0.8634538
Upregulated Genes	GO_REGULATION_OF_LEUKOCYTE_APOPTOTIC_PROCESS	1	0.7736668	0.82718444
Upregulated Genes	GO_NEGATIVE_REGULATION_OF_ION_TRANSMEMBRANE_TRANSPORT	1	0.7734705	0.84646463
Upregulated Genes	GO_PREPULSE_INHIBITION	1	0.77334636	0.83435583
Upregulated Genes	GO_MEMBRANE_LIPID_METABOLIC_PROCESS	2	0.773038	0.7724426
Upregulated Genes	GO_CELLULAR_RESPONSE_TO_LEPTIN_STIMULUS	1	0.7727189	0.82806325
Upregulated Genes	GO_REGULATION_OF_B_CELL_APOPTOTIC_PROCESS	1	0.7726066	0.8388998

Upregulated Genes	GO_CHROMATIN_MEDIATED_MAINTENANCE_OF_TRANSCRIPTION	1	0.772464	0.8607595
Upregulated Genes	GO_REGULATION_OF_CALCIUM_ION_TRANSMEMBRANE_TRANSPORTER_ACTIVITY	1	0.7722297	0.831643
Upregulated Genes	GO_INOSITOL_PHOSPHATE_METABOLIC_PROCESS	1	0.7722104	0.8463074
Upregulated Genes	GO_NEGATIVE_REGULATION_OF_MORPHOGENESIS_OF_AN_EPITHELIUM	1	0.7721718	0.85510206
Upregulated Genes	GO_SYNAPTIC_VESICLE_CLUSTERING	1	0.7721183	0.8515464
Upregulated Genes	GO_NEURON_PROJECTION_ORGANIZATION	1	0.7720622	0.80932206
Upregulated Genes	GO_FATTY_ACID_DERIVATIVE_METABOLIC_PROCESS	4	0.77184314	0.76521736
Upregulated Genes	GO_REGULATION_OF_CATION_CHANNEL_ACTIVITY	1	0.7716193	0.84305835
Upregulated Genes	GO_CELLULAR_RESPONSE_TO_ABIOTIC_STIMULUS	11	0.77143437	0.7192118
Upregulated Genes	GO_NEGATIVE_REGULATION_OF_EXCITATORY_POSTSYNAPTIC_POTENTIAL	1	0.7711323	0.8483034
Upregulated Genes	GO_NEGATIVE_REGULATION_OF_PEPTIDYL_SERINE_PHOSPHORYLATION	1	0.77109826	0.85539716
Upregulated Genes	GO_REGULATION_OF_SYNAPTIC_TRANSMISSION_GABAERGIC	1	0.7710521	0.85110664
Upregulated Genes	GO_INTRASPECIES_INTERACTION_BETWEEN_ORGANISMS	1	0.77088445	0.83697814
Upregulated Genes	GO_MEMBRANE_LIPID_BIOSYNTHETIC_PROCESS	2	0.7704988	0.7564655
Upregulated Genes	GO_NEGATIVE_REGULATION_OF_SMOOTH_MUSCLE_CELL_PROLIFERATION	1	0.77044743	0.8245243
Upregulated Genes	GO_SYNAPTIC_TRANSMISSION_GABAERGIC	1	0.7702993	0.851927
Upregulated Genes	GO_ALCOHOL_CATABOLIC_PROCESS	1	0.7702614	0.8426295
Upregulated Genes	GO_CELLULAR_RESPONSE_TO_EPINEPHRINE_STIMULUS	1	0.77019125	0.8579767
Upregulated Genes	GO_LIPID_MODIFICATION	2	0.7693526	0.7463312
Upregulated Genes	GO_REGULATION_OF_CALCIUM_ION_TRANSMEMBRANE_TRANSPORT	1	0.76859045	0.8479532
Upregulated Genes	GO_REGULATION_OF_RELAXATION_OF_CARDIAC_MUSCLE	1	0.76848894	0.85215604
Upregulated Genes	GO_POSITIVE_REGULATION_OF_INTERLEUKIN_8_PRODUCTION	3	0.7678184	0.7189696
Upregulated Genes	GO_VASCULATURE_DEVELOPMENT	9	0.7677165	0.72921616
Upregulated Genes	GO_CYCLIC_NUCLEOTIDE_CATABOLIC_PROCESS	1	0.7673713	0.8515464
Upregulated Genes	GO_DEMETHYLATION	1	0.7673615	0.85862786
Upregulated Genes	GO_CELLULAR_RESPONSE_TO_DRUG	1	0.7671645	0.8466387
Upregulated Genes	GO_TETRAPYRROLE_METABOLIC_PROCESS	1	0.76682496	0.83501005
Upregulated Genes	GO_POSITIVE_REGULATION_OF_CELL_SUBSTRATE_ADHESION	1	0.76661646	0.832998
Upregulated Genes	GO_GLYCOSPHINGOLIPID_METABOLIC_PROCESS	2	0.76657325	0.7933884
Upregulated Genes	GO_REGULATION_OF_DENDRITIC_SPINE_MORPHOGENESIS	1	0.76582444	0.85146445
Upregulated Genes	GO_LIPOSACCHARIDE_METABOLIC_PROCESS	2	0.765819	0.7873684
Upregulated Genes	GO_RIBONUCLEOTIDE_CATABOLIC_PROCESS	1	0.7657882	0.8346154
Upregulated Genes	GO_REGULATION_OF_RELAXATION_OF_MUSCLE	1	0.7657852	0.8434959
Upregulated Genes	GO_REGULATION_OF_VOLTAGE_GATED_CALCIUM_CHANNEL_ACTIVITY	1	0.76553965	0.8643123
Upregulated Genes	GO_RESPONSE_TO_EPINEPHRINE	1	0.7651651	0.8404908
Upregulated Genes	GO_REGULATION_OF_KERATINOCYTE_MIGRATION	1	0.7650712	0.8497942
Upregulated Genes	GO_VIRAL_GENE_EXPRESSION	2	0.76496375	0.78541666
Upregulated Genes	GO_CERAMIDE_BIOSYNTHETIC_PROCESS	2	0.76487345	0.793722
Upregulated Genes	GO_CYCLIC_NUCLEOTIDE_METABOLIC_PROCESS	1	0.7647136	0.85328186
Upregulated Genes	GO_NEGATIVE_REGULATION_OF_EPITHELIAL_CELL_MIGRATION	3	0.7643965	0.7887029
Upregulated Genes	GO_INTERLEUKIN_6_PRODUCTION	5	0.7642973	0.72771084
Upregulated Genes	GO_CERAMIDE_METABOLIC_PROCESS	2	0.7641564	0.7777778
Upregulated Genes	GO_PROGESTERONE_RECEPTOR_SIGNALING_PATHWAY	1	0.76402175	0.85009485
Upregulated Genes	GO_MEMBRANE_BIOGENESIS	1	0.7637084	0.8649789
Upregulated Genes	GO_POSTSYNAPSE_ASSEMBLY	1	0.7625753	0.8379447
Upregulated Genes	GO_ENDOTHELIAL_CELL_MATRIX_ADHESION	1	0.76240337	0.85119045
Upregulated Genes	GO_T_CELL_MEDIATED_IMMUNITY	3	0.7623386	0.7664399
Upregulated Genes	GO_POSITIVE_REGULATION_OF_EPITHELIAL_CELL_MIGRATION	3	0.7614675	0.8066667
Upregulated Genes	GO_REGULATION_OF_HIGH_VOLTAGE_GATED_CALCIUM_CHANNEL_ACTIVITY	1	0.7611056	0.8359375
Upregulated Genes	GO_CENTRAL_NERVOUS_SYSTEM_MYELIN_MAINTENANCE	2	0.7608882	0.7777778
Upregulated Genes	GO_NEPHRON_DEVELOPMENT	2	0.76079434	0.7995595
Upregulated Genes	GO_POSITIVE_REGULATION_OF_INTERLEUKIN_6_PRODUCTION	5	0.76033133	0.786036
Upregulated Genes	GO_MULTI_ORGANISM_PROCESS	25	0.76031375	0.7642487
Upregulated Genes	GO_PROTEIN_CONTAINING_COMPLEX_LOCALIZATION	2	0.76023036	0.81666666
Upregulated Genes	GO_NEGATIVE_REGULATION_OF_NERVOUS_SYSTEM_PROCESS	2	0.7601255	0.7860262
Upregulated Genes	GO_NEUTROPHIL_HOMEOSTASIS	3	0.7601228	0.776699
Upregulated Genes	GO_REGULATION_OF_VASCULOGENESIS	1	0.76011014	0.8459959
Upregulated Genes	GO_CAMP_CATABOLIC_PROCESS	1	0.7595411	0.8562368
Upregulated Genes	GO_COBALAMIN_METABOLIC_PROCESS	1	0.75915676	0.8489796
Upregulated Genes	GO_REGULATION_OF_CELLULAR_RESPONSE_TO_HEAT	2	0.7590791	0.81696427
Upregulated Genes	GO_REGULATION_OF_MONONUCLEAR_CELL_MIGRATION	2	0.75894177	0.7907489
Upregulated Genes	GO_SPHINGOLIPID_METABOLIC_PROCESS	2	0.758818	0.7782258
Upregulated Genes	GO_HOMOLOGOUS_CHROMOSOME_PAIRING_AT_MEIOSIS	3	0.75881636	0.7792793
Upregulated Genes	GO_REGULATION_OF_MICROGLIAL_CELL_ACTIVATION	2	0.75865763	0.7831579
Upregulated Genes	GO_POSITIVE_REGULATION_OF_INTERFERON_GAMMA_PRODUCTION	1	0.75860053	0.8594705
Upregulated Genes	GO_REGULATION_OF_DNA_BINDING_TRANSCRIPTION_FACTOR_ACTIVITY	7	0.75851214	0.75
Upregulated Genes	GO_NEGATIVE_REGULATION_OF_MYELINATION	2	0.75799674	0.7854251
Upregulated Genes	GO_POSITIVE_REGULATION_OF_CELL_MATRIX_ADHESION	1	0.7576016	0.87473905
Upregulated Genes	GO_REGULATION_OF_NEUROINFLAMMATORY_RESPONSE	2	0.7571737	0.79707116
Upregulated Genes	GO_BLOOD_VESSEL_ENDOTHELIAL_CELL_MIGRATION	2	0.75678223	0.77904326

Upregulated Genes	GO_NEGATIVE_REGULATION_OF_RELAXATION_OF_MUSCLE	1	0.75667614	0.8505976
Upregulated Genes	GO_OLIGODENDROCYTE_DEVELOPMENT	2	0.75640345	0.8004158
Upregulated Genes	GO_PATTERN_RECOGNITION_RECEPTOR_SIGNALING_PATHWAY	7	0.75610316	0.7799043
Upregulated Genes	GO_NUCLEOTIDE_EXCISION_REPAIR_PREINCISION_COMPLEX_ASSEMBLY	1	0.7549816	0.8354167
Upregulated Genes	GO_POSITIVE_REGULATION_OF_VASCULOGENESIS	1	0.75437135	0.8553459
Upregulated Genes	GO_RIBOSE_PHOSPHATE_METABOLIC_PROCESS	5	0.75426507	0.7470726
Upregulated Genes	GO_POSITIVE_REGULATION_OF_NEUROINFLAMMATORY_RESPONSE	2	0.7542585	0.8181818
Upregulated Genes	GO_MITOTIC_DNA_REPLICATION	2	0.75389564	0.8012685
Upregulated Genes	GO_RELAXATION_OF_MUSCLE	1	0.7538951	0.876
Upregulated Genes	GO_LEUKOCYTE_HOMEOSTASIS	3	0.7527561	0.8051392
Upregulated Genes	GO_HISTONE_DEACETYLATION	1	0.7524234	0.8428571
Upregulated Genes	GO_MONOCARBOXYLIC_ACID_METABOLIC_PROCESS	14	0.7522367	0.73696685
Upregulated Genes	GO_CELLULAR_RESPONSE_TO_HORMONE_STIMULUS	15	0.75203156	0.7581522
Upregulated Genes	GO_POSITIVE_REGULATION_OF_AUTOPHAGY	3	0.7515023	0.7995444
Upregulated Genes	GO_NUCLEOTIDE_EXCISION_REPAIR_PREINCISION_COMPLEX_STABILIZATION	1	0.75002307	0.8496094
Upregulated Genes	GO_POSITIVE_REGULATION_OF_ENDOTHELIAL_CELL_MATRIX_ADHESION_VIA_FIBRONECTIN	1	0.74877733	0.8694779
Upregulated Genes	GO_RESPONSE_TO_ESTRADIOL	6	0.7487202	0.76513314
Upregulated Genes	GO_AXON_ENSHEATHMENT_IN_CENTRAL_NERVOUS_SYSTEM	2	0.74868315	0.8137045
Upregulated Genes	GO_REGULATION_OF_ACTIN_FILAMENT_BASED_PROCESS	11	0.7477467	0.7577197
Upregulated Genes	GO_AXONAL_TRANSPORT	1	0.7475501	0.8780992
Upregulated Genes	GO_ENDOTHELIAL_CELL_MATRIX_ADHESION_VIA_FIBRONECTIN	1	0.74737895	0.8707071
Upregulated Genes	GO_NONRIBOSOMAL_PEPTIDE_BIOSYNTHETIC_PROCESS	1	0.7472279	0.8734694
Upregulated Genes	GO_RESPONSE_TO_FIBROBLAST_GROWTH_FACTOR	2	0.7460214	0.8258197
Upregulated Genes	GO_SPHINGOLIPID_BIOSYNTHETIC_PROCESS	2	0.7452846	0.81681037
Upregulated Genes	GO_REGULATION_OF_MYELINATION	2	0.7452642	0.7991453
Upregulated Genes	GO_REGULATION_OF_ENDOTHELIAL_CELL_MIGRATION	3	0.7450706	0.80865604
Upregulated Genes	GO_POSITIVE_REGULATION_OF_T_CELL_PROLIFERATION	3	0.7448307	0.7829787
Upregulated Genes	GO_MYELIN_MAINTENANCE	2	0.7446458	0.8103448
Upregulated Genes	GO_RESPONSE_TO_INTERLEUKIN_4	2	0.7444162	0.8040541
Upregulated Genes	GO_DEVELOPMENTAL_GROWTH_INVOLVED_IN_MORPHOGENESIS	5	0.74383456	0.7627119
Upregulated Genes	GO_CELL_SURFACE_RECEPTOR_SIGNALING_PATHWAY_INVOLVED_IN_CELL_CELL_SIGNALING	12	0.7437013	0.7915633
Upregulated Genes	GO_AXO_DENDRITIC_TRANSPORT	1	0.7432013	0.8974359
Upregulated Genes	GO_REGULATION_OF_VESICLE_TRANSPORT_ALONG_MICROTUBULE	1	0.74117017	0.88095236
Upregulated Genes	GO_EPIDERMAL_CELL_DIFFERENTIATION	8	0.74032086	0.7505995
Upregulated Genes	GO_ANTEROGRADE_AXONAL_TRANSPORT	1	0.73992103	0.8839779
Upregulated Genes	GO_REGULATION_OF_SYNAPTIC_VESICLE_TRANSPORT	1	0.7381201	0.88737863
Upregulated Genes	GO_REGULATION_OF_TRANSLATIONAL_TERMINATION	1	0.73739153	0.9001996
Upregulated Genes	GO_REGULATION_OF_GLIAL_CELL_PROLIFERATION	2	0.7372966	0.8076063
Upregulated Genes	GO_SENSORY_ORGAN_DEVELOPMENT	4	0.73687303	0.8072289
Upregulated Genes	GO_SYNAPTIC_VESICLE_TRANSPORT	1	0.73633105	0.89161557
Upregulated Genes	GO_NEGATIVE_REGULATION_OF_DEVELOPMENTAL_PROCESS	18	0.7356041	0.78933334
Upregulated Genes	GO_REGULATION_OF_MICROTUBULE_BASED_MOVEMENT	1	0.7352492	0.8957529
Upregulated Genes	GO_POSITIVE_REGULATION_OF_PROTEIN_CONTAINING_COMPLEX_DISASSEMBLY	1	0.7343277	0.9163347
Upregulated Genes	GO_PEPTIDYL_LYSINE_DIMETHYLATION	1	0.73417485	0.9108911
Upregulated Genes	GO_SYNAPTIC_VESICLE_CYTOSKELETAL_TRANSPORT	1	0.7335593	0.904943
Upregulated Genes	GO_REGULATION_OF_ORGANELLE_TRANSPORT_ALONG_MICROTUBULE	1	0.7323719	0.8918919
Upregulated Genes	GO_CELLULAR_RESPONSE_TO_CORTICOSTEROID_STIMULUS	4	0.7306525	0.7986577
Upregulated Genes	GO_POSITIVE_REGULATION_OF_TRANSLATIONAL_ELONGATION	1	0.7297402	0.8929336
Upregulated Genes	GO_TRANSLATIONAL_TERMINATION	1	0.7289551	0.9140969
Upregulated Genes	GO_POSITIVE_REGULATION_OF_GLIAL_CELL_PROLIFERATION	2	0.7278718	0.8076923
Upregulated Genes	GO_PURINE_NUCLEOBASE_METABOLIC_PROCESS	3	0.726008	0.81333333
Upregulated Genes	GO_APPENDAGE_MORPHOGENESIS	1	0.72593164	0.92759293
Upregulated Genes	GO_RESPONSE_TO_MOLECULE_OF_BACTERIAL_ORIGIN	9	0.7254435	0.7613365
Upregulated Genes	GO_PEPTIDYL_LYSINE_ACETYLATION	1	0.7246139	0.9219331
Upregulated Genes	GO_PEPTIDYL_LYSINE_TRIMETHYLATION	1	0.72434574	0.9066148
Upregulated Genes	GO_HISTONE_H3_K9_DIMETHYLATION	1	0.72430974	0.913371
Upregulated Genes	GO_DNA_METHYLATION	4	0.723774	0.8187919
Upregulated Genes	GO_NEGATIVE_REGULATION_OF_CIRCADIAN_RHYTHM	1	0.7230679	0.9271255
Upregulated Genes	GO_N_TERMINAL_PROTEIN_AMINO_ACID_MODIFICATION	1	0.72283536	0.92016804
Upregulated Genes	GO_CENTROMERIC_SISTER_CHROMATID_COHESION	1	0.7219127	0.92248064
Upregulated Genes	GO_ESTABLISHMENT_OF_SISTER_CHROMATID_COHESION	1	0.7213066	0.9068441
Upregulated Genes	GO_CELLULAR_RESPONSE_TO_LIPID	15	0.7209667	0.7777778
Upregulated Genes	GO_EMBRYONIC_APPENDAGE_MORPHOGENESIS	1	0.7198535	0.924
Upregulated Genes	GO_REGULATION_OF_SMOOTHENED_SIGNALING_PATHWAY	2	0.71983564	0.81737196
Upregulated Genes	GO_HISTONE_H3_K9_TRIMETHYLATION	1	0.7198326	0.8996139
Upregulated Genes	GO_NUCLEOBASE_CONTAINING_SMALL_MOLECULE_METABOLIC_PROCESS	12	0.71931523	0.75
Upregulated Genes	GO_APPENDAGE_DEVELOPMENT	1	0.71893173	0.92110455
Upregulated Genes	GO_CELL_CELL_SIGNALING_BY_WNT	12	0.718478	0.77862597
Upregulated Genes	GO_REGULATION_OF_PROTEIN_CONTAINING_COMPLEX_ASSEMBLY	17	0.71699166	0.7979798
Upregulated Genes	GO_POSITIVE_REGULATION_OF_LEUKOCYTE_MEDIATED_IMMUNITY	5	0.7167586	0.79820627

Upregulated Genes	GO_GLAND_MORPHOGENESIS	3	0.7166594	0.8088889
Upregulated Genes	GO_REGULATION_OF_CILIUM_ASSEMBLY	2	0.7141962	0.835443
Upregulated Genes	GO_HISTONE_H4_ACETYLATION	1	0.71316475	0.9241803
Upregulated Genes	GO_METANEPHROS_MORPHOGENESIS	1	0.71272916	0.922179
Upregulated Genes	GO_N_TERMINAL_PROTEIN_AMINO_ACID_ACETYLATION	1	0.71271074	0.9153226
Upregulated Genes	GO_PLASMA_MEMBRANE_TUBULATION	1	0.710865	0.9214876
Upregulated Genes	GO_RESPONSE_TO_KETONE	9	0.70881665	0.8045977
Upregulated Genes	GO_REGULATION_OF_ENDOCYTIC_RECYCLING	1	0.7063211	0.9500998
Upregulated Genes	GO_NEGATIVE_REGULATION_OF_FAT_CELL_DIFFERENTIATION	2	0.7055924	0.86315787
Upregulated Genes	GO_POSITIVE_REGULATION_OF_MYOTUBE_DIFFERENTIATION	1	0.7043573	0.9430894
Upregulated Genes	GO_REGULATION_OF_MYOBLAST_FUSION	1	0.70405996	0.936803
Upregulated Genes	GO_POSITIVE_REGULATION_OF_ENDOCYTIC_RECYCLING	1	0.70399845	0.93360996
Upregulated Genes	GO_REGULATION_OF_MYOTUBE_DIFFERENTIATION	1	0.7033956	0.9592668
Upregulated Genes	GO_RESPONSE_TO_PROGESTERONE	4	0.70053625	0.83076924
Upregulated Genes	GO_NCRNA_TRANSCRIPTION	1	0.6996779	0.9564356
Upregulated Genes	GO_PROTEIN_LOCALIZATION_TO_NUCLEAR_ENVELOPE	1	0.6995953	0.9634146
Upregulated Genes	GO_DENDRITE_MORPHOGENESIS	2	0.699186	0.8660714
Upregulated Genes	GO_REGULATION_OF_STEM_CELL_DIFFERENTIATION	2	0.6985579	0.85281384
Upregulated Genes	GO_UV_PROTECTION	1	0.69727534	0.94768614
Upregulated Genes	GO_POSITIVE_REGULATION_OF_MUSCLE_CELL_DIFFERENTIATION	1	0.69680285	0.9541985
Upregulated Genes	GO_SNRNA_TRANSCRIPTION	1	0.6963728	0.9533074
Upregulated Genes	GO_ENDOSOMAL_TRANSPORT	1	0.6962086	0.9442149
Upregulated Genes	GO_REGULATION_OF_SYNCYTIUM_FORMATION_BY_PLASMA_MEMBRANE_FUSION	1	0.69599897	0.93465346
Upregulated Genes	GO_NUCLEAR_TRANSCRIBED_MRNA_CATABOLIC_PROCESS_DEADENYLATION_DEPENDENT_DEC	2	0.6953008	0.871308
Upregulated Genes	GO_FAT_CELL_DIFFERENTIATION	2	0.69493437	0.8703297
Upregulated Genes	GO_ENDOCYTIC_RECYCLING	1	0.6947824	0.93968874
Upregulated Genes	GO_POSITIVE_REGULATION_OF_STRIATED_MUSCLE_CELL_DIFFERENTIATION	1	0.69454324	0.93686354
Upregulated Genes	GO_REGULATION_OF_FERTILIZATION	1	0.6921935	0.9469548
Upregulated Genes	GO_OXIDATION_REDUCTION_PROCESS	19	0.6921407	0.8226221
Upregulated Genes	GO_REGULATION_OF_MRNA_CATABOLIC_PROCESS	2	0.690298	0.86247087
Upregulated Genes	GO_RESPONSE_TO_HYPEROXIA	2	0.6885899	0.87606835
Upregulated Genes	GO_DNA_METHYLATION_OR_DEMETHYLATION	4	0.6885254	0.83908045
Upregulated Genes	GO_CELLULAR_RESPONSE_TO_NITRIC_OXIDE	1	0.68849343	0.9675456
Upregulated Genes	GO_POSITIVE_REGULATION_OF_PROTEIN_POLYMERIZATION	2	0.68777424	0.89102566
Upregulated Genes	GO_RNA_DESTABILIZATION	2	0.68675673	0.88666666
Upregulated Genes	GO_MACROPHAGE_ACTIVATION	4	0.68660676	0.8390558
Upregulated Genes	GO_POSITIVE_REGULATION_OF_MRNA_CATABOLIC_PROCESS	2	0.68643785	0.8584071
Upregulated Genes	GO_POSITIVE_REGULATION_OF_ACTIN_FILAMENT_POLYMERIZATION	2	0.6837648	0.86157024
Upregulated Genes	GO_RESPONSE_TO_NITRIC_OXIDE	1	0.6837298	0.9725159
Upregulated Genes	GO_REGULATION_OF_FAT_CELL_DIFFERENTIATION	2	0.68333375	0.8842105
Upregulated Genes	GO_REGULATION_OF_PROTEIN_LOCALIZATION_TO_PLASMA_MEMBRANE	2	0.68227047	0.88650966
Upregulated Genes	GO_REGULATION_OF_CELLULAR_COMPONENT_BIOGENESIS	25	0.68045115	0.86
Upregulated Genes	GO_REGULATION_OF_PROTEASOMAL_UBIQUITIN_DEPENDENT_PROTEIN_CATABOLIC_PROCESS	1	0.68042135	0.9799197
Upregulated Genes	GO_CELLULAR_RESPONSE_TO_REACTIVE_NITROGEN_SPECIES	1	0.6777965	0.97556007
Upregulated Genes	GO_REGULATION_OF_PROTEIN_LOCALIZATION_TO_CELL_PERIPHERY	2	0.676621	0.8624454
Upregulated Genes	GO_PROTEIN_K63_LINKED_UBIQUITINATION	1	0.676428	0.9795082
Upregulated Genes	GO_REGULATION_OF_PROTEASOMAL_PROTEIN_CATABOLIC_PROCESS	1	0.6756569	0.9714286
Upregulated Genes	GO_FC_RECEPTOR_SIGNALING_PATHWAY	2	0.675161	0.88053095
Upregulated Genes	GO_POSITIVE_REGULATION_OF_SUPRAMOLECULAR_FIBER_ORGANIZATION	2	0.6734288	0.8898305
Upregulated Genes	GO_NEGATIVE_REGULATION_OF_NEURON_DIFFERENTIATION	4	0.6728264	0.8639309
Upregulated Genes	GO_TISSUE_HOMEOSTASIS	3	0.67109495	0.8590022
Upregulated Genes	GO_NUCLEOBASE_CONTAINING_SMALL_MOLECULE_CATABOLIC_PROCESS	1	0.670952	0.99793816
Upregulated Genes	GO_PYRIMIDINE_NUCLEOSIDE_CATABOLIC_PROCESS	1	0.66967714	0.9920477
Upregulated Genes	GO_REGULATION_OF_GENE_SILENCING	4	0.66953444	0.8835617
Upregulated Genes	GO_NUCLEOSIDE_CATABOLIC_PROCESS	1	0.66885984	0.99013805
Upregulated Genes	GO_GLYCOSYL_COMPOUND_CATABOLIC_PROCESS	1	0.6682719	0.99364406
Upregulated Genes	GO_FEMALE_SEX_DIFFERENTIATION	4	0.66753304	0.8765133
Upregulated Genes	GO_RESPONSE_TO_INCREASED_OXYGEN_LEVELS	2	0.66570556	0.8924051
Upregulated Genes	GO_MYELOID_LEUKOCYTE_MEDIATED_IMMUNITY	8	0.6646624	0.86585367
Upregulated Genes	GO_LOCOMOTORY_BEHAVIOR	4	0.6615052	0.87234044
Upregulated Genes	GO_NERVOUS_SYSTEM_PROCESS	12	0.66097885	0.8688119
Upregulated Genes	GO_REGULATION_OF_T_CELL_MEDIATED_IMMUNITY	2	0.6604058	0.90413946
Upregulated Genes	GO_PROTEIN_DEPHOSPHORYLATION	7	0.6588359	0.87439615
Upregulated Genes	GO_REGULATION_OF_AXONOGENESIS	4	0.65865993	0.9066667
Upregulated Genes	GO_DEVELOPMENT_OF_PRIMARY_FEMALE_SEXUAL_CHARACTERISTICS	4	0.65679085	0.8891304
Upregulated Genes	GO_MYELOID_DENDRITIC_CELL_ACTIVATION	2	0.65624887	0.90833336
Upregulated Genes	GO_RESPONSE_TO_ACID_CHEMICAL	2	0.65533745	0.9059081
Upregulated Genes	GO_NEGATIVE_REGULATION_OF_HEMOPOIESIS	2	0.654687	0.9100877
Upregulated Genes	GO_NEGATIVE_REGULATION_OF_ALPHA_BETA_T_CELL_ACTIVATION	2	0.654299	0.9189189
Upregulated Genes	GO_CENTRAL_NERVOUS_SYSTEM_NEURON_DIFFERENTIATION	3	0.6541335	0.8842795

Upregulated Genes	GO_NEGATIVE_REGULATION_OF_CELL_MORPHOGENESIS_INVOLVED_IN_DIFFERENTIATION	4	0.6536954	0.89767444
Upregulated Genes	GO_POSITIVE_REGULATION_OF_PEPTIDASE_ACTIVITY	2	0.6530992	0.9113402
Upregulated Genes	GO_NEGATIVE_REGULATION_OF_LEUKOCYTE_DIFFERENTIATION	2	0.65247536	0.88761467
Upregulated Genes	GO_NEGATIVE_REGULATION_OF_MAPK_CASCADE	5	0.6521103	0.85057473
Upregulated Genes	GO_MONOCYTE_CHEMOTAXIS	2	0.65195495	0.9053498
Upregulated Genes	GO_POSITIVE_REGULATION_OF_LEUKOCYTE_PROLIFERATION	5	0.65152663	0.87616825
Upregulated Genes	GO_REGULATION_OF_CYSTEINE_TYPE_ENDOPEPTIDASE_ACTIVITY	2	0.6511594	0.9161426
Upregulated Genes	GO_POSITIVE_REGULATION_OF_CHEMOKINE_PRODUCTION	2	0.65115595	0.92
Upregulated Genes	GO_DENDRITIC_CELL_DIFFERENTIATION	2	0.6492823	0.8898305
Upregulated Genes	GO_POSITIVE_REGULATION_OF_DNA_BINDING_TRANSCRIPTION_FACTOR_ACTIVITY	5	0.64808077	0.8953771
Upregulated Genes	GO_CHEMOKINE_PRODUCTION	2	0.6479947	0.92339545
Upregulated Genes	GO_NEGATIVE_REGULATION_OF_T_CELL_DIFFERENTIATION	2	0.64624965	0.90838206
Upregulated Genes	GO_REGULATION_OF_TOLERANCE_INDUCTION	2	0.6459111	0.9361234
Upregulated Genes	GO_NEGATIVE_REGULATION_OF_DEVELOPMENTAL_GROWTH	4	0.64564687	0.89237666
Upregulated Genes	GO_MICROTUBULE_POLYMERIZATION	4	0.6450855	0.8957871
Upregulated Genes	GO_POSITIVE_REGULATION_OF_INTERLEUKIN_1_PRODUCTION	2	0.6427498	0.89419085
Upregulated Genes	GO_NEGATIVE_REGULATION_OF_CD4_POSITIVE_ALPHA_BETA_T_CELL_DIFFERENTIATION	2	0.6427254	0.9052864
Upregulated Genes	GO_TOLERANCE_INDUCTION	2	0.6419452	0.9107505
Upregulated Genes	GO_NEGATIVE_REGULATION_OF_LYMPHOCYTE_DIFFERENTIATION	2	0.6388713	0.9190871
Upregulated Genes	GO_POSITIVE_REGULATION_OF_CYSTEINE_TYPE_ENDOPEPTIDASE_ACTIVITY	2	0.63860315	0.9197397
Upregulated Genes	GO_POSITIVE_REGULATION_OF_INTERLEUKIN_10_PRODUCTION	2	0.6383135	0.9223947
Upregulated Genes	GO_ACTIVATED_T_CELL_PROLIFERATION	2	0.6382667	0.9202454
Upregulated Genes	GO_ORGANOPHOSPHATE_BIOSYNTHETIC_PROCESS	9	0.63728064	0.8924485
Upregulated Genes	GO_POSITIVE_REGULATION_OF_CELL_ACTIVATION	10	0.63416684	0.8816425
Upregulated Genes	GO_MOLTING_CYCLE	3	0.6333503	0.9072398
Upregulated Genes	GO_EMBRYONIC_ORGAN_MORPHOGENESIS	2	0.6322243	0.9145299
Upregulated Genes	GO_NEGATIVE_REGULATION_OF_CELL_GROWTH	3	0.6315187	0.90174675
Upregulated Genes	GO_EMBRYONIC_HEART_TUBE_DEVELOPMENT	2	0.6315046	0.91722596
Upregulated Genes	GO_NEGATIVE_REGULATION_OF_ALPHA_BETA_T_CELL_DIFFERENTIATION	2	0.63077086	0.9208925
Upregulated Genes	GO_NEGATIVE_REGULATION_OF_CD4_POSITIVE_ALPHA_BETA_T_CELL_ACTIVATION	2	0.6305035	0.9183673
Upregulated Genes	GO_PROTEIN_MODIFICATION_BY_SMALL_PROTEIN_CONJUGATION	20	0.6282442	0.8919598
Upregulated Genes	GO_SPECIFICATION_OF_SYMMETRY	2	0.62716264	0.92389005
Upregulated Genes	GO_EMBRYONIC_HEART_TUBE_MORPHOGENESIS	2	0.626995	0.9352818
Upregulated Genes	GO_CELLULAR_RESPONSE_TO_UV	2	0.62544733	0.9353448
Upregulated Genes	GO_POSITIVE_REGULATION_OF_NUCLEOBASE_CONTAINING_COMPOUND_METABOLIC_PROCES	33	0.6253903	0.92582417
Upregulated Genes	GO_PROTEIN_HOMOOLIGOMERIZATION	6	0.62529904	0.8997555
Upregulated Genes	GO_EMBRYONIC_PATTERN_SPECIFICATION	2	0.6246889	0.91121495
Upregulated Genes	GO_CELLULAR_RESPONSE_TO_LIGHT_STIMULUS	2	0.61964476	0.92967033
Upregulated Genes	GO_AMEBOIDAL_TYPE_CELL_MIGRATION	11	0.61547005	0.91219515
Upregulated Genes	GO_POST_TRANSLATIONAL_PROTEIN_MODIFICATION	9	0.60944843	0.91803277
Upregulated Genes	GO_TISSUE_MIGRATION	8	0.608467	0.9009434
Upregulated Genes	GO_RESPONSE_TO_ESTROGEN	3	0.6054662	0.9221968
Upregulated Genes	GO_INTERLEUKIN_8_PRODUCTION	4	0.60233724	0.9521531
Upregulated Genes	GO_POSITIVE_REGULATION_OF_CYTOSKELETON_ORGANIZATION	4	0.6011172	0.9369159
Upregulated Genes	GO_STEROID_HORMONE_MEDIATED_SIGNALING_PATHWAY	3	0.5987038	0.93273544
Upregulated Genes	GO_REGULATION_OF_EXTENT_OF_CELL_GROWTH	3	0.592773	0.92600423
Upregulated Genes	GO_REGULATION_OF_POSTTRANSCRIPTIONAL_GENE_SILENCING	3	0.59219164	0.9504505
Upregulated Genes	GO_NEURON_PROJECTION_EXTENSION	3	0.5876688	0.92825115
Upregulated Genes	GO_INTRACELLULAR_STEROID_HORMONE_RECEPTOR_SIGNALING_PATHWAY	3	0.5845982	0.930131
Upregulated Genes	GO_NEUROBLAST_PROLIFERATION	3	0.57704616	0.9440716
Upregulated Genes	GO_NEGATIVE_REGULATION_OF_NEURON_PROJECTION_DEVELOPMENT	3	0.57648027	0.93166286
Upregulated Genes	GO_NEGATIVE_REGULATION_OF_AXONOGENESIS	3	0.57615495	0.9477124
Upregulated Genes	GO_AXON_EXTENSION	3	0.5732953	0.9471366
Upregulated Genes	GO_NEURAL_PRECURSOR_CELL_PROLIFERATION	3	0.5732142	0.9632035
Upregulated Genes	GO_NEGATIVE_REGULATION_OF_CELL_PROJECTION_ORGANIZATION	3	0.5710799	0.94057375
Upregulated Genes	GO_FOREBRAIN_NEURON_DIFFERENTIATION	2	0.56396914	0.96588486
Upregulated Genes	GO_T_CELL_SELECTION	2	0.5636253	0.9741936
Upregulated Genes	GO_REGULATION_OF_WNT_SIGNALING_PATHWAY	8	0.5620629	0.9381443
Upregulated Genes	GO_CAMERA_TYPE_EYE_DEVELOPMENT	2	0.55225974	0.9752066
Upregulated Genes	GO_FOREBRAIN_GENERATION_OF_NEURONS	2	0.5490584	0.972103
Upregulated Genes	GO_NEGATIVE_REGULATION_OF_CATALYTIC_ACTIVITY	13	0.54644144	0.9419192
Upregulated Genes	GO_POSITIVE_T_CELL_SELECTION	2	0.54636985	0.9714286
Upregulated Genes	GO_CELLULAR_RESPONSE_TO_DEXAMETHASONE_STIMULUS	2	0.5345297	0.9822222
Upregulated Genes	GO_RESPONSE_TO_COCAINE	2	0.5336312	0.9788136
Upregulated Genes	GO_NUCLEOBASE_METABOLIC_PROCESS	4	0.5224857	0.98571426
Upregulated Genes	GO_CELL_MORPHOGENESIS_INVOLVED_IN_DIFFERENTIATION	11	0.5206486	0.96733665
Upregulated Genes	GO_MAMMARY_GLAND_MORPHOGENESIS	2	0.50425476	1
Upregulated Genes	GO_REGULATION_OF_INTRACELLULAR_ESTROGEN_RECEPTOR_SIGNALING_PATHWAY	2	0.5004275	1
Upregulated Genes	GO_BRANCHING_MORPHOGENESIS_OF_AN_EPITHELIAL_TUBE	2	0.49720648	0.9977876
Upregulated Genes	GO_INTRACELLULAR_ESTROGEN_RECEPTOR_SIGNALING_PATHWAY	2	0.49672318	1

Upregulated Genes	GO_MORPHOGENESIS_OF_A_BRANCHING_STRUCTURE	2	0.49407676	0.99557525
Upregulated Genes	GO_MAINTENANCE_OF_LOCATION	5	0.49354413	0.9888143
Upregulated Genes	GO_REGULATION_OF_INTRACELLULAR_STEROID_HORMONE_RECEPTOR_SIGNALING_PATHWAY	2	0.48996946	0.99785864
Upregulated Genes	GO_RNA_PHOSPHODIESTER_BOND_HYDROLYSIS	4	0.46813473	0.989083
Upregulated Genes	GO_REGULATION_OF_ANATOMICAL_STRUCTURE_SIZE	10	0.46695444	0.9804878
Upregulated Genes	GO_TRANSMEMBRANE_RECEPTOR_PROTEIN_TYROSINE_KINASE_SIGNALING_PATHWAY	6	0.4256239	0.99285716
