# Supplementary material for: Estrogen Receptor-Regulated Gene Signatures in Invasive Breast Cancer Cells and Aggressive Breast Tumors
Source: Cancers (Basel). 2022 Jun 9;14(12):2848. doi: 10.3390/cancers14122848 (PMC9221274; doi:10.3390/cancers14122848)
Supplement: Supplementary file 1 [file cancers-14-02848-s001.zip › Table S3.pdf]

Table S3: List of ER regulated genes forming signature 2 in ER and IKKβ co-activated invasive MCF-7 cells

ENS Gene ID	Gene	FC	FDR
ENSG00000116260	QSOX1	0.66	0.00
ENSG00000198892	SHISA4	1.20	0.00
ENSG00000184897	H1FX	-0.66	0.00
ENSG00000102265	TIMP1	0.61	0.00
ENSG00000138119	MYOF	0.56	0.00
ENSG00000134970	TMED7	-1.11	0.00
ENSG00000142089	IFITM3	0.40	0.00
ENSG00000173848	NET1	-0.80	0.00
ENSG00000165272	AQP3	1.17	0.00
ENSG00000125505	MBOAT7	0.37	0.00
ENSG00000143742	SRP9	-1.13	0.00
ENSG00000103855	CD276	0.43	0.00
ENSG00000102034	ELF4	0.53	0.00
ENSG00000125844	RRBP1	0.39	0.00
ENSG00000187720	THSD4	0.44	0.00
ENSG00000268104	SLC6A14	-4.81	0.00
ENSG00000198910	L1CAM	0.51	0.00
ENSG00000197045	GMFB	-0.71	0.00
ENSG00000127152	BCL11B	-0.78	0.00
ENSG00000185347	C14orf80	-0.56	0.00
ENSG00000243279	PRAF2	0.56	0.00
ENSG00000110492	MDK	0.39	0.00
ENSG00000151239	TWF1	-0.65	0.00
ENSG00000170477	KRT4	-1.60	0.00
ENSG00000178209	PLEC	0.66	0.00
ENSG00000116299	KIAA1324	-0.60	0.00
ENSG00000205269	TMEM170B	-1.07	0.00
ENSG00000100744	GSKIP	-0.64	0.00
ENSG00000169504	CLIC4	-0.43	0.00
ENSG00000166908	PIP4K2C	0.46	0.00
ENSG00000151176	PLBD2	0.38	0.00
ENSG00000142632	ARHGEF19	-0.82	0.00
ENSG00000213390	ARHGAP19	-1.03	0.00
ENSG00000169139	UBE2V2	-0.61	0.00
ENSG00000006062	MAP3K14	0.63	0.00
ENSG00000012171	SEMA3B	-0.81	0.00
ENSG00000198467	TPM2	0.46	0.00
ENSG00000182985	CADM1	0.94	0.00
ENSG00000147687	TATDN1	-0.49	0.00
ENSG00000134775	FHOD3	-1.06	0.00
ENSG00000120063	GNA13	-0.44	0.00
ENSG00000171522	PTGER4	1.89	0.00
ENSG00000104853	CLPTM1	0.34	0.00
ENSG00000076770	MBNL3	-0.85	0.00
ENSG00000143578	CREB3L4	-0.83	0.00

ENSG00000198252	STYX	-0.46	0.00
ENSG00000069011	PITX1	-0.39	0.00
ENSG00000123989	CHPF	0.47	0.00
ENSG00000165997	ARL5B	-0.65	0.00
ENSG00000198598	MMP17	0.45	0.00
ENSG00000104442	ARMC1	-0.70	0.00
ENSG00000180776	ZDHHC20	-0.48	0.00
ENSG00000178695	KCTD12	1.50	0.00
ENSG00000169710	FASN	0.53	0.00
ENSG00000101911	PRPS2	-0.51	0.00
ENSG00000137203	TFAP2A	0.48	0.00
ENSG00000205476	CCDC85C	-0.33	0.00
ENSG00000174327	SLC16A13	0.51	0.00
ENSG00000139726	DENR	-0.40	0.00
ENSG00000157851	DPYSL5	-0.71	0.00
ENSG00000085998	POMGNT1	0.35	0.00
ENSG00000213281	NRAS	-0.35	0.00
ENSG00000115419	GLS	-0.45	0.00
ENSG00000134049	IER3IP1	-1.15	0.00
ENSG00000133657	ATP13A3	-0.39	0.00
ENSG00000076554	TPD52	-0.54	0.00
ENSG00000137876	RSL24D1	-0.44	0.00
ENSG00000108602	ALDH3A1	0.86	0.00
ENSG00000175061	LRRC75A-AS1	-0.26	0.00
ENSG00000100319	ZMAT5	0.48	0.00
ENSG00000233198	RNF224	1.09	0.00
ENSG00000126458	RRAS	-0.57	0.00
ENSG00000122778	KIAA1549	0.44	0.00
ENSG00000204539	CDSN	1.07	0.00
ENSG00000148143	ZNF462	0.37	0.00
ENSG00000173064	HECTD4	0.37	0.00
ENSG00000123728	RAP2C	-0.40	0.00
ENSG00000086300	SNX10	-1.02	0.00
ENSG00000120992	LYPLA1	-0.52	0.00
ENSG00000137770	CTDSPL2	-0.51	0.00
ENSG00000074181	NOTCH3	0.32	0.00
ENSG00000257923	CUX1	0.27	0.00
ENSG00000000460	C1orf112	-0.74	0.00
ENSG00000145555	MYO10	0.35	0.00
ENSG00000091136	LAMB1	0.34	0.00
ENSG00000189266	PNRC2	-0.50	0.00
ENSG00000167595	PROSER3	0.46	0.00
ENSG00000112081	SRSF3	-0.41	0.00
ENSG00000196220	SRGAP3	-0.73	0.00
ENSG00000146729	GBAS	-0.38	0.00
ENSG00000151366	NDUFC2	-0.89	0.00
ENSG00000186638	KIF24	-1.17	0.00

ENSG00000197982	C1orf122	0.41	0.00
ENSG00000149257	SERPINH1	0.26	0.00
ENSG00000103005	USB1	0.34	0.00
ENSG00000177425	PAWR	-0.34	0.00
ENSG00000261068	RP11-7K24.3	1.22	0.00
ENSG00000174567	GOLT1A	0.53	0.00
ENSG00000108821	COL1A1	0.82	0.00
ENSG00000168264	IRF2BP2	-0.34	0.00
ENSG00000179776	CDH5	0.85	0.00
ENSG00000136167	LCP1	0.70	0.00
ENSG00000074527	NTN4	0.44	0.00
ENSG00000013288	MAN2B2	0.33	0.00
ENSG00000142798	HSPG2	0.36	0.00
ENSG00000171262	FAM98B	-0.44	0.00
ENSG00000120725	SIL1	0.45	0.00
ENSG00000162368	CMPK1	-0.36	0.00
ENSG00000154978	VOPP1	0.36	0.00
ENSG00000149541	B3GAT3	0.41	0.00
ENSG00000175785	PRIMA1	-1.41	0.00
ENSG00000148396	SEC16A	0.27	0.00
ENSG00000174130	TLR6	-0.82	0.00
ENSG00000116209	TMEM59	0.41	0.00
ENSG00000165731	RET	0.36	0.00
ENSG00000083444	PLOD1	0.31	0.00
ENSG00000180998	GPR137C	-0.89	0.00
ENSG00000101294	HM13	0.32	0.00
ENSG00000138092	CENPO	-0.49	0.00
ENSG00000164889	SLC4A2	0.26	0.00
ENSG00000177034	MTX3	-0.51	0.00
ENSG00000173598	NUDT4	-0.49	0.00
ENSG00000135677	GNS	0.30	0.00
ENSG00000138777	PPA2	-0.38	0.00
ENSG00000173894	CBX2	-0.33	0.00
ENSG00000180817	PPA1	-0.30	0.00
ENSG00000065665	SEC61A2	0.58	0.00
ENSG00000130600	H19	-2.16	0.00
ENSG00000176105	YES1	-0.39	0.00
ENSG00000109762	SNX25	0.59	0.00
ENSG00000197081	IGF2R	0.41	0.00
ENSG00000101417	PXMP4	-0.47	0.00
ENSG00000196126	HLA-DRB1	3.45	0.00
ENSG00000127418	FGFRL1	0.32	0.00
ENSG00000197070	ARRDC1	0.31	0.00
ENSG00000153944	MSI2	-0.39	0.00
ENSG00000163703	CRELD1	0.52	0.00
ENSG00000152413	HOMER1	-0.46	0.00
ENSG00000124191	TOX2	-0.49	0.00

ENSG00000178074	C2orf69	-0.58	0.00
ENSG00000130159	ECSIT	-0.42	0.00
ENSG00000157637	SLC38A10	0.33	0.00
ENSG00000135486	HNRNPA1	-0.27	0.00
ENSG00000241399	CD302	-0.68	0.00
ENSG00000125746	EML2	0.42	0.00
ENSG00000237973	MTCO1P12	0.49	0.00
ENSG00000127507	ADGRE2	0.62	0.00
ENSG00000091129	NRCAM	0.32	0.00
ENSG00000126453	BCL2L12	-0.45	0.00
ENSG00000112964	GHR	-0.54	0.00
ENSG00000163743	RCHY1	-0.60	0.00
ENSG00000090372	STRN4	0.27	0.00
ENSG00000176912	TYMSOS	-1.38	0.00
ENSG00000131037	EPS8L1	0.37	0.00
ENSG00000120137	PANK3	-0.38	0.00
ENSG00000109881	CCDC34	-0.72	0.00
ENSG00000129317	PUS7L	-0.61	0.00
ENSG00000008300	CELSR3	0.29	0.00
ENSG00000101888	NXT2	-0.43	0.00
ENSG00000187583	PLEKHN1	0.50	0.00
ENSG00000135245	HILPDA	-0.72	0.00
ENSG00000067167	TRAM1	-0.31	0.00
ENSG00000121068	TBX2	0.36	0.00
ENSG00000143486	EIF2D	-0.33	0.00
ENSG00000203668	CHML	-0.52	0.00
ENSG00000120896	SORBS3	-0.32	0.00
ENSG00000198569	SLC34A3	1.82	0.00
ENSG00000034677	RNF19A	-0.34	0.00
ENSG00000136521	NDUFB5	-0.33	0.00
ENSG00000165929	TC2N	-0.34	0.00
ENSG00000275052	PPP4R3B	-0.44	0.00
ENSG00000184990	SIVA1	-0.31	0.00
ENSG00000180008	SOCS4	-0.39	0.00
ENSG00000153130	SCOC	-0.53	0.00
ENSG00000177565	TBL1XR1	-0.35	0.00
ENSG00000165832	TRUB1	-0.42	0.00
ENSG00000164976	KIAA1161	0.31	0.00
ENSG00000005486	RHBDD2	0.34	0.00
ENSG00000144320	LNPK	-0.50	0.00
ENSG00000072682	P4HA2	0.42	0.00
ENSG00000160298	C21orf58	-0.78	0.00
ENSG00000171903	CYP4F11	0.50	0.00
ENSG00000203485	INF2	0.31	0.00
ENSG00000139624	CERS5	0.38	0.00
ENSG00000188610	FAM72B	-1.85	0.00
ENSG00000163975	MELTF	0.64	0.00

ENSG00000100796	PPP4R3A	-0.26	0.00
ENSG00000183248	PRR36	0.36	0.00
ENSG00000271383	NBPF19	0.57	0.00
ENSG00000149418	ST14	0.25	0.00
ENSG00000049283	EPN3	-0.33	0.00
ENSG00000050344	NFE2L3	-0.32	0.00
ENSG00000147471	PROSC	-0.59	0.00
ENSG00000164403	SHROOM1	0.71	0.00
ENSG00000243646	IL10RB	0.46	0.00
ENSG00000198931	APRT	0.27	0.00
ENSG00000197472	ZNF695	-1.34	0.00
ENSG00000198142	SOWAHC	-0.40	0.00
ENSG00000089050	RBBP9	-0.50	0.00
ENSG00000143811	PYCR2	0.30	0.00
ENSG00000135506	OS9	0.27	0.00
ENSG00000133393	FOPNL	-0.58	0.00
ENSG00000099783	HNRNPM	-0.33	0.00
ENSG00000180385	EMC3-AS1	-0.55	0.00
ENSG00000196923	PDLIM7	0.29	0.00
ENSG00000178177	LCORL	-0.85	0.00
ENSG00000109332	UBE2D3	-0.28	0.00
ENSG00000171928	TVP23B	-0.48	0.00
ENSG00000198146	ZNF770	-0.40	0.00
ENSG00000125971	DYNLRB1	0.33	0.00
ENSG00000170899	GSTA4	-1.98	0.00
ENSG00000274276	CBSL	1.33	0.00
ENSG00000137198	GMPR	1.02	0.00
ENSG00000122367	LDB3	0.70	0.00
ENSG00000101337	TM9SF4	0.27	0.00
ENSG00000152689	RASGRP3	1.44	0.00
ENSG00000151445	VIPAS39	0.31	0.00
ENSG00000138835	RGS3	-0.52	0.00
ENSG00000172270	BSG	0.27	0.00
ENSG00000027847	B4GALT7	0.33	0.00
ENSG00000163001	CFAP36	0.41	0.00
ENSG00000163605	PPP4R2	-0.39	0.00
ENSG00000136244	IL6	0.57	0.00
ENSG00000204536	CCHCR1	-0.47	0.00
ENSG00000023041	ZDHHC6	-0.41	0.00
ENSG00000138642	HERC6	0.78	0.00
ENSG00000168724	DNAJC21	-0.29	0.00
ENSG00000183943	PRKX	-0.30	0.00
ENSG00000197948	FCHSD1	0.58	0.00
ENSG00000112414	ADGRG6	-0.48	0.00
ENSG00000204370	SDHD	-0.83	0.00
ENSG00000197150	ABCB8	0.30	0.00
ENSG00000197006	METTL9	-0.26	0.00

ENSG00000121897	LIAS	-0.73	0.00
ENSG00000157368	IL34	0.86	0.00
ENSG00000118564	FBXL5	-0.55	0.00
ENSG00000103064	SLC7A6	0.28	0.00
ENSG00000152056	AP1S3	-0.45	0.00
ENSG00000103507	BCKDK	-0.31	0.00
ENSG00000151632	AKR1C2	-1.22	0.00
ENSG00000104723	TUSC3	0.29	0.00
ENSG00000196678	ERI2	-0.89	0.00
ENSG00000128422	KRT17	-0.50	0.00
ENSG00000213399	AC022210.2	-0.53	0.00
ENSG00000108506	INTS2	-0.29	0.00
ENSG00000166200	COPS2	-0.46	0.00
ENSG00000149428	HYOU1	0.35	0.00
ENSG00000033627	ATP6V0A1	0.36	0.00
ENSG00000198546	ZNF511	-0.43	0.00
ENSG00000099308	MAST3	0.33	0.00
ENSG00000103512	NOMO1	0.27	0.00
ENSG00000116747	TROVE2	-0.32	0.00
ENSG00000108375	RNF43	-0.52	0.00
ENSG00000130762	ARHGEF16	0.33	0.00
ENSG00000124839	RAB17	0.33	0.00
ENSG00000090863	GLG1	0.29	0.00
ENSG00000196132	MYT1	0.39	0.00
ENSG00000070404	FSTL3	0.43	0.00
ENSG00000130304	SLC27A1	0.53	0.00
ENSG00000108828	VAT1	0.25	0.00
ENSG00000168813	ZNF507	-0.47	0.00
ENSG00000151748	SAV1	-0.43	0.00
ENSG00000197226	TBC1D9B	0.29	0.00
ENSG00000150054	MPP7	-0.52	0.00
ENSG00000117226	GBP3	2.09	0.00
ENSG00000023445	BIRC3	-0.33	0.00
ENSG00000001617	SEMA3F	0.32	0.00
ENSG00000163513	TGFBR2	0.60	0.00
ENSG00000079277	MKNK1	0.32	0.00
ENSG00000134900	TPP2	-0.42	0.00
ENSG00000141349	G6PC3	0.35	0.00
ENSG00000156599	ZDHHC5	0.27	0.00
ENSG00000131094	C1QL1	-1.18	0.00
ENSG00000085511	MAP3K4	-0.52	0.00
ENSG00000197594	ENPP1	-0.42	0.00
ENSG00000123179	EBPL	-0.47	0.00
ENSG00000111615	KRR1	-0.34	0.00
ENSG00000138356	AOX1	-0.75	0.00
ENSG00000081760	AACS	0.29	0.00
ENSG00000104408	EIF3E	-0.27	0.00

ENSG00000147853	AK3	-0.26	0.00
ENSG00000169740	ZNF32	-0.46	0.00
ENSG00000105619	TFPT	0.29	0.00
ENSG00000020129	NCDN	0.26	0.00
ENSG00000119953	SMNDC1	-0.36	0.00
ENSG00000128311	TST	-0.33	0.00
ENSG00000163040	CCDC74A	-0.78	0.00
ENSG00000204434	POTEKP	0.97	0.00
ENSG00000064601	CTSA	0.34	0.00
ENSG00000162298	SYVN1	0.26	0.00
ENSG00000130479	MAP1S	0.30	0.00
ENSG00000139746	RBM26	-0.28	0.00
ENSG00000135297	MTO1	-0.40	0.00
ENSG00000203879	GDI1	0.37	0.00
ENSG00000132824	SERINC3	0.25	0.00
ENSG00000144228	SPOPL	-0.34	0.00
ENSG00000140545	MFGE8	0.48	0.00
ENSG00000135374	ELF5	-1.54	0.00
ENSG00000146281	PM20D2	-0.52	0.00
ENSG00000126016	AMOT	-0.40	0.00
ENSG00000082512	TRAF5	0.53	0.00
ENSG00000136051	WASHC4	-0.32	0.00
ENSG00000164054	SHISA5	0.33	0.00
ENSG00000111832	RWDD1	-0.34	0.00
ENSG00000167548	KMT2D	0.43	0.00
ENSG00000241343	RPL36A	0.57	0.00
ENSG00000116191	RALGPS2	-0.56	0.00
ENSG00000145740	SLC30A5	-0.29	0.00
ENSG00000116455	WDR77	-0.27	0.00
ENSG00000129993	CBFA2T3	0.28	0.00
ENSG00000105088	OLFM2	0.31	0.00
ENSG00000166685	COG1	0.31	0.00
ENSG00000164574	GALNT10	0.31	0.00
ENSG00000138750	NUP54	-0.40	0.00
ENSG00000058673	ZC3H11A	1.23	0.00
ENSG00000116774	OLFML3	1.12	0.00
ENSG00000035664	DAPK2	-0.75	0.00
ENSG00000155256	ZFYVE27	0.45	0.00
ENSG00000053747	LAMA3	0.91	0.00
ENSG00000155959	VBP1	-0.62	0.00
ENSG00000115596	WNT6	-0.57	0.00
ENSG00000167994	RAB3IL1	-0.53	0.00
ENSG00000187522	HSPA14	-0.36	0.00
ENSG00000169592	INO80E	-0.30	0.00
ENSG00000155629	PIK3AP1	-1.41	0.00
ENSG00000064042	LIMCH1	0.28	0.00
ENSG00000101210	EEF1A2	0.29	0.00

ENSG00000163507	KIAA1524	-0.87	0.00
ENSG00000154654	NCAM2	-0.59	0.00
ENSG00000106689	LHX2	-1.13	0.00
ENSG00000135045	C9orf40	-0.57	0.00
ENSG00000161677	JOSD2	0.30	0.00
ENSG00000037280	FLT4	-1.16	0.00
ENSG00000163541	SUCLG1	-0.26	0.00
ENSG00000161960	EIF4A1	1.16	0.00
ENSG00000196961	AP2A1	0.26	0.00
ENSG00000165934	CPSF2	-0.35	0.00
ENSG00000162341	TPCN2	0.36	0.00
ENSG00000114744	COMMD2	-0.46	0.00
ENSG00000135740	SLC9A5	0.84	0.00
ENSG00000116701	NCF2	0.74	0.00
ENSG00000281162	LINC01127	4.78	0.00
ENSG00000142669	SH3BGRL3	0.34	0.00
ENSG00000129353	SLC44A2	0.26	0.00
ENSG00000162961	DPY30	-0.32	0.00
ENSG00000139668	WDFY2	-0.67	0.00
ENSG00000244176	RP11-810P12.1	2.89	0.00
ENSG00000213160	KLHL23	-0.50	0.00
ENSG00000163428	LRRC58	-0.60	0.00
ENSG00000166452	AKIP1	-0.54	0.00
ENSG00000178718	RPP25	-0.26	0.00
ENSG00000129480	DTD2	-0.46	0.00
ENSG00000109089	CDR2L	-0.27	0.00
ENSG00000167978	SRRM2	0.29	0.00
ENSG00000113966	ARL6	-1.27	0.00
ENSG00000175606	TMEM70	-0.42	0.00
ENSG00000185745	IFIT1	2.10	0.00
ENSG00000119729	RHOQ	-0.33	0.00
ENSG00000196352	CD55	0.39	0.00
ENSG00000188428	BLOC1S5	-1.04	0.00
ENSG00000145476	CYP4V2	0.79	0.00
ENSG00000105707	HPN	0.51	0.00
ENSG00000036054	TBC1D23	-0.42	0.00
ENSG00000177675	CD163L1	1.41	0.00
ENSG00000159086	PAXBP1	-0.45	0.00
ENSG00000143412	ANXA9	0.51	0.00
ENSG00000219797	PPIAP9	-2.47	0.00
ENSG00000125901	MRPS26	-0.26	0.00
ENSG00000107951	MTPAP	-0.30	0.00
ENSG00000043093	DCUN1D1	-0.33	0.00
ENSG00000102554	KLF5	-0.26	0.00
ENSG00000171227	TMEM37	-0.73	0.00
ENSG00000085365	SCAMP1	-0.42	0.00
ENSG00000070214	SLC44A1	-0.27	0.00

ENSG00000158711	ELK4	-0.26	0.00
ENSG00000025772	TOMM34	0.25	0.00
ENSG00000184731	FAM110C	0.45	0.00
ENSG00000008282	SYPL1	-0.27	0.00
ENSG00000176624	MEX3C	-0.33	0.00
ENSG00000112210	RAB23	-0.58	0.00
ENSG00000113658	SMAD5	-0.29	0.00
ENSG00000069974	RAB27A	-0.44	0.00
ENSG00000115540	MOB4	-0.39	0.00
ENSG00000116791	CRYZ	-0.33	0.00
ENSG00000011347	SYT7	-0.28	0.00
ENSG00000182196	ARL6IP4	1.51	0.00
ENSG00000118418	HMGN3	-0.39	0.00
ENSG00000111962	UST	0.53	0.00
ENSG00000033170	FUT8	0.29	0.00
ENSG00000156504	FAM122B	-0.29	0.00
ENSG00000176834	VSIG10	-0.37	0.00
ENSG00000249395	CASC9	-1.40	0.00
ENSG00000142655	PEX14	0.33	0.00
ENSG00000158258	CLSTN2	-0.67	0.00
ENSG00000164211	STARD4	0.76	0.00
ENSG00000196338	NLGN3	1.04	0.00
ENSG00000169071	ROR2	0.29	0.00
ENSG00000171680	PLEKHG5	0.32	0.00
ENSG00000106355	LSM5	-0.59	0.00
ENSG00000104765	BNIP3L	-0.43	0.00
ENSG00000078177	N4BP2	-0.57	0.00
ENSG00000129636	ITFG1	0.29	0.00
ENSG00000075303	SLC25A40	-0.57	0.00
ENSG00000111880	RNGTT	-0.31	0.00
ENSG00000275342	PRAG1	0.37	0.00
ENSG00000151729	SLC25A4	0.34	0.00
ENSG00000152291	TGOLN2	0.26	0.00
ENSG00000182580	EPHB3	-0.51	0.00
ENSG00000038210	PI4K2B	-0.52	0.00
ENSG00000177697	CD151	0.25	0.00
ENSG00000105429	MEGF8	0.30	0.00
ENSG00000149927	DOC2A	-0.60	0.00
ENSG00000146757	ZNF92	-0.56	0.00
ENSG00000174227	PIGG	0.37	0.00
ENSG00000260565	ERVK13-1	0.63	0.00
ENSG00000261061	RP11-303E16.2	-0.70	0.00
ENSG00000160953	MUM1	0.28	0.00
ENSG00000167880	EVPL	0.31	0.01
ENSG00000169851	PCDH7	4.55	0.01
ENSG00000259882	RP11-293B20.2	3.86	0.01
ENSG00000104047	DTWD1	-0.42	0.01

ENSG00000076864	RAP1GAP	0.29	0.01
ENSG00000135052	GOLM1	0.35	0.01
ENSG00000144034	TPRKB	-0.66	0.01
ENSG00000181392	SYNE4	0.35	0.01
ENSG00000141298	SSH2	0.42	0.01
ENSG00000249158	PCDHA11	0.35	0.01
ENSG00000172771	EFCAB12	2.68	0.01
ENSG00000121966	CXCR4	-1.22	0.01
ENSG00000183688	RFLNB	-0.61	0.01
ENSG00000125966	MMP24	-0.57	0.01
ENSG00000181830	SLC35C1	0.31	0.01
ENSG00000141741	MIEN1	0.25	0.01
ENSG00000100949	RABGGTA	0.31	0.01
ENSG00000065534	MYLK	-0.98	0.01
ENSG00000101955	SRPX	-4.51	0.01
ENSG00000223855	HRAT92	1.63	0.01
ENSG00000242114	MTFP1	-0.46	0.01
ENSG00000136156	ITM2B	0.31	0.01
ENSG00000141026	MED9	-0.38	0.01
ENSG00000137261	KIAA0319	0.96	0.01
ENSG00000169814	BTD	0.40	0.01
ENSG00000127334	DYRK2	-0.33	0.01
ENSG00000075089	ACTR6	-0.51	0.01
ENSG00000153237	CCDC148	1.21	0.01
ENSG00000204634	TBC1D8	0.41	0.01
ENSG00000267321	LINC02001	-0.74	0.01
ENSG00000196247	ZNF107	-0.58	0.01
ENSG00000277142	LINC00235	-1.06	0.01
ENSG00000141985	SH3GL1	0.35	0.01
ENSG00000106266	SNX8	0.29	0.01
ENSG00000179715	PCED1B	0.75	0.01
ENSG00000243660	ZNF487	-0.82	0.01
ENSG00000135605	TEC	1.03	0.01
ENSG00000181061	HIGD1A	-0.37	0.01
ENSG00000168228	ZCCHC4	0.74	0.01
ENSG00000241962	RP11-111H13.1	-2.64	0.01
ENSG00000197965	MPZL1	0.26	0.01
ENSG00000165660	FAM175B	-0.37	0.01
ENSG00000214110	LDHAP4	-0.53	0.01
ENSG00000126351	THRA	-0.28	0.01
ENSG00000243024	RPS11P6	4.31	0.01
ENSG00000151917	BEND6	-0.53	0.01
ENSG00000136383	ALPK3	0.90	0.01
ENSG00000172731	LRRC20	-0.55	0.01
ENSG00000101935	AMMECR1	-0.34	0.01
ENSG00000186687	LYRM7	-0.62	0.01
ENSG00000068305	MEF2A	0.37	0.01

ENSG00000104447	TRPS1	0.28	0.01
ENSG00000111325	OGFOD2	0.69	0.01
ENSG00000088035	ALG6	-0.68	0.01
ENSG00000196542	SPTSSB	-0.61	0.01
ENSG00000170523	KRT83	-1.54	0.01
ENSG00000018610	CXorf56	-0.40	0.01
ENSG00000108474	PIGL	0.55	0.01
ENSG00000165887	ANKRD2	-0.97	0.01
ENSG00000204540	PSORS1C1	0.67	0.01
ENSG00000163481	RNF25	0.30	0.01
ENSG00000143942	CHAC2	-1.16	0.01
ENSG00000153395	LPCAT1	0.37	0.01
ENSG00000114631	PODXL2	0.27	0.01
ENSG00000165156	ZHX1	-0.31	0.01
ENSG00000138688	KIAA1109	0.34	0.01
ENSG00000152217	SETBP1	1.23	0.01
ENSG00000169692	AGPAT2	0.31	0.01
ENSG00000274717	RP1-47A17.1	3.87	0.01
ENSG00000168116	KIAA1586	-0.64	0.01
ENSG00000107331	ABCA2	0.31	0.01
ENSG00000177119	ANO6	0.42	0.01
ENSG00000065308	TRAM2	0.32	0.01
ENSG00000137947	GTF2B	0.55	0.01
ENSG00000107758	PPP3CB	-0.41	0.01
