# Supplementary material for: Estrogen Receptor-Regulated Gene Signatures in Invasive Breast Cancer Cells and Aggressive Breast Tumors
Source: Cancers (Basel). 2022 Jun 9;14(12):2848. doi: 10.3390/cancers14122848 (PMC9221274; doi:10.3390/cancers14122848)
Supplement: Supplementary file 1 [file cancers-14-02848-s001.zip › Table S4.pdf]

Table S4: GSEA analysis of signature 2 genes showing enriched Hallmark and gene ontology gene sets.

ICI Regulation Group	Term	SIZE	NES	NOM p-val
Down Regulated Genes	HALLMARK_HYPOXIA	4	-1.80883	0
Down Regulated Genes	HALLMARK_KRAS_SIGNALING_UP	4	-1.64874	0.028169014
Down Regulated Genes	HALLMARK_MYOGENESIS	9	-1.49083	0.111363634
Down Regulated Genes	HALLMARK_P53_PATHWAY	3	-1.32093	0.14256619
Down Regulated Genes	HALLMARK_APICAL_SURFACE	1	-1.3036	0
Down Regulated Genes	HALLMARK_WNT_BETA_CATENIN_SIGNALING	1	-1.19884	0.18867925
Down Regulated Genes	HALLMARK_PI3K_AKT_MTOR_SIGNALING	4	-1.13364	0.30426356
Down Regulated Genes	HALLMARK_UV_RESPONSE_UP	7	-1.02959	0.4054054
Down Regulated Genes	HALLMARK_MTORC1_SIGNALING	5	-0.92772	0.53592235
Down Regulated Genes	HALLMARK_FATTY_ACID_METABOLISM	2	-0.89462	0.6113281
Down Regulated Genes	HALLMARK_TNFA_SIGNALING_VIA_NFKB	1	-0.83377	0.73540854
Down Regulated Genes	HALLMARK_ADIPOGENESIS	8	-0.82818	0.6191406
Down Regulated Genes	HALLMARK_GLYCOLYSIS	9	-0.75859	0.7413442
Down Regulated Genes	HALLMARK_TGF_BETA_SIGNALING	1	-0.71673	0.9107505
Down Regulated Genes	HALLMARK_ANDROGEN_RESPONSE	1	-0.69432	0.9570094
Down Regulated Genes	HALLMARK_HEDGEHOG_SIGNALING	2	-0.53359	0.9773196
Down Regulated Genes	HALLMARK_HEME_METABOLISM	8	-0.4596	0.9796748
Upregulated Genes	HALLMARK_UNFOLDED_PROTEIN_RESPONSE	4	1.662103	0.024896266
Upregulated Genes	HALLMARK_INFLAMMATORY_RESPONSE	3	1.578235	0.049484536
Upregulated Genes	HALLMARK_APICAL_JUNCTION	6	1.449136	0.14052953
Upregulated Genes	HALLMARK_ESTROGEN_RESPONSE_EARLY	6	1.416446	0.1680498
Upregulated Genes	HALLMARK_INTERFERON_GAMMA_RESPONSE	2	1.365956	0.12396694
Upregulated Genes	HALLMARK_COAGULATION	1	1.21285	0.18217821
Upregulated Genes	HALLMARK_KRAS_SIGNALING_DN	2	1.174649	0.29713115
Upregulated Genes	HALLMARK_PEROXISOME	2	1.153437	0.30241936
Upregulated Genes	HALLMARK_IL6_JAK_STAT3_SIGNALING	1	1.139411	0.278
Upregulated Genes	HALLMARK_CHOLESTEROL_HOMEOSTASIS	2	1.097562	0.37708333
Upregulated Genes	HALLMARK_INTERFERON_ALPHA_RESPONSE	1	1.092279	0.35456476
Upregulated Genes	HALLMARK_ESTROGEN_RESPONSE_LATE	5	1.004997	0.45306122
Upregulated Genes	HALLMARK_UV_RESPONSE_DN	1	0.922944	0.6216216
Upregulated Genes	HALLMARK_EPITHELIAL_MESENCHYMAL_TRANSITION	6	0.917823	0.5106383
Upregulated Genes	HALLMARK_NOTCH_SIGNALING	1	0.913919	0.6199187
Upregulated Genes	HALLMARK_DNA_REPAIR	4	0.904135	0.56092435
Upregulated Genes	HALLMARK_IL2_STAT5_SIGNALING	3	0.89253	0.588694

Upregulated Genes	HALLMARK_MYC_TARGETS_V1	4	0.868221	0.5620915
Upregulated Genes	HALLMARK_BILE_ACID_METABOLISM	1	0.847604	0.71456313
Upregulated Genes	HALLMARK_XENOBIOTIC_METABOLISM	1	0.766526	0.8170974
Upregulated Genes	HALLMARK_OXIDATIVE_PHOSPHORYLATION	3	0.741629	0.7533207
Upregulated Genes	HALLMARK_APOPTOSIS	4	0.673601	0.8019802
Upregulated Genes	HALLMARK_COMPLEMENT	2	0.62219	0.92948717
Upregulated Genes	HALLMARK_MITOTIC_SPINDLE	3	0.563489	0.93603414
Upregulated Genes	HALLMARK_PROTEIN_SECRETION	2	0.537482	0.9744597
Down Regulated Genes	GO_REGULATION_OF_APOPTOTIC_SIGNALING_PATHWAY	10	-1.95516	0.002074689
Down Regulated Genes	GO_APOPTOTIC_SIGNALING_PATHWAY	16	-1.85379	0.011441648
Down Regulated Genes	GO_DETECTION_OF_STIMULUS	5	-1.84642	0.008298756
Down Regulated Genes	GO_VESICLE_ORGANIZATION	7	-1.8356	0.002079002
Down Regulated Genes	GO_ORGANELLE_ASSEMBLY	10	-1.81117	0.016494846
Down Regulated Genes	GO_RESPONSE_TO_ENDOPLASMIC_RETICULUM_STRESS	8	-1.79986	0.020661157
Down Regulated Genes	GO_EXTRINSIC_APOPTOTIC_SIGNALING_PATHWAY	5	-1.76444	0.012072435
Down Regulated Genes	GO_POSITIVE_REGULATION_OF_APOPTOTIC_SIGNALING_PATHWAY	4	-1.75385	0.013916501
Down Regulated Genes	GO_REGULATION_OF_CELL_POPULATION_PROLIFERATION	26	-1.73179	0.028708134
Down Regulated Genes	GO_REGULATION_OF_EXTRINSIC_APOPTOTIC_SIGNALING_PATHWAY	4	-1.72937	0.018595042
Down Regulated Genes	GO_PROCESS_UTILIZING_AUTOPHAGIC_MECHANISM	11	-1.70735	0.03125
Down Regulated Genes	GO_REGULATION_OF_CELL_DEATH	32	-1.70266	0.017156864
Down Regulated Genes	GO_LYTIC_VACUOLE_ORGANIZATION	3	-1.69357	0.00996016
Down Regulated Genes	GO_PHAGOSOME_MATURATION	3	-1.69205	0.017681729
Down Regulated Genes	GO_POSITIVE_REGULATION_OF_EXTRINSIC_APOPTOTIC_SIGNALING_PATH\	3	-1.69139	0.00744879
Down Regulated Genes	GO_POSITIVE_REGULATION_OF_SIGNALING	28	-1.67972	0.033573143
Down Regulated Genes	GO_VACUOLE_ORGANIZATION	5	-1.67049	0.03137255
Down Regulated Genes	GO_MOLTING_CYCLE	4	-1.65991	0.024208566
Down Regulated Genes	GO_NEGATIVE_REGULATION_OF_CELL_POPULATION_PROLIFERATION	10	-1.6575	0.04
Down Regulated Genes	GO_POSITIVE_REGULATION_OF_CELLULAR_COMPONENT_MOVEMENT	14	-1.65675	0.04793028
Down Regulated Genes	GO_SKIN_DEVELOPMENT	7	-1.62921	0.059813086
Down Regulated Genes	GO_PHAGOLYSOSOME_ASSEMBLY	2	-1.60124	0.004048583
Down Regulated Genes	GO_MUSCLE_CONTRACTION	8	-1.59942	0.060240965
Down Regulated Genes	GO_EPITHELIAL_CELL_DIFFERENTIATION	16	-1.59039	0.062893085
Down Regulated Genes	GO_REGULATION_OF_CALCIUM_ION_TRANSPORT	3	-1.58739	0.028735632
Down Regulated Genes	GO_POSITIVE_REGULATION_OF_WOUND_HEALING	3	-1.58713	0.034979425
Down Regulated Genes	GO_POSITIVE_REGULATION_OF_EXTRINSIC_APOPTOTIC_SIGNALING_PATH\	2	-1.58698	0.009881423
Down Regulated Genes	GO_PHAGOCYTOSIS	10	-1.5692	0.07515658

Down Regulated Genes	GO_SIGNAL_TRANSDUCTION_IN_ABSENCE_OF_LIGAND	2	-1.56468	0.015841585
Down Regulated Genes	GO_REGULATION_OF_EXTRINSIC_APOPTOTIC_SIGNALING_PATHWAY_IN_A	2	-1.5646	0.016736401
Down Regulated Genes	GO_TRNA_MODIFICATION	4	-1.55484	0.046728972
Down Regulated Genes	GO_DETECTION_OF_STIMULUS_INVOLVED_IN_SENSORY_PERCEPTION_OF_	2	-1.55451	0.020876827
Down Regulated Genes	GO_TRNA_PROCESSING	6	-1.54885	0.05742574
Down Regulated Genes	GO_SENSORY_PERCEPTION_OF_TEMPERATURE_STIMULUS	2	-1.54149	0.027829314
Down Regulated Genes	GO_APOPTOTIC_PROCESS	40	-1.53957	0.058171745
Down Regulated Genes	GO_DETECTION_OF_TEMPERATURE_STIMULUS_INVOLVED_IN_SENSORY_P	2	-1.5324	0.024193548
Down Regulated Genes	GO_ESTABLISHMENT_OF_PIGMENT_GRANULE_LOCALIZATION	4	-1.53154	0.0625
Down Regulated Genes	GO_CYCLIC_NUCLEOTIDE_MEDIATED_SIGNALING	3	-1.52896	0.058467742
Down Regulated Genes	GO_TRNA_METABOLIC_PROCESS	9	-1.52593	0.085365854
Down Regulated Genes	GO_PIGMENT_GRANULE_LOCALIZATION	4	-1.52271	0.057539683
Down Regulated Genes	GO_DETECTION_OF_TEMPERATURE_STIMULUS	2	-1.51885	0.027722772
Down Regulated Genes	GO_KERATINOCYTE_DIFFERENTIATION	6	-1.51532	0.072265625
Down Regulated Genes	GO_CELLULAR_PIGMENTATION	4	-1.5122	0.06349207
Down Regulated Genes	GO_PIGMENTATION	5	-1.49892	0.08467742
Down Regulated Genes	GO_POSITIVE_REGULATION_OF_INTRACELLULAR_SIGNAL_TRANSDUCTION	14	-1.49299	0.07002188
Down Regulated Genes	GO_SKIN_EPIDERMIS_DEVELOPMENT	3	-1.49215	0.06692913
Down Regulated Genes	GO_SENSORY_PERCEPTION_OF_PAIN	2	-1.48815	0.028806584
Down Regulated Genes	GO_EPIDERMAL_CELL_DIFFERENTIATION	6	-1.48692	0.11787819
Down Regulated Genes	GO_MUSCLE_SYSTEM_PROCESS	9	-1.48301	0.12
Down Regulated Genes	GO_REGULATION_OF_TRANSFERASE_ACTIVITY	11	-1.47817	0.10559006
Down Regulated Genes	GO_MAINTENANCE_OF_CELL_NUMBER	3	-1.47467	0.07170542
Down Regulated Genes	GO_POSITIVE_REGULATION_OF_RESPONSE_TO_WOUNDING	4	-1.4739	0.07442748
Down Regulated Genes	GO_DEVELOPMENTAL_PIGMENTATION	3	-1.47232	0.090405904
Down Regulated Genes	GO_HEAD_DEVELOPMENT	14	-1.46605	0.112474434
Down Regulated Genes	GO_POSITIVE_REGULATION_OF_MAPK_CASCADE	6	-1.46344	0.09504951
Down Regulated Genes	GO_POSITIVE_REGULATION_OF_CELL_DEATH	17	-1.4606	0.08993576
Down Regulated Genes	GO_PATTERN_SPECIFICATION_PROCESS	9	-1.44849	0.11273486
Down Regulated Genes	GO_DIVALENT_INORGANIC_CATION_TRANSPORT	9	-1.44777	0.13877551
Down Regulated Genes	GO_NUCLEOSOME_ORGANIZATION	2	-1.43973	0.064
Down Regulated Genes	GO_HISTONE_EXCHANGE	2	-1.43926	0.059880238
Down Regulated Genes	GO_ANATOMICAL_STRUCTURE_FORMATION_INVOLVED_IN_MORPHOGENE	29	-1.4385	0.086538464
Down Regulated Genes	GO_PIGMENT_CELL_DIFFERENTIATION	3	-1.43708	0.07782101
Down Regulated Genes	GO_ATP_DEPENDENT_CHROMATIN_REMODELING	2	-1.43697	0.058467742
Down Regulated Genes	GO_REGULATION_OF_METAL_ION_TRANSPORT	4	-1.43088	0.115079366

Down Regulated Genes	GO_LEUKOCYTE_CHEMOTAXIS	4	-1.42735	0.103515625
Down Regulated Genes	GO_SECOND_MESSENGER_MEDIATED_SIGNALING	7	-1.42407	0.12863071
Down Regulated Genes	GO_RESPONSE_TO_ACTIVITY	2	-1.3983	0.07647059
Down Regulated Genes	GO_KERATINIZATION	4	-1.39078	0.11890838
Down Regulated Genes	GO_MUSCLE_ORGAN_DEVELOPMENT	11	-1.38819	0.13616072
Down Regulated Genes	GO_ENDOTHELIUM_DEVELOPMENT	3	-1.3845	0.10097087
Down Regulated Genes	GO_CAMERA_TYPE_EYE_DEVELOPMENT	8	-1.38253	0.15019763
Down Regulated Genes	GO_RETINA_DEVELOPMENT_IN_CAMERA_TYPE_EYE	6	-1.38053	0.14862385
Down Regulated Genes	GO_TUBE_MORPHOGENESIS	20	-1.37765	0.1554054
Down Regulated Genes	GO_POSITIVE_REGULATION_OF_CALCIUM_ION_TRANSPORT	2	-1.37636	0.107407406
Down Regulated Genes	GO_POSITIVE_REGULATION_OF_PHOSPHATIDYLINOSITOL_3_KINASE_SIGNA	2	-1.37554	0.09657948
Down Regulated Genes	GO_RESPONSE_TO_ALKALOID	2	-1.37525	0.08193669
Down Regulated Genes	GO_INFLAMMATORY_RESPONSE	8	-1.37135	0.15490197
Down Regulated Genes	GO_TELENCEPHALON_DEVELOPMENT	7	-1.37103	0.13385826
Down Regulated Genes	GO_PROTEIN_LOCALIZATION_TO_NUCLEUS	3	-1.37086	0.1332008
Down Regulated Genes	GO_CORNIFICATION	4	-1.3707	0.13502936
Down Regulated Genes	GO_POSITIVE_REGULATION_OF_CHEMOTAXIS	5	-1.36235	0.1527495
Down Regulated Genes	GO_CELLULAR_COMPONENT_MAINTENANCE	2	-1.36198	0.103174604
Down Regulated Genes	GO_ESTABLISHMENT_OF_PROTEIN_LOCALIZATION_TO_PLASMA_MEMBRAI	2	-1.36106	0.10331384
Down Regulated Genes	GO_RESPONSE_TO_BIOTIC_STIMULUS	15	-1.36002	0.16595745
Down Regulated Genes	GO_DETECTION_OF_CELL_DENSITY	1	-1.35826	0
Down Regulated Genes	GO_REGULATION_OF_MAPK_CASCADE	7	-1.35577	0.156
Down Regulated Genes	GO_ERK1_AND_ERK2_CASCADE	2	-1.35267	0.124756336
Down Regulated Genes	GO_REGULATION_OF_INTRINSIC_APOPTOTIC_SIGNALING_PATHWAY_BY_P.	2	-1.35204	0.10131332
Down Regulated Genes	GO_EPITHELIUM_DEVELOPMENT	31	-1.34348	0.13681592
Down Regulated Genes	GO_EPITHELIAL_TUBE_FORMATION	4	-1.34019	0.15415822
Down Regulated Genes	GO_DETECTION_OF_STIMULUS_INVOLVED_IN_SENSORY_PERCEPTION	3	-1.33843	0.13972056
Down Regulated Genes	GO_PROTEIN_LOCALIZATION_TO_NON_MOTILE_CILIUM	1	-1.33765	0.034274194
Down Regulated Genes	GO_RESPONSE_TO_TEMPERATURE_STIMULUS	4	-1.33691	0.17153996
Down Regulated Genes	GO_TEMPERATURE_HOMEOSTASIS	2	-1.33629	0.110687025
Down Regulated Genes	GO_TOLL_LIKE_RECEPTOR_2_SIGNALING_PATHWAY	1	-1.33549	0.01980198
Down Regulated Genes	GO_DETECTION_OF_BIOTIC_STIMULUS	1	-1.33544	0
Down Regulated Genes	GO_ECTODERM_DEVELOPMENT	1	-1.33534	0.022916667
Down Regulated Genes	GO_ADENYLATE_CYCLASE_ACTIVATING_G_PROTEIN_COUPLED_RECEPTOR_	2	-1.33434	0.123791106
Down Regulated Genes	GO_TOLL_LIKE_RECEPTOR_7_SIGNALING_PATHWAY	1	-1.33362	0.014767933
Down Regulated Genes	GO_CELL_FATE_COMMITMENT_INVOLVED_IN_FORMATION_OF_PRIMARY_	1	-1.33344	0.010162601

Down Regulated Genes	GO_SENSORY_SYSTEM_DEVELOPMENT	8	-1.33273	0.1627409
Down Regulated Genes	GO_MEMBRANE_FUSION	2	-1.33176	0.12092131
Down Regulated Genes	GO_NEURAL_TUBE_DEVELOPMENT	2	-1.33028	0.119284295
Down Regulated Genes	GO_MAMMARY_GLAND_EPITHELIAL_CELL_DIFFERENTIATION	1	-1.32913	0.014492754
Down Regulated Genes	GO_MYOBLAST_PROLIFERATION	2	-1.32865	0.11819887
Down Regulated Genes	GO_REGULATION_OF_PROTEIN_KINASE_C_SIGNALING	1	-1.32861	0.029411765
Down Regulated Genes	GO_POSITIVE_REGULATION_OF_TRANSFERASE_ACTIVITY	8	-1.32847	0.1754386
Down Regulated Genes	GO_ESTABLISHMENT_OF_ORGANELLE_LOCALIZATION	8	-1.32783	0.17561984
Down Regulated Genes	GO_MAMMARY_GLAND_DEVELOPMENT	3	-1.32594	0.16862746
Down Regulated Genes	GO_SOMATIC_STEM_CELL_POPULATION_MAINTENANCE	1	-1.3248	0.012244898
Down Regulated Genes	GO_NEURAL_TUBE_FORMATION	2	-1.32474	0.13076924
Down Regulated Genes	GO_ENSHEATHMENT_OF_NEURONS	3	-1.3245	0.15037593
Down Regulated Genes	GO_VASCULATURE_DEVELOPMENT	17	-1.32309	0.16666667
Down Regulated Genes	GO_SYNAPTIC_VESICLE_TRANSPORT	2	-1.32262	0.14202334
Down Regulated Genes	GO_REGULATION_OF_VASCULAR_WOUND_HEALING	1	-1.3223	0.031809144
Down Regulated Genes	GO_ECTODERMAL_CELL_DIFFERENTIATION	1	-1.32168	0.027196653
Down Regulated Genes	GO_LOCALIZATION_WITHIN_MEMBRANE	2	-1.32162	0.14437367
Down Regulated Genes	GO_NEURON_RECOGNITION	7	-1.32128	0.16666667
Down Regulated Genes	GO_MAMMARY_GLAND_EPITHELIUM_DEVELOPMENT	1	-1.32111	0.011764706
Down Regulated Genes	GO_MULTI_ORGANISM_MEMBRANE_ORGANIZATION	1	-1.32036	0.029821074
Down Regulated Genes	GO_POSITIVE_REGULATION_OF_COLD_INDUCED_THERMOGENESIS	2	-1.3194	0.15243903
Down Regulated Genes	GO_G_PROTEIN_COUPLED_RECEPTOR_SIGNALING_PATHWAY_COUPLED_T	2	-1.31909	0.1194332
Down Regulated Genes	GO_SENSORY_PERCEPTION	11	-1.31883	0.17570499
Down Regulated Genes	GO_TELENCEPHALON_REGIONALIZATION	1	-1.31486	0.058939096
Down Regulated Genes	GO_ADAPTIVE_THERMOGENESIS	2	-1.31467	0.12548262
Down Regulated Genes	GO_TOLL_LIKE_RECEPTOR_SIGNALING_PATHWAY	3	-1.31315	0.15506959
Down Regulated Genes	GO_DETECTION_OF_MECHANICAL_STIMULUS_INVOLVED_IN_SENSORY_PEI	1	-1.31273	0.036659878
Down Regulated Genes	GO_REGULATION_OF_EPITHELIAL_CELL_MIGRATION	1	-1.31269	0.036659878
Down Regulated Genes	GO_BLOOD_VESSEL_REMODELING	1	-1.3121	0.044871796
Down Regulated Genes	GO_VASCULAR_WOUND_HEALING	1	-1.31174	0.0546875
Down Regulated Genes	GO_REGULATION_OF_CHEMOTAXIS	6	-1.3103	0.20724346
Down Regulated Genes	GO_TUBE_FORMATION	4	-1.30971	0.17165668
Down Regulated Genes	GO_CARDIAC_MUSCLE_CONTRACTION	2	-1.30935	0.14071295
Down Regulated Genes	GO_NEGATIVE_REGULATION_OF_MYOBLAST_DIFFERENTIATION	1	-1.30829	0.073619634
Down Regulated Genes	GO_LUNG_ALVEOLUS_DEVELOPMENT	1	-1.30828	0.050607286
Down Regulated Genes	GO_POSITIVE_REGULATION_OF_PROTEIN_KINASE_C_SIGNALING	1	-1.308	0.048169557

Down Regulated Genes	GO_DENDRITE_EXTENSION	1	-1.30713	0.037593983
Down Regulated Genes	GO_POSITIVE_REGULATION_OF_ERK1_AND_ERK2_CASCADE	1	-1.30694	0.052930057
Down Regulated Genes	GO_FAT_CELL_DIFFERENTIATION	8	-1.3069	0.2025862
Down Regulated Genes	GO_VESICLE_TRANSPORT_ALONG_MICROTUBULE	1	-1.30613	0.06431536
Down Regulated Genes	GO_REGULATION_OF_DENDRITE_EXTENSION	1	-1.30608	0.051136363
Down Regulated Genes	GO_MYELIN_MAINTENANCE	1	-1.30581	0.04518664
Down Regulated Genes	GO_MORPHOGENESIS_OF_AN_ENDOTHELIUM	1	-1.30425	0.025742574
Down Regulated Genes	GO_POSITIVE_REGULATION_OF_ENDOTHELIAL_CELL_MIGRATION	1	-1.30395	0.042857144
Down Regulated Genes	GO_POSITIVE_REGULATION_OF_DEVELOPMENTAL_PIGMENTATION	1	-1.30363	0.06517312
Down Regulated Genes	GO_SYNAPTIC_VESICLE_CYTOSKELETAL_TRANSPORT	1	-1.30289	0.059642147
Down Regulated Genes	GO_REGULATION_OF_DEVELOPMENTAL_PIGMENTATION	1	-1.30279	0.061143983
Down Regulated Genes	GO_CELLULAR_RESPONSE_TO_VASCULAR_ENDOTHELIAL_GROWTH_FACTO	1	-1.30248	0.058467742
Down Regulated Genes	GO_TOLL_LIKE_RECEPTOR_4_SIGNALING_PATHWAY	1	-1.30227	0.016194332
Down Regulated Genes	GO_VASCULAR_ENDOTHELIAL_GROWTH_FACTOR_SIGNALING_PATHWAY	1	-1.30053	0.064
Down Regulated Genes	GO_SPROUTING_ANGIOGENESIS	1	-1.30043	0.03883495
Down Regulated Genes	GO_RESPONSE_TO_OSMOTIC_STRESS	1	-1.29985	0.056751467
Down Regulated Genes	GO_GLUTATHIONE_CATABOLIC_PROCESS	1	-1.29966	0.02952756
Down Regulated Genes	GO_PROTEIN_TRANSPORT_WITHIN_LIPID_BILAYER	1	-1.29963	0.035928145
Down Regulated Genes	GO_REGULATION_OF_ENDOTHELIAL_CELL_MIGRATION	1	-1.29903	0.06198347
Down Regulated Genes	GO_POSITIVE_REGULATION_OF_VASCULAR_WOUND_HEALING	1	-1.29893	0.048780486
Down Regulated Genes	GO_HYPOTONIC_RESPONSE	1	-1.29889	0.06805293
Down Regulated Genes	GO_RESPONSE_TO_CHEMOKINE	1	-1.29812	0.04296875
Down Regulated Genes	GO_MESODERM_DEVELOPMENT	1	-1.29738	0.042596348
Down Regulated Genes	GO_TOLL_LIKE_RECEPTOR_9_SIGNALING_PATHWAY	1	-1.29648	0.028513238
Down Regulated Genes	GO_NEGATIVE_REGULATION_OF_BIOMINERALIZATION	2	-1.29638	0.16763006
Down Regulated Genes	GO_TUBE_DEVELOPMENT	25	-1.29605	0.1977528
Down Regulated Genes	GO_RESPONSE_TO_ISOQUINOLINE_ALKALOID	1	-1.29595	0.04660194
Down Regulated Genes	GO_PROTEIN_LOCALIZATION_TO_CILIUM	1	-1.29592	0.03448276
Down Regulated Genes	GO_POSITIVE_REGULATION_OF_EPITHELIAL_CELL_MIGRATION	1	-1.29569	0.049484536
Down Regulated Genes	GO_POSITIVE_REGULATION_OF_VASCULAR_ENDOTHELIAL_GROWTH_FACT	1	-1.29498	0.03586498
Down Regulated Genes	GO_AXONAL_TRANSPORT	1	-1.29485	0.06042885
Down Regulated Genes	GO_DORSAL_VENTRAL_PATTERN_FORMATION	1	-1.29424	0.05697446
Down Regulated Genes	GO_OLFACTORY_LOBE_DEVELOPMENT	1	-1.29402	0.051587302
Down Regulated Genes	GO_PIGMENT_ACCUMULATION	1	-1.29385	0.07862903
Down Regulated Genes	GO_PROTEIN_KINASE_C_SIGNALING	1	-1.29377	0.05210421
Down Regulated Genes	GO_MELANOCYTE_DIFFERENTIATION	2	-1.2935	0.15800416

Down Regulated Genes	GO_AXO_DENDRITIC_TRANSPORT	1	-1.2928	0.05263158
Down Regulated Genes	GO_ANGIOGENESIS_INVOLVED_IN_WOUND_HEALING	1	-1.29272	0.04761905
Down Regulated Genes	GO_NEGATIVE_REGULATION_OF_TRANSCRIPTION_REGULATORY_REGION_I	1	-1.29157	0.04918033
Down Regulated Genes	GO_MAINTENANCE_OF_APICAL_BASAL_CELL_POLARITY	1	-1.29157	0.05210421
Down Regulated Genes	GO_VASCULAR_ENDOTHELIAL_GROWTH_FACTOR_PRODUCTION	1	-1.2914	0.03359684
Down Regulated Genes	GO_MULTI_ORGANISM_MEMBRANE_FUSION	1	-1.29096	0.047244094
Down Regulated Genes	GO_MUSCLE_ORGAN_MORPHOGENESIS	1	-1.29089	0.076045625
Down Regulated Genes	GO_DETECTION_OF_ABIOTIC_STIMULUS	3	-1.29079	0.16735537
Down Regulated Genes	GO_GLUTATHIONE_METABOLIC_PROCESS	1	-1.29062	0.049484536
Down Regulated Genes	GO_MAINTENANCE_OF_CELL_POLARITY	1	-1.29007	0.064257026
Down Regulated Genes	GO_NEGATIVE_REGULATION_OF_STRIATED_MUSCLE_CELL_DIFFERENTIATIO	1	-1.29	0.06613226
Down Regulated Genes	GO_REGULATION_OF_EOSINOPHIL_MIGRATION	1	-1.28806	0.088477366
Down Regulated Genes	GO_ANTEROGRADE_AXONAL_TRANSPORT	1	-1.28671	0.058091287
Down Regulated Genes	GO_CELLULAR_RESPONSE_TO_OSMOTIC_STRESS	1	-1.28637	0.08510638
Down Regulated Genes	GO_REGULATION_OF_INTRACELLULAR_SIGNAL_TRANSDUCTION	31	-1.28598	0.1970803
Down Regulated Genes	GO_EPITHELIAL_TUBE_MORPHOGENESIS	11	-1.28537	0.21212122
Down Regulated Genes	GO_FOREBRAIN_REGIONALIZATION	1	-1.28229	0.07692308
Down Regulated Genes	GO_PIGMENT_GRANULE_ORGANIZATION	1	-1.28203	0.085185185
Down Regulated Genes	GO_REGULATION_OF_STRIATED_MUSCLE_CELL_DIFFERENTIATION	1	-1.28166	0.068359375
Down Regulated Genes	GO_POSITIVE_REGULATION_OF_RESPONSE_TO_CYTOKINE_STIMULUS	2	-1.27989	0.15087041
Down Regulated Genes	GO_CELL_CHEMOTAXIS	6	-1.27987	0.21212122
Down Regulated Genes	GO_CELLULAR_MODIFIED_AMINO_ACID_CATABOLIC_PROCESS	1	-1.27972	0.065868266
Down Regulated Genes	GO_NONRIBOSOMAL_PEPTIDE_BIOSYNTHETIC_PROCESS	1	-1.27972	0.062248997
Down Regulated Genes	GO_REGULATION_OF_PIGMENT_CELL_DIFFERENTIATION	1	-1.27962	0.08317215
Down Regulated Genes	GO_PIGMENT_GRANULE_MATURATION	1	-1.27954	0.09883721
Down Regulated Genes	GO_CELLULAR_HYPOTONIC_RESPONSE	1	-1.27931	0.06990291
Down Regulated Genes	GO_PEPTIDYL_CYSTEINE_MODIFICATION	2	-1.27878	0.18023255
Down Regulated Genes	GO_CELLULAR_RESPONSE_TO_ABIOTIC_STIMULUS	1	-1.27818	0.06412826
Down Regulated Genes	GO_CELLULAR_MODIFIED_AMINO_ACID_BIOSYNTHETIC_PROCESS	1	-1.27789	0.06854839
Down Regulated Genes	GO_POSITIVE_REGULATION_OF_RESPONSE_TO_EXTERNAL_STIMULUS	6	-1.27704	0.21073559
Down Regulated Genes	GO_REGULATION_OF_TRANSCRIPTION_FROM_RNA_POLYMERASE_II_PROM	1	-1.27614	0.08661418
Down Regulated Genes	GO_PATTERN_RECOGNITION_RECEPTOR_SIGNALING_PATHWAY	3	-1.27556	0.18458419
Down Regulated Genes	GO_PEPTIDE_CATABOLIC_PROCESS	1	-1.27484	0.057692308
Down Regulated Genes	GO_REGULATION_OF_BLOOD_VESSEL_REMODELING	1	-1.27483	0.05785124
Down Regulated Genes	GO_REGULATION_OF_MYOBLAST_DIFFERENTIATION	1	-1.2731	0.07936508
Down Regulated Genes	GO_MYOTUBE_DIFFERENTIATION	3	-1.27179	0.19962335

Down Regulated Genes	GO_ANION_HOMEOSTASIS	2	-1.27163	0.1733871
Down Regulated Genes	GO_OLIGOSACCHARIDE_LIPID_INTERMEDIATE_BIOSYNTHETIC_PROCESS	1	-1.27157	0.11561866
Down Regulated Genes	GO_NEGATIVE_REGULATION_OF_MYOTUBE_DIFFERENTIATION	1	-1.27132	0.08952381
Down Regulated Genes	GO_TONIC_SMOOTH_MUSCLE_CONTRACTION	1	-1.27116	0.090361446
Down Regulated Genes	GO_BLOOD_VESSEL_MORPHOGENESIS	15	-1.27113	0.22222222
Down Regulated Genes	GO_ACTIVATION_OF_MAPK_ACTIVITY	2	-1.27112	0.19193858
Down Regulated Genes	GO_NEGATIVE_REGULATION_OF_BONE_MINERALIZATION	2	-1.27063	0.18913858
Down Regulated Genes	GO_RIBOSE_PHOSPHATE_METABOLIC_PROCESS	10	-1.27033	0.22222222
Down Regulated Genes	GO_STRIATED_MUSCLE_CONTRACTION	3	-1.26877	0.19324578
Down Regulated Genes	GO_VESICLE_CYTOSKELETAL_TRAFFICKING	1	-1.26811	0.07966457
Down Regulated Genes	GO_REGULATION_OF_MYOTUBE_DIFFERENTIATION	1	-1.26688	0.08383234
Down Regulated Genes	GO_SKELETAL_MUSCLE_CELL_DIFFERENTIATION	3	-1.26682	0.2102161
Down Regulated Genes	GO_CILIUM_ORGANIZATION	5	-1.26537	0.22485207
Down Regulated Genes	GO_REGULATION_OF_GLIAL_CELL_DIFFERENTIATION	2	-1.26527	0.19653179
Down Regulated Genes	GO_ANOIKIS	1	-1.26406	0.10566038
Down Regulated Genes	GO_NEGATIVE_REGULATION_OF_MUSCLE_CELL_DIFFERENTIATION	1	-1.26404	0.076771654
Down Regulated Genes	GO_LEUKOCYTE_MEDIATED_CYTOTOXICITY	2	-1.26368	0.18546845
Down Regulated Genes	GO_EOSINOPHIL_CHEMOTAXIS	1	-1.26265	0.104417674
Down Regulated Genes	GO_AORTA_SMOOTH_MUSCLE_TISSUE_MORPHOGENESIS	1	-1.26131	0.07581967
Down Regulated Genes	GO_EOSINOPHIL_MIGRATION	1	-1.26118	0.10224949
Down Regulated Genes	GO_REGULATION_OF_INTRINSIC_APOPTOTIC_SIGNALING_PATHWAY	6	-1.25671	0.21789883
Down Regulated Genes	GO_ACTIVATION_OF_PROTEIN_KINASE_ACTIVITY	2	-1.25599	0.19038461
Down Regulated Genes	GO_SMOOTH_MUSCLE_TISSUE_DEVELOPMENT	1	-1.25577	0.069327734
Down Regulated Genes	GO_POSITIVE_REGULATION_OF_GLIOGENESIS	2	-1.25574	0.1953125
Down Regulated Genes	GO_NUCLEOBASE_CONTAINING_SMALL_MOLECULE_METABOLIC_PROCESS	11	-1.2554	0.22540984
Down Regulated Genes	GO_PEPTIDYL_L_CYSTEINE_S_PALMITOYLATION	2	-1.2553	0.20123203
Down Regulated Genes	GO_NUCLEOSIDE_PHOSPHATE_CATABOLIC_PROCESS	2	-1.25487	0.20990099
Down Regulated Genes	GO_REGULATION_OF_OLIGODENDROCYTE_DIFFERENTIATION	2	-1.25249	0.2195122
Down Regulated Genes	GO_EPIDERMIS_DEVELOPMENT	10	-1.25236	0.22792608
Down Regulated Genes	GO_REGULATION_OF_PHOSPHATIDYLINOSITOL_3_KINASE_SIGNALING	3	-1.25138	0.21317829
Down Regulated Genes	GO_PHOSPHATIDYLINOSITOL_3_KINASE_SIGNALING	3	-1.25127	0.20871143
Down Regulated Genes	GO_PROTEIN_TETRAMERIZATION	3	-1.24973	0.20930232
Down Regulated Genes	GO_CELLULAR_RESPONSE_TO_DRUG	2	-1.24903	0.18773946
Down Regulated Genes	GO_REGULATION_OF_MAP_KINASE_ACTIVITY	3	-1.24825	0.22064777
Down Regulated Genes	GO_PURINE_CONTAINING_COMPOUND_METABOLIC_PROCESS	10	-1.24526	0.23838384
Down Regulated Genes	GO_PROTEIN_COMPLEX_OLIGOMERIZATION	3	-1.24402	0.20883535

Down Regulated Genes	GO_DENDRITIC_CELL_MIGRATION	2	-1.244	0.21084337
Down Regulated Genes	GO_MITOCHONDRIAL_RESPIRATORY_CHAIN_COMPLEX_III_ASSEMBLY	1	-1.24387	0.12314225
Down Regulated Genes	GO_T_CELL_MEDIATED_CYTOTOXICITY	2	-1.24209	0.20309478
Down Regulated Genes	GO_POSITIVE_REGULATION_OF_OLIGODENDROCYTE_DIFFERENTIATION	2	-1.24209	0.24857685
Down Regulated Genes	GO_TRNA_THREONYLCARBAMOYLADENOSINE_METABOLIC_PROCESS	2	-1.2415	0.23577236
Down Regulated Genes	GO_SIGNAL_TRANSDUCTION_BY_PROTEIN_PHOSPHORYLATION	9	-1.24146	0.24123712
Down Regulated Genes	GO_POSITIVE_REGULATION_OF_GLIAL_CELL_DIFFERENTIATION	2	-1.24096	0.23366337
Down Regulated Genes	GO_MULTI_ORGANISM_CELLULAR_PROCESS	2	-1.24081	0.22179732
Down Regulated Genes	GO_POSITIVE_REGULATION_OF_VASCULATURE_DEVELOPMENT	2	-1.24067	0.23076923
Down Regulated Genes	GO_SKELETAL_MUSCLE_ORGAN_DEVELOPMENT	6	-1.23859	0.23060797
Down Regulated Genes	GO_LYMPH_VESSEL_MORPHOGENESIS	2	-1.23775	0.23137255
Down Regulated Genes	GO_LYMPHANGIOGENESIS	2	-1.2376	0.22058824
Down Regulated Genes	GO_TUBULIN_COMPLEX_ASSEMBLY	1	-1.23703	0.14717741
Down Regulated Genes	GO_REGULATION_OF_GLIOGENESIS	2	-1.23636	0.20373832
Down Regulated Genes	GO_SPECIFICATION_OF_SYMMETRY	3	-1.23434	0.23968565
Down Regulated Genes	GO_HIPPO_SIGNALING	2	-1.23373	0.21305183
Down Regulated Genes	GO_CYTOCHROME_COMPLEX_ASSEMBLY	1	-1.23348	0.1444653
Down Regulated Genes	GO_NEGATIVE_REGULATION_OF_PROTEIN_LOCALIZATION_TO_NUCLEUS	1	-1.23343	0.156
Down Regulated Genes	GO_NEURAL_PRECURSOR_CELL_PROLIFERATION	2	-1.23293	0.2238806
Down Regulated Genes	GO_NEGATIVE_REGULATION_OF_DNA_BINDING	2	-1.23279	0.22524272
Down Regulated Genes	GO_NEGATIVE_REGULATION_OF_CELL_DIFFERENTIATION	14	-1.23209	0.24435319
Down Regulated Genes	GO_REGULATION_OF_PROTEIN_AUTOPHOSPHORYLATION	2	-1.23156	0.22403258
Down Regulated Genes	GO_INOSITOL_LIPID_MEDIATED_SIGNALING	3	-1.23146	0.21359223
Down Regulated Genes	GO_NEGATIVE_REGULATION_OF_CHONDROCYTE_DIFFERENTIATION	1	-1.23001	0.15708812
Down Regulated Genes	GO_NEGATIVE_REGULATION_OF_BONE_DEVELOPMENT	1	-1.22984	0.14396887
Down Regulated Genes	GO_NEGATIVE_REGULATION_OF_CARTILAGE_DEVELOPMENT	1	-1.22542	0.18
Down Regulated Genes	GO_SPONTANEOUS_SYNAPTIC_TRANSMISSION	1	-1.2244	0.16472869
Down Regulated Genes	GO_EPITHELIAL_CELL_DEVELOPMENT	5	-1.22345	0.23762377
Down Regulated Genes	GO_REGULATION_OF_BONE_DEVELOPMENT	1	-1.22206	0.16194332
Down Regulated Genes	GO_REGULATION_OF_GLUCOSE_METABOLIC_PROCESS	3	-1.22145	0.23379175
Down Regulated Genes	GO_RIBONUCLEOPROTEIN_COMPLEX_SUBUNIT_ORGANIZATION	5	-1.2195	0.24271844
Down Regulated Genes	GO_NUCLEAR_TRANSCRIBED_MRNA_CATABOLIC_PROCESS_EXONUCLEOLY	1	-1.2191	0.17588933
Down Regulated Genes	GO_INSULIN_SECRETION_INVOLVED_IN_CELLULAR_RESPONSE_TO_GLUCO	2	-1.21833	0.2152381
Down Regulated Genes	GO_REGULATION_OF_ION_TRANSPORT	10	-1.21782	0.25103736
Down Regulated Genes	GO_REGULATION_OF_NEURAL_PRECURSOR_CELL_PROLIFERATION	2	-1.21685	0.23699422
Down Regulated Genes	GO_REGULATION_OF_CELLULAR_COMPONENT_MOVEMENT	22	-1.21676	0.24423963

Down Regulated Genes	GO_REGULATION_OF_CHONDROCYTE_DEVELOPMENT	1	-1.21492	0.17706238
Down Regulated Genes	GO_NEGATIVE_REGULATION_OF_PROTEIN_IMPORT	1	-1.2139	0.17171717
Down Regulated Genes	GO_SENSORY_PERCEPTION_OF_CHEMICAL_STIMULUS	1	-1.21341	0.19262294
Down Regulated Genes	GO_FOREBRAIN_DEVELOPMENT	9	-1.21225	0.24763705
Down Regulated Genes	GO_SPONTANEOUS_NEUROTRANSMITTER_SECRETION	1	-1.21202	0.18650794
Down Regulated Genes	GO_CELL_CELL_ADHESION_MEDIATED_BY_CADHERIN	1	-1.21198	0.1910569
Down Regulated Genes	GO_LYMPH_VESSEL_DEVELOPMENT	2	-1.2117	0.24801587
Down Regulated Genes	GO_NUCLEAR_TRANSCRIBED_MRNA_CATABOLIC_PROCESS_DEADENYLATIC	1	-1.21044	0.19193858
Down Regulated Genes	GO_SENSORY_PERCEPTION_OF_SMELL	1	-1.20948	0.15368421
Down Regulated Genes	GO_REGULATION_OF_RESPONSE_TO_DNA_DAMAGE_STIMULUS	2	-1.20935	0.24665391
Down Regulated Genes	GO_ORGANELLE_DISASSEMBLY	3	-1.20913	0.2368932
Down Regulated Genes	GO_SENSORY_ORGAN_DEVELOPMENT	10	-1.20836	0.2616633
Down Regulated Genes	GO_NEGATIVE_REGULATION_OF_NUCLEOCYTOPLASMIC_TRANSPORT	1	-1.20825	0.17159763
Down Regulated Genes	GO_AGING	5	-1.20814	0.26209676
Down Regulated Genes	GO_REGULATION_OF_BONE_MINERALIZATION_INVOLVED_IN_BONE_MATU	1	-1.2079	0.19507186
Down Regulated Genes	GO_REGULATION_OF_WOUND_HEALING	4	-1.20587	0.23517786
Down Regulated Genes	GO_PROTEIN_KINASE_B_SIGNALING	3	-1.20582	0.270428
Down Regulated Genes	GO_SULFUR_COMPOUND_CATABOLIC_PROCESS	3	-1.20563	0.25367647
Down Regulated Genes	GO_EPITHELIAL_MESENCHYMAL_CELL_SIGNALING	1	-1.20261	0.20404041
Down Regulated Genes	GO_REGULATION_OF_RESPONSE_TO_CYTOKINE_STIMULUS	3	-1.20106	0.2716763
Down Regulated Genes	GO_REGULATION_OF_SIGNAL_TRANSDUCTION_BY_P53_CLASS_MEDIATOR	3	-1.20041	0.274
Down Regulated Genes	GO_DENDRITIC_CELL_CHEMOTAXIS	2	-1.19989	0.26959848
Down Regulated Genes	GO_ENDOTHELIAL_CELL_DEVELOPMENT	2	-1.19979	0.26122448
Down Regulated Genes	GO_REGULATION_OF_PROTEIN_LOCALIZATION_TO_NUCLEUS	2	-1.19976	0.24950495
Down Regulated Genes	GO_REGULATION_OF_PROTEIN_MODIFICATION_BY_SMALL_PROTEIN_CON	3	-1.19907	0.2696177
Down Regulated Genes	GO_REGULATION_OF_PROTEIN_IMPORT	2	-1.19889	0.2372549
Down Regulated Genes	GO_CELL_RECOGNITION	8	-1.19802	0.2672234
Down Regulated Genes	GO_IMPORT_INTO_NUCLEUS	4	-1.19722	0.2578125
Down Regulated Genes	GO_RETINA_LAYER_FORMATION	2	-1.19711	0.24440299
Down Regulated Genes	GO_PROTEIN_SUMOYLATION	2	-1.19562	0.26814517
Down Regulated Genes	GO_NEURONAL_STEM_CELL_POPULATION_MAINTENANCE	1	-1.1953	0.2112676
Down Regulated Genes	GO_REGULATION_OF_PROTEIN_TYROSINE_KINASE_ACTIVITY	2	-1.19408	0.24448898
Down Regulated Genes	GO_RENAL_TUBULE_DEVELOPMENT	1	-1.19259	0.197065
Down Regulated Genes	GO_NEPHRON_TUBULE_FORMATION	1	-1.19246	0.21010101
Down Regulated Genes	GO_POSITIVE_REGULATION_OF_MAP_KINASE_ACTIVITY	3	-1.19213	0.27148438
Down Regulated Genes	GO_POSITIVE_REGULATION_OF_PROTEIN_KINASE_B_SIGNALING	3	-1.19209	0.27865613

Down Regulated Genes	GO_CHROMATIN_REMODELING	3	-1.19078	0.27005872
Down Regulated Genes	GO_NEURAL_RETINA_DEVELOPMENT	2	-1.18997	0.26930693
Down Regulated Genes	GO_BRANCHING_MORPHOGENESIS_OF_AN_EPITHELIAL_TUBE	4	-1.18935	0.25736737
Down Regulated Genes	GO_G_PROTEIN_COUPLED_RECEPTOR_SIGNALING_PATHWAY	8	-1.18694	0.28853756
Down Regulated Genes	GO_NEPHRON_MORPHOGENESIS	1	-1.18693	0.21946564
Down Regulated Genes	GO_CYTOKINE_PRODUCTION	8	-1.1868	0.28183717
Down Regulated Genes	GO_CORNEA_DEVELOPMENT_IN_CAMERA_TYPE_EYE	1	-1.18552	0.22134387
Down Regulated Genes	GO_CELLULAR_RESPONSE_TO_CHEMICAL_STRESS	7	-1.18419	0.25963488
Down Regulated Genes	GO_NEPHRON_EPITHELIUM_DEVELOPMENT	1	-1.1835	0.20238096
Down Regulated Genes	GO_AORTA_DEVELOPMENT	2	-1.18318	0.26336634
Down Regulated Genes	GO_POSITIVE_REGULATION_OF_NEURAL_PRECURSOR_CELL_PROLIFERATIO	2	-1.18102	0.2589792
Down Regulated Genes	GO_MITOCHONDRIAL_RESPIRATORY_CHAIN_COMPLEX_ASSEMBLY	3	-1.18067	0.27220076
Down Regulated Genes	GO_HEMATOPOIETIC_PROGENITOR_CELL_DIFFERENTIATION	2	-1.18066	0.28031808
Down Regulated Genes	GO_MESONEPHRIC_TUBULE_MORPHOGENESIS	1	-1.18058	0.23076923
Down Regulated Genes	GO_FOREBRAIN_CELL_MIGRATION	3	-1.17956	0.2519084
Down Regulated Genes	GO_REGULATION_OF_TISSUE_REMODELING	3	-1.17891	0.294
Down Regulated Genes	GO_NUCLEOSIDE_TRIPHOSPHATE_METABOLIC_PROCESS	5	-1.17877	0.28277153
Down Regulated Genes	GO_DETECTION_OF_MECHANICAL_STIMULUS_INVOLVED_IN_SENSORY_PEI	2	-1.17776	0.25
Down Regulated Genes	GO_SMOOTH_MUSCLE_CONTRACTION	2	-1.17569	0.26746505
Down Regulated Genes	GO_REGULATION_OF_MITOCHONDRIAL_MEMBRANE_POTENTIAL	2	-1.17526	0.26508227
Down Regulated Genes	GO_PLASMA_MEMBRANE_ORGANIZATION	3	-1.1748	0.2988048
Down Regulated Genes	GO_CELL_FATE_COMMITMENT	7	-1.17297	0.28125
Down Regulated Genes	GO_ENDOSOME_ORGANIZATION	3	-1.17124	0.29469547
Down Regulated Genes	GO_REGULATION_OF_SMOOTHENED_SIGNALING_PATHWAY	3	-1.17039	0.29708737
Down Regulated Genes	GO_RETINA_MORPHOGENESIS_IN_CAMERA_TYPE_EYE	2	-1.16997	0.28060263
Down Regulated Genes	GO_REGULATION_OF_NIK_NF_KAPPAB_SIGNALING	1	-1.16599	0.25198412
Down Regulated Genes	GO_MORPHOGENESIS_OF_EMBRYONIC_EPITHELIUM	5	-1.16379	0.2840909
Down Regulated Genes	GO_AORTA_MORPHOGENESIS	2	-1.16214	0.29198474
Down Regulated Genes	GO_REGULATION_OF_HAIR_FOLLICLE_DEVELOPMENT	1	-1.1574	0.2568421
Down Regulated Genes	GO_CALCIUM_MEDIATED_SIGNALING	5	-1.15674	0.30214426
Down Regulated Genes	GO_REGULATION_OF_INFLAMMATORY_RESPONSE	4	-1.15639	0.30374753
Down Regulated Genes	GO_CENTRAL_NERVOUS_SYSTEM_NEURON_AXONOGENESIS	1	-1.15562	0.27559054
Down Regulated Genes	GO_REGULATION_OF_FAT_CELL_DIFFERENTIATION	5	-1.15401	0.2977867
Down Regulated Genes	GO_DIGESTIVE_TRACT_MORPHOGENESIS	1	-1.15245	0.26019418
Down Regulated Genes	GO_PROTEIN_IMPORT	4	-1.1518	0.30769232
Down Regulated Genes	GO_EPIDERMIS_MORPHOGENESIS	1	-1.15111	0.26546907

Down Regulated Genes	GO_DIADENOSINE_POLYPHOSPHATE_CATABOLIC_PROCESS	1	-1.15056	0.2848233
Down Regulated Genes	GO_DETECTION_OF_MECHANICAL_STIMULUS	2	-1.15013	0.29333332
Down Regulated Genes	GO_CENTRAL_NERVOUS_SYSTEM_PROJECTION_NEURON_AXONOGENESIS	1	-1.15003	0.26326963
Down Regulated Genes	GO_INTERMEDIATE_FILAMENT_ORGANIZATION	1	-1.14949	0.27184466
Down Regulated Genes	GO_POSITIVE_REGULATION_OF_HAIR_CYCLE	1	-1.14903	0.2641129
Down Regulated Genes	GO_ESTABLISHMENT_OR_MAINTENANCE_OF_BIPOLAR_CELL_POLARITY	3	-1.14598	0.32677165
Down Regulated Genes	GO_VESICLE_LOCALIZATION	7	-1.14556	0.3065134
Down Regulated Genes	GO_ORGANELLE_TRANSPORT_ALONG_MICROTUBULE	3	-1.14235	0.29158878
Down Regulated Genes	GO_REGULATION_OF_EPIDERMIS_DEVELOPMENT	1	-1.14219	0.3007663
Down Regulated Genes	GO_INTRINSIC_APOPTOTIC_SIGNALING_PATHWAY	8	-1.14171	0.2781186
Down Regulated Genes	GO_THYMUS_DEVELOPMENT	1	-1.14107	0.26923078
Down Regulated Genes	GO_POSITIVE_REGULATION_OF_EPIDERMIS_DEVELOPMENT	1	-1.14099	0.2945591
Down Regulated Genes	GO_CELLULAR_PROTEIN_CONTAINING_COMPLEX_ASSEMBLY	18	-1.14093	0.31154683
Down Regulated Genes	GO_CENTRAL_NERVOUS_SYSTEM_NEURON_DIFFERENTIATION	1	-1.14068	0.284
Down Regulated Genes	GO_REGULATION_OF_HAIR_CYCLE	1	-1.14024	0.28169015
Down Regulated Genes	GO_CIRCULATORY_SYSTEM_DEVELOPMENT	23	-1.13875	0.29357797
Down Regulated Genes	GO_HEART_TRABECULA_FORMATION	1	-1.13807	0.28402367
Down Regulated Genes	GO_REGULATION_OF_DNA_TEMPLATED_TRANSCRIPTION_IN_RESPONSE_T	2	-1.13754	0.34241244
Down Regulated Genes	GO_CENTRAL_NERVOUS_SYSTEM_NEURON_DEVELOPMENT	1	-1.1355	0.28915662
Down Regulated Genes	GO_EYE_MORPHOGENESIS	3	-1.13463	0.33992094
Down Regulated Genes	GO_DIADENOSINE_POLYPHOSPHATE_METABOLIC_PROCESS	1	-1.13379	0.28373015
Down Regulated Genes	GO_REGULATION_OF_EXOCYTOSIS	3	-1.13378	0.32313576
Down Regulated Genes	GO_REGULATION_OF_ENDOPLASMIC_RETICULUM_TUBULAR_NETWORK_O	1	-1.13344	0.2990654
Down Regulated Genes	GO_REGULATION_OF_CELL_DIFFERENTIATION	44	-1.13247	0.3002611
Down Regulated Genes	GO_RIBONUCLEOTIDE_CATABOLIC_PROCESS	1	-1.13226	0.27431905
Down Regulated Genes	GO_INOSITOL_PHOSPHATE_METABOLIC_PROCESS	1	-1.13101	0.3247012
Down Regulated Genes	GO_REGULATION_OF_PROTEIN_KINASE_ACTIVITY	6	-1.131	0.30578512
Down Regulated Genes	GO_ORGANIC_ACID_CATABOLIC_PROCESS	3	-1.13021	0.34285715
Down Regulated Genes	GO_CORPUS_CALLOSUM_DEVELOPMENT	1	-1.12986	0.3067961
Down Regulated Genes	GO_POSITIVE_REGULATION_OF_PROTEIN_KINASE_ACTIVITY	4	-1.12962	0.3210634
Down Regulated Genes	GO_CENTROMERE_COMPLEX_ASSEMBLY	1	-1.12898	0.29746836
Down Regulated Genes	GO_NUCLEOSOME_ASSEMBLY	1	-1.12872	0.30658436
Down Regulated Genes	GO_SCHWANN_CELL_DEVELOPMENT	1	-1.12652	0.30739298
Down Regulated Genes	GO_NUCLEOSIDE_BISPHOSPHATE_METABOLIC_PROCESS	2	-1.12586	0.3301887
Down Regulated Genes	GO_CAMERA_TYPE_EYE_MORPHOGENESIS	3	-1.12547	0.3596154
Down Regulated Genes	GO_SCHWANN_CELL_DIFFERENTIATION	1	-1.12251	0.30722892

Down Regulated Genes	GO_CHROMATIN_REMODELING_AT_CENTROMERE	1	-1.12248	0.31557378
Down Regulated Genes	GO_DNA_CONFORMATION_CHANGE	1	-1.12091	0.32190475
Down Regulated Genes	GO_POSITIVE_REGULATION_OF_FAT_CELL_DIFFERENTIATION	3	-1.12078	0.3539652
Down Regulated Genes	GO_CELLULAR_AMINO_ACID_CATABOLIC_PROCESS	3	-1.12025	0.34082398
Down Regulated Genes	GO_TRABECULA_MORPHOGENESIS	1	-1.11987	0.32222223
Down Regulated Genes	GO_HEART_TRABECULA_MORPHOGENESIS	1	-1.11953	0.32186234
Down Regulated Genes	GO_REGULATION_OF_PHOSPHORYLATION	20	-1.1195	0.34441805
Down Regulated Genes	GO_TRABECULA_FORMATION	1	-1.11833	0.3407258
Down Regulated Genes	GO_DIPHOSPHOINOSITOL_POLYPHOSPHATE_METABOLIC_PROCESS	1	-1.11707	0.3203125
Down Regulated Genes	GO_POSITIVE_REGULATION_OF_ENDOTHELIAL_CELL_PROLIFERATION	3	-1.11693	0.3385214
Down Regulated Genes	GO_DNA_PACKAGING	1	-1.11661	0.3275862
Down Regulated Genes	GO_POSITIVE_REGULATION_OF_PROTEIN_SERINE_THREONINE_KINASE_AC	4	-1.115	0.36127743
Down Regulated Genes	GO_REGULATION_OF_PROTEIN_SERINE_THREONINE_KINASE_ACTIVITY	4	-1.11445	0.33828998
Down Regulated Genes	GO_DNA_REPLICATION_INDEPENDENT_NUCLEOSOME_ORGANIZATION	1	-1.11197	0.34791252
Down Regulated Genes	GO_PROTEIN_POLYMERIZATION	3	-1.11142	0.36893204
Down Regulated Genes	GO_PERIPHERAL_NERVOUS_SYSTEM_DEVELOPMENT	1	-1.10927	0.3493976
Down Regulated Genes	GO_NUCLEOTIDE_EXCISION_REPAIR	1	-1.10915	0.3384913
Down Regulated Genes	GO_REGULATION_OF_RESPONSE_TO_STRESS	23	-1.10852	0.34741783
Down Regulated Genes	GO_PURINE_CONTAINING_COMPOUND_CATABOLIC_PROCESS	1	-1.10772	0.34146342
Down Regulated Genes	GO_BLEB_ASSEMBLY	2	-1.10757	0.33262712
Down Regulated Genes	GO_PROTEIN_AUTOPHOSPHORYLATION	9	-1.10723	0.34555984
Down Regulated Genes	GO_ENDOTHELIAL_CELL_MIGRATION	4	-1.10531	0.3659889
Down Regulated Genes	GO_TISSUE_REMODELING	4	-1.1021	0.34942085
Down Regulated Genes	GO_REGULATION_OF_STRESS_ACTIVATED_PROTEIN_KINASE_SIGNALING_C	3	-1.10097	0.3469388
Down Regulated Genes	GO_REGULATION_OF_REGULATED_SECRETORY_PATHWAY	3	-1.10092	0.38142294
Down Regulated Genes	GO_REGULATION_OF_GENE_EXPRESSION_EPIGENETIC	3	-1.10056	0.36647174
Down Regulated Genes	GO_NUCLEOTIDE_EXCISION_REPAIR_DNA_DAMAGE_RECOGNITION	1	-1.1001	0.35783365
Down Regulated Genes	GO_REGULATION_OF_CALCIUM_ION_IMPORT	1	-1.09979	0.34313726
Down Regulated Genes	GO_REGULATION_OF_TRANSCRIPTION_REGULATORY_REGION_DNA_BINDI	2	-1.0994	0.34226805
Down Regulated Genes	GO_NEGATIVE_REGULATION_OF_GENE_EXPRESSION_EPIGENETIC	3	-1.09808	0.39138943
Down Regulated Genes	GO_CALCIUM_ION_IMPORT	1	-1.09785	0.34489796
Down Regulated Genes	GO_INNER_CELL_MASS_CELL_PROLIFERATION	1	-1.09767	0.35699797
Down Regulated Genes	GO_MULTICELLULAR_ORGANISM_GROWTH	2	-1.09685	0.36346152
Down Regulated Genes	GO_TISSUE_MIGRATION	4	-1.0965	0.36772984
Down Regulated Genes	GO_REGULATION_OF_TRANSLATIONAL_FIDELITY	1	-1.09558	0.34583333
Down Regulated Genes	GO_CELLULAR_MODIFIED_AMINO_ACID_METABOLIC_PROCESS	3	-1.09446	0.4008097

Down Regulated Genes	GO_STRESS_ACTIVATED_PROTEIN_KINASE_SIGNALING_CASCADE	3	-1.09328	0.37278107
Down Regulated Genes	GO_WOUND_HEALING	11	-1.093	0.37044534
Down Regulated Genes	GO_DENDRITIC_SPINE_MAINTENANCE	1	-1.09065	0.37475345
Down Regulated Genes	GO_REGULATION_OF_CELL_AGING	2	-1.08986	0.35315984
Down Regulated Genes	GO_MICROTUBULE_BASED_PROCESS	7	-1.0898	0.3583815
Down Regulated Genes	GO_POSITIVE_REGULATION_OF_STRESS_ACTIVATED_PROTEIN_KINASE_SIG	3	-1.08954	0.3628866
Down Regulated Genes	GO_RESPONSE_TO_MUSCLE_ACTIVITY	1	-1.08931	0.34960938
Down Regulated Genes	GO_BLASTOCYST_GROWTH	1	-1.08809	0.36853004
Down Regulated Genes	GO_BLASTOCYST_DEVELOPMENT	2	-1.08765	0.37623763
Down Regulated Genes	GO_REGULATION_OF_STORE_OPERATED_CALCIUM_ENTRY	1	-1.08548	0.37214136
Down Regulated Genes	GO_ENDOMEMBRANE_SYSTEM_ORGANIZATION	13	-1.08508	0.35714287
Down Regulated Genes	GO_PROTEIN_HOMOOLIGOMERIZATION	2	-1.08461	0.37577003
Down Regulated Genes	GO_STORE_OPERATED_CALCIUM_ENTRY	1	-1.08353	0.37944663
Down Regulated Genes	GO_REGULATION_OF_POSTSYNAPTIC_NEUROTRANSMITTER_RECEPTOR_AC	1	-1.08298	0.3647059
Down Regulated Genes	GO_ALPHA_AMINO_ACID_CATABOLIC_PROCESS	1	-1.08211	0.3529412
Down Regulated Genes	GO_MUSCLE_FIBER_DEVELOPMENT	1	-1.08191	0.37214136
Down Regulated Genes	GO_PROTEIN_DENEDDYLATION	1	-1.08152	0.37577003
Down Regulated Genes	GO_NEGATIVE_REGULATION_OF_UBIQUITIN_DEPENDENT_PROTEIN_CATAB	1	-1.08077	0.3653061
Down Regulated Genes	GO_NEGATIVE_REGULATION_OF_PROTEASOMAL_UBIQUITIN_DEPENDENT_	1	-1.08056	0.35586482
Down Regulated Genes	GO_PHOSPHOLIPASE_C_ACTIVATING_G_PROTEIN_COUPLED_RECEPTOR_SI	1	-1.07921	0.362
Down Regulated Genes	GO_DICARBOXYLIC_ACID_BIOSYNTHETIC_PROCESS	1	-1.07893	0.36580518
Down Regulated Genes	GO_CENTRAL_NERVOUS_SYSTEM_DEVELOPMENT	22	-1.07864	0.38636363
Down Regulated Genes	GO_ENDOTHELIAL_CELL_PROLIFERATION	4	-1.07838	0.38195777
Down Regulated Genes	GO_RESPONSE_TO_COCAINE	1	-1.07823	0.35555556
Down Regulated Genes	GO_BEHAVIORAL_RESPONSE_TO_COCAINE	1	-1.07799	0.354717
Down Regulated Genes	GO_TRANSCRIPTION_COUPLED_NUCLEOTIDE_EXCISION_REPAIR	1	-1.07718	0.39494163
Down Regulated Genes	GO_SCF_DEPENDENT_PROTEASOMAL_UBIQUITIN_DEPENDENT_PROTEIN_C	1	-1.07654	0.38715953
Down Regulated Genes	GO_G_PROTEIN_COUPLED_GLUTAMATE_RECEPTOR_SIGNALING_PATHWAY	1	-1.07592	0.3884462
Down Regulated Genes	GO_AMINOACYL_TRNA_METABOLISM_INVOLVED_IN_TRANSLATIONAL_FID	1	-1.07473	0.37333333
Down Regulated Genes	GO_DICARBOXYLIC_ACID_TRANSPORT	1	-1.07457	0.37551868
Down Regulated Genes	GO_MYOTUBE_CELL_DEVELOPMENT	1	-1.07442	0.392233
Down Regulated Genes	GO_REGULATION_OF_CARBOHYDRATE_METABOLIC_PROCESS	6	-1.07403	0.39071566
Down Regulated Genes	GO_SKELETAL_MUSCLE_CONTRACTION	1	-1.07401	0.39958158
Down Regulated Genes	GO_JNK_CASCADE	3	-1.07386	0.396
Down Regulated Genes	GO_MULTIVESICULAR_BODY_ORGANIZATION	1	-1.07378	0.39376217
Down Regulated Genes	GO_REGULATION_OF_DENDRITIC_SPINE_MAINTENANCE	1	-1.07338	0.3581489

Down Regulated Genes	GO_SMOOTHENED_SIGNALING_PATHWAY	5	-1.07334	0.3767821
Down Regulated Genes	GO_NATURAL_KILLER_CELL_MEDIATED_IMMUNITY	1	-1.07328	0.36951983
Down Regulated Genes	GO_AMINO_ACID_HOMEOSTASIS	1	-1.07252	0.39473686
Down Regulated Genes	GO_NEGATIVE_REGULATION_OF_INTRINSIC_APOPTOTIC_SIGNALING_PATH	1	-1.07179	0.37473685
Down Regulated Genes	GO_POSITIVE_REGULATION_OF_CHROMATIN_ORGANIZATION	1	-1.07166	0.4163424
Down Regulated Genes	GO_MUSCLE_TISSUE_DEVELOPMENT	10	-1.07115	0.3858586
Down Regulated Genes	GO_NEGATIVE_REGULATION_OF_CYSTEINE_TYPE_ENDOPEPTIDASE_ACTIVI	2	-1.07074	0.37731957
Down Regulated Genes	GO_REGULATION_OF_REACTIVE_OXYGEN_SPECIES_BIOSYNTHETIC_PROCES	1	-1.07015	0.3801498
Down Regulated Genes	GO_POSITIVE_REGULATION_OF_JNK_CASCADE	3	-1.06988	0.38264298
Down Regulated Genes	GO_POSITIVE_REGULATION_OF_HISTONE_METHYLATION	1	-1.06895	0.37964776
Down Regulated Genes	GO_REGULATION_OF_INTRINSIC_APOPTOTIC_SIGNALING_PATHWAY_IN_RI	1	-1.06886	0.39484128
Down Regulated Genes	GO_GLUTAMINE_METABOLIC_PROCESS	1	-1.0679	0.4024145
Down Regulated Genes	GO_SKELETAL_MUSCLE_CELL_PROLIFERATION	1	-1.06729	0.3968254
Down Regulated Genes	GO_GLUTAMATE_SECRETION	1	-1.06723	0.37278107
Down Regulated Genes	GO_REGULATION_OF_HISTONE_METHYLATION	1	-1.06593	0.39430895
Down Regulated Genes	GO_ORGANELLE_LOCALIZATION	10	-1.06556	0.38155136
Down Regulated Genes	GO_MULTICELLULAR_ORGANISMAL_MOVEMENT	1	-1.06483	0.4181818
Down Regulated Genes	GO_ACIDIC_AMINO_ACID_TRANSPORT	1	-1.0642	0.38821137
Down Regulated Genes	GO_HEART_PROCESS	5	-1.06405	0.3824092
Down Regulated Genes	GO_HOMEOSTASIS_OF_NUMBER_OF_CELLS	3	-1.06361	0.39121756
Down Regulated Genes	GO_REGULATION_OF_GLUCAN_BIOSYNTHETIC_PROCESS	2	-1.06359	0.35546875
Down Regulated Genes	GO_DICARBOXYLIC_ACID_METABOLIC_PROCESS	1	-1.06237	0.3987976
Down Regulated Genes	GO_POSITIVE_REGULATION_OF_REACTIVE_OXYGEN_SPECIES_METABOLIC_	1	-1.06182	0.40038684
Down Regulated Genes	GO_POSITIVE_REGULATION_OF_PHOSPHORUS_METABOLIC_PROCESS	14	-1.06135	0.38779956
Down Regulated Genes	GO_CELLULAR_GLUCAN_METABOLIC_PROCESS	2	-1.06104	0.4027505
Down Regulated Genes	GO_NEGATIVE_REGULATION_OF_CELLULAR_SENESCENCE	1	-1.06084	0.41164657
Down Regulated Genes	GO_NEGATIVE_REGULATION_OF_INTRINSIC_APOPTOTIC_SIGNALING_PATH	1	-1.06084	0.4
Down Regulated Genes	GO_INHIBITION_OF_CYSTEINE_TYPE_ENDOPEPTIDASE_ACTIVITY_INVOLVED	2	-1.0608	0.4069098
Down Regulated Genes	GO_ENERGY_RESERVE_METABOLIC_PROCESS	2	-1.06077	0.37911025
Down Regulated Genes	GO_SUCKLING_BEHAVIOR	1	-1.06077	0.41735536
Down Regulated Genes	GO_POSITIVE_REGULATION_OF_CELLULAR_CARBOHYDRATE_METABOLIC_F	2	-1.06076	0.38857144
Down Regulated Genes	GO_METAL_ION_TRANSPORT	14	-1.06068	0.37878788
Down Regulated Genes	GO_CALCIUM_ION_REGULATED_EXOCYTOSIS	3	-1.06013	0.41199225
Down Regulated Genes	GO_MITOCHONDRION_ORGANIZATION	15	-1.05931	0.38651684
Down Regulated Genes	GO_NATURAL_KILLER_CELL_ACTIVATION	1	-1.05827	0.40952381
Down Regulated Genes	GO_REGULATION_OF_GLYCOGEN_METABOLIC_PROCESS	2	-1.05826	0.3976143

Down Regulated Genes	GO_NEGATIVE_REGULATION_OF_SIGNAL_TRANSDUCTION_BY_P53_CLASS_	1	-1.05686	0.40115163
Down Regulated Genes	GO_NEGATIVE_REGULATION_OF_CELL_AGING	1	-1.05677	0.41811174
Down Regulated Genes	GO_NCRNA_METABOLIC_PROCESS	13	-1.05637	0.3898305
Down Regulated Genes	GO_CELLULAR_CARBOHYDRATE_BIOSYNTHETIC_PROCESS	2	-1.05554	0.42190668
Down Regulated Genes	GO_NATURAL_KILLER_CELL_DEGRANULATION	1	-1.05541	0.41700405
Down Regulated Genes	GO_REGULATION_OF_INTRINSIC_APOPTOTIC_SIGNALING_PATHWAY_IN_RI	1	-1.05533	0.4168421
Down Regulated Genes	GO_GLUTAMATE_METABOLIC_PROCESS	1	-1.05516	0.43811396
Down Regulated Genes	GO_NATURAL_KILLER_CELL_ACTIVATION_INVOLVED_IN_IMMUNE_RESPON	1	-1.05475	0.4157088
Down Regulated Genes	GO_REGIONALIZATION	5	-1.05471	0.39562625
Down Regulated Genes	GO_MULTIVESICULAR_BODY_SORTING_PATHWAY	1	-1.05443	0.41769546
Down Regulated Genes	GO_NEGATIVE_REGULATION_OF_RESPONSE_TO_DNA_DAMAGE_STIMULUS	1	-1.05385	0.4352227
Down Regulated Genes	GO_REGULATION_OF_MUSCLE_CELL_DIFFERENTIATION	3	-1.05349	0.40630797
Down Regulated Genes	GO_GLYCOGEN_BIOSYNTHETIC_PROCESS	2	-1.05349	0.40630797
Down Regulated Genes	GO_LEUKOCYTE_MIGRATION	11	-1.05299	0.38955823
Down Regulated Genes	GO_REGULATION_OF_HISTONE_MODIFICATION	1	-1.05281	0.4194831
Down Regulated Genes	GO_GLUTAMINE_FAMILY_AMINO_ACID_CATABOLIC_PROCESS	1	-1.05268	0.44642857
Down Regulated Genes	GO_GLUTAMATE_BIOSYNTHETIC_PROCESS	1	-1.05232	0.42629483
Down Regulated Genes	GO_RNA_SPLICING_VIA_ENDONUCLEOLYTIC_CLEAVAGE_AND_LIGATION	1	-1.05196	0.4214876
Down Regulated Genes	GO_REGULATION_OF_LIPASE_ACTIVITY	1	-1.05161	0.39641434
Down Regulated Genes	GO_CALCINEURIN_MEDIATED_SIGNALING	2	-1.0516	0.39173228
Down Regulated Genes	GO_REGULATION_OF_VASCULATURE_DEVELOPMENT	4	-1.05097	0.39583334
Down Regulated Genes	GO_REACTIVE_OXYGEN_SPECIES_BIOSYNTHETIC_PROCESS	1	-1.05062	0.40963855
Down Regulated Genes	GO_IRES_DEPENDENT_VIRAL_TRANSLATIONAL_INITIATION	2	-1.05054	0.416499
Down Regulated Genes	GO_POSITIVE_REGULATION_OF_GLUCONEOGENESIS	1	-1.05034	0.40778688
Down Regulated Genes	GO_MITOCHONDRIAL_OUTER_MEMBRANE_PERMEABILIZATION	1	-1.05031	0.4158215
Down Regulated Genes	GO_POSITIVE_REGULATION_OF_GLUCOSE_METABOLIC_PROCESS	2	-1.05026	0.3942857
Down Regulated Genes	GO_POSITIVE_REGULATION_OF_MYOBLAST_PROLIFERATION	1	-1.0502	0.4253112
Down Regulated Genes	GO_PROTEIN_HOMOTETRAMERIZATION	2	-1.04997	0.42095238
Down Regulated Genes	GO_MITOCHONDRIAL_TRANSPORT	4	-1.04965	0.4034417
Down Regulated Genes	GO_TRANSPORT_ALONG_MICROTUBULE	4	-1.0496	0.41269842
Down Regulated Genes	GO_DNA_METABOLIC_PROCESS	8	-1.04903	0.36034116
Down Regulated Genes	GO_MITOCHONDRIAL_PROTEIN_CATABOLIC_PROCESS	1	-1.04859	0.4034417
Down Regulated Genes	GO_POSITIVE_REGULATION_OF_CARBOHYDRATE_METABOLIC_PROCESS	2	-1.04856	0.41555977
Down Regulated Genes	GO_POSITIVE_REGULATION_OF_PROTEIN_MODIFICATION_BY_SMALL_PRO	2	-1.04763	0.42828685
Down Regulated Genes	GO_REGULATION_OF_NUCLEOCYTOPLASMIC_TRANSPORT	3	-1.04594	0.4288425
Down Regulated Genes	GO_REGULATION_OF_MITOCHONDRIAL_MEMBRANE_PERMEABILITY_INVO	1	-1.04525	0.43159923

Down Regulated Genes	GO_NEGATIVE_REGULATION_OF_CARDIAC_MUSCLE_TISSUE_GROWTH	1	-1.04521	0.41532257
Down Regulated Genes	GO_REGULATION_OF_MEMBRANE_PERMEABILITY	1	-1.04419	0.4296875
Down Regulated Genes	GO_MITOCHONDRIAL_MEMBRANE_ORGANIZATION	1	-1.04377	0.4299611
Down Regulated Genes	GO_ENERGY_DERIVATION_BY_OXIDATION_OF_ORGANIC_COMPOUNDS	6	-1.04325	0.3910387
Down Regulated Genes	GO_REGULATION_OF_AUTOPHAGY_OF_MITOCHONDRION	1	-1.04301	0.4288618
Down Regulated Genes	GO_CONSTITUTIVE_SECRETORY_PATHWAY	1	-1.04295	0.45576924
Down Regulated Genes	GO_NEUTROPHIL_MIGRATION	2	-1.04295	0.4
Down Regulated Genes	GO_CELL_PROJECTION_ASSEMBLY	14	-1.04139	0.43870968
Down Regulated Genes	GO_REGULATION_OF_CYSTEINE_TYPE_ENDOPEPTIDASE_ACTIVITY	2	-1.04092	0.41208792
Down Regulated Genes	GO_ACTIVATION_OF_PHOSPHOLIPASE_D_ACTIVITY	1	-1.03941	0.4317719
Down Regulated Genes	GO_NEGATIVE_REGULATION_OF_MEMBRANE_POTENTIAL	1	-1.0393	0.4288499
Down Regulated Genes	GO_POSITIVE_REGULATION_OF_LIPASE_ACTIVITY	1	-1.03927	0.4301887
Down Regulated Genes	GO_REGULATION_OF_PHOSPHOLIPASE_ACTIVITY	1	-1.03922	0.4573304
Down Regulated Genes	GO_INOSITOL_PHOSPHATE_MEDIATED_SIGNALING	2	-1.03864	0.43222004
Down Regulated Genes	GO_POSITIVE_REGULATION_OF_REACTIVE_OXYGEN_SPECIES_BIOSYNTHETI	1	-1.03845	0.45842218
Down Regulated Genes	GO_POSITIVE_REGULATION_OF_PHOSPHOLIPASE_ACTIVITY	1	-1.03792	0.446281
Down Regulated Genes	GO_ZYMOGEN_INHIBITION	2	-1.03787	0.4266145
Down Regulated Genes	GO_REGULATION_OF_INNATE_IMMUNE_RESPONSE	2	-1.03759	0.40874526
Down Regulated Genes	GO_POSITIVE_REGULATION_OF_PROTEIN_MODIFICATION_PROCESS	15	-1.03501	0.41935483
Down Regulated Genes	GO_POSITIVE_REGULATION_OF_GRANULOCYTE_CHEMOTAXIS	2	-1.03434	0.41967872
Down Regulated Genes	GO_REGULATION_OF_PROTEIN_MODIFICATION_PROCESS	24	-1.03417	0.42610836
Down Regulated Genes	GO_RIBOSOME_ASSEMBLY	1	-1.03381	0.4556701
Down Regulated Genes	GO_AUTOPHAGY_OF_MITOCHONDRION	1	-1.0337	0.45136186
Down Regulated Genes	GO_POSITIVE_REGULATION_OF_MEMBRANE_PERMEABILITY	1	-1.03365	0.4515464
Down Regulated Genes	GO_NEGATIVE_REGULATION_OF_CARDIAC_MUSCLE_CELL_PROLIFERATION	1	-1.03357	0.43737575
Down Regulated Genes	GO_RNA_MODIFICATION	5	-1.03165	0.41649485
Down Regulated Genes	GO_RIBOSOME_DISASSEMBLY	2	-1.03092	0.4431599
Down Regulated Genes	GO_NEGATIVE_REGULATION_OF_CARDIAC_MUSCLE_TISSUE_DEVELOPMEN	1	-1.03015	0.45553538
Down Regulated Genes	GO_DEVELOPMENTAL_MATURATION	7	-1.03011	0.4092742
Down Regulated Genes	GO_TISSUE_MORPHOGENESIS	21	-1.03009	0.42256638
Down Regulated Genes	GO_REGULATION_OF_GLUCONEOGENESIS	1	-1.02971	0.45785442
Down Regulated Genes	GO_POSITIVE_REGULATION_OF_DEVELOPMENTAL_PROCESS	31	-1.0292	0.4591346
Down Regulated Genes	GO_NEGATIVE_REGULATION_OF_ORGAN_GROWTH	1	-1.02916	0.44989774
Down Regulated Genes	GO_POSITIVE_REGULATION_OF_NEUTROPHIL_MIGRATION	2	-1.02904	0.4248497
Down Regulated Genes	GO_RIBOSOMAL_LARGE_SUBUNIT_ASSEMBLY	1	-1.02887	0.46975806
Down Regulated Genes	GO_NUCLEOBASE_CONTAINING_SMALL_MOLECULE_BIOSYNTHETIC_PROCE	3	-1.02872	0.42829457

Down Regulated Genes	GO_FORMATION_OF_PRIMARY_GERM_LAYER	3	-1.02807	0.44094488
Down Regulated Genes	GO_RETINA_VASCULATURE_DEVELOPMENT_IN_CAMERA_TYPE_EYE	1	-1.02788	0.45908183
Down Regulated Genes	GO_REGULATION_OF_NEUTROPHIL_CHEMOTAXIS	2	-1.02746	0.44071147
Down Regulated Genes	GO_FORMATION_OF_TRANSLATION_PREINITIATION_COMPLEX	2	-1.02688	0.4390244
Down Regulated Genes	GO_MYOBLAST_DIFFERENTIATION	3	-1.02653	0.40954274
Down Regulated Genes	GO_REGULATION_OF_STEM_CELL_POPULATION_MAINTENANCE	1	-1.0265	0.46516007
Down Regulated Genes	GO_NIK_NF_KAPPAB_SIGNALING	2	-1.02632	0.4491018
Down Regulated Genes	GO_REGULATION_OF_NEUTROPHIL_MIGRATION	2	-1.0261	0.43855932
Down Regulated Genes	GO_MICROTUBULE_BASED_TRANSPORT	4	-1.02562	0.4229249
Down Regulated Genes	GO_NEGATIVE_REGULATION_OF_GLUCOSE_TRANSMEMBRANE_TRANSPOR	1	-1.02525	0.43157893
Down Regulated Genes	GO_CELL_CELL_SIGNALING_BY_WNT	8	-1.02469	0.41776937
Down Regulated Genes	GO_RECEPTOR_CLUSTERING	1	-1.0245	0.47692308
Down Regulated Genes	GO_REGULATION_OF_GRANULOCYTE_CHEMOTAXIS	2	-1.02417	0.44827586
Down Regulated Genes	GO_NEUROTRANSMITTER_TRANSPORT	2	-1.02237	0.43653846
Down Regulated Genes	GO_NEUTROPHIL_CHEMOTAXIS	2	-1.02083	0.4418146
Down Regulated Genes	GO_ENDOTHELIAL_CELL_MORPHOGENESIS	1	-1.02074	0.46218488
Down Regulated Genes	GO_REGULATION_OF_CALCIUM_ION_DEPENDENT_EXOCYTOSIS	2	-1.02048	0.4484127
Down Regulated Genes	GO_VIRAL_TRANSLATION	2	-1.01981	0.45703125
Down Regulated Genes	GO_RETINA_VASCULATURE_MORPHOGENESIS_IN_CAMERA_TYPE_EYE	1	-1.01975	0.45957446
Down Regulated Genes	GO_RIBOSOMAL_LARGE_SUBUNIT_BIOGENESIS	1	-1.01955	0.48987854
Down Regulated Genes	GO_NUCLEIC_ACID_PHOSPHODIESTER_BOND_HYDROLYSIS	6	-1.019	0.42477876
Down Regulated Genes	GO_NEGATIVE_REGULATION_OF_MUSCLE_TISSUE_DEVELOPMENT	1	-1.01886	0.4691358
Down Regulated Genes	GO_NEGATIVE_REGULATION_OF_DEVELOPMENTAL_PROCESS	21	-1.01876	0.43507972
Down Regulated Genes	GO_NEGATIVE_REGULATION_OF_GLYCOGEN_METABOLIC_PROCESS	1	-1.01808	0.45417514
Down Regulated Genes	GO_REGULATION_OF_CARBOHYDRATE_BIOSYNTHETIC_PROCESS	4	-1.01704	0.46963564
Down Regulated Genes	GO_MICROTUBULE_BASED_MOVEMENT	4	-1.01668	0.4448743
Down Regulated Genes	GO_MRNA_PSEUDOURIDINE_SYNTHESIS	1	-1.0157	0.47667342
Down Regulated Genes	GO_INTRINSIC_APOPTOTIC_SIGNALING_PATHWAY_BY_P53_CLASS_MEDIAT	4	-1.01555	0.4474708
Down Regulated Genes	GO_REGULATION_OF_NEUROTRANSMITTER_LEVELS	2	-1.01525	0.4730539
Down Regulated Genes	GO_ALTERNATIVE_MRNA_SPLICING_VIA_SPLICEOSOME	3	-1.01491	0.44176707
Down Regulated Genes	GO_MRNA_MODIFICATION	1	-1.01441	0.48565573
Down Regulated Genes	GO_PSEUDOURIDINE_SYNTHESIS	1	-1.01343	0.44894028
Down Regulated Genes	GO_NEGATIVE_REGULATION_OF_TRANSCRIPTION_BY_RNA_POLYMERASE_	22	-1.01332	0.46205357
Down Regulated Genes	GO_SIGNAL_TRANSDUCTION_BY_P53_CLASS_MEDIATOR	4	-1.0129	0.48360655
Down Regulated Genes	GO_GRANULOCYTE_CHEMOTAXIS	2	-1.01194	0.4526112
Down Regulated Genes	GO_PROTON_TRANSPORTING_ATP_SYNTHASE_COMPLEX_ASSEMBLY	1	-1.00937	0.47325101

Down Regulated Genes	GO_POSITIVE_REGULATION_OF_INTERLEUKIN_6_PRODUCTION	1	-1.00862	0.47008547
Down Regulated Genes	GO_SEQUESTERING_OF_TRIGLYCERIDE	1	-1.00826	0.48949578
Down Regulated Genes	GO_NEGATIVE_REGULATION_OF_FAT_CELL_DIFFERENTIATION	1	-1.00754	0.4896694
Down Regulated Genes	GO_REGULATION_OF_PROTEIN_COMPLEX_STABILITY	1	-1.00718	0.46734694
Down Regulated Genes	GO_REGULATION_OF_CYTOSOLIC_CALCIUM_ION_CONCENTRATION	4	-1.00629	0.46183953
Down Regulated Genes	GO_REGULATION_OF_CELLULAR_RESPONSE_TO_INSULIN_STIMULUS	1	-1.00552	0.504
Down Regulated Genes	GO_CELLULAR_DIVALENT_INORGANIC_ANION_HOMEOSTASIS	1	-1.00506	0.49278352
Down Regulated Genes	GO_CELLULAR_ANION_HOMEOSTASIS	1	-1.00504	0.49499
Down Regulated Genes	GO_VESICLE_TETHERING_TO_GOLGI	1	-1.00474	0.4844358
Down Regulated Genes	GO_MONOVALENT_INORGANIC_ANION_HOMEOSTASIS	1	-1.00454	0.502
Down Regulated Genes	GO_VESICLE_TETHERING	1	-1.00453	0.4802495
Down Regulated Genes	GO_ENTRY_INTO_HOST	5	-1.00419	0.47131148
Down Regulated Genes	GO_3_PHOSPHOADENOSINE_5_PHOSPHOSULFATE_METABOLIC_PROCESS	1	-1.00359	0.5097276
Down Regulated Genes	GO_FLAVIN_CONTAINING_COMPOUND_METABOLIC_PROCESS	1	-1.0028	0.49801588
Down Regulated Genes	GO_NADH_DEHYDROGENASE_COMPLEX_ASSEMBLY	2	-1.00258	0.47692308
Down Regulated Genes	GO_NEGATIVE_REGULATION_OF_CELLULAR_RESPONSE_TO_INSULIN_STIMU	1	-1.00196	0.5146771
Down Regulated Genes	GO_MUSCLE_STRUCTURE_DEVELOPMENT	14	-1.00184	0.4819533
Down Regulated Genes	GO_GRANULOCYTE_MIGRATION	2	-1.00161	0.45792565
Down Regulated Genes	GO_RIBONUCLEOSIDE_TRIPHOSPHATE_METABOLIC_PROCESS	3	-1.001	0.4731801
Down Regulated Genes	GO_T_CELL_HOMEOSTASIS	1	-0.99966	0.502008
Down Regulated Genes	GO_REGULATION_OF_PHOSPHORUS_METABOLIC_PROCESS	25	-0.99962	0.4335512
Down Regulated Genes	GO_DIVALENT_INORGANIC_ANION_HOMEOSTASIS	1	-0.99943	0.50718683
Down Regulated Genes	GO_SYNAPTIC_VESICLE_EXOCYTOSIS	2	-0.99924	0.45792565
Down Regulated Genes	GO_REGULATION_OF_OXIDOREDUCTASE_ACTIVITY	2	-0.9986	0.45279384
Down Regulated Genes	GO_NEURON_PROJECTION_GUIDANCE	14	-0.99856	0.44948453
Down Regulated Genes	GO_PROTEIN_TARGETING	15	-0.99827	0.4804928
Down Regulated Genes	GO_EMBRYONIC_BRAIN_DEVELOPMENT	1	-0.99811	0.4909091
Down Regulated Genes	GO_GASTRULATION	5	-0.99809	0.46351084
Down Regulated Genes	GO_NEUROTRANSMITTER_SECRETION	2	-0.99769	0.47878787
Down Regulated Genes	GO_GTP_METABOLIC_PROCESS	3	-0.99722	0.47619048
Down Regulated Genes	GO_INTERLEUKIN_6_PRODUCTION	1	-0.99709	0.5049116
Down Regulated Genes	GO_REGULATION_OF_T_CELL_MEDIATED_CYTOTOXICITY	1	-0.99696	0.505176
Down Regulated Genes	GO_LOCOMOTION_INVOLVED_IN_LOCOMOTORY_BEHAVIOR	1	-0.99617	0.48795182
Down Regulated Genes	GO_POSITIVE_REGULATION_OF_CELL_DIFFERENTIATION	19	-0.99556	0.46724892
Down Regulated Genes	GO_TUMOR_NECROSIS_FACTOR_SUPERFAMILY_CYTOKINE_PRODUCTION	1	-0.99526	0.5101626
Down Regulated Genes	GO_NUCLEOSIDE_TRIPHOSPHATE_CATABOLIC_PROCESS	1	-0.99497	0.50992066

Down Regulated Genes	GO_NEGATIVE_REGULATION_OF_GLUCOSE_IMPORT	1	-0.99425	0.51102203
Down Regulated Genes	GO_TAXIS	20	-0.99405	0.4520548
Down Regulated Genes	GO_MITOCHONDRIAL_RNA_PROCESSING	1	-0.99279	0.4919355
Down Regulated Genes	GO_MOVEMENT_IN_HOST_ENVIRONMENT	5	-0.99207	0.45147678
Down Regulated Genes	GO_REGULATION_OF_PROTEASOMAL_PROTEIN_CATABOLIC_PROCESS	5	-0.99177	0.45472062
Down Regulated Genes	GO_LEUKOCYTE_HOMEOSTASIS	1	-0.9916	0.48742747
Down Regulated Genes	GO_BLOOD_VESSEL_ENDOTHELIAL_CELL_MIGRATION	1	-0.99148	0.49250937
Down Regulated Genes	GO_REGULATION_OF_DNA_BINDING	3	-0.98993	0.47173488
Down Regulated Genes	GO_LYMPHOCYTE_HOMEOSTASIS	1	-0.98983	0.5099602
Down Regulated Genes	GO_NEGATIVE_REGULATION_OF_T_CELL_MEDIATED_CYTOTOXICITY	1	-0.98914	0.51934826
Down Regulated Genes	GO_PURINE_NUCLEOSIDE_TRIPHOSPHATE_METABOLIC_PROCESS	3	-0.98892	0.47953215
Down Regulated Genes	GO_REGULATION_OF_RESPONSE_TO_WOUNDING	6	-0.98865	0.4547244
Down Regulated Genes	GO_EMBRYONIC_MORPHOGENESIS	15	-0.98851	0.48076922
Down Regulated Genes	GO_NEGATIVE_REGULATION_OF_TRANSMEMBRANE_TRANSPORT	1	-0.98851	0.5386064
Down Regulated Genes	GO_REGULATION_OF_T_CELL_MEDIATED_IMMUNITY	1	-0.98812	0.5243665
Down Regulated Genes	GO_PROTEIN_PALMITOYLATION	3	-0.98718	0.48046875
Down Regulated Genes	GO_REGULATION_OF_SECRETION	7	-0.98494	0.47254902
Down Regulated Genes	GO_REGULATION_OF_LEUKOCYTE_MEDIATED_CYTOTOXICITY	1	-0.9848	0.52455795
Down Regulated Genes	GO_NEGATIVE_REGULATION_OF_LYMPHOCYTE_MEDIATED_IMMUNITY	1	-0.98461	0.50901806
Down Regulated Genes	GO_CELL_PROJECTION_ORGANIZATION	45	-0.98443	0.49608356
Down Regulated Genes	GO_POSITIVE_REGULATION_OF_INSULIN_SECRETION_INVOLVED_IN_CELLU	1	-0.98411	0.50406504
Down Regulated Genes	GO_NEGATIVE_REGULATION_OF_T_CELL_MEDIATED_IMMUNITY	1	-0.98385	0.499002
Down Regulated Genes	GO_REGULATION_OF_LEUKOCYTE_MEDIATED_IMMUNITY	1	-0.98358	0.53688526
Down Regulated Genes	GO_SULFUR_COMPOUND_BIOSYNTHETIC_PROCESS	3	-0.98356	0.47851562
Down Regulated Genes	GO_GUANOSINE_CONTAINING_COMPOUND_METABOLIC_PROCESS	3	-0.98311	0.5020161
Down Regulated Genes	GO_PROTEIN_CONTAINING_COMPLEX_SUBUNIT_ORGANIZATION	30	-0.98254	0.4763033
Down Regulated Genes	GO_MRNA_CIS_SPLICING_VIA_SPLICEOSOME	1	-0.98221	0.5317919
Down Regulated Genes	GO_MITOCHONDRIAL_RNA_METABOLIC_PROCESS	1	-0.98192	0.49492902
Down Regulated Genes	GO_CONNECTIVE_TISSUE_DEVELOPMENT	13	-0.98165	0.4888393
Down Regulated Genes	GO_NEGATIVE_REGULATION_OF_LEUKOCYTE_MEDIATED_CYTOTOXICITY	1	-0.9809	0.54133856
Down Regulated Genes	GO_VASCULAR_ENDOTHELIAL_GROWTH_FACTOR_RECEPTOR_SIGNALING_I	2	-0.98085	0.49031007
Down Regulated Genes	GO_TRNA_METHYLATION	1	-0.98071	0.51361865
Down Regulated Genes	GO_REGULATION_OF_ADAPTIVE_IMMUNE_RESPONSE	1	-0.98062	0.5218254
Down Regulated Genes	GO_NEGATIVE_REGULATION_OF_CELL_KILLING	1	-0.9795	0.5294118
Down Regulated Genes	GO_POSITIVE_REGULATION_OF_EMBRYONIC_DEVELOPMENT	1	-0.97897	0.51428574
Down Regulated Genes	GO_POSITIVE_REGULATION_OF_DEVELOPMENTAL_GROWTH	5	-0.97888	0.4737864

Down Regulated Genes	GO_NEGATIVE_REGULATION_OF_ADAPTIVE_IMMUNE_RESPONSE	1	-0.97855	0.54313725
Down Regulated Genes	GO_NEGATIVE_REGULATION_OF_PROTEIN_AUTOPHOSPHORYLATION	1	-0.97785	0.56150794
Down Regulated Genes	GO_NEGATIVE_REGULATION_OF_LEUKOCYTE_MEDIATED_IMMUNITY	1	-0.97699	0.5320388
Down Regulated Genes	GO_POSITIVE_REGULATION_OF_PROTEIN_AUTOPHOSPHORYLATION	1	-0.97698	0.5139442
Down Regulated Genes	GO_RNA_POLYADENYLATION	2	-0.97546	0.48790324
Down Regulated Genes	GO_COLUMNAR_CUBOIDAL_EPITHELIAL_CELL_DIFFERENTIATION	3	-0.97496	0.5110664
Down Regulated Genes	GO_RIBONUCLEOSIDE_METABOLIC_PROCESS	4	-0.97323	0.5009901
Down Regulated Genes	GO_REGULATION_OF_LYMPHOCYTE_MEDIATED_IMMUNITY	1	-0.9731	0.5425331
Down Regulated Genes	GO_TRNA_WOBBLE_URIDINE_MODIFICATION	1	-0.97067	0.5627451
Down Regulated Genes	GO_PROTEIN_LOCALIZATION_TO_MITOCHONDRION	3	-0.97059	0.4893204
Down Regulated Genes	GO_VASCULOGENESIS	1	-0.97043	0.5409836
Down Regulated Genes	GO_TRNA_WOBBLE_BASE_MODIFICATION	1	-0.97015	0.556
Down Regulated Genes	GO_REGULATION_OF_UBIQUITIN_DEPENDENT_PROTEIN_CATABOLIC_PROC	4	-0.96962	0.5029354
Down Regulated Genes	GO_GLYCOSYL_COMPOUND_METABOLIC_PROCESS	4	-0.9685	0.4895238
Down Regulated Genes	GO_ESTABLISHMENT_OF_ENDOTHELIAL_INTESTINAL_BARRIER	1	-0.96827	0.53144014
Down Regulated Genes	GO_MULTICELLULAR_ORGANISMAL_HOMEOSTASIS	6	-0.96736	0.49011856
Down Regulated Genes	GO_MITOCHONDRIAL_RNA_MODIFICATION	1	-0.96714	0.54633206
Down Regulated Genes	GO_RIBOSE_PHOSPHATE_BIOSYNTHETIC_PROCESS	4	-0.96687	0.4765625
Down Regulated Genes	GO_NEGATIVE_REGULATION_OF_GTPASE_ACTIVITY	1	-0.9662	0.54313725
Down Regulated Genes	GO_RESPONSE_TO_HEAT	1	-0.96375	0.5408163
Down Regulated Genes	GO_CELLULAR_AMINO_ACID_METABOLIC_PROCESS	7	-0.96366	0.48790324
Down Regulated Genes	GO_MITOCHONDRIAL_TRNA_PROCESSING	1	-0.963	0.5482546
Down Regulated Genes	GO_ESTABLISHMENT_OR_MAINTENANCE_OF_CELL_POLARITY	5	-0.96249	0.49905482
Down Regulated Genes	GO_SENSORY_ORGAN_MORPHOGENESIS	5	-0.96224	0.50769234
Down Regulated Genes	GO_REGULATION_OF_GENE_SILENCING	1	-0.96003	0.5708955
Down Regulated Genes	GO_REGULATION_OF_POSTTRANSCRIPTIONAL_GENE_SILENCING	1	-0.9595	0.5807087
Down Regulated Genes	GO_PROTEIN_LOCALIZATION_TO_NUCLEAR_ENVELOPE	1	-0.95949	0.556
Down Regulated Genes	GO_TRNA_TRANSPORT	1	-0.95913	0.5496183
Down Regulated Genes	GO_ESTABLISHMENT_OF_ENDOTHELIAL_BARRIER	1	-0.95881	0.5503876
Down Regulated Genes	GO_CELLULAR_RESPONSE_TO_HEAT	1	-0.95879	0.5525773
Down Regulated Genes	GO_CRANIOFACIAL_SUTURE_MORPHOGENESIS	2	-0.95763	0.52286285
Down Regulated Genes	GO_NCRNA_EXPORT_FROM_NUCLEUS	1	-0.95736	0.5538793
Down Regulated Genes	GO_REGULATION_OF_CELLULAR_RESPONSE_TO_HEAT	1	-0.95509	0.58
Down Regulated Genes	GO_NUCLEAR_PORE_ORGANIZATION	1	-0.95458	0.5449219
Down Regulated Genes	GO_AMINO_ACID_ACTIVATION	2	-0.95448	0.516129
Down Regulated Genes	GO_PALLIUM_DEVELOPMENT	5	-0.95382	0.49904397

Down Regulated Genes	GO_RAP_PROTEIN_SIGNAL_TRANSDUCTION	1	-0.95347	0.57473683
Down Regulated Genes	GO_AXONAL_FASCICULATION	5	-0.95289	0.51759833
Down Regulated Genes	GO_SENSORY_PERCEPTION_OF_MECHANICAL_STIMULUS	4	-0.95231	0.55009454
Down Regulated Genes	GO_DIENCEPHALON_DEVELOPMENT	1	-0.95145	0.56133056
Down Regulated Genes	GO_NEGATIVE_REGULATION_OF_NUCLEOBASE_CONTAINING_COMPOUND_	29	-0.95096	0.51167727
Down Regulated Genes	GO_HINDLIMB_MORPHOGENESIS	1	-0.95096	0.55284554
Down Regulated Genes	GO_REGULATION_OF_CELLULAR_CARBOHYDRATE_METABOLIC_PROCESS	4	-0.95084	0.52362204
Down Regulated Genes	GO_KIDNEY_MORPHOGENESIS	2	-0.95077	0.5494506
Down Regulated Genes	GO_NEGATIVE_REGULATION_OF_ERBB_SIGNALING_PATHWAY	1	-0.95072	0.5609284
Down Regulated Genes	GO_RESPIRATORY_SYSTEM_DEVELOPMENT	5	-0.95012	0.5564516
Down Regulated Genes	GO_MYOBLAST_FATE_COMMITMENT	1	-0.95004	0.5811088
Down Regulated Genes	GO_NUCLEOSIDE_METABOLIC_PROCESS	4	-0.94909	0.53306615
Down Regulated Genes	GO_PROTEIN_TARGETING_TO_MITOCHONDRION	3	-0.94835	0.5150301
Down Regulated Genes	GO_REGULATION_OF_PROTEASOMAL_UBIQUITIN_DEPENDENT_PROTEIN_C	4	-0.94774	0.52692306
Down Regulated Genes	GO_ORGANONITROGEN_COMPOUND_CATABOLIC_PROCESS	27	-0.94764	0.49887133
Down Regulated Genes	GO_NUCLEOSIDE_PHOSPHATE_BIOSYNTHETIC_PROCESS	4	-0.94706	0.5140562
Down Regulated Genes	GO_DOUBLE_STRAND_BREAK_REPAIR	1	-0.9467	0.562249
Down Regulated Genes	GO_ODONTOGENESIS_OF_DENTIN_CONTAINING_TOOTH	2	-0.94668	0.539916
Down Regulated Genes	GO_NLS_BEARING_PROTEIN_IMPORT_INTO_NUCLEUS	1	-0.94577	0.5871369
Down Regulated Genes	GO_ERYTHROCYTE_HOMEOSTASIS	2	-0.94412	0.53949904
Down Regulated Genes	GO_CEREBRAL_CORTEX_DEVELOPMENT	5	-0.9436	0.5281553
Down Regulated Genes	GO_EMBRYONIC_HINDLIMB_MORPHOGENESIS	1	-0.94335	0.59006214
Down Regulated Genes	GO_REGULATION_OF_DNA_REPAIR	1	-0.94243	0.59225094
Down Regulated Genes	GO_AXIS_SPECIFICATION	2	-0.94206	0.5305343
Down Regulated Genes	GO_ERBB_SIGNALING_PATHWAY	1	-0.94159	0.58958334
Down Regulated Genes	GO_ANIMAL_ORGAN_MORPHOGENESIS	25	-0.94136	0.5179856
Down Regulated Genes	GO_REGULATION_OF_ERBB_SIGNALING_PATHWAY	1	-0.941	0.582505
Down Regulated Genes	GO_PURINE_NUCLEOSIDE_METABOLIC_PROCESS	4	-0.94035	0.54989815
Down Regulated Genes	GO_REGULATION_OF_DOUBLE_STRAND_BREAK_REPAIR_VIA_HOMOLOGOU	1	-0.94018	0.5615866
Down Regulated Genes	GO_GLIAL_CELL_DIFFERENTIATION	7	-0.93893	0.4970297
Down Regulated Genes	GO_HISTONE_MRNA_METABOLIC_PROCESS	2	-0.93791	0.54651165
Down Regulated Genes	GO_PITUITARY_GLAND_DEVELOPMENT	1	-0.9378	0.60749507
Down Regulated Genes	GO_RECOMBINATIONAL_REPAIR	1	-0.93771	0.5840164
Down Regulated Genes	GO_LUNG_CELL_DIFFERENTIATION	2	-0.9375	0.5553398
Down Regulated Genes	GO_CYTOSOLIC_TRANSPORT	4	-0.93741	0.5118577
Down Regulated Genes	GO_NEURON_PROJECTION_EXTENSION	10	-0.93686	0.5123967

Down Regulated Genes	GO_REGULATION_OF_DOUBLE_STRAND_BREAK_REPAIR	1	-0.93666	0.5955734
Down Regulated Genes	GO_CYTOSKELETON_DEPENDENT_INTRACELLULAR_TRANSPORT	5	-0.93652	0.5511811
Down Regulated Genes	GO_STRIATED_MUSCLE_CELL_PROLIFERATION	3	-0.93589	0.53707415
Down Regulated Genes	GO_POSITIVE_REGULATION_OF_EPITHELIAL_CELL_PROLIFERATION	5	-0.93443	0.5380228
Down Regulated Genes	GO_REGULATION_OF_VASCULAR_PERMEABILITY	1	-0.93388	0.58365756
Down Regulated Genes	GO_POLYSACCHARIDE_BIOSYNTHETIC_PROCESS	3	-0.93325	0.5540275
Down Regulated Genes	GO_ARTERY_DEVELOPMENT	4	-0.93295	0.55226827
Down Regulated Genes	GO_REGULATION_OF_ESTABLISHMENT_OF_PROTEIN_LOCALIZATION_TO_M	2	-0.93188	0.5761719
Down Regulated Genes	GO_NEGATIVE_REGULATION_OF_PROTEIN_TYROSINE_KINASE_ACTIVITY	1	-0.93182	0.5936255
Down Regulated Genes	GO_LYMPHOCYTE_COSTIMULATION	1	-0.93092	0.5927419
Down Regulated Genes	GO_REGULATION_OF_POLYSACCHARIDE_METABOLIC_PROCESS	3	-0.93091	0.5576923
Down Regulated Genes	GO_EPIDERMAL_GROWTH_FACTOR_RECEPTOR_SIGNALING_PATHWAY	1	-0.9309	0.60728747
Down Regulated Genes	GO_NEGATIVE_REGULATION_OF_EPIDERMAL_GROWTH_FACTOR_ACTIVAT	1	-0.93049	0.59137577
Down Regulated Genes	GO_REGULATION_OF_DNA_RECOMBINATION	1	-0.93029	0.6104651
Down Regulated Genes	GO_NEGATIVE_REGULATION_OF_PEPTIDYL_TYROSINE_PHOSPHORYLATION	1	-0.92976	0.58536583
Down Regulated Genes	GO_VASCULAR_PROCESS_IN_CIRCULATORY_SYSTEM	1	-0.92967	0.60634327
Down Regulated Genes	GO_B_CELL_ACTIVATION	3	-0.92966	0.5323887
Down Regulated Genes	GO_NEGATIVE_REGULATION_OF_TRANSFERASE_ACTIVITY	1	-0.92875	0.6052104
Down Regulated Genes	GO_CELLULAR_PROTEIN_CATABOLIC_PROCESS	15	-0.92792	0.5051975
Down Regulated Genes	GO_NEGATIVE_REGULATION_OF_KINASE_ACTIVITY	1	-0.92755	0.59090906
Down Regulated Genes	GO_ORGANELLE_FUSION	3	-0.92678	0.5684411
Down Regulated Genes	GO_NEGATIVE_REGULATION_OF_SIGNALING_RECEPTOR_ACTIVITY	1	-0.92644	0.6191446
Down Regulated Genes	GO_MYD88_INDEPENDENT_TOLL_LIKE_RECEPTOR_SIGNALING_PATHWAY	2	-0.92585	0.56164384
Down Regulated Genes	GO_REGULATION_OF_EPIDERMAL_GROWTH_FACTOR_ACTIVATED_RECEPT	1	-0.92581	0.60081464
Down Regulated Genes	GO_DNA_REPAIR	5	-0.92575	0.53831774
Down Regulated Genes	GO_NEGATIVE_REGULATION_OF_OSSIFICATION	3	-0.92562	0.5653924
Down Regulated Genes	GO_CELLULAR_CARBOHYDRATE_METABOLIC_PROCESS	4	-0.92507	0.56448597
Down Regulated Genes	GO_COENZYME_A_BIOSYNTHETIC_PROCESS	1	-0.92406	0.5833333
Down Regulated Genes	GO_POLYSACCHARIDE_METABOLIC_PROCESS	3	-0.9235	0.56790125
Down Regulated Genes	GO_REGULATION_OF_PROTEIN_TARGETING_TO_MITOCHONDRION	2	-0.92317	0.54709417
Down Regulated Genes	GO_LUNG_EPITHELIUM_DEVELOPMENT	2	-0.92154	0.5793499
Down Regulated Genes	GO_RESPONSE_TO_PLATELET_DERIVED_GROWTH_FACTOR	1	-0.9209	0.61445785
Down Regulated Genes	GO_EPHRIN_RECEPTOR_SIGNALING_PATHWAY	3	-0.92052	0.5780933
Down Regulated Genes	GO_DEVELOPMENTAL_GROWTH_INVOLVED_IN_MORPHOGENESIS	11	-0.9205	0.53091687
Down Regulated Genes	GO_MYELOID_CELL_HOMEOSTASIS	2	-0.91899	0.59386975
Down Regulated Genes	GO_COENZYME_A_METABOLIC_PROCESS	1	-0.91871	0.6128405

Down Regulated Genes	GO_POSITIVE_REGULATION_OF_NERVOUS_SYSTEM_DEVELOPMENT	14	-0.91843	0.5383023
Down Regulated Genes	GO_CELLULAR_RESPIRATION	4	-0.91699	0.55289423
Down Regulated Genes	GO_MITOCHONDRIAL_RESPIRASOME_ASSEMBLY	1	-0.91591	0.601227
Down Regulated Genes	GO_POSITIVE_REGULATION_OF_MITOCHONDRION_ORGANIZATION	2	-0.91565	0.5730769
Down Regulated Genes	GO_POSITIVE_REGULATION_OF_EXOCYTOSIS	2	-0.9156	0.5854127
Down Regulated Genes	GO_DE_NOVO_PROTEIN_FOLDING	1	-0.91481	0.62151396
Down Regulated Genes	GO_NUCLEOSIDE_DIPHOSPHATE_METABOLIC_PROCESS	4	-0.91455	0.54347825
Down Regulated Genes	GO_NEGATIVE_REGULATION_OF_RELEASE_OF_CYTOCHROME_C_FROM_M	1	-0.91397	0.610101
Down Regulated Genes	GO_NUCLEOSIDE_BISPHOSPHATE_BIOSYNTHETIC_PROCESS	1	-0.91221	0.62736845
Down Regulated Genes	GO_POSITIVE_REGULATION_OF_MULTICELLULAR_ORGANISMAL_PROCESS	30	-0.91144	0.56973994
Down Regulated Genes	GO_POSITIVE_REGULATION_OF_REGULATED_SECRETORY_PATHWAY	2	-0.90875	0.5897436
Down Regulated Genes	GO_ARTERY_MORPHOGENESIS	4	-0.90835	0.56063616
Down Regulated Genes	GO_ATP_METABOLIC_PROCESS	4	-0.90515	0.5804481
Down Regulated Genes	GO_CELLULAR_RESPONSE_TO_STARVATION	2	-0.90453	0.5900383
Down Regulated Genes	GO_REGULATION_OF_MRNA_SPLICING_VIA_SPLICEOSOME	2	-0.90381	0.5839552
Down Regulated Genes	GO_REGULATION_OF_CHONDROCYTE_DIFFERENTIATION	5	-0.90314	0.5755102
Down Regulated Genes	GO_RELEASE_OF_CYTOCHROME_C_FROM_MITOCHONDRIA	1	-0.90279	0.63531667
Down Regulated Genes	GO_ACTIN_FILAMENT_BUNDLE_ORGANIZATION	4	-0.9024	0.5957895
Down Regulated Genes	GO_DEVELOPMENTAL_CELL_GROWTH	12	-0.90177	0.5602537
Down Regulated Genes	GO_PYRIMIDINE_NUCLEOTIDE_BIOSYNTHETIC_PROCESS	1	-0.90153	0.6225681
Down Regulated Genes	GO_CHAPERONE_MEDIATED_PROTEIN_FOLDING	1	-0.8999	0.6515151
Down Regulated Genes	GO_REGULATION_OF_RNA_SPLICING	2	-0.89982	0.6114519
Down Regulated Genes	GO_METAL_ION_HOMEOSTASIS	6	-0.89947	0.5644531
Down Regulated Genes	GO_CERAMIDE_BIOSYNTHETIC_PROCESS	2	-0.89895	0.60714287
Down Regulated Genes	GO_REGULATION_OF_CARTILAGE_DEVELOPMENT	5	-0.89892	0.6048387
Down Regulated Genes	GO_REGULATION_OF_CELLULAR_PROTEIN_CATABOLIC_PROCESS	6	-0.8982	0.5692308
Down Regulated Genes	GO_CELLULAR_RESPONSE_TO_EXTRACELLULAR_STIMULUS	2	-0.89819	0.60665363
Down Regulated Genes	GO_POSITIVE_REGULATION_OF_CELL_GROWTH	6	-0.89819	0.54562736
Down Regulated Genes	GO_NEGATIVE_REGULATION_OF_RNA_BIOSYNTHETIC_PROCESS	28	-0.89746	0.5903614
Down Regulated Genes	GO_REGULATION_OF_MRNA_PROCESSING	2	-0.89651	0.61410016
Down Regulated Genes	GO_MRNA_CLEAVAGE_INVOLVED_IN_MRNA_PROCESSING	1	-0.89409	0.6395349
Down Regulated Genes	GO_PROTEIN_MODIFICATION_BY_SMALL_PROTEIN_REMOVAL	5	-0.89406	0.563327
Down Regulated Genes	GO_MACROMOLECULE_DEACYLATION	2	-0.89402	0.6264591
Down Regulated Genes	GO_CELLULAR_RESPONSE_TO_GLUCOSE_STARVATION	2	-0.89392	0.62813103
Down Regulated Genes	GO_RESPONSE_TO_DIETARY_EXCESS	1	-0.8934	0.63692945
Down Regulated Genes	GO_CERAMIDE_METABOLIC_PROCESS	2	-0.89269	0.63580245

Down Regulated Genes	GO_REGULATION_OF_GENERATION_OF_PRECURSOR_METABOLITES_AND_	4	-0.89252	0.5891182
Down Regulated Genes	GO_CHONDROCYTE_DEVELOPMENT	3	-0.89242	0.6015936
Down Regulated Genes	GO_PYRIMIDINE_NUCLEOBASE_METABOLIC_PROCESS	1	-0.89235	0.6567164
Down Regulated Genes	GO_PROTEIN_DEPHOSPHORYLATION	8	-0.89151	0.5694165
Down Regulated Genes	GO_SMALL_MOLECULE_CATABOLIC_PROCESS	4	-0.89149	0.6199187
Down Regulated Genes	GO_PYRIMIDINE_RIBONUCLEOSIDE_MONOPHOSPHATE_METABOLIC_PROC	1	-0.89079	0.6757895
Down Regulated Genes	GO_POSITIVE_REGULATION_OF_CYTOKINE_PRODUCTION	6	-0.89055	0.61208576
Down Regulated Genes	GO_REGULATION_OF_BIOMINERALIZATION	5	-0.89048	0.5869981
Down Regulated Genes	GO_CHAPERONE_COFACTOR_DEPENDENT_PROTEIN_REFOLDING	1	-0.89015	0.6557377
Down Regulated Genes	GO_POSITIVE_REGULATION_OF_CHROMOSOME_ORGANIZATION	2	-0.89014	0.61228406
Down Regulated Genes	GO_PROTEIN_REFOLDING	1	-0.89001	0.6596558
Down Regulated Genes	GO_PURINE_NUCLEOTIDE_TRANSPORT	2	-0.8894	0.62035227
Down Regulated Genes	GO_NUCLEOBASE_CONTAINING_SMALL_MOLECULE_INTERCONVERSION	1	-0.88919	0.6621094
Down Regulated Genes	GO_ORGANOPHOSPHATE_CATABOLIC_PROCESS	3	-0.88895	0.59117085
Down Regulated Genes	GO_REGULATION_OF_TRIGLYCERIDE_METABOLIC_PROCESS	1	-0.88816	0.6461233
Down Regulated Genes	GO_REGULATION_OF_RELEASE_OF_CYTOCHROME_C_FROM_MITOCHONDI	1	-0.88795	0.67951316
Down Regulated Genes	GO_PYRIMIDINE_CONTAINING_COMPOUND_BIOSYNTHETIC_PROCESS	1	-0.88695	0.6619718
Down Regulated Genes	GO_AMEBOIDAL_TYPE_CELL_MIGRATION	7	-0.88636	0.576779
Down Regulated Genes	GO_NEGATIVE_REGULATION_OF_PEPTIDASE_ACTIVITY	3	-0.88617	0.6003717
Down Regulated Genes	GO_DNA_TEMPLATED_TRANSCRIPTION_TERMINATION	1	-0.88592	0.6760563
Down Regulated Genes	GO_PYRIMIDINE_NUCLEOSIDE_MONOPHOSPHATE_METABOLIC_PROCESS	1	-0.88575	0.6822034
Down Regulated Genes	GO_POSITIVE_REGULATION_OF_CELL_POPULATION_PROLIFERATION	16	-0.88574	0.5977528
Down Regulated Genes	GO_CIRCULATORY_SYSTEM_PROCESS	7	-0.88507	0.58019805
Down Regulated Genes	GO_MRNA_CLEAVAGE	1	-0.88486	0.6721992
Down Regulated Genes	GO_INNATE_IMMUNE_RESPONSE_ACTIVATING_SIGNAL_TRANSDUCTION	1	-0.88455	0.67906064
Down Regulated Genes	GO_PYRIMIDINE_NUCLEOBASE_BIOSYNTHETIC_PROCESS	1	-0.88451	0.67611337
Down Regulated Genes	GO_CELLULAR_RESPONSE_TO_EXTERNAL_STIMULUS	2	-0.88418	0.6397638
Down Regulated Genes	GO_DIVALENT_INORGANIC_CATION_HOMEOSTASIS	6	-0.88416	0.582
Down Regulated Genes	GO_PROTEIN_LOCALIZATION_TO_PLASMA_MEMBRANE	8	-0.88322	0.5717345
Down Regulated Genes	GO_NUCLEAR_TRANSPORT	10	-0.88229	0.60580915
Down Regulated Genes	GO_REGULATION_OF_TRANSCRIPTION_FROM_RNA_POLYMERASE_II_PROM	1	-0.88201	0.682
Down Regulated Genes	GO_NEUTRAL_LIPID_METABOLIC_PROCESS	1	-0.88179	0.65163934
Down Regulated Genes	GO_TRIGLYCERIDE_METABOLIC_PROCESS	1	-0.88039	0.6571429
Down Regulated Genes	GO_ATP_TRANSPORT	2	-0.8802	0.6443089
Down Regulated Genes	GO_MRNA_3_END_PROCESSING_BY_STEM_LOOP_BINDING_AND_CLEAVAC	1	-0.88012	0.6809816
Down Regulated Genes	GO_REGULATION_OF_DNA_METABOLIC_PROCESS	2	-0.8793	0.6204819

Down Regulated Genes	GO_DE_NOVO_PYRIMIDINE_NUCLEOBASE_BIOSYNTHETIC_PROCESS	1	-0.87911	0.69057375
Down Regulated Genes	GO_RIBONUCLEOSIDE_DIPHOSPHATE_BIOSYNTHETIC_PROCESS	2	-0.87897	0.63372093
Down Regulated Genes	GO_POSITIVE_REGULATION_OF_INNATE_IMMUNE_RESPONSE	1	-0.87814	0.67474747
Down Regulated Genes	GO_TERMINATION_OF_RNA_POLYMERASE_II_TRANSCRIPTION	1	-0.87806	0.6773547
Down Regulated Genes	GO_RESPONSE_TO_EXTRACELLULAR_STIMULUS	4	-0.87694	0.59607846
Down Regulated Genes	GO_HISTONE_DEACETYLATION	2	-0.87692	0.6332665
Down Regulated Genes	GO_BONE_MATURATION	2	-0.87688	0.63992536
Down Regulated Genes	GO_RESPONSE_TO_DRUG	7	-0.87673	0.5888031
Down Regulated Genes	GO_ACTIVATION_OF_INNATE_IMMUNE_RESPONSE	1	-0.87657	0.68
Down Regulated Genes	GO_RIBONUCLEOSIDE_MONOPHOSPHATE_METABOLIC_PROCESS	3	-0.87478	0.60227275
Down Regulated Genes	GO_NUCLEOTIDE_TRANSPORT	2	-0.87473	0.64136624
Down Regulated Genes	GO_BONE_MINERALIZATION_INVOLVED_IN_BONE_MATURATION	2	-0.87434	0.64299804
Down Regulated Genes	GO_PYRIMIDINE_RIBONUCLEOTIDE_METABOLIC_PROCESS	2	-0.87368	0.64
Down Regulated Genes	GO_ORGANISM_EMERGENCE_FROM_PROTECTIVE_STRUCTURE	1	-0.87335	0.6757895
Down Regulated Genes	GO_RESPONSE_TO_BACTERIUM	2	-0.8733	0.6374502
Down Regulated Genes	GO_PURINE_CONTAINING_COMPOUND_BIOSYNTHETIC_PROCESS	3	-0.87096	0.6333333
Down Regulated Genes	GO_POSITIVE_REGULATION_OF_RESPONSE_TO_BIOTIC_STIMULUS	1	-0.86967	0.7128099
Down Regulated Genes	GO_PROTEIN_STABILIZATION	3	-0.86858	0.6130268
Down Regulated Genes	GO_CARTILAGE_DEVELOPMENT	11	-0.86845	0.6055777
Down Regulated Genes	GO_FAT_PAD_DEVELOPMENT	1	-0.86845	0.7
Down Regulated Genes	GO_NERVOUS_SYSTEM_PROCESS	20	-0.86826	0.59903383
Down Regulated Genes	GO_NEGATIVE_REGULATION_OF_PROTEIN_MODIFICATION_BY_SMALL_PRO	1	-0.86826	0.697286
Down Regulated Genes	GO_REGULATION_OF_NERVOUS_SYSTEM_DEVELOPMENT	21	-0.86657	0.6293996
Down Regulated Genes	GO_REGULATION_OF_OXIDATIVE_STRESS_INDUCED_CELL_DEATH	1	-0.86506	0.6979592
Down Regulated Genes	GO_NUCLEOSIDE_DIPHOSPHATE_BIOSYNTHETIC_PROCESS	2	-0.86435	0.64534885
Down Regulated Genes	GO_B_CELL_PROLIFERATION	1	-0.86388	0.7148515
Down Regulated Genes	GO_POSITIVE_REGULATION_OF_RNA_BIOSYNTHETIC_PROCESS	31	-0.86379	0.6407767
Down Regulated Genes	GO_RESPONSE_TO_CARBOHYDRATE	3	-0.86362	0.6205534
Down Regulated Genes	GO_RESPONSE_TO_HYDROGEN_PEROXIDE	1	-0.86255	0.70726913
Down Regulated Genes	GO_POSITIVE_REGULATION_OF_SECRETION	4	-0.86235	0.6166008
Down Regulated Genes	GO_NUCLEOSIDE_MONOPHOSPHATE_METABOLIC_PROCESS	3	-0.86233	0.62330097
Down Regulated Genes	GO_REGULATION_OF_ANTIGEN_RECEPTOR_MEDIATED_SIGNALING_PATHW	1	-0.86214	0.7066116
Down Regulated Genes	GO_POSITIVE_REGULATION_OF_RESPONSE_TO_OXIDATIVE_STRESS	1	-0.86204	0.69539076
Down Regulated Genes	GO_POSITIVE_REGULATION_OF_OXIDATIVE_STRESS_INDUCED_CELL_DEATH	1	-0.8619	0.6982922
Down Regulated Genes	GO_POSITIVE_REGULATION_OF_HYDROGEN_PEROXIDE_INDUCED_CELL_DE	1	-0.86109	0.7037037
Down Regulated Genes	GO_NEGATIVE_REGULATION_OF_LEUKOCYTE_PROLIFERATION	1	-0.86095	0.7040169

Down Regulated Genes	GO_SENSORY_PERCEPTION_OF_LIGHT_STIMULUS	6	-0.8604	0.60305345
Down Regulated Genes	GO_PYRIMIDINE_NUCLEOTIDE_METABOLIC_PROCESS	2	-0.86018	0.64478767
Down Regulated Genes	GO_POSTSYNAPTIC_MODULATION_OF_CHEMICAL_SYNAPTIC_TRANSMISSIC	1	-0.85997	0.7063492
Down Regulated Genes	GO_NEGATIVE_REGULATION_OF_ANTIGEN_RECEPTOR_MEDIATED_SIGNAL	1	-0.85989	0.7017544
Down Regulated Genes	GO_POSITIVE_REGULATION_OF_CELL_AGING	1	-0.8592	0.6904762
Down Regulated Genes	GO_REGULATION_OF_B_CELL_PROLIFERATION	1	-0.85887	0.7191235
Down Regulated Genes	GO_CELL_DEATH_IN_RESPONSE_TO_HYDROGEN_PEROXIDE	1	-0.85869	0.69186044
Down Regulated Genes	GO_REGULATION_OF_T_CELL_RECEPTOR_SIGNALING_PATHWAY	1	-0.85833	0.719917
Down Regulated Genes	GO_CELLULAR_GLUCOSE_HOMEOSTASIS	3	-0.85812	0.63419485
Down Regulated Genes	GO_NEGATIVE_REGULATION_OF_T_CELL_RECEPTOR_SIGNALING_PATHWA\	1	-0.85802	0.70640177
Down Regulated Genes	GO_CELLULAR_RESPONSE_TO_CARBOHYDRATE_STIMULUS	3	-0.85782	0.6421268
Down Regulated Genes	GO_CELLULAR_RESPONSE_TO_HYDROGEN_PEROXIDE	1	-0.8573	0.7299578
Down Regulated Genes	GO_POSITIVE_REGULATION_OF_HYDROGEN_PEROXIDE_MEDIATED_PROGF	1	-0.85693	0.72
Down Regulated Genes	GO_ANDROGEN_RECEPTOR_SIGNALING_PATHWAY	1	-0.8569	0.7230483
Down Regulated Genes	GO_REGULATION_OF_B_CELL_ACTIVATION	1	-0.85667	0.7003891
Down Regulated Genes	GO_REGULATION_OF_RESPONSE_TO_REACTIVE_OXYGEN_SPECIES	1	-0.85626	0.6781609
Down Regulated Genes	GO_CATION_TRANSPORT	20	-0.85618	0.6107383
Down Regulated Genes	GO_MITOCHONDRIAL_GENE_EXPRESSION	2	-0.85575	0.6749522
Down Regulated Genes	GO_NEGATIVE_REGULATION_OF_T_CELL_PROLIFERATION	1	-0.85532	0.7008032
Down Regulated Genes	GO_NUCLEOSIDE_TRIPHOSPHATE_BIOSYNTHETIC_PROCESS	2	-0.85531	0.6767486
Down Regulated Genes	GO_POSITIVE_REGULATION_OF_CATALYTIC_ACTIVITY	19	-0.85513	0.6269663
Down Regulated Genes	GO_NEGATIVE_REGULATION_OF_B_CELL_PROLIFERATION	1	-0.85511	0.69274807
Down Regulated Genes	GO_NEGATIVE_REGULATION_OF_B_CELL_ACTIVATION	1	-0.85511	0.7222222
Down Regulated Genes	GO_RESPONSE_TO_MONOSACCHARIDE	3	-0.85507	0.65384614
Down Regulated Genes	GO_POSITIVE_REGULATION_OF_RESPONSE_TO_REACTIVE_OXYGEN_SPECIE	1	-0.85474	0.7234849
Down Regulated Genes	GO_POST_GOLGI_VESICLE_MEDIATED_TRANSPORT	3	-0.85401	0.6336449
Down Regulated Genes	GO_PROGRAMMED_CELL_DEATH_IN_RESPONSE_TO_REACTIVE_OXYGEN_S	1	-0.85392	0.7205589
Down Regulated Genes	GO_RESPONSE_TO_XENOBIOTIC_STIMULUS	1	-0.85316	0.72899157
Down Regulated Genes	GO_NUCLEOTIDE_PHOSPHORYLATION	3	-0.85212	0.67157894
Down Regulated Genes	GO_INTESTINAL_EPITHELIAL_CELL_DIFFERENTIATION	2	-0.85126	0.67178506
Down Regulated Genes	GO_NEGATIVE_REGULATION_OF_PHOSPHORYLATION	3	-0.85053	0.6796296
Down Regulated Genes	GO_POSITIVE_REGULATION_OF_ORGANELLE_ORGANIZATION	9	-0.85036	0.6084337
Down Regulated Genes	GO_REGULATION_OF_UBIQUITIN_PROTEIN_TRANSFERASE_ACTIVITY	1	-0.8493	0.7258979
Down Regulated Genes	GO_RESPONSE_TO_LEUKEMIA_INHIBITORY_FACTOR	2	-0.84885	0.6948819
Down Regulated Genes	GO_CELLULAR_RESPONSE_TO_REACTIVE_OXYGEN_SPECIES	1	-0.84795	0.7211155
Down Regulated Genes	GO_PYRIMIDINE_CONTAINING_COMPOUND_METABOLIC_PROCESS	2	-0.84786	0.66927594

Down Regulated Genes	GO_CELLULAR_RESPONSE_TO_DNA_DAMAGE_STIMULUS	9	-0.8474	0.6182573
Down Regulated Genes	GO_MODIFICATION_DEPENDENT_MACROMOLECULE_CATABOLIC_PROCESS	11	-0.84736	0.622
Down Regulated Genes	GO_PROTEIN_LOCALIZATION_TO_CELL_PERIPHERY	9	-0.84697	0.6137255
Down Regulated Genes	GO_NUCLEOSIDE_MONOPHOSPHATE_PHOSPHORYLATION	2	-0.84679	0.6693227
Down Regulated Genes	GO_CELL_DEATH_IN_RESPONSE_TO_OXIDATIVE_STRESS	1	-0.84643	0.75445545
Down Regulated Genes	GO_RNA_SPLICING_VIA_TRANSESTERIFICATION_REACTIONS	9	-0.84629	0.6047904
Down Regulated Genes	GO_CELL_MOTILITY	37	-0.84627	0.65736043
Down Regulated Genes	GO_CELL_AGING	4	-0.84501	0.650924
Down Regulated Genes	GO_XENOBIOTIC_CATABOLIC_PROCESS	1	-0.84395	0.7364017
Down Regulated Genes	GO_PROTEASOMAL_PROTEIN_CATABOLIC_PROCESS	11	-0.84375	0.60493827
Down Regulated Genes	GO_POSITIVE_REGULATION_OF_FILOPODIUM_ASSEMBLY	2	-0.8435	0.6841046
Down Regulated Genes	GO_REGULATION_OF_TRANSPORT	36	-0.84226	0.7067669
Down Regulated Genes	GO_RESPONSE_TO_REACTIVE_OXYGEN_SPECIES	1	-0.84202	0.73175544
Down Regulated Genes	GO_ENERGY_HOMEOSTASIS	1	-0.84058	0.7149123
Down Regulated Genes	GO_POSITIVE_REGULATION_OF_UBIQUITIN_PROTEIN_TRANSFERASE_ACTIV	1	-0.8393	0.73861384
Down Regulated Genes	GO_SERINE_FAMILY_AMINO_ACID_METABOLIC_PROCESS	2	-0.83924	0.69714284
Down Regulated Genes	GO_CYTOPLASMIC_PATTERN_RECOGNITION_RECEPTOR_SIGNALING_PATHV	1	-0.83823	0.74418604
Down Regulated Genes	GO_MORPHOGENESIS_OF_A_BRANCHING_STRUCTURE	6	-0.83797	0.63983905
Down Regulated Genes	GO_POSITIVE_REGULATION_OF_GLYCOGEN_METABOLIC_PROCESS	1	-0.8377	0.74950296
Down Regulated Genes	GO_NON_CANONICAL_WNT_SIGNALING_PATHWAY	4	-0.83701	0.6490566
Down Regulated Genes	GO_REGULATION_OF_CALCIUM_MEDIATED_SIGNALING	1	-0.83623	0.74556214
Down Regulated Genes	GO_NUCLEOTIDE_BINDING_DOMAIN_LEUCINE_RICH_REPEAT_CONTAINING	1	-0.83608	0.73293173
Down Regulated Genes	GO_PEPTIDYL_THREONINE_MODIFICATION	1	-0.83467	0.73306775
Down Regulated Genes	GO_RESPONSE_TO_TRANSFORMING_GROWTH_FACTOR_BETA	4	-0.83421	0.65794766
Down Regulated Genes	GO_REGULATION_OF_PATTERN_RECOGNITION_RECEPTOR_SIGNALING_PAT	1	-0.8338	0.74769795
Down Regulated Genes	GO_CHONDROCYTE_HYPERTROPHY	1	-0.83361	0.74
Down Regulated Genes	GO_REGULATION_OF_TUMOR_NECROSIS_FACTOR_MEDIATED_SIGNALING_	1	-0.83357	0.73833674
Down Regulated Genes	GO_REGULATION_OF_MITOCHONDRION_ORGANIZATION	6	-0.83357	0.6539924
Down Regulated Genes	GO_PYRUVATE_METABOLIC_PROCESS	3	-0.83313	0.6794355
Down Regulated Genes	GO_REGULATION_OF_TOLL_LIKE_RECEPTOR_SIGNALING_PATHWAY	1	-0.83311	0.7061144
Down Regulated Genes	GO_REGULATION_OF_LEUKOCYTE_MIGRATION	3	-0.83304	0.66464645
Down Regulated Genes	GO_REGULATION_OF_CALCINEURIN_MEDIATED_SIGNALING	1	-0.83296	0.7537155
Down Regulated Genes	GO_RESPONSE_TO_OXYGEN_LEVELS	8	-0.83281	0.64796907
Down Regulated Genes	GO_REGULATION_OF_CELL_DEVELOPMENT	21	-0.8318	0.6318182
Down Regulated Genes	GO_IN_UTERO_EMBRYONIC_DEVELOPMENT	7	-0.83101	0.6313646
Down Regulated Genes	GO_CELLULAR_RESPONSE_TO_VIRUS	1	-0.82977	0.7543186

Down Regulated Genes	GO_PROTEIN_NEDDYLATION	1	-0.82966	0.75697213
Down Regulated Genes	GO_MITOCHONDRIAL_FUSION	2	-0.82948	0.69979715
Down Regulated Genes	GO_NEGATIVE_REGULATION_OF_CALCIUM_MEDIATED_SIGNALING	1	-0.8294	0.74899596
Down Regulated Genes	GO_NEGATIVE_REGULATION_OF_CARBOHYDRATE_METABOLIC_PROCESS	3	-0.82924	0.66536206
Down Regulated Genes	GO_NEGATIVE_REGULATION_OF_CALCINEURIN_MEDIATED_SIGNALING	1	-0.82912	0.7565392
Down Regulated Genes	GO_T_CELL_MEDIATED_IMMUNITY	3	-0.82895	0.64615387
Down Regulated Genes	GO_REGULATION_OF_NUCLEOTIDE_BINDING_OLIGOMERIZATION_DOMAIN	1	-0.82769	0.7648262
Down Regulated Genes	GO_REGULATION_OF_VIRAL_INDUCED_CYTOPLASMIC_PATTERN_RECOGNI	1	-0.82764	0.7626775
Down Regulated Genes	GO_PROTEIN_DNA_COMPLEX_SUBUNIT_ORGANIZATION	4	-0.82751	0.6414048
Down Regulated Genes	GO_PROTEIN_TARGETING_TO_MEMBRANE	7	-0.82675	0.65767634
Down Regulated Genes	GO_POSITIVE_REGULATION_OF_LEUKOCYTE_MIGRATION	3	-0.8263	0.6716698
Down Regulated Genes	GO_RESPONSE_TO_WOUNDING	13	-0.82602	0.64908725
Down Regulated Genes	GO_POSITIVE_REGULATION_OF_UBIQUITIN_DEPENDENT_PROTEIN_CATAB(	3	-0.82585	0.67913383
Down Regulated Genes	GO_NEGATIVE_REGULATION_OF_PROTEIN_MODIFICATION_PROCESS	4	-0.82493	0.66994107
Down Regulated Genes	GO_CYTOPLASMIC_PATTERN_RECOGNITION_RECEPTOR_SIGNALING_PATH\	1	-0.82492	0.75051975
Down Regulated Genes	GO_REGULATION_OF_MITOCHONDRIAL_FUSION	2	-0.82463	0.72469634
Down Regulated Genes	GO_NEGATIVE_REGULATION_OF_MITOCHONDRION_ORGANIZATION	2	-0.8238	0.7077535
Down Regulated Genes	GO_HOMOCYSTEINE_METABOLIC_PROCESS	1	-0.82291	0.75303644
Down Regulated Genes	GO_POSITIVE_REGULATION_OF_GROWTH	7	-0.8227	0.6404959
Down Regulated Genes	GO_LYMPHOCYTE_MEDIATED_IMMUNITY	3	-0.82244	0.6946565
Down Regulated Genes	GO_SULFUR_AMINO_ACID_CATABOLIC_PROCESS	1	-0.82149	0.7514451
Down Regulated Genes	GO_GLIOGENESIS	9	-0.82138	0.64959013
Down Regulated Genes	GO_EMBRYO_DEVELOPMENT	24	-0.82137	0.6636971
Down Regulated Genes	GO_CELL_KILLING	3	-0.82036	0.6744639
Down Regulated Genes	GO_EPITHELIAL_CELL_MORPHOGENESIS	2	-0.81994	0.7078431
Down Regulated Genes	GO_REGULATION_OF_LEUKOCYTE_CHEMOTAXIS	3	-0.81913	0.6693387
Down Regulated Genes	GO_RRNA_TRANSPORT	1	-0.81894	0.7405941
Down Regulated Genes	GO_POSITIVE_REGULATION_OF_PROTEOLYSIS_INVOLVED_IN_CELLULAR_PI	3	-0.81883	0.7137331
Down Regulated Genes	GO_ORGANIC_CATION_TRANSPORT	2	-0.81841	0.7145709
Down Regulated Genes	GO_CELLULAR_RESPONSE_TO_RETINOIC_ACID	3	-0.81751	0.66202784
Down Regulated Genes	GO_OLIGODENDROCYTE_DIFFERENTIATION	4	-0.81731	0.66597074
Down Regulated Genes	GO_RIG_I_SIGNALING_PATHWAY	1	-0.81675	0.7962185
Down Regulated Genes	GO_POSITIVE_REGULATION_OF_LEUKOCYTE_CHEMOTAXIS	3	-0.81617	0.6863158
Down Regulated Genes	GO_NEURON_MIGRATION	5	-0.81608	0.6923077
Down Regulated Genes	GO_HOMOSERINE_METABOLIC_PROCESS	1	-0.81595	0.7777778
Down Regulated Genes	GO_SULFUR_AMINO_ACID_METABOLIC_PROCESS	1	-0.81532	0.7808219

Down Regulated Genes	GO_RESPIRATORY_ELECTRON_TRANSPORT_CHAIN	1	-0.81497	0.7728155
Down Regulated Genes	GO_PEPTIDYL_TYROSINE_MODIFICATION	9	-0.81468	0.66064256
Down Regulated Genes	GO_NEUROGENESIS	40	-0.81444	0.69727045
Down Regulated Genes	GO_ADAPTIVE_IMMUNE_RESPONSE_BASED_ON_SOMATIC_RECOMBINATIC	3	-0.81248	0.69454545
Down Regulated Genes	GO_CHOLINE_TRANSPORT	2	-0.81067	0.7096115
Down Regulated Genes	GO_MITOCHONDRIAL_TRANSMEMBRANE_TRANSPORT	1	-0.81012	0.77755904
Down Regulated Genes	GO_MITOCHONDRIAL_ELECTRON_TRANSPORT_NADH_TO_UBIQUINONE	1	-0.80997	0.77321815
Down Regulated Genes	GO_RNA_IMPORT_INTO_MITOCHONDRION	1	-0.80933	0.7881874
Down Regulated Genes	GO_PROTEIN_LOCALIZATION_TO_ORGANELLE	13	-0.80863	0.64605546
Down Regulated Genes	GO_NCRNA_PROCESSING	10	-0.80812	0.6474227
Down Regulated Genes	GO_OXIDATIVE_PHOSPHORYLATION	1	-0.8073	0.7948207
Down Regulated Genes	GO_CYSTEINE_METABOLIC_PROCESS	1	-0.80629	0.7976424
Down Regulated Genes	GO_ATP_SYNTHESIS_COUPLED_ELECTRON_TRANSPORT	1	-0.80615	0.7906067
Down Regulated Genes	GO_IMMATURE_B_CELL_DIFFERENTIATION	2	-0.80532	0.73203886
Down Regulated Genes	GO_RETINAL_GANGLION_CELL_AXON_GUIDANCE	2	-0.80445	0.7433962
Down Regulated Genes	GO_PROTEIN_K48_LINKED_UBIQUITINATION	2	-0.80375	0.7549801
Down Regulated Genes	GO_CORTICAL_ACTIN_CYTOSKELETON_ORGANIZATION	1	-0.8028	0.7815631
Down Regulated Genes	GO_REGULATION_OF_DEFENSE_RESPONSE_TO_VIRUS_BY_VIRUS	2	-0.80082	0.75970423
Down Regulated Genes	GO_REGULATION_OF_PEPTIDASE_ACTIVITY	4	-0.80014	0.6980769
Down Regulated Genes	GO_MORPHOGENESIS_OF_AN_EPITHELIUM	20	-0.79988	0.6860215
Down Regulated Genes	GO_ANTIGEN_PROCESSING_AND_PRESENTATION_OF_PEPTIDE_OR_POLYSA	2	-0.79761	0.73146296
Down Regulated Genes	GO_B_CELL_DIFFERENTIATION	2	-0.79747	0.7186312
Down Regulated Genes	GO_BIOMINERALIZATION	6	-0.79744	0.7057654
Down Regulated Genes	GO_CORTICAL_CYTOSKELETON_ORGANIZATION	1	-0.79731	0.8066406
Down Regulated Genes	GO_RESPIRATORY_SYSTEM_PROCESS	3	-0.79366	0.7131474
Down Regulated Genes	GO_NEGATIVE_REGULATION_OF_PYRUVATE_DEHYDROGENASE_ACTIVITY	1	-0.79336	0.8081633
Down Regulated Genes	GO_AUTOPHAGOSOME_ORGANIZATION	2	-0.79291	0.765625
Down Regulated Genes	GO_POSITIVE_REGULATION_OF_NEURON_PROJECTION_DEVELOPMENT	9	-0.79165	0.6799163
Down Regulated Genes	GO_DNA_RECOMBINATION	3	-0.79162	0.7151052
Down Regulated Genes	GO_RNA_CAPPING	1	-0.78973	0.8095238
Down Regulated Genes	GO_CHROMATIN_SILENCING_AT_TELOMERE	1	-0.78955	0.83938813
Down Regulated Genes	GO_INTERMEDIATE_FILAMENT_BASED_PROCESS	2	-0.78941	0.7436893
Down Regulated Genes	GO_POSITIVE_REGULATION_OF_CELLULAR_PROTEIN_CATABOLIC_PROCESS	4	-0.78869	0.6861167
Down Regulated Genes	GO_RESPONSE_TO_STARVATION	3	-0.78753	0.73956263
Down Regulated Genes	GO_SIGNAL_RELEASE	6	-0.78685	0.7031872
Down Regulated Genes	GO_PROTEIN_CATABOLIC_PROCESS	19	-0.78551	0.7066116

Down Regulated Genes	GO_BRANCHED_CHAIN_AMINO_ACID_METABOLIC_PROCESS	1	-0.78306	0.8360656
Down Regulated Genes	GO_ALPHA_AMINO_ACID_METABOLIC_PROCESS	4	-0.78266	0.7137255
Down Regulated Genes	GO_ESTABLISHMENT_OF_PROTEIN_LOCALIZATION_TO_MEMBRANE	8	-0.78032	0.71031743
Down Regulated Genes	GO_ION_HOMEOSTASIS	13	-0.77921	0.68869936
Down Regulated Genes	GO_RESPIRATORY_GASEOUS_EXCHANGE_BY_RESPIRATORY_SYSTEM	4	-0.77884	0.7128514
Down Regulated Genes	GO_NEGATIVE_REGULATION_OF_OXIDOREDUCTASE_ACTIVITY	1	-0.77662	0.8370221
Down Regulated Genes	GO_CHROMATIN_ASSEMBLY_OR_DISASSEMBLY	2	-0.7736	0.7673469
Down Regulated Genes	GO_CARBOHYDRATE_TRANSMEMBRANE_TRANSPORT	4	-0.77294	0.72746783
Down Regulated Genes	GO_REGULATION_OF_PYRUVATE_DEHYDROGENASE_ACTIVITY	1	-0.76995	0.83609957
Down Regulated Genes	GO_POSITIVE_REGULATION_OF_NUCLEOBASE_CONTAINING_COMPOUND_	33	-0.7697	0.75061727
Down Regulated Genes	GO_CELL_GROWTH	16	-0.76961	0.7319149
Down Regulated Genes	GO_ALCOHOL_METABOLIC_PROCESS	2	-0.76939	0.7790927
Down Regulated Genes	GO_HISTONE_MRNA_CATABOLIC_PROCESS	1	-0.76871	0.841785
Down Regulated Genes	GO_POLYOL_METABOLIC_PROCESS	2	-0.76777	0.76996195
Down Regulated Genes	GO_CHROMOSOME_ORGANIZATION	15	-0.76689	0.71161824
Down Regulated Genes	GO_SKELETAL_SYSTEM_MORPHOGENESIS	8	-0.76664	0.74846625
Down Regulated Genes	GO_EPITHELIAL_CELL_PROLIFERATION	8	-0.76653	0.74233127
Down Regulated Genes	GO_REGULATION_OF_GLUCOSE_TRANSMEMBRANE_TRANSPORT	4	-0.76644	0.7347328
Down Regulated Genes	GO_NUCLEOSIDE_MONOPHOSPHATE_BIOSYNTHETIC_PROCESS	2	-0.76582	0.79083663
Down Regulated Genes	GO_PROTEIN_N_LINKED_GLYCOSYLATION	4	-0.76392	0.75287354
Down Regulated Genes	GO_CELL_DIFFERENTIATION_INVOLVED_IN_KIDNEY_DEVELOPMENT	1	-0.76274	0.84023666
Down Regulated Genes	GO_PROTEIN_MODIFICATION_BY_SMALL_PROTEIN_CONJUGATION	18	-0.76273	0.71304345
Down Regulated Genes	GO_MYELOID_LEUKOCYTE_MIGRATION	4	-0.76262	0.749522
Down Regulated Genes	GO_EPITHELIAL_CELL_DIFFERENTIATION_INVOLVED_IN_KIDNEY_DEVELOPN	1	-0.7623	0.85856575
Down Regulated Genes	GO_SULFUR_COMPOUND_METABOLIC_PROCESS	7	-0.7617	0.722334
Down Regulated Genes	GO_RESPONSE_TO_MERCURY_ION	2	-0.76117	0.79926336
Down Regulated Genes	GO_NEURON_DIFFERENTIATION	36	-0.76092	0.7671233
Down Regulated Genes	GO_COLLAGEN_CATABOLIC_PROCESS	2	-0.76054	0.77955914
Down Regulated Genes	GO_SUBSTRATE_ADHESION_DEPENDENT_CELL_SPREADING	5	-0.7599	0.7444853
Down Regulated Genes	GO_NEGATIVE_REGULATION_OF_PROTEASOMAL_PROTEIN_CATABOLIC_PR	2	-0.75933	0.7941176
Down Regulated Genes	GO_PROTON_TRANSPORTING_TWO_SECTOR_ATPASE_COMPLEX_ASSEMBL	2	-0.75757	0.8034682
Down Regulated Genes	GO_NUCLEOBASE_METABOLIC_PROCESS	2	-0.75641	0.7885714
Down Regulated Genes	GO_RIBONUCLEOSIDE_MONOPHOSPHATE_BIOSYNTHETIC_PROCESS	2	-0.75608	0.80040324
Down Regulated Genes	GO_GLAND_DEVELOPMENT	13	-0.75591	0.7295082
Down Regulated Genes	GO_CARDIAC_CHAMBER_DEVELOPMENT	4	-0.75456	0.7708738
Down Regulated Genes	GO_REGULATION_OF_EPITHELIAL_CELL_DIFFERENTIATION_INVOLVED_IN_K	1	-0.75442	0.86939573

Down Regulated Genes	GO_POSITIVE_REGULATION_OF_CELLULAR_BIOSYNTHETIC_PROCESS	40	-0.75407	0.7945946
Down Regulated Genes	GO_CELLULAR_RESPONSE_TO_OXYGEN_CONTAINING_COMPOUND	14	-0.75405	0.7408257
Down Regulated Genes	GO_EMBRYO_DEVELOPMENT_ENDING_IN_BIRTH_OR_EGG_HATCHING	11	-0.75391	0.74207187
Down Regulated Genes	GO_ZINC_ION_TRANSPORT	1	-0.75038	0.8634538
Down Regulated Genes	GO_NUCLEOBASE_BIOSYNTHETIC_PROCESS	2	-0.74804	0.833652
Down Regulated Genes	GO_REGULATION_OF_THYROID_HORMONE_MEDIATED_SIGNALING_PATHV	1	-0.74796	0.8778468
Down Regulated Genes	GO_CELLULAR_TRANSITION_METAL_ION_HOMEOSTASIS	1	-0.74749	0.858
Down Regulated Genes	GO_NEGATIVE_REGULATION_OF_CELLULAR_PROTEIN_CATABOLIC_PROCES	2	-0.74706	0.80487806
Down Regulated Genes	GO_RIBONUCLEOPROTEIN_COMPLEX_BIOGENESIS	8	-0.74696	0.74583334
Down Regulated Genes	GO_TRANSITION_METAL_ION_HOMEOSTASIS	1	-0.74515	0.88594705
Down Regulated Genes	GO_REGULATION_OF_PROTON_TRANSPORT	1	-0.74475	0.87673956
Down Regulated Genes	GO_RESPONSE_TO_ZINC_ION	1	-0.74407	0.8602362
Down Regulated Genes	GO_REGULATION_OF_CHROMOSOME_ORGANIZATION	3	-0.74348	0.77756286
Down Regulated Genes	GO_SYNAPTIC_VESICLE_LOCALIZATION	3	-0.74255	0.7642436
Down Regulated Genes	GO_VESICLE_TARGETING	2	-0.74201	0.794971
Down Regulated Genes	GO_RESPONSE_TO_COLD	1	-0.74166	0.897541
Down Regulated Genes	GO_NEGATIVE_REGULATION_OF_INTRACELLULAR_PROTEIN_TRANSPORT	3	-0.74136	0.7946768
Down Regulated Genes	GO_POSITIVE_REGULATION_OF_TRANSCRIPTION_BY_RNA_POLYMERASE_II	23	-0.74113	0.751693
Down Regulated Genes	GO_SPHINGOLIPID_BIOSYNTHETIC_PROCESS	3	-0.741	0.78612715
Down Regulated Genes	GO_REGULATION_OF_TOOTH_MINERALIZATION	2	-0.74023	0.8255814
Down Regulated Genes	GO_COBALT_ION_TRANSPORT	1	-0.7394	0.8813187
Down Regulated Genes	GO_PROTEIN_MODIFICATION_BY_SMALL_PROTEIN_CONJUGATION_OR_RE	22	-0.73939	0.78444445
Down Regulated Genes	GO_POSITIVE_REGULATION_OF_ESTABLISHMENT_OF_PROTEIN_LOCALIZAT	1	-0.73817	0.8957055
Down Regulated Genes	GO_TOOTH_MINERALIZATION	2	-0.73789	0.79387754
Down Regulated Genes	GO_NEGATIVE_REGULATION_OF_TRANSCRIPTION_INITIATION_FROM_RNA	1	-0.73717	0.8822355
Down Regulated Genes	GO_POSITIVE_REGULATION_OF_PROTEIN_METABOLIC_PROCESS	25	-0.73574	0.78636366
Down Regulated Genes	GO_ZINC_ION_HOMEOSTASIS	1	-0.73554	0.8943089
Down Regulated Genes	GO_MATING	1	-0.73544	0.8964803
Down Regulated Genes	GO_NEGATIVE_REGULATION_OF_ESTABLISHMENT_OF_PROTEIN_LOCALIZA	3	-0.73543	0.77797836
Down Regulated Genes	GO_DOPAMINE_TRANSPORT	1	-0.73452	0.8815534
Down Regulated Genes	GO_REGULATION_OF_RNA_POLYMERASE_II_TRANSCRIPTION_PREINITIATIC	1	-0.7335	0.9116466
Down Regulated Genes	GO_REGULATION_OF_ODONTOGENESIS	2	-0.73298	0.8227612
Down Regulated Genes	GO_CELLULAR_AMINO_ACID_BIOSYNTHETIC_PROCESS	3	-0.73275	0.7659575
Down Regulated Genes	GO_STRESS_FIBER_ASSEMBLY	2	-0.73273	0.81963927
Down Regulated Genes	GO_REPRODUCTIVE_BEHAVIOR	1	-0.73251	0.8995984
Down Regulated Genes	GO_THYROID_GLAND_DEVELOPMENT	1	-0.73248	0.9005848

Down Regulated Genes	GO_POSITIVE_REGULATION_OF_TRANSCRIPTION_FROM_RNA_POLYMERAS	1	-0.73211	0.8990099
Down Regulated Genes	GO_POSITIVE_REGULATION_OF_NUCLEOCYTOPLASMIC_TRANSPORT	1	-0.73197	0.8996063
Down Regulated Genes	GO_POSITIVE_REGULATION_OF_TOOTH_MINERALIZATION	2	-0.73064	0.851272
Down Regulated Genes	GO_POSITIVE_REGULATION_OF_STRESS_FIBER_ASSEMBLY	2	-0.73025	0.8231827
Down Regulated Genes	GO_PHAGOSOME_LYSOSOME_FUSION	1	-0.72985	0.9023904
Down Regulated Genes	GO_REGULATION_OF_SYNAPTIC_VESICLE_EXOCYTOSIS	1	-0.72962	0.91
Down Regulated Genes	GO_REGULATION_OF_LIPID_CATABOLIC_PROCESS	1	-0.72951	0.89245284
Down Regulated Genes	GO_NEGATIVE_REGULATION_OF_DNA_TEMPLATED_TRANSCRIPTION_INITI/	1	-0.7294	0.8879837
Down Regulated Genes	GO_FEMALE_MATING_BEHAVIOR	1	-0.72938	0.9131274
Down Regulated Genes	GO_REGULATION_OF_GLUCAGON_SECRETION	1	-0.72926	0.8901961
Down Regulated Genes	GO_ORGANELLE_MEMBRANE_FUSION	1	-0.72925	0.8888889
Down Regulated Genes	GO_PEPTIDE_SECRETION	7	-0.72924	0.7692308
Down Regulated Genes	GO_TYPE_I_PNEUMOCYTE_DIFFERENTIATION	1	-0.72915	0.8794466
Down Regulated Genes	GO_CELLULAR_RESPONSE_TO_CADMIUM_ION	1	-0.7291	0.91198504
Down Regulated Genes	GO_REGULATION_OF_TRANSLATIONAL_INITIATION	1	-0.72896	0.9224319
Down Regulated Genes	GO_VESICLE_FUSION_TO_PLASMA_MEMBRANE	1	-0.72867	0.9037736
Down Regulated Genes	GO_PROTEIN_K11_LINKED_UBIQUITINATION	1	-0.7283	0.8875969
Down Regulated Genes	GO_CELLULAR_RESPONSE_TO_OXYGEN_LEVELS	3	-0.72785	0.8046875
Down Regulated Genes	GO_MYELOID_CELL_APOPTOTIC_PROCESS	1	-0.72784	0.902439
Down Regulated Genes	GO_POSITIVE_REGULATION_OF_CYTOSKELETON_ORGANIZATION	2	-0.72776	0.8154762
Down Regulated Genes	GO_RESPONSE_TO_CADMIUM_ION	1	-0.72776	0.9006342
Down Regulated Genes	GO_CATECHOLAMINE_SECRETION	1	-0.72713	0.9034908
Down Regulated Genes	GO_THYROID_HORMONE_MEDIATED_SIGNALING_PATHWAY	1	-0.72706	0.8815534
Down Regulated Genes	GO_PROTEIN_MONOUBIQUITINATION	1	-0.72697	0.9209486
Down Regulated Genes	GO_REGULATION_OF_ACTIN_FILAMENT_BUNDLE_ASSEMBLY	2	-0.72674	0.8497942
Down Regulated Genes	GO_MATING_BEHAVIOR	1	-0.72653	0.8974855
Down Regulated Genes	GO_CELLULAR_NITROGEN_COMPOUND_CATABOLIC_PROCESS	9	-0.72639	0.7565392
Down Regulated Genes	GO_POSITIVE_REGULATION_OF_ODONTOGENESIS	2	-0.7263	0.84540117
Down Regulated Genes	GO_ANATOMICAL_STRUCTURE_REGRESSION	1	-0.7254	0.9127907
Down Regulated Genes	GO_MONOAMINE_TRANSPORT	1	-0.72508	0.921371
Down Regulated Genes	GO_REGULATION_OF_CARBOHYDRATE_CATABOLIC_PROCESS	2	-0.72488	0.8509804
Down Regulated Genes	GO_CALCIUM_ION_REGULATED_EXOCYTOSIS_OF_NEUROTRANSMITTER	1	-0.72476	0.8942308
Down Regulated Genes	GO_POSITIVE_REGULATION_OF_ACTIN_FILAMENT_BUNDLE_ASSEMBLY	2	-0.72433	0.8336557
Down Regulated Genes	GO_POSITIVE_REGULATION_OF_NUCLEOBASE_CONTAINING_COMPOUND_	1	-0.72425	0.8893204
Down Regulated Genes	GO_POSITIVE_REGULATION_OF_SUPRAMOLECULAR_FIBER_ORGANIZATION	2	-0.72348	0.8317215
Down Regulated Genes	GO_REGULATION_OF_NEUROTRANSMITTER_TRANSPORT	1	-0.72278	0.9270833

Down Regulated Genes	GO_POSITIVE_REGULATION_OF_PROTEIN_TARGETING_TO_MITOCHONDRI(	1	-0.72252	0.89378756
Down Regulated Genes	GO_ENDOPLASMIC_RETICULUM_ORGANIZATION	6	-0.72196	0.7917485
Down Regulated Genes	GO_POSITIVE_REGULATION_OF_NEUROTRANSMITTER_SECRETION	1	-0.72162	0.9257028
Down Regulated Genes	GO_REGULATION_OF_DOPAMINE_SECRETION	1	-0.72104	0.90588236
Down Regulated Genes	GO_REGULATION_OF_ACTOMYOSIN_STRUCTURE_ORGANIZATION	2	-0.72085	0.8316633
Down Regulated Genes	GO_REGULATION_OF_NUCLEOBASE_CONTAINING_COMPOUND_TRANSPOI	1	-0.72069	0.9025845
Down Regulated Genes	GO_POSITIVE_REGULATION_OF_VESICLE_FUSION	1	-0.71996	0.9284294
Down Regulated Genes	GO_EXOCYTIC_PROCESS	1	-0.71974	0.9137931
Down Regulated Genes	GO_MACROAUTOPHAGY	4	-0.71943	0.807767
Down Regulated Genes	GO_PROSTATE_GLANDULAR_ACINUS_DEVELOPMENT	1	-0.71862	0.93167704
Down Regulated Genes	GO_DEPHOSPHORYLATION	10	-0.71817	0.771134
Down Regulated Genes	GO_PROTEIN_AUTOUBIQUITINATION	1	-0.71795	0.93050194
Down Regulated Genes	GO_ALPHA_AMINO_ACID_BIOSYNTHETIC_PROCESS	3	-0.71759	0.8170974
Down Regulated Genes	GO_REGULATION_OF_VESICLE_FUSION	1	-0.71697	0.91762453
Down Regulated Genes	GO_CARDIAC_CHAMBER_MORPHOGENESIS	4	-0.71689	0.78968257
Down Regulated Genes	GO_POSITIVE_REGULATION_OF_SYNAPTIC_VESICLE_EXOCYTOSIS	1	-0.71667	0.9195171
Down Regulated Genes	GO_POSITIVE_REGULATION_OF_NEURON_DIFFERENTIATION	11	-0.7166	0.78781927
Down Regulated Genes	GO_REGULATION_OF_RNA_EXPORT_FROM_NUCLEUS	1	-0.71651	0.90192306
Down Regulated Genes	GO_APOPTOTIC_MITOCHONDRIAL_CHANGES	3	-0.71618	0.8098859
Down Regulated Genes	GO_NEGATIVE_REGULATION_OF_DEPHOSPHORYLATION	1	-0.71588	0.90120965
Down Regulated Genes	GO_REGULATION_OF_TELOMERE_MAINTENANCE	1	-0.71544	0.9166667
Down Regulated Genes	GO_FORMATION_OF_CYTOPLASMIC_TRANSLATION_INITIATION_COMPLEX	1	-0.71533	0.9315069
Down Regulated Genes	GO_POSITIVE_REGULATION_OF_NEUROTRANSMITTER_TRANSPORT	1	-0.71531	0.9221557
Down Regulated Genes	GO_POSITIVE_REGULATION_OF_CALCIUM_ION_DEPENDENT_EXOCYTOSIS	1	-0.71506	0.93004113
Down Regulated Genes	GO_ANTERIOR_POSTERIOR_AXIS_SPECIFICATION	1	-0.71494	0.9229209
Down Regulated Genes	GO_POSITIVE_REGULATION_OF_RNA_BINDING	1	-0.71439	0.93248945
Down Regulated Genes	GO_REGULATION_OF_SYNAPTIC_VESICLE_FUSION_TO_PRESYNAPTIC_ACTI\	1	-0.71374	0.92175573
Down Regulated Genes	GO_AMINE_METABOLIC_PROCESS	1	-0.71353	0.9432485
Down Regulated Genes	GO_PROTEIN_METHYLATION	4	-0.71349	0.80943024
Down Regulated Genes	GO_AMINE_TRANSPORT	1	-0.71345	0.9437984
Down Regulated Genes	GO_NEGATIVE_REGULATION_OF_TRANSLATIONAL_INITIATION	1	-0.71262	0.9265873
Down Regulated Genes	GO_GLANDULAR_EPITHELIAL_CELL_DIFFERENTIATION	1	-0.71209	0.92047715
Down Regulated Genes	GO_TRIPARTITE_REGIONAL_SUBDIVISION	1	-0.71208	0.9478079
Down Regulated Genes	GO_RETROGRADE_TRANSPORT_ENDOSOME_TO_GOLGI	2	-0.71193	0.8326996
Down Regulated Genes	GO_REGULATION_OF_DNA_BIOSYNTHETIC_PROCESS	1	-0.71163	0.9391635
Down Regulated Genes	GO_EPITHELIAL_CELL_DIFFERENTIATION_INVOLVED_IN_PROSTATE_GLAND_	1	-0.7116	0.9309665

Down Regulated Genes	GO_REGULATION_OF_RNA_BINDING	1	-0.7115	0.937751
Down Regulated Genes	GO_POSITIVE_REGULATION_OF_TELOMERE_MAINTENANCE_VIA_TELOMER	1	-0.71119	0.9411765
Down Regulated Genes	GO_PLASMA_MEMBRANE_REPAIR	1	-0.70955	0.91581106
Down Regulated Genes	GO_REGULATION_OF_REPRODUCTIVE_PROCESS	1	-0.70954	0.9295499
Down Regulated Genes	GO_PROSTATE_GLAND_DEVELOPMENT	1	-0.70944	0.93992245
Down Regulated Genes	GO_NEGATIVE_REGULATION_OF_EPITHELIAL_CELL_PROLIFERATION_INVOL	1	-0.70906	0.93333334
Down Regulated Genes	GO_POSITIVE_REGULATION_OF_CELL_DEVELOPMENT	11	-0.70895	0.76299375
Down Regulated Genes	GO_REGULATION_OF_ATP_METABOLIC_PROCESS	2	-0.70873	0.8170974
Down Regulated Genes	GO_AMINE_CATABOLIC_PROCESS	1	-0.70872	0.9292929
Down Regulated Genes	GO_EPITHELIAL_CELL_PROLIFERATION_INVOLVED_IN_PROSTATE_GLAND_D	1	-0.70852	0.9306358
Down Regulated Genes	GO_TELOMERE_MAINTENANCE_VIA_TELOMERE_LENGTHENING	1	-0.70837	0.92885375
Down Regulated Genes	GO_TRNA_5_LEADER_REMOVAL	1	-0.70811	0.9514563
Down Regulated Genes	GO_MUSCLE_CELL_PROLIFERATION	4	-0.70764	0.8034682
Down Regulated Genes	GO_AMMONIUM_ION_METABOLIC_PROCESS	1	-0.70672	0.9161677
Down Regulated Genes	GO_REGULATION_OF_MRNA_BINDING	1	-0.70663	0.9288618
Down Regulated Genes	GO_REGULATION_OF_NUCLEOTIDE_METABOLIC_PROCESS	2	-0.70648	0.86417323
Down Regulated Genes	GO_SPLICEOSOMAL_SNRNP_ASSEMBLY	1	-0.70606	0.92263055
Down Regulated Genes	GO_NEGATIVE_REGULATION_OF_TELOMERE_MAINTENANCE_VIA_TELOME	1	-0.70586	0.93877554
Down Regulated Genes	GO_CHOLINE_CATABOLIC_PROCESS	1	-0.705	0.937247
Down Regulated Genes	GO_CHOLINE_METABOLIC_PROCESS	1	-0.70444	0.9318182
Down Regulated Genes	GO_NEGATIVE_REGULATION_OF_CHROMOSOME_ORGANIZATION	1	-0.70431	0.94578314
Down Regulated Genes	GO_RESPONSE_TO_SALT	1	-0.70412	0.93373495
Down Regulated Genes	GO_NEGATIVE_REGULATION_OF_TELOMERE_MAINTENANCE	1	-0.70374	0.944
Down Regulated Genes	GO_POSITIVE_REGULATION_OF_DNA_METABOLIC_PROCESS	1	-0.70352	0.9517103
Down Regulated Genes	GO_RNA_DEPENDENT_DNA_BIOSYNTHETIC_PROCESS	1	-0.70272	0.9282787
Down Regulated Genes	GO_NEGATIVE_REGULATION_OF_REPRODUCTIVE_PROCESS	1	-0.70254	0.9535354
Down Regulated Genes	GO_CELLULAR_RESPONSE_TO_SODIUM_ARSENITE	1	-0.7024	0.92678225
Down Regulated Genes	GO_TRNA_5_END_PROCESSING	1	-0.7023	0.9432485
Down Regulated Genes	GO_RESPONSE_TO_ARSENIC_CONTAINING_SUBSTANCE	1	-0.70204	0.9375
Down Regulated Genes	GO_MESONEPHROS_DEVELOPMENT	3	-0.70162	0.8018868
Down Regulated Genes	GO_CELLULAR_RESPONSE_TO_SALT	1	-0.70158	0.9275654
Down Regulated Genes	GO_NEGATIVE_REGULATION_OF_DNA_BIOSYNTHETIC_PROCESS	1	-0.70152	0.9285714
Down Regulated Genes	GO_CYTOPLASM_ORGANIZATION	1	-0.70137	0.94692147
Down Regulated Genes	GO_LIPID_PHOSPHORYLATION	3	-0.70109	0.824
Down Regulated Genes	GO_CELLULAR_BIOGENIC_AMINE_METABOLIC_PROCESS	1	-0.70091	0.92156863
Down Regulated Genes	GO_TELOMERE_ORGANIZATION	1	-0.7006	0.94139886

Down Regulated Genes	GO_MITOCHONDRIAL_FISSION	2	-0.70005	0.90215266
Down Regulated Genes	GO_REGULATION_OF_TELOMERE_MAINTENANCE_VIA_TELOMERE_LENGTH	1	-0.6996	0.93686354
Down Regulated Genes	GO_EMBRYONIC_AXIS_SPECIFICATION	1	-0.69892	0.9479769
Down Regulated Genes	GO_CELLULAR_RESPONSE_TO_ARSENIC_CONTAINING_SUBSTANCE	1	-0.69884	0.94174755
Down Regulated Genes	GO_POSITIVE_REGULATION_OF_TELOMERE_MAINTENANCE	1	-0.69881	0.95757574
Down Regulated Genes	GO_RNA_5_END_PROCESSING	1	-0.69787	0.9322034
Down Regulated Genes	GO_NEGATIVE_REGULATION_OF_DNA_METABOLIC_PROCESS	1	-0.6977	0.9484536
Down Regulated Genes	GO_CELL_SURFACE_RECEPTOR_SIGNALING_PATHWAY_INVOLVED_IN_CELL_	10	-0.69753	0.7923387
Down Regulated Genes	GO_ANIMAL_ORGAN_MATURATION	3	-0.69679	0.8313953
Down Regulated Genes	GO_DNA_BIOSYNTHETIC_PROCESS	1	-0.69636	0.94163424
Down Regulated Genes	GO_NEGATIVE_REGULATION_OF_TELOMERE_MAINTENANCE_VIA_TELOME	1	-0.69607	0.95047617
Down Regulated Genes	GO_POSITIVE_REGULATION_OF_CELL_PROJECTION_ORGANIZATION	12	-0.69589	0.7814433
Down Regulated Genes	GO_AMYLOID_PRECURSOR_PROTEIN_METABOLIC_PROCESS	3	-0.69549	0.80798477
Down Regulated Genes	GO_REGULATION_OF_RESPONSE_TO_OXIDATIVE_STRESS	2	-0.69494	0.8649706
Down Regulated Genes	GO_POSITIVE_REGULATION_OF_CANONICAL_WNT_SIGNALING_PATHWAY	2	-0.6942	0.85252523
Down Regulated Genes	GO_PHOSPHATIDYLINOSITOL_PHOSPHORYLATION	3	-0.69409	0.83235866
Down Regulated Genes	GO_WNT_SIGNALING_PATHWAY_CALCIUM_MODULATING_PATHWAY	2	-0.69363	0.8585086
Down Regulated Genes	GO_HISTONE_H3_DEACETYLATION	1	-0.69264	0.9658444
Down Regulated Genes	GO_POSITIVE_REGULATION_OF_DNA_BIOSYNTHETIC_PROCESS	1	-0.69238	0.93907565
Down Regulated Genes	GO_RESPONSE_TO_SODIUM_ARSENITE	1	-0.69216	0.9559118
Down Regulated Genes	GO_POSITIVE_REGULATION_OF_WNT_SIGNALING_PATHWAY	2	-0.69083	0.8774319
Down Regulated Genes	GO_TRICARBOXYLIC_ACID_CYCLE	1	-0.68918	0.9564315
Down Regulated Genes	GO_RNA_PHOSPHODIESTER_BOND_HYDROLYSIS_ENDONUCLEOLYTIC	1	-0.68891	0.95841587
Down Regulated Genes	GO_AMYLOID_PRECURSOR_PROTEIN_BIOSYNTHETIC_PROCESS	3	-0.6886	0.8366142
Down Regulated Genes	GO_NEGATIVE_REGULATION_OF_CELLULAR_COMPONENT_MOVEMENT	4	-0.68834	0.8385214
Down Regulated Genes	GO_CELL_CELL_SIGNALING	25	-0.68801	0.81463414
Down Regulated Genes	GO_KIDNEY_EPITHELIUM_DEVELOPMENT	3	-0.6876	0.84393066
Down Regulated Genes	GO_ORGANELLE_FISSION	2	-0.68756	0.86282307
Down Regulated Genes	GO_ADIPOSE_TISSUE_DEVELOPMENT	2	-0.68711	0.86153847
Down Regulated Genes	GO_ADP_BIOSYNTHETIC_PROCESS	1	-0.68596	0.98031497
Down Regulated Genes	GO_CHONDROCYTE_DIFFERENTIATION	7	-0.68544	0.8313492
Down Regulated Genes	GO_REGULATION_OF_LIPID_KINASE_ACTIVITY	2	-0.6834	0.8822394
Down Regulated Genes	GO_ENDOSOMAL_TRANSPORT	6	-0.68337	0.8104839
Down Regulated Genes	GO_INTESTINAL_EPITHELIAL_CELL_DEVELOPMENT	1	-0.68306	0.96747965
Down Regulated Genes	GO_PYRIMIDINE_RIBONUCLEOSIDE_TRIPHOSPHATE_METABOLIC_PROCESS	1	-0.68271	0.97868216
Down Regulated Genes	GO_RESPONSE_TO_KETONE	2	-0.68127	0.8929889

Down Regulated Genes	GO_CELLULAR_RESPONSE_TO_TESTOSTERONE_STIMULUS	2	-0.68112	0.8804781
Down Regulated Genes	GO_DNA_DAMAGE_RESPONSE_DETECTION_OF_DNA_DAMAGE	1	-0.68083	0.975
Down Regulated Genes	GO_UTP_METABOLIC_PROCESS	1	-0.68046	0.97319585
Down Regulated Genes	GO_STRIATED_MUSCLE_CELL_DIFFERENTIATION	5	-0.67972	0.81142855
Down Regulated Genes	GO_LOCOMOTORY_BEHAVIOR	3	-0.67918	0.85714287
Down Regulated Genes	GO_POSITIVE_REGULATION_OF_HORMONE_SECRETION	2	-0.6783	0.86417323
Down Regulated Genes	GO_REGULATION_OF_SYNAPSE_STRUCTURE_OR_ACTIVITY	7	-0.67751	0.8565965
Down Regulated Genes	GO_RESPONSE_TO_TESTOSTERONE	2	-0.67722	0.8721374
Down Regulated Genes	GO_PRI_MIRNA_TRANSCRIPTION_BY_RNA_POLYMERASE_II	1	-0.67718	0.97455966
Down Regulated Genes	GO_SKELETAL_MUSCLE_SATELLITE_CELL_DIFFERENTIATION	1	-0.67702	0.98245615
Down Regulated Genes	GO_PYRIMIDINE_NUCLEOSIDE_TRIPHOSPHATE_METABOLIC_PROCESS	1	-0.6761	0.98210734
Down Regulated Genes	GO_POSITIVE_REGULATION_OF_PEPTIDE_HORMONE_SECRETION	2	-0.67528	0.8762279
Down Regulated Genes	GO_WHITE_FAT_CELL_DIFFERENTIATION	2	-0.67392	0.87795275
Down Regulated Genes	GO_GLUTAMINE_FAMILY_AMINO_ACID_METABOLIC_PROCESS	2	-0.67235	0.86293435
Down Regulated Genes	GO_TRANSLATIONAL_TERMINATION	1	-0.67215	0.98178136
Down Regulated Genes	GO_POSITIVE_REGULATION_OF_PEPTIDE_SECRETION	2	-0.67187	0.88622755
Down Regulated Genes	GO_MITOCHONDRIAL_TRANSLATION	1	-0.67168	0.9939148
Down Regulated Genes	GO_RESPONSE_TO_SELENIUM_ION	1	-0.67161	0.99215686
Down Regulated Genes	GO_POSITIVE_REGULATION_OF_PRI_MIRNA_TRANSCRIPTION_BY_RNA_POI	1	-0.67157	0.9589322
Down Regulated Genes	GO_SYNAPSE_ORGANIZATION	7	-0.67117	0.858
Down Regulated Genes	GO_SKELETAL_MUSCLE_TISSUE_REGENERATION	1	-0.6695	0.9768786
Down Regulated Genes	GO_MITOCHONDRIAL_TRANSLATIONAL_TERMINATION	1	-0.66872	0.9843137
Down Regulated Genes	GO_POSITIVE_REGULATION_OF_INSULIN_SECRETION	2	-0.66847	0.87712663
Down Regulated Genes	GO_NEPHRON_DEVELOPMENT	3	-0.66763	0.86534655
Down Regulated Genes	GO_COLUMNAR_CUBOIDAL_EPITHELIAL_CELL_DEVELOPMENT	1	-0.6674	0.9825243
Down Regulated Genes	GO_SKELETAL_MUSCLE_SATELLITE_CELL_ACTIVATION	1	-0.66594	0.9837067
Down Regulated Genes	GO_GOLGI_ORGANIZATION	3	-0.66558	0.8276515
Down Regulated Genes	GO_REGULATION_OF_CHROMATIN_ORGANIZATION	2	-0.66333	0.90457255
Down Regulated Genes	GO_INSULIN_SECRETION	5	-0.66293	0.84931505
Down Regulated Genes	GO_MYOTUBE_DIFFERENTIATION_INVOLVED_IN_SKELETAL_MUSCLE_REGE	1	-0.6626	0.98214287
Down Regulated Genes	GO_BONE_DEVELOPMENT	9	-0.66215	0.8293651
Down Regulated Genes	GO_CELLULAR_ION_HOMEOSTASIS	12	-0.66122	0.8080808
Down Regulated Genes	GO_CELLULAR_RESPONSE_TO_KETONE	2	-0.66117	0.9025341
Down Regulated Genes	GO_NEGATIVE_REGULATION_OF_NOTCH_SIGNALING_PATHWAY	4	-0.65992	0.8364312
Down Regulated Genes	GO_ION_TRANSPORT	27	-0.65829	0.8668224
Down Regulated Genes	GO_PEPTIDE_HORMONE_SECRETION	5	-0.65708	0.8460039

Down Regulated Genes	GO_REGULATION_OF_PEPTIDE_HORMONE_SECRETION	5	-0.65618	0.85546875
Down Regulated Genes	GO_GLUTAMINE_FAMILY_AMINO_ACID_BIOSYNTHETIC_PROCESS	2	-0.65392	0.9110672
Down Regulated Genes	GO_INORGANIC_ANION_TRANSPORT	4	-0.65109	0.85458165
Down Regulated Genes	GO_GASTRULATION_WITH_MOUTH_FORMING_SECOND	2	-0.6503	0.8976834
Down Regulated Genes	GO_REGULATION_OF_PROTEIN_CATABOLIC_PROCESS	10	-0.65009	0.83428574
Down Regulated Genes	GO_SMALL_GTPASE_MEDIATED_SIGNAL_TRANSDUCTION	12	-0.64718	0.8769932
Down Regulated Genes	GO_REGULATION_OF_PEPTIDE_SECRETION	5	-0.64655	0.84812623
Down Regulated Genes	GO_NEGATIVE_REGULATION_OF_SMOOTHENED_SIGNALING_PATHWAY	2	-0.64411	0.9077213
Down Regulated Genes	GO_REGULATION_OF_HORMONE_SECRETION	5	-0.64228	0.8780952
Down Regulated Genes	GO_REGULATION_OF_INSULIN_SECRETION	5	-0.64163	0.8548387
Down Regulated Genes	GO_RESPONSE_TO_OXIDATIVE_STRESS	7	-0.63924	0.84765625
Down Regulated Genes	GO_CELLULAR_SENESCENCE	3	-0.63868	0.8901961
Down Regulated Genes	GO_ENDOPLASMIC_RETICULUM_TUBULAR_NETWORK_ORGANIZATION	2	-0.6386	0.8982726
Down Regulated Genes	GO_POSITIVE_REGULATION_OF_MOLECULAR_FUNCTION	26	-0.63766	0.8340807
Down Regulated Genes	GO_HORMONE_TRANSPORT	5	-0.63518	0.8659794
Down Regulated Genes	GO_INTRINSIC_APOPTOTIC_SIGNALING_PATHWAY_IN_RESPONSE_TO_DNA	3	-0.63509	0.88973385
Down Regulated Genes	GO_INTRINSIC_APOPTOTIC_SIGNALING_PATHWAY_IN_RESPONSE_TO_DNA	3	-0.63239	0.8912621
Down Regulated Genes	GO_PROTEIN_FOLDING	4	-0.63226	0.88188976
Down Regulated Genes	GO_CELL_MIGRATION_INVOLVED_IN_GASTRULATION	2	-0.63218	0.9186992
Down Regulated Genes	GO_CRANIAL_SKELETAL_SYSTEM_DEVELOPMENT	3	-0.6308	0.88586956
Down Regulated Genes	GO_EXTRACELLULAR_VESICLE_BIOGENESIS	2	-0.62986	0.94011974
Down Regulated Genes	GO_ORGANIC_CYCLIC_COMPOUND_CATABOLIC_PROCESS	8	-0.62927	0.8622754
Down Regulated Genes	GO_RESPONSE_TO_PEPTIDE	6	-0.62884	0.875
Down Regulated Genes	GO_CHROMATIN_ORGANIZATION	13	-0.62875	0.863354
Down Regulated Genes	GO_CHEMICAL_HOMEOSTASIS_WITHIN_A_TISSUE	2	-0.62689	0.93385214
Down Regulated Genes	GO_POSITIVE_REGULATION_OF_GLYCOPROTEIN_METABOLIC_PROCESS	2	-0.62593	0.93135434
Down Regulated Genes	GO_RESPONSE_TO_GROWTH_FACTOR	13	-0.62201	0.8583878
Down Regulated Genes	GO_RESPONSE_TO_OXYGEN_CONTAINING_COMPOUND	18	-0.61918	0.87471527
Down Regulated Genes	GO_CARBOHYDRATE_CATABOLIC_PROCESS	4	-0.61843	0.8849903
Down Regulated Genes	GO_NEGATIVE_REGULATION_OF_TRANSPORT	5	-0.61358	0.87096775
Down Regulated Genes	GO_POSITIVE_REGULATION_OF_GLYCOPROTEIN_BIOSYNTHETIC_PROCESS	2	-0.60659	0.92843693
Down Regulated Genes	GO_REGULATION_OF_PROTEIN_LOCALIZATION	20	-0.60577	0.8710407
Down Regulated Genes	GO_CARBOHYDRATE_BIOSYNTHETIC_PROCESS	6	-0.60571	0.9054326
Down Regulated Genes	GO_REGULATION_OF_SMALL_GTPASE_MEDIATED_SIGNAL_TRANSDUCTION	10	-0.60547	0.9066937
Down Regulated Genes	GO_NEGATIVE_REGULATION_OF_INTRACELLULAR_TRANSPORT	4	-0.60393	0.8974855
Down Regulated Genes	GO_NEGATIVE_REGULATION_OF_PROTEOLYSIS	7	-0.60312	0.8910506

Down Regulated Genes	GO_DIGESTIVE_SYSTEM_DEVELOPMENT	7	-0.59446	0.9072165
Down Regulated Genes	GO_NEGATIVE_REGULATION_OF_CATABOLIC_PROCESS	4	-0.58988	0.9300971
Down Regulated Genes	GO_MONOSACCHARIDE_BIOSYNTHETIC_PROCESS	2	-0.5871	0.96380955
Down Regulated Genes	GO_CARDIAC_VENTRICLE_DEVELOPMENT	2	-0.58462	0.9583333
Down Regulated Genes	GO_REGULATION_OF_PROTEIN_STABILITY	4	-0.5833	0.9260628
Down Regulated Genes	GO_REGULATION_OF_INTRACELLULAR_PROTEIN_TRANSPORT	9	-0.58232	0.90361446
Down Regulated Genes	GO_REGULATION_OF_PEPTIDE_TRANSPORT	14	-0.58071	0.91268194
Down Regulated Genes	GO_VENTRICULAR_SEPTUM_MORPHOGENESIS	2	-0.5802	0.9389764
Down Regulated Genes	GO_NEGATIVE_REGULATION_OF_CELL_DEATH	12	-0.58014	0.92016804
Down Regulated Genes	GO_GLUCOSE_METABOLIC_PROCESS	5	-0.57971	0.9150943
Down Regulated Genes	GO_ORGANIC_ACID_TRANSPORT	3	-0.57541	0.9245283
Down Regulated Genes	GO_NEGATIVE_REGULATION_OF_BIOSYNTHETIC_PROCESS	37	-0.57248	0.9322034
Down Regulated Genes	GO_VENTRICULAR_SEPTUM_DEVELOPMENT	2	-0.57244	0.9765166
Down Regulated Genes	GO_RAS_PROTEIN_SIGNAL_TRANSDUCTION	8	-0.56749	0.9284294
Down Regulated Genes	GO_HEART_MORPHOGENESIS	5	-0.56709	0.9204082
Down Regulated Genes	GO_AMINO_ACID_TRANSPORT	3	-0.5644	0.94140625
Down Regulated Genes	GO_MUSCLE_CELL_DIFFERENTIATION	7	-0.56298	0.9325397
Down Regulated Genes	GO_PROTEIN_ACYLATION	4	-0.55328	0.92871284
Down Regulated Genes	GO_EMBRYONIC_DIGIT_MORPHOGENESIS	3	-0.55293	0.9666012
Down Regulated Genes	GO_SPHINGOLIPID_METABOLIC_PROCESS	5	-0.55261	0.94268775
Down Regulated Genes	GO_VACUOLAR_ACIDIFICATION	2	-0.54192	0.9841584
Down Regulated Genes	GO_ESTABLISHMENT_OF_PROTEIN_LOCALIZATION_TO_ORGANELLE	10	-0.54133	0.94855964
Down Regulated Genes	GO_LYMPHOCYTE_ACTIVATION_INVOLVED_IN_IMMUNE_RESPONSE	2	-0.54116	0.96477497
Down Regulated Genes	GO_GLUCOSE_IMPORT	3	-0.54032	0.95938104
Down Regulated Genes	GO_CELLULAR_HOMEOSTASIS	18	-0.53966	0.92410713
Down Regulated Genes	GO_RESPONSE_TO_CALCIUM_ION	5	-0.53913	0.9604743
Down Regulated Genes	GO_PEPTIDYL_AMINO_ACID_MODIFICATION	24	-0.53898	0.9330254
Down Regulated Genes	GO_REGULATION_OF_ORGAN_GROWTH	2	-0.53769	0.97843134
Down Regulated Genes	GO_ORGAN_GROWTH	2	-0.53695	0.97307694
Down Regulated Genes	GO_COMPLEMENT_DEPENDENT_CYTOTOXICITY	2	-0.53689	0.9861111
Down Regulated Genes	GO_T_CELL_ACTIVATION_INVOLVED_IN_IMMUNE_RESPONSE	2	-0.53675	0.96862745
Down Regulated Genes	GO_NUCLEAR_TRANSCRIBED_MRNA_CATABOLIC_PROCESS	4	-0.53562	0.962
Down Regulated Genes	GO_BRANCHING_INVOLVED_IN_BLOOD_VESSEL_MORPHOGENESIS	2	-0.53549	0.981378
Down Regulated Genes	GO_NEGATIVE_REGULATION_OF_PROTEIN_CATABOLIC_PROCESS	3	-0.5353	0.9665428
Down Regulated Genes	GO_REGULATION_OF_GLUCOSE_IMPORT	3	-0.53474	0.9688716
Down Regulated Genes	GO_NEGATIVE_REGULATION_OF_APOPTOTIC_SIGNALING_PATHWAY	4	-0.53421	0.96015936

Down Regulated Genes	GO_REGULATION_OF_CELLULAR_PROTEIN_LOCALIZATION	14	-0.53299	0.94860816
Down Regulated Genes	GO_INSULIN_RECEPTOR_SIGNALING_PATHWAY	3	-0.53238	0.9592593
Down Regulated Genes	GO_HEART_GROWTH	2	-0.53226	0.98479086
Down Regulated Genes	GO_NEGATIVE_REGULATION_OF_INTRACELLULAR_SIGNAL_TRANSDUCTION	4	-0.53135	0.95137423
Down Regulated Genes	GO_PROTEIN_LOCALIZATION_TO_MEMBRANE	17	-0.5305	0.95723015
Down Regulated Genes	GO_REGULATION_OF_CELLULAR_RESPONSE_TO_STRESS	13	-0.53011	0.9452954
Down Regulated Genes	GO_IMMUNE_SYSTEM_DEVELOPMENT	16	-0.5291	0.93186814
Down Regulated Genes	GO_CARDIAC_MUSCLE_CELL_PROLIFERATION	2	-0.52893	0.9836066
Down Regulated Genes	GO_NEGATIVE_REGULATION_OF_CELLULAR_CATABOLIC_PROCESS	3	-0.52734	0.9769674
Down Regulated Genes	GO_REGULATION_OF_MUSCLE_ORGAN_DEVELOPMENT	2	-0.52705	0.98058254
Down Regulated Genes	GO_REGULATION_OF_HEART_GROWTH	2	-0.52692	0.97669905
Down Regulated Genes	GO_RESPONSE_TO_RETINOIC_ACID	4	-0.52631	0.9685658
Down Regulated Genes	GO_PH_REDUCTION	2	-0.52448	0.98214287
Down Regulated Genes	GO_CANONICAL_WNT_SIGNALING_PATHWAY	4	-0.52396	0.97
Down Regulated Genes	GO_REGULATION_OF_CARDIAC_MUSCLE_TISSUE_DEVELOPMENT	2	-0.52348	0.9903475
Down Regulated Genes	GO_CARBOHYDRATE_DERIVATIVE_CATABOLIC_PROCESS	3	-0.5104	0.9582505
Down Regulated Genes	GO_ODONTOGENESIS	4	-0.5068	0.96037734
Down Regulated Genes	GO_CELL_SUBSTRATE_ADHESION	8	-0.50392	0.9534413
Down Regulated Genes	GO_INTRACELLULAR_PROTEIN_TRANSPORT	37	-0.50216	0.9727723
Down Regulated Genes	GO_REGULATION_OF_INTRACELLULAR_TRANSPORT	11	-0.50007	0.9559748
Down Regulated Genes	GO_CELLULAR_MACROMOLECULE_CATABOLIC_PROCESS	21	-0.49983	0.93846154
Down Regulated Genes	GO_NEGATIVE_REGULATION_OF_INTRINSIC_APOPTOTIC_SIGNALING_PATH	3	-0.48067	0.9903101
Down Regulated Genes	GO_ANATOMICAL_STRUCTURE_HOMEOSTASIS	4	-0.47408	0.9844961
Down Regulated Genes	GO_SYNAPTIC_SIGNALING	14	-0.47109	0.9681529
Down Regulated Genes	GO_HISTONE_METHYLATION	3	-0.47089	0.9882583
Down Regulated Genes	GO_NEGATIVE_REGULATION_OF_CELLULAR_PROTEIN_LOCALIZATION	5	-0.44902	0.9842209
Down Regulated Genes	GO_TISSUE_HOMEOSTASIS	3	-0.44207	0.9882353
Down Regulated Genes	GO_CELLULAR_MACROMOLECULE_LOCALIZATION	47	-0.40236	0.992228
Down Regulated Genes	GO_SKELETAL_SYSTEM_DEVELOPMENT	17	-0.39284	0.99784017
Down Regulated Genes	GO_INTRACELLULAR_TRANSPORT	52	-0.34314	1
Upregulated Genes	GO_POSITIVE_REGULATION_OF_VIRAL_PROCESS	4	1.699167	0.027027028
Upregulated Genes	GO_REGULATION_OF_ANATOMICAL_STRUCTURE_SIZE	9	1.628052	0.050200805
Upregulated Genes	GO_GLAND_MORPHOGENESIS	4	1.60674	0.07068607
Upregulated Genes	GO_PROTEOGLYCAN_BIOSYNTHETIC_PROCESS	3	1.594316	0.044145875
Upregulated Genes	GO_NEGATIVE_REGULATION_OF_DENDRITE_DEVELOPMENT	2	1.574589	0.007984032
Upregulated Genes	GO_INTERACTION_WITH_SYMBIONT	3	1.574208	0.082474224

Upregulated Genes	GO_MODULATION_BY_HOST_OF_SYMBIONT_PROCESS	3	1.57316	0.058212057
Upregulated Genes	GO_HOMOPHILIC_CELL_ADHESION_VIA_PLASMA_MEMBRANE_ADHESION_	6	1.570335	0.09746589
Upregulated Genes	GO_NEGATIVE_REGULATION_OF_NEURON_DIFFERENTIATION	6	1.555436	0.099609375
Upregulated Genes	GO_REGULATION_OF_VIRAL_TRANSCRIPTION	3	1.552373	0.07337526
Upregulated Genes	GO_REGENERATION	5	1.548567	0.09311741
Upregulated Genes	GO_MODULATION_OF_PROCESS_OF_OTHER_ORGANISM	3	1.547129	0.07302231
Upregulated Genes	GO_REGULATION_OF_CELLULAR_COMPONENT_SIZE	9	1.545932	0.08964144
Upregulated Genes	GO_INTEGRIN_MEDIATED_SIGNALING_PATHWAY	3	1.506891	0.09072581
Upregulated Genes	GO_POSITIVE_REGULATION_BY_HOST_OF_VIRAL_TRANSCRIPTION	2	1.505276	0.039915968
Upregulated Genes	GO_REGULATION_OF_CELL_SIZE	8	1.499959	0.12090164
Upregulated Genes	GO_POSITIVE_REGULATION_OF_VIRAL_TRANSCRIPTION	2	1.489737	0.060796645
Upregulated Genes	GO_MONOCARBOXYLIC_ACID_BIOSYNTHETIC_PROCESS	2	1.487869	0.039215688
Upregulated Genes	GO_NEGATIVE_REGULATION_OF_NEURON_PROJECTION_DEVELOPMENT	5	1.485945	0.12579957
Upregulated Genes	GO_REGULATION_OF_DENDRITE_DEVELOPMENT	4	1.475159	0.14226805
Upregulated Genes	GO_CELL_CELL_ADHESION_VIA_PLASMA_MEMBRANE_ADHESION_MOLECU	8	1.468557	0.12653062
Upregulated Genes	GO_PROTEOGLYCAN_METABOLIC_PROCESS	4	1.459938	0.14343435
Upregulated Genes	GO_REGULATION_OF_ATPASE_ACTIVITY	2	1.448793	0.07185629
Upregulated Genes	GO_CARDIOCYTE_DIFFERENTIATION	3	1.447255	0.12083333
Upregulated Genes	GO_POSITIVE_REGULATION_OF_CELL_ADHESION	7	1.446424	0.17768595
Upregulated Genes	GO_NEGATIVE_REGULATION_OF_PROTEIN_POLYMERIZATION	2	1.434896	0.08686869
Upregulated Genes	GO_MULTICELLULAR_ORGANISMAL_RESPONSE_TO_STRESS	3	1.431896	0.11222445
Upregulated Genes	GO_REGULATION_OF_EXTENT_OF_CELL_GROWTH	6	1.419269	0.17760618
Upregulated Genes	GO_NEURON_APOPTOTIC_PROCESS	3	1.417872	0.13742071
Upregulated Genes	GO_NEGATIVE_REGULATION_OF_CELL_MORPHOGENESIS_INVOLVED_IN_D	4	1.416275	0.14822134
Upregulated Genes	GO_NEGATIVE_REGULATION_OF_CYTOSKELETON_ORGANIZATION	2	1.415514	0.11359404
Upregulated Genes	GO_REGULATION_OF_PROTEIN_POLYMERIZATION	2	1.411788	0.09146342
Upregulated Genes	GO_NEURON_DEATH	3	1.408836	0.13473684
Upregulated Genes	GO_REGULATION_OF_COAGULATION	2	1.408046	0.11290322
Upregulated Genes	GO_LIPOSACCHARIDE_METABOLIC_PROCESS	3	1.405084	0.12970711
Upregulated Genes	GO_REGULATION_OF_VIRAL_LIFE_CYCLE	2	1.398393	0.10504202
Upregulated Genes	GO_POSITIVE_REGULATION_OF_BONE_MINERALIZATION	2	1.394934	0.11133603
Upregulated Genes	GO_NEGATIVE_REGULATION_OF_VIRAL_LIFE_CYCLE	2	1.393868	0.108510636
Upregulated Genes	GO_POTASSIUM_ION_TRANSPORT	4	1.393659	0.16155419
Upregulated Genes	GO_REGULATION_OF_CELL_MORPHOGENESIS_INVOLVED_IN_DIFFERENTIA	12	1.390657	0.17751479
Upregulated Genes	GO_CHONDROITIN_SULFATE_PROTEOGLYCAN_BIOSYNTHETIC_PROCESS	2	1.390483	0.12550607
Upregulated Genes	GO_CHONDROITIN_SULFATE_PROTEOGLYCAN_METABOLIC_PROCESS	2	1.389319	0.1194332

Upregulated Genes	GO_REGULATION_OF_SYMBIOTIC_PROCESS	6	1.388575	0.19537815
Upregulated Genes	GO_REGULATION_OF_DEFENSE_RESPONSE_TO_VIRUS	2	1.38803	0.1018711
Upregulated Genes	GO_NEGATIVE_REGULATION_OF_SUPRAMOLECULAR_FIBER_ORGANIZATIO	2	1.385763	0.119760476
Upregulated Genes	GO_POSITIVE_REGULATION_OF_CELL_CELL_ADHESION	6	1.385382	0.20808081
Upregulated Genes	GO_SODIUM_ION_TRANSMEMBRANE_TRANSPORT	2	1.384643	0.1030303
Upregulated Genes	GO_AMINOGLYCAN_BIOSYNTHETIC_PROCESS	6	1.379057	0.19762845
Upregulated Genes	GO_VIRAL_GENOME_REPLICATION	2	1.375791	0.12398374
Upregulated Genes	GO_NEGATIVE_REGULATION_OF_VIRAL_GENOME_REPLICATION	2	1.375639	0.12765957
Upregulated Genes	GO_POSITIVE_REGULATION_OF_ION_TRANSMEMBRANE_TRANSPORT	2	1.37491	0.10364683
Upregulated Genes	GO_LIVER_MORPHOGENESIS	2	1.374511	0.113490365
Upregulated Genes	GO_DERMATAN_SULFATE_PROTEOGLYCAN_METABOLIC_PROCESS	2	1.370272	0.11576846
Upregulated Genes	GO_REGULATION_OF_VIRAL_GENOME_REPLICATION	2	1.368562	0.1482966
Upregulated Genes	GO_NEGATIVE_REGULATION_OF_RESPONSE_TO_WOUNDING	2	1.365773	0.13598326
Upregulated Genes	GO_MONONUCLEAR_CELL_MIGRATION	2	1.365432	0.14285715
Upregulated Genes	GO_POSITIVE_REGULATION_OF_T_CELL_PROLIFERATION	2	1.36473	0.12627292
Upregulated Genes	GO_NEGATIVE_REGULATION_OF_CELL_PROJECTION_ORGANIZATION	6	1.364166	0.21259843
Upregulated Genes	GO_MEMBRANE_INVAGINATION	2	1.363753	0.12350598
Upregulated Genes	GO_BASEMENT_MEMBRANE_ORGANIZATION	3	1.361886	0.16198704
Upregulated Genes	GO_NEGATIVE_REGULATION_OF_VIRAL_PROCESS	2	1.361533	0.12421053
Upregulated Genes	GO_NEGATIVE_REGULATION_OF_LEUKOCYTE_DIFFERENTIATION	2	1.357749	0.14529915
Upregulated Genes	GO_RESPONSE_TO_AUDITORY_STIMULUS	2	1.357558	0.13656388
Upregulated Genes	GO_REGULATION_OF_EPITHELIAL_TO_MESENCHYMAL_TRANSITION	2	1.355359	0.1330724
Upregulated Genes	GO_ADRENAL_GLAND_DEVELOPMENT	2	1.35411	0.13026053
Upregulated Genes	GO_CELL_CELL_ADHESION	24	1.353046	0.14079422
Upregulated Genes	GO_EPITHELIAL_CELL_APOPTOTIC_PROCESS	2	1.349873	0.14615385
Upregulated Genes	GO_REGULATION_OF_MORPHOGENESIS_OF_A_BRANCHING_STRUCTURE	2	1.348467	0.13590264
Upregulated Genes	GO_RESPONSE_TO_TYPE_I_INTERFERON	2	1.347684	0.123173274
Upregulated Genes	GO_POSITIVE_REGULATION_OF_HEPATOCYTE_PROLIFERATION	2	1.344544	0.16561845
Upregulated Genes	GO_CARDIAC_CELL_DEVELOPMENT	2	1.344535	0.16032064
Upregulated Genes	GO_NEGATIVE_REGULATION_OF_HEMOPOIESIS	2	1.342689	0.146
Upregulated Genes	GO_EXTRACELLULAR_MATRIX_ASSEMBLY	3	1.3399	0.19565217
Upregulated Genes	GO_REGULATION_OF_EPITHELIAL_CELL_APOPTOTIC_PROCESS	2	1.339809	0.15071283
Upregulated Genes	GO_REGULATION_OF_RHO_PROTEIN_SIGNAL_TRANSDUCTION	2	1.33754	0.16565657
Upregulated Genes	GO_REGULATION_OF_AXONOGENESIS	9	1.334134	0.2235529
Upregulated Genes	GO_REGULATION_OF_MONONUCLEAR_CELL_MIGRATION	2	1.330305	0.17021276
Upregulated Genes	GO_SODIUM_ION_TRANSPORT	3	1.327394	0.1875

Upregulated Genes	GO_REGULATION_OF_HEPATOCYTE_PROLIFERATION	2	1.325474	0.16313559
Upregulated Genes	GO_CARDIAC_MUSCLE_CELL_DIFFERENTIATION	2	1.323926	0.1627907
Upregulated Genes	GO_PLATELET_DEGRANULATION	1	1.32362	0.008130081
Upregulated Genes	GO_MULTI_ORGANISM_LOCALIZATION	3	1.323013	0.19957536
Upregulated Genes	GO_POSITIVE_REGULATION_OF_VIRAL_LIFE_CYCLE	1	1.322929	0.04631579
Upregulated Genes	GO_REGULATION_OF_RECEPTOR_SIGNALING_PATHWAY_VIA_STAT	2	1.321737	0.15677966
Upregulated Genes	GO_POSITIVE_REGULATION_OF_LEUKOCYTE_PROLIFERATION	2	1.321097	0.1680498
Upregulated Genes	GO_RESPONSE_TO_DSRNA	1	1.320071	0.03088803
Upregulated Genes	GO_DEFENSE_RESPONSE_TO_VIRUS	6	1.319347	0.21765913
Upregulated Genes	GO_RESPIRATORY_BURST	2	1.317837	0.15873016
Upregulated Genes	GO_REGULATION_OF_RESPONSE_TO_BIOTIC_STIMULUS	4	1.315714	0.21129707
Upregulated Genes	GO_NEGATIVE_REGULATION_OF_PROTEIN_BINDING	3	1.315712	0.19832985
Upregulated Genes	GO_GLYCOLIPID_BIOSYNTHETIC_PROCESS	2	1.315168	0.17165668
Upregulated Genes	GO_CELLULAR_RESPONSE_TO_DSRNA	1	1.314565	0.049250536
Upregulated Genes	GO_REGULATION_BY_VIRUS_OF_VIRAL_PROTEIN_LEVELS_IN_HOST_CELL	1	1.314247	0.040899795
Upregulated Genes	GO_REGULATION_OF_PROTEIN_BINDING	3	1.308353	0.21729958
Upregulated Genes	GO_CELLULAR_RESPONSE_TO_EXOGENOUS_DSRNA	1	1.306419	0.040733196
Upregulated Genes	GO_NEGATIVE_REGULATION_OF_HELICASE_ACTIVITY	1	1.306031	0.036809817
Upregulated Genes	GO_REGULATION_OF_TRANSMEMBRANE_RECEPTOR_PROTEIN_SERINE_TH	3	1.304484	0.188
Upregulated Genes	GO_NEGATIVE_REGULATION_OF_RESPONSE_TO_EXTERNAL_STIMULUS	3	1.304395	0.19628099
Upregulated Genes	GO_POSITIVE_REGULATION_OF_VIRAL_GENOME_REPLICATION	1	1.302956	0.034816246
Upregulated Genes	GO_MONOVALENT_INORGANIC_CATION_TRANSPORT	7	1.300884	0.22903885
Upregulated Genes	GO_PREASSEMBLY_OF_GPI_ANCHOR_IN_ER_MEMBRANE	2	1.29966	0.17681728
Upregulated Genes	GO_NERVE_DEVELOPMENT	3	1.298724	0.19450317
Upregulated Genes	GO_NEGATIVE_REGULATION_OF_ATPASE_ACTIVITY	1	1.298559	0.055555556
Upregulated Genes	GO_EMBRYONIC_CAMERA_TYPE_EYE_MORPHOGENESIS	2	1.297185	0.18554688
Upregulated Genes	GO_GPI_ANCHOR_METABOLIC_PROCESS	2	1.296504	0.1724846
Upregulated Genes	GO_RECEPTOR_MEDIATED_ENDOCYTOSIS	8	1.294803	0.24603175
Upregulated Genes	GO_CYTOKINE_MEDIATED_SIGNALING_PATHWAY	8	1.293661	0.25984251
Upregulated Genes	GO_POSTSYNAPTIC_MEMBRANE_ASSEMBLY	1	1.29311	0.08076923
Upregulated Genes	GO_REGULATION_OF_LONG_TERM_SYNAPTIC_POTENTIATION	1	1.292989	0.054108217
Upregulated Genes	GO_REGULATION_OF_HELICASE_ACTIVITY	1	1.291106	0.046464648
Upregulated Genes	GO_HEPATICOBILIARY_SYSTEM_DEVELOPMENT	4	1.29091	0.22653061
Upregulated Genes	GO_POLY_A_PLUS_MRNA_EXPORT_FROM_NUCLEUS	1	1.289679	0.06097561
Upregulated Genes	GO_VOCALIZATION_BEHAVIOR	1	1.287217	0.078947365
Upregulated Genes	GO_AUTONOMIC_NERVOUS_SYSTEM_DEVELOPMENT	3	1.28708	0.20987654

Upregulated Genes	GO_RECEPTOR_SIGNALING_PATHWAY_VIA_STAT	2	1.286954	0.1783567
Upregulated Genes	GO_LONG_TERM_SYNAPTIC_POTENTIATION	1	1.286871	0.06538462
Upregulated Genes	GO_MEMBRANE_BIOGENESIS	1	1.284636	0.103869654
Upregulated Genes	GO_POSITIVE_REGULATION_OF_EXCITATORY_POSTSYNAPTIC_POTENTIAL	1	1.284202	0.07185629
Upregulated Genes	GO_AMINOGLYCAN_METABOLIC_PROCESS	7	1.283924	0.24193548
Upregulated Genes	GO_REGULATION_OF_SMAD_PROTEIN_SIGNAL_TRANSDUCTION	1	1.283728	0.0882353
Upregulated Genes	GO_POSTSYNAPSE_ASSEMBLY	1	1.282896	0.068136275
Upregulated Genes	GO_REGULATION_OF_PRESYNAPSE_ORGANIZATION	1	1.282354	0.079918034
Upregulated Genes	GO_NEURON_MATURATION	3	1.282168	0.22052401
Upregulated Genes	GO_POSITIVE_REGULATION_OF_NERVOUS_SYSTEM_PROCESS	1	1.281777	0.064386316
Upregulated Genes	GO_NEGATIVE_REGULATION_OF_SYNAPTIC_TRANSMISSION	1	1.281186	0.09795918
Upregulated Genes	GO_ISOPRENOID_METABOLIC_PROCESS	2	1.281084	0.20612244
Upregulated Genes	GO_INNER_EAR_MORPHOGENESIS	3	1.278155	0.20272905
Upregulated Genes	GO_REGULATION_OF_NMDA_RECEPTOR_ACTIVITY	1	1.277688	0.076612905
Upregulated Genes	GO_POSITIVE_REGULATION_OF_GLUTAMATE_RECEPTOR_SIGNALING_PATH	1	1.277025	0.079457365
Upregulated Genes	GO_REGULATION_OF_GLUTAMATE_RECEPTOR_SIGNALING_PATHWAY	1	1.276805	0.08092485
Upregulated Genes	GO_SYNAPTIC_VESICLE_CLUSTERING	1	1.275876	0.08171206
Upregulated Genes	GO_POSITIVE_REGULATION_OF_TRANSPORTER_ACTIVITY	1	1.275355	0.08668076
Upregulated Genes	GO_POSITIVE_REGULATION_OF_AMPA_RECEPTOR_ACTIVITY	1	1.275277	0.08196721
Upregulated Genes	GO_REGULATION_OF_AMPA_RECEPTOR_ACTIVITY	1	1.275261	0.092369474
Upregulated Genes	GO_TERPENOID_METABOLIC_PROCESS	2	1.275172	0.2106383
Upregulated Genes	GO_NEGATIVE_REGULATION_OF_NEURON_PROJECTION_REGENERATION	1	1.275149	0.0787234
Upregulated Genes	GO_POSITIVE_REGULATION_OF_CATION_CHANNEL_ACTIVITY	1	1.274058	0.092369474
Upregulated Genes	GO_POSITIVE_REGULATION_OF_GTPASE_ACTIVITY	5	1.27365	0.2345679
Upregulated Genes	GO_NEGATIVE_REGULATION_OF_AXONOGENESIS	3	1.273631	0.22698073
Upregulated Genes	GO_RESPONSE_TO_AXON_INJURY	1	1.273278	0.092337914
Upregulated Genes	GO_CELLULAR_RESPONSE_TO_NITROGEN_COMPOUND	6	1.272676	0.24740125
Upregulated Genes	GO_POSITIVE_REGULATION_OF_TRANSMEMBRANE_RECEPTOR_PROTEIN_S	1	1.272162	0.09561753
Upregulated Genes	GO_NEGATIVE_REGULATION_OF_SYNAPSE_ORGANIZATION	1	1.271969	0.08190476
Upregulated Genes	GO_CELLULAR_RESPONSE_TO_ORGANIC_CYCLIC_COMPOUND	6	1.271395	0.2494759
Upregulated Genes	GO_REGULATION_OF_SYNAPTIC_VESICLE_CLUSTERING	1	1.271155	0.06790123
Upregulated Genes	GO_T_CELL_RECEPTOR_SIGNALING_PATHWAY	3	1.27115	0.21544716
Upregulated Genes	GO_INTRASPECIES_INTERACTION_BETWEEN_ORGANISMS	1	1.270554	0.08695652
Upregulated Genes	GO_NEGATIVE_REGULATION_OF_NERVOUS_SYSTEM_PROCESS	1	1.26968	0.08471075
Upregulated Genes	GO_REGULATION_OF_DENDRITIC_SPINE_MORPHOGENESIS	1	1.269559	0.08146639
Upregulated Genes	GO_PRESYNAPSE_ORGANIZATION	1	1.269129	0.087318085

Upregulated Genes	GO_NEGATIVE_REGULATION_OF_DENDRITIC_SPINE_MORPHOGENESIS	1	1.268563	0.09467456
Upregulated Genes	GO_NEGATIVE_REGULATION_OF_EXCITATORY_POSTSYNAPTIC_POTENTIAL	1	1.268377	0.09741551
Upregulated Genes	GO_POSITIVE_REGULATION_OF_SMAD_PROTEIN_SIGNAL_TRANSDUCTION	1	1.267733	0.11899791
Upregulated Genes	GO_TERMINAL_BUTTON_ORGANIZATION	1	1.267122	0.09
Upregulated Genes	GO_REGULATION_OF_DENDRITIC_SPINE_DEVELOPMENT	1	1.26705	0.08588957
Upregulated Genes	GO_REGULATION_OF_PLATELET_ACTIVATION	1	1.266994	0.09072978
Upregulated Genes	GO_POSITIVE_REGULATION_OF_TRANSMEMBRANE_TRANSPORT	4	1.265655	0.23293173
Upregulated Genes	GO_NEGATIVE_REGULATION_OF_DENDRITIC_SPINE_DEVELOPMENT	1	1.265613	0.08266129
Upregulated Genes	GO_POSTSYNAPTIC_MEMBRANE_ORGANIZATION	1	1.265303	0.09561753
Upregulated Genes	GO_ASSOCIATIVE_LEARNING	1	1.2652	0.097087376
Upregulated Genes	GO_PRESYNAPTIC_MEMBRANE_ORGANIZATION	1	1.265008	0.09052631
Upregulated Genes	GO_EMBRYONIC_ORGAN_MORPHOGENESIS	5	1.2646	0.24532224
Upregulated Genes	GO_INTERMEMBRANE_LIPID_TRANSFER	1	1.264003	0.10358566
Upregulated Genes	GO_B_CELL_RECEPTOR_SIGNALING_PATHWAY	1	1.263775	0.091451295
Upregulated Genes	GO_INORGANIC_ION_IMPORT_ACROSS_PLASMA_MEMBRANE	1	1.262672	0.115226336
Upregulated Genes	GO_RRNA_MODIFICATION	1	1.26118	0.11
Upregulated Genes	GO_REGULATION_OF_NEURON_PROJECTION_REGENERATION	1	1.260938	0.11434109
Upregulated Genes	GO_IMPORT_ACROSS_PLASMA_MEMBRANE	1	1.260355	0.11042945
Upregulated Genes	GO_POSITIVE_REGULATION_OF_SIGNALING_RECEPTOR_ACTIVITY	1	1.259233	0.114
Upregulated Genes	GO_BILE_ACID_METABOLIC_PROCESS	1	1.258979	0.11500975
Upregulated Genes	GO_INHIBITORY_POSTSYNAPTIC_POTENTIAL	1	1.258595	0.098076925
Upregulated Genes	GO_NEURON_PROJECTION_REGENERATION	1	1.258397	0.12248996
Upregulated Genes	GO_SUPEROXIDE_ANION_GENERATION	1	1.258163	0.12820514
Upregulated Genes	GO_STEROID_BIOSYNTHETIC_PROCESS	1	1.257847	0.12394958
Upregulated Genes	GO_EAR_MORPHOGENESIS	3	1.25769	0.2058212
Upregulated Genes	GO_NEGATIVE_REGULATION_OF_NEURON_DEATH	2	1.256902	0.22874494
Upregulated Genes	GO_TISSUE_REGENERATION	3	1.256139	0.21841542
Upregulated Genes	GO_REGULATION_OF_IMMUNE_EFFECTOR_PROCESS	4	1.255517	0.24369748
Upregulated Genes	GO_REGULATION_OF_STEROID_BIOSYNTHETIC_PROCESS	1	1.255321	0.12804878
Upregulated Genes	GO_NEGATIVE_REGULATION_OF_DENDRITE_MORPHOGENESIS	1	1.254481	0.09475806
Upregulated Genes	GO_EMBRYONIC_EYE_MORPHOGENESIS	2	1.252013	0.2244898
Upregulated Genes	GO_REGULATION_OF_CELL_ACTIVATION	7	1.250376	0.26086956
Upregulated Genes	GO_EAR_DEVELOPMENT	3	1.249726	0.2398374
Upregulated Genes	GO_POSITIVE_REGULATION_OF_STEROID_BIOSYNTHETIC_PROCESS	1	1.249487	0.10195228
Upregulated Genes	GO_LIPID_OXIDATION	1	1.248633	0.116412215
Upregulated Genes	GO_POSITIVE_REGULATION_OF_LIPID_BIOSYNTHETIC_PROCESS	1	1.248196	0.13097712

Upregulated Genes	GO_STEROL_IMPORT	1	1.247974	0.11111111
Upregulated Genes	GO_SPROUTING_OF_INJURED_AXON	1	1.247672	0.11646587
Upregulated Genes	GO_ANTIGEN_PROCESSING_AND_PRESENTATION_OF_EXOGENOUS_PEPTID	1	1.247658	0.12033195
Upregulated Genes	GO_POSITIVE_REGULATION_OF_SYNAPTIC_VESICLE_CLUSTERING	1	1.24726	0.073068894
Upregulated Genes	GO_POSITIVE_REGULATION_OF_CHOLESTEROL_ESTERIFICATION	1	1.247239	0.12185687
Upregulated Genes	GO_IMPORT_INTO_CELL	1	1.247165	0.12151395
Upregulated Genes	GO_REGULATION_OF_BILE_ACID_BIOSYNTHETIC_PROCESS	1	1.247016	0.124497995
Upregulated Genes	GO_FATTY_ACID_OMEGA_OXIDATION	1	1.246446	0.09394572
Upregulated Genes	GO_CELL_REDOX_HOMEOSTASIS	1	1.246058	0.09583333
Upregulated Genes	GO_NEGATIVE_REGULATION_OF_NEURON_APOPTOTIC_PROCESS	2	1.245478	0.20987654
Upregulated Genes	GO_REGULATION_OF_CELLULAR_KETONE_METABOLIC_PROCESS	1	1.245162	0.12903225
Upregulated Genes	GO_EMBRYONIC_CAMERA_TYPE_EYE_DEVELOPMENT	2	1.244341	0.2248394
Upregulated Genes	GO_CHOLESTEROL_STORAGE	1	1.243389	0.12727273
Upregulated Genes	GO_BILE_ACID_BIOSYNTHETIC_PROCESS	1	1.241799	0.11456311
Upregulated Genes	GO_SODIUM_ION_IMPORT_ACROSS_PLASMA_MEMBRANE	1	1.241446	0.1252485
Upregulated Genes	GO_ENDODERM_DEVELOPMENT	2	1.240574	0.23340471
Upregulated Genes	GO_ENDODERMAL_CELL_DIFFERENTIATION	2	1.239716	0.21789883
Upregulated Genes	GO_REGULATION_OF_BILE_ACID_METABOLIC_PROCESS	1	1.239276	0.14176245
Upregulated Genes	GO_POSITIVE_REGULATION_OF_STEROID_METABOLIC_PROCESS	1	1.238869	0.12007874
Upregulated Genes	GO_SUPEROXIDE_METABOLIC_PROCESS	1	1.237818	0.12015504
Upregulated Genes	GO_ANTIGEN_PROCESSING_AND_PRESENTATION_OF_PEPTIDE_ANTIGEN_\	1	1.236862	0.15028901
Upregulated Genes	GO_CELLULAR_KETONE_METABOLIC_PROCESS	1	1.235194	0.15429688
Upregulated Genes	GO_ANTIGEN_RECEPTOR_MEDIATED_SIGNALING_PATHWAY	3	1.232742	0.24710424
Upregulated Genes	GO_NEGATIVE_REGULATION_OF_EPITHELIAL_TO_MESENCHYMAL_TRANSIT	1	1.232646	0.17038539
Upregulated Genes	GO_PROTEIN_HYDROXYLATION	3	1.232093	0.23968565
Upregulated Genes	GO_REGULATION_OF_POSTSYNAPTIC_MEMBRANE_POTENTIAL	2	1.231172	0.25263157
Upregulated Genes	GO_HORMONE_METABOLIC_PROCESS	1	1.229438	0.16935484
Upregulated Genes	GO_POSITIVE_REGULATION_OF_CELL_MORPHOGENESIS_INVOLVED_IN_DIF	5	1.228914	0.2774327
Upregulated Genes	GO_REGULATION_OF_THYROID_HORMONE_GENERATION	1	1.228085	0.15
Upregulated Genes	GO_ENDODERM_FORMATION	2	1.227595	0.2515213
Upregulated Genes	GO_REGULATION_OF_DENDRITE_MORPHOGENESIS	2	1.227593	0.24414062
Upregulated Genes	GO_RRNA_METHYLATION	1	1.226697	0.16188525
Upregulated Genes	GO_REGULATION_OF_T_CELL_DIFFERENTIATION	2	1.226336	0.25
Upregulated Genes	GO_REGULATION_OF_CORE_PROMOTER_BINDING	1	1.22577	0.16596639
Upregulated Genes	GO_REGULATION_OF_PLASMINOGEN_ACTIVATION	1	1.222385	0.16733871
Upregulated Genes	GO_MODULATION_OF_EXCITATORY_POSTSYNAPTIC_POTENTIAL	2	1.222079	0.22033899

Upregulated Genes	GO_STEROID_METABOLIC_PROCESS	4	1.218221	0.2616633
Upregulated Genes	GO_POSITIVE_REGULATION_OF_TRANSCRIPTION_REGULATORY_REGION_D	1	1.217531	0.16829745
Upregulated Genes	GO_CEREBRAL_CORTEX_RADIALLY_ORIENTED_CELL_MIGRATION	2	1.217015	0.25
Upregulated Genes	GO_CHEMICAL_SYNAPTIC_TRANSMISSION_POSTSYNAPTIC	2	1.216814	0.23809524
Upregulated Genes	GO_PROTEIN_ACETYLATION	1	1.216506	0.18609408
Upregulated Genes	GO_REGULATION_OF_HORMONE_METABOLIC_PROCESS	1	1.215864	0.16767676
Upregulated Genes	GO_TRANSLATIONAL_INITIATION	5	1.214399	0.26041666
Upregulated Genes	GO_POSITIVE_REGULATION_OF_TRANSCRIPTION_INITIATION_FROM_RNA_	1	1.213702	0.1812977
Upregulated Genes	GO_POSITIVE_REGULATION_OF_CORE_PROMOTER_BINDING	1	1.212673	0.1660079
Upregulated Genes	GO_POSITIVE_REGULATION_OF_DNA_TEMPLATED_TRANSCRIPTION_INITIA	1	1.211762	0.17473684
Upregulated Genes	GO_PROTEIN_K63_LINKED_UBIQUITINATION	1	1.211614	0.18087319
Upregulated Genes	GO_POSITIVE_REGULATION_OF_MRNA_METABOLIC_PROCESS	1	1.211601	0.15876289
Upregulated Genes	GO_BASEMENT_MEMBRANE_ASSEMBLY	2	1.210902	0.25298804
Upregulated Genes	GO_RNA_DESTABILIZATION	1	1.210883	0.18163672
Upregulated Genes	GO_REGULATION_OF_LYMPHOCYTE_DIFFERENTIATION	2	1.210547	0.24948876
Upregulated Genes	GO_POSITIVE_REGULATION_OF_DNA_BINDING	1	1.210178	0.16306484
Upregulated Genes	GO_POSITIVE_REGULATION_OF_HORMONE_METABOLIC_PROCESS	1	1.210022	0.1949807
Upregulated Genes	GO_ZYMOGEN_ACTIVATION	1	1.209996	0.18811882
Upregulated Genes	GO_DERMATAN_SULFATE_METABOLIC_PROCESS	1	1.209144	0.18867925
Upregulated Genes	GO_SULFATION	1	1.20863	0.20204082
Upregulated Genes	GO_RESPONSE_TO_THYROID_HORMONE	1	1.208565	0.19291338
Upregulated Genes	GO_PLASMINOGEN_ACTIVATION	1	1.208295	0.17864476
Upregulated Genes	GO_PHENOL_CONTAINING_COMPOUND_METABOLIC_PROCESS	1	1.208173	0.17208412
Upregulated Genes	GO_GLIAL_CELL_MIGRATION	2	1.207865	0.2246696
Upregulated Genes	GO_EXTRACELLULAR_STRUCTURE_ORGANIZATION	11	1.207311	0.28767124
Upregulated Genes	GO_TELENCEPHALON_GLIAL_CELL_MIGRATION	2	1.206218	0.24798387
Upregulated Genes	GO_COCHLEA_MORPHOGENESIS	1	1.206215	0.19556452
Upregulated Genes	GO_MITOCHONDRIAL_GENOME_MAINTENANCE	2	1.204656	0.2672414
Upregulated Genes	GO_DETECTION_OF_MECHANICAL_STIMULUS_INVOLVED_IN_SENSORY_PEI	1	1.204199	0.20440882
Upregulated Genes	GO_SYNAPTIC_TRANSMISSION_CHOLINERGIC	1	1.203352	0.21292776
Upregulated Genes	GO_REGULATION_OF_IMMUNE_SYSTEM_PROCESS	18	1.202529	0.28846154
Upregulated Genes	GO_POSITIVE_REGULATION_OF_MRNA_CATABOLIC_PROCESS	1	1.202101	0.18816067
Upregulated Genes	GO_REFLEX	1	1.201973	0.19315895
Upregulated Genes	GO_PEPTIDYL_LYSINE_ACETYLATION	1	1.201219	0.18737271
Upregulated Genes	GO_COCHLEA_DEVELOPMENT	1	1.200913	0.2019802
Upregulated Genes	GO_POSITIVE_REGULATION_OF_PROTEIN_MATURATION	1	1.199298	0.18711019

Upregulated Genes	GO_THYROID_HORMONE_METABOLIC_PROCESS	1	1.199173	0.2020202
Upregulated Genes	GO_PHOSPHATIDYLINOSITOL_BIOSYNTHETIC_PROCESS	5	1.198951	0.30923694
Upregulated Genes	GO_PROTEIN_SULFATION	1	1.198935	0.21917808
Upregulated Genes	GO_CEREBRAL_CORTEX_CELL_MIGRATION	2	1.198726	0.2735043
Upregulated Genes	GO_PHAGOCYTOSIS_RECOGNITION	1	1.198719	0.21627408
Upregulated Genes	GO_APOPTOTIC_CELL_CLEARANCE	1	1.197671	0.21458334
Upregulated Genes	GO_NEGATIVE_REGULATION_OF_REACTIVE_OXYGEN_SPECIES_METABOLIC_	1	1.197544	0.19144602
Upregulated Genes	GO_EYELID_DEVELOPMENT_IN_CAMERA_TYPE_EYE	1	1.196795	0.20717132
Upregulated Genes	GO_ORGANIC_HYDROXY_COMPOUND_METABOLIC_PROCESS	6	1.195975	0.30994153
Upregulated Genes	GO_POSITIVE_REGULATION_OF_NEURON_DEATH	1	1.195658	0.19805825
Upregulated Genes	GO_EMBRYONIC_CRANIAL_SKELETON_MORPHOGENESIS	1	1.1956	0.20396039
Upregulated Genes	GO_3_UTR_MEDIATED_MRNA_DESTABILIZATION	1	1.195121	0.20808081
Upregulated Genes	GO_POSITIVE_REGULATION_OF_SYNAPTIC_TRANSMISSION_GLUTAMATERG	2	1.194945	0.27935222
Upregulated Genes	GO_PROTEIN_O_LINKED_GLYCOSYLATION	3	1.192715	0.28857717
Upregulated Genes	GO_HEPATOCYTE_GROWTH_FACTOR_RECEPTOR_SIGNALING_PATHWAY	1	1.192712	0.20930232
Upregulated Genes	GO_MONOCARBOXYLIC_ACID_METABOLIC_PROCESS	8	1.192635	0.31322956
Upregulated Genes	GO_GLYCOPROTEIN_BIOSYNTHETIC_PROCESS	14	1.192485	0.30813953
Upregulated Genes	GO_CATION_TRANSMEMBRANE_TRANSPORT	12	1.192424	0.31809524
Upregulated Genes	GO_REGULATION_OF_ORGANELLE_ASSEMBLY	1	1.189571	0.2185567
Upregulated Genes	GO_CELLULAR_RESPONSE_TO_IRON_ION	1	1.189138	0.20958084
Upregulated Genes	GO_POSITIVE_REGULATION_OF_PLASMINOGEN_ACTIVATION	1	1.187583	0.20883535
Upregulated Genes	GO_POSITIVE_REGULATION_OF_NEURON_APOPTOTIC_PROCESS	1	1.187394	0.19193858
Upregulated Genes	GO_NEGATIVE_REGULATION_OF_HYDROLASE_ACTIVITY	6	1.186922	0.30846775
Upregulated Genes	GO_CRANIAL_NERVE_FORMATION	1	1.186868	0.20594059
Upregulated Genes	GO_RESPONSE_TO_LIGHT_STIMULUS	2	1.186775	0.2781186
Upregulated Genes	GO_REGULATION_OF_MORPHOGENESIS_OF_AN_EPITHELIUM	6	1.186063	0.29821074
Upregulated Genes	GO_REGULATION_OF_VACUOLE_ORGANIZATION	1	1.185606	0.23188406
Upregulated Genes	GO_OPTIC_CUP_MORPHOGENESIS_INVOLVED_IN_CAMERA_TYPE_EYE_DEV	1	1.185509	0.21428572
Upregulated Genes	GO_RESPONSE_TO_RADIATION	2	1.185032	0.27634194
Upregulated Genes	GO_POSITIVE_REGULATION_OF_VACUOLE_ORGANIZATION	1	1.184616	0.21861471
Upregulated Genes	GO_RESPONSE_TO_IRON_ION	1	1.184516	0.21899225
Upregulated Genes	GO_VISUAL_BEHAVIOR	2	1.184396	0.27402863
Upregulated Genes	GO_EMBRYONIC_CAMERA_TYPE_EYE_FORMATION	1	1.182532	0.20449898
Upregulated Genes	GO_POSITIVE_REGULATION_OF_CELL_ACTIVATION	4	1.181846	0.27027026
Upregulated Genes	GO_SINGLE_FERTILIZATION	1	1.181476	0.22981367
Upregulated Genes	GO_CHONDROITIN_SULFATE_BIOSYNTHETIC_PROCESS	1	1.180277	0.214

Upregulated Genes	GO_REGULATION_OF_LIPID_BIOSYNTHETIC_PROCESS	3	1.177461	0.29979035
Upregulated Genes	GO_REGULATION_OF_LEUKOCYTE_DIFFERENTIATION	4	1.176457	0.28163266
Upregulated Genes	GO_REACTIVE_OXYGEN_SPECIES_METABOLIC_PROCESS	3	1.176328	0.27710843
Upregulated Genes	GO_HEMIDESMOSOME_ASSEMBLY	2	1.175015	0.2801724
Upregulated Genes	GO_ELASTIC_FIBER_ASSEMBLY	1	1.173527	0.23651452
Upregulated Genes	GO_DENDRITE_DEVELOPMENT	7	1.173332	0.31643003
Upregulated Genes	GO_NEGATIVE_REGULATION_OF_TRANSCRIPTION_BY_COMPETITIVE_PROM	1	1.171408	0.23060344
Upregulated Genes	GO_REGULATION_OF_AUTOPHAGOSOME_ASSEMBLY	1	1.171289	0.22875817
Upregulated Genes	GO_NEGATIVE_REGULATION_OF_CELLULAR_COMPONENT_ORGANIZATION	13	1.170345	0.36245352
Upregulated Genes	GO_MUSCLE_FILAMENT_SLIDING	1	1.167978	0.23809524
Upregulated Genes	GO_POSITIVE_REGULATION_OF_AXONOGENESIS	3	1.167921	0.28600824
Upregulated Genes	GO_EXOCYTOSIS	20	1.166355	0.30912477
Upregulated Genes	GO_NEUROTROPHIN_SIGNALING_PATHWAY	1	1.166117	0.23895583
Upregulated Genes	GO_POSITIVE_REGULATION_OF_AUTOPHAGOSOME_ASSEMBLY	1	1.163308	0.25490198
Upregulated Genes	GO_INNATE_IMMUNE_RESPONSE	10	1.161561	0.33333334
Upregulated Genes	GO_POSITIVE_REGULATION_OF_ORGANELLE_ASSEMBLY	1	1.160894	0.2734375
Upregulated Genes	GO_NEUROTROPHIN_TRK_RECEPTOR_SIGNALING_PATHWAY	1	1.160465	0.25726143
Upregulated Genes	GO_PARASYMPATHETIC_NERVOUS_SYSTEM_DEVELOPMENT	2	1.15943	0.29122055
Upregulated Genes	GO_ENDOPLASMIC_RETICULUM_TO_GOLGI_VESICLE_MEDIATED_TRANSPO	4	1.157816	0.29253113
Upregulated Genes	GO_DRINKING_BEHAVIOR	1	1.156828	0.25159913
Upregulated Genes	GO_REGULATION_OF_BRANCHING_INVOLVED_IN_SALIVARY_GLAND_MORI	1	1.155074	0.26272914
Upregulated Genes	GO_NEGATIVE_REGULATION_OF_OSTEOCLAST_DIFFERENTIATION	1	1.154241	0.26937985
Upregulated Genes	GO_NEGATIVE_REGULATION_OF_AXON_EXTENSION	2	1.152256	0.31736526
Upregulated Genes	GO_POSITIVE_REGULATION_OF_AXON_EXTENSION	3	1.152111	0.31392932
Upregulated Genes	GO_GLOMERULUS_DEVELOPMENT	2	1.15094	0.30873787
Upregulated Genes	GO_ACTIVIN_RECEPTOR_SIGNALING_PATHWAY	1	1.15061	0.26915115
Upregulated Genes	GO_POSITIVE_REGULATION_OF_RECEPTOR_SIGNALING_PATHWAY_VIA_ST/	1	1.150114	0.2555332
Upregulated Genes	GO_POSITIVE_REGULATION_OF_LEUKOCYTE_CELL_CELL_ADHESION	4	1.149626	0.30674848
Upregulated Genes	GO_ACTIVATION_OF_GTPASE_ACTIVITY	3	1.149305	0.29862475
Upregulated Genes	GO_NEGATIVE_REGULATION_OF_MYELOID_CELL_DIFFERENTIATION	1	1.148414	0.25813007
Upregulated Genes	GO_CARDIAC_MUSCLE_TISSUE_DEVELOPMENT	4	1.148198	0.30040324
Upregulated Genes	GO_EXTRACELLULAR_MATRIX_CELL_SIGNALING	1	1.147747	0.26597938
Upregulated Genes	GO_BRANCHING_INVOLVED_IN_SALIVARY_GLAND_MORPHOGENESIS	1	1.144863	0.27579364
Upregulated Genes	GO_FATTY_ACID_METABOLIC_PROCESS	2	1.1447	0.32673267
Upregulated Genes	GO_SALIVARY_GLAND_DEVELOPMENT	1	1.141978	0.2821577
Upregulated Genes	GO_HISTONE_H3_K4_TRIMETHYLATION	1	1.141753	0.28282827

Upregulated Genes	GO_ENDOCYTIC_RECYCLING	2	1.14153	0.32738096
Upregulated Genes	GO_REGULATION_OF_MONOCYTE_CHEMOTAXIS	1	1.141101	0.29157668
Upregulated Genes	GO_HISTONE_H3_K4_MONOMETHYLATION	1	1.140647	0.29622266
Upregulated Genes	GO_ENDOCYTOSIS	20	1.139558	0.3216783
Upregulated Genes	GO_MEMBRANE_PROTEIN_PROTEOLYSIS	2	1.138997	0.32226562
Upregulated Genes	GO_BETA_CATENIN_TCF_COMPLEX_ASSEMBLY	1	1.137642	0.29489604
Upregulated Genes	GO_POSITIVE_REGULATION_OF_MONONUCLEAR_CELL_MIGRATION	1	1.137095	0.270751
Upregulated Genes	GO_NEURAL_CREST_CELL_DIFFERENTIATION	2	1.136939	0.35270542
Upregulated Genes	GO_PLASMA_MEMBRANE_PHOSPHOLIPID_SCRAMBLING	1	1.136908	0.29693487
Upregulated Genes	GO_NEGATIVE_REGULATION_OF_BMP_SIGNALING_PATHWAY	1	1.136606	0.30844793
Upregulated Genes	GO_NEGATIVE_REGULATION_OF_MYELOID_LEUKOCYTE_DIFFERENTIATION	1	1.136193	0.2848723
Upregulated Genes	GO_PEPTIDYL_PROLINE_MODIFICATION	1	1.135865	0.284
Upregulated Genes	GO_PEPTIDYL_LYSINE_TRIMETHYLATION	1	1.135852	0.2706131
Upregulated Genes	GO_NEGATIVE_REGULATION_OF_ACTIVIN_RECEPTOR_SIGNALING_PATHWA	1	1.135754	0.29803923
Upregulated Genes	GO_NEURAL_CREST_CELL_MIGRATION	2	1.1356	0.33190578
Upregulated Genes	GO_REGULATION_OF_BMP_SIGNALING_PATHWAY	1	1.135292	0.2937365
Upregulated Genes	GO_NEGATIVE_REGULATION_OF_ACTIN_FILAMENT_POLYMERIZATION	1	1.135289	0.28458497
Upregulated Genes	GO_INTERFERON_GAMMA_PRODUCTION	1	1.1348	0.29166666
Upregulated Genes	GO_OSTEOCLAST_DIFFERENTIATION	1	1.133701	0.30268198
Upregulated Genes	GO_EXOCRINE_SYSTEM_DEVELOPMENT	1	1.133046	0.28514057
Upregulated Genes	GO_PEPTIDYL_PROLINE_HYDROXYLATION_TO_4_HYDROXY_L_PROLINE	1	1.132574	0.29791668
Upregulated Genes	GO_TRIGEMINAL_NERVE_DEVELOPMENT	2	1.13253	0.3211382
Upregulated Genes	GO_RESPONSE_TO_CYTOKINE	11	1.131859	0.36761904
Upregulated Genes	GO_PEPTIDYL_PROLINE_HYDROXYLATION	1	1.130773	0.28657314
Upregulated Genes	GO_POSITIVE_REGULATION_OF_PEPTIDYL_TYROSINE_PHOSPHORYLATION	2	1.130541	0.35654008
Upregulated Genes	GO_REGULATION_OF_MICROTUBULE_POLYMERIZATION	1	1.130216	0.2930328
Upregulated Genes	GO_MULTICELLULAR_ORGANISMAL_SIGNALING	2	1.130189	0.35022026
Upregulated Genes	GO_HISTONE_H3_K4_DIMETHYLATION	1	1.130131	0.28803244
Upregulated Genes	GO_LEARNING	2	1.129796	0.32683983
Upregulated Genes	GO_MORPHOGENESIS_OF_A_POLARIZED_EPITHELIUM	5	1.129579	0.34303534
Upregulated Genes	GO_POSITIVE_REGULATION_OF_INTRACELLULAR_ESTROGEN_RECEPTOR_SI	1	1.129496	0.2964427
Upregulated Genes	GO_PEPTIDYL_LYSINE_DIMETHYLATION	1	1.12859	0.30095237
Upregulated Genes	GO_HEPARAN_SULFATE_PROTEOGLYCAN_METABOLIC_PROCESS	1	1.12696	0.327451
Upregulated Genes	GO_NEGATIVE_REGULATION_OF_MICROTUBULE_POLYMERIZATION_OR_DI	1	1.126715	0.31034482
Upregulated Genes	GO_NEURON_REMODELING	1	1.126527	0.2911111
Upregulated Genes	GO_REGULATION_OF_MICROTUBULE_NUCLEATION	1	1.125025	0.29333332

Upregulated Genes	GO_REGULATION_OF_INTRACELLULAR_ESTROGEN_RECEPTOR_SIGNALING_	1	1.124807	0.33401638
Upregulated Genes	GO_ANATOMICAL_STRUCTURE_ARRANGEMENT	2	1.124777	0.33604887
Upregulated Genes	GO_MRNA_METABOLIC_PROCESS	19	1.124542	0.3583815
Upregulated Genes	GO_REGULATION_OF_INTRACELLULAR_STEROID_HORMONE_RECEPTOR_SI	1	1.12338	0.31428573
Upregulated Genes	GO_POSITIVE_REGULATION_OF_INTRACELLULAR_STEROID_HORMONE_REC	1	1.123189	0.33268481
Upregulated Genes	GO_MICROTUBULE_POLYMERIZATION	1	1.123155	0.31262136
Upregulated Genes	GO_ACTIN_POLYMERIZATION_OR_DEPOLYMERIZATION	1	1.123003	0.31451613
Upregulated Genes	GO_POSITIVE_REGULATION_OF_INTERFERON_GAMMA_PRODUCTION	1	1.122792	0.34046692
Upregulated Genes	GO_POSITIVE_REGULATION_OF_COAGULATION	1	1.122083	0.33464566
Upregulated Genes	GO_POSITIVE_REGULATION_OF_EPITHELIAL_CELL_APOPTOTIC_PROCESS	1	1.121749	0.3225108
Upregulated Genes	GO_NEGATIVE_REGULATION_OF_CELL_VOLUME	1	1.121188	0.30705395
Upregulated Genes	GO_REGULATION_OF_ACTIVIN_RECEPTOR_SIGNALING_PATHWAY	1	1.120841	0.29807693
Upregulated Genes	GO_REGULATION_OF_OSTEOCLAST_DIFFERENTIATION	1	1.120139	0.32285115
Upregulated Genes	GO_HEPARAN_SULFATE_PROTEOGLYCAN_BIOSYNTHETIC_PROCESS	1	1.120053	0.30443975
Upregulated Genes	GO_PEPTIDYL_TYROSINE_AUTOPHOSPHORYLATION	2	1.119588	0.34655532
Upregulated Genes	GO_MICROTUBULE_NUCLEATION	1	1.119141	0.312749
Upregulated Genes	GO_CRANIAL_NERVE_MORPHOGENESIS	2	1.119079	0.3432836
Upregulated Genes	GO_REGULATION_OF_MEMBRANE_INVAGINATION	1	1.119074	0.32186234
Upregulated Genes	GO_POSITIVE_REGULATION_OF_PHAGOCYTOSIS_ENGULFMENT	1	1.118773	0.33333334
Upregulated Genes	GO_INTRACELLULAR_ESTROGEN_RECEPTOR_SIGNALING_PATHWAY	1	1.117701	0.30528376
Upregulated Genes	GO_REGULATION_OF_PROTEIN_CONTAINING_COMPLEX_ASSEMBLY	3	1.117637	0.3268817
Upregulated Genes	GO_MONOCYTE_CHEMOTAXIS	1	1.116596	0.32098764
Upregulated Genes	GO_FC_EPSILON_RECEPTOR_SIGNALING_PATHWAY	2	1.116324	0.32985386
Upregulated Genes	GO_CALCIUM_ACTIVATED_PHOSPHOLIPID_SCRAMBLING	1	1.115357	0.3184466
Upregulated Genes	GO_RESPONSE_TO_ESTROGEN	1	1.114424	0.31198347
Upregulated Genes	GO_POSITIVE_REGULATION_OF_ENDOTHELIAL_CELL_APOPTOTIC_PROCESS	1	1.114192	0.32700422
Upregulated Genes	GO_EXPORT_ACROSS_PLASMA_MEMBRANE	1	1.113991	0.30722892
Upregulated Genes	GO_CELL_VOLUME_HOMEOSTASIS	1	1.113975	0.32213438
Upregulated Genes	GO_NEGATIVE_REGULATION_OF_MICROTUBULE_POLYMERIZATION	1	1.113718	0.29545453
Upregulated Genes	GO_PROTEIN_EXIT_FROM_ENDOPLASMIC_RETICULUM	5	1.11294	0.3577075
Upregulated Genes	GO_POSITIVE_REGULATION_OF_MONOCYTE_CHEMOTAXIS	1	1.112561	0.33465347
Upregulated Genes	GO_ACUTE_INFLAMMATORY_RESPONSE	1	1.112336	0.3187251
Upregulated Genes	GO_REGULATION_OF_POTASSIUM_ION_EXPORT_ACROSS_PLASMA_MEMB	1	1.111834	0.32505175
Upregulated Genes	GO_DEFENSE_RESPONSE_TO_OTHER_ORGANISM	12	1.111633	0.40936863
Upregulated Genes	GO_REGULATION_OF_ACTIN_FILAMENT_LENGTH	1	1.111554	0.3205645
Upregulated Genes	GO_POSITIVE_REGULATION_OF_LYMPHOCYTE_ACTIVATION	4	1.110409	0.36325678

Upregulated Genes	GO_NEGATIVE_REGULATION_OF_VIRAL_TRANSCRIPTION	1	1.110042	0.31284916
Upregulated Genes	GO_VIRAL_GENE_EXPRESSION	7	1.109571	0.37030074
Upregulated Genes	GO_REGULATION_OF_POTASSIUM_ION_TRANSPORT	1	1.108614	0.3415638
Upregulated Genes	GO_ENDOTHELIAL_CELL_APOPTOTIC_PROCESS	1	1.108586	0.34917355
Upregulated Genes	GO_POSITIVE_REGULATION_OF_POTASSIUM_ION_TRANSPORT	1	1.10849	0.34879032
Upregulated Genes	GO_ORGANIC_HYDROXY_COMPOUND_BIOSYNTHETIC_PROCESS	2	1.108419	0.3592437
Upregulated Genes	GO_POSITIVE_REGULATION_OF_LIPID_METABOLIC_PROCESS	2	1.107562	0.36
Upregulated Genes	GO_POSITIVE_REGULATION_BY_HOST_OF_VIRAL_PROCESS	1	1.106583	0.35339805
Upregulated Genes	GO_NEGATIVE_REGULATION_OF_PROTEIN_CONTAINING_COMPLEX_ASSEM	3	1.10624	0.34565216
Upregulated Genes	GO_PHOSPHOLIPID_TRANSPORT	1	1.105802	0.33398822
Upregulated Genes	GO_MODULATION_BY_HOST_OF_VIRAL_PROCESS	1	1.105246	0.36196318
Upregulated Genes	GO_CRANIAL_NERVE_DEVELOPMENT	2	1.105026	0.35607675
Upregulated Genes	GO_POTASSIUM_ION_EXPORT_ACROSS_PLASMA_MEMBRANE	1	1.104262	0.34029227
Upregulated Genes	GO_TRANSMEMBRANE_RECEPTOR_PROTEIN_SERINE_THREONINE_KINASE_	8	1.10343	0.38326848
Upregulated Genes	GO_PURINERGIC_NUCLEOTIDE_RECEPTOR_SIGNALING_PATHWAY	1	1.101929	0.34901962
Upregulated Genes	GO_VACUOLAR_TRANSPORT	2	1.101322	0.35684648
Upregulated Genes	GO_GLUTAMATE_RECEPTOR_SIGNALING_PATHWAY	2	1.100765	0.37028825
Upregulated Genes	GO_INSULIN_LIKE_GROWTH_FACTOR_RECEPTOR_SIGNALING_PATHWAY	1	1.099472	0.32186234
Upregulated Genes	GO_POSITIVE_REGULATION_OF_LYMPHOCYTE_DIFFERENTIATION	1	1.09899	0.34004024
Upregulated Genes	GO_ADULT_BEHAVIOR	2	1.098924	0.3668639
Upregulated Genes	GO_RHYTHMIC_PROCESS	1	1.0984	0.33988214
Upregulated Genes	GO_POST_EMBRYONIC_DEVELOPMENT	1	1.098275	0.35019454
Upregulated Genes	GO_ANIMAL_ORGAN_REGENERATION	1	1.098237	0.35871744
Upregulated Genes	GO_ELECTRON_TRANSPORT_CHAIN	4	1.097923	0.35106382
Upregulated Genes	GO_ESTROUS_CYCLE	1	1.097473	0.35729846
Upregulated Genes	GO_HIPPOCAMPUS_DEVELOPMENT	1	1.097008	0.35059762
Upregulated Genes	GO_STEROID_ESTERIFICATION	2	1.0968	0.37687367
Upregulated Genes	GO_REGULATION_OF_SUBSTRATE_ADHESION_DEPENDENT_CELL_SPREADII	1	1.096566	0.33592233
Upregulated Genes	GO_KERATINOCYTE_PROLIFERATION	1	1.096157	0.35841584
Upregulated Genes	GO_PORE_COMPLEX_ASSEMBLY	1	1.095943	0.35051546
Upregulated Genes	GO_CELL_SUBSTRATE_JUNCTION_ORGANIZATION	3	1.094935	0.36099586
Upregulated Genes	GO_POSITIVE_REGULATION_OF_MACROPHAGE_MIGRATION	1	1.094637	0.33137256
Upregulated Genes	GO_RESPONSE_TO_MOLECULE_OF_BACTERIAL_ORIGIN	1	1.093755	0.35112935
Upregulated Genes	GO_DENTATE_GYRUS_DEVELOPMENT	1	1.093613	0.34615386
Upregulated Genes	GO_FEAR_RESPONSE	1	1.093409	0.3616601
Upregulated Genes	GO_CEREBELLAR_CORTEX_DEVELOPMENT	1	1.093376	0.35741445

Upregulated Genes	GO_POSITIVE_REGULATION_OF_SMOOTH_MUSCLE_CELL_MIGRATION	1	1.093368	0.34666666
Upregulated Genes	GO_CYTOKINE_PRODUCTION_INVOLVED_IN_IMMUNE_RESPONSE	1	1.093009	0.34623218
Upregulated Genes	GO_POSITIVE_REGULATION_OF_CELLULAR_EXTRAVASATION	1	1.091332	0.36032388
Upregulated Genes	GO_REGULATION_OF_CELL_DIVISION	1	1.091021	0.36196318
Upregulated Genes	GO_REGULATION_OF_ALPHA_BETA_T_CELL_ACTIVATION	1	1.090059	0.3529412
Upregulated Genes	GO_PEPTIDYL_LYSINE_HYDROXYLATION	2	1.089767	0.401222
Upregulated Genes	GO_REGULATION_OF_NEURON_MIGRATION	1	1.089452	0.34934497
Upregulated Genes	GO_POSITIVE_REGULATION_OF_ALPHA_BETA_T_CELL_ACTIVATION	1	1.089125	0.36520076
Upregulated Genes	GO_HINDBRAIN_DEVELOPMENT	1	1.089115	0.3484536
Upregulated Genes	GO_NEGATIVE_REGULATION_OF_MUSCLE_CELL_APOPTOTIC_PROCESS	1	1.088833	0.35757574
Upregulated Genes	GO_REGULATION_OF_NEUTROPHIL_EXTRAVASATION	1	1.08872	0.36746988
Upregulated Genes	GO_POSITIVE_REGULATION_OF_INTERLEUKIN_12_PRODUCTION	1	1.088058	0.35797665
Upregulated Genes	GO_B_CELL_MEDIATED_IMMUNITY	1	1.087552	0.35523614
Upregulated Genes	GO_NEGATIVE_REGULATION_OF_VIRAL_ENTRY_INTO_HOST_CELL	1	1.087488	0.35918367
Upregulated Genes	GO_RESPONSE_TO_INTERFERON_GAMMA	1	1.087273	0.37972167
Upregulated Genes	GO_LEUKOCYTE_CHEMOTAXIS_INVOLVED_IN_INFLAMMATORY_RESPONSE	1	1.087043	0.35829958
Upregulated Genes	GO_BIOTIN_METABOLIC_PROCESS	1	1.087026	0.358
Upregulated Genes	GO_REGULATION_OF_INFLAMMATORY_RESPONSE_TO_WOUNDING	1	1.086891	0.3340206
Upregulated Genes	GO_INFLAMMATORY_RESPONSE_TO_WOUNDING	1	1.086701	0.37809917
Upregulated Genes	GO_REGULATION_OF_KERATINOCYTE_PROLIFERATION	1	1.086263	0.3565737
Upregulated Genes	GO_CELL_DIVISION	1	1.086214	0.3515625
Upregulated Genes	GO_NEUTROPHIL_EXTRAVASATION	1	1.086118	0.37310606
Upregulated Genes	GO_REGULATION_OF_SMOOTH_MUSCLE_CELL_CHEMOTAXIS	1	1.085915	0.3709369
Upregulated Genes	GO_REGULATION_OF_ARTERY_MORPHOGENESIS	1	1.085848	0.36363637
Upregulated Genes	GO_RESPONSE_TO_INTERFERON_ALPHA	1	1.085529	0.37711865
Upregulated Genes	GO_POSITIVE_REGULATION_OF_CELL_DIVISION	1	1.085439	0.36534446
Upregulated Genes	GO_RESPONSE_TO_CORTICOSTEROID	1	1.08529	0.39049587
Upregulated Genes	GO_REGULATION_OF_HUMORAL_IMMUNE_RESPONSE	1	1.085209	0.36417323
Upregulated Genes	GO_VASCULAR_ENDOTHELIAL_CELL_PROLIFERATION	1	1.085192	0.38430583
Upregulated Genes	GO_POSITIVE_REGULATION_OF_CARTILAGE_DEVELOPMENT	1	1.084922	0.36507937
Upregulated Genes	GO_LYSOSOMAL_TRANSPORT	2	1.084542	0.39419088
Upregulated Genes	GO_MACROPHAGE_CHEMOTAXIS	1	1.084324	0.35515872
Upregulated Genes	GO_POSITIVE_REGULATION_OF_NEURON_MIGRATION	1	1.084293	0.38640776
Upregulated Genes	GO_GLYCOSYLATION	10	1.084172	0.3856859
Upregulated Genes	GO_RESPONSE_TO_INTERFERON_BETA	1	1.084095	0.3951613
Upregulated Genes	GO_POSITIVE_REGULATION_OF_LEUKOCYTE_ADHESION_TO_VASCULAR_EN	1	1.083535	0.37747034

Upregulated Genes	GO_OVULATION_CYCLE	1	1.083098	0.3484536
Upregulated Genes	GO_T_CELL_CYTOKINE_PRODUCTION	1	1.083057	0.37021276
Upregulated Genes	GO_EXCRETION	1	1.082893	0.3787575
Upregulated Genes	GO_REGULATION_OF_MACROPHAGE_MIGRATION	1	1.082614	0.37425742
Upregulated Genes	GO_REGULATORY_T_CELL_DIFFERENTIATION	1	1.082529	0.37227723
Upregulated Genes	GO_POSITIVE_REGULATION_OF_EPITHELIAL_TO_MESENCHYMAL_TRANSITI	1	1.082278	0.35019454
Upregulated Genes	GO_REGULATION_OF_LIPOPOLYSACCHARIDE_MEDIATED_SIGNALING_PATH	1	1.081636	0.3966942
Upregulated Genes	GO_NEGATIVE_REGULATION_OF_INFLAMMATORY_RESPONSE	1	1.080802	0.3767535
Upregulated Genes	GO_POSITIVE_REGULATION_OF_INFLAMMATORY_RESPONSE	1	1.080505	0.378
Upregulated Genes	GO_NEGATIVE_REGULATION_OF_STRIATED_MUSCLE_CELL_APOPTOTIC_PR	1	1.08034	0.3707415
Upregulated Genes	GO_MYELOID_LEUKOCYTE_DIFFERENTIATION	3	1.080161	0.36726546
Upregulated Genes	GO_METENCEPHALON_DEVELOPMENT	1	1.079725	0.3852459
Upregulated Genes	GO_CD4_POSITIVE_ALPHA_BETA_T_CELL_ACTIVATION	1	1.079609	0.37943926
Upregulated Genes	GO_ACTIN_CYTOSKELETON_REORGANIZATION	1	1.07934	0.39139345
Upregulated Genes	GO_CELLULAR_RESPONSE_TO_MOLECULE_OF_BACTERIAL_ORIGIN	1	1.079054	0.3838384
Upregulated Genes	GO_CYTOPLASMIC_TRANSLATION	6	1.078876	0.37931034
Upregulated Genes	GO_NEGATIVE_REGULATION_OF_DEFENSE_RESPONSE	1	1.07876	0.3709369
Upregulated Genes	GO_NEGATIVE_REGULATION_OF_HUMORAL_IMMUNE_RESPONSE	1	1.07867	0.35363457
Upregulated Genes	GO_SHORT_TERM_MEMORY	1	1.078526	0.38336715
Upregulated Genes	GO_COMPLEMENT_ACTIVATION	1	1.078411	0.41666666
Upregulated Genes	GO_PLATELET_ACTIVATION	2	1.078397	0.39499035
Upregulated Genes	GO_NEGATIVE_REGULATION_OF_COMPLEMENT_ACTIVATION	1	1.078349	0.37972167
Upregulated Genes	GO_LEUKOCYTE_MIGRATION_INVOLVED_IN_INFLAMMATORY_RESPONSE	1	1.078318	0.39039665
Upregulated Genes	GO_POSITIVE_REGULATION_OF_VASCULAR_ENDOTHELIAL_CELL_PROLIFER/	1	1.078209	0.39489195
Upregulated Genes	GO_HUMORAL_IMMUNE_RESPONSE_MEDIATED_BY_CIRCULATING_IMMUI	1	1.078156	0.37724552
Upregulated Genes	GO_REGULATION_OF_BEHAVIOR	1	1.078077	0.40246406
Upregulated Genes	GO_ERAD_PATHWAY	4	1.077677	0.3809524
Upregulated Genes	GO_LIPOPOLYSACCHARIDE_MEDIATED_SIGNALING_PATHWAY	1	1.077599	0.3768116
Upregulated Genes	GO_REGULATION_OF_LEUKOCYTE_ADHESION_TO_VASCULAR_ENDOTHELIA	1	1.07746	0.396728
Upregulated Genes	GO_REGULATION_OF_CHOLESTEROL_ESTERIFICATION	2	1.077417	0.38339922
Upregulated Genes	GO_CELLULAR_RESPONSE_TO_BIOTIC_STIMULUS	1	1.077315	0.38193017
Upregulated Genes	GO_REGULATION_OF_CATION_CHANNEL_ACTIVITY	2	1.077218	0.3838384
Upregulated Genes	GO_INTERLEUKIN_12_PRODUCTION	1	1.077212	0.38019803
Upregulated Genes	GO_SMOOTH_MUSCLE_CELL_MIGRATION	1	1.077139	0.40194175
Upregulated Genes	GO_MUSCLE_CELL_MIGRATION	1	1.076844	0.3888889
Upregulated Genes	GO_MODULATION_BY_SYMBIONT_OF_ENTRY_INTO_HOST	1	1.076778	0.38825756

Upregulated Genes	GO_POSITIVE_REGULATION_OF_MUSCLE_CELL_DIFFERENTIATION	2	1.076471	0.4178499
Upregulated Genes	GO_PROTON_TRANSMEMBRANE_TRANSPORT	3	1.075139	0.37190083
Upregulated Genes	GO_REGULATION_OF_CD4_POSITIVE_ALPHA_BETA_T_CELL_ACTIVATION	1	1.07478	0.37423313
Upregulated Genes	GO_REGULATION_OF_VIRAL_ENTRY_INTO_HOST_CELL	1	1.074702	0.3800813
Upregulated Genes	GO_REGULATION_OF_TRANSPORTER_ACTIVITY	2	1.074626	0.40292275
Upregulated Genes	GO_LIMBIC_SYSTEM_DEVELOPMENT	1	1.073731	0.38476562
Upregulated Genes	GO_REGULATION_OF_CELLULAR_EXTRAVASATION	1	1.073578	0.39539748
Upregulated Genes	GO_SECRETION	26	1.073534	0.3763251
Upregulated Genes	GO_CD4_POSITIVE_ALPHA_BETA_T_CELL_PROLIFERATION	1	1.073326	0.39181286
Upregulated Genes	GO_NEGATIVE_REGULATION_OF_T_CELL_DIFFERENTIATION	1	1.073146	0.39166668
Upregulated Genes	GO_CEREBELLAR_GRANULAR_LAYER_DEVELOPMENT	1	1.072696	0.3920792
Upregulated Genes	GO_PHOSPHOLIPID_CATABOLIC_PROCESS	1	1.072264	0.38663968
Upregulated Genes	GO_REGULATION_OF_NEUROTRANSMITTER_RECEPTOR_ACTIVITY	2	1.072246	0.40222222
Upregulated Genes	GO_REGULATION_OF_RESPIRATORY_GASEOUS_EXCHANGE_BY_NERVOUS_	2	1.072022	0.40361446
Upregulated Genes	GO_REGULATION_OF_ACTIN_CYTOSKELETON_REORGANIZATION	1	1.071483	0.3808554
Upregulated Genes	GO_POSITIVE_REGULATION_OF_MACROPHAGE_CHEMOTAXIS	1	1.071136	0.4
Upregulated Genes	GO_LAYER_FORMATION_IN_CEREBRAL_CORTEX	1	1.070618	0.3916849
Upregulated Genes	GO_O_GLYCAN_PROCESSING	2	1.070419	0.4030501
Upregulated Genes	GO_CD4_POSITIVE_ALPHA_BETA_T_CELL_CYTOKINE_PRODUCTION	1	1.070127	0.40041068
Upregulated Genes	GO_PRODUCTION_OF_MOLECULAR_MEDIATOR_OF_IMMUNE_RESPONSE	1	1.069831	0.3798768
Upregulated Genes	GO_POSITIVE_REGULATION_OF_ANIMAL_ORGAN_MORPHOGENESIS	4	1.069751	0.36820084
Upregulated Genes	GO_ALPHA_BETA_T_CELL_ACTIVATION	1	1.069727	0.41682974
Upregulated Genes	GO_CELLULAR_LIPID_CATABOLIC_PROCESS	1	1.069284	0.38036808
Upregulated Genes	GO_POSITIVE_REGULATION_OF_KERATINOCYTE_PROLIFERATION	1	1.06905	0.3706721
Upregulated Genes	GO_POSITIVE_REGULATION_OF_ALPHA_BETA_T_CELL_PROLIFERATION	1	1.069001	0.39961758
Upregulated Genes	GO_NEGATIVE_REGULATION_OF_LYMPHOCYTE_DIFFERENTIATION	1	1.068794	0.38986355
Upregulated Genes	GO_REGULATION_OF_MACROPHAGE_CHEMOTAXIS	1	1.067795	0.4090909
Upregulated Genes	GO_HUMORAL_IMMUNE_RESPONSE	1	1.067568	0.39
Upregulated Genes	GO_NEGATIVE_REGULATION_OF_EPITHELIAL_CELL_APOPTOTIC_PROCESS	1	1.067211	0.3959596
Upregulated Genes	GO_POSITIVE_REGULATION_OF_CD4_POSITIVE_ALPHA_BETA_T_CELL_ACTI	1	1.067115	0.39405942
Upregulated Genes	GO_NEGATIVE_REGULATION_OF_REGULATORY_T_CELL_DIFFERENTIATION	1	1.066743	0.43699187
Upregulated Genes	GO_POSITIVE_REGULATION_OF_MORPHOGENESIS_OF_AN_EPITHELIUM	1	1.066386	0.40365112
Upregulated Genes	GO_VENTRICULAR_SYSTEM_DEVELOPMENT	1	1.065916	0.386
Upregulated Genes	GO_REGULATION_OF_RESPIRATORY_GASEOUS_EXCHANGE	2	1.064404	0.3973799
Upregulated Genes	GO_ALPHA_BETA_T_CELL_PROLIFERATION	1	1.063978	0.38787878
Upregulated Genes	GO_NEGATIVE_REGULATION_OF_NERVOUS_SYSTEM_DEVELOPMENT	7	1.063945	0.38735178

Upregulated Genes	GO_SMOOTH_MUSCLE_CELL_CHEMOTAXIS	1	1.063871	0.39676112
Upregulated Genes	GO_NOTCH_SIGNALING_PATHWAY	7	1.063287	0.38779527
Upregulated Genes	GO_MUCOPOLYSACCHARIDE_METABOLIC_PROCESS	4	1.06294	0.3717694
Upregulated Genes	GO_POSITIVE_REGULATION_OF_SUBSTRATE_ADHESION_DEPENDENT_CELL	1	1.06293	0.39770555
Upregulated Genes	GO_MUSCLE_CELL_APOPTOTIC_PROCESS	1	1.062857	0.39411765
Upregulated Genes	GO_MULTICELLULAR_ORGANISM_REPRODUCTION	11	1.062692	0.41811174
Upregulated Genes	GO_REGULATION_OF_RESPIRATORY_SYSTEM_PROCESS	2	1.06267	0.42028984
Upregulated Genes	GO_CARDIAC_CONDUCTION	1	1.062487	0.4
Upregulated Genes	GO_STRIATED_MUSCLE_CELL_APOPTOTIC_PROCESS	1	1.061516	0.39648438
Upregulated Genes	GO_POSITIVE_REGULATION_OF_CELL_SUBSTRATE_ADHESION	1	1.059838	0.42209074
Upregulated Genes	GO_REGULATION_OF_COMPLEMENT_DEPENDENT_CYTOTOXICITY	1	1.058815	0.3996063
Upregulated Genes	GO_PHOSPHATIDYLINOSITOL_ACYL_CHAIN_REMODELING	1	1.058411	0.42362526
Upregulated Genes	GO_REGULATION_OF_PROTEIN_TARGETING_TO_MEMBRANE	1	1.05835	0.39726028
Upregulated Genes	GO_NEGATIVE_REGULATION_OF_PHOSPHOLIPID_BIOSYNTHETIC_PROCESS	2	1.058276	0.40442654
Upregulated Genes	GO_GLYCOPROTEIN_METABOLIC_PROCESS	16	1.05759	0.43389198
Upregulated Genes	GO_FEMALE_GAMETE_GENERATION	3	1.057281	0.4116424
Upregulated Genes	GO_RUFFLE_ASSEMBLY	1	1.055683	0.40983605
Upregulated Genes	GO_POSITIVE_REGULATION_OF_CD4_POSITIVE_ALPHA_BETA_T_CELL_PRO	1	1.055568	0.432
Upregulated Genes	GO_RUFFLE_ORGANIZATION	1	1.055315	0.39245284
Upregulated Genes	GO_REGULATION_OF_RUFFLE_ASSEMBLY	1	1.054571	0.41666666
Upregulated Genes	GO_OOGENESIS	3	1.054318	0.38865545
Upregulated Genes	GO_DENDRITIC_SPINE_MORPHOGENESIS	2	1.052885	0.40792078
Upregulated Genes	GO_AMINOGLYCAN_CATABOLIC_PROCESS	2	1.052387	0.40918162
Upregulated Genes	GO_REGULATION_OF_STEROID_METABOLIC_PROCESS	2	1.05121	0.42116183
Upregulated Genes	GO_REGULATION_OF_PHOSPHOLIPID_BIOSYNTHETIC_PROCESS	2	1.050997	0.42217484
Upregulated Genes	GO_SYMBIOTIC_PROCESS	23	1.050495	0.42602494
Upregulated Genes	GO_NEGATIVE_REGULATION_OF_GLYCOPROTEIN_METABOLIC_PROCESS	2	1.050368	0.4262295
Upregulated Genes	GO_REGULATION_OF_PHOSPHATIDYLCHOLINE_METABOLIC_PROCESS	1	1.050152	0.40524194
Upregulated Genes	GO_NEGATIVE_REGULATION_OF_LIPID_METABOLIC_PROCESS	2	1.048961	0.412
Upregulated Genes	GO_REGULATION_OF_MICROTUBULE_CYTOSKELETON_ORGANIZATION	2	1.048886	0.44092828
Upregulated Genes	GO_REGULATION_OF_MUSCLE_ADAPTATION	1	1.048835	0.43469387
Upregulated Genes	GO_REGULATION_OF_MEMBRANE_LIPID_DISTRIBUTION	2	1.048121	0.44636014
Upregulated Genes	GO_REGULATION_OF_MUSCLE_SYSTEM_PROCESS	1	1.046872	0.43578947
Upregulated Genes	GO_DEVELOPMENTAL_PROCESS_INVOLVED_IN_REPRODUCTION	13	1.04685	0.4448743
Upregulated Genes	GO_POSITIVE_REGULATION_OF_SMALL_GTPASE_MEDIATED_SIGNAL_TRAN	1	1.046842	0.4368932
Upregulated Genes	GO_REGULATION_OF_MUSCLE_HYPERTROPHY	1	1.046703	0.42798355

Upregulated Genes	GO_NEGATIVE_REGULATION_OF_LIPID_BIOSYNTHETIC_PROCESS	2	1.04605	0.436
Upregulated Genes	GO_REGULATION_OF_RESPONSE_TO_ENDOPLASMIC_RETICULUM_STRESS	4	1.046021	0.4117647
Upregulated Genes	GO_NEGATIVE_REGULATION_OF_PROTEIN_TARGETING_TO_MEMBRANE	1	1.0458	0.43801653
Upregulated Genes	GO_REGULATION_OF_CELL_MORPHOGENESIS	18	1.045538	0.47192982
Upregulated Genes	GO_CELLULAR_COMPONENT_ASSEMBLY_INVOLVED_IN_MORPHOGENESIS	2	1.044866	0.44230768
Upregulated Genes	GO_REGULATION_OF_PHOSPHATIDYLCHOLINE_BIOSYNTHETIC_PROCESS	1	1.044824	0.43055555
Upregulated Genes	GO_POSITIVE_REGULATION_OF_RUFFLE_ASSEMBLY	1	1.044288	0.43259558
Upregulated Genes	GO_PHOSPHATIDIC_ACID_METABOLIC_PROCESS	2	1.043892	0.40711462
Upregulated Genes	GO_MYOFIBRIL_ASSEMBLY	1	1.043678	0.41769546
Upregulated Genes	GO_SMAD_PROTEIN_SIGNAL_TRANSDUCTION	3	1.042386	0.41188523
Upregulated Genes	GO_NEGATIVE_REGULATION_OF_PHOSPHOLIPID_METABOLIC_PROCESS	2	1.041963	0.43683085
Upregulated Genes	GO_REGULATION_OF_MICROTUBULE_BASED_PROCESS	2	1.040603	0.4491018
Upregulated Genes	GO_METANEPHRIC_NEPHRON_DEVELOPMENT	1	1.039531	0.434238
Upregulated Genes	GO_EMBRYONIC_SKELETAL_SYSTEM_MORPHOGENESIS	2	1.038818	0.41407868
Upregulated Genes	GO_REGULATION_OF_NEURON_PROJECTION_DEVELOPMENT	17	1.038661	0.43818182
Upregulated Genes	GO_VENTRICULAR_CARDIAC_MUSCLE_CELL_DIFFERENTIATION	1	1.038369	0.4625
Upregulated Genes	GO_LIPID_TRANSLOCATION	2	1.038257	0.43451142
Upregulated Genes	GO_SERINE_PHOSPHORYLATION_OF_STAT_PROTEIN	1	1.037805	0.4347826
Upregulated Genes	GO_POSITIVE_REGULATION_OF_GLOMERULUS_DEVELOPMENT	1	1.037132	0.44227007
Upregulated Genes	GO_VENTRICULAR_CARDIAC_MUSCLE_CELL_DEVELOPMENT	1	1.037108	0.44400784
Upregulated Genes	GO_POSITIVE_REGULATION_OF_ENDOCYTOSIS	4	1.036921	0.41787943
Upregulated Genes	GO_REGULATION_OF_CATION_TRANSMEMBRANE_TRANSPORT	4	1.03661	0.41463414
Upregulated Genes	GO_MUSCLE_ADAPTATION	1	1.036146	0.4391635
Upregulated Genes	GO_PHOSPHATIDYLGLYCEROL_ACYL_CHAIN_REMODELING	1	1.036021	0.4189189
Upregulated Genes	GO_PHOSPHATIDYLGLYCEROL_METABOLIC_PROCESS	1	1.035979	0.4308617
Upregulated Genes	GO_REGULATION_OF_HEMOPOIESIS	6	1.035408	0.41633466
Upregulated Genes	GO_PROTEIN_DEPOLYMERIZATION	2	1.035108	0.42417583
Upregulated Genes	GO_CELL_FATE_SPECIFICATION	1	1.034551	0.43153527
Upregulated Genes	GO_MRNA_3_END_PROCESSING	4	1.034155	0.3902439
Upregulated Genes	GO_REGULATION_OF_PROTEIN_GLYCOSYLATION	2	1.033936	0.43776825
Upregulated Genes	GO_SURFACTANT_HOMEOSTASIS	1	1.033794	0.4566474
Upregulated Genes	GO_CARDIOBLAST_DIFFERENTIATION	1	1.03351	0.42063493
Upregulated Genes	GO_URETER_DEVELOPMENT	1	1.033478	0.44246033
Upregulated Genes	GO_EMBRYONIC_SKELETAL_SYSTEM_DEVELOPMENT	2	1.033337	0.43762377
Upregulated Genes	GO_POSITIVE_REGULATION_OF_PEPTIDYL_SERINE_PHOSPHORYLATION	1	1.033003	0.4564797
Upregulated Genes	GO_PHOSPHATIDYLCHOLINE_ACYL_CHAIN_REMODELING	1	1.032752	0.44532803

Upregulated Genes	GO_LYMPHOCYTE_MIGRATION	1	1.032374	0.45
Upregulated Genes	GO_INTERACTION_WITH_HOST	6	1.032229	0.41210938
Upregulated Genes	GO_POSITIVE_REGULATION_OF_CELLULAR_PROTEIN_LOCALIZATION	6	1.031738	0.40524194
Upregulated Genes	GO_POSITIVE_REGULATION_OF_CELL_ADHESION_MEDIATED_BY_INTEGRIN	1	1.031227	0.4302554
Upregulated Genes	GO_REGULATION_OF_TRANSCRIPTION_FROM_RNA_POLYMERASE_II_PROM	1	1.031178	0.43570057
Upregulated Genes	GO_REGULATION_OF_NEURON_MATURATION	1	1.031054	0.43360996
Upregulated Genes	GO_MESENCHYME_MORPHOGENESIS	1	1.030934	0.46470588
Upregulated Genes	GO_RAB_PROTEIN_SIGNAL_TRANSDUCTION	1	1.030586	0.4979757
Upregulated Genes	GO_POSITIVE_REGULATION_OF_RHO_PROTEIN_SIGNAL_TRANSDUCTION	1	1.030569	0.451417
Upregulated Genes	GO_NEURON_PROJECTION_EXTENSION_INVOLVED_IN_NEURON_PROJECTI	2	1.030456	0.43436295
Upregulated Genes	GO_POST_TRANSLATIONAL_PROTEIN_MODIFICATION	11	1.03021	0.42964354
Upregulated Genes	GO_MUSCLE_CELL_FATE_COMMITMENT	1	1.029992	0.46230158
Upregulated Genes	GO_MUSCLE_HYPERTROPHY	1	1.028341	0.4595661
Upregulated Genes	GO_OUTFLOW_TRACT_MORPHOGENESIS	1	1.028169	0.4520548
Upregulated Genes	GO_REGULATION_OF_GLOMERULUS_DEVELOPMENT	1	1.027918	0.46969697
Upregulated Genes	GO_MIDGUT_DEVELOPMENT	1	1.027796	0.44621515
Upregulated Genes	GO_METANEPHROS_DEVELOPMENT	1	1.027445	0.46336633
Upregulated Genes	GO_REGULATION_OF_CELL_ADHESION_MEDIATED_BY_INTEGRIN	1	1.02719	0.45882353
Upregulated Genes	GO_POSITIVE_REGULATION_OF_CELL_MATURATION	1	1.026989	0.4580777
Upregulated Genes	GO_POSITIVE_REGULATION_OF_ORGAN_GROWTH	1	1.026896	0.4661017
Upregulated Genes	GO_REGULATION_OF_PEPTIDYL_SERINE_PHOSPHORYLATION	1	1.02686	0.47058824
Upregulated Genes	GO_POSITIVE_REGULATION_OF_KIDNEY_DEVELOPMENT	1	1.026563	0.43153527
Upregulated Genes	GO_POSITIVE_REGULATION_OF_METANEPHROS_DEVELOPMENT	1	1.026425	0.4621514
Upregulated Genes	GO_POSITIVE_REGULATION_OF_MUSCLE_HYPERTROPHY	1	1.026328	0.4779874
Upregulated Genes	GO_PHOSPHATIDYLINOSITOL_METABOLIC_PROCESS	6	1.026226	0.41528925
Upregulated Genes	GO_METANEPHRIC_GLOMERULUS_DEVELOPMENT	1	1.025867	0.46216768
Upregulated Genes	GO_POSITIVE_REGULATION_OF_CARDIAC_MUSCLE_CELL_PROLIFERATION	1	1.025809	0.4491363
Upregulated Genes	GO_LIPID_BIOSYNTHETIC_PROCESS	13	1.025476	0.4566929
Upregulated Genes	GO_ECTODERMAL_PLACODE_DEVELOPMENT	1	1.025375	0.47047246
Upregulated Genes	GO_MITOCHONDRION_DISTRIBUTION	1	1.024686	0.47764227
Upregulated Genes	GO_POSITIVE_REGULATION_OF_METANEPHRIC_GLOMERULUS_DEVELOPM	1	1.024636	0.46332046
Upregulated Genes	GO_REGULATION_OF_METANEPHROS_DEVELOPMENT	1	1.024373	0.46107784
Upregulated Genes	GO_MITOCHONDRION_LOCALIZATION	2	1.023662	0.47239265
Upregulated Genes	GO_INNERVATION	1	1.022935	0.45908183
Upregulated Genes	GO_ENTERIC_NERVOUS_SYSTEM_DEVELOPMENT	1	1.022736	0.46548325
Upregulated Genes	GO_CELL_ADHESION_MEDIATED_BY_INTEGRIN	1	1.022638	0.47679326

Upregulated Genes	GO_CARDIAC_MYOFIBRIL_ASSEMBLY	1	1.022591	0.4729459
Upregulated Genes	GO_ENDOCARDIAL_CUSHION_DEVELOPMENT	1	1.022584	0.4581749
Upregulated Genes	GO_POSITIVE_REGULATION_OF_HEART_GROWTH	1	1.022477	0.48225468
Upregulated Genes	GO_MUCOSAL_ASSOCIATED_LYMPHOID_TISSUE_DEVELOPMENT	1	1.021934	0.47563353
Upregulated Genes	GO_OUTFLOW_TRACT_SEPTUM_MORPHOGENESIS	1	1.021699	0.4679089
Upregulated Genes	GO_REGULATION_OF_CELL_MATURATION	1	1.019751	0.49
Upregulated Genes	GO_MAMMARY_GLAND_FORMATION	1	1.019482	0.4597938
Upregulated Genes	GO_CELL_FATE_DETERMINATION	1	1.019348	0.4717742
Upregulated Genes	GO_EMBRYONIC_HEART_TUBE_MORPHOGENESIS	2	1.01916	0.44333997
Upregulated Genes	GO_MICROTUBULE_POLYMERIZATION_OR_DEPOLYMERIZATION	2	1.018863	0.4612245
Upregulated Genes	GO_PROTEIN_CONTAINING_COMPLEX_DISASSEMBLY	4	1.018691	0.43786982
Upregulated Genes	GO_CARDIAC_MUSCLE_CELL_MYOBLAST_DIFFERENTIATION	1	1.018471	0.46723044
Upregulated Genes	GO_DENDRITIC_SPINE_DEVELOPMENT	2	1.018471	0.4294606
Upregulated Genes	GO_NEGATIVE_REGULATION_OF_CELLULAR_RESPONSE_TO_HYPOXIA	1	1.018414	0.45633188
Upregulated Genes	GO_ENDOCARDIAL_CUSHION_MORPHOGENESIS	1	1.018323	0.49804688
Upregulated Genes	GO_EMBRYONIC_HEART_TUBE_DEVELOPMENT	2	1.018137	0.45294118
Upregulated Genes	GO_RESPONSE_TO_PAIN	1	1.017196	0.4888889
Upregulated Genes	GO_REGULATION_OF_BINDING	7	1.016992	0.41714287
Upregulated Genes	GO_NEGATIVE_REGULATION_OF_EMBRYONIC_DEVELOPMENT	1	1.016735	0.46975806
Upregulated Genes	GO_REGULATION_OF_MICROTUBULE_POLYMERIZATION_OR_DEPOLYMERI	2	1.016719	0.47311828
Upregulated Genes	GO_REGULATION_OF_METANEPHRIC_GLOMERULUS_DEVELOPMENT	1	1.016692	0.48757172
Upregulated Genes	GO_INTRINSIC_APOPTOTIC_SIGNALING_PATHWAY_IN_RESPONSE_TO_HYP	1	1.01621	0.4597938
Upregulated Genes	GO_CARDIAC_CHAMBER_FORMATION	1	1.015915	0.49596775
Upregulated Genes	GO_NUCLEAR_TRANSCRIBED_MRNA_CATABOLIC_PROCESS_NONSENSE_ME	2	1.015773	0.43505156
Upregulated Genes	GO_REGULATION_OF_PROTEIN_EXIT_FROM_ENDOPLASMIC_RETICULUM	3	1.015583	0.4722222
Upregulated Genes	GO_GLIAL_CELL_DERIVED_NEUROTROPHIC_FACTOR_RECEPTOR_SIGNALIN	1	1.015468	0.4789272
Upregulated Genes	GO_REGULATION_OF_HEART_MORPHOGENESIS	1	1.01541	0.47698745
Upregulated Genes	GO_ATRIOVENTRICULAR_CANAL_DEVELOPMENT	1	1.015384	0.47440946
Upregulated Genes	GO_CELLULAR_PROTEIN_COMPLEX_DISASSEMBLY	4	1.015	0.43313372
Upregulated Genes	GO_POSITIVE_REGULATION_OF_CELL_SIZE	1	1.014899	0.48185483
Upregulated Genes	GO_NEGATIVE_REGULATION_OF_ANIMAL_ORGAN_MORPHOGENESIS	1	1.014601	0.46796116
Upregulated Genes	GO_PHAGOSOME_ACIDIFICATION	1	1.014271	0.47826087
Upregulated Genes	GO_POSITIVE_REGULATION_OF_NEURON_MATURATION	1	1.013753	0.48162475
Upregulated Genes	GO_CALCIUM_ION_TRANSMEMBRANE_IMPORT_INTO_CYTOSOL	1	1.012879	0.4691358
Upregulated Genes	GO_REPRODUCTIVE_SYSTEM_DEVELOPMENT	5	1.012727	0.42566192
Upregulated Genes	GO_CYTOPLASMIC_TRANSLATIONAL_INITIATION	4	1.012256	0.43639922

Upregulated Genes	GO_TOXIN_TRANSPORT	1	1.012087	0.48155737
Upregulated Genes	GO_NEGATIVE_REGULATION_OF_TRANSMEMBRANE_RECEPTOR_PROTEIN_	2	1.01158	0.4449244
Upregulated Genes	GO_REGULATION_OF_CELLULAR_RESPONSE_TO_HYPOXIA	1	1.011362	0.45474613
Upregulated Genes	GO_MAMMARY_GLAND_MORPHOGENESIS	1	1.011081	0.4949495
Upregulated Genes	GO_LYMPHOCYTE_MIGRATION_INTO_LYMPHOID_ORGANS	1	1.011005	0.5092025
Upregulated Genes	GO_POSITIVE_REGULATION_OF_MUSCLE_TISSUE_DEVELOPMENT	1	1.010875	0.5
Upregulated Genes	GO_CYTOSOLIC_CALCIUM_ION_TRANSPORT	1	1.010281	0.512974
Upregulated Genes	GO_ENDOCYTOSIS_INVOLVED_IN_VIRAL_ENTRY_INTO_HOST_CELL	1	1.009708	0.48242188
Upregulated Genes	GO_SEQUESTERING_OF_CALCIUM_ION	1	1.009448	0.4743083
Upregulated Genes	GO_IRON_ION_TRANSPORT	1	1.009194	0.503937
Upregulated Genes	GO_REGULATION_OF_AXON_GUIDANCE	2	1.008524	0.47844827
Upregulated Genes	GO_POSITIVE_REGULATION_OF_CARDIAC_MUSCLE_TISSUE_DEVELOPMENT	1	1.008456	0.5
Upregulated Genes	GO_ACTIVATION_OF_IMMUNE_RESPONSE	7	1.008117	0.42137095
Upregulated Genes	GO_RESPONSE_TO_ALCOHOL	2	1.00682	0.48109242
Upregulated Genes	GO_REGULATION_OF_SYNAPTIC_TRANSMISSION_GLUTAMATERGIC	3	1.006767	0.45064378
Upregulated Genes	GO_NEGATIVE_REGULATION_OF_RESPONSE_TO_ENDOPLASMIC_RETICULU	3	1.005661	0.47272727
Upregulated Genes	GO_REGULATION_OF_PEPTIDYL_SERINE_PHOSPHORYLATION_OF_STAT_PR	1	1.005484	0.4979757
Upregulated Genes	GO_REGULATION_OF_CELL_PROJECTION_ASSEMBLY	8	1.003353	0.4520548
Upregulated Genes	GO_NEGATIVE_REGULATION_OF_HYPOXIA_INDUCED_INTRINSIC_APOPTOT	1	1.002315	0.47758284
Upregulated Genes	GO_SYNAPTIC_TRANSMISSION_GLUTAMATERGIC	3	1.0019	0.43897218
Upregulated Genes	GO_REGULATION_OF_NEURON_DIFFERENTIATION	19	0.999077	0.50185186
Upregulated Genes	GO_NEGATIVE_REGULATION_OF_MORPHOGENESIS_OF_AN_EPITHELIUM	1	0.998193	0.515748
Upregulated Genes	GO_MRNA_PROCESSING	15	0.997407	0.4678899
Upregulated Genes	GO_REGULATION_OF_CHAPERONE_MEDIATED_AUTOPHAGY	2	0.996433	0.45983934
Upregulated Genes	GO_TRANSFERRIN_TRANSPORT	1	0.995559	0.5281837
Upregulated Genes	GO_POSITIVE_REGULATION_OF_NF_KAPPAB_TRANSCRIPTION_FACTOR_AC	2	0.99516	0.47046843
Upregulated Genes	GO_HEXOSE_PHOSPHATE_TRANSPORT	1	0.993526	0.5029586
Upregulated Genes	GO_NEGATIVE_REGULATION_OF_BINDING	5	0.993522	0.45081967
Upregulated Genes	GO_POSITIVE_REGULATION_OF_IMMUNE_RESPONSE	7	0.991422	0.47184467
Upregulated Genes	GO_POSITIVE_REGULATION_OF_BINDING	2	0.991403	0.47433266
Upregulated Genes	GO_ENDOPLASMIC_RETICULUM_TO_CYTOSOL_TRANSPORT	3	0.991369	0.46502057
Upregulated Genes	GO_LIPID_MODIFICATION	8	0.991128	0.4586614
Upregulated Genes	GO_ESTABLISHMENT_OR_MAINTENANCE_OF_MONOPOLAR_CELL_POLARIT	1	0.990766	0.5151515
Upregulated Genes	GO_NEGATIVE_REGULATION_OF_CELL_GROWTH	3	0.990485	0.476
Upregulated Genes	GO_NEGATIVE_REGULATION_OF_CELLULAR_RESPONSE_TO_GROWTH_FAC	2	0.989419	0.5
Upregulated Genes	GO_RESPONSE_TO_STEROL	2	0.989383	0.47357723

Upregulated Genes	GO_VESICLE_UNCOATING	1	0.989177	0.48818898
Upregulated Genes	GO_CELLULAR_COMPONENT_MORPHOGENESIS	25	0.989126	0.48591548
Upregulated Genes	GO_GLUCOSE_6_PHOSPHATE_METABOLIC_PROCESS	1	0.988501	0.5265306
Upregulated Genes	GO_POSITIVE_REGULATION_OF_SYNAPTIC_VESICLE_RECYCLING	1	0.988262	0.5175097
Upregulated Genes	GO_POLARIZED_EPITHELIAL_CELL_DIFFERENTIATION	1	0.987882	0.5364891
Upregulated Genes	GO_UBIQUITIN_DEPENDENT_ERAD_PATHWAY	3	0.987646	0.47628865
Upregulated Genes	GO_ESTABLISHMENT_OF_TISSUE_POLARITY	3	0.987025	0.47389558
Upregulated Genes	GO_RECEPTOR_METABOLIC_PROCESS	3	0.986846	0.47283703
Upregulated Genes	GO_CHAPERONE_MEDIATED_AUTOPHAGY	2	0.986619	0.48987854
Upregulated Genes	GO_NEGATIVE_REGULATION_OF_DEVELOPMENTAL_GROWTH	3	0.985953	0.47717842
Upregulated Genes	GO_GLYCEROLIPID_BIOSYNTHETIC_PROCESS	9	0.985597	0.47674417
Upregulated Genes	GO_RESPONSE_TO_ISCHEMIA	1	0.985517	0.5222672
Upregulated Genes	GO_ESTABLISHMENT_OF_EPITHELIAL_CELL_POLARITY	1	0.98528	0.51276594
Upregulated Genes	GO_T_CELL_DIFFERENTIATION_IN_THYMUS	1	0.984325	0.50927836
Upregulated Genes	GO_REGULATION_OF_EMBRYONIC_DEVELOPMENT	3	0.984182	0.47379455
Upregulated Genes	GO_MYELOID_CELL_DIFFERENTIATION	8	0.984077	0.45564517
Upregulated Genes	GO_REGULATION_OF_LYMPHOCYTE_ACTIVATION	6	0.983485	0.4396887
Upregulated Genes	GO_REGULATION_OF_RESPONSE_TO_EXTERNAL_STIMULUS	14	0.983162	0.509542
Upregulated Genes	GO_REGULATION_OF_MYELOID_LEUKOCYTE_DIFFERENTIATION	2	0.982703	0.4896694
Upregulated Genes	GO_CELL_JUNCTION_ASSEMBLY	8	0.9814	0.4832347
Upregulated Genes	GO_NEGATIVE_REGULATION_OF_MULTICELLULAR_ORGANISMAL_PROCESS	17	0.981278	0.50889677
Upregulated Genes	GO_POSITIVE_REGULATION_OF_INTRACELLULAR_TRANSPORT	5	0.980596	0.44624746
Upregulated Genes	GO_FUCOSYLATION	2	0.979256	0.48681542
Upregulated Genes	GO_ESTABLISHMENT_OF_EPITHELIAL_CELL_APICAL_BASAL_POLARITY	1	0.979139	0.54048586
Upregulated Genes	GO_REGULATION_OF_NERVOUS_SYSTEM_PROCESS	3	0.97795	0.48643005
Upregulated Genes	GO_MESENCHYME_DEVELOPMENT	6	0.977788	0.46736842
Upregulated Genes	GO_CELL_PART_MORPHOGENESIS	24	0.977521	0.52037036
Upregulated Genes	GO_NUCLEOBASE_TRANSPORT	1	0.977521	0.5272727
Upregulated Genes	GO_NEGATIVE_REGULATION_OF_WNT_SIGNALING_PATHWAY	2	0.977127	0.50212765
Upregulated Genes	GO_POSITIVE_REGULATION_OF_SYNAPTIC_VESICLE_ENDOCYTOSIS	1	0.977025	0.5390625
Upregulated Genes	GO_MRNA_EXPORT_FROM_NUCLEUS	4	0.976548	0.44758064
Upregulated Genes	GO_PEPTIDYL_ASPARAGINE_MODIFICATION	2	0.975659	0.5113402
Upregulated Genes	GO_REGULATION_OF_LEUKOCYTE_PROLIFERATION	3	0.975572	0.47082496
Upregulated Genes	GO_REGULATION_OF_CELLULAR_RESPONSE_TO_GROWTH_FACTOR_STIMU	2	0.975457	0.51950717
Upregulated Genes	GO_NEGATIVE_REGULATION_OF_CATALYTIC_ACTIVITY	10	0.971931	0.4846154
Upregulated Genes	GO_OLIGOSACCHARIDE_METABOLIC_PROCESS	2	0.971875	0.5091278

Upregulated Genes	GO_SYNAPTIC_VESICLE_UNCOATING	1	0.971852	0.55349797
Upregulated Genes	GO_POSITIVE_REGULATION_OF_HEMOPOIESIS	2	0.971264	0.53846157
Upregulated Genes	GO_ENDOPLASMIC_RETICULUM_UNFOLDED_PROTEIN_RESPONSE	3	0.970848	0.5077821
Upregulated Genes	GO_REGULATION_OF_POSTSYNAPSE_ORGANIZATION	4	0.970389	0.4330544
Upregulated Genes	GO_PHOSPHOLIPID_BIOSYNTHETIC_PROCESS	10	0.969197	0.46743295
Upregulated Genes	GO_AMINO_ACID_TRANSMEMBRANE_TRANSPORT	2	0.968419	0.5
Upregulated Genes	GO_NEGATIVE_REGULATION_OF_CANONICAL_WNT_SIGNALING_PATHWAY	2	0.968078	0.5168317
Upregulated Genes	GO_RNA_PHOSPHODIESTER_BOND_HYDROLYSIS_EXONUCLEOLYTIC	1	0.967787	0.5371901
Upregulated Genes	GO_RNA_METHYLATION	2	0.967278	0.5132383
Upregulated Genes	GO_RNA_POLYMERASE_II_PREINITIATION_COMPLEX_ASSEMBLY	2	0.967065	0.49899396
Upregulated Genes	GO_INORGANIC_ION_TRANSMEMBRANE_TRANSPORT	11	0.965443	0.48975793
Upregulated Genes	GO_REGULATION_OF_T_CELL_DIFFERENTIATION_IN_THYMUS	1	0.965047	0.55069584
Upregulated Genes	GO_RESPONSE_TO_BMP	5	0.964569	0.4547244
Upregulated Genes	GO_MUSCLE_CELL_DEVELOPMENT	3	0.963649	0.49796748
Upregulated Genes	GO_REGULATION_OF_SYNAPTIC_PLASTICITY	4	0.962722	0.48373982
Upregulated Genes	GO_INTRINSIC_APOPTOTIC_SIGNALING_PATHWAY_IN_RESPONSE_TO_END	3	0.962311	0.4906054
Upregulated Genes	GO_PROTEIN_GLYCOSYLATION_IN_GOLGI	2	0.962192	0.52988046
Upregulated Genes	GO_CELL_MATURATION	4	0.96216	0.4888438
Upregulated Genes	GO_REGULATION_OF_CELL_PROJECTION_ORGANIZATION	25	0.961264	0.54727274
Upregulated Genes	GO_POSITIVE_REGULATION_OF_LEUKOCYTE_DIFFERENTIATION	2	0.960665	0.4928717
Upregulated Genes	GO_INTRACILIARY_TRANSPORT_INVOLVED_IN_CILIUM_ASSEMBLY	1	0.960131	0.54780877
Upregulated Genes	GO_REGULATION_OF_T_CELL_ACTIVATION	6	0.95965	0.4668008
Upregulated Genes	GO_GLYCOSPHINGOLIPID_METABOLIC_PROCESS	1	0.958564	0.5667351
Upregulated Genes	GO_ORGANIC_ACID_BIOSYNTHETIC_PROCESS	6	0.958451	0.4647303
Upregulated Genes	GO_INTRACILIARY_TRANSPORT	1	0.957427	0.56017506
Upregulated Genes	GO_LIPID_STORAGE	2	0.957006	0.5194274
Upregulated Genes	GO_POSITIVE_REGULATION_OF_PHAGOCYTOSIS	3	0.956971	0.5
Upregulated Genes	GO_MACROPHAGE_MIGRATION	2	0.956173	0.5229167
Upregulated Genes	GO_NEGATIVE_REGULATION_OF_AUTOPHAGY	1	0.955628	0.57494867
Upregulated Genes	GO_REGULATION_OF_ANIMAL_ORGAN_MORPHOGENESIS	9	0.954545	0.47034764
Upregulated Genes	GO_REGULATION_OF_CELL_SUBSTRATE_ADHESION	2	0.954523	0.53333336
Upregulated Genes	GO_ACTIN_MEDIATED_CELL_CONTRACTION	2	0.953224	0.5282258
Upregulated Genes	GO_ACTIN_FILAMENT_BASED_MOVEMENT	2	0.952705	0.5387755
Upregulated Genes	GO_GAMETE_GENERATION	9	0.952631	0.49129593
Upregulated Genes	GO_OLIGOSACCHARIDE_CATABOLIC_PROCESS	1	0.950297	0.5566406
Upregulated Genes	GO_NEGATIVE_REGULATION_OF_PROTEIN_LOCALIZATION_TO_MEMBRANI	3	0.950259	0.50980395

Upregulated Genes	GO_MANNOSE_METABOLIC_PROCESS	1	0.949857	0.5770751
Upregulated Genes	GO_REGULATION_OF_TRANSCRIPTION_INITIATION_FROM_RNA_POLYMER/	2	0.949563	0.5230461
Upregulated Genes	GO_ORGANIC_ACID_TRANSMEMBRANE_TRANSPORT	2	0.948548	0.51731604
Upregulated Genes	GO_REGULATION_OF_BLOOD_CIRCULATION	3	0.94634	0.52663934
Upregulated Genes	GO_TRANSCRIPTION_PREINITIATION_COMPLEX_ASSEMBLY	2	0.945848	0.56236327
Upregulated Genes	GO_PURINE_NUCLEOBASE_TRANSPORT	1	0.944377	0.602
Upregulated Genes	GO_PROTEIN_TRANSPORT_ALONG_MICROTUBULE	1	0.941469	0.5968064
Upregulated Genes	GO_REGULATION_OF_DNA_TEMPLATED_TRANSCRIPTION_INITIATION	2	0.940946	0.55186725
Upregulated Genes	GO_MICROTUBULE_ANCHORING	1	0.937441	0.59498954
Upregulated Genes	GO_CARBOHYDRATE_DERIVATIVE_BIOSYNTHETIC_PROCESS	22	0.936477	0.55755395
Upregulated Genes	GO_STEROL_METABOLIC_PROCESS	2	0.936111	0.53927815
Upregulated Genes	GO_SMOOTH_MUSCLE_CELL_PROLIFERATION	1	0.935662	0.5946502
Upregulated Genes	GO_SNRNA_TRANSCRIPTION	2	0.933098	0.5372549
Upregulated Genes	GO_REGULATION_OF_SIGNALING_RECEPTOR_ACTIVITY	3	0.931971	0.5134529
Upregulated Genes	GO_INTRACELLULAR_LIPID_TRANSPORT	3	0.93074	0.52008456
Upregulated Genes	GO_REGULATION_OF_CELL_CELL_ADHESION	8	0.93052	0.4970874
Upregulated Genes	GO_IMMUNE_EFFECTOR_PROCESS	25	0.929952	0.55499154
Upregulated Genes	GO_POSITIVE_REGULATION_OF_IMMUNE_SYSTEM_PROCESS	11	0.92983	0.49808428
Upregulated Genes	GO_TRANSCYTOSIS	1	0.929126	0.61538464
Upregulated Genes	GO_ORGANIC_HYDROXY_COMPOUND_TRANSPORT	3	0.928861	0.54545456
Upregulated Genes	GO_REPRODUCTION	16	0.928423	0.5447619
Upregulated Genes	GO_INTRACELLULAR_STEROL_TRANSPORT	3	0.926091	0.5416667
Upregulated Genes	GO_CELL_MORPHOGENESIS_INVOLVED_IN_NEURON_DIFFERENTIATION	23	0.925948	0.5737122
Upregulated Genes	GO_PROTEIN_IMPORT_INTO_PEROXISOME_MATRIX	1	0.925729	0.5995763
Upregulated Genes	GO_SIGNAL_PEPTIDE_PROCESSING	1	0.925532	0.602459
Upregulated Genes	GO_PROTEIN_TRANSMEMBRANE_IMPORT_INTO_INTRACELLULAR_ORGANE	1	0.925376	0.61473686
Upregulated Genes	GO_RESPONSE_TO_VIRUS	7	0.924637	0.51473475
Upregulated Genes	GO_CELLULAR_PROCESS_INVOLVED_IN_REPRODUCTION_IN_MULTICELLUL/	4	0.924152	0.50220263
Upregulated Genes	GO_TRIGEMINAL_NERVE_MORPHOGENESIS	1	0.924017	0.6079665
Upregulated Genes	GO_NEGATIVE_REGULATION_OF_DNA_BINDING_TRANSCRIPTION_FACTOR_	1	0.924001	0.609375
Upregulated Genes	GO_ORGAN_OR_TISSUE_SPECIFIC_IMMUNE_RESPONSE	1	0.923998	0.61078995
Upregulated Genes	GO_PROTEIN_CONTAINING_COMPLEX_LOCALIZATION	5	0.923935	0.50501
Upregulated Genes	GO_PRESYNAPTIC_ENDOCYTOSIS	5	0.923819	0.49705306
Upregulated Genes	GO_REGULATION_OF_SYNAPTIC_VESICLE_CYCLE	4	0.92314	0.5080645
Upregulated Genes	GO_REGULATION_OF_ENDOPLASMIC_RETICULUM_STRESS_INDUCED_INTR	3	0.922949	0.56631577
Upregulated Genes	GO_INTRA_GOLGI_VESICLE_MEDIATED_TRANSPORT	2	0.922676	0.5654886

Upregulated Genes	GO_GLOMERULUS_VASCULATURE_MORPHOGENESIS	1	0.922016	0.60271317
Upregulated Genes	GO_VIRAL_LIFE_CYCLE	7	0.921842	0.51292247
Upregulated Genes	GO_POSITIVE_REGULATION_OF_SMOOTH_MUSCLE_CELL_PROLIFERATION	1	0.92177	0.5868644
Upregulated Genes	GO_STEROL_TRANSPORT	3	0.920168	0.5511811
Upregulated Genes	GO_MAINTENANCE_OF_LOCATION_IN_CELL	2	0.919839	0.5681818
Upregulated Genes	GO_NCRNA_TRANSCRIPTION	2	0.919053	0.5821206
Upregulated Genes	GO_POSITIVE_REGULATION_OF_AUTOPHAGY	3	0.918704	0.5720165
Upregulated Genes	GO_GLOMERULUS_MORPHOGENESIS	1	0.917523	0.63013697
Upregulated Genes	GO_RENAL_SYSTEM_VASCULATURE_DEVELOPMENT	1	0.916469	0.6359918
Upregulated Genes	GO_RENAL_SYSTEM_VASCULATURE_MORPHOGENESIS	1	0.916368	0.608871
Upregulated Genes	GO_NEURONAL_ION_CHANNEL_CLUSTERING	1	0.916313	0.6091954
Upregulated Genes	GO_GERM_CELL_DEVELOPMENT	4	0.915646	0.55150217
Upregulated Genes	GO_SYNAPTIC_VESICLE_RECYCLING	5	0.915183	0.53781515
Upregulated Genes	GO_NEGATIVE_CHEMOTAXIS	1	0.914693	0.63434345
Upregulated Genes	GO_NEGATIVE_REGULATION_OF_IMMUNE_SYSTEM_PROCESS	5	0.914299	0.5233266
Upregulated Genes	GO_RESPONSE_TO_PURINE_CONTAINING_COMPOUND	2	0.913826	0.552017
Upregulated Genes	GO_PROTEIN_LOCALIZATION_TO_ENDOPLASMIC_RETICULUM	5	0.913612	0.5122449
Upregulated Genes	GO_PROTEIN_LOCALIZATION_TO_CELL_SURFACE	2	0.913332	0.5737052
Upregulated Genes	GO_NEURON_FATE_COMMITMENT	1	0.912629	0.65407556
Upregulated Genes	GO_REGULATION_OF_PROTEIN_LOCALIZATION_TO_CELL_SURFACE	2	0.91226	0.5685885
Upregulated Genes	GO_NEGATIVE_REGULATION_OF_AXON_GUIDANCE	1	0.91146	0.6524272
Upregulated Genes	GO_NEGATIVE_REGULATION_OF_CELLULAR_AMIDE_METABOLIC_PROCESS	2	0.911435	0.56589144
Upregulated Genes	GO_POSITIVE_REGULATION_OF_PROTEIN_LOCALIZATION_TO_MEMBRANE	2	0.911051	0.5851064
Upregulated Genes	GO_FACIAL_NERVE_STRUCTURAL_ORGANIZATION	1	0.910892	0.61584157
Upregulated Genes	GO_POSITIVE_REGULATION_OF_INTRACELLULAR_PROTEIN_TRANSPORT	4	0.910521	0.5530146
Upregulated Genes	GO_POSITIVE_REGULATION_OF_OSSIFICATION	4	0.909499	0.5614754
Upregulated Genes	GO_GANGLION_DEVELOPMENT	1	0.908924	0.63617885
Upregulated Genes	GO_DIAPHRAGM_DEVELOPMENT	1	0.908472	0.64285713
Upregulated Genes	GO_ACTION_POTENTIAL	1	0.908194	0.63461536
Upregulated Genes	GO_SYMPATHETIC_GANGLION_DEVELOPMENT	1	0.907371	0.62240666
Upregulated Genes	GO_PEROXISOMAL_MEMBRANE_TRANSPORT	1	0.907239	0.6563107
Upregulated Genes	GO_SEMAPHORIN_PLEXIN_SIGNALING_PATHWAY	1	0.907221	0.638
Upregulated Genes	GO_FACIAL_NERVE_MORPHOGENESIS	1	0.90713	0.6265306
Upregulated Genes	GO_POSITIVE_REGULATION_OF_TRANSCRIPTION_OF_NOTCH_RECEPTOR_T	1	0.906899	0.65537846
Upregulated Genes	GO_MULTI_ORGANISM_PROCESS	17	0.90669	0.5515267
Upregulated Genes	GO_REGULATION_OF_SYSTEM_PROCESS	7	0.905839	0.48333332

Upregulated Genes	GO_CELLULAR_LIPID_METABOLIC_PROCESS	23	0.904559	0.59574467
Upregulated Genes	GO_REGULATION_OF_MYELOID_CELL_DIFFERENTIATION	4	0.903987	0.52208835
Upregulated Genes	GO_HEART_VALVE_DEVELOPMENT	1	0.903742	0.6541502
Upregulated Genes	GO_SEMAPHORIN_PLEXIN_SIGNALING_PATHWAY_INVOLVED_IN_NEURON_	1	0.902468	0.6635514
Upregulated Genes	GO_NEURON_CELL_CELL_ADHESION	3	0.902307	0.5829694
Upregulated Genes	GO_CRANIAL_NERVE_STRUCTURAL_ORGANIZATION	1	0.901754	0.6535433
Upregulated Genes	GO_TRANSMISSION_OF_NERVE_IMPULSE	1	0.901456	0.63727456
Upregulated Genes	GO_PHOSPHATIDYLCHOLINE_BIOSYNTHETIC_PROCESS	3	0.900413	0.5847107
Upregulated Genes	GO_NEURONAL_ACTION_POTENTIAL_PROPAGATION	1	0.899376	0.672165
Upregulated Genes	GO_EXTRACELLULAR_MATRIX_DISASSEMBLY	2	0.898785	0.589404
Upregulated Genes	GO_POSITIVE_REGULATION_OF_PROTEIN_LOCALIZATION_TO_CELL_PERIPH	2	0.898679	0.5807128
Upregulated Genes	GO_BRANCHIOMOTOR_NEURON_AXON_GUIDANCE	1	0.898566	0.6323232
Upregulated Genes	GO_HETEROTYPIC_CELL_CELL_ADHESION	1	0.897448	0.6568421
Upregulated Genes	GO_ENDOTHELIAL_CELL_CHEMOTAXIS	1	0.89739	0.6471774
Upregulated Genes	GO_SYMPATHETIC_NERVOUS_SYSTEM_DEVELOPMENT	1	0.897272	0.6742268
Upregulated Genes	GO_NEGATIVE_REGULATION_OF_CHEMOTAXIS	1	0.896998	0.64534885
Upregulated Genes	GO_RNA_EXPORT_FROM_NUCLEUS	6	0.894969	0.55186725
Upregulated Genes	GO_LEUKOCYTE_ADHESION_TO_VASCULAR_ENDOTHELIAL_CELL	2	0.894459	0.58208954
Upregulated Genes	GO_NEURAL_CREST_CELL_MIGRATION_INVOLVED_IN_AUTONOMIC_NERV(	1	0.893793	0.64882225
Upregulated Genes	GO_POSITIVE_REGULATION_OF_PROTEIN_EXIT_FROM_ENDOPLASMIC_RET	2	0.893506	0.609127
Upregulated Genes	GO_MOTOR_NEURON_AXON_GUIDANCE	1	0.893506	0.6666667
Upregulated Genes	GO_CLUSTERING_OF_VOLTAGE_GATED_SODIUM_CHANNELS	1	0.893403	0.67196816
Upregulated Genes	GO_RNA_3_END_PROCESSING	6	0.893256	0.53846157
Upregulated Genes	GO_RRNA_METABOLIC_PROCESS	3	0.893254	0.5532359
Upregulated Genes	GO_REGULATION_OF_SUPRAMOLECULAR_FIBER_ORGANIZATION	5	0.893167	0.54375
Upregulated Genes	GO_PROTEIN_DEMANNOSYLATION	2	0.89307	0.59375
Upregulated Genes	GO_ADAPTIVE_IMMUNE_RESPONSE	4	0.892328	0.5575049
Upregulated Genes	GO_ION_TRANSMEMBRANE_TRANSPORT	16	0.891542	0.57592595
Upregulated Genes	GO_REGULATION_OF_MITOCHONDRIAL_FISSION	1	0.891285	0.6707317
Upregulated Genes	GO_PROTEIN_DEGLYCOSYLATION	2	0.889733	0.6064257
Upregulated Genes	GO_REGULATION_OF_DEFENSE_RESPONSE	6	0.88901	0.52988046
Upregulated Genes	GO_COLLAGEN_FIBRIL_ORGANIZATION	2	0.888515	0.6122881
Upregulated Genes	GO_RESPONSE_TO_ENDOGENOUS_STIMULUS	23	0.888189	0.60940325
Upregulated Genes	GO_ENDOSOME_TO_LYSOSOME_TRANSPORT	1	0.887428	0.6582809
Upregulated Genes	GO_COLLAGEN_BIOSYNTHETIC_PROCESS	2	0.886826	0.5720524
Upregulated Genes	GO_EXTRACELLULAR_TRANSPORT	1	0.88647	0.68237704

Upregulated Genes	GO_NEURON_PROJECTION_ORGANIZATION	3	0.886255	0.59009904
Upregulated Genes	GO_FC_RECEPTOR_SIGNALING_PATHWAY	4	0.885952	0.56842107
Upregulated Genes	GO_GOLGI_VESICLE_TRANSPORT	8	0.885799	0.5363458
Upregulated Genes	GO_COGNITION	5	0.882917	0.54831934
Upregulated Genes	GO_SEGMENTATION	3	0.88251	0.59561753
Upregulated Genes	GO_NEGATIVE_REGULATION_OF_ENDOPLASMIC_RETICULUM_STRESS_INDU	2	0.882336	0.627572
Upregulated Genes	GO_NEGATIVE_REGULATION_OF_SMALL_MOLECULE_METABOLIC_PROCES	2	0.8808	0.60327196
Upregulated Genes	GO_SMOOTH_MUSCLE_CELL_DIFFERENTIATION	1	0.880601	0.65052634
Upregulated Genes	GO_ENDOPLASMIC_RETICULUM_MANNOSE_TRIMMING	2	0.880404	0.61554193
Upregulated Genes	GO_SEXUAL_REPRODUCTION	12	0.880044	0.5626168
Upregulated Genes	GO_CELLULAR_EXTRAVASATION	2	0.879163	0.6087866
Upregulated Genes	GO_LOW_DENSITY_LIPOPROTEIN_PARTICLE_RECEPTOR_CATABOLIC_PROCE	2	0.876113	0.6120332
Upregulated Genes	GO_PHOSPHATIDYLCHOLINE_METABOLIC_PROCESS	3	0.874972	0.60082304
Upregulated Genes	GO_TUMOR_NECROSIS_FACTOR_MEDIATED_SIGNALING_PATHWAY	2	0.874563	0.6180698
Upregulated Genes	GO_REGULATION_OF_RECEPTOR_MEDIATED_ENDOCYTOSIS	2	0.874113	0.6083499
Upregulated Genes	GO_POSITIVE_REGULATION_OF_SMOOTH_MUSCLE_CELL_DIFFERENTIATIOI	1	0.873815	0.6919831
Upregulated Genes	GO_REVERSIBLE_DIFFERENTIATION	1	0.873773	0.6937618
Upregulated Genes	GO_RECEPTOR_CATABOLIC_PROCESS	2	0.8735	0.6331967
Upregulated Genes	GO_REGULATION_OF_VASCULAR_ASSOCIATED_SMOOTH_MUSCLE_CELL_D	1	0.87345	0.6584867
Upregulated Genes	GO_REGULATION_OF_PHENOTYPIC_SWITCHING	1	0.872704	0.6872428
Upregulated Genes	GO_PHENOTYPIC_SWITCHING	1	0.87157	0.7071583
Upregulated Genes	GO_IRE1_MEDIATED_UNFOLDED_PROTEIN_RESPONSE	2	0.870785	0.6376812
Upregulated Genes	GO_RESPONSE_TO_TUMOR_NECROSIS_FACTOR	2	0.866674	0.63377196
Upregulated Genes	GO_CELL_JUNCTION_ORGANIZATION	10	0.865869	0.56787765
Upregulated Genes	GO_DEDIFFERENTIATION	1	0.86584	0.7131474
Upregulated Genes	GO_LIPID_GLYCOSYLATION	1	0.865583	0.68615985
Upregulated Genes	GO_REGULATION_OF_ANATOMICAL_STRUCTURE_MORPHOGENESIS	31	0.864454	0.6511628
Upregulated Genes	GO_LOW_DENSITY_LIPOPROTEIN_RECEPTOR_PARTICLE_METABOLIC_PROC	2	0.863408	0.6454918
Upregulated Genes	GO_REGULATION_OF_ACTIN_FILAMENT_BASED_PROCESS	5	0.863125	0.5971944
Upregulated Genes	GO_NUCLEOTIDE_SUGAR_TRANSMEMBRANE_TRANSPORT	1	0.862829	0.7202268
Upregulated Genes	GO_DENDRITE_MORPHOGENESIS	4	0.861953	0.60162604
Upregulated Genes	GO_REGULATION_OF_SMOOTH_MUSCLE_CELL_DIFFERENTIATION	1	0.861945	0.70254403
Upregulated Genes	GO_REGULATION_OF_CHOLESTEROL_EFFLUX	1	0.861243	0.716141
Upregulated Genes	GO_SPHINGOSINE_BIOSYNTHETIC_PROCESS	1	0.860885	0.70392156
Upregulated Genes	GO_NEGATIVE_REGULATION_OF_AMYLOID_PRECURSOR_PROTEIN_BIOSYN	1	0.859983	0.6952191
Upregulated Genes	GO_EMBRYONIC_ORGAN_DEVELOPMENT	7	0.858178	0.5462185

Upregulated Genes	GO_SPHINGOID_METABOLIC_PROCESS	1	0.858177	0.6890244
Upregulated Genes	GO_REGULATION_OF_PROTEIN_LOCALIZATION_TO_MEMBRANE	5	0.855881	0.5638507
Upregulated Genes	GO_CDP_DIACYLGLYCEROL_METABOLIC_PROCESS	1	0.855757	0.71564883
Upregulated Genes	GO_REGULATION_OF_PROTEIN_LOCALIZATION_TO_CELL_PERIPHERY	5	0.855736	0.5896947
Upregulated Genes	GO_EMBRYONIC_PATTERN_SPECIFICATION	3	0.85412	0.60465115
Upregulated Genes	GO_PEPTIDE_CROSS_LINKING	1	0.854107	0.72895277
Upregulated Genes	GO_AMYLOID_BETA_FORMATION	1	0.853564	0.69721115
Upregulated Genes	GO_REGULATION_OF_INTRACELLULAR_LIPID_TRANSPORT	1	0.852879	0.7186898
Upregulated Genes	GO_MALE_SEX_DIFFERENTIATION	3	0.852615	0.6367432
Upregulated Genes	GO_DIOL_BIOSYNTHETIC_PROCESS	1	0.85237	0.70422536
Upregulated Genes	GO_CELL_MORPHOGENESIS	33	0.852245	0.63763064
Upregulated Genes	GO_PROTEIN_O_LINKED_FUCOSYLATION	1	0.852185	0.7299413
Upregulated Genes	GO_TRANSCRIPTION_INITIATION_FROM_RNA_POLYMERASE_II_PROMOTER	3	0.85212	0.6113445
Upregulated Genes	GO_BONE_REMODELING	3	0.852077	0.5991379
Upregulated Genes	GO_OLIGODENDROCYTE_DEVELOPMENT	1	0.852021	0.73320156
Upregulated Genes	GO_CHROMATIN_DISASSEMBLY	1	0.851298	0.7222222
Upregulated Genes	GO_AXON_EXTENSION	9	0.850784	0.578
Upregulated Genes	GO_REGULATION_OF_RECEPTOR_CATABOLIC_PROCESS	1	0.850217	0.7069307
Upregulated Genes	GO_NEGATIVE_REGULATION_OF_CELL_DEVELOPMENT	8	0.85016	0.57338554
Upregulated Genes	GO_POSITIVE_REGULATION_OF_RECEPTOR_CATABOLIC_PROCESS	1	0.849181	0.7134615
Upregulated Genes	GO_PROTEIN_GERANYLGERANYLATION	1	0.848826	0.7375746
Upregulated Genes	GO_AMYLOID_BETA_METABOLIC_PROCESS	1	0.848814	0.7344961
Upregulated Genes	GO_NEGATIVE_REGULATION_OF_SPHINGOLIPID_BIOSYNTHETIC_PROCESS	1	0.846937	0.71862346
Upregulated Genes	GO_POSITIVE_REGULATION_OF_HYDROLASE_ACTIVITY	6	0.846889	0.57843137
Upregulated Genes	GO_MESENCHYMAL_CELL_DIFFERENTIATION	5	0.846378	0.6043478
Upregulated Genes	GO_PLACENTA_DEVELOPMENT	2	0.846081	0.6679842
Upregulated Genes	GO_POLYOL_BIOSYNTHETIC_PROCESS	1	0.845011	0.7295597
Upregulated Genes	GO_ANION_TRANSMEMBRANE_TRANSPORT	6	0.844943	0.57473683
Upregulated Genes	GO_RECEPTOR_MEDIATED_ENDOCYTOSIS_INVOLVED_IN_CHOLESTEROL_TI	1	0.844375	0.7321063
Upregulated Genes	GO_POSITIVE_REGULATION_OF_BIOMINERALIZATION	3	0.843922	0.64166665
Upregulated Genes	GO_REGULATION_OF_LIPID_TRANSPORT	1	0.843231	0.73306775
Upregulated Genes	GO_PRENYLATION	1	0.843219	0.74233127
Upregulated Genes	GO_REGULATION_OF_SPHINGOLIPID_BIOSYNTHETIC_PROCESS	1	0.843133	0.75049114
Upregulated Genes	GO_MRNA_TRANSPORT	6	0.842871	0.59266406
Upregulated Genes	GO_NEGATIVE_REGULATION_OF_STEROL_TRANSPORT	1	0.842851	0.71428573
Upregulated Genes	GO_NEGATIVE_REGULATION_OF_LIPID_TRANSPORT	1	0.842423	0.74327123

Upregulated Genes	GO_CELLULAR_SPHINGOLIPID_HOMEOSTASIS	1	0.841905	0.7255985
Upregulated Genes	GO_REGULATION_OF_RECEPTOR_MEDIATED_ENDOCYTOSIS_INVOLVED_IN	1	0.841542	0.73150104
Upregulated Genes	GO_REGULATION_OF_LOW_DENSITY_LIPOPROTEIN_PARTICLE_RECEPTOR_	1	0.84151	0.7692308
Upregulated Genes	GO_REGULATION_OF_STEROL_TRANSPORT	1	0.841336	0.73493975
Upregulated Genes	GO_EPIBOLY	2	0.841308	0.6779279
Upregulated Genes	GO_NEGATIVE_REGULATION_OF_ENDOCYTOSIS	1	0.840706	0.7348643
Upregulated Genes	GO_RESPONSE_TO_STEROID_HORMONE	4	0.84056	0.609901
Upregulated Genes	GO_REGULATION_OF_PROTEIN_DEPOLYMERIZATION	1	0.840414	0.72037035
Upregulated Genes	GO_POSITIVE_REGULATION_OF_AMYLOID_BETA_FORMATION	1	0.840183	0.7244259
Upregulated Genes	GO_POSITIVE_REGULATION_OF_AMYLOID_PRECURSOR_PROTEIN_CATABO	1	0.840163	0.74590164
Upregulated Genes	GO_REGULATION_OF_MRNA_CATABOLIC_PROCESS	2	0.839388	0.65048546
Upregulated Genes	GO_REGULATION_OF_AMYLOID_PRECURSOR_PROTEIN_CATABOLIC_PROCE	1	0.839235	0.7561905
Upregulated Genes	GO_PROLINE_METABOLIC_PROCESS	1	0.839055	0.7398844
Upregulated Genes	GO_PROLINE_BIOSYNTHETIC_PROCESS	1	0.838829	0.72289157
Upregulated Genes	GO_REGULATION_OF_LIPID_LOCALIZATION	1	0.838811	0.7352381
Upregulated Genes	GO_CERAMIDE_TRANSPORT	1	0.83825	0.74174756
Upregulated Genes	GO_MICROVILLUS_ASSEMBLY	2	0.838109	0.64605546
Upregulated Genes	GO_AXON_ENSHEATHMENT_IN_CENTRAL_NERVOUS_SYSTEM	1	0.837919	0.74951077
Upregulated Genes	GO_LIPID_LOCALIZATION	5	0.83788	0.6200417
Upregulated Genes	GO_ALCOHOL_BIOSYNTHETIC_PROCESS	1	0.836717	0.74788135
Upregulated Genes	GO_AMYLOID_PRECURSOR_PROTEIN_CATABOLIC_PROCESS	1	0.836695	0.7504798
Upregulated Genes	GO_REGULATION_OF_AMYLOID_BETA_FORMATION	1	0.835169	0.74563104
Upregulated Genes	GO_POSITIVE_REGULATION_OF_SYNAPTIC_TRANSMISSION	4	0.835148	0.64128256
Upregulated Genes	GO_STEROL_HOMEOSTASIS	1	0.835095	0.7463918
Upregulated Genes	GO_LIPID_HOMEOSTASIS	1	0.835031	0.7356322
Upregulated Genes	GO_REGULATION_OF_FILOPODIUM_ASSEMBLY	4	0.834941	0.607362
Upregulated Genes	GO_NEGATIVE_REGULATION_OF_LIPID_LOCALIZATION	1	0.83492	0.7371541
Upregulated Genes	GO_MYELIN_ASSEMBLY	1	0.834373	0.7542857
Upregulated Genes	GO_MALE_GAMETE_GENERATION	5	0.833942	0.6164383
Upregulated Genes	GO_REGULATION_OF_CHROMATIN_ASSEMBLY_OR_DISASSEMBLY	1	0.833405	0.7505071
Upregulated Genes	GO_KERATAN_SULFATE_METABOLIC_PROCESS	1	0.833192	0.72669494
Upregulated Genes	GO_BIOLOGICAL_ADHESION	34	0.832944	0.6937394
Upregulated Genes	GO_PHOSPHOLIPID_METABOLIC_PROCESS	13	0.832707	0.6302368
Upregulated Genes	GO_STEM_CELL_DIFFERENTIATION	4	0.832212	0.62450594
Upregulated Genes	GO_REGULATION_OF_CELLULAR_AMIDE_METABOLIC_PROCESS	6	0.831275	0.6474359
Upregulated Genes	GO_REGULATION_OF_PROTEIN_CONTAINING_COMPLEX_DISASSEMBLY	1	0.830475	0.7470817

Upregulated Genes	GO_MICROVILLUS_ORGANIZATION	2	0.830203	0.661157
Upregulated Genes	GO_KERATAN_SULFATE_CATABOLIC_PROCESS	1	0.830063	0.7407407
Upregulated Genes	GO_MICROTUBULE_DEPOLYMERIZATION	1	0.830039	0.7345679
Upregulated Genes	GO_CHOLESTEROL_EFFLUX	1	0.830028	0.7378049
Upregulated Genes	GO_DIOL_METABOLIC_PROCESS	1	0.829497	0.7656566
Upregulated Genes	GO_PURINE_NUCLEOSIDE_MONOPHOSPHATE_METABOLIC_PROCESS	2	0.828929	0.6809816
Upregulated Genes	GO_NEGATIVE_REGULATION_OF_RECEPTOR_MEDIATED_ENDOCYTOSIS	1	0.827367	0.7470588
Upregulated Genes	GO_REGULATION_OF_PEPTIDYL_TYROSINE_PHOSPHORYLATION	4	0.826521	0.675
Upregulated Genes	GO_NEGATIVE_REGULATION_OF_CHOLESTEROL_EFFLUX	1	0.82644	0.77220076
Upregulated Genes	GO_POSITIVE_REGULATION_OF_I_KAPPAB_KINASE_NF_KAPPAB_SIGNALIN(	5	0.825594	0.60330576
Upregulated Genes	GO_OSTEOBLAST_DIFFERENTIATION	3	0.824743	0.67351127
Upregulated Genes	GO_REGULATION_OF_MICROTUBULE_DEPOLYMERIZATION	1	0.824708	0.76829267
Upregulated Genes	GO_ESTABLISHMENT_OF_MITOCHONDRION_LOCALIZATION	1	0.824504	0.7525355
Upregulated Genes	GO_MORPHOGENESIS_OF_AN_EPITHELIAL_SHEET	2	0.824037	0.6889353
Upregulated Genes	GO_TRANSLATIONAL_ELONGATION	2	0.823578	0.7004049
Upregulated Genes	GO_MICROTUBULE_BUNDLE_FORMATION	1	0.823206	0.78811884
Upregulated Genes	GO_RESPONSE_TO_ORGANIC_CYCLIC_COMPOUND	11	0.822634	0.60078275
Upregulated Genes	GO_I_KAPPAB_KINASE_NF_KAPPAB_SIGNALING	5	0.822257	0.6129666
Upregulated Genes	GO_AMP_METABOLIC_PROCESS	2	0.821456	0.6666667
Upregulated Genes	GO_TRANSFORMING_GROWTH_FACTOR_BETA_RECEPTOR_SIGNALING_PAT	3	0.821336	0.6496945
Upregulated Genes	GO_REGULATION_OF_PHAGOCYTOSIS	4	0.82123	0.64915967
Upregulated Genes	GO_CORONARY_VASCULATURE_DEVELOPMENT	1	0.819452	0.76817286
Upregulated Genes	GO_POSITIVE_REGULATION_OF_AXON_GUIDANCE	1	0.818559	0.7689243
Upregulated Genes	GO_NEGATIVE_REGULATION_OF_CELLULAR_RESPONSE_TO_TRANSFORMIN	1	0.816054	0.78119004
Upregulated Genes	GO_DETERMINATION_OF_DIGESTIVE_TRACT_LEFT_RIGHT_ASYMMETRY	1	0.814767	0.8096234
Upregulated Genes	GO_RESPONSE_TO_MECHANICAL_STIMULUS	4	0.814649	0.6439232
Upregulated Genes	GO_MAGNESIUM_ION_TRANSPORT	1	0.814529	0.76759064
Upregulated Genes	GO_REGULATION_OF_MICROVILLUS_ORGANIZATION	2	0.81409	0.7117647
Upregulated Genes	GO_LEFT_RIGHT_PATTERN_FORMATION	1	0.813477	0.78373015
Upregulated Genes	GO_DNA_TEMPLATED_TRANSCRIPTION_INITIATION	3	0.813409	0.6555118
Upregulated Genes	GO_ANTIGEN_PROCESSING_AND_PRESENTATION_OF_PEPTIDE_ANTIGEN	3	0.812653	0.6531049
Upregulated Genes	GO_DOPAMINERGIC_NEURON_AXON_GUIDANCE	1	0.812628	0.7733333
Upregulated Genes	GO_POSITIVE_REGULATION_OF_SMALL_MOLECULE_METABOLIC_PROCESS	3	0.812204	0.64718163
Upregulated Genes	GO_REGULATION_OF_MEMBRANE_POTENTIAL	5	0.809895	0.6082251
Upregulated Genes	GO_PEPTIDYL_SERINE_MODIFICATION	5	0.809781	0.62343097
Upregulated Genes	GO_GLYCEROPHOSPHOLIPID_METABOLIC_PROCESS	11	0.808766	0.60491496

Upregulated Genes	GO_CELLULAR_RESPONSE_TO_CHOLESTEROL	1	0.808022	0.7996071
Upregulated Genes	GO_LEUKOCYTE_CELL_CELL_ADHESION	6	0.807945	0.62355214
Upregulated Genes	GO_REGULATION_OF_CELL_SHAPE	5	0.807556	0.6352459
Upregulated Genes	GO_REGULATION_OF_MICROVILLUS_ASSEMBLY	2	0.80703	0.7190776
Upregulated Genes	GO_CALCIUM_ION_TRANSMEMBRANE_TRANSPORT	4	0.806937	0.6592742
Upregulated Genes	GO_MEMBRANE_LIPID_BIOSYNTHETIC_PROCESS	5	0.806303	0.63580245
Upregulated Genes	GO_REGULATION_OF_PROTEIN_LOCALIZATION_TO_PLASMA_MEMBRANE	4	0.806004	0.6687898
Upregulated Genes	GO_CELLULAR_RESPONSE_TO_STEROL	1	0.805508	0.7956349
Upregulated Genes	GO_OSSIFICATION	11	0.805382	0.65536726
Upregulated Genes	GO_OOCYTE_DIFFERENTIATION	2	0.804718	0.7081545
Upregulated Genes	GO_CELLULAR_RESPONSE_TO_INORGANIC_SUBSTANCE	6	0.804446	0.6257198
Upregulated Genes	GO_CELL_MORPHOGENESIS_INVOLVED_IN_DIFFERENTIATION	28	0.802672	0.6991597
Upregulated Genes	GO_FILOPODIUM_ASSEMBLY	4	0.802611	0.67157894
Upregulated Genes	GO_ESTABLISHMENT_OF_CELL_POLARITY	3	0.801304	0.6701245
Upregulated Genes	GO_RESPONSE_TO_FATTY_ACID	1	0.800944	0.78350514
Upregulated Genes	GO_FATTY_ACID_DERIVATIVE_METABOLIC_PROCESS	1	0.800617	0.80040324
Upregulated Genes	GO_ESTABLISHMENT_OF_PROTEIN_LOCALIZATION_TO_ENDOPLASMIC_RET	3	0.800536	0.6592292
Upregulated Genes	GO_MEMBRANE_LIPID_METABOLIC_PROCESS	7	0.799152	0.6363636
Upregulated Genes	GO_RESPONSE_TO_OLEIC_ACID	1	0.797937	0.80290455
Upregulated Genes	GO_NEGATIVE_REGULATION_OF_GROWTH	4	0.797656	0.6679612
Upregulated Genes	GO_CELLULAR_RESPONSE_TO_ENDOGENOUS_STIMULUS	19	0.797589	0.67537314
Upregulated Genes	GO_KETONE_BODY_METABOLIC_PROCESS	1	0.797344	0.79960316
Upregulated Genes	GO_POSITIVE_REGULATION_OF_CELL_JUNCTION_ASSEMBLY	3	0.797256	0.6953781
Upregulated Genes	GO_REGULATION_OF_CELLULAR_RESPONSE_TO_TRANSFORMING_GROWTI	1	0.795223	0.7906504
Upregulated Genes	GO_RESPONSE_TO_ETHANOL	1	0.794255	0.82684827
Upregulated Genes	GO_KETONE_BODY_BIOSYNTHETIC_PROCESS	1	0.794165	0.8165681
Upregulated Genes	GO_FUCOSE_METABOLIC_PROCESS	1	0.794028	0.8128655
Upregulated Genes	GO_ANTERIOR_POSTERIOR_PATTERN_SPECIFICATION	2	0.793188	0.73185486
Upregulated Genes	GO_POSITIVE_REGULATION_OF_LIPID_KINASE_ACTIVITY	1	0.792499	0.7871486
Upregulated Genes	GO_AEROBIC_RESPIRATION	2	0.791445	0.7295082
Upregulated Genes	GO_CELLULAR_DEFENSE_RESPONSE	2	0.791428	0.7378436
Upregulated Genes	GO_MEMBRANE_ORGANIZATION	17	0.790934	0.6889313
Upregulated Genes	GO_PROTEIN_TRANSMEMBRANE_TRANSPORT	3	0.790526	0.68443495
Upregulated Genes	GO_N_GLYCAN_PROCESSING	1	0.78887	0.8228106
Upregulated Genes	GO_BLASTODERM_SEGMENTATION	2	0.788724	0.74115044
Upregulated Genes	GO_FATTY_ACID_DERIVATIVE_BIOSYNTHETIC_PROCESS	1	0.788021	0.8416834

Upregulated Genes	GO_RESPONSE_TO_NUTRIENT	1	0.787059	0.81346154
Upregulated Genes	GO_POSITIVE_REGULATION_OF_MYELOID_CELL_DIFFERENTIATION	1	0.786892	0.82703775
Upregulated Genes	GO_RESPONSE_TO_ELECTRICAL_STIMULUS	1	0.786806	0.8110236
Upregulated Genes	GO_HEXOSE_CATABOLIC_PROCESS	1	0.785159	0.829703
Upregulated Genes	GO_REGULATION_OF_PROTEIN_KINASE_C_ACTIVITY	1	0.784811	0.8108108
Upregulated Genes	GO_GENITALIA_DEVELOPMENT	1	0.783412	0.8178054
Upregulated Genes	GO_REGULATION_OF_MACROPHAGE_DIFFERENTIATION	1	0.783244	0.8488806
Upregulated Genes	GO_MONOSACCHARIDE_CATABOLIC_PROCESS	1	0.78282	0.8333333
Upregulated Genes	GO_REGULATION_OF_ACTIN_FILAMENT_ORGANIZATION	3	0.782808	0.69609857
Upregulated Genes	GO_CELLULAR_RESPONSE_TO_ALCOHOL	1	0.782361	0.84063745
Upregulated Genes	GO_ASTROCYTE_DEVELOPMENT	1	0.781987	0.833002
Upregulated Genes	GO_OLIGOSACCHARIDE_BIOSYNTHETIC_PROCESS	1	0.781836	0.82965934
Upregulated Genes	GO_MALE_GENITALIA_DEVELOPMENT	1	0.781648	0.80241936
Upregulated Genes	GO_POSITIVE_REGULATION_OF_SYNAPSE_ASSEMBLY	3	0.781209	0.7237113
Upregulated Genes	GO_FUCOSE_CATABOLIC_PROCESS	1	0.78085	0.8255814
Upregulated Genes	GO_REGULATION_OF_ENDOCYTOSIS	8	0.780374	0.6536965
Upregulated Genes	GO_REGULATION_OF_JUN_KINASE_ACTIVITY	1	0.780307	0.83433133
Upregulated Genes	GO_SOMITOGENESIS	1	0.779865	0.8279352
Upregulated Genes	GO_REGULATION_OF_MACROAUTOPHAGY	3	0.779582	0.69877046
Upregulated Genes	GO_POSITIVE_REGULATION_OF_MACROPHAGE_DIFFERENTIATION	1	0.77947	0.8134172
Upregulated Genes	GO_RESPONSE_TO_NITROGEN_COMPOUND	14	0.779448	0.64738804
Upregulated Genes	GO_GENITALIA_MORPHOGENESIS	1	0.779439	0.8422131
Upregulated Genes	GO_PRESYNAPTIC_MODULATION_OF_CHEMICAL_SYNAPTIC_TRANSMISSIOI	2	0.779388	0.7490196
Upregulated Genes	GO_POSITIVE_REGULATION_OF_TRANSLATION	3	0.779052	0.6985447
Upregulated Genes	GO_MACROPHAGE_DIFFERENTIATION	1	0.778821	0.8220859
Upregulated Genes	GO_ASTROCYTE_DIFFERENTIATION	1	0.777376	0.84462154
Upregulated Genes	GO_REGULATION_OF_GLYCOPROTEIN_METABOLIC_PROCESS	4	0.776693	0.69649804
Upregulated Genes	GO_POSITIVE_REGULATION_OF_MYELOID_LEUKOCYTE_DIFFERENTIATION	1	0.775594	0.82186234
Upregulated Genes	GO_SOMITE_DEVELOPMENT	1	0.774273	0.84962404
Upregulated Genes	GO_COTRANSLATIONAL_PROTEIN_TARGETING_TO_MEMBRANE	3	0.773533	0.7338552
Upregulated Genes	GO_REGULATION_OF_IMMUNE_RESPONSE	9	0.772491	0.64777327
Upregulated Genes	GO_POSTSYNAPSE_ORGANIZATION	5	0.771864	0.67469877
Upregulated Genes	GO_NEGATIVE_REGULATION_OF_PLASMA_MEMBRANE_BOUNDED_CELL_P	1	0.771255	0.8458498
Upregulated Genes	GO_MICROTUBULE_CYTOSKELETON_ORGANIZATION	4	0.771161	0.72190475
Upregulated Genes	GO_L_AMINO_ACID_TRANSPORT	1	0.771156	0.85360825
Upregulated Genes	GO_SEX_DIFFERENTIATION	4	0.77081	0.7099391

Upregulated Genes	GO_BASIC_AMINO_ACID_TRANSPORT	1	0.769686	0.87903225
Upregulated Genes	GO_NEGATIVE_REGULATION_OF_ATP_METABOLIC_PROCESS	1	0.769101	0.85542166
Upregulated Genes	GO_NUCLEOTIDE_SUGAR_METABOLIC_PROCESS	1	0.769013	0.87344396
Upregulated Genes	GO_EARLY_ENDOSOME_TO_GOLGI_TRANSPORT	1	0.768098	0.83232325
Upregulated Genes	GO_ORNITHINE_TRANSPORT	1	0.768098	0.8408163
Upregulated Genes	GO_RNA_PROCESSING	25	0.76775	0.75
Upregulated Genes	GO_L_ALPHA_AMINO_ACID_TRANSMEMBRANE_TRANSPORT	1	0.76667	0.84294236
Upregulated Genes	GO_REGULATION_OF_HYDROLASE_ACTIVITY	17	0.766609	0.68441063
Upregulated Genes	GO_REGULATION_OF_NOTCH_SIGNALING_PATHWAY	5	0.76653	0.7083333
Upregulated Genes	GO_PEROXISOME_ORGANIZATION	2	0.766295	0.7631027
Upregulated Genes	GO_ACTOMYOSIN_STRUCTURE_ORGANIZATION	3	0.7652	0.742
Upregulated Genes	GO_CELL_AGGREGATION	2	0.764517	0.7407407
Upregulated Genes	GO_REGULATION_OF_CELL_ADHESION	13	0.76447	0.6659751
Upregulated Genes	GO_NEGATIVE_REGULATION_OF_GLYCOLYTIC_PROCESS	1	0.764378	0.83233535
Upregulated Genes	GO_REGULATION_OF_CELLULAR_RESPIRATION	1	0.764313	0.85943776
Upregulated Genes	GO_POSITIVE_REGULATION_OF_JUN_KINASE_ACTIVITY	1	0.763647	0.8517034
Upregulated Genes	GO_REGULATION_OF_CELL_SUBSTRATE_JUNCTION_ORGANIZATION	1	0.763537	0.85490197
Upregulated Genes	GO_NEGATIVE_REGULATION_OF_NUCLEOTIDE_METABOLIC_PROCESS	1	0.763155	0.85971946
Upregulated Genes	GO_ORGANOPHOSPHATE_ESTER_TRANSPORT	4	0.762888	0.6875
Upregulated Genes	GO_FOCAL_ADHESION_ASSEMBLY	1	0.762578	0.8576998
Upregulated Genes	GO_RHO_PROTEIN_SIGNAL_TRANSDUCTION	4	0.76251	0.720339
Upregulated Genes	GO_AXON_DEVELOPMENT	22	0.762169	0.71875
Upregulated Genes	GO_GRANULOCYTE_DIFFERENTIATION	1	0.761709	0.8559838
Upregulated Genes	GO_NEURON_DEVELOPMENT	33	0.760587	0.76829267
Upregulated Genes	GO_REGULATION_OF_CELLULAR_COMPONENT_BIOGENESIS	18	0.759936	0.6865672
Upregulated Genes	GO_BASIC_AMINO_ACID_TRANSMEMBRANE_TRANSPORT	1	0.759237	0.86220473
Upregulated Genes	GO_REGULATION_OF_AEROBIC_RESPIRATION	1	0.75818	0.86153847
Upregulated Genes	GO_CELL_MATRIX_ADHESION	1	0.758137	0.86773545
Upregulated Genes	GO_EPITHELIAL_TO_MESENCHYMAL_TRANSITION	3	0.756547	0.7470588
Upregulated Genes	GO_EXTRINSIC_APOPTOTIC_SIGNALING_PATHWAY_VIA_DEATH_DOMAIN_I	1	0.756462	0.8522954
Upregulated Genes	GO_PEROXISOMAL_TRANSPORT	2	0.755772	0.76086956
Upregulated Genes	GO_PEPTIDE_BIOSYNTHETIC_PROCESS	17	0.755115	0.7262136
Upregulated Genes	GO_REGULATION_OF_EXTRINSIC_APOPTOTIC_SIGNALING_PATHWAY_VIA_I	1	0.754881	0.8703704
Upregulated Genes	GO_REGULATION_OF_SYNAPSE_ASSEMBLY	4	0.754367	0.71161824
Upregulated Genes	GO_REGULATION_OF_CELL_MATRIX_ADHESION	1	0.75253	0.8742268
Upregulated Genes	GO_MIDBRAIN_DEVELOPMENT	1	0.751954	0.88291746

Upregulated Genes	GO_SUBSTANTIA_NIGRA_DEVELOPMENT	1	0.751429	0.84909457
Upregulated Genes	GO_CELL_ACTIVATION	27	0.751349	0.7321739
Upregulated Genes	GO_REGULATION_OF_BONE_REMODELING	2	0.75086	0.7751938
Upregulated Genes	GO_GOLGI_TO_PLASMA_MEMBRANE_TRANSPORT	1	0.749945	0.8847737
Upregulated Genes	GO_RNA_LOCALIZATION	8	0.74988	0.68530023
Upregulated Genes	GO_POSITIVE_REGULATION_OF_OSTEOBLAST_DIFFERENTIATION	2	0.749738	0.7770961
Upregulated Genes	GO_PROTEIN_LOCALIZATION_TO_ENDOPLASMIC_RETICULUM_EXIT_SITE	1	0.749159	0.88114756
Upregulated Genes	GO_PIGMENT_BIOSYNTHETIC_PROCESS	1	0.748625	0.8701826
Upregulated Genes	GO_MAINTENANCE_OF_LOCATION	4	0.746668	0.7257019
Upregulated Genes	GO_LACTATION	1	0.746662	0.8799172
Upregulated Genes	GO_VESICLE_TARGETING_TO_FROM_OR_WITHIN_GOLGI	1	0.745224	0.86742425
Upregulated Genes	GO_GROOMING_BEHAVIOR	1	0.744656	0.89361703
Upregulated Genes	GO_GOLGI_VESICLE_BUDDING	1	0.743989	0.8732106
Upregulated Genes	GO_POSITIVE_REGULATION_OF_PROTEIN_LOCALIZATION_TO_CELL_SURFA	1	0.743571	0.90427697
Upregulated Genes	GO_NUCLEOSIDE_SALVAGE	1	0.743392	0.8792079
Upregulated Genes	GO_POSITIVE_REGULATION_OF_PLASMA_MEMBRANE_BOUNDED_CELL_PR	3	0.742977	0.73596674
Upregulated Genes	GO_REGULATION_OF_ERAD_PATHWAY	1	0.742074	0.9087221
Upregulated Genes	GO_PURINE_CONTAINING_COMPOUND_SALVAGE	1	0.741728	0.8797595
Upregulated Genes	GO_DEFENSE_RESPONSE	19	0.740485	0.7246377
Upregulated Genes	GO_GOLGI_TO_PLASMA_MEMBRANE_PROTEIN_TRANSPORT	1	0.740415	0.8861004
Upregulated Genes	GO_NUCLEOTIDE_SALVAGE	1	0.740394	0.9089069
Upregulated Genes	GO_COPII_COATED_VESICLE_BUDDING	1	0.740304	0.8904665
Upregulated Genes	GO_POSITIVE_REGULATION_OF_DENDRITE_MORPHOGENESIS	1	0.739702	0.8981289
Upregulated Genes	GO_VESICLE_MEDIATED_TRANSPORT_TO_THE_PLASMA_MEMBRANE	1	0.739425	0.9063097
Upregulated Genes	GO_REGULATION_OF_OSTEOBLAST_DIFFERENTIATION	2	0.738939	0.8049281
Upregulated Genes	GO_PURINE_NUCLEOBASE_METABOLIC_PROCESS	1	0.73841	0.8998035
Upregulated Genes	GO_ENDOCRINE_SYSTEM_DEVELOPMENT	4	0.737946	0.74552685
Upregulated Genes	GO_ESTABLISHMENT_OF_RNA_LOCALIZATION	8	0.737807	0.6857749
Upregulated Genes	GO_PURINE_NUCLEOBASE_BIOSYNTHETIC_PROCESS	1	0.737573	0.8819876
Upregulated Genes	GO_BODY_FLUID_SECRETION	1	0.737362	0.8795918
Upregulated Genes	GO_RESPONSE_TO_CAMP	1	0.737356	0.8984375
Upregulated Genes	GO_VACUOLAR_PROTON_TRANSPORTING_V_TYPE_ATPASE_COMPLEX_ASS	1	0.736715	0.91451293
Upregulated Genes	GO_CELLULAR_METABOLIC_COMPOUND_SALVAGE	1	0.73595	0.9163347
Upregulated Genes	GO_PIGMENT_METABOLIC_PROCESS	1	0.735879	0.88235295
Upregulated Genes	GO_POSITIVE_REGULATION_OF_EXTRINSIC_APOPTOTIC_SIGNALING_PATH\	1	0.735653	0.8785425
Upregulated Genes	GO_POSITIVE_REGULATION_OF_DENDRITE_DEVELOPMENT	1	0.735642	0.8914286

Upregulated Genes	GO_GLYCOSYL_COMPOUND_BIOSYNTHETIC_PROCESS	1	0.735448	0.8962076
Upregulated Genes	GO_NEGATIVE_REGULATION_OF_PROTEIN_EXIT_FROM_ENDOPLASMIC_RE	1	0.735186	0.90039843
Upregulated Genes	GO_NEURAL_NUCLEUS_DEVELOPMENT	1	0.735095	0.8978723
Upregulated Genes	GO_VESICLE_BUDDING_FROM_MEMBRANE	1	0.734798	0.89506173
Upregulated Genes	GO_RESPONSE_TO_LIPID	10	0.734469	0.6881497
Upregulated Genes	GO_PURINE_NUCLEOTIDE_SALVAGE	1	0.734233	0.88867563
Upregulated Genes	GO_MATERNAL_PLACENTA_DEVELOPMENT	1	0.73371	0.8852459
Upregulated Genes	GO_MAINTENANCE_OF_PROTEIN_LOCATION_IN_CELL	1	0.733519	0.9190871
Upregulated Genes	GO_REGULATION_OF_AUTOPHAGY	8	0.733372	0.6764706
Upregulated Genes	GO_MAINTENANCE_OF_PROTEIN_LOCATION	1	0.733342	0.88729507
Upregulated Genes	GO_PURINE_RIBONUCLEOSIDE_SALVAGE	1	0.733273	0.89361703
Upregulated Genes	GO_PURINE_NUCLEOSIDE_MONOPHOSPHATE_BIOSYNTHETIC_PROCESS	1	0.732872	0.8947368
Upregulated Genes	GO_DECIDUALIZATION	1	0.732033	0.917505
Upregulated Genes	GO_POSTTRANSCRIPTIONAL_REGULATION_OF_GENE_EXPRESSION	7	0.73191	0.71647507
Upregulated Genes	GO_REGULATION_OF_RETROGRADE_PROTEIN_TRANSPORT_ER_TO_CYTOSO	1	0.731834	0.8900204
Upregulated Genes	GO_PROTEIN_RETENTION_IN_ER_LUMEN	1	0.73155	0.91020405
Upregulated Genes	GO_AMP_BIOSYNTHETIC_PROCESS	1	0.730476	0.9136842
Upregulated Genes	GO_PURINE_NUCLEOSIDE_BIOSYNTHETIC_PROCESS	1	0.729904	0.90120965
Upregulated Genes	GO_REGULATION_OF_CELLULAR_PH	5	0.729243	0.7246377
Upregulated Genes	GO_NEGATIVE_REGULATION_OF_ERAD_PATHWAY	1	0.729151	0.9217221
Upregulated Genes	GO_MAINTENANCE_OF_PROTEIN_LOCALIZATION_IN_ENDOPLASMIC_RETIC	1	0.727003	0.9007937
Upregulated Genes	GO_MAINTENANCE_OF_PROTEIN_LOCALIZATION_IN_ORGANELLE	1	0.726691	0.9107505
Upregulated Genes	GO_TRANSITION_METAL_ION_TRANSPORT	2	0.726567	0.82441115
Upregulated Genes	GO_RESPONSE_TO_ORGANOPHOSPHORUS	1	0.726143	0.9007937
Upregulated Genes	GO_OXIDATION_REDUCTION_PROCESS	16	0.726037	0.7294333
Upregulated Genes	GO_INTRACELLULAR_RECEPTOR_SIGNALING_PATHWAY	4	0.726027	0.7479839
Upregulated Genes	GO_POSITIVE_REGULATION_OF_CATABOLIC_PROCESS	9	0.725492	0.70406187
Upregulated Genes	GO_EMBRYO_IMPLANTATION	1	0.725183	0.9137931
Upregulated Genes	GO_ANATOMICAL_STRUCTURE_MATURATION	6	0.725089	0.7423935
Upregulated Genes	GO_MATERNAL_PROCESS_INVOLVED_IN_FEMALE_PREGNANCY	1	0.724449	0.9137577
Upregulated Genes	GO_DENDRITE_SELF_AVOIDANCE	1	0.724175	0.9276596
Upregulated Genes	GO_SNRNA_METABOLIC_PROCESS	2	0.724164	0.8164948
Upregulated Genes	GO_LEUKOCYTE_TETHERING_OR_ROLLING	1	0.723499	0.9185336
Upregulated Genes	GO_RESPONSE_TO_FIBROBLAST_GROWTH_FACTOR	3	0.723308	0.77935225
Upregulated Genes	GO_NCRNA_3_END_PROCESSING	2	0.72329	0.8221344
Upregulated Genes	GO_PROTON_TRANSPORTING_V_TYPE_ATPASE_COMPLEX_ASSEMBLY	1	0.723027	0.91566265

Upregulated Genes	GO_MULTI_MULTICELLULAR_ORGANISM_PROCESS	1	0.722952	0.90380764
Upregulated Genes	GO_ROOF_OF_MOUTH_DEVELOPMENT	3	0.7229	0.75206614
Upregulated Genes	GO_MONOVALENT_INORGANIC_CATION_HOMEOSTASIS	5	0.722224	0.75951904
Upregulated Genes	GO_REGULATION_OF_PH	5	0.721315	0.7371541
Upregulated Genes	GO_SNRNA_PROCESSING	2	0.719434	0.8181818
Upregulated Genes	GO_FIBROBLAST_GROWTH_FACTOR_RECEPTOR_SIGNALING_PATHWAY	3	0.719164	0.77342045
Upregulated Genes	GO_IMMUNE_RESPONSE_REGULATING_SIGNALING_PATHWAY	7	0.718892	0.72904485
Upregulated Genes	GO_REGULATION_OF_KIDNEY_DEVELOPMENT	2	0.718272	0.8143712
Upregulated Genes	GO_PEPTIDE_METABOLIC_PROCESS	19	0.718231	0.7358834
Upregulated Genes	GO_CARTILAGE_DEVELOPMENT_INVOLVED_IN_ENDOCHONDRAL_BONE_M	1	0.715801	0.907173
Upregulated Genes	GO_REGULATION_OF_CELL_JUNCTION_ASSEMBLY	5	0.714892	0.74285716
Upregulated Genes	GO_BONE_RESORPTION	1	0.71469	0.93073595
Upregulated Genes	GO_REGULATION_OF_EPITHELIAL_CELL_DIFFERENTIATION	2	0.714469	0.8293651
Upregulated Genes	GO_ORGANIC_ACID_METABOLIC_PROCESS	19	0.713949	0.7490637
Upregulated Genes	GO_RNA_CATABOLIC_PROCESS	6	0.713275	0.7413442
Upregulated Genes	GO_CELLULAR_MONOVALENT_INORGANIC_CATION_HOMEOSTASIS	5	0.713209	0.753937
Upregulated Genes	GO_REGULATION_OF_DEVELOPMENTAL_GROWTH	9	0.713007	0.7024292
Upregulated Genes	GO_POSITIVE_REGULATION_OF_CELLULAR_AMIDE_METABOLIC_PROCESS	4	0.712003	0.7572614
Upregulated Genes	GO_CHONDROCYTE_DEVELOPMENT_INVOLVED_IN_ENDOCHONDRAL_BON	1	0.711266	0.9332024
Upregulated Genes	GO_COAGULATION	6	0.709015	0.7379576
Upregulated Genes	GO_HISTONE_H3_K4_METHYLATION	2	0.708915	0.8471954
Upregulated Genes	GO_BICARBONATE_TRANSPORT	1	0.708129	0.9437751
Upregulated Genes	GO_CHONDROCYTE_DIFFERENTIATION_INVOLVED_IN_ENDOCHONDRAL_B	1	0.70783	0.93227094
Upregulated Genes	GO_DEVELOPMENTAL_GROWTH	20	0.707649	0.7800752
Upregulated Genes	GO_REGULATION_OF_NEURONAL_SYNAPTIC_PLASTICITY	1	0.707499	0.93162394
Upregulated Genes	GO_T_CELL_DIFFERENTIATION	3	0.707396	0.7846482
Upregulated Genes	GO_HYALURONAN_METABOLIC_PROCESS	1	0.70692	0.9444444
Upregulated Genes	GO_NEGATIVE_REGULATION_OF_MOLECULAR_FUNCTION	14	0.706682	0.74121994
Upregulated Genes	GO_NEGATIVE_REGULATION_OF_ORGANELLE_ORGANIZATION	5	0.706496	0.75
Upregulated Genes	GO_PROTEIN_INSERTION_INTO_ER_MEMBRANE	2	0.705415	0.83613443
Upregulated Genes	GO_ENDOCHONDRAL_BONE_MORPHOGENESIS	1	0.704385	0.9437751
Upregulated Genes	GO_INTRACELLULAR_STEROID_HORMONE_RECEPTOR_SIGNALING_PATHW	2	0.704162	0.8141414
Upregulated Genes	GO_SYNAPSE_ASSEMBLY	5	0.703019	0.7698574
Upregulated Genes	GO_RESPONSE_TO_TOPOLOGICALLY_INCORRECT_PROTEIN	5	0.701901	0.79716027
Upregulated Genes	GO_REGULATION_OF_REACTIVE_OXYGEN_SPECIES_METABOLIC_PROCESS	2	0.700804	0.8594705
Upregulated Genes	GO_PLASMA_LIPOPROTEIN_PARTICLE_CLEARANCE	1	0.700623	0.939834

Upregulated Genes	GO_LIPID_METABOLIC_PROCESS	28	0.700567	0.8109966
Upregulated Genes	GO_NUCLEAR_EXPORT	8	0.700305	0.72336066
Upregulated Genes	GO_SRP_DEPENDENT_COTRANSLATIONAL_PROTEIN_TARGETING_TO_MEM	2	0.698268	0.83914727
Upregulated Genes	GO_DEVELOPMENT_OF_PRIMARY_SEXUAL_CHARACTERISTICS	2	0.697603	0.8218263
Upregulated Genes	GO_GLYCEROLIPID_METABOLIC_PROCESS	12	0.697193	0.74647886
Upregulated Genes	GO_POSITIVE_REGULATION_OF_RECEPTOR_MEDIATED_ENDOCYTOSIS	1	0.696919	0.9364754
Upregulated Genes	GO_CHROMATIN_SILENCING	2	0.696647	0.8582996
Upregulated Genes	GO_CLATHRIN_DEPENDENT_ENDOCYTOSIS	1	0.695021	0.9559387
Upregulated Genes	GO_PEPTIDYL_TYROSINE_DEPHOSPHORYLATION	2	0.694226	0.8309859
Upregulated Genes	GO_HEART_DEVELOPMENT	9	0.694004	0.736952
Upregulated Genes	GO_RIBOSOME_BIOGENESIS	4	0.693936	0.80404043
Upregulated Genes	GO_REGULATION_OF_HYALURONAN_BIOSYNTHETIC_PROCESS	1	0.693887	0.9483471
Upregulated Genes	GO_REGULATION_OF_MEGAKARYOCYTE_DIFFERENTIATION	2	0.693498	0.83984375
Upregulated Genes	GO_PROTEIN_INSERTION_INTO_MEMBRANE	2	0.692966	0.8385214
Upregulated Genes	GO_FERTILIZATION	2	0.692016	0.825462
Upregulated Genes	GO_PEPTIDYL_LYSINE_METHYLATION	2	0.691776	0.8661088
Upregulated Genes	GO_REGULATION_OF_PLASMA_LIPOPROTEIN_PARTICLE_LEVELS	1	0.691441	0.9590643
Upregulated Genes	GO_HOMEOSTATIC_PROCESS	29	0.691428	0.79280823
Upregulated Genes	GO_CELL_DIFFERENTIATION_INVOLVED_IN_EMBRYONIC_PLACENTA_DEVEL	1	0.690793	0.9812108
Upregulated Genes	GO_MEGAKARYOCYTE_DIFFERENTIATION	2	0.689709	0.8503119
Upregulated Genes	GO_LOW_DENSITY_LIPOPROTEIN_PARTICLE_CLEARANCE	1	0.689463	0.95519346
Upregulated Genes	GO_CHROMATIN_ORGANIZATION_INVOLVED_IN_REGULATION_OF_TRANS(	2	0.689153	0.8436214
Upregulated Genes	GO_LABYRINTHINE_LAYER_DEVELOPMENT	1	0.686591	0.9588015
Upregulated Genes	GO_GOLGI_TO_ENDOSOME_TRANSPORT	1	0.686501	0.9548872
Upregulated Genes	GO_STEROID_HORMONE_MEDIATED_SIGNALING_PATHWAY	2	0.68619	0.862423
Upregulated Genes	GO_BRANCHING_INVOLVED_IN_LABYRINTHINE_LAYER_MORPHOGENESIS	1	0.686136	0.9676113
Upregulated Genes	GO_EMBRYONIC_PLACENTA_MORPHOGENESIS	1	0.684484	0.98568505
Upregulated Genes	GO_L_SERINE_BIOSYNTHETIC_PROCESS	1	0.683698	0.98964804
Upregulated Genes	GO_POSITIVE_REGULATION_OF_ION_TRANSPORT	4	0.683513	0.79273504
Upregulated Genes	GO_NEGATIVE_REGULATION_OF_MITOCHONDRIAL_FUSION	1	0.682086	0.98054475
Upregulated Genes	GO_HYALURONAN_BIOSYNTHETIC_PROCESS	1	0.681986	0.95151514
Upregulated Genes	GO_VESICLE_MEDIATED_TRANSPORT_IN_SYNAPSE	6	0.681054	0.79
Upregulated Genes	GO_CELL_ACTIVATION_INVOLVED_IN_IMMUNE_RESPONSE	16	0.680856	0.76833975
Upregulated Genes	GO_L_SERINE_METABOLIC_PROCESS	1	0.679614	0.9831144
Upregulated Genes	GO_RESPONSE_TO_ABIOTIC_STIMULUS	18	0.678731	0.78846157
Upregulated Genes	GO_DETECTION_OF_VIRUS	1	0.67837	0.98723406

Upregulated Genes	GO_SERINE_FAMILY_AMINO_ACID_BIOSYNTHETIC_PROCESS	1	0.677964	0.9802371
Upregulated Genes	GO_PHOSPHATIDYLSERINE_METABOLIC_PROCESS	1	0.676386	0.97722566
Upregulated Genes	GO_EMBRYONIC_PLACENTA_DEVELOPMENT	1	0.676168	0.97556007
Upregulated Genes	GO_POSITIVE_REGULATION_OF_MACROAUTOPHAGY	2	0.673317	0.866
Upregulated Genes	GO_CARBOHYDRATE_DERIVATIVE_METABOLIC_PROCESS	33	0.672771	0.8358459
Upregulated Genes	GO_POSITIVE_REGULATION_OF_INTRINSIC_APOPTOTIC_SIGNALING_PATHV	1	0.671727	0.9862745
Upregulated Genes	GO_AMIDE_BIOSYNTHETIC_PROCESS	19	0.67124	0.8018182
Upregulated Genes	GO_LABYRINTHINE_LAYER_MORPHOGENESIS	1	0.669894	0.9801193
Upregulated Genes	GO_POSITIVE_REGULATION_OF_RESPONSE_TO_ENDOPLASMIC_RETICULUN	1	0.668764	0.98841697
Upregulated Genes	GO_CELLULAR_RESPONSE_TO_STEROID_HORMONE_STIMULUS	2	0.668278	0.8842315
Upregulated Genes	GO_HORMONE_MEDIATED_SIGNALING_PATHWAY	3	0.667963	0.8126273
Upregulated Genes	GO_POSITIVE_REGULATION_OF_GLUCOSE_TRANSMEMBRANE_TRANSPORT	2	0.667078	0.8661088
Upregulated Genes	GO_POSITIVE_REGULATION_OF_ENDOPLASMIC_RETICULUM_STRESS_INDU	1	0.66568	0.99159664
Upregulated Genes	GO_NEGATIVE_REGULATION_OF_PROTEIN_LOCALIZATION_TO_CELL_PERIP	2	0.665024	0.88293654
Upregulated Genes	GO_REGULATION_OF_RAS_PROTEIN_SIGNAL_TRANSDUCTION	3	0.664967	0.8229167
Upregulated Genes	GO_ANTIGEN_PROCESSING_AND_PRESENTATION	4	0.661539	0.8255578
Upregulated Genes	GO_CHEMICAL_HOMEOSTASIS	22	0.660468	0.84859157
Upregulated Genes	GO_RESPONSE_TO_HORMONE	11	0.657394	0.80487806
Upregulated Genes	GO_LEUKOCYTE_DIFFERENTIATION	8	0.657375	0.80670613
Upregulated Genes	GO_POSITIVE_REGULATION_OF_PROTEIN_CATABOLIC_PROCESS	5	0.656841	0.80078894
Upregulated Genes	GO_CELLULAR_RESPONSE_TO_TOPOLOGICALLY_INCORRECT_PROTEIN	4	0.654784	0.80728054
Upregulated Genes	GO_REGULATION_OF_BODY_FLUID_LEVELS	7	0.65135	0.8235294
Upregulated Genes	GO_CELLULAR_AMIDE_METABOLIC_PROCESS	22	0.647767	0.84695655
Upregulated Genes	GO_REGULATION_OF_PROTEIN_MATURATION	5	0.646563	0.82669324
Upregulated Genes	GO_GROWTH	25	0.641289	0.8442029
Upregulated Genes	GO_REGULATION_OF_NECROTIC_CELL_DEATH	2	0.636142	0.88912135
Upregulated Genes	GO_FORELIMB_MORPHOGENESIS	2	0.635798	0.89917696
Upregulated Genes	GO_NECROTIC_CELL_DEATH	2	0.635147	0.90140843
Upregulated Genes	GO_FEEDING_BEHAVIOR	2	0.635023	0.9109731
Upregulated Genes	GO_ORGANOPHOSPHATE_BIOSYNTHETIC_PROCESS	14	0.634298	0.80755395
Upregulated Genes	GO_REGULATION_OF_WNT_SIGNALING_PATHWAY	3	0.63342	0.89320385
Upregulated Genes	GO_LIPID_CATABOLIC_PROCESS	3	0.631676	0.8545455
Upregulated Genes	GO_METHYLATION	6	0.631148	0.8277228
Upregulated Genes	GO_MYELOID_LEUKOCYTE_MEDIATED_IMMUNITY	15	0.630508	0.8099631
Upregulated Genes	GO_NEGATIVE_REGULATION_OF_RESPONSE_TO_STIMULUS	23	0.629141	0.8357401
Upregulated Genes	GO_LEUKOCYTE_MEDIATED_IMMUNITY	16	0.628978	0.8113208

Upregulated Genes	GO_REGULATION_OF_BONE_MINERALIZATION	4	0.624825	0.8816326
Upregulated Genes	GO_REGULATION_OF_PROGRAMMED_NECROTIC_CELL_DEATH	2	0.623713	0.90448344
Upregulated Genes	GO_MYELOID_LEUKOCYTE_ACTIVATION	15	0.623318	0.8027344
Upregulated Genes	GO_NEGATIVE_REGULATION_OF_NECROTIC_CELL_DEATH	2	0.62223	0.92083335
Upregulated Genes	GO_EMBRYONIC_FORELIMB_MORPHOGENESIS	2	0.621863	0.91295546
Upregulated Genes	GO_PROTEIN_PROCESSING	6	0.620388	0.83767533
Upregulated Genes	GO_NEGATIVE_REGULATION_OF_EPITHELIAL_CELL_PROLIFERATION	3	0.619434	0.8741722
Upregulated Genes	GO_RNA_PHOSPHODIESTER_BOND_HYDROLYSIS	3	0.619382	0.8957055
Upregulated Genes	GO_BEHAVIOR	11	0.616841	0.83567137
Upregulated Genes	GO_PROGRAMMED_NECROTIC_CELL_DEATH	2	0.61565	0.912766
Upregulated Genes	GO_REGULATION_OF_CYTOSKELETON_ORGANIZATION	8	0.615119	0.8205645
Upregulated Genes	GO_RNA_SPLICING	13	0.615039	0.82129276
Upregulated Genes	GO_REGULATION_OF_HORMONE_LEVELS	6	0.613563	0.83265305
Upregulated Genes	GO_REGULATION_OF_GROWTH	14	0.611116	0.8586066
Upregulated Genes	GO_POSITIVE_REGULATION_OF_PROTEOLYSIS	4	0.609848	0.8649789
Upregulated Genes	GO_CARBOHYDRATE_METABOLIC_PROCESS	12	0.602106	0.816
Upregulated Genes	GO_POSITIVE_REGULATION_OF_ESTABLISHMENT_OF_PROTEIN_LOCALIZAT	6	0.600593	0.85375494
Upregulated Genes	GO_NEGATIVE_REGULATION_OF_CELL_ACTIVATION	2	0.600397	0.9237473
Upregulated Genes	GO_TRANSMEMBRANE_TRANSPORT	31	0.599092	0.88505745
Upregulated Genes	GO_REGULATION_OF_OSSIFICATION	7	0.597431	0.85996056
Upregulated Genes	GO_NEGATIVE_REGULATION_OF_LEUKOCYTE_CELL_CELL_ADHESION	2	0.597012	0.93153524
Upregulated Genes	GO_COVALENT_CHROMATIN_MODIFICATION	6	0.592314	0.8580247
Upregulated Genes	GO_CELLULAR_RESPONSE_TO_HORMONE_STIMULUS	7	0.590013	0.8862745
Upregulated Genes	GO_POSITIVE_REGULATION_OF_CELLULAR_COMPONENT_BIOGENESIS	9	0.589847	0.85388994
Upregulated Genes	GO_NEGATIVE_REGULATION_OF_CELL_CELL_ADHESION	2	0.589145	0.94693875
Upregulated Genes	GO_NEGATIVE_REGULATION_OF_FIBROBLAST_PROLIFERATION	2	0.588852	0.925636
Upregulated Genes	GO_NUCLEOBASE_CONTAINING_COMPOUND_TRANSPORT	11	0.586456	0.85921323
Upregulated Genes	GO_SUPRAMOLECULAR_FIBER_ORGANIZATION	14	0.586079	0.8444882
Upregulated Genes	GO_CARBOHYDRATE_DERIVATIVE_TRANSPORT	4	0.586041	0.8893443
Upregulated Genes	GO_RESPONSE_TO_METAL_ION	10	0.585916	0.852552
Upregulated Genes	GO_REGULATION_OF_VESICLE_MEDIATED_TRANSPORT	12	0.585018	0.8528864
Upregulated Genes	GO_NEGATIVE_REGULATION_OF_PHOSPHORUS_METABOLIC_PROCESS	6	0.583821	0.8965517
Upregulated Genes	GO_REGULATION_OF_ION_TRANSMEMBRANE_TRANSPORT	7	0.5826	0.84934086
Upregulated Genes	GO_LYMPHOCYTE_ACTIVATION	11	0.580124	0.84479374
Upregulated Genes	GO_FIBROBLAST_PROLIFERATION	2	0.580035	0.9561753
Upregulated Genes	GO_CARBOHYDRATE_HOMEOSTASIS	5	0.579827	0.9029703

Upregulated Genes	GO_NEGATIVE_REGULATION_OF_LYMPHOCYTE_ACTIVATION	2	0.579296	0.9497908
Upregulated Genes	GO_NEGATIVE_REGULATION_OF_CELL_ADHESION	2	0.577983	0.954
Upregulated Genes	GO_LYMPHOCYTE_DIFFERENTIATION	5	0.576075	0.8994083
Upregulated Genes	GO_REGULATION_OF_PROTEIN_DEPHOSPHORYLATION	2	0.572972	0.9381663
Upregulated Genes	GO_REGULATION_OF_PHOSPHOPROTEIN_PHOSPHATASE_ACTIVITY	2	0.572843	0.93787575
Upregulated Genes	GO_REGULATION_OF_PHOSPHOLIPID_METABOLIC_PROCESS	3	0.570878	0.9199179
Upregulated Genes	GO_POSITIVE_REGULATION_OF_DEFENSE_RESPONSE	2	0.570146	0.9517103
Upregulated Genes	GO_NEGATIVE_REGULATION_OF_PROTEIN_METABOLIC_PROCESS	14	0.568129	0.8782435
Upregulated Genes	GO_REGULATION_OF_PROTEOLYSIS	13	0.564679	0.8929293
Upregulated Genes	GO_PROTEIN_MATURATION	7	0.56374	0.89453125
Upregulated Genes	GO_CARDIAC_SEPTUM_MORPHOGENESIS	3	0.563389	0.91775703
Upregulated Genes	GO_INORGANIC_ANION_TRANSMEMBRANE_TRANSPORT	2	0.561627	0.9395248
Upregulated Genes	GO_LEUKOCYTE_PROLIFERATION	5	0.561434	0.9191489
Upregulated Genes	GO_PROTEIN_POLYUBIQUITINATION	5	0.561025	0.92479676
Upregulated Genes	GO_T_CELL_PROLIFERATION	5	0.559286	0.9229287
Upregulated Genes	GO_CELLULAR_COMPONENT_DISASSEMBLY	9	0.558227	0.8910891
Upregulated Genes	GO_CARDIAC_SEPTUM_DEVELOPMENT	3	0.55819	0.9248927
Upregulated Genes	GO_REGULATION_OF_PHOSPHATASE_ACTIVITY	3	0.555892	0.9510638
Upregulated Genes	GO_CHLORIDE_TRANSPORT	2	0.555288	0.966457
Upregulated Genes	GO_REGULATION_OF_DEPHOSPHORYLATION	3	0.55459	0.9404255
Upregulated Genes	GO_ENZYME_LINKED_RECEPTOR_PROTEIN_SIGNALING_PATHWAY	25	0.543243	0.91519433
Upregulated Genes	GO_REGULATION_OF_GTPASE_ACTIVITY	7	0.539768	0.90816325
Upregulated Genes	GO_CELLULAR_RESPONSE_TO_PEPTIDE	5	0.539171	0.92105263
Upregulated Genes	GO_REGULATION_OF_LIPID_METABOLIC_PROCESS	9	0.536447	0.9085821
Upregulated Genes	GO_GENE_SILENCING	3	0.536236	0.9580838
Upregulated Genes	GO_CARBOHYDRATE_TRANSPORT	5	0.535099	0.941048
Upregulated Genes	GO_GLIAL_CELL_DEVELOPMENT	4	0.533409	0.96066254
Upregulated Genes	GO_ANION_TRANSPORT	11	0.533389	0.89668614
Upregulated Genes	GO_RESPONSE_TO_INORGANIC_SUBSTANCE	14	0.530444	0.9007491
Upregulated Genes	GO_T_CELL_ACTIVATION	9	0.529969	0.92730844
Upregulated Genes	GO_UROGENITAL_SYSTEM_DEVELOPMENT	10	0.525063	0.9158317
Upregulated Genes	GO_BONE_MINERALIZATION	5	0.524318	0.93762994
Upregulated Genes	GO_RESPONSE_TO_PEPTIDE_HORMONE	5	0.524075	0.9453781
Upregulated Genes	GO_REGULATION_OF_DNA_BINDING_TRANSCRIPTION_FACTOR_ACTIVITY	6	0.523047	0.9089069
Upregulated Genes	GO_ORGANONITROGEN_COMPOUND_BIOSYNTHETIC_PROCESS	50	0.515442	0.9644588
Upregulated Genes	GO_BONE_MORPHOGENESIS	5	0.514399	0.96162045

Upregulated Genes	GO_MACROMOLECULE_CATABOLIC_PROCESS	27	0.513563	0.9244288
Upregulated Genes	GO_CELLULAR_RESPONSE_TO_INSULIN_STIMULUS	4	0.513414	0.95427436
Upregulated Genes	GO_COLLAGEN_METABOLIC_PROCESS	5	0.513252	0.94351465
Upregulated Genes	GO_WATER_SOLUBLE_VITAMIN_METABOLIC_PROCESS	2	0.511959	0.9811321
Upregulated Genes	GO_MONOSACCHARIDE_METABOLIC_PROCESS	7	0.510337	0.936255
Upregulated Genes	GO_REGULATION_OF_MRNA_METABOLIC_PROCESS	4	0.510323	0.956
Upregulated Genes	GO_FC_RECEPTOR_MEDIATED_STIMULATORY_SIGNALING_PATHWAY	2	0.504879	0.9851064
Upregulated Genes	GO_VITAMIN_METABOLIC_PROCESS	2	0.504169	0.9778226
Upregulated Genes	GO_CELLULAR_RESPONSE_TO_PEPTIDE_HORMONE_STIMULUS	4	0.503901	0.9659319
Upregulated Genes	GO_RESPONSE_TO_INSULIN	4	0.503126	0.96875
Upregulated Genes	GO_REGULATION_OF_TRANS_SYNAPTIC_SIGNALING	10	0.498267	0.9476744
Upregulated Genes	GO_POSITIVE_REGULATION_OF_DNA_BINDING_TRANSCRIPTION_FACTOR_/	4	0.494086	0.975048
Upregulated Genes	GO_REGULATION_OF_SYNAPTIC_VESICLE_ENDOCYTOSIS	3	0.493817	0.972973
Upregulated Genes	GO_LIPOPROTEIN_BIOSYNTHETIC_PROCESS	5	0.492206	0.96487606
Upregulated Genes	GO_LIPOPROTEIN_METABOLIC_PROCESS	5	0.490851	0.97137403
Upregulated Genes	GO_REGULATION_OF_SMALL_MOLECULE_METABOLIC_PROCESS	7	0.490672	0.93426293
Upregulated Genes	GO_REGULATION_OF_CELLULAR_LOCALIZATION	21	0.489928	0.93333334
Upregulated Genes	GO_MEMORY	2	0.48961	0.99598396
Upregulated Genes	GO_NEGATIVE_REGULATION_OF_IMMUNE_EFFECTOR_PROCESS	2	0.489115	0.98767966
Upregulated Genes	GO_REGULATION_OF_SYNAPTIC_VESICLE_RECYCLING	3	0.488692	0.97052634
Upregulated Genes	GO_NEGATIVE_REGULATION_OF_IMMUNE_RESPONSE	2	0.486031	0.9980198
Upregulated Genes	GO_REGULATION_OF_TRANSMEMBRANE_TRANSPORT	11	0.484187	0.95841587
Upregulated Genes	GO_REGULATION_OF_CELL_KILLING	2	0.48247	0.99591005
Upregulated Genes	GO_CELLULAR_RESPONSE_TO_LIPID	7	0.481133	0.9653768
Upregulated Genes	GO_ORGANIC_ANION_TRANSPORT	8	0.478485	0.95183045
Upregulated Genes	GO_APPENDAGE_MORPHOGENESIS	6	0.474563	0.9700934
Upregulated Genes	GO_NEGATIVE_REGULATION_OF_SIGNALING	18	0.474236	0.9415094
Upregulated Genes	GO_PEPTIDYL_LYSINE_MODIFICATION	7	0.471652	0.9588235
Upregulated Genes	GO_REGULATION_OF_ORGANELLE_ORGANIZATION	19	0.469636	0.94049907
Upregulated Genes	GO_ACTIN_FILAMENT_ORGANIZATION	6	0.467892	0.9561753
Upregulated Genes	GO_NUCLEUS_ORGANIZATION	2	0.461349	0.9957537
Upregulated Genes	GO_EMBRYONIC_APPENDAGE_MORPHOGENESIS	6	0.450001	0.9742063
Upregulated Genes	GO_APPENDAGE_DEVELOPMENT	6	0.444664	0.9815951
Upregulated Genes	GO_CYTOSKELETON_ORGANIZATION	20	0.444656	0.97416973
Upregulated Genes	GO_SMALL_MOLECULE_BIOSYNTHETIC_PROCESS	13	0.43836	0.967803
Upregulated Genes	GO_CELLULAR_RESPONSE_TO_CALCIUM_ION	3	0.43696	0.98977506

Upregulated Genes	GO_NEGATIVE_REGULATION_OF_VASCULATURE_DEVELOPMENT	2	0.435351	1
Upregulated Genes	GO_REGULATION_OF_PROTEIN_TARGETING	3	0.417428	1
Upregulated Genes	GO_POSITIVE_REGULATION_OF_TRANSPORT	19	0.415262	0.9704251
Upregulated Genes	GO_NEGATIVE_REGULATION_OF_PROTEIN_MATURATION	4	0.410734	0.98995984
Upregulated Genes	GO_TRANSMEMBRANE_RECEPTOR_PROTEIN_TYROSINE_KINASE_SIGNALIN(	18	0.400444	0.98571426
Upregulated Genes	GO_ACTIN_FILAMENT_BASED_PROCESS	12	0.386825	0.99067163
Upregulated Genes	GO_POSITIVE_REGULATION_OF_CELLULAR_COMPONENT_ORGANIZATION	27	0.376341	0.989899
Upregulated Genes	GO_PROTEOLYSIS	31	0.370545	0.98307955
Upregulated Genes	GO_REGULATION_OF_CATABOLIC_PROCESS	20	0.367388	0.990991
Upregulated Genes	GO_REGULATION_OF_CELLULAR_CATABOLIC_PROCESS	16	0.362556	0.9963964
Upregulated Genes	GO_ORGANOPHOSPHATE_METABOLIC_PROCESS	24	0.33103	1
Upregulated Genes	GO_GENERATION_OF_PRECURSOR_METABOLITES_AND_ENERGY	12	0.318943	0.9961612
