# Supplementary material for: Estrogen Receptor-Regulated Gene Signatures in Invasive Breast Cancer Cells and Aggressive Breast Tumors
Source: Cancers (Basel). 2022 Jun 9;14(12):2848. doi: 10.3390/cancers14122848 (PMC9221274; doi:10.3390/cancers14122848)
Supplement: Supplementary file 1 [file cancers-14-02848-s001.zip › Table S5.pdf]

**Table S5. Differentially expressed genes for each cluster identified in the scRNA-seq dataset derived from invasive cells.**

| gene | p_val | avg_logFC | pct.1 | pct.2 | p_val_adj | cluster |
|---|---|---|---|---|---|---|
| KRT80 | 1.98E-25 | 0.60270402 | 0.959 | 0.773 | 4.08E-21 | 0 |
| HSPB1 | 4.37E-25 | 0.50777804 | 1 | 1 | 9.01E-21 | 0 |
| HSPB1P1 | 1.88E-24 | 0.53744161 | 1 | 0.994 | 3.88E-20 | 0 |
| HSPB1P2 | 5.42E-24 | 0.5440248 | 1 | 0.982 | 1.12E-19 | 0 |
| ACTN1 | 1.32E-22 | 0.53837403 | 1 | 0.966 | 2.72E-18 | 0 |
| MYADM | 2.12E-21 | 0.55453201 | 0.848 | 0.506 | 4.37E-17 | 0 |
| MYL12A | 1.34E-18 | 0.33247382 | 1 | 0.985 | 2.76E-14 | 0 |
| EDN1 | 1.59E-16 | 0.46026932 | 0.558 | 0.199 | 3.27E-12 | 0 |
| TUFT1 | 1.29E-14 | 0.41801843 | 0.677 | 0.362 | 2.66E-10 | 0 |
| CNN2 | 9.01E-14 | 0.42046517 | 0.899 | 0.724 | 1.86E-09 | 0 |
| AREG | 1.31E-13 | 0.60362054 | 0.521 | 0.233 | 2.70E-09 | 0 |
| VCL | 2.40E-13 | 0.38365857 | 0.765 | 0.503 | 4.94E-09 | 0 |
| TPM1 | 9.13E-13 | 0.36042726 | 0.995 | 0.945 | 1.88E-08 | 0 |
| ZYX | 4.57E-12 | 0.30156484 | 0.829 | 0.571 | 9.43E-08 | 0 |
| PRSS23 | 6.04E-12 | 0.47471609 | 0.581 | 0.304 | 1.25E-07 | 0 |
| FHL2 | 1.62E-11 | 0.34069352 | 0.475 | 0.202 | 3.35E-07 | 0 |
| PDLIM5 | 1.69E-11 | 0.37368943 | 0.71 | 0.436 | 3.49E-07 | 0 |
| ACTG2 | 2.56E-11 | 0.27113299 | 0.281 | 0.067 | 5.29E-07 | 0 |
| MACC1 | 1.15E-10 | 0.23547647 | 0.327 | 0.104 | 2.37E-06 | 0 |
| TPM4 | 1.53E-10 | 0.30376011 | 0.945 | 0.868 | 3.16E-06 | 0 |
| THBS1 | 1.78E-10 | 0.38669512 | 0.664 | 0.393 | 3.66E-06 | 0 |
| STK38L | 6.85E-10 | 0.30395276 | 0.382 | 0.16 | 1.41E-05 | 0 |
| CSRP1 | 1.76E-09 | 0.26612969 | 0.585 | 0.319 | 3.64E-05 | 0 |
| S100A10 | 2.37E-09 | 0.41121727 | 0.982 | 0.939 | 4.88E-05 | 0 |
| AMOTL2 | 3.10E-09 | 0.20044924 | 0.512 | 0.236 | 6.40E-05 | 0 |
| FLNB | 5.42E-09 | 0.24083799 | 0.507 | 0.258 | 0.000112 | 0 |
| FLNA | 5.68E-09 | 0.2773926 | 0.959 | 0.871 | 0.000117 | 0 |
| ERRFI1 | 5.73E-09 | 0.43557616 | 0.479 | 0.255 | 0.000118 | 0 |
| FAM84B | 7.14E-09 | 0.30597968 | 0.691 | 0.445 | 0.000147 | 0 |
| SRI | 9.47E-09 | 0.31635033 | 0.627 | 0.408 | 0.000196 | 0 |
| TACSTD2 | 1.18E-08 | 0.50748057 | 0.857 | 0.702 | 0.000244 | 0 |
| CAV2 | 1.54E-08 | 0.24437997 | 0.355 | 0.15 | 0.000319 | 0 |
| SLC39A14 | 1.67E-08 | 0.22644222 | 0.705 | 0.472 | 0.000345 | 0 |
| ATP5G1 | 2.11E-08 | 0.27154112 | 0.931 | 0.791 | 0.000435 | 0 |
| EPPK1 | 2.45E-08 | 0.29673643 | 0.576 | 0.353 | 0.000506 | 0 |
| S100A11 | 2.46E-08 | 0.22106617 | 0.991 | 0.972 | 0.000508 | 0 |
| PALLD | 2.95E-08 | 0.28897004 | 0.488 | 0.267 | 0.000609 | 0 |
| FDPS | 3.75E-08 | 0.32443729 | 0.94 | 0.822 | 0.000775 | 0 |
| ANXA3 | 4.12E-08 | 0.28446918 | 0.853 | 0.693 | 0.000851 | 0 |
| MYL6 | 5.64E-08 | 0.23214713 | 0.982 | 0.951 | 0.001164 | 0 |
| AC098614. | 8.30E-08 | 0.27446399 | 0.829 | 0.666 | 0.001712 | 0 |
| NDUFB9 | 1.02E-07 | 0.21643071 | 0.977 | 0.905 | 0.002107 | 0 |
| STARD5 | 1.02E-07 | 0.21059894 | 0.604 | 0.371 | 0.002108 | 0 |
| LOXL2 | 1.28E-07 | 0.27756936 | 0.576 | 0.344 | 0.00265 | 0 |

| | | | | | | |
|---|---|---|---|---|---|---|
| ATP5G3 | 2.60E-07 | 0.21163667 | 0.977 | 0.923 | 0.005374 | 0 |
| RP11-2C24 | 3.62E-07 | 0.16258016 | 0.346 | 0.153 | 0.007463 | 0 |
| FERMT2 | 3.76E-07 | 0.22955338 | 0.567 | 0.365 | 0.007761 | 0 |
| MYH9 | 3.79E-07 | 0.27080964 | 0.959 | 0.89 | 0.007829 | 0 |
| MALAT1 | 4.49E-07 | 0.37566309 | 0.926 | 0.77 | 0.009273 | 0 |
| GPRC5A | 4.52E-07 | 0.30172296 | 0.493 | 0.294 | 0.009334 | 0 |
| RBMX2P5 | 4.66E-07 | 0.18480195 | 0.424 | 0.218 | 0.009618 | 0 |
| RP9 | 5.63E-07 | 0.1701559 | 0.475 | 0.258 | 0.01162 | 0 |
| ACTN4 | 6.34E-07 | 0.2146008 | 0.954 | 0.911 | 0.013087 | 0 |
| NUAK2 | 7.06E-07 | 0.33990587 | 0.396 | 0.209 | 0.014562 | 0 |
| RP11-15K1 | 7.63E-07 | 0.21804921 | 0.512 | 0.301 | 0.015738 | 0 |
| ITGB1 | 1.03E-06 | 0.25886739 | 0.954 | 0.874 | 0.021223 | 0 |
| GS1-44D20 | 1.16E-06 | 0.21886301 | 0.512 | 0.313 | 0.023845 | 0 |
| CLDN4 | 1.16E-06 | 0.25813864 | 0.959 | 0.813 | 0.023953 | 0 |
| RP11-69L1 | 1.41E-06 | 0.19555268 | 0.788 | 0.555 | 0.029145 | 0 |
| GBP2 | 1.50E-06 | 0.17067151 | 0.336 | 0.16 | 0.030996 | 0 |
| AC012354. | 1.68E-06 | 0.19389656 | 0.802 | 0.632 | 0.034777 | 0 |
| SLIRP | 1.91E-06 | 0.21783413 | 0.917 | 0.791 | 0.03938 | 0 |
| AZIN1 | 1.91E-06 | 0.19347316 | 0.885 | 0.77 | 0.039466 | 0 |
| COTL1 | 1.96E-06 | 0.18700825 | 0.691 | 0.475 | 0.040472 | 0 |
| SLC7A11 | 1.96E-06 | 0.24568031 | 0.714 | 0.518 | 0.040516 | 0 |
| SBDS | 2.02E-06 | 0.18481932 | 0.945 | 0.862 | 0.041624 | 0 |
| PDLIM7 | 2.04E-06 | 0.18743413 | 0.677 | 0.463 | 0.042073 | 0 |
| MIR4435-2 | 2.45E-06 | 0.257993 | 0.885 | 0.791 | 0.050493 | 0 |
| PRNP | 3.19E-06 | 0.1952738 | 0.516 | 0.316 | 0.065931 | 0 |
| RTN4 | 3.52E-06 | 0.20691521 | 0.968 | 0.896 | 0.072598 | 0 |
| RPL22L1 | 3.71E-06 | 0.20529958 | 0.949 | 0.899 | 0.07664 | 0 |
| LCP1 | 3.73E-06 | 0.19448698 | 0.406 | 0.224 | 0.077039 | 0 |
| TRIO | 3.83E-06 | 0.23974889 | 0.7 | 0.503 | 0.078989 | 0 |
| COX6A1 | 3.99E-06 | 0.1944813 | 0.972 | 0.908 | 0.082416 | 0 |
| TIMP3 | 4.58E-06 | 0.17482752 | 0.281 | 0.126 | 0.094572 | 0 |
| RP11-201K | 5.03E-06 | 0.19154017 | 0.889 | 0.807 | 0.103831 | 0 |
| KRT18 | 5.21E-06 | 0.18165626 | 0.88 | 0.755 | 0.107459 | 0 |
| SNRPF | 5.21E-06 | 0.20325272 | 0.894 | 0.822 | 0.10756 | 0 |
| MRPS17 | 5.64E-06 | 0.1936331 | 0.59 | 0.402 | 0.116502 | 0 |
| KRT7 | 6.26E-06 | 0.22428053 | 0.82 | 0.663 | 0.129298 | 0 |
| PSMA6P1 | 6.57E-06 | 0.19385842 | 0.691 | 0.479 | 0.135637 | 0 |
| ACTG1 | 7.20E-06 | 0.13124346 | 1 | 1 | 0.148624 | 0 |
| PDCD11 | 7.79E-06 | 0.12017325 | 0.323 | 0.15 | 0.160865 | 0 |
| CYCSP45 | 8.36E-06 | 0.17474589 | 0.479 | 0.285 | 0.17257 | 0 |
| ENC1 | 8.44E-06 | 0.21426273 | 0.553 | 0.347 | 0.174202 | 0 |
| SSBP1 | 9.62E-06 | 0.18982072 | 0.926 | 0.761 | 0.198644 | 0 |
| U2AF1 | 9.94E-06 | 0.18283543 | 0.788 | 0.647 | 0.205049 | 0 |
| CYR61 | 1.00E-05 | 0.21184799 | 0.594 | 0.383 | 0.206478 | 0 |
| MGLL | 1.03E-05 | 0.25566124 | 0.654 | 0.442 | 0.21251 | 0 |
| RBMX2 | 1.13E-05 | 0.16788439 | 0.479 | 0.282 | 0.233523 | 0 |
| NRP1 | 1.18E-05 | 0.20995212 | 0.585 | 0.393 | 0.243205 | 0 |

| | | | | | | |
|---|---|---|---|---|---|---|
| AJUBA | 1.18E-05 | 0.25003407 | 0.562 | 0.383 | 0.243837 | 0 |
| RHEB | 1.22E-05 | 0.17378773 | 0.866 | 0.696 | 0.25275 | 0 |
| SLC31A2 | 1.30E-05 | 0.18662735 | 0.442 | 0.261 | 0.268288 | 0 |
| HACD2 | 1.39E-05 | 0.17518352 | 0.664 | 0.46 | 0.287358 | 0 |
| SLC2A1 | 1.49E-05 | 0.20894831 | 0.535 | 0.337 | 0.307336 | 0 |
| MYL12B | 1.72E-05 | 0.13128633 | 1 | 0.994 | 0.35469 | 0 |
| IFITM3 | 1.89E-05 | 0.17907319 | 0.977 | 0.933 | 0.389288 | 0 |
| DUSP4 | 1.93E-05 | 0.23509918 | 0.659 | 0.491 | 0.397821 | 0 |
| ENDOD1 | 2.11E-05 | 0.12615162 | 0.286 | 0.135 | 0.435881 | 0 |
| FDPSP5 | 2.22E-05 | 0.17423726 | 0.355 | 0.199 | 0.459148 | 0 |
| RP11-403P | 2.33E-05 | 0.19612237 | 0.829 | 0.709 | 0.481095 | 0 |
| PFDN2 | 2.49E-05 | 0.22029673 | 0.567 | 0.399 | 0.513541 | 0 |
| EEF1B2P6 | 2.60E-05 | 0.13924212 | 0.995 | 0.975 | 0.535933 | 0 |
| IRS2 | 2.62E-05 | 0.19222599 | 0.456 | 0.285 | 0.541159 | 0 |
| LIMS1 | 3.29E-05 | 0.19856694 | 0.562 | 0.365 | 0.67962 | 0 |
| PDCD5 | 3.37E-05 | 0.16367544 | 0.843 | 0.702 | 0.695473 | 0 |
| LDLR | 3.47E-05 | 0.21171281 | 0.659 | 0.475 | 0.716299 | 0 |
| CAPN12 | 3.77E-05 | 0.1905074 | 0.76 | 0.632 | 0.777945 | 0 |
| IER3 | 3.82E-05 | 0.20209814 | 0.88 | 0.684 | 0.788612 | 0 |
| C22orf29 | 4.40E-05 | 0.11215488 | 0.318 | 0.16 | 0.907472 | 0 |
| SLC7A5 | 4.47E-05 | 0.14650688 | 0.931 | 0.828 | 0.923477 | 0 |
| NOL7 | 4.51E-05 | 0.17016873 | 0.751 | 0.564 | 0.930857 | 0 |
| MYO1B | 4.72E-05 | 0.27244577 | 0.677 | 0.512 | 0.97367 | 0 |
| PRPF38B | 5.30E-05 | 0.16551241 | 0.535 | 0.347 | 1 | 0 |
| ATP2B1 | 5.40E-05 | 0.23354069 | 0.627 | 0.454 | 1 | 0 |
| HSPB11 | 5.44E-05 | 0.16991268 | 0.475 | 0.301 | 1 | 0 |
| CTD-2287C | 6.28E-05 | 0.13976241 | 0.991 | 0.979 | 1 | 0 |
| SMG1P3 | 6.30E-05 | 0.10631528 | 0.438 | 0.239 | 1 | 0 |
| CYTOR | 6.36E-05 | 0.19004534 | 0.839 | 0.733 | 1 | 0 |
| KIAA1217 | 6.67E-05 | 0.13113545 | 0.401 | 0.233 | 1 | 0 |
| HSP90B2P | 6.91E-05 | 0.09659873 | 0.286 | 0.141 | 1 | 0 |
| ACTR2 | 6.94E-05 | 0.21639249 | 0.908 | 0.84 | 1 | 0 |
| CD9 | 7.03E-05 | 0.20537712 | 0.853 | 0.693 | 1 | 0 |
| TIPARP | 7.68E-05 | 0.17515683 | 0.493 | 0.316 | 1 | 0 |
| ETS2 | 7.78E-05 | 0.12197429 | 0.263 | 0.126 | 1 | 0 |
| RP11-561B | 7.87E-05 | 0.16650321 | 0.903 | 0.801 | 1 | 0 |
| B4GALT1 | 7.94E-05 | 0.13249922 | 0.608 | 0.429 | 1 | 0 |
| NME1 | 8.78E-05 | 0.17197014 | 0.931 | 0.877 | 1 | 0 |
| DDX18 | 8.81E-05 | 0.19432683 | 0.756 | 0.598 | 1 | 0 |
| RPL22P13 | 8.85E-05 | 0.16027102 | 0.912 | 0.847 | 1 | 0 |
| SMG1P4 | 9.43E-05 | 0.13031789 | 0.438 | 0.27 | 1 | 0 |
| TMEM265 | 0.0001 | 0.13468263 | 0.484 | 0.307 | 1 | 0 |
| HMGCS1 | 0.000106 | 0.20160659 | 0.71 | 0.518 | 1 | 0 |
| CACNG4 | 0.000109 | 0.12528968 | 0.369 | 0.209 | 1 | 0 |
| BRIP1 | 0.000109 | 0.13406628 | 0.419 | 0.252 | 1 | 0 |
| RPL26L1 | 0.000109 | 0.12454141 | 0.581 | 0.396 | 1 | 0 |
| RP11-152C | 0.00011 | 0.07327853 | 0.359 | 0.19 | 1 | 0 |

| | | | | | | |
|---|---|---|---|---|---|---|
| SRSF7 | 0.00011 | 0.23673859 | 0.733 | 0.583 | 1 | 0 |
| WSB1 | 0.000118 | 0.08629853 | 0.465 | 0.282 | 1 | 0 |
| P3H4 | 0.000126 | 0.1263947 | 0.392 | 0.224 | 1 | 0 |
| LYAR | 0.000129 | 0.17255957 | 0.604 | 0.433 | 1 | 0 |
| KRTCAP2 | 0.000129 | 0.18595635 | 0.862 | 0.767 | 1 | 0 |
| CAP1 | 0.000134 | 0.19633653 | 0.889 | 0.794 | 1 | 0 |
| GTF2H5 | 0.000138 | 0.1109109 | 0.415 | 0.248 | 1 | 0 |
| RP11-219A | 0.000138 | 0.11228587 | 0.369 | 0.212 | 1 | 0 |
| MCL1 | 0.000144 | 0.175148 | 0.834 | 0.681 | 1 | 0 |
| S100A8 | 0.000146 | 0.31884806 | 0.585 | 0.426 | 1 | 0 |
| KLF6 | 0.000151 | 0.36119224 | 0.438 | 0.294 | 1 | 0 |
| PAWR | 0.000166 | 0.22983912 | 0.77 | 0.635 | 1 | 0 |
| DAD1 | 0.000174 | 0.14097272 | 0.576 | 0.393 | 1 | 0 |
| NDUFC2 | 0.000176 | 0.15569917 | 0.926 | 0.745 | 1 | 0 |
| RND3 | 0.000178 | 0.19200315 | 0.806 | 0.653 | 1 | 0 |
| MAP3K20 | 0.00018 | 0.22700099 | 0.751 | 0.632 | 1 | 0 |
| TM4SF19-T | 0.000188 | 0.12824719 | 0.488 | 0.322 | 1 | 0 |
| GTF2A2 | 0.000198 | 0.14111232 | 0.728 | 0.583 | 1 | 0 |
| SERTAD2 | 0.000201 | 0.20505925 | 0.71 | 0.558 | 1 | 0 |
| ABRACL | 0.000208 | 0.17488575 | 0.834 | 0.687 | 1 | 0 |
| EMC6 | 0.000219 | 0.15351617 | 0.719 | 0.531 | 1 | 0 |
| RBM8B | 0.000235 | 0.16096291 | 0.811 | 0.644 | 1 | 0 |
| SDHB | 0.000244 | 0.12356309 | 0.535 | 0.362 | 1 | 0 |
| PIN4 | 0.000254 | 0.11669181 | 0.705 | 0.509 | 1 | 0 |
| ZDHHC7 | 0.000271 | 0.16937652 | 0.479 | 0.322 | 1 | 0 |
| INHBB | 0.000279 | 0.14241981 | 0.359 | 0.215 | 1 | 0 |
| DSTN | 0.00028 | 0.19471588 | 0.959 | 0.899 | 1 | 0 |
| TMEM267 | 0.000284 | 0.12849548 | 0.323 | 0.181 | 1 | 0 |
| SNRPB2 | 0.000289 | 0.15764948 | 0.756 | 0.583 | 1 | 0 |
| CRIM1 | 0.000292 | 0.17981096 | 0.733 | 0.571 | 1 | 0 |
| RP11-2C24 | 0.000295 | 0.10726408 | 0.553 | 0.365 | 1 | 0 |
| TES | 0.000298 | 0.15064456 | 0.341 | 0.196 | 1 | 0 |
| TATDN1 | 0.000336 | 0.15285582 | 0.857 | 0.742 | 1 | 0 |
| KRT8 | 0.000346 | 0.14355722 | 0.922 | 0.791 | 1 | 0 |
| SMG1P5 | 0.000361 | 0.13344912 | 0.452 | 0.298 | 1 | 0 |
| GPATCH4 | 0.000366 | 0.11237499 | 0.604 | 0.451 | 1 | 0 |
| HSPA5 | 0.000381 | 0.11407682 | 0.853 | 0.727 | 1 | 0 |
| RPP40 | 0.000386 | 0.12777778 | 0.452 | 0.285 | 1 | 0 |
| WWTR1 | 0.000388 | 0.17496124 | 0.378 | 0.242 | 1 | 0 |
| WDR82 | 0.000393 | 0.09614282 | 0.465 | 0.291 | 1 | 0 |
| SRF | 0.000403 | 0.14466477 | 0.488 | 0.328 | 1 | 0 |
| RP11-72B4 | 0.000404 | 0.12099973 | 0.392 | 0.239 | 1 | 0 |
| SMG1P2 | 0.000412 | 0.13146473 | 0.41 | 0.258 | 1 | 0 |
| FAUP1 | 0.000413 | 0.14748112 | 0.71 | 0.567 | 1 | 0 |
| RPL26P30 | 0.000414 | 0.12080814 | 0.355 | 0.209 | 1 | 0 |
| CDH3 | 0.000417 | 0.12821032 | 0.525 | 0.374 | 1 | 0 |
| UCHL3 | 0.000421 | 0.14314908 | 0.779 | 0.61 | 1 | 0 |

| | | | | | | |
|---|---|---|---|---|---|---|
| ILK | 0.000439 | 0.13013497 | 0.369 | 0.218 | 1 | 0 |
| CKLF-CMTM | 0.000441 | 0.08832841 | 0.355 | 0.209 | 1 | 0 |
| RP11-345J4 | 0.000456 | 0.15398868 | 0.645 | 0.5 | 1 | 0 |
| PCK2 | 0.000473 | 0.15945935 | 0.585 | 0.399 | 1 | 0 |
| PRDX5 | 0.000482 | 0.15636805 | 0.691 | 0.528 | 1 | 0 |
| CBWD1 | 0.000488 | 0.12997549 | 0.802 | 0.641 | 1 | 0 |
| KIF5B | 0.000496 | 0.14224367 | 0.945 | 0.874 | 1 | 0 |
| SMG1P6 | 0.000512 | 0.11269697 | 0.387 | 0.236 | 1 | 0 |
| RPS23P8 | 0.000518 | 0.072958 | 1 | 1 | 1 | 0 |
| RPS13 | 0.00053 | 0.06897061 | 0.995 | 1 | 1 | 0 |
| MSMO1 | 0.000546 | 0.10051554 | 0.47 | 0.313 | 1 | 0 |
| GALNT6 | 0.000546 | 0.09466202 | 0.502 | 0.34 | 1 | 0 |
| NDUFC2-KC | 0.000559 | 0.12068462 | 0.908 | 0.715 | 1 | 0 |
| RP5-827C2 | 0.000565 | 0.11732401 | 0.627 | 0.454 | 1 | 0 |
| LTV1 | 0.000569 | 0.15309433 | 0.488 | 0.337 | 1 | 0 |
| GOPC | 0.000584 | 0.10531446 | 0.304 | 0.175 | 1 | 0 |
| SNHG1 | 0.000607 | 0.14428179 | 0.309 | 0.184 | 1 | 0 |
| TSC22D2 | 0.000614 | 0.141369 | 0.35 | 0.212 | 1 | 0 |
| RPP21 | 0.000626 | 0.14915047 | 0.502 | 0.356 | 1 | 0 |
| SAMD4A | 0.000629 | 0.14874529 | 0.507 | 0.344 | 1 | 0 |
| PRDX4 | 0.000636 | 0.10785216 | 0.544 | 0.396 | 1 | 0 |
| BUB3 | 0.000666 | 0.07087186 | 0.498 | 0.337 | 1 | 0 |
| EEF1B2P3 | 0.000672 | 0.09317311 | 0.991 | 0.966 | 1 | 0 |
| RP4-775C1 | 0.0007 | 0.13242007 | 0.415 | 0.264 | 1 | 0 |
| LGALS3 | 0.000703 | 0.18637736 | 0.834 | 0.684 | 1 | 0 |
| SNRPFP1 | 0.000707 | 0.18114755 | 0.682 | 0.561 | 1 | 0 |
| EIF2S2 | 0.000708 | 0.12490156 | 0.931 | 0.868 | 1 | 0 |
| ZNF593 | 0.00072 | 0.08073159 | 0.346 | 0.206 | 1 | 0 |
| FDPSP3 | 0.000731 | 0.11080469 | 0.401 | 0.255 | 1 | 0 |
| DDX21 | 0.000732 | 0.13050389 | 0.972 | 0.929 | 1 | 0 |
| SNTB2 | 0.000739 | 0.14066695 | 0.373 | 0.233 | 1 | 0 |
| RBM26 | 0.000767 | 0.08042036 | 0.525 | 0.353 | 1 | 0 |
| C21orf59 | 0.000787 | 0.14528978 | 0.442 | 0.294 | 1 | 0 |
| NOP58 | 0.000797 | 0.10663379 | 0.447 | 0.294 | 1 | 0 |
| SLC25A4 | 0.0008 | 0.1673649 | 0.447 | 0.31 | 1 | 0 |
| S100A9 | 0.000809 | 0.39749463 | 0.816 | 0.807 | 1 | 0 |
| USMG5 | 0.000811 | 0.13716342 | 0.912 | 0.868 | 1 | 0 |
| DNAJB11 | 0.000822 | 0.13612484 | 0.636 | 0.466 | 1 | 0 |
| GDAP2 | 0.000826 | 0.08582471 | 0.29 | 0.169 | 1 | 0 |
| SQLE | 0.00083 | 0.08232201 | 0.687 | 0.469 | 1 | 0 |
| GADD45A | 0.000831 | 0.25085667 | 0.594 | 0.457 | 1 | 0 |
| STC2 | 0.000864 | 0.16361806 | 0.548 | 0.393 | 1 | 0 |
| PITX1 | 0.000866 | 0.13350481 | 0.484 | 0.337 | 1 | 0 |
| NDUFB2 | 0.000903 | 0.1114661 | 0.945 | 0.844 | 1 | 0 |
| CYP51A1 | 0.000915 | 0.13899287 | 0.737 | 0.604 | 1 | 0 |
| AC006028. | 0.000927 | 0.10287958 | 0.65 | 0.494 | 1 | 0 |
| RPL32 | 0.000935 | 0.08104719 | 1 | 0.997 | 1 | 0 |

| | | | | | | |
|---|---|---|---|---|---|---|
| PRAG1 | 0.00095 | 0.10427659 | 0.276 | 0.153 | 1 | 0 |
| NDUFC1 | 0.000966 | 0.08925005 | 0.922 | 0.767 | 1 | 0 |
| RP11-603J2 | 0.000977 | 0.10829209 | 0.415 | 0.279 | 1 | 0 |
| MT-ATP8 | 0.000999 | 0.16596261 | 0.995 | 0.972 | 1 | 0 |
| CCDC84 | 0.001 | 0.09658721 | 0.355 | 0.218 | 1 | 0 |
| PDCD5P2 | 0.00101 | 0.10197107 | 0.327 | 0.193 | 1 | 0 |
| ZRANB1 | 0.001018 | 0.10442322 | 0.346 | 0.209 | 1 | 0 |
| BTF3P2 | 0.001049 | 0.09286889 | 0.636 | 0.448 | 1 | 0 |
| RHEBP1 | 0.001052 | 0.14013308 | 0.346 | 0.221 | 1 | 0 |
| FKBP15 | 0.001062 | 0.12521254 | 0.521 | 0.368 | 1 | 0 |
| SYPL1 | 0.001066 | 0.12278904 | 0.691 | 0.521 | 1 | 0 |
| MRPS16 | 0.001076 | 0.12916393 | 0.774 | 0.586 | 1 | 0 |
| EIF5 | 0.001099 | 0.10861138 | 0.977 | 0.914 | 1 | 0 |
| RPS23 | 0.001102 | 0.06547683 | 1 | 1 | 1 | 0 |
| C9orf3 | 0.001121 | 0.11947037 | 0.415 | 0.273 | 1 | 0 |
| GNG12 | 0.001123 | 0.14154935 | 0.419 | 0.282 | 1 | 0 |
| RPL35A | 0.001131 | 0.07344098 | 1 | 0.997 | 1 | 0 |
| AHNAK | 0.001138 | 0.08005599 | 0.484 | 0.319 | 1 | 0 |
| PQBP1 | 0.001142 | 0.11399814 | 0.415 | 0.273 | 1 | 0 |
| RRN3 | 0.001146 | 0.15531134 | 0.373 | 0.255 | 1 | 0 |
| RP1-249H1 | 0.001166 | 0.12080542 | 0.866 | 0.776 | 1 | 0 |
| U2AF1L5 | 0.00117 | 0.14205326 | 0.765 | 0.647 | 1 | 0 |
| RP11-283C | 0.001196 | 0.0963552 | 0.392 | 0.242 | 1 | 0 |
| F12 | 0.001196 | 0.11083866 | 0.281 | 0.163 | 1 | 0 |
| HSP90AA2I | 0.001231 | 0.13888041 | 0.982 | 0.84 | 1 | 0 |
| ADGRG1 | 0.001426 | 0.20101479 | 0.613 | 0.485 | 1 | 0 |
| SLC7A1 | 0.001434 | 0.13104868 | 0.558 | 0.402 | 1 | 0 |
| DYNLL1 | 0.001438 | 0.10562275 | 0.949 | 0.929 | 1 | 0 |
| CYBA | 0.001441 | 0.14503532 | 0.521 | 0.362 | 1 | 0 |
| SF3B6 | 0.001451 | 0.12011555 | 0.885 | 0.788 | 1 | 0 |
| PSMA6 | 0.001486 | 0.16179206 | 0.839 | 0.776 | 1 | 0 |
| YWHAZ | 0.001492 | 0.11446493 | 0.991 | 0.963 | 1 | 0 |
| RHEBP2 | 0.001503 | 0.08255014 | 0.295 | 0.172 | 1 | 0 |
| SCD | 0.001507 | 0.14039419 | 0.954 | 0.914 | 1 | 0 |
| RP11-321A | 0.001534 | 0.08253483 | 0.406 | 0.261 | 1 | 0 |
| MTHFD1L | 0.001541 | 0.09977968 | 0.382 | 0.252 | 1 | 0 |
| CCDC6 | 0.001543 | 0.10424853 | 0.687 | 0.537 | 1 | 0 |
| NDUFAF2 | 0.001548 | 0.08128891 | 0.433 | 0.279 | 1 | 0 |
| NDUFB3 | 0.00155 | 0.14295635 | 0.751 | 0.653 | 1 | 0 |
| ITGB6 | 0.001554 | 0.12391519 | 0.286 | 0.169 | 1 | 0 |
| EEF1E1 | 0.00159 | 0.13656969 | 0.498 | 0.353 | 1 | 0 |
| RP11-270C | 0.001631 | 0.14099987 | 0.917 | 0.868 | 1 | 0 |
| TOMM40 | 0.001694 | 0.12878249 | 0.691 | 0.54 | 1 | 0 |
| COX6A1P2 | 0.001714 | 0.12719206 | 0.774 | 0.617 | 1 | 0 |
| UBA52P6 | 0.001738 | 0.06663876 | 0.276 | 0.153 | 1 | 0 |
| SRSF11 | 0.001773 | 0.11218755 | 0.604 | 0.451 | 1 | 0 |
| FBXO45 | 0.001804 | 0.10034595 | 0.525 | 0.365 | 1 | 0 |

| | | | | | | |
|---|---|---|---|---|---|---|
| CBWD3 | 0.00188 | 0.15002409 | 0.862 | 0.755 | 1 | 0 |
| VPS29 | 0.001891 | 0.17024748 | 0.654 | 0.509 | 1 | 0 |
| VANGL1 | 0.001904 | 0.10349477 | 0.493 | 0.356 | 1 | 0 |
| CARHSP1 | 0.001939 | 0.09218864 | 0.429 | 0.285 | 1 | 0 |
| COX6B1 | 0.001947 | 0.13250014 | 0.889 | 0.816 | 1 | 0 |
| LYPD3 | 0.00197 | 0.13663041 | 0.424 | 0.285 | 1 | 0 |
| HACD1 | 0.001997 | 0.11511501 | 0.484 | 0.334 | 1 | 0 |
| DDX10 | 0.00201 | 0.11586996 | 0.35 | 0.227 | 1 | 0 |
| SLC20A2 | 0.002014 | 0.07980648 | 0.286 | 0.169 | 1 | 0 |
| NSD3 | 0.002031 | 0.08077313 | 0.373 | 0.239 | 1 | 0 |
| RP11-51L5. | 0.002045 | 0.07515455 | 0.401 | 0.258 | 1 | 0 |
| XBP1 | 0.002056 | 0.19050291 | 0.774 | 0.666 | 1 | 0 |
| PNN | 0.00206 | 0.10118783 | 0.618 | 0.451 | 1 | 0 |
| PIK3C2A | 0.002107 | 0.10271557 | 0.47 | 0.325 | 1 | 0 |
| RSU1 | 0.002145 | 0.1343047 | 0.848 | 0.758 | 1 | 0 |
| ICA1 | 0.002172 | 0.08685538 | 0.47 | 0.328 | 1 | 0 |
| NPM1P39 | 0.002275 | 0.07097034 | 0.313 | 0.187 | 1 | 0 |
| EIF3K | 0.002355 | 0.1096159 | 0.908 | 0.85 | 1 | 0 |
| PSMA3 | 0.002372 | 0.12226129 | 0.899 | 0.81 | 1 | 0 |
| RP11-686G | 0.002379 | 0.12625059 | 0.691 | 0.528 | 1 | 0 |
| TMSB4X | 0.00239 | 0.11907099 | 1 | 0.994 | 1 | 0 |
| PPP1R13L | 0.002392 | 0.11125372 | 0.346 | 0.218 | 1 | 0 |
| USP32P2 | 0.002441 | 0.07416145 | 0.41 | 0.27 | 1 | 0 |
| TXNP5 | 0.002453 | 0.09579125 | 0.406 | 0.264 | 1 | 0 |
| SYTL2 | 0.002489 | 0.14941536 | 0.346 | 0.227 | 1 | 0 |
| ITSN2 | 0.00254 | 0.11980129 | 0.346 | 0.227 | 1 | 0 |
| ANKRD17 | 0.002542 | 0.15035132 | 0.438 | 0.307 | 1 | 0 |
| EXOSC3 | 0.002548 | 0.07550884 | 0.258 | 0.15 | 1 | 0 |
| RIOX1 | 0.002567 | 0.07726087 | 0.29 | 0.172 | 1 | 0 |
| RP11-378G | 0.002573 | 0.07007461 | 0.304 | 0.187 | 1 | 0 |
| PSMA6P4 | 0.002588 | 0.10678859 | 0.544 | 0.383 | 1 | 0 |
| IFRD1 | 0.002598 | 0.14130092 | 0.424 | 0.301 | 1 | 0 |
| CIPC | 0.002608 | 0.12588802 | 0.475 | 0.325 | 1 | 0 |
| DNAJB1 | 0.002615 | 0.13305702 | 0.631 | 0.479 | 1 | 0 |
| PSMB6 | 0.002655 | 0.11136856 | 0.871 | 0.745 | 1 | 0 |
| UTP11 | 0.002681 | 0.04904758 | 0.544 | 0.38 | 1 | 0 |
| AL021546.6 | 0.002685 | 0.10868034 | 0.304 | 0.193 | 1 | 0 |
| AC007229.1 | 0.002713 | 0.08967445 | 0.318 | 0.199 | 1 | 0 |
| NR2F2 | 0.002715 | 0.1213957 | 0.627 | 0.5 | 1 | 0 |
| RP11-447L1 | 0.002717 | 0.11418548 | 0.65 | 0.5 | 1 | 0 |
| SNRPEP4 | 0.00273 | 0.08062272 | 0.516 | 0.353 | 1 | 0 |
| FEM1B | 0.002735 | 0.0957808 | 0.438 | 0.294 | 1 | 0 |
| TRIB3 | 0.002741 | 0.08572043 | 0.415 | 0.276 | 1 | 0 |
| DHX36 | 0.00275 | 0.09994088 | 0.664 | 0.497 | 1 | 0 |
| SH3GL1 | 0.002764 | 0.11147175 | 0.475 | 0.328 | 1 | 0 |
| MRPS18C | 0.002795 | 0.1238408 | 0.502 | 0.362 | 1 | 0 |
| AFTPH | 0.002827 | 0.09868451 | 0.401 | 0.267 | 1 | 0 |

| | | | | | | |
|---|---|---|---|---|---|---|
| COA6 | 0.002894 | 0.10871449 | 0.429 | 0.294 | 1 | 0 |
| RBM8A | 0.002935 | 0.13301371 | 0.94 | 0.883 | 1 | 0 |
| DEGS1 | 0.002962 | 0.08243423 | 0.327 | 0.206 | 1 | 0 |
| DUSP11 | 0.003023 | 0.12957048 | 0.309 | 0.199 | 1 | 0 |
| OSBPL8 | 0.00305 | 0.11766551 | 0.286 | 0.178 | 1 | 0 |
| TRIM16 | 0.003053 | 0.09137626 | 0.949 | 0.911 | 1 | 0 |
| ZNF26 | 0.00307 | 0.11727625 | 0.387 | 0.27 | 1 | 0 |
| ADAMTSL3 | 0.00308 | 0.07673634 | 0.272 | 0.163 | 1 | 0 |
| MYC | 0.003126 | 0.13601911 | 0.585 | 0.448 | 1 | 0 |
| PPL | 0.003151 | 0.09540645 | 0.369 | 0.248 | 1 | 0 |
| RPL14 | 0.003171 | 0.08695892 | 1 | 1 | 1 | 0 |
| PITPNB | 0.003269 | 0.09913644 | 0.562 | 0.402 | 1 | 0 |
| EBNA1BP2 | 0.003294 | 0.12519968 | 0.604 | 0.482 | 1 | 0 |
| SEC63 | 0.003314 | 0.11890401 | 0.581 | 0.445 | 1 | 0 |
| RPL9 | 0.003338 | 0.07186331 | 0.995 | 0.997 | 1 | 0 |
| RRP15 | 0.003352 | 0.11180353 | 0.493 | 0.359 | 1 | 0 |
| RPS6 | 0.00339 | 0.07853812 | 0.995 | 0.997 | 1 | 0 |
| C5AR2 | 0.003392 | 0.0628847 | 0.323 | 0.202 | 1 | 0 |
| NLRP12 | 0.003425 | 0.08488083 | 0.276 | 0.166 | 1 | 0 |
| PPRC1 | 0.003448 | 0.06847695 | 0.281 | 0.169 | 1 | 0 |
| KLHL42 | 0.003474 | 0.08562647 | 0.327 | 0.202 | 1 | 0 |
| DTNA | 0.003494 | 0.06088128 | 0.378 | 0.245 | 1 | 0 |
| SELENOK | 0.003506 | 0.09998907 | 0.47 | 0.334 | 1 | 0 |
| SMG1P1 | 0.003533 | 0.10784187 | 0.406 | 0.282 | 1 | 0 |
| PHACTR4 | 0.003551 | 0.08219157 | 0.415 | 0.282 | 1 | 0 |
| RANP2 | 0.003587 | 0.07219592 | 0.599 | 0.436 | 1 | 0 |
| SH3BP4 | 0.003591 | 0.1328077 | 0.41 | 0.273 | 1 | 0 |
| CCDC59 | 0.003597 | 0.10737614 | 0.498 | 0.365 | 1 | 0 |
| RPS29P16 | 0.003613 | 0.09530987 | 0.995 | 0.985 | 1 | 0 |
| MRPS14 | 0.003658 | 0.08462442 | 0.571 | 0.417 | 1 | 0 |
| C8orf59 | 0.003729 | 0.09681718 | 0.53 | 0.383 | 1 | 0 |
| TFAP2A | 0.003744 | 0.10368148 | 0.341 | 0.227 | 1 | 0 |
| FAM114A1 | 0.003787 | 0.07405748 | 0.364 | 0.236 | 1 | 0 |
| CREBBP | 0.003815 | 0.09271903 | 0.424 | 0.298 | 1 | 0 |
| ACAT2 | 0.003817 | 0.10668853 | 0.525 | 0.39 | 1 | 0 |
| PROSER2 | 0.003824 | 0.09661234 | 0.3 | 0.19 | 1 | 0 |
| DLC1 | 0.003841 | 0.0917208 | 0.419 | 0.279 | 1 | 0 |
| REXO2 | 0.003844 | 0.0653729 | 0.3 | 0.184 | 1 | 0 |
| PSMA4 | 0.003963 | 0.12742795 | 0.903 | 0.822 | 1 | 0 |
| CTPS1 | 0.003988 | 0.07936714 | 0.401 | 0.264 | 1 | 0 |
| RPS13P2 | 0.004023 | 0.08065313 | 0.982 | 0.994 | 1 | 0 |
| DNAJA2 | 0.004044 | 0.07349798 | 0.419 | 0.276 | 1 | 0 |
| SDE2 | 0.004074 | 0.054641 | 0.267 | 0.16 | 1 | 0 |
| RPS29P3 | 0.004122 | 0.07740214 | 0.521 | 0.365 | 1 | 0 |
| AIMP1 | 0.004153 | 0.15570885 | 0.576 | 0.46 | 1 | 0 |
| RP11-302B | 0.004172 | 0.04878773 | 0.253 | 0.147 | 1 | 0 |
| NPTN | 0.004199 | 0.0942419 | 0.659 | 0.543 | 1 | 0 |

| | | | | | | |
|---|---|---|---|---|---|---|
| RPLP2 | 0.004212 | 0.06999007 | 0.995 | 1 | 1 | 0 |
| HSPE1-MO | 0.004233 | 0.1225521 | 0.848 | 0.742 | 1 | 0 |
| BLVRB | 0.004292 | 0.12288263 | 0.756 | 0.626 | 1 | 0 |
| CYCS | 0.004339 | 0.08151991 | 0.926 | 0.862 | 1 | 0 |
| METTL8 | 0.004349 | 0.12871285 | 0.442 | 0.316 | 1 | 0 |
| CHERP | 0.00435 | 0.06641714 | 0.309 | 0.193 | 1 | 0 |
| ZNF789 | 0.004396 | 0.0910095 | 0.29 | 0.19 | 1 | 0 |
| TCTEX1D2 | 0.004455 | 0.10331565 | 0.742 | 0.607 | 1 | 0 |
| PPME1 | 0.004482 | 0.0866774 | 0.396 | 0.27 | 1 | 0 |
| FRMD6 | 0.004533 | 0.16281798 | 0.488 | 0.374 | 1 | 0 |
| NAA25 | 0.004538 | 0.09374651 | 0.442 | 0.313 | 1 | 0 |
| RBFOX2 | 0.004635 | 0.07319979 | 0.327 | 0.209 | 1 | 0 |
| MALSU1 | 0.004635 | 0.08155294 | 0.3 | 0.193 | 1 | 0 |
| RP11-703G | 0.004691 | 0.10042065 | 0.475 | 0.353 | 1 | 0 |
| RSBN1 | 0.004691 | 0.08041341 | 0.263 | 0.156 | 1 | 0 |
| ASAP1 | 0.004716 | 0.10181621 | 0.562 | 0.411 | 1 | 0 |
| RPL31P49 | 0.004815 | 0.10603568 | 0.986 | 0.972 | 1 | 0 |
| KLF5 | 0.004845 | 0.09244037 | 0.327 | 0.209 | 1 | 0 |
| RP4-604A2 | 0.004871 | 0.0682012 | 0.576 | 0.42 | 1 | 0 |
| TIMM10 | 0.004893 | 0.12392203 | 0.53 | 0.414 | 1 | 0 |
| LTBR | 0.004955 | 0.09014907 | 0.263 | 0.166 | 1 | 0 |
| ERLIN2 | 0.0051 | 0.08644315 | 0.258 | 0.16 | 1 | 0 |
| PDLIM1 | 0.005138 | 0.17717692 | 0.631 | 0.515 | 1 | 0 |
| NPIPB4 | 0.005153 | 0.07974484 | 0.304 | 0.193 | 1 | 0 |
| CTD-2031P | 0.005158 | 0.08744819 | 0.539 | 0.387 | 1 | 0 |
| CAMKK2 | 0.005188 | 0.08670756 | 0.318 | 0.206 | 1 | 0 |
| SUB1P1 | 0.005189 | 0.06706 | 0.645 | 0.482 | 1 | 0 |
| SLC25A32 | 0.005202 | 0.05706399 | 0.276 | 0.166 | 1 | 0 |
| RBX1 | 0.005213 | 0.10258673 | 0.677 | 0.525 | 1 | 0 |
| BIRC6 | 0.005225 | 0.11746418 | 0.484 | 0.356 | 1 | 0 |
| PDCL3 | 0.005327 | 0.08386494 | 0.396 | 0.258 | 1 | 0 |
| SPCS2P4 | 0.005358 | 0.07272569 | 0.3 | 0.187 | 1 | 0 |
| HSP90AA5I | 0.005365 | 0.10077459 | 0.705 | 0.54 | 1 | 0 |
| PFN1 | 0.005389 | 0.07832145 | 1 | 0.994 | 1 | 0 |
| ELOCP18 | 0.005425 | 0.10784252 | 0.719 | 0.577 | 1 | 0 |
| CTC-308K2 | 0.005465 | 0.0990431 | 0.765 | 0.669 | 1 | 0 |
| RALA | 0.005486 | 0.12912542 | 0.65 | 0.537 | 1 | 0 |
| RP11-43D4 | 0.005591 | 0.07411034 | 0.327 | 0.206 | 1 | 0 |
| DNAJA3 | 0.00561 | 0.0835757 | 0.53 | 0.393 | 1 | 0 |
| CTC-260F2 | 0.005619 | 0.1157008 | 0.876 | 0.748 | 1 | 0 |
| RP11-146N | 0.005638 | 0.09047353 | 0.963 | 0.954 | 1 | 0 |
| PPTC7 | 0.005666 | 0.10423796 | 0.304 | 0.199 | 1 | 0 |
| PAK1IP1 | 0.005722 | 0.08757191 | 0.276 | 0.169 | 1 | 0 |
| RPS21 | 0.005737 | 0.07064411 | 0.991 | 0.994 | 1 | 0 |
| SCML1 | 0.005775 | 0.13212956 | 0.465 | 0.35 | 1 | 0 |
| IQGAP1 | 0.005805 | 0.07141603 | 0.604 | 0.457 | 1 | 0 |
| NPIPB5 | 0.005826 | 0.07424996 | 0.327 | 0.215 | 1 | 0 |

| | | | | | | |
|---|---|---|---|---|---|---|
| RNF13 | 0.005858 | 0.09985109 | 0.369 | 0.252 | 1 | 0 |
| PTPN14 | 0.006239 | 0.07525207 | 0.272 | 0.163 | 1 | 0 |
| MRPL19 | 0.00627 | 0.09412342 | 0.479 | 0.362 | 1 | 0 |
| NABP1 | 0.006324 | 0.10738588 | 0.41 | 0.276 | 1 | 0 |
| RBM25 | 0.006387 | 0.12689196 | 0.705 | 0.61 | 1 | 0 |
| SIAH2 | 0.006478 | 0.05386216 | 0.286 | 0.175 | 1 | 0 |
| CTB-147C2 | 0.00648 | 0.09247577 | 0.484 | 0.353 | 1 | 0 |
| CAB39 | 0.006509 | 0.08959235 | 0.336 | 0.23 | 1 | 0 |
| RPS29 | 0.006522 | 0.06736999 | 1 | 1 | 1 | 0 |
| CUL1 | 0.00654 | 0.06837511 | 0.29 | 0.184 | 1 | 0 |
| ERHP1 | 0.00659 | 0.05554449 | 0.355 | 0.23 | 1 | 0 |
| TATDN1P1 | 0.006591 | 0.10223486 | 0.618 | 0.503 | 1 | 0 |
| PDCD5P1 | 0.006613 | 0.06051213 | 0.263 | 0.163 | 1 | 0 |
| TMEM45A | 0.006675 | 0.1003545 | 0.318 | 0.215 | 1 | 0 |
| CDH1 | 0.006722 | 0.13636606 | 0.797 | 0.699 | 1 | 0 |
| EBPL | 0.006776 | 0.07941668 | 0.452 | 0.313 | 1 | 0 |
| SLC38A1 | 0.006807 | 0.13764615 | 0.747 | 0.641 | 1 | 0 |
| GLRX2 | 0.006838 | 0.12959302 | 0.488 | 0.359 | 1 | 0 |
| NDUFA12 | 0.006843 | 0.11269733 | 0.862 | 0.801 | 1 | 0 |
| PPP2R2A | 0.006869 | 0.10179849 | 0.535 | 0.399 | 1 | 0 |
| DNAL1 | 0.006884 | 0.07018266 | 0.433 | 0.298 | 1 | 0 |
| SMARCC1 | 0.006922 | 0.07587224 | 0.415 | 0.282 | 1 | 0 |
| IWS1 | 0.007045 | 0.10170931 | 0.461 | 0.334 | 1 | 0 |
| NDUFAF4 | 0.007057 | 0.08032221 | 0.336 | 0.221 | 1 | 0 |
| TRAM1 | 0.007156 | 0.09124912 | 0.502 | 0.374 | 1 | 0 |
| THBD | 0.007191 | 0.07922459 | 0.263 | 0.163 | 1 | 0 |
| DLG1 | 0.007242 | 0.10623629 | 0.659 | 0.537 | 1 | 0 |
| NDUFS5 | 0.007422 | 0.10226323 | 0.959 | 0.905 | 1 | 0 |
| SEC61B | 0.007465 | 0.06932547 | 0.986 | 0.966 | 1 | 0 |
| AP000275.[illegible] | 0.007479 | 0.09225049 | 0.336 | 0.23 | 1 | 0 |
| RP11-70F1[illegible] | 0.007502 | 0.05720245 | 0.336 | 0.221 | 1 | 0 |
| PBDC1 | 0.007549 | 0.07726984 | 0.479 | 0.35 | 1 | 0 |
| NSRP1 | 0.007672 | 0.04074051 | 0.318 | 0.209 | 1 | 0 |
| RAB3IP | 0.007672 | 0.07217565 | 0.378 | 0.258 | 1 | 0 |
| ASNS | 0.007732 | 0.1306875 | 0.825 | 0.73 | 1 | 0 |
| RP11-744D | 0.007762 | 0.04806311 | 0.364 | 0.236 | 1 | 0 |
| SMTN | 0.007804 | 0.07632309 | 0.253 | 0.156 | 1 | 0 |
| CTD-2303H | 0.008003 | 0.07481665 | 0.396 | 0.276 | 1 | 0 |
| GPR155 | 0.008069 | 0.06981523 | 0.355 | 0.236 | 1 | 0 |
| ESYT2 | 0.008119 | 0.13344899 | 0.581 | 0.445 | 1 | 0 |
| AP1S3 | 0.00815 | 0.07514853 | 0.309 | 0.206 | 1 | 0 |
| INTS2 | 0.008178 | 0.11152795 | 0.272 | 0.175 | 1 | 0 |
| USP31 | 0.008188 | 0.06271035 | 0.392 | 0.273 | 1 | 0 |
| RPS24P13 | 0.008206 | 0.05122396 | 0.258 | 0.153 | 1 | 0 |
| IRX2 | 0.008364 | 0.0847386 | 0.65 | 0.521 | 1 | 0 |
| CCDC43 | 0.008429 | 0.05433587 | 0.35 | 0.233 | 1 | 0 |
| ZFP36L1 | 0.008449 | 0.13881391 | 0.866 | 0.782 | 1 | 0 |

| | | | | | | |
|---|---|---|---|---|---|---|
| INSIG2 | 0.008474 | 0.09610544 | 0.323 | 0.212 | 1 | 0 |
| SLC35E1 | 0.00851 | 0.09990412 | 0.696 | 0.567 | 1 | 0 |
| AC004453.. | 0.00857 | 0.08191613 | 0.972 | 0.979 | 1 | 0 |
| CAPN2 | 0.00861 | 0.1035154 | 0.295 | 0.187 | 1 | 0 |
| TMEM65 | 0.008619 | 0.09726812 | 0.429 | 0.31 | 1 | 0 |
| TSPAN17 | 0.008641 | 0.06347803 | 0.267 | 0.169 | 1 | 0 |
| AIMP2 | 0.008642 | 0.1027813 | 0.65 | 0.5 | 1 | 0 |
| RMDN1 | 0.008644 | 0.05671003 | 0.286 | 0.178 | 1 | 0 |
| RP11-29G8 | 0.008648 | 0.09976344 | 0.645 | 0.521 | 1 | 0 |
| MRPL20 | 0.00868 | 0.07797925 | 0.742 | 0.589 | 1 | 0 |
| RP11-176H | 0.008684 | 0.09996176 | 0.562 | 0.417 | 1 | 0 |
| FAM129B | 0.008765 | 0.07017384 | 0.281 | 0.175 | 1 | 0 |
| AFDN | 0.008767 | 0.07645877 | 0.327 | 0.221 | 1 | 0 |
| RNF115 | 0.008777 | 0.0864006 | 0.53 | 0.411 | 1 | 0 |
| DYNLL1P7 | 0.008801 | 0.06525874 | 0.272 | 0.175 | 1 | 0 |
| EIF5B | 0.008853 | 0.10636724 | 0.931 | 0.911 | 1 | 0 |
| ACTR3 | 0.008943 | 0.14494249 | 0.917 | 0.804 | 1 | 0 |
| RP4-800G7 | 0.009009 | 0.09468595 | 0.797 | 0.675 | 1 | 0 |
| LAMTOR5 | 0.009049 | 0.10438053 | 0.853 | 0.742 | 1 | 0 |
| RAP2B | 0.00905 | 0.15439491 | 0.442 | 0.353 | 1 | 0 |
| COX5A | 0.009055 | 0.07116127 | 0.963 | 0.929 | 1 | 0 |
| DHCR7 | 0.009162 | 0.09917483 | 0.857 | 0.709 | 1 | 0 |
| NDUFB10 | 0.009166 | 0.10999707 | 0.783 | 0.684 | 1 | 0 |
| TRIM26 | 0.009184 | 0.10317822 | 0.318 | 0.212 | 1 | 0 |
| RP11-467L1 | 0.009279 | 0.10429667 | 0.622 | 0.491 | 1 | 0 |
| CBWD7 | 0.00938 | 0.12135178 | 0.788 | 0.684 | 1 | 0 |
| CBFB | 0.009539 | 0.11607491 | 0.263 | 0.172 | 1 | 0 |
| GEMIN7 | 0.00957 | 0.06779978 | 0.35 | 0.233 | 1 | 0 |
| DMKN | 0.009587 | 0.09542531 | 0.613 | 0.482 | 1 | 0 |
| AK2 | 0.009619 | 0.08956978 | 0.793 | 0.69 | 1 | 0 |
| RPL30 | 0.009655 | 0.0606592 | 1 | 1 | 1 | 0 |
| MRPS28 | 0.009686 | 0.0525637 | 0.29 | 0.184 | 1 | 0 |
| RYBP | 0.009705 | 0.1020555 | 0.396 | 0.282 | 1 | 0 |
| LETM1 | 0.009729 | 0.1152426 | 0.41 | 0.294 | 1 | 0 |
| TYW3 | 0.009803 | 0.07656897 | 0.438 | 0.331 | 1 | 0 |
| USP32P1 | 0.009819 | 0.08354423 | 0.424 | 0.301 | 1 | 0 |
| KCNK15 | 0.009869 | 0.04144312 | 0.276 | 0.175 | 1 | 0 |
| STX3 | 0.00988 | 0.07830995 | 0.336 | 0.218 | 1 | 0 |
| RPL30P14 | 0.009973 | 0.06504098 | 1 | 0.997 | 1 | 0 |
| GABBR1 | 1.17E-44 | 1.04920853 | 0.867 | 0.333 | 2.42E-40 | 1 |
| UBD | 1.62E-43 | 1.0330906 | 0.855 | 0.32 | 3.35E-39 | 1 |
| IL32 | 7.95E-38 | 0.94454135 | 0.976 | 0.582 | 1.64E-33 | 1 |
| B2M | 1.73E-33 | 0.57254247 | 1 | 0.976 | 3.58E-29 | 1 |
| SERPINA3 | 8.46E-28 | 0.79291264 | 0.661 | 0.214 | 1.75E-23 | 1 |
| RP11-986E | 9.97E-27 | 0.75846794 | 0.661 | 0.209 | 2.06E-22 | 1 |
| HLA-A | 4.68E-25 | 0.6426719 | 0.952 | 0.841 | 9.65E-21 | 1 |
| RPS27L | 2.37E-21 | 0.65678394 | 0.903 | 0.701 | 4.88E-17 | 1 |

| | | | | | | |
|---|---|---|---|---|---|---|
| EBI3 | 2.65E-21 | 0.5647252 | 0.552 | 0.177 | 5.47E-17 | 1 |
| PARD6B | 5.70E-21 | 0.56993913 | 0.958 | 0.778 | 1.18E-16 | 1 |
| TIMP1 | 2.55E-19 | 0.74187116 | 0.879 | 0.733 | 5.27E-15 | 1 |
| CD63 | 8.29E-19 | 0.46581999 | 0.952 | 0.754 | 1.71E-14 | 1 |
| HLA-C | 8.88E-18 | 0.47936064 | 0.885 | 0.622 | 1.83E-13 | 1 |
| ZMAT3 | 3.47E-17 | 0.50666019 | 0.788 | 0.484 | 7.16E-13 | 1 |
| UBB | 4.51E-17 | 0.34017497 | 0.988 | 0.963 | 9.30E-13 | 1 |
| GRN | 8.93E-17 | 0.42968862 | 0.933 | 0.786 | 1.84E-12 | 1 |
| CCL2 | 9.66E-17 | 0.55870579 | 0.933 | 0.704 | 1.99E-12 | 1 |
| HLA-B | 1.05E-16 | 0.51204421 | 0.842 | 0.577 | 2.17E-12 | 1 |
| RFTN1 | 1.60E-16 | 0.3984452 | 0.418 | 0.116 | 3.29E-12 | 1 |
| CD59 | 4.55E-16 | 0.44263476 | 0.909 | 0.688 | 9.39E-12 | 1 |
| PSAP | 3.38E-15 | 0.38561653 | 0.964 | 0.849 | 6.98E-11 | 1 |
| FST | 3.88E-15 | 0.29446684 | 0.37 | 0.09 | 8.01E-11 | 1 |
| H3F3AP6 | 1.28E-14 | 0.39019011 | 0.933 | 0.741 | 2.64E-10 | 1 |
| GTF2IP1 | 4.23E-14 | 0.3415619 | 0.964 | 0.892 | 8.73E-10 | 1 |
| GTF2I | 6.30E-14 | 0.31653982 | 0.994 | 0.971 | 1.30E-09 | 1 |
| SOX4 | 1.16E-13 | 0.41151473 | 0.933 | 0.796 | 2.39E-09 | 1 |
| CYP1A1 | 1.22E-13 | 0.45397274 | 0.976 | 0.825 | 2.52E-09 | 1 |
| ATP1B1 | 3.74E-13 | 0.43198232 | 0.964 | 0.815 | 7.71E-09 | 1 |
| CDKN1A | 5.49E-13 | 0.44195286 | 0.915 | 0.68 | 1.13E-08 | 1 |
| BBOX1 | 2.66E-12 | 0.23600785 | 0.255 | 0.05 | 5.50E-08 | 1 |
| H3F3AP4 | 2.93E-12 | 0.37372206 | 0.909 | 0.738 | 6.04E-08 | 1 |
| UBBP4 | 4.36E-12 | 0.32603891 | 0.921 | 0.807 | 9.01E-08 | 1 |
| OST4 | 4.81E-12 | 0.31180752 | 0.976 | 0.947 | 9.93E-08 | 1 |
| CTC-260F2( | 5.65E-12 | 0.3495367 | 0.933 | 0.741 | 1.17E-07 | 1 |
| PHLDA1 | 5.84E-12 | 0.38708919 | 0.594 | 0.315 | 1.21E-07 | 1 |
| TMBIM6 | 1.35E-11 | 0.33640983 | 0.97 | 0.905 | 2.79E-07 | 1 |
| TMSB10 | 2.15E-11 | 0.28314486 | 1 | 0.984 | 4.44E-07 | 1 |
| EGR1 | 3.14E-11 | 0.42128508 | 0.661 | 0.36 | 6.48E-07 | 1 |
| TAPBP | 3.34E-11 | 0.37012111 | 0.939 | 0.81 | 6.89E-07 | 1 |
| ODC1 | 3.94E-11 | 0.42875426 | 0.776 | 0.603 | 8.14E-07 | 1 |
| HIST2H2AA | 4.26E-11 | 0.3721298 | 0.661 | 0.389 | 8.80E-07 | 1 |
| MT-ND6 | 5.32E-11 | 0.27157908 | 1 | 0.96 | 1.10E-06 | 1 |
| RNASEK | 9.43E-11 | 0.28355796 | 0.97 | 0.884 | 1.95E-06 | 1 |
| H3F3A | 1.42E-10 | 0.21071145 | 1 | 0.995 | 2.92E-06 | 1 |
| GDF15 | 1.68E-10 | 0.42808357 | 0.57 | 0.294 | 3.47E-06 | 1 |
| NFKBIA | 1.77E-10 | 0.33824547 | 0.933 | 0.767 | 3.65E-06 | 1 |
| HIST2H2AA | 1.81E-10 | 0.3613722 | 0.648 | 0.386 | 3.73E-06 | 1 |
| HES1 | 2.02E-10 | 0.37249451 | 0.8 | 0.516 | 4.18E-06 | 1 |
| PSME1 | 2.18E-10 | 0.33568893 | 0.855 | 0.675 | 4.51E-06 | 1 |
| CFB | 2.48E-10 | 0.34128764 | 0.43 | 0.188 | 5.12E-06 | 1 |
| NDUFA13 | 3.25E-10 | 0.31670839 | 0.933 | 0.73 | 6.71E-06 | 1 |
| HIST1H1C | 6.59E-10 | 0.26934253 | 0.436 | 0.19 | 1.36E-05 | 1 |
| ANXA2P2 | 7.10E-10 | 0.28149549 | 0.945 | 0.799 | 1.47E-05 | 1 |
| SEZ6L2 | 8.36E-10 | 0.2908711 | 0.739 | 0.484 | 1.72E-05 | 1 |
| CCNG2 | 1.03E-09 | 0.31890481 | 0.503 | 0.259 | 2.12E-05 | 1 |

| | | | | | | |
|---|---|---|---|---|---|---|
| SULF2 | 1.49E-09 | 0.29271491 | 0.945 | 0.794 | 3.07E-05 | 1 |
| COX6C | 1.55E-09 | 0.2865652 | 0.994 | 0.947 | 3.20E-05 | 1 |
| RNASEK-C1 | 1.74E-09 | 0.23639101 | 1 | 0.952 | 3.59E-05 | 1 |
| GTF2IP4 | 1.79E-09 | 0.26395853 | 0.97 | 0.91 | 3.70E-05 | 1 |
| BTG1 | 4.39E-09 | 0.29607122 | 0.576 | 0.333 | 9.06E-05 | 1 |
| PSMD6 | 4.80E-09 | 0.2376947 | 0.976 | 0.984 | 9.90E-05 | 1 |
| RP1-180M1 | 5.95E-09 | 0.30476298 | 0.945 | 0.751 | 0.000123 | 1 |
| COX7C | 7.16E-09 | 0.25056634 | 1 | 0.968 | 0.000148 | 1 |
| PTRH2 | 9.18E-09 | 0.31874827 | 0.715 | 0.5 | 0.000189 | 1 |
| LTB | 9.44E-09 | 0.33715305 | 0.988 | 0.894 | 0.000195 | 1 |
| NKX3-1 | 1.15E-08 | 0.21077261 | 0.358 | 0.132 | 0.000237 | 1 |
| FSCN1 | 1.28E-08 | 0.2419388 | 0.4 | 0.177 | 0.000263 | 1 |
| MYLIP | 1.32E-08 | 0.19469366 | 0.309 | 0.108 | 0.000273 | 1 |
| HIPK2 | 1.33E-08 | 0.28619107 | 0.564 | 0.323 | 0.000275 | 1 |
| ABAT | 1.52E-08 | 0.29165826 | 0.745 | 0.495 | 0.000314 | 1 |
| CXXC5 | 2.28E-08 | 0.28504662 | 0.412 | 0.193 | 0.00047 | 1 |
| UBBP1 | 2.36E-08 | 0.24456124 | 0.655 | 0.431 | 0.000486 | 1 |
| BPIFA4P | 2.93E-08 | 0.24601327 | 0.285 | 0.106 | 0.000605 | 1 |
| FRMD3 | 3.07E-08 | 0.20158182 | 0.309 | 0.119 | 0.000633 | 1 |
| RP11-349N | 3.30E-08 | 0.27902427 | 0.697 | 0.46 | 0.00068 | 1 |
| ORMDL3 | 3.38E-08 | 0.25413876 | 0.461 | 0.23 | 0.000697 | 1 |
| C3 | 3.70E-08 | 0.23358239 | 0.521 | 0.283 | 0.000764 | 1 |
| PPIAP11 | 4.71E-08 | 0.2555169 | 0.988 | 0.929 | 0.000972 | 1 |
| LPCAT1 | 5.46E-08 | 0.24540117 | 0.564 | 0.325 | 0.001127 | 1 |
| ST3GAL1 | 6.09E-08 | 0.29032022 | 0.752 | 0.532 | 0.001258 | 1 |
| CIR1 | 6.64E-08 | 0.21451966 | 0.552 | 0.312 | 0.001371 | 1 |
| CD58 | 7.53E-08 | 0.20600879 | 0.394 | 0.177 | 0.001555 | 1 |
| ATP5J2-PT( | 8.36E-08 | 0.2331919 | 0.891 | 0.69 | 0.001725 | 1 |
| PPIAP22 | 8.41E-08 | 0.21103805 | 0.994 | 0.979 | 0.001736 | 1 |
| NECAB1 | 8.43E-08 | 0.34164912 | 0.582 | 0.381 | 0.00174 | 1 |
| VAMP8 | 8.46E-08 | 0.30215518 | 0.939 | 0.836 | 0.001745 | 1 |
| PSME2 | 8.46E-08 | 0.32026678 | 0.8 | 0.653 | 0.001745 | 1 |
| PXDN | 8.70E-08 | 0.29099927 | 0.63 | 0.407 | 0.001795 | 1 |
| HLA-F | 8.70E-08 | 0.19566493 | 0.315 | 0.13 | 0.001796 | 1 |
| BIRC3 | 1.49E-07 | 0.29619908 | 0.648 | 0.434 | 0.003085 | 1 |
| NDUFB4 | 1.50E-07 | 0.26507178 | 0.939 | 0.799 | 0.003102 | 1 |
| SETD1B | 1.82E-07 | 0.24308312 | 0.527 | 0.294 | 0.003749 | 1 |
| RAC1P2 | 2.05E-07 | 0.24777931 | 0.964 | 0.847 | 0.004235 | 1 |
| C8orf88 | 2.71E-07 | 0.18205341 | 0.261 | 0.095 | 0.005599 | 1 |
| TMEM59 | 2.73E-07 | 0.29845124 | 0.758 | 0.593 | 0.005634 | 1 |
| PPIAP31 | 2.85E-07 | 0.20777845 | 0.976 | 0.95 | 0.005882 | 1 |
| UBC | 3.22E-07 | 0.28006138 | 0.933 | 0.87 | 0.006642 | 1 |
| HSP90AA2I | 3.59E-07 | 0.23263429 | 0.958 | 0.87 | 0.007405 | 1 |
| ZFP36 | 3.70E-07 | 0.26095265 | 0.455 | 0.246 | 0.007633 | 1 |
| RP11-665C | 3.87E-07 | 0.21582475 | 0.673 | 0.429 | 0.00798 | 1 |
| HSPA1A | 4.44E-07 | 0.26347524 | 0.818 | 0.635 | 0.009159 | 1 |
| SYF2 | 4.45E-07 | 0.23074281 | 0.442 | 0.243 | 0.009187 | 1 |

| | | | | | | |
|---|---|---|---|---|---|---|
| COX7CP1 | 4.55E-07 | 0.23184406 | 0.891 | 0.759 | 0.009398 | 1 |
| ENY2 | 4.61E-07 | 0.24014533 | 0.915 | 0.804 | 0.00951 | 1 |
| MAGED1 | 4.75E-07 | 0.26607627 | 0.885 | 0.828 | 0.009806 | 1 |
| TAP1 | 5.16E-07 | 0.19999385 | 0.388 | 0.193 | 0.010649 | 1 |
| CTD-3214H | 5.70E-07 | 0.17666462 | 0.491 | 0.259 | 0.011768 | 1 |
| SREBF1 | 6.00E-07 | 0.26188074 | 0.703 | 0.495 | 0.012392 | 1 |
| SRSF5 | 6.17E-07 | 0.26787394 | 0.776 | 0.624 | 0.012739 | 1 |
| PGPEP1 | 7.00E-07 | 0.22507593 | 0.539 | 0.317 | 0.014452 | 1 |
| RPL36AL | 7.11E-07 | 0.18387581 | 0.982 | 0.979 | 0.01467 | 1 |
| FXR1 | 7.62E-07 | 0.26218609 | 0.655 | 0.442 | 0.015724 | 1 |
| CDIPT | 8.34E-07 | 0.30979904 | 0.697 | 0.526 | 0.017206 | 1 |
| TRIOBP | 8.41E-07 | 0.19860558 | 0.491 | 0.283 | 0.017363 | 1 |
| TMEM205 | 9.36E-07 | 0.27277019 | 0.873 | 0.741 | 0.019326 | 1 |
| GPRC5B | 1.03E-06 | 0.13088112 | 0.255 | 0.095 | 0.021184 | 1 |
| RPS27P27 | 1.13E-06 | 0.16286284 | 0.297 | 0.127 | 0.023301 | 1 |
| TSKU | 1.14E-06 | 0.31053447 | 0.582 | 0.392 | 0.023462 | 1 |
| NDUFB4P1 | 1.26E-06 | 0.18067054 | 0.533 | 0.315 | 0.025918 | 1 |
| AC093850. | 1.29E-06 | 0.22343444 | 0.655 | 0.458 | 0.026691 | 1 |
| SLC12A2 | 1.48E-06 | 0.17363632 | 0.412 | 0.206 | 0.030471 | 1 |
| H3F3BP1 | 1.58E-06 | 0.18656011 | 0.606 | 0.41 | 0.032565 | 1 |
| FIS1 | 1.71E-06 | 0.21280144 | 0.952 | 0.915 | 0.035381 | 1 |
| SOX9 | 1.84E-06 | 0.26052796 | 0.485 | 0.267 | 0.038073 | 1 |
| NDUFB11 | 2.00E-06 | 0.24854709 | 0.885 | 0.778 | 0.041346 | 1 |
| PNRC1 | 2.15E-06 | 0.23870895 | 0.709 | 0.484 | 0.044418 | 1 |
| NDUFB1 | 2.21E-06 | 0.20354827 | 0.848 | 0.696 | 0.04553 | 1 |
| NDUFA3 | 2.37E-06 | 0.23994535 | 0.836 | 0.693 | 0.048873 | 1 |
| CDK6 | 2.45E-06 | 0.26387107 | 0.576 | 0.376 | 0.050598 | 1 |
| XXbac-BPG | 2.78E-06 | 0.20825968 | 0.339 | 0.164 | 0.057351 | 1 |
| TP53TG1 | 2.80E-06 | 0.30052534 | 0.497 | 0.315 | 0.057797 | 1 |
| CSTB | 2.80E-06 | 0.16576981 | 0.994 | 0.987 | 0.057834 | 1 |
| RP11-391L3 | 2.95E-06 | 0.20707495 | 0.988 | 0.939 | 0.060867 | 1 |
| ABCA5 | 2.98E-06 | 0.19823588 | 0.321 | 0.153 | 0.061486 | 1 |
| PSEN1 | 3.04E-06 | 0.21839354 | 0.509 | 0.31 | 0.062841 | 1 |
| AKAP9 | 3.20E-06 | 0.20309966 | 0.679 | 0.487 | 0.066144 | 1 |
| PMS2P1 | 3.28E-06 | 0.1862628 | 0.455 | 0.262 | 0.067662 | 1 |
| LAMTOR4 | 3.36E-06 | 0.27180539 | 0.745 | 0.593 | 0.069404 | 1 |
| LGALS1 | 3.80E-06 | 0.27847954 | 0.436 | 0.259 | 0.078399 | 1 |
| CHD6 | 4.06E-06 | 0.17369236 | 0.467 | 0.259 | 0.083858 | 1 |
| ZMYND8 | 4.23E-06 | 0.27241184 | 0.752 | 0.603 | 0.08738 | 1 |
| MTND6P4 | 4.29E-06 | 0.20503213 | 0.836 | 0.693 | 0.088447 | 1 |
| TRIM37 | 4.61E-06 | 0.2000925 | 0.933 | 0.892 | 0.09512 | 1 |
| CCDC159 | 4.87E-06 | 0.25068782 | 0.697 | 0.471 | 0.100468 | 1 |
| AGRN | 5.18E-06 | 0.2885466 | 0.636 | 0.418 | 0.106907 | 1 |
| SPATS2L | 5.28E-06 | 0.2248536 | 0.848 | 0.759 | 0.108984 | 1 |
| HSP90AA1 | 5.64E-06 | 0.12737176 | 1 | 1 | 0.116433 | 1 |
| JAK1 | 6.13E-06 | 0.18163118 | 0.491 | 0.283 | 0.126435 | 1 |
| NDUFA1 | 6.65E-06 | 0.2115006 | 0.939 | 0.854 | 0.137239 | 1 |

| | | | | | | |
|---|---|---|---|---|---|---|
| FAM210B | 6.80E-06 | 0.29065153 | 0.842 | 0.767 | 0.140311 | 1 |
| YPEL5 | 6.98E-06 | 0.24177992 | 0.612 | 0.423 | 0.144033 | 1 |
| MBNL2 | 7.25E-06 | 0.21397518 | 0.358 | 0.188 | 0.14969 | 1 |
| AC002398.! | 7.39E-06 | 0.21094922 | 0.467 | 0.278 | 0.15243 | 1 |
| H3F3B | 7.66E-06 | 0.1601806 | 1 | 0.987 | 0.158016 | 1 |
| OCIAD2 | 7.84E-06 | 0.2366479 | 0.788 | 0.646 | 0.161728 | 1 |
| HIST1H4H | 7.99E-06 | 0.1995073 | 0.382 | 0.201 | 0.164868 | 1 |
| RBM38 | 8.06E-06 | 0.21262899 | 0.727 | 0.542 | 0.166406 | 1 |
| FTL | 8.09E-06 | 0.18430166 | 0.952 | 0.926 | 0.167008 | 1 |
| MB21D2 | 8.61E-06 | 0.25119186 | 0.594 | 0.429 | 0.177667 | 1 |
| HIST1H2BK | 8.84E-06 | 0.19268974 | 0.539 | 0.347 | 0.182399 | 1 |
| LGALS3BP | 9.44E-06 | 0.24470726 | 0.315 | 0.159 | 0.194788 | 1 |
| SUB1 | 1.01E-05 | 0.17107899 | 0.988 | 0.96 | 0.20802 | 1 |
| NAA38 | 1.12E-05 | 0.20504101 | 0.727 | 0.55 | 0.230666 | 1 |
| UGCG | 1.12E-05 | 0.27331067 | 0.576 | 0.378 | 0.230676 | 1 |
| MDK | 1.28E-05 | 0.35984049 | 0.721 | 0.608 | 0.263785 | 1 |
| PLXDC2 | 1.57E-05 | 0.21978157 | 0.424 | 0.259 | 0.32327 | 1 |
| RP11-691N | 1.58E-05 | 0.13966071 | 0.424 | 0.243 | 0.325791 | 1 |
| ATP6V0E1 | 1.67E-05 | 0.20118244 | 0.945 | 0.926 | 0.344373 | 1 |
| IRF1 | 1.77E-05 | 0.23954966 | 0.321 | 0.167 | 0.364428 | 1 |
| POLR2K | 1.95E-05 | 0.21131421 | 0.855 | 0.683 | 0.401524 | 1 |
| PPIAP29 | 1.95E-05 | 0.1821497 | 0.939 | 0.857 | 0.401956 | 1 |
| PTTG1IP | 1.97E-05 | 0.2219643 | 0.873 | 0.738 | 0.405771 | 1 |
| RRM2B | 1.97E-05 | 0.21053578 | 0.515 | 0.32 | 0.406274 | 1 |
| RP11-402J6 | 2.01E-05 | 0.15809046 | 0.576 | 0.394 | 0.415102 | 1 |
| SSR4 | 2.01E-05 | 0.25183724 | 0.824 | 0.698 | 0.415139 | 1 |
| TMEM132A | 2.07E-05 | 0.1902406 | 0.806 | 0.638 | 0.427521 | 1 |
| IFNGR1 | 2.18E-05 | 0.19745101 | 0.655 | 0.455 | 0.449538 | 1 |
| OPTN | 2.22E-05 | 0.17379779 | 0.624 | 0.45 | 0.457996 | 1 |
| HLA-J | 2.24E-05 | 0.14693381 | 0.358 | 0.185 | 0.462147 | 1 |
| SDC4 | 2.25E-05 | 0.18048346 | 0.8 | 0.659 | 0.464131 | 1 |
| ISCU | 2.33E-05 | 0.26842602 | 0.782 | 0.661 | 0.481128 | 1 |
| PSMB8-AS1 | 2.37E-05 | 0.14094323 | 0.382 | 0.209 | 0.489593 | 1 |
| ITGAV | 2.52E-05 | 0.16904153 | 0.939 | 0.802 | 0.520185 | 1 |
| CTD-2545G | 2.61E-05 | 0.20346011 | 0.933 | 0.899 | 0.538999 | 1 |
| ARL6IP5 | 2.66E-05 | 0.24337842 | 0.764 | 0.577 | 0.549104 | 1 |
| HEXIM1 | 2.70E-05 | 0.13694658 | 0.545 | 0.344 | 0.556959 | 1 |
| PODXL | 2.72E-05 | 0.21304074 | 0.436 | 0.27 | 0.560807 | 1 |
| RAD51C | 3.05E-05 | 0.26118964 | 0.8 | 0.698 | 0.629405 | 1 |
| MAP1LC3B | 3.21E-05 | 0.23562936 | 0.867 | 0.728 | 0.661566 | 1 |
| TRIM56 | 3.59E-05 | 0.17686199 | 0.824 | 0.646 | 0.739972 | 1 |
| IFITM1 | 3.83E-05 | 0.18956475 | 0.842 | 0.717 | 0.790521 | 1 |
| NDUFS5 | 4.05E-05 | 0.1882719 | 0.964 | 0.91 | 0.835917 | 1 |
| WIPF2 | 4.11E-05 | 0.20626949 | 0.776 | 0.582 | 0.847361 | 1 |
| DPYSL2 | 4.12E-05 | 0.19559217 | 0.406 | 0.238 | 0.850448 | 1 |
| CST3 | 4.15E-05 | 0.17071716 | 0.8 | 0.632 | 0.856516 | 1 |
| IFI30 | 4.41E-05 | 0.20544358 | 0.636 | 0.481 | 0.909883 | 1 |

| | | | | | | |
|---|---|---|---|---|---|---|
| LAPTM4A | 4.48E-05 | 0.21313905 | 0.891 | 0.82 | 0.925634 | 1 |
| MYL12B | 4.61E-05 | 0.1328948 | 1 | 0.995 | 0.95117 | 1 |
| ATP5E | 4.62E-05 | 0.13602216 | 1 | 1 | 0.953604 | 1 |
| ZKSCAN1 | 4.89E-05 | 0.19794746 | 0.758 | 0.611 | 1 | 1 |
| COX4I1 | 5.54E-05 | 0.15618713 | 1 | 0.937 | 1 | 1 |
| IGSF3 | 5.72E-05 | 0.17060962 | 0.691 | 0.487 | 1 | 1 |
| RHOV | 6.16E-05 | 0.10928962 | 0.339 | 0.177 | 1 | 1 |
| COX7A2 | 6.53E-05 | 0.17925466 | 0.952 | 0.892 | 1 | 1 |
| CUTA | 6.77E-05 | 0.20420373 | 0.77 | 0.659 | 1 | 1 |
| AC007192.4 | 6.82E-05 | 0.20059501 | 0.782 | 0.696 | 1 | 1 |
| ITM2B | 7.27E-05 | 0.21673824 | 0.758 | 0.601 | 1 | 1 |
| CYB5A | 7.37E-05 | 0.18717933 | 0.855 | 0.759 | 1 | 1 |
| TMBIM4 | 7.39E-05 | 0.22259672 | 0.642 | 0.471 | 1 | 1 |
| VPS39 | 7.49E-05 | 0.08176923 | 0.267 | 0.124 | 1 | 1 |
| FAM83A | 7.53E-05 | 0.14756405 | 0.388 | 0.217 | 1 | 1 |
| UBL5 | 7.77E-05 | 0.20133437 | 0.8 | 0.669 | 1 | 1 |
| IRGQ | 7.85E-05 | 0.15834293 | 0.503 | 0.333 | 1 | 1 |
| SOD1 | 8.01E-05 | 0.17655337 | 0.952 | 0.905 | 1 | 1 |
| PPIG | 8.14E-05 | 0.17943815 | 0.733 | 0.556 | 1 | 1 |
| NDUFA4 | 8.43E-05 | 0.17471787 | 0.982 | 0.95 | 1 | 1 |
| RPS19 | 8.51E-05 | 0.10661287 | 1 | 1 | 1 | 1 |
| HELZ2 | 8.55E-05 | 0.19065155 | 0.279 | 0.143 | 1 | 1 |
| GNAI2 | 8.82E-05 | 0.16995712 | 0.764 | 0.619 | 1 | 1 |
| NEO1 | 8.87E-05 | 0.19130582 | 0.412 | 0.257 | 1 | 1 |
| TNIP1 | 9.12E-05 | 0.18598543 | 0.552 | 0.373 | 1 | 1 |
| RP11-251G | 9.37E-05 | 0.21230147 | 0.685 | 0.508 | 1 | 1 |
| DDB2 | 9.48E-05 | 0.13290221 | 0.352 | 0.193 | 1 | 1 |
| RP11-745O | 9.50E-05 | 0.20044626 | 0.648 | 0.495 | 1 | 1 |
| IFNAR2 | 9.56E-05 | 0.15334915 | 0.43 | 0.272 | 1 | 1 |
| RNF181 | 9.64E-05 | 0.19969333 | 0.727 | 0.558 | 1 | 1 |
| HSP90AA5I | 9.70E-05 | 0.15186414 | 0.733 | 0.55 | 1 | 1 |
| NME7 | 9.77E-05 | 0.19299697 | 0.558 | 0.397 | 1 | 1 |
| RAC1 | 9.88E-05 | 0.13339072 | 1 | 0.992 | 1 | 1 |
| E2F3 | 0.000102 | 0.12636505 | 0.358 | 0.204 | 1 | 1 |
| ORMDL2 | 0.000103 | 0.15801124 | 0.388 | 0.228 | 1 | 1 |
| RP11-395L1 | 0.000108 | 0.12022985 | 0.273 | 0.135 | 1 | 1 |
| PET100 | 0.000112 | 0.16476523 | 0.442 | 0.275 | 1 | 1 |
| YEATS2 | 0.000113 | 0.12073006 | 0.279 | 0.143 | 1 | 1 |
| TFDP2 | 0.000117 | 0.15881021 | 0.4 | 0.246 | 1 | 1 |
| PSENEN | 0.000117 | 0.18720089 | 0.497 | 0.336 | 1 | 1 |
| PRAF2 | 0.000119 | 0.18350818 | 0.424 | 0.267 | 1 | 1 |
| NDUFA12 | 0.00012 | 0.20798304 | 0.909 | 0.788 | 1 | 1 |
| SLU7 | 0.000121 | 0.12590011 | 0.37 | 0.214 | 1 | 1 |
| SRP14 | 0.000125 | 0.14636926 | 0.976 | 0.947 | 1 | 1 |
| UVRAG | 0.000126 | 0.13234129 | 0.345 | 0.193 | 1 | 1 |
| CYBA | 0.000126 | 0.18618714 | 0.539 | 0.376 | 1 | 1 |
| REPIN1 | 0.000126 | 0.17727067 | 0.679 | 0.503 | 1 | 1 |

| | | | | | | |
|---|---|---|---|---|---|---|
| RBM8A | 0.000132 | 0.15635096 | 0.952 | 0.886 | 1 | 1 |
| NCOA3 | 0.000133 | 0.1889588 | 0.915 | 0.884 | 1 | 1 |
| PCYOX1 | 0.000136 | 0.16823694 | 0.648 | 0.474 | 1 | 1 |
| UROD | 0.000137 | 0.13442063 | 0.406 | 0.241 | 1 | 1 |
| GABARAP | 0.000138 | 0.20726898 | 0.945 | 0.897 | 1 | 1 |
| FOXA1 | 0.000143 | 0.1878221 | 0.539 | 0.373 | 1 | 1 |
| NEAT1 | 0.000148 | 0.21656025 | 0.655 | 0.505 | 1 | 1 |
| ATP5J2 | 0.000151 | 0.17032544 | 0.994 | 0.913 | 1 | 1 |
| C19orf33 | 0.000153 | 0.21195733 | 0.642 | 0.5 | 1 | 1 |
| DBI | 0.000153 | 0.16397328 | 0.976 | 0.934 | 1 | 1 |
| EDF1 | 0.000153 | 0.12992001 | 0.933 | 0.878 | 1 | 1 |
| APLP2 | 0.000156 | 0.2172358 | 0.885 | 0.841 | 1 | 1 |
| C6orf141 | 0.00016 | 0.13794512 | 0.273 | 0.14 | 1 | 1 |
| ELOB | 0.00016 | 0.16124537 | 0.921 | 0.833 | 1 | 1 |
| SYNE4 | 0.000161 | 0.16089625 | 0.442 | 0.288 | 1 | 1 |
| CISD1 | 0.000161 | 0.22153535 | 0.57 | 0.41 | 1 | 1 |
| NINJ1 | 0.000165 | 0.20514452 | 0.642 | 0.471 | 1 | 1 |
| UBXN4 | 0.000166 | 0.21214667 | 0.745 | 0.616 | 1 | 1 |
| NPC2 | 0.000168 | 0.29250187 | 0.685 | 0.574 | 1 | 1 |
| COX7B | 0.000169 | 0.16681071 | 0.958 | 0.823 | 1 | 1 |
| PSMB4 | 0.000172 | 0.16542746 | 0.97 | 0.91 | 1 | 1 |
| HBP1 | 0.000178 | 0.14715714 | 0.279 | 0.151 | 1 | 1 |
| CHST15 | 0.00018 | 0.16106265 | 0.485 | 0.32 | 1 | 1 |
| NDUFC2 | 0.000184 | 0.18761829 | 0.915 | 0.775 | 1 | 1 |
| PRDX1P1 | 0.000185 | 0.14936402 | 0.867 | 0.685 | 1 | 1 |
| DAAM1 | 0.000188 | 0.20276386 | 0.855 | 0.743 | 1 | 1 |
| COG5 | 0.000192 | 0.12727731 | 0.358 | 0.209 | 1 | 1 |
| MOV10 | 0.000197 | 0.11698158 | 0.303 | 0.161 | 1 | 1 |
| CAMK2N1 | 0.000199 | 0.10736311 | 0.358 | 0.201 | 1 | 1 |
| SPINT2 | 0.0002 | 0.16386228 | 0.976 | 0.939 | 1 | 1 |
| SCARB2 | 0.000204 | 0.18780149 | 0.6 | 0.429 | 1 | 1 |
| MINOS1-NI | 0.000204 | 0.17173932 | 0.794 | 0.653 | 1 | 1 |
| EGFR | 0.00021 | 0.19528532 | 0.315 | 0.175 | 1 | 1 |
| PLXNB2 | 0.000213 | 0.1742885 | 0.515 | 0.36 | 1 | 1 |
| HIST2H2AC | 0.000216 | 0.15450733 | 0.309 | 0.169 | 1 | 1 |
| CFAP36 | 0.000216 | 0.15366656 | 0.539 | 0.381 | 1 | 1 |
| LAMA5 | 0.000216 | 0.17929626 | 0.648 | 0.495 | 1 | 1 |
| TMEM50A | 0.000218 | 0.22177123 | 0.558 | 0.397 | 1 | 1 |
| NUMB | 0.000218 | 0.14085326 | 0.539 | 0.36 | 1 | 1 |
| FAM214A | 0.000225 | 0.13310845 | 0.333 | 0.185 | 1 | 1 |
| CANT1 | 0.000228 | 0.13809813 | 0.461 | 0.299 | 1 | 1 |
| DHCR24 | 0.000236 | 0.17717916 | 0.661 | 0.503 | 1 | 1 |
| CYFIP2 | 0.000237 | 0.18185056 | 0.273 | 0.146 | 1 | 1 |
| TAX1BP1 | 0.000242 | 0.16825196 | 0.83 | 0.733 | 1 | 1 |
| NDUFC2-K( | 0.000243 | 0.193448 | 0.915 | 0.738 | 1 | 1 |
| SNRNP70 | 0.000247 | 0.173777 | 0.442 | 0.291 | 1 | 1 |
| KHNYN | 0.000249 | 0.11637066 | 0.388 | 0.233 | 1 | 1 |

| | | | | | | |
|---|---|---|---|---|---|---|
| ATP9A | 0.00025 | 0.18359971 | 0.745 | 0.614 | 1 | 1 |
| MBOAT7 | 0.00025 | 0.19465616 | 0.77 | 0.664 | 1 | 1 |
| ZNHIT1 | 0.000256 | 0.18315042 | 0.848 | 0.701 | 1 | 1 |
| ST20-MTHF | 0.000258 | 0.15458051 | 0.491 | 0.331 | 1 | 1 |
| ZNF217 | 0.000261 | 0.18397854 | 0.642 | 0.489 | 1 | 1 |
| DDX6 | 0.000264 | 0.16435066 | 0.636 | 0.495 | 1 | 1 |
| HSPA1B | 0.000265 | 0.14915551 | 0.515 | 0.368 | 1 | 1 |
| SPPL2A | 0.000272 | 0.1550035 | 0.855 | 0.704 | 1 | 1 |
| SMAD3 | 0.000273 | 0.10529789 | 0.576 | 0.384 | 1 | 1 |
| RP11-480I1 | 0.000275 | 0.16695912 | 0.806 | 0.683 | 1 | 1 |
| IER5 | 0.00028 | 0.13478498 | 0.291 | 0.161 | 1 | 1 |
| TRAFD1 | 0.000287 | 0.12850509 | 0.406 | 0.251 | 1 | 1 |
| EIF4A2 | 0.000288 | 0.16651407 | 0.794 | 0.648 | 1 | 1 |
| ARL3 | 0.000289 | 0.14677008 | 0.406 | 0.257 | 1 | 1 |
| ATP6V1E1 | 0.000292 | 0.17250044 | 0.806 | 0.712 | 1 | 1 |
| TNFAIP2 | 0.000302 | 0.1840488 | 0.279 | 0.148 | 1 | 1 |
| RP11-79L9. | 0.000306 | 0.13703343 | 0.691 | 0.524 | 1 | 1 |
| MORN2 | 0.000308 | 0.19694735 | 0.412 | 0.267 | 1 | 1 |
| FKBP11 | 0.000315 | 0.20087095 | 0.576 | 0.444 | 1 | 1 |
| ATP5EP2 | 0.000323 | 0.13439852 | 1 | 0.937 | 1 | 1 |
| PPIAP19 | 0.000331 | 0.13767974 | 0.855 | 0.735 | 1 | 1 |
| FRMD4B | 0.000334 | 0.11520829 | 0.333 | 0.193 | 1 | 1 |
| UBE2H | 0.000336 | 0.15395169 | 0.588 | 0.41 | 1 | 1 |
| HEXB | 0.000342 | 0.15927498 | 0.509 | 0.333 | 1 | 1 |
| TRPS1 | 0.000348 | 0.19101449 | 0.539 | 0.397 | 1 | 1 |
| PHTF1 | 0.000352 | 0.16332978 | 0.279 | 0.153 | 1 | 1 |
| HSPE1 | 0.000354 | 0.16248953 | 0.945 | 0.854 | 1 | 1 |
| ICAM1 | 0.000371 | 0.18997583 | 0.467 | 0.315 | 1 | 1 |
| PBDC1 | 0.000375 | 0.19210549 | 0.509 | 0.354 | 1 | 1 |
| SEMA4C | 0.000388 | 0.11684944 | 0.448 | 0.28 | 1 | 1 |
| RAB11A | 0.000404 | 0.15533077 | 0.909 | 0.783 | 1 | 1 |
| LGALS3 | 0.000413 | 0.16013976 | 0.812 | 0.714 | 1 | 1 |
| IER2 | 0.000417 | 0.25019521 | 0.576 | 0.463 | 1 | 1 |
| SEM1 | 0.00042 | 0.12349844 | 0.976 | 0.939 | 1 | 1 |
| PMEPA1 | 0.000433 | 0.13382159 | 0.715 | 0.524 | 1 | 1 |
| TTC39C | 0.000441 | 0.09937793 | 0.321 | 0.177 | 1 | 1 |
| SLC43A2 | 0.000445 | 0.13026373 | 0.321 | 0.185 | 1 | 1 |
| RNF5 | 0.000445 | 0.13978388 | 0.503 | 0.328 | 1 | 1 |
| MTCO1P40 | 0.000452 | 0.14081901 | 0.994 | 0.958 | 1 | 1 |
| NBPF14 | 0.000457 | 0.14541238 | 0.739 | 0.619 | 1 | 1 |
| RSF1 | 0.000467 | 0.20917903 | 0.624 | 0.492 | 1 | 1 |
| ACBD3 | 0.000476 | 0.12894207 | 0.552 | 0.389 | 1 | 1 |
| ATOX1 | 0.00048 | 0.20390661 | 0.679 | 0.558 | 1 | 1 |
| ABI2 | 0.000482 | 0.17446606 | 0.594 | 0.434 | 1 | 1 |
| NIT2 | 0.000485 | 0.17288518 | 0.533 | 0.389 | 1 | 1 |
| TCEAL3 | 0.00049 | 0.14256202 | 0.43 | 0.28 | 1 | 1 |
| IK | 0.000494 | 0.18790626 | 0.588 | 0.431 | 1 | 1 |

| | | | | | | |
|---|---|---|---|---|---|---|
| KIF5C | 0.000498 | 0.15674676 | 0.309 | 0.185 | 1 | 1 |
| FTH1P1 | 0.000504 | 0.1266279 | 0.533 | 0.376 | 1 | 1 |
| ADD3 | 0.000505 | 0.16928633 | 0.273 | 0.148 | 1 | 1 |
| CLOCK | 0.000514 | 0.17590376 | 0.533 | 0.389 | 1 | 1 |
| RP11-212P | 0.000534 | 0.1434827 | 0.406 | 0.265 | 1 | 1 |
| KCTD2 | 0.000539 | 0.10225263 | 0.333 | 0.193 | 1 | 1 |
| DDR1 | 0.000557 | 0.16403816 | 0.709 | 0.55 | 1 | 1 |
| MRPL39 | 0.000563 | 0.09650372 | 0.285 | 0.156 | 1 | 1 |
| IFITM3 | 0.00058 | 0.16773433 | 0.964 | 0.944 | 1 | 1 |
| PARK7 | 0.000583 | 0.14838531 | 0.921 | 0.876 | 1 | 1 |
| HSPE1P2 | 0.000589 | 0.13993272 | 0.915 | 0.825 | 1 | 1 |
| UFD1L | 0.000619 | 0.20124806 | 0.733 | 0.616 | 1 | 1 |
| C14orf119 | 0.000657 | 0.18149714 | 0.515 | 0.378 | 1 | 1 |
| UNC50 | 0.000691 | 0.14342566 | 0.364 | 0.222 | 1 | 1 |
| MTCH1 | 0.000691 | 0.15989921 | 0.691 | 0.526 | 1 | 1 |
| PPIAP3 | 0.000702 | 0.13339317 | 0.491 | 0.325 | 1 | 1 |
| TBCA | 0.000712 | 0.13441446 | 0.952 | 0.907 | 1 | 1 |
| ANXA2 | 0.000719 | 0.10275767 | 0.988 | 0.976 | 1 | 1 |
| SELENOW | 0.000721 | 0.15609784 | 0.939 | 0.939 | 1 | 1 |
| ANGEL1 | 0.000738 | 0.16699588 | 0.345 | 0.209 | 1 | 1 |
| FOXO3 | 0.00076 | 0.16175819 | 0.582 | 0.426 | 1 | 1 |
| ZMIZ2 | 0.000802 | 0.18978501 | 0.673 | 0.553 | 1 | 1 |
| FXYD3 | 0.000807 | 0.28776251 | 0.436 | 0.31 | 1 | 1 |
| SUZ12 | 0.000814 | 0.12493957 | 0.412 | 0.27 | 1 | 1 |
| CBX6 | 0.000818 | 0.16785491 | 0.642 | 0.511 | 1 | 1 |
| SAT1 | 0.000836 | 0.18592311 | 0.558 | 0.405 | 1 | 1 |
| GATA3 | 0.000842 | 0.13676163 | 0.873 | 0.735 | 1 | 1 |
| PDCD6 | 0.000845 | 0.15903173 | 0.958 | 0.918 | 1 | 1 |
| PIN4 | 0.000852 | 0.17828867 | 0.691 | 0.542 | 1 | 1 |
| NECAP2 | 0.000873 | 0.14946521 | 0.321 | 0.19 | 1 | 1 |
| GALNT2 | 0.000884 | 0.13667226 | 0.461 | 0.317 | 1 | 1 |
| DAG1 | 0.000886 | 0.14012371 | 0.448 | 0.296 | 1 | 1 |
| MELTF | 0.000892 | 0.11096911 | 0.279 | 0.153 | 1 | 1 |
| YIPF3 | 0.0009 | 0.18837913 | 0.533 | 0.41 | 1 | 1 |
| SEC61A2 | 0.000907 | 0.09189815 | 0.291 | 0.161 | 1 | 1 |
| FIP1L1 | 0.000912 | 0.09988457 | 0.303 | 0.175 | 1 | 1 |
| OS9 | 0.000921 | 0.1247362 | 0.424 | 0.283 | 1 | 1 |
| OAZ2 | 0.000924 | 0.15487864 | 0.727 | 0.582 | 1 | 1 |
| ZNF226 | 0.000929 | 0.13724687 | 0.297 | 0.169 | 1 | 1 |
| NFKB1 | 0.000956 | 0.17503518 | 0.588 | 0.439 | 1 | 1 |
| NDUFS4 | 0.000972 | 0.16801027 | 0.606 | 0.447 | 1 | 1 |
| BCL2L11 | 0.001015 | 0.10980574 | 0.333 | 0.193 | 1 | 1 |
| USP22 | 0.001016 | 0.18091259 | 0.794 | 0.643 | 1 | 1 |
| PSPC1 | 0.001026 | 0.12371719 | 0.321 | 0.188 | 1 | 1 |
| PAF1 | 0.001033 | 0.13406919 | 0.333 | 0.198 | 1 | 1 |
| B4GALT5 | 0.001045 | 0.16681712 | 0.679 | 0.537 | 1 | 1 |
| RP11-386M | 0.001049 | 0.11583502 | 0.424 | 0.275 | 1 | 1 |

| | | | | | | |
|---|---|---|---|---|---|---|
| PTPRF | 0.001051 | 0.23546794 | 0.6 | 0.468 | 1 | 1 |
| GNPTG | 0.001055 | 0.15751164 | 0.539 | 0.41 | 1 | 1 |
| NDUFS5P5 | 0.001067 | 0.16619573 | 0.752 | 0.611 | 1 | 1 |
| RP11-270C | 0.001072 | 0.14325677 | 0.97 | 0.852 | 1 | 1 |
| ZNF609 | 0.001091 | 0.10846932 | 0.333 | 0.198 | 1 | 1 |
| TBC1D9 | 0.001092 | 0.1734459 | 0.594 | 0.455 | 1 | 1 |
| NBR1 | 0.001122 | 0.16118165 | 0.358 | 0.228 | 1 | 1 |
| MFSD3 | 0.001133 | 0.11267294 | 0.267 | 0.151 | 1 | 1 |
| PPIAL4D | 0.001161 | 0.10657576 | 0.491 | 0.331 | 1 | 1 |
| LINC02043 | 0.001208 | 0.10812597 | 0.345 | 0.212 | 1 | 1 |
| CTB-102L5. | 0.001235 | 0.17953055 | 0.539 | 0.426 | 1 | 1 |
| MAU2 | 0.001258 | 0.13240194 | 0.309 | 0.196 | 1 | 1 |
| MTND6P3 | 0.001287 | 0.12100823 | 0.473 | 0.323 | 1 | 1 |
| FKBP2 | 0.001295 | 0.17560331 | 0.57 | 0.447 | 1 | 1 |
| MAP4 | 0.001301 | 0.14729776 | 0.582 | 0.442 | 1 | 1 |
| IRF3 | 0.001309 | 0.14122789 | 0.345 | 0.23 | 1 | 1 |
| PSMA4 | 0.00131 | 0.12493092 | 0.927 | 0.823 | 1 | 1 |
| GOLPH3L | 0.001311 | 0.10827118 | 0.309 | 0.185 | 1 | 1 |
| MAGED2 | 0.001312 | 0.18674922 | 0.497 | 0.354 | 1 | 1 |
| SNX19 | 0.001347 | 0.12817681 | 0.394 | 0.251 | 1 | 1 |
| RHBDD2 | 0.001356 | 0.15143597 | 0.618 | 0.479 | 1 | 1 |
| HSPE1-MO | 0.001362 | 0.15057875 | 0.873 | 0.746 | 1 | 1 |
| GCA | 0.00137 | 0.09509169 | 0.364 | 0.228 | 1 | 1 |
| NOMO2 | 0.00137 | 0.15076255 | 0.873 | 0.728 | 1 | 1 |
| GNAS | 0.001415 | 0.05873721 | 1 | 1 | 1 | 1 |
| STK10 | 0.001422 | 0.10078178 | 0.255 | 0.146 | 1 | 1 |
| RPN2 | 0.001441 | 0.16160588 | 0.806 | 0.722 | 1 | 1 |
| MTF2 | 0.00146 | 0.08702801 | 0.267 | 0.146 | 1 | 1 |
| RP11-402P | 0.00152 | 0.15139497 | 0.606 | 0.474 | 1 | 1 |
| INTS6 | 0.001531 | 0.12911195 | 0.418 | 0.275 | 1 | 1 |
| RP11-165H | 0.00154 | 0.14765024 | 0.473 | 0.336 | 1 | 1 |
| RDX | 0.00155 | 0.15927815 | 0.655 | 0.526 | 1 | 1 |
| TCEAL1 | 0.001604 | 0.13178777 | 0.412 | 0.272 | 1 | 1 |
| APP | 0.001609 | 0.16258426 | 0.764 | 0.63 | 1 | 1 |
| VEZF1 | 0.00163 | 0.16051038 | 0.412 | 0.291 | 1 | 1 |
| H3F3C | 0.001632 | 0.1154646 | 0.958 | 0.899 | 1 | 1 |
| DNAJA1 | 0.001636 | 0.19863448 | 0.745 | 0.68 | 1 | 1 |
| RABAC1 | 0.001646 | 0.12723108 | 0.545 | 0.394 | 1 | 1 |
| NRCAM | 0.00167 | 0.11841521 | 0.303 | 0.18 | 1 | 1 |
| ARF3 | 0.001684 | 0.12337406 | 0.885 | 0.825 | 1 | 1 |
| RAI1 | 0.001693 | 0.15225042 | 0.412 | 0.283 | 1 | 1 |
| ATM | 0.001701 | 0.1341534 | 0.406 | 0.27 | 1 | 1 |
| TPP1 | 0.001702 | 0.1345905 | 0.309 | 0.188 | 1 | 1 |
| ATP6AP2 | 0.001712 | 0.13011975 | 0.776 | 0.635 | 1 | 1 |
| C12orf57 | 0.001716 | 0.13395876 | 0.648 | 0.5 | 1 | 1 |
| SLC2A6 | 0.001763 | 0.19579102 | 0.57 | 0.447 | 1 | 1 |
| BCL9L | 0.001805 | 0.10540281 | 0.57 | 0.423 | 1 | 1 |

| | | | | | | |
|---|---|---|---|---|---|---|
| DDX5 | 0.001851 | 0.11945791 | 0.964 | 0.921 | 1 | 1 |
| LUZP1 | 0.00187 | 0.10259383 | 0.255 | 0.143 | 1 | 1 |
| DYNC1H1 | 0.001892 | 0.12742059 | 0.691 | 0.521 | 1 | 1 |
| CUX1 | 0.001928 | 0.14216286 | 0.709 | 0.593 | 1 | 1 |
| SERF1A | 0.001932 | 0.17736014 | 0.727 | 0.624 | 1 | 1 |
| NDUFB6 | 0.001955 | 0.15366371 | 0.655 | 0.5 | 1 | 1 |
| SCAF11 | 0.001983 | 0.1419689 | 0.539 | 0.41 | 1 | 1 |
| C12orf76 | 0.001992 | 0.11602345 | 0.255 | 0.146 | 1 | 1 |
| S100A11 | 0.002027 | 0.11351085 | 1 | 0.971 | 1 | 1 |
| ATP6V1F | 0.002031 | 0.18775217 | 0.8 | 0.717 | 1 | 1 |
| ERBB2 | 0.002063 | 0.13249458 | 0.327 | 0.212 | 1 | 1 |
| HSPA8P5 | 0.002066 | 0.11267852 | 0.515 | 0.365 | 1 | 1 |
| CTC-470C1 | 0.002076 | 0.10498508 | 0.297 | 0.188 | 1 | 1 |
| RP11-686G | 0.002078 | 0.1585785 | 0.691 | 0.55 | 1 | 1 |
| QPRT | 0.002086 | 0.16126275 | 0.618 | 0.497 | 1 | 1 |
| ZNF431 | 0.002104 | 0.08952296 | 0.345 | 0.217 | 1 | 1 |
| HIST1H2AC | 0.002132 | 0.1055252 | 0.333 | 0.204 | 1 | 1 |
| ACSL3 | 0.002145 | 0.11019151 | 0.539 | 0.399 | 1 | 1 |
| ARPC3P1 | 0.002165 | 0.07400759 | 0.273 | 0.159 | 1 | 1 |
| NBDY | 0.002179 | 0.14649318 | 0.782 | 0.709 | 1 | 1 |
| CERS6-AS1 | 0.002185 | 0.09607496 | 0.406 | 0.275 | 1 | 1 |
| SNIP1 | 0.002187 | 0.10128801 | 0.267 | 0.159 | 1 | 1 |
| AP002381.: | 0.002197 | 0.09971219 | 0.436 | 0.302 | 1 | 1 |
| TNFAIP3 | 0.002222 | 0.14138149 | 0.533 | 0.386 | 1 | 1 |
| ZNF664 | 0.002222 | 0.12671641 | 0.655 | 0.513 | 1 | 1 |
| SUB1P3 | 0.00223 | 0.11074452 | 0.473 | 0.317 | 1 | 1 |
| CD47 | 0.002266 | 0.18479927 | 0.915 | 0.796 | 1 | 1 |
| BAG3 | 0.002278 | 0.17461247 | 0.418 | 0.304 | 1 | 1 |
| UBE2L3 | 0.002313 | 0.11752861 | 0.933 | 0.892 | 1 | 1 |
| IFNGR2 | 0.00234 | 0.08308549 | 0.339 | 0.212 | 1 | 1 |
| ORAI1 | 0.002348 | 0.09827893 | 0.37 | 0.233 | 1 | 1 |
| GOSR1 | 0.002349 | 0.14416567 | 0.57 | 0.439 | 1 | 1 |
| AAMDC | 0.002398 | 0.07722651 | 0.303 | 0.177 | 1 | 1 |
| BUD31 | 0.002403 | 0.14381605 | 0.915 | 0.847 | 1 | 1 |
| IGF2R | 0.002452 | 0.15167366 | 0.4 | 0.275 | 1 | 1 |
| STX6 | 0.002522 | 0.10153064 | 0.376 | 0.246 | 1 | 1 |
| TMEM219 | 0.00253 | 0.11845483 | 0.297 | 0.18 | 1 | 1 |
| MAP1LC3B | 0.002544 | 0.1634891 | 0.467 | 0.336 | 1 | 1 |
| RNF4 | 0.002582 | 0.11737604 | 0.527 | 0.381 | 1 | 1 |
| IFT43 | 0.002629 | 0.17754114 | 0.527 | 0.407 | 1 | 1 |
| LAMB1 | 0.002647 | 0.15022812 | 0.467 | 0.347 | 1 | 1 |
| NDUFB9 | 0.002678 | 0.1151572 | 0.976 | 0.915 | 1 | 1 |
| RP11-485M | 0.002698 | 0.14686397 | 0.867 | 0.728 | 1 | 1 |
| UBE2L5P | 0.00273 | 0.10955424 | 0.588 | 0.444 | 1 | 1 |
| KIDINS220 | 0.002733 | 0.15988253 | 0.424 | 0.299 | 1 | 1 |
| TFF1 | 0.002781 | 0.19312409 | 0.952 | 0.894 | 1 | 1 |
| AREL1 | 0.002789 | 0.1692303 | 0.436 | 0.317 | 1 | 1 |

| | | | | | | |
|---|---|---|---|---|---|---|
| TCEAL4 | 0.002808 | 0.12272103 | 0.897 | 0.831 | 1 | 1 |
| TM2D1 | 0.002816 | 0.11247886 | 0.418 | 0.296 | 1 | 1 |
| MRPL41 | 0.002841 | 0.12077704 | 0.794 | 0.688 | 1 | 1 |
| NEK5 | 0.002934 | 0.11436514 | 0.315 | 0.201 | 1 | 1 |
| TMEM208 | 0.002935 | 0.11348975 | 0.352 | 0.225 | 1 | 1 |
| SPR | 0.002944 | 0.11689924 | 0.564 | 0.423 | 1 | 1 |
| BLOC1S6 | 0.002947 | 0.11245324 | 0.594 | 0.45 | 1 | 1 |
| TSC22D1 | 0.002953 | 0.15076999 | 0.576 | 0.463 | 1 | 1 |
| RBM8B | 0.002965 | 0.13355423 | 0.812 | 0.667 | 1 | 1 |
| ATP5H | 0.003 | 0.12683237 | 0.897 | 0.823 | 1 | 1 |
| FGD6 | 0.003002 | 0.13708152 | 0.442 | 0.31 | 1 | 1 |
| MRNIP | 0.003014 | 0.15215085 | 0.83 | 0.675 | 1 | 1 |
| PCSK1N | 0.003018 | 0.09900689 | 0.285 | 0.177 | 1 | 1 |
| WWC3 | 0.00306 | 0.1722631 | 0.57 | 0.447 | 1 | 1 |
| SQSTM1 | 0.003073 | 0.12703813 | 0.83 | 0.701 | 1 | 1 |
| ATP6AP1 | 0.003087 | 0.10762615 | 0.873 | 0.759 | 1 | 1 |
| GSTO1 | 0.003093 | 0.11679331 | 0.648 | 0.503 | 1 | 1 |
| FAM134A | 0.003097 | 0.11337063 | 0.267 | 0.159 | 1 | 1 |
| RP11-21J1[illegible] | 0.003109 | 0.14678278 | 0.57 | 0.444 | 1 | 1 |
| ZNF267 | 0.00311 | 0.11107404 | 0.267 | 0.161 | 1 | 1 |
| PDIA3 | 0.003137 | 0.11974252 | 0.618 | 0.466 | 1 | 1 |
| PSME2P2 | 0.00315 | 0.09468257 | 0.473 | 0.328 | 1 | 1 |
| TMEM120[illegible] | 0.003163 | 0.11343629 | 0.418 | 0.291 | 1 | 1 |
| S100A8 | 0.003168 | 0.19090529 | 0.588 | 0.447 | 1 | 1 |
| WBP1 | 0.003191 | 0.16938921 | 0.618 | 0.481 | 1 | 1 |
| NFIB | 0.003273 | 0.13443872 | 0.642 | 0.529 | 1 | 1 |
| EIF1P7 | 0.003304 | 0.10119948 | 0.255 | 0.148 | 1 | 1 |
| RP11-134F[illegible] | 0.003325 | 0.09108796 | 0.376 | 0.257 | 1 | 1 |
| GNS | 0.00336 | 0.11326773 | 0.521 | 0.384 | 1 | 1 |
| CNKSR3 | 0.003374 | 0.10587233 | 0.339 | 0.23 | 1 | 1 |
| BMPR2 | 0.003384 | 0.11252588 | 0.339 | 0.22 | 1 | 1 |
| MB | 0.003404 | 0.09667882 | 0.315 | 0.196 | 1 | 1 |
| TUBA1C | 0.00343 | 0.09030477 | 0.97 | 0.963 | 1 | 1 |
| CDK13 | 0.003475 | 0.08756043 | 0.315 | 0.196 | 1 | 1 |
| TMEM2 | 0.003501 | 0.10242014 | 0.352 | 0.225 | 1 | 1 |
| RP11-835E[illegible] | 0.003515 | 0.11085241 | 0.927 | 0.86 | 1 | 1 |
| NOMO3 | 0.003553 | 0.13725806 | 0.776 | 0.675 | 1 | 1 |
| TUBA1A | 0.003608 | 0.15053813 | 0.976 | 0.921 | 1 | 1 |
| PPIAL4A | 0.003627 | 0.10259774 | 0.679 | 0.521 | 1 | 1 |
| PPP2R5C | 0.003644 | 0.13723796 | 0.721 | 0.577 | 1 | 1 |
| PTK6 | 0.003657 | 0.08626021 | 0.279 | 0.164 | 1 | 1 |
| PPIA | 0.003657 | 0.05200965 | 1 | 1 | 1 | 1 |
| CALR | 0.003669 | 0.17123774 | 0.8 | 0.706 | 1 | 1 |
| TRAK1 | 0.00369 | 0.11685575 | 0.309 | 0.204 | 1 | 1 |
| ATP5J2P5 | 0.003731 | 0.08461724 | 0.503 | 0.333 | 1 | 1 |
| PTCD1 | 0.003776 | 0.12158678 | 0.903 | 0.841 | 1 | 1 |
| ATP1A1 | 0.003828 | 0.12554257 | 0.903 | 0.825 | 1 | 1 |

| | | | | | | |
|---|---|---|---|---|---|---|
| ZBTB43 | 0.003862 | 0.11330405 | 0.279 | 0.172 | 1 | 1 |
| CYB561 | 0.003867 | 0.13065062 | 0.703 | 0.577 | 1 | 1 |
| SIK2 | 0.003873 | 0.08100182 | 0.273 | 0.161 | 1 | 1 |
| MCM3AP | 0.003885 | 0.07854259 | 0.309 | 0.193 | 1 | 1 |
| BRD4 | 0.00395 | 0.1232238 | 0.521 | 0.381 | 1 | 1 |
| TAX1BP3 | 0.004013 | 0.13447194 | 0.667 | 0.595 | 1 | 1 |
| SF3B6 | 0.004084 | 0.10233869 | 0.897 | 0.796 | 1 | 1 |
| TRIM33 | 0.004172 | 0.15216502 | 0.903 | 0.825 | 1 | 1 |
| NDUFA2 | 0.004251 | 0.1621013 | 0.715 | 0.598 | 1 | 1 |
| MAP4K4 | 0.004265 | 0.0839587 | 0.57 | 0.434 | 1 | 1 |
| UPF2 | 0.0043 | 0.13546392 | 0.418 | 0.296 | 1 | 1 |
| IAH1 | 0.004338 | 0.10653356 | 0.679 | 0.548 | 1 | 1 |
| ACAP2 | 0.004375 | 0.10050175 | 0.339 | 0.222 | 1 | 1 |
| PDPR | 0.004382 | 0.1123027 | 0.321 | 0.206 | 1 | 1 |
| RP11-762I7 | 0.004422 | 0.09838478 | 0.358 | 0.238 | 1 | 1 |
| CTSB | 0.004495 | 0.13671945 | 0.436 | 0.312 | 1 | 1 |
| RAB13 | 0.0045 | 0.11375683 | 0.921 | 0.831 | 1 | 1 |
| CH507-42P | 0.004583 | 0.0985837 | 0.503 | 0.36 | 1 | 1 |
| RPL36AP21 | 0.004594 | 0.09814186 | 0.406 | 0.291 | 1 | 1 |
| CHDH | 0.0046 | 0.13015472 | 0.309 | 0.198 | 1 | 1 |
| NDFIP1 | 0.004617 | 0.15284507 | 0.533 | 0.423 | 1 | 1 |
| C16orf62 | 0.004617 | 0.10982908 | 0.364 | 0.246 | 1 | 1 |
| C2CD3 | 0.004625 | 0.17581823 | 0.448 | 0.339 | 1 | 1 |
| SETD6 | 0.004644 | 0.08626673 | 0.455 | 0.328 | 1 | 1 |
| RNF19A | 0.004675 | 0.09412242 | 0.309 | 0.201 | 1 | 1 |
| COL27A1 | 0.004691 | 0.08436864 | 0.291 | 0.183 | 1 | 1 |
| ZG16B | 0.004711 | 0.11095989 | 0.273 | 0.169 | 1 | 1 |
| MAST4 | 0.004758 | 0.09408574 | 0.291 | 0.183 | 1 | 1 |
| MXD4 | 0.004777 | 0.06666093 | 0.533 | 0.378 | 1 | 1 |
| BSDC1 | 0.004779 | 0.0957827 | 0.424 | 0.294 | 1 | 1 |
| TIMP2 | 0.004877 | 0.20345112 | 0.455 | 0.341 | 1 | 1 |
| TPT1 | 0.004931 | 0.06834828 | 1 | 0.997 | 1 | 1 |
| MTND4P35 | 0.005009 | 0.10697345 | 1 | 0.968 | 1 | 1 |
| KYNU | 0.005046 | 0.14292317 | 0.903 | 0.831 | 1 | 1 |
| MIEN1 | 0.005054 | 0.13505607 | 0.752 | 0.616 | 1 | 1 |
| H2BFS | 0.005055 | 0.09092908 | 0.436 | 0.304 | 1 | 1 |
| CHMP3 | 0.005057 | 0.15532807 | 0.879 | 0.825 | 1 | 1 |
| GOLGB1 | 0.005066 | 0.08450983 | 0.352 | 0.23 | 1 | 1 |
| RYK | 0.005077 | 0.11594251 | 0.315 | 0.209 | 1 | 1 |
| PATZ1 | 0.005082 | 0.08422378 | 0.315 | 0.198 | 1 | 1 |
| GNA12 | 0.005198 | 0.11886468 | 0.467 | 0.336 | 1 | 1 |
| RTF1 | 0.00523 | 0.13533914 | 0.582 | 0.426 | 1 | 1 |
| EIF2S2P4 | 0.005267 | 0.14176238 | 0.703 | 0.569 | 1 | 1 |
| FAM174A | 0.005284 | 0.10631716 | 0.255 | 0.153 | 1 | 1 |
| TRAPPC2B | 0.005332 | 0.09948174 | 0.315 | 0.204 | 1 | 1 |
| CTSC | 0.005341 | 0.1201305 | 0.321 | 0.212 | 1 | 1 |
| FOXK1 | 0.005357 | 0.08680432 | 0.442 | 0.315 | 1 | 1 |

| | | | | | | |
|---|---|---|---|---|---|---|
| AGGF1 | 0.005409 | 0.13153514 | 0.285 | 0.185 | 1 | 1 |
| UTP3 | 0.005419 | 0.10876805 | 0.436 | 0.307 | 1 | 1 |
| PFN2 | 0.005427 | 0.1256051 | 0.861 | 0.807 | 1 | 1 |
| PPIAP9 | 0.005457 | 0.11662745 | 0.848 | 0.738 | 1 | 1 |
| GSTO3P | 0.005586 | 0.08073147 | 0.321 | 0.212 | 1 | 1 |
| EFNA5 | 0.005633 | 0.10331908 | 0.291 | 0.185 | 1 | 1 |
| SETX | 0.005638 | 0.09750355 | 0.285 | 0.175 | 1 | 1 |
| KIAA1328 | 0.005755 | 0.08411219 | 0.436 | 0.302 | 1 | 1 |
| HABP4 | 0.005756 | 0.07284403 | 0.267 | 0.159 | 1 | 1 |
| FOXO1 | 0.00577 | 0.11416618 | 0.442 | 0.325 | 1 | 1 |
| POLR2L | 0.005781 | 0.12739369 | 0.921 | 0.833 | 1 | 1 |
| NBPF20 | 0.00582 | 0.18070903 | 0.8 | 0.685 | 1 | 1 |
| COX5B | 0.005865 | 0.10147603 | 0.939 | 0.815 | 1 | 1 |
| LACTB | 0.005885 | 0.13269529 | 0.558 | 0.442 | 1 | 1 |
| POLR2A | 0.005961 | 0.09262219 | 0.418 | 0.286 | 1 | 1 |
| AGBL5 | 0.006068 | 0.13109716 | 0.558 | 0.45 | 1 | 1 |
| RAB18 | 0.006287 | 0.15094695 | 0.739 | 0.64 | 1 | 1 |
| BID | 0.006331 | 0.15800103 | 0.727 | 0.601 | 1 | 1 |
| RP4-592A1 | 0.006334 | 0.0868788 | 0.382 | 0.254 | 1 | 1 |
| BPTF | 0.006343 | 0.11324626 | 0.642 | 0.521 | 1 | 1 |
| APPBP2 | 0.006349 | 0.14905109 | 0.788 | 0.749 | 1 | 1 |
| NBPF1 | 0.00635 | 0.12841597 | 0.57 | 0.45 | 1 | 1 |
| ORC6 | 0.006384 | 0.09349488 | 0.491 | 0.357 | 1 | 1 |
| POMP | 0.006448 | 0.11591205 | 0.939 | 0.878 | 1 | 1 |
| GGA2 | 0.006454 | 0.0898923 | 0.279 | 0.172 | 1 | 1 |
| AKT1 | 0.006544 | 0.12784939 | 0.727 | 0.632 | 1 | 1 |
| PLBD2 | 0.006545 | 0.08967405 | 0.345 | 0.225 | 1 | 1 |
| EMC3 | 0.006577 | 0.130627 | 0.824 | 0.685 | 1 | 1 |
| KIF1C | 0.006752 | 0.07013725 | 0.552 | 0.407 | 1 | 1 |
| SYNRG | 0.006822 | 0.09198133 | 0.339 | 0.235 | 1 | 1 |
| TUSC3 | 0.006856 | 0.11904941 | 0.624 | 0.474 | 1 | 1 |
| KIF1B | 0.006924 | 0.0872927 | 0.345 | 0.233 | 1 | 1 |
| COX6A1 | 0.006939 | 0.12069855 | 0.97 | 0.918 | 1 | 1 |
| TRIM47 | 0.006986 | 0.08975856 | 0.394 | 0.272 | 1 | 1 |
| SDF4 | 0.006987 | 0.11709448 | 0.521 | 0.384 | 1 | 1 |
| SMIM7 | 0.006997 | 0.11140673 | 0.545 | 0.418 | 1 | 1 |
| CRIP2 | 0.007002 | 0.12696322 | 0.558 | 0.447 | 1 | 1 |
| COL5A1 | 0.007032 | 0.13736206 | 0.479 | 0.341 | 1 | 1 |
| ASPHD1 | 0.007064 | 0.08085415 | 0.315 | 0.204 | 1 | 1 |
| RP11-305B | 0.00707 | 0.09930424 | 0.406 | 0.291 | 1 | 1 |
| NMI | 0.007075 | 0.09433937 | 0.467 | 0.347 | 1 | 1 |
| SMAD6 | 0.007127 | 0.10771962 | 0.291 | 0.19 | 1 | 1 |
| ETFB | 0.007276 | 0.09093598 | 0.794 | 0.704 | 1 | 1 |
| ARFGEF2 | 0.007288 | 0.09692682 | 0.382 | 0.254 | 1 | 1 |
| QSOX1 | 0.007309 | 0.12779577 | 0.576 | 0.466 | 1 | 1 |
| EMC10 | 0.007314 | 0.10072743 | 0.345 | 0.225 | 1 | 1 |
| KPNA2P3 | 0.007394 | 0.06847974 | 0.291 | 0.185 | 1 | 1 |

| | | | | | | |
|---|---|---|---|---|---|---|
| GGNBP2 | 0.007406 | 0.14092303 | 0.424 | 0.312 | 1 | 1 |
| F8A1 | 0.007544 | 0.08249929 | 0.479 | 0.349 | 1 | 1 |
| KCTD20 | 0.007563 | 0.09504503 | 0.57 | 0.458 | 1 | 1 |
| AC111155. | 0.007569 | 0.11389431 | 0.733 | 0.646 | 1 | 1 |
| UQCRB | 0.00757 | 0.13173169 | 0.933 | 0.852 | 1 | 1 |
| CD2BP2 | 0.007582 | 0.08145426 | 0.388 | 0.265 | 1 | 1 |
| NSUN5P2 | 0.007626 | 0.1176971 | 0.636 | 0.532 | 1 | 1 |
| NKAP | 0.007634 | 0.10768048 | 0.491 | 0.368 | 1 | 1 |
| MED13 | 0.007651 | 0.12203254 | 0.733 | 0.608 | 1 | 1 |
| CCDC186 | 0.007781 | 0.08536007 | 0.267 | 0.167 | 1 | 1 |
| KLF10 | 0.007842 | 0.06663199 | 0.406 | 0.28 | 1 | 1 |
| PPP2R1B | 0.007872 | 0.08638442 | 0.279 | 0.177 | 1 | 1 |
| C17orf62 | 0.00788 | 0.11311714 | 0.261 | 0.167 | 1 | 1 |
| MYL12AP1 | 0.007899 | 0.08617769 | 0.327 | 0.217 | 1 | 1 |
| NRIP1 | 0.008004 | 0.06764284 | 0.558 | 0.405 | 1 | 1 |
| POLR2J3 | 0.008033 | 0.13771827 | 0.806 | 0.68 | 1 | 1 |
| WSB2 | 0.008042 | 0.10442096 | 0.515 | 0.381 | 1 | 1 |
| MACF1 | 0.008073 | 0.1328999 | 0.418 | 0.325 | 1 | 1 |
| GPR89A | 0.008155 | 0.07833245 | 0.291 | 0.185 | 1 | 1 |
| SOD2 | 0.008162 | 0.10838553 | 0.976 | 0.899 | 1 | 1 |
| FAM134B | 0.008168 | 0.07966885 | 0.303 | 0.193 | 1 | 1 |
| ZC3HAV1 | 0.008207 | 0.13758086 | 0.448 | 0.331 | 1 | 1 |
| PRKACA | 0.008253 | 0.08677702 | 0.582 | 0.439 | 1 | 1 |
| MDM2 | 0.008305 | 0.10139483 | 0.497 | 0.373 | 1 | 1 |
| GGCX | 0.008327 | 0.11867723 | 0.624 | 0.492 | 1 | 1 |
| UXT | 0.008331 | 0.10633649 | 0.648 | 0.55 | 1 | 1 |
| KIAA0922 | 0.008355 | 0.11684789 | 0.382 | 0.275 | 1 | 1 |
| FOXJ3 | 0.008369 | 0.1254884 | 0.297 | 0.204 | 1 | 1 |
| MINOS1 | 0.008424 | 0.11398903 | 0.794 | 0.698 | 1 | 1 |
| YWHAB | 0.008454 | 0.09291851 | 1 | 0.992 | 1 | 1 |
| ATG14 | 0.008506 | 0.06911079 | 0.255 | 0.156 | 1 | 1 |
| BMI1 | 0.008509 | 0.09507843 | 0.564 | 0.437 | 1 | 1 |
| ARHGEF12 | 0.008672 | 0.13885711 | 0.588 | 0.458 | 1 | 1 |
| ADD1 | 0.008736 | 0.13004736 | 0.479 | 0.37 | 1 | 1 |
| ELOCP2 | 0.008743 | 0.12158642 | 0.739 | 0.598 | 1 | 1 |
| VOPP1 | 0.008791 | 0.1274732 | 0.521 | 0.399 | 1 | 1 |
| KPNA6 | 0.008848 | 0.13196277 | 0.758 | 0.675 | 1 | 1 |
| PIK3R2 | 0.009022 | 0.13333838 | 0.527 | 0.421 | 1 | 1 |
| PACS1 | 0.009068 | 0.12814101 | 0.297 | 0.204 | 1 | 1 |
| EP300 | 0.00908 | 0.08813513 | 0.309 | 0.209 | 1 | 1 |
| CLSTN1 | 0.009133 | 0.04953611 | 0.321 | 0.204 | 1 | 1 |
| SLC38A2 | 0.009155 | 0.1390989 | 0.564 | 0.46 | 1 | 1 |
| PIGN | 0.009187 | 0.11344533 | 0.776 | 0.698 | 1 | 1 |
| SUGP2 | 0.009194 | 0.12108039 | 0.406 | 0.288 | 1 | 1 |
| TERF1 | 0.00923 | 0.08429036 | 0.327 | 0.222 | 1 | 1 |
| TXN | 0.009256 | 0.09507902 | 1 | 0.96 | 1 | 1 |
| TMED4 | 0.009298 | 0.1309124 | 0.527 | 0.407 | 1 | 1 |

| | | | | | | |
|---|---|---|---|---|---|---|
| GNB1 | 0.00931 | 0.11745054 | 0.782 | 0.68 | 1 | 1 |
| GRB14 | 0.009346 | 0.17267034 | 0.358 | 0.259 | 1 | 1 |
| SFSWAP | 0.009383 | 0.11115594 | 0.285 | 0.188 | 1 | 1 |
| GRINA | 0.009396 | 0.11219382 | 0.612 | 0.487 | 1 | 1 |
| TPT1P9 | 0.009426 | 0.10947445 | 0.988 | 0.974 | 1 | 1 |
| VPS53 | 0.009464 | 0.13822228 | 0.412 | 0.312 | 1 | 1 |
| LINC00506 | 0.00965 | 0.08566491 | 0.794 | 0.675 | 1 | 1 |
| C12orf49 | 0.009672 | 0.11630641 | 0.424 | 0.302 | 1 | 1 |
| NPNT | 0.009714 | 0.11605035 | 0.309 | 0.206 | 1 | 1 |
| RP11-249L2 | 0.009747 | 0.11721969 | 0.733 | 0.616 | 1 | 1 |
| NDUFV2 | 0.009768 | 0.15955073 | 0.558 | 0.439 | 1 | 1 |
| NECTIN2 | 0.009855 | 0.09737613 | 0.521 | 0.402 | 1 | 1 |
| RP11-434D | 0.009884 | 0.1289836 | 0.303 | 0.209 | 1 | 1 |
| RP11-15E1 | 0.009894 | 0.09862961 | 0.745 | 0.611 | 1 | 1 |
| ACTB | 2.21E-20 | 0.40326213 | 1 | 1 | 4.57E-16 | 2 |
| ENO1 | 1.01E-12 | 0.35022332 | 1 | 0.985 | 2.07E-08 | 2 |
| ACTG1 | 1.51E-11 | 0.29415717 | 1 | 1 | 3.13E-07 | 2 |
| HSPD1 | 1.95E-11 | 0.35128378 | 0.989 | 0.967 | 4.03E-07 | 2 |
| CYP1B1 | 4.18E-11 | 0.35412049 | 1 | 0.954 | 8.63E-07 | 2 |
| HNRNPK | 1.33E-10 | 0.35766981 | 0.966 | 0.904 | 2.74E-06 | 2 |
| MTHFD2 | 5.10E-10 | 0.42438321 | 0.966 | 0.855 | 1.05E-05 | 2 |
| TPM1 | 1.27E-09 | 0.47825743 | 0.966 | 0.965 | 2.62E-05 | 2 |
| PDIA6 | 2.89E-09 | 0.37401495 | 0.897 | 0.741 | 5.97E-05 | 2 |
| CD81 | 5.04E-09 | 0.37898221 | 0.92 | 0.831 | 0.000104 | 2 |
| ANP32B | 1.21E-08 | 0.32512603 | 1 | 0.976 | 0.000251 | 2 |
| TSPAN3 | 1.46E-08 | 0.41070197 | 0.759 | 0.548 | 0.000301 | 2 |
| ATP6V1C2 | 2.25E-08 | 0.35071708 | 0.874 | 0.693 | 0.000464 | 2 |
| TRIM16 | 4.25E-08 | 0.33895855 | 1 | 0.912 | 0.000878 | 2 |
| DDX21 | 6.67E-08 | 0.28077863 | 0.989 | 0.939 | 0.001377 | 2 |
| PGD | 7.36E-08 | 0.32510572 | 0.724 | 0.507 | 0.001519 | 2 |
| TRIM16L | 8.92E-08 | 0.34223342 | 0.977 | 0.91 | 0.001841 | 2 |
| SLC1A5 | 1.25E-07 | 0.35812029 | 0.701 | 0.478 | 0.00257 | 2 |
| TMED10 | 1.56E-07 | 0.32478722 | 0.989 | 0.974 | 0.003217 | 2 |
| NELFCD | 2.67E-07 | 0.30672543 | 0.747 | 0.542 | 0.005507 | 2 |
| STIP1 | 2.74E-07 | 0.34343026 | 0.77 | 0.544 | 0.005648 | 2 |
| CEBPB | 3.46E-07 | 0.34182936 | 0.897 | 0.713 | 0.007139 | 2 |
| STC2 | 4.61E-07 | 0.35468136 | 0.655 | 0.417 | 0.009522 | 2 |
| KPNB1 | 4.81E-07 | 0.32421469 | 0.966 | 0.919 | 0.009917 | 2 |
| HNRNPA0 | 5.62E-07 | 0.36668235 | 0.828 | 0.671 | 0.011607 | 2 |
| H2AFY | 9.79E-07 | 0.31642589 | 0.828 | 0.704 | 0.02021 | 2 |
| TMED9 | 1.07E-06 | 0.2761985 | 0.977 | 0.877 | 0.022139 | 2 |
| GTF3A | 1.23E-06 | 0.31821931 | 0.839 | 0.713 | 0.025285 | 2 |
| SCD | 1.49E-06 | 0.29099246 | 0.989 | 0.919 | 0.030854 | 2 |
| IDI1 | 1.54E-06 | 0.36085018 | 0.862 | 0.711 | 0.031815 | 2 |
| ATP6AP1 | 1.55E-06 | 0.38405042 | 0.897 | 0.774 | 0.031939 | 2 |
| EEF2 | 1.96E-06 | 0.23216615 | 0.989 | 1 | 0.040473 | 2 |
| ACTR3 | 2.26E-06 | 0.30862988 | 0.897 | 0.84 | 0.046565 | 2 |

| | | | | | | |
|---|---|---|---|---|---|---|
| RPS6KB1 | 2.38E-06 | 0.28777473 | 0.966 | 0.862 | 0.049063 | 2 |
| HSPA5 | 2.55E-06 | 0.33920343 | 0.862 | 0.761 | 0.052631 | 2 |
| TMEM184/ | 3.11E-06 | 0.23661022 | 0.391 | 0.184 | 0.06421 | 2 |
| SPINT2 | 3.95E-06 | 0.27594013 | 0.977 | 0.945 | 0.081467 | 2 |
| PSAT1 | 5.47E-06 | 0.33913297 | 0.747 | 0.526 | 0.11292 | 2 |
| TRA2B | 5.73E-06 | 0.30137551 | 0.805 | 0.64 | 0.118273 | 2 |
| GDI2 | 5.90E-06 | 0.31356523 | 0.885 | 0.816 | 0.121849 | 2 |
| CD164 | 6.56E-06 | 0.34392774 | 0.816 | 0.596 | 0.135339 | 2 |
| RDH11 | 6.67E-06 | 0.29871998 | 0.862 | 0.708 | 0.13758 | 2 |
| SLC7A11 | 7.11E-06 | 0.30367247 | 0.782 | 0.561 | 0.14675 | 2 |
| PGK1 | 7.37E-06 | 0.26576652 | 0.943 | 0.934 | 0.152013 | 2 |
| KRT80 | 8.20E-06 | 0.28492505 | 0.966 | 0.825 | 0.169142 | 2 |
| NPTN | 9.52E-06 | 0.3333559 | 0.724 | 0.564 | 0.196585 | 2 |
| XPOT | 9.69E-06 | 0.26900388 | 0.897 | 0.746 | 0.200033 | 2 |
| PKM | 1.06E-05 | 0.23605814 | 1 | 0.987 | 0.219005 | 2 |
| EIF3B | 1.08E-05 | 0.29444061 | 0.885 | 0.82 | 0.222524 | 2 |
| LRRD1 | 1.14E-05 | 0.29868197 | 0.828 | 0.654 | 0.234608 | 2 |
| TFRC | 1.20E-05 | 0.37916857 | 0.931 | 0.803 | 0.246737 | 2 |
| ZHX1 | 1.30E-05 | 0.32206032 | 0.793 | 0.618 | 0.267833 | 2 |
| FAM49B | 1.41E-05 | 0.29073375 | 0.77 | 0.577 | 0.291359 | 2 |
| MYH9 | 1.45E-05 | 0.29320408 | 0.954 | 0.91 | 0.300199 | 2 |
| RSL24D1 | 1.57E-05 | 0.29924298 | 0.885 | 0.796 | 0.323793 | 2 |
| RPN1 | 1.76E-05 | 0.265283 | 0.448 | 0.246 | 0.363006 | 2 |
| YBX1 | 1.85E-05 | 0.19641938 | 1 | 0.98 | 0.381561 | 2 |
| CHAC1 | 1.86E-05 | 0.224006 | 0.322 | 0.138 | 0.383748 | 2 |
| ACTN1 | 1.91E-05 | 0.29788592 | 0.977 | 0.98 | 0.393226 | 2 |
| DDIT4 | 1.91E-05 | 0.41379531 | 0.678 | 0.5 | 0.394314 | 2 |
| DUSP4 | 2.30E-05 | 0.26413289 | 0.713 | 0.529 | 0.474499 | 2 |
| EIF3I | 2.39E-05 | 0.20909442 | 0.77 | 0.55 | 0.49356 | 2 |
| MRFAP1 | 2.51E-05 | 0.23700057 | 0.989 | 0.932 | 0.517265 | 2 |
| KANTR | 2.73E-05 | 0.23907249 | 0.874 | 0.803 | 0.563461 | 2 |
| OAT | 2.85E-05 | 0.39769948 | 0.655 | 0.476 | 0.588862 | 2 |
| NRAS | 3.24E-05 | 0.24118834 | 0.977 | 0.939 | 0.668452 | 2 |
| OLA1 | 3.32E-05 | 0.27166965 | 0.874 | 0.783 | 0.685641 | 2 |
| PRSS8 | 3.61E-05 | 0.29100633 | 0.632 | 0.436 | 0.744922 | 2 |
| SLC39A1 | 3.76E-05 | 0.2727452 | 0.874 | 0.757 | 0.775063 | 2 |
| SERBP1 | 4.23E-05 | 0.22261293 | 0.966 | 0.93 | 0.872547 | 2 |
| ZYX | 4.57E-05 | 0.31163481 | 0.782 | 0.654 | 0.943155 | 2 |
| ASS1 | 4.94E-05 | 0.21910911 | 0.966 | 0.971 | 1 | 2 |
| CSDE1 | 5.02E-05 | 0.1508864 | 1 | 1 | 1 | 2 |
| G6PD | 5.18E-05 | 0.2622609 | 0.908 | 0.868 | 1 | 2 |
| GK5 | 5.53E-05 | 0.1925978 | 0.356 | 0.173 | 1 | 2 |
| HNRNPA3 | 5.95E-05 | 0.2189777 | 0.989 | 0.958 | 1 | 2 |
| PYCR1 | 6.03E-05 | 0.25273305 | 0.644 | 0.434 | 1 | 2 |
| U2AF2 | 6.19E-05 | 0.26219379 | 0.713 | 0.61 | 1 | 2 |
| MT-CO3 | 6.59E-05 | 0.12896828 | 1 | 0.998 | 1 | 2 |
| AGPAT5 | 6.64E-05 | 0.187823 | 0.437 | 0.23 | 1 | 2 |

| | | | | | | |
|---|---|---|---|---|---|---|
| TNFRSF12A | 7.08E-05 | 0.23436777 | 0.908 | 0.862 | 1 | 2 |
| LPP | 7.31E-05 | 0.24082204 | 0.897 | 0.781 | 1 | 2 |
| TRIB3 | 7.78E-05 | 0.18394402 | 0.506 | 0.298 | 1 | 2 |
| C1QTNF3-A | 7.82E-05 | 0.15219032 | 0.31 | 0.143 | 1 | 2 |
| CKAP4 | 8.07E-05 | 0.22478089 | 0.874 | 0.732 | 1 | 2 |
| SLC7A5 | 8.26E-05 | 0.24995017 | 0.931 | 0.857 | 1 | 2 |
| STARD7 | 8.59E-05 | 0.20450648 | 0.851 | 0.667 | 1 | 2 |
| MARCKS | 8.64E-05 | 0.20954923 | 0.943 | 0.86 | 1 | 2 |
| SHB | 8.73E-05 | 0.16760266 | 0.391 | 0.208 | 1 | 2 |
| RBM14-RBI | 8.89E-05 | 0.28641952 | 0.782 | 0.594 | 1 | 2 |
| RPS29 | 9.29E-05 | 0.1383379 | 1 | 1 | 1 | 2 |
| IFNGR1 | 9.48E-05 | 0.25623495 | 0.667 | 0.487 | 1 | 2 |
| SND1 | 9.87E-05 | 0.27898427 | 0.644 | 0.487 | 1 | 2 |
| RP11-281O | 0.000104 | 0.26807125 | 0.816 | 0.724 | 1 | 2 |
| DHX9 | 0.000106 | 0.27786994 | 0.736 | 0.583 | 1 | 2 |
| MTRNR2L8 | 0.000106 | 0.14292581 | 1 | 0.989 | 1 | 2 |
| CITED4 | 0.000108 | 0.22311776 | 0.414 | 0.243 | 1 | 2 |
| CFL1 | 0.000113 | 0.15802156 | 1 | 1 | 1 | 2 |
| GALNT3 | 0.000113 | 0.23480731 | 0.552 | 0.344 | 1 | 2 |
| MRPL3 | 0.000114 | 0.27427768 | 0.839 | 0.706 | 1 | 2 |
| OCLN | 0.000116 | 0.34615757 | 0.563 | 0.399 | 1 | 2 |
| FKBP4 | 0.000135 | 0.25415148 | 0.839 | 0.754 | 1 | 2 |
| MTRNR2L1 | 0.000137 | 0.1337746 | 1 | 0.996 | 1 | 2 |
| PSPH | 0.000143 | 0.21224257 | 0.609 | 0.43 | 1 | 2 |
| FTH1P10 | 0.000147 | 0.14674857 | 1 | 0.998 | 1 | 2 |
| RBM3 | 0.000151 | 0.23763017 | 0.805 | 0.686 | 1 | 2 |
| PLK2 | 0.000153 | 0.30494586 | 0.621 | 0.469 | 1 | 2 |
| EFEMP1 | 0.000157 | 0.27580442 | 0.437 | 0.27 | 1 | 2 |
| ZNF274 | 0.000159 | 0.19484472 | 0.322 | 0.169 | 1 | 2 |
| LARS | 0.000167 | 0.19087024 | 0.575 | 0.357 | 1 | 2 |
| TPM4 | 0.00018 | 0.21067618 | 0.954 | 0.888 | 1 | 2 |
| MTND2P28 | 0.000183 | 0.16232122 | 1 | 0.989 | 1 | 2 |
| APP | 0.000184 | 0.34474848 | 0.782 | 0.649 | 1 | 2 |
| DDX10 | 0.000191 | 0.19534677 | 0.437 | 0.246 | 1 | 2 |
| PAWR | 0.000197 | 0.26317225 | 0.805 | 0.667 | 1 | 2 |
| CEBPG | 0.000212 | 0.2405845 | 0.54 | 0.368 | 1 | 2 |
| PDIA4 | 0.000214 | 0.24409296 | 0.632 | 0.467 | 1 | 2 |
| SEC11A | 0.000217 | 0.25603781 | 0.793 | 0.693 | 1 | 2 |
| PHKG1 | 0.000221 | 0.22375084 | 0.368 | 0.204 | 1 | 2 |
| CYP51A1 | 0.000223 | 0.2662297 | 0.782 | 0.634 | 1 | 2 |
| MTND4P12 | 0.000238 | 0.18294185 | 1 | 0.98 | 1 | 2 |
| FASN | 0.000246 | 0.27812545 | 0.874 | 0.809 | 1 | 2 |
| ACTN4 | 0.000257 | 0.25210449 | 0.954 | 0.923 | 1 | 2 |
| P4HB | 0.000265 | 0.27281408 | 0.908 | 0.844 | 1 | 2 |
| PREP | 0.000282 | 0.22618573 | 0.46 | 0.289 | 1 | 2 |
| CLDN7 | 0.000289 | 0.25130426 | 0.736 | 0.662 | 1 | 2 |
| HMGN1 | 0.000294 | 0.19420125 | 1 | 0.967 | 1 | 2 |

| | | | | | | |
|---|---|---|---|---|---|---|
| PPP1R15B | 0.000297 | 0.27423848 | 0.529 | 0.36 | 1 | 2 |
| ABCF2 | 0.000311 | 0.22514424 | 0.414 | 0.25 | 1 | 2 |
| AHCYL1 | 0.000324 | 0.25072144 | 0.862 | 0.84 | 1 | 2 |
| MT-ND4 | 0.000328 | 0.12746299 | 1 | 1 | 1 | 2 |
| RBM4 | 0.000337 | 0.26651219 | 0.77 | 0.601 | 1 | 2 |
| CBS | 0.000349 | 0.20300357 | 0.805 | 0.695 | 1 | 2 |
| TMEM267 | 0.000353 | 0.20153472 | 0.379 | 0.211 | 1 | 2 |
| MAEA | 0.00037 | 0.20323212 | 0.506 | 0.325 | 1 | 2 |
| OPHN1 | 0.000371 | 0.22385201 | 0.529 | 0.371 | 1 | 2 |
| ITGB1 | 0.000405 | 0.28238009 | 0.943 | 0.899 | 1 | 2 |
| PGAM1 | 0.000424 | 0.24618676 | 0.989 | 0.921 | 1 | 2 |
| 11-Sep | 0.000426 | 0.21734981 | 0.667 | 0.535 | 1 | 2 |
| CARM1 | 0.00043 | 0.26095008 | 0.552 | 0.41 | 1 | 2 |
| USP14 | 0.000433 | 0.28173033 | 0.805 | 0.684 | 1 | 2 |
| NRDC | 0.000434 | 0.24308901 | 0.609 | 0.439 | 1 | 2 |
| CHRAC1 | 0.000442 | 0.22890485 | 0.402 | 0.243 | 1 | 2 |
| HNRNPA2B | 0.000449 | 0.22196469 | 0.966 | 0.961 | 1 | 2 |
| SESN2 | 0.000459 | 0.16984222 | 0.322 | 0.169 | 1 | 2 |
| RP11-666A | 0.000459 | 0.23694252 | 0.782 | 0.673 | 1 | 2 |
| TNS3 | 0.000485 | 0.24234729 | 0.874 | 0.765 | 1 | 2 |
| MTCO2P2 | 0.000495 | 0.1517187 | 1 | 0.987 | 1 | 2 |
| MT-ND2 | 0.000501 | 0.14519308 | 1 | 0.996 | 1 | 2 |
| ZNF354C | 0.000521 | 0.22124993 | 0.839 | 0.763 | 1 | 2 |
| HMGCS1 | 0.000538 | 0.31432091 | 0.667 | 0.581 | 1 | 2 |
| EPCAM | 0.000544 | 0.20244892 | 0.966 | 0.846 | 1 | 2 |
| MTCO3P12 | 0.000626 | 0.11180187 | 1 | 0.998 | 1 | 2 |
| HNRNPF | 0.000686 | 0.22871261 | 0.828 | 0.728 | 1 | 2 |
| HNRNPC | 0.000713 | 0.19435861 | 0.977 | 0.934 | 1 | 2 |
| ATF4 | 0.000715 | 0.26803738 | 0.782 | 0.721 | 1 | 2 |
| CYR61 | 0.000718 | 0.28465702 | 0.598 | 0.443 | 1 | 2 |
| GLI3 | 0.000747 | 0.18611581 | 0.264 | 0.134 | 1 | 2 |
| XXbac-BPG | 0.00076 | 0.23693714 | 0.437 | 0.294 | 1 | 2 |
| CLUAP1 | 0.000766 | 0.16261416 | 0.287 | 0.145 | 1 | 2 |
| SLC7A1 | 0.000774 | 0.25586155 | 0.586 | 0.441 | 1 | 2 |
| SLC39A14 | 0.000818 | 0.29339454 | 0.69 | 0.542 | 1 | 2 |
| FNBP4 | 0.000819 | 0.16286988 | 0.54 | 0.377 | 1 | 2 |
| DLD | 0.00085 | 0.22879575 | 0.782 | 0.669 | 1 | 2 |
| C1orf122 | 0.000882 | 0.19810989 | 0.782 | 0.691 | 1 | 2 |
| HERPUD1 | 0.00089 | 0.26847838 | 0.414 | 0.257 | 1 | 2 |
| TGOLN2 | 0.000902 | 0.24555252 | 0.736 | 0.682 | 1 | 2 |
| NPM1 | 0.000927 | 0.13521547 | 1 | 1 | 1 | 2 |
| KHSRP | 0.000949 | 0.23266665 | 0.632 | 0.482 | 1 | 2 |
| XBP1 | 0.000951 | 0.19853521 | 0.816 | 0.689 | 1 | 2 |
| PMF1 | 0.000994 | 0.18514358 | 0.575 | 0.384 | 1 | 2 |
| PIGT | 0.001047 | 0.21876559 | 0.609 | 0.491 | 1 | 2 |
| TRIM33 | 0.001056 | 0.18905032 | 0.92 | 0.836 | 1 | 2 |
| SPTSSA | 0.001057 | 0.2664211 | 0.908 | 0.862 | 1 | 2 |

| | | | | | | |
|---|---|---|---|---|---|---|
| CDH1 | 0.001082 | 0.17723752 | 0.828 | 0.721 | 1 | 2 |
| SLC25A6 | 0.00112 | 0.19574497 | 0.908 | 0.842 | 1 | 2 |
| PAAF1 | 0.001129 | 0.14243693 | 0.345 | 0.193 | 1 | 2 |
| MT-CO1 | 0.001154 | 0.11733408 | 1 | 0.996 | 1 | 2 |
| SHMT2 | 0.001179 | 0.17601072 | 0.563 | 0.393 | 1 | 2 |
| MAPK6 | 0.001184 | 0.22746968 | 0.77 | 0.623 | 1 | 2 |
| GARS | 0.001235 | 0.20138617 | 0.897 | 0.831 | 1 | 2 |
| FTH1P20 | 0.001251 | 0.13183406 | 1 | 0.998 | 1 | 2 |
| SNHG5 | 0.001271 | 0.18810588 | 0.989 | 0.98 | 1 | 2 |
| FTH1P15 | 0.001273 | 0.19899953 | 0.989 | 0.928 | 1 | 2 |
| NFE2L2 | 0.001277 | 0.19851697 | 0.701 | 0.539 | 1 | 2 |
| AP5Z1 | 0.001292 | 0.12296733 | 0.264 | 0.134 | 1 | 2 |
| RPP14 | 0.0013 | 0.22702785 | 0.379 | 0.254 | 1 | 2 |
| FTH1 | 0.001305 | 0.10343065 | 1 | 1 | 1 | 2 |
| BZW2 | 0.001325 | 0.22581855 | 0.678 | 0.559 | 1 | 2 |
| LYN | 0.001327 | 0.11565913 | 0.276 | 0.138 | 1 | 2 |
| CPSF4 | 0.001345 | 0.2439067 | 0.494 | 0.349 | 1 | 2 |
| CBSL | 0.001362 | 0.19672701 | 0.77 | 0.695 | 1 | 2 |
| CCT6A | 0.001394 | 0.20214258 | 0.943 | 0.853 | 1 | 2 |
| LSMEM2 | 0.001398 | 0.14167383 | 0.276 | 0.143 | 1 | 2 |
| GORASP2 | 0.00143 | 0.25784012 | 0.678 | 0.581 | 1 | 2 |
| YARS | 0.00149 | 0.23728238 | 0.69 | 0.577 | 1 | 2 |
| KIAA2013 | 0.001525 | 0.22380594 | 0.299 | 0.182 | 1 | 2 |
| KREMEN2 | 0.001557 | 0.1350983 | 0.563 | 0.384 | 1 | 2 |
| CTNNA1 | 0.001596 | 0.19779012 | 0.839 | 0.721 | 1 | 2 |
| CCNI | 0.001598 | 0.18508728 | 0.989 | 0.952 | 1 | 2 |
| SBDS | 0.001639 | 0.20098535 | 0.885 | 0.897 | 1 | 2 |
| TMEM87B | 0.00164 | 0.1765415 | 0.414 | 0.252 | 1 | 2 |
| SYNGR2 | 0.001643 | 0.18062948 | 0.851 | 0.717 | 1 | 2 |
| CLNS1A | 0.001662 | 0.20503114 | 0.77 | 0.675 | 1 | 2 |
| RBBP4 | 0.001673 | 0.19638718 | 0.517 | 0.362 | 1 | 2 |
| ATPAF1 | 0.001705 | 0.14581423 | 0.414 | 0.263 | 1 | 2 |
| BEST1 | 0.001709 | 0.10459356 | 1 | 1 | 1 | 2 |
| VARS | 0.001767 | 0.18318863 | 0.368 | 0.228 | 1 | 2 |
| LOXL2 | 0.00177 | 0.24367299 | 0.575 | 0.41 | 1 | 2 |
| MAFK | 0.00178 | 0.1467761 | 0.276 | 0.147 | 1 | 2 |
| TFG | 0.001797 | 0.21814259 | 0.724 | 0.638 | 1 | 2 |
| RDH14 | 0.001813 | 0.22893149 | 0.402 | 0.268 | 1 | 2 |
| RPL12 | 0.001814 | 0.13387266 | 1 | 1 | 1 | 2 |
| ASNS | 0.001831 | 0.21147685 | 0.862 | 0.75 | 1 | 2 |
| EPPK1 | 0.001843 | 0.19415969 | 0.598 | 0.412 | 1 | 2 |
| STARD5 | 0.001857 | 0.23549132 | 0.586 | 0.441 | 1 | 2 |
| MTATP6P1 | 0.001896 | 0.13538334 | 1 | 0.998 | 1 | 2 |
| NOMO1 | 0.001897 | 0.20945386 | 0.736 | 0.651 | 1 | 2 |
| SF3B3 | 0.001917 | 0.21054536 | 0.736 | 0.632 | 1 | 2 |
| XPNPEP1 | 0.001952 | 0.13919992 | 0.402 | 0.25 | 1 | 2 |
| ACLY | 0.001957 | 0.1860966 | 0.747 | 0.658 | 1 | 2 |

| | | | | | | |
|---|---|---|---|---|---|---|
| SQLE | 0.001958 | 0.29340971 | 0.632 | 0.542 | 1 | 2 |
| BMP7 | 0.002 | 0.27541784 | 0.793 | 0.768 | 1 | 2 |
| MT-ATP6 | 0.002009 | 0.12985196 | 1 | 1 | 1 | 2 |
| CNTD1 | 0.002021 | 0.13307054 | 0.287 | 0.158 | 1 | 2 |
| CDH3 | 0.00204 | 0.23394868 | 0.563 | 0.41 | 1 | 2 |
| TVP23B | 0.002072 | 0.20495616 | 0.471 | 0.34 | 1 | 2 |
| TACSTD2 | 0.002179 | 0.21108387 | 0.828 | 0.752 | 1 | 2 |
| GRPEL2 | 0.002227 | 0.20598655 | 0.391 | 0.246 | 1 | 2 |
| WARS | 0.002231 | 0.21643786 | 0.747 | 0.748 | 1 | 2 |
| ZNF598 | 0.002231 | 0.17262126 | 0.356 | 0.228 | 1 | 2 |
| DKC1 | 0.002242 | 0.25075735 | 0.69 | 0.575 | 1 | 2 |
| TMEM45B | 0.002248 | 0.21865256 | 0.391 | 0.252 | 1 | 2 |
| NUS1 | 0.002254 | 0.27001883 | 0.506 | 0.371 | 1 | 2 |
| UGGT1 | 0.002257 | 0.17038239 | 0.437 | 0.296 | 1 | 2 |
| ELOVL1 | 0.002275 | 0.23289235 | 0.494 | 0.384 | 1 | 2 |
| AHCY | 0.002282 | 0.18876535 | 0.759 | 0.618 | 1 | 2 |
| ABCC3 | 0.002291 | 0.21655567 | 0.747 | 0.596 | 1 | 2 |
| OSTC | 0.002306 | 0.17254324 | 0.839 | 0.728 | 1 | 2 |
| SLC44A1 | 0.002314 | 0.18794321 | 0.874 | 0.746 | 1 | 2 |
| SNX17 | 0.002325 | 0.18588904 | 0.632 | 0.522 | 1 | 2 |
| SSRP1 | 0.002327 | 0.21397404 | 0.529 | 0.408 | 1 | 2 |
| FTH1P3 | 0.002388 | 0.15438391 | 0.989 | 0.985 | 1 | 2 |
| MT-ND5 | 0.002472 | 0.16239269 | 1 | 0.978 | 1 | 2 |
| TARS | 0.00248 | 0.1931502 | 0.874 | 0.814 | 1 | 2 |
| RP4-758J1ɛ | 0.002496 | 0.13048002 | 0.333 | 0.191 | 1 | 2 |
| RP5-1042K | 0.002503 | 0.16629369 | 0.379 | 0.246 | 1 | 2 |
| RPL7L1 | 0.002504 | 0.15017946 | 0.897 | 0.829 | 1 | 2 |
| FDFT1 | 0.002522 | 0.22144119 | 0.736 | 0.627 | 1 | 2 |
| PNISR | 0.002559 | 0.17806219 | 0.563 | 0.436 | 1 | 2 |
| JUNB | 0.00259 | 0.12623874 | 0.632 | 0.441 | 1 | 2 |
| RP11-1112. | 0.002683 | 0.17478869 | 0.655 | 0.485 | 1 | 2 |
| HNRNPAB | 0.002717 | 0.1662382 | 0.897 | 0.868 | 1 | 2 |
| IRF2BP2 | 0.002739 | 0.18069725 | 0.598 | 0.452 | 1 | 2 |
| EEF1A1 | 0.002765 | 0.07253664 | 1 | 1 | 1 | 2 |
| HSPA9 | 0.002776 | 0.32126643 | 0.77 | 0.713 | 1 | 2 |
| CTB-89H12 | 0.002805 | 0.17766582 | 0.77 | 0.638 | 1 | 2 |
| ATP6V1A | 0.002843 | 0.15361004 | 0.851 | 0.654 | 1 | 2 |
| NFX1 | 0.002853 | 0.13202492 | 0.368 | 0.23 | 1 | 2 |
| TRNT1 | 0.002864 | 0.24737055 | 0.494 | 0.382 | 1 | 2 |
| AC016739. | 0.002924 | 0.09015741 | 1 | 1 | 1 | 2 |
| IMP3 | 0.002943 | 0.20465419 | 0.759 | 0.607 | 1 | 2 |
| RBBP7 | 0.003008 | 0.17305535 | 0.736 | 0.59 | 1 | 2 |
| PPP2R5E | 0.003012 | 0.26736447 | 0.759 | 0.667 | 1 | 2 |
| KDM2A | 0.003028 | 0.16619058 | 0.471 | 0.344 | 1 | 2 |
| TCEA1 | 0.003043 | 0.27499494 | 0.552 | 0.447 | 1 | 2 |
| RIC8A | 0.003079 | 0.18518221 | 0.437 | 0.3 | 1 | 2 |
| TMEM154 | 0.003105 | 0.11856534 | 0.253 | 0.132 | 1 | 2 |

| | | | | | | |
|---|---|---|---|---|---|---|
| ID3 | 0.003153 | 0.28198948 | 0.471 | 0.338 | 1 | 2 |
| ALKBH5 | 0.003184 | 0.1973496 | 0.46 | 0.327 | 1 | 2 |
| PRDX3 | 0.003217 | 0.19429469 | 0.805 | 0.711 | 1 | 2 |
| TKFC | 0.003239 | 0.17283109 | 0.483 | 0.331 | 1 | 2 |
| FTH1P16 | 0.003252 | 0.13577102 | 1 | 0.965 | 1 | 2 |
| ESRP1 | 0.003262 | 0.22167858 | 0.471 | 0.333 | 1 | 2 |
| NOB1 | 0.003283 | 0.16144762 | 0.529 | 0.393 | 1 | 2 |
| CORO1C | 0.003317 | 0.22034028 | 0.517 | 0.399 | 1 | 2 |
| PLEKHF2 | 0.003354 | 0.0713945 | 0.379 | 0.228 | 1 | 2 |
| SHISA5 | 0.003362 | 0.1690711 | 0.448 | 0.316 | 1 | 2 |
| BZW1 | 0.003372 | 0.17008562 | 0.851 | 0.82 | 1 | 2 |
| TPBG | 0.003375 | 0.21736026 | 0.506 | 0.362 | 1 | 2 |
| PHGDH | 0.003394 | 0.15420011 | 0.644 | 0.474 | 1 | 2 |
| RCN2 | 0.003506 | 0.25531448 | 0.678 | 0.553 | 1 | 2 |
| RP11-512M | 0.003543 | 0.16633941 | 0.448 | 0.303 | 1 | 2 |
| PPM1G | 0.003567 | 0.1864561 | 0.701 | 0.61 | 1 | 2 |
| NCL | 0.003574 | 0.15084369 | 0.897 | 0.82 | 1 | 2 |
| CDC37 | 0.00363 | 0.21677421 | 0.724 | 0.702 | 1 | 2 |
| TNRC6B | 0.003632 | 0.12436059 | 0.299 | 0.171 | 1 | 2 |
| CD46 | 0.003636 | 0.17818766 | 0.425 | 0.296 | 1 | 2 |
| COTL1 | 0.003697 | 0.4233412 | 0.632 | 0.548 | 1 | 2 |
| ENAH | 0.003701 | 0.24274862 | 0.724 | 0.616 | 1 | 2 |
| PPAT | 0.003702 | 0.20782718 | 0.333 | 0.211 | 1 | 2 |
| SET | 0.003836 | 0.1691483 | 1 | 0.987 | 1 | 2 |
| HSP90B1 | 0.004008 | 0.16836255 | 0.943 | 0.873 | 1 | 2 |
| ERP29 | 0.004008 | 0.22065107 | 0.793 | 0.735 | 1 | 2 |
| SLC12A9 | 0.004018 | 0.13477273 | 0.379 | 0.239 | 1 | 2 |
| ADK | 0.004047 | 0.19481971 | 0.667 | 0.557 | 1 | 2 |
| XRCC6 | 0.004065 | 0.21931486 | 0.701 | 0.621 | 1 | 2 |
| AP1S1 | 0.004072 | 0.15304343 | 0.759 | 0.669 | 1 | 2 |
| MTCO1P12 | 0.004089 | 0.11285149 | 1 | 0.993 | 1 | 2 |
| UBR4 | 0.004091 | 0.19749586 | 0.483 | 0.34 | 1 | 2 |
| STAG2 | 0.004095 | 0.22365579 | 0.46 | 0.325 | 1 | 2 |
| JDP2 | 0.004173 | 0.08493397 | 0.264 | 0.138 | 1 | 2 |
| B3GALNT1 | 0.00429 | 0.21773763 | 0.437 | 0.314 | 1 | 2 |
| RPAP2 | 0.004309 | 0.19871848 | 0.586 | 0.43 | 1 | 2 |
| SMARCA2 | 0.004327 | 0.17077061 | 0.31 | 0.186 | 1 | 2 |
| YWHAG | 0.004336 | 0.16823578 | 0.989 | 0.963 | 1 | 2 |
| LAPTM4B | 0.004371 | 0.19555257 | 0.851 | 0.774 | 1 | 2 |
| ESYT2 | 0.004403 | 0.17315125 | 0.609 | 0.478 | 1 | 2 |
| UTP6 | 0.004428 | 0.16427888 | 0.333 | 0.206 | 1 | 2 |
| STMN3 | 0.004523 | 0.15977341 | 0.425 | 0.281 | 1 | 2 |
| PKP4 | 0.004538 | 0.20841164 | 0.586 | 0.469 | 1 | 2 |
| SERPINH1 | 0.004546 | 0.20231958 | 0.793 | 0.721 | 1 | 2 |
| TMEM123 | 0.004598 | 0.1728949 | 0.874 | 0.86 | 1 | 2 |
| TSPAN13 | 0.004637 | 0.11269866 | 0.736 | 0.649 | 1 | 2 |
| DDOST | 0.004733 | 0.15636842 | 0.713 | 0.577 | 1 | 2 |

| | | | | | | |
|---|---|---|---|---|---|---|
| DIABLO | 0.004738 | 0.20424905 | 0.437 | 0.307 | 1 | 2 |
| SEC23B | 0.004775 | 0.16073893 | 0.529 | 0.382 | 1 | 2 |
| SUV39H2 | 0.004851 | 0.10174743 | 0.276 | 0.149 | 1 | 2 |
| ATP6AP2 | 0.004871 | 0.26134921 | 0.724 | 0.669 | 1 | 2 |
| TMEM248 | 0.004909 | 0.2363322 | 0.782 | 0.682 | 1 | 2 |
| IGFBP2 | 0.004933 | 0.16604291 | 0.494 | 0.364 | 1 | 2 |
| DROSHA | 0.004959 | 0.2190419 | 0.368 | 0.241 | 1 | 2 |
| LYPLA1 | 0.004977 | 0.27290558 | 0.494 | 0.371 | 1 | 2 |
| MTIF2 | 0.004979 | 0.24278816 | 0.494 | 0.366 | 1 | 2 |
| HNRNPA1P | 0.005004 | 0.16216083 | 0.391 | 0.268 | 1 | 2 |
| PTP4A2 | 0.005033 | 0.24272173 | 0.862 | 0.805 | 1 | 2 |
| C21orf33 | 0.005041 | 0.13873269 | 0.54 | 0.395 | 1 | 2 |
| STAU1 | 0.005044 | 0.17235975 | 0.782 | 0.64 | 1 | 2 |
| IARS | 0.005047 | 0.26205521 | 0.805 | 0.77 | 1 | 2 |
| SRSF2 | 0.005104 | 0.29045169 | 0.759 | 0.68 | 1 | 2 |
| FAM98B | 0.005195 | 0.15153159 | 0.437 | 0.314 | 1 | 2 |
| RFWD2 | 0.005198 | 0.18741694 | 0.287 | 0.173 | 1 | 2 |
| BLOC1S5-T | 0.005201 | 0.20348554 | 0.609 | 0.524 | 1 | 2 |
| KLC1 | 0.005242 | 0.22588966 | 0.644 | 0.572 | 1 | 2 |
| PDSS1 | 0.005344 | 0.11635254 | 0.299 | 0.173 | 1 | 2 |
| SLC25A38 | 0.005354 | 0.16660977 | 0.333 | 0.206 | 1 | 2 |
| SURF4 | 0.005365 | 0.174691 | 0.747 | 0.632 | 1 | 2 |
| GNAS | 0.005457 | 0.09811802 | 1 | 1 | 1 | 2 |
| ELAVL1 | 0.005479 | 0.19762804 | 0.69 | 0.601 | 1 | 2 |
| PTGES3 | 0.005486 | 0.18402512 | 0.874 | 0.77 | 1 | 2 |
| FTH1P2 | 0.005545 | 0.1051931 | 1 | 0.993 | 1 | 2 |
| UBE2M | 0.005657 | 0.22715202 | 0.644 | 0.588 | 1 | 2 |
| AC004069. | 0.005665 | 0.21146588 | 0.563 | 0.443 | 1 | 2 |
| NCBP2 | 0.005699 | 0.27763377 | 0.759 | 0.691 | 1 | 2 |
| NDUFA9 | 0.005807 | 0.12183987 | 0.471 | 0.331 | 1 | 2 |
| YY1AP1 | 0.005838 | 0.12016399 | 0.402 | 0.274 | 1 | 2 |
| SPIN1 | 0.005902 | 0.21601926 | 0.609 | 0.485 | 1 | 2 |
| RP11-48B3 | 0.00597 | 0.12802671 | 0.264 | 0.151 | 1 | 2 |
| HSPA8 | 0.00604 | 0.1603152 | 1 | 0.969 | 1 | 2 |
| BCLAF1 | 0.006069 | 0.17548795 | 0.655 | 0.531 | 1 | 2 |
| INTS14 | 0.006118 | 0.22587578 | 0.368 | 0.239 | 1 | 2 |
| SFPQ | 0.006162 | 0.15498264 | 0.724 | 0.607 | 1 | 2 |
| C14orf1 | 0.006179 | 0.18545024 | 0.747 | 0.654 | 1 | 2 |
| SEPHS2 | 0.006182 | 0.13615396 | 0.69 | 0.555 | 1 | 2 |
| UBA1 | 0.006242 | 0.1996895 | 0.805 | 0.737 | 1 | 2 |
| RPLP1 | 0.006271 | 0.05583205 | 1 | 1 | 1 | 2 |
| ZCCHC9 | 0.006275 | 0.22727543 | 0.299 | 0.189 | 1 | 2 |
| IFRD2 | 0.006275 | 0.1439082 | 0.345 | 0.226 | 1 | 2 |
| PEF1 | 0.006315 | 0.13532783 | 0.379 | 0.248 | 1 | 2 |
| IFRD1 | 0.006374 | 0.23873428 | 0.448 | 0.331 | 1 | 2 |
| ATXN2L | 0.00638 | 0.1749839 | 0.517 | 0.406 | 1 | 2 |
| MTRNR2L1 | 0.006385 | 0.12483252 | 1 | 0.996 | 1 | 2 |

| | | | | | | |
|---|---|---|---|---|---|---|
| CMPK1 | 0.00647 | 0.15941658 | 0.609 | 0.485 | 1 | 2 |
| DHRS4L2 | 0.006604 | 0.20850634 | 0.598 | 0.48 | 1 | 2 |
| TOR3A | 0.006623 | 0.13005328 | 0.287 | 0.173 | 1 | 2 |
| SNX13 | 0.006632 | 0.14199834 | 0.322 | 0.204 | 1 | 2 |
| MT-CO2 | 0.006748 | 0.11080197 | 1 | 1 | 1 | 2 |
| SSR1 | 0.006751 | 0.13090138 | 0.793 | 0.697 | 1 | 2 |
| STX12 | 0.006805 | 0.13285556 | 0.471 | 0.333 | 1 | 2 |
| GPT2 | 0.006841 | 0.16674427 | 0.54 | 0.406 | 1 | 2 |
| MALAT1 | 0.00687 | 0.13563249 | 0.908 | 0.818 | 1 | 2 |
| LRPPRC | 0.006986 | 0.17171577 | 0.655 | 0.5 | 1 | 2 |
| POLD2 | 0.007033 | 0.13954962 | 0.425 | 0.311 | 1 | 2 |
| RAB11FIP1 | 0.00707 | 0.15895604 | 0.414 | 0.279 | 1 | 2 |
| CCPG1 | 0.007253 | 0.11557556 | 0.483 | 0.325 | 1 | 2 |
| CCT4 | 0.007272 | 0.16377864 | 0.92 | 0.873 | 1 | 2 |
| WDR3 | 0.007301 | 0.18944271 | 0.563 | 0.41 | 1 | 2 |
| FOXN3 | 0.007325 | 0.16355322 | 0.322 | 0.208 | 1 | 2 |
| TXNDC12 | 0.007336 | 0.24948861 | 0.425 | 0.3 | 1 | 2 |
| CCND1 | 0.007352 | 0.10611166 | 0.747 | 0.603 | 1 | 2 |
| MIEF1 | 0.007516 | 0.12484727 | 0.437 | 0.3 | 1 | 2 |
| PURB | 0.007572 | 0.19443123 | 0.805 | 0.763 | 1 | 2 |
| AC098614. | 0.007605 | 0.1612079 | 0.828 | 0.713 | 1 | 2 |
| SEMA3C | 0.007676 | 0.19541844 | 0.943 | 0.921 | 1 | 2 |
| ACTR1B | 0.007791 | 0.14673092 | 0.345 | 0.219 | 1 | 2 |
| CLIC4 | 0.007907 | 0.23404285 | 0.759 | 0.654 | 1 | 2 |
| CCNC | 0.008037 | 0.1880301 | 0.529 | 0.425 | 1 | 2 |
| NDUFS1 | 0.008072 | 0.15857482 | 0.517 | 0.41 | 1 | 2 |
| RPIA | 0.008131 | 0.20275582 | 0.299 | 0.182 | 1 | 2 |
| KDELR2 | 0.00817 | 0.14667176 | 0.874 | 0.827 | 1 | 2 |
| TRMT12 | 0.008189 | 0.08551389 | 0.322 | 0.195 | 1 | 2 |
| TRIP10 | 0.008199 | 0.17877384 | 0.356 | 0.25 | 1 | 2 |
| FTSJ1 | 0.008201 | 0.15369966 | 0.276 | 0.164 | 1 | 2 |
| NAA50 | 0.008216 | 0.21200429 | 0.724 | 0.651 | 1 | 2 |
| RTCB | 0.008217 | 0.23836402 | 0.632 | 0.544 | 1 | 2 |
| GCLC | 0.008236 | 0.24034879 | 0.46 | 0.353 | 1 | 2 |
| NUP107 | 0.008312 | 0.14977982 | 0.299 | 0.182 | 1 | 2 |
| RBM17 | 0.008413 | 0.16374582 | 0.793 | 0.673 | 1 | 2 |
| NCK1 | 0.008421 | 0.108327 | 0.31 | 0.191 | 1 | 2 |
| SETD3 | 0.008488 | 0.22093242 | 0.563 | 0.48 | 1 | 2 |
| GRB10 | 0.00862 | 0.1087891 | 0.322 | 0.197 | 1 | 2 |
| FAM86C2P | 0.008725 | 0.13028157 | 0.276 | 0.164 | 1 | 2 |
| SLC38A1 | 0.008741 | 0.21586922 | 0.793 | 0.662 | 1 | 2 |
| HSPBP1 | 0.008789 | 0.18506534 | 0.414 | 0.289 | 1 | 2 |
| NAA25 | 0.008887 | 0.13194771 | 0.483 | 0.342 | 1 | 2 |
| GSS | 0.008888 | 0.13897101 | 0.506 | 0.375 | 1 | 2 |
| RSPH10B2 | 0.008943 | 0.1409479 | 0.368 | 0.254 | 1 | 2 |
| AAMP | 0.008988 | 0.1665656 | 0.552 | 0.428 | 1 | 2 |
| IL27RA | 0.00907 | 0.1517375 | 0.448 | 0.327 | 1 | 2 |

| | | | | | | |
|---|---|---|---|---|---|---|
| 5-Mar | 0.009125 | 0.20095804 | 0.517 | 0.406 | 1 | 2 |
| RMND5A | 0.009127 | 0.16205782 | 0.483 | 0.355 | 1 | 2 |
| APMAP | 0.009214 | 0.22690228 | 0.609 | 0.489 | 1 | 2 |
| EIF4EBP1 | 0.009236 | 0.17131751 | 0.701 | 0.55 | 1 | 2 |
| FTH1P11 | 0.009267 | 0.08948028 | 1 | 0.998 | 1 | 2 |
| ANXA7 | 0.009268 | 0.14764387 | 0.724 | 0.616 | 1 | 2 |
| GTF2E2 | 0.009299 | 0.23920698 | 0.494 | 0.406 | 1 | 2 |
| STX16 | 0.009678 | 0.19967727 | 0.782 | 0.695 | 1 | 2 |
| POTEI | 0.009709 | 0.12579166 | 0.425 | 0.311 | 1 | 2 |
| FAM32A | 0.009735 | 0.22700024 | 0.529 | 0.432 | 1 | 2 |
| WDR1 | 0.009738 | 0.17449593 | 0.851 | 0.829 | 1 | 2 |
| MRNIP | 0.009881 | 0.13345938 | 0.793 | 0.708 | 1 | 2 |
| RITA1 | 0.00989 | 0.12204312 | 0.322 | 0.204 | 1 | 2 |
| SNHG16 | 0.009927 | 0.13084863 | 0.874 | 0.789 | 1 | 2 |
| RPL10 | 3.45E-17 | 0.58240157 | 1 | 1 | 7.13E-13 | 3 |
| CTD-2192J1 | 7.02E-17 | 0.92509101 | 0.976 | 0.81 | 1.45E-12 | 3 |
| RPS2 | 2.51E-16 | 0.57723537 | 1 | 1 | 5.19E-12 | 3 |
| RPL10P16 | 5.55E-16 | 0.64605131 | 1 | 0.994 | 1.14E-11 | 3 |
| RP3-342P2 | 1.28E-15 | 0.67449233 | 1 | 0.97 | 2.65E-11 | 3 |
| RPS12 | 2.49E-15 | 0.50671202 | 1 | 1 | 5.14E-11 | 3 |
| RP11-296A | 2.86E-15 | 0.65760059 | 0.976 | 0.976 | 5.91E-11 | 3 |
| RP11-613F | 1.16E-14 | 0.60612731 | 1 | 0.996 | 2.40E-10 | 3 |
| RPS2P7 | 2.81E-14 | 0.58728918 | 1 | 1 | 5.79E-10 | 3 |
| RPS2P5 | 5.23E-14 | 0.53066529 | 1 | 0.992 | 1.08E-09 | 3 |
| RPLP1 | 5.57E-14 | 0.43714234 | 1 | 1 | 1.15E-09 | 3 |
| AC016739. | 1.12E-13 | 0.44868166 | 1 | 1 | 2.32E-09 | 3 |
| RPS2P48 | 1.18E-13 | 0.6588104 | 1 | 0.968 | 2.43E-09 | 3 |
| AC009413. | 1.57E-13 | 0.599492 | 1 | 0.998 | 3.24E-09 | 3 |
| RPL38 | 2.69E-13 | 0.50343559 | 1 | 0.994 | 5.56E-09 | 3 |
| RPS15A | 4.84E-13 | 0.56459119 | 1 | 0.996 | 1.00E-08 | 3 |
| RPL39 | 5.34E-13 | 0.55372734 | 1 | 0.992 | 1.10E-08 | 3 |
| RP11-364L | 5.67E-13 | 0.56119718 | 0.976 | 0.99 | 1.17E-08 | 3 |
| RPS2P46 | 8.73E-13 | 0.50994815 | 1 | 0.996 | 1.80E-08 | 3 |
| RP11-192C | 1.31E-12 | 0.63544783 | 1 | 0.956 | 2.70E-08 | 3 |
| GAPDH | 1.87E-12 | 0.60875597 | 1 | 0.988 | 3.86E-08 | 3 |
| PPIA | 3.33E-12 | 0.33560638 | 1 | 1 | 6.88E-08 | 3 |
| RPL11 | 3.73E-12 | 0.42986996 | 1 | 0.998 | 7.70E-08 | 3 |
| RP5-900K1 | 4.09E-12 | 0.68806565 | 0.976 | 0.934 | 8.44E-08 | 3 |
| UBB | 4.36E-12 | 0.56691034 | 1 | 0.968 | 8.99E-08 | 3 |
| RPL18AP3 | 5.17E-12 | 0.49760608 | 1 | 0.996 | 1.07E-07 | 3 |
| SERF2 | 6.69E-12 | 0.5020054 | 1 | 0.978 | 1.38E-07 | 3 |
| RPS25 | 1.08E-11 | 0.44133743 | 1 | 0.994 | 2.24E-07 | 3 |
| RPS2P4 | 1.11E-11 | 0.60195536 | 0.976 | 0.974 | 2.29E-07 | 3 |
| RPLP0P6 | 1.12E-11 | 0.53959799 | 1 | 0.996 | 2.32E-07 | 3 |
| RPL29 | 1.51E-11 | 0.52829847 | 0.952 | 0.924 | 3.11E-07 | 3 |
| LRRC75A-A | 1.90E-11 | 0.61788186 | 1 | 0.902 | 3.92E-07 | 3 |
| RPL29P11 | 4.01E-11 | 0.6152704 | 0.952 | 0.862 | 8.28E-07 | 3 |

| | | | | | | |
|---|---|---|---|---|---|---|
| RPLP0 | 5.78E-11 | 0.425969 | 1 | 1 | 1.19E-06 | 3 |
| RPS3AP26 | 7.09E-11 | 0.48018689 | 1 | 0.956 | 1.46E-06 | 3 |
| MIF | 7.35E-11 | 0.54481396 | 1 | 0.944 | 1.52E-06 | 3 |
| RPL8 | 8.08E-11 | 0.47533756 | 1 | 1 | 1.67E-06 | 3 |
| PPIAP22 | 9.81E-11 | 0.47904649 | 1 | 0.982 | 2.02E-06 | 3 |
| RPL15 | 1.66E-10 | 0.3110264 | 1 | 0.998 | 3.42E-06 | 3 |
| RPL30P14 | 2.04E-10 | 0.41913183 | 1 | 0.998 | 4.21E-06 | 3 |
| RP11-632C | 2.10E-10 | 0.6214777 | 0.976 | 0.838 | 4.33E-06 | 3 |
| ARPC1B | 2.67E-10 | 0.61995681 | 0.976 | 0.918 | 5.51E-06 | 3 |
| GNB2 | 3.38E-10 | 0.54315431 | 0.929 | 0.699 | 6.97E-06 | 3 |
| CTB-63M2 | 3.97E-10 | 0.46221242 | 1 | 0.984 | 8.18E-06 | 3 |
| AC079250. | 5.00E-10 | 0.46118116 | 1 | 0.992 | 1.03E-05 | 3 |
| TFF1 | 5.08E-10 | 0.79367195 | 1 | 0.904 | 1.05E-05 | 3 |
| RP11-1035 | 5.94E-10 | 0.55463409 | 0.976 | 0.956 | 1.23E-05 | 3 |
| PPIAP31 | 7.74E-10 | 0.51357879 | 1 | 0.954 | 1.60E-05 | 3 |
| NME2 | 9.34E-10 | 0.36510039 | 1 | 0.996 | 1.93E-05 | 3 |
| RPS17 | 9.71E-10 | 0.4465407 | 1 | 0.994 | 2.00E-05 | 3 |
| RPS28 | 1.04E-09 | 0.46333878 | 1 | 0.988 | 2.14E-05 | 3 |
| RPL23AP57 | 1.04E-09 | 0.56921707 | 0.69 | 0.331 | 2.15E-05 | 3 |
| RPL10A | 1.09E-09 | 0.40121925 | 1 | 0.998 | 2.25E-05 | 3 |
| FTL | 1.15E-09 | 0.63716761 | 0.976 | 0.93 | 2.37E-05 | 3 |
| PPIAP11 | 1.37E-09 | 0.49857308 | 1 | 0.942 | 2.82E-05 | 3 |
| PSMA7 | 1.91E-09 | 0.4903373 | 1 | 0.96 | 3.94E-05 | 3 |
| RPL10P9 | 1.95E-09 | 0.51108225 | 1 | 0.974 | 4.02E-05 | 3 |
| RPL18A | 1.99E-09 | 0.44354307 | 1 | 0.994 | 4.10E-05 | 3 |
| RP11-864N | 2.19E-09 | 0.45987297 | 1 | 0.998 | 4.52E-05 | 3 |
| RPL7P9 | 2.23E-09 | 0.32186026 | 1 | 1 | 4.61E-05 | 3 |
| RPL7A | 2.38E-09 | 0.36212258 | 1 | 1 | 4.91E-05 | 3 |
| RP11-234A | 2.80E-09 | 0.42683865 | 1 | 0.996 | 5.77E-05 | 3 |
| RPL10AP2 | 3.08E-09 | 0.41582467 | 1 | 0.998 | 6.36E-05 | 3 |
| MDH2 | 3.14E-09 | 0.48541446 | 0.976 | 0.966 | 6.47E-05 | 3 |
| RPL28 | 3.55E-09 | 0.4417954 | 1 | 0.996 | 7.34E-05 | 3 |
| RPL30 | 4.71E-09 | 0.34903341 | 1 | 1 | 9.72E-05 | 3 |
| ZNF808 | 6.66E-09 | 0.54606856 | 0.952 | 0.844 | 0.000137 | 3 |
| RPSAP58 | 1.08E-08 | 0.46335236 | 0.976 | 0.942 | 0.000223 | 3 |
| EEF1A1P14 | 1.12E-08 | 0.43759004 | 1 | 0.948 | 0.000231 | 3 |
| EEF1A1P13 | 1.47E-08 | 0.41642506 | 1 | 0.976 | 0.000303 | 3 |
| AP000721. | 1.58E-08 | 0.57563111 | 0.81 | 0.613 | 0.000326 | 3 |
| UBA52 | 1.63E-08 | 0.35747395 | 1 | 0.994 | 0.000336 | 3 |
| EEF1A1P19 | 2.12E-08 | 0.4851224 | 1 | 0.94 | 0.000438 | 3 |
| RPL7P23 | 2.44E-08 | 0.35750896 | 1 | 0.996 | 0.000503 | 3 |
| RPS4X | 2.78E-08 | 0.4065865 | 1 | 0.996 | 0.000573 | 3 |
| RP11-761N | 3.01E-08 | 0.45583514 | 0.976 | 0.976 | 0.000622 | 3 |
| RPS18P9 | 3.03E-08 | 0.42573989 | 1 | 0.98 | 0.000625 | 3 |
| RPS13 | 3.89E-08 | 0.35030207 | 1 | 0.998 | 0.000803 | 3 |
| PRDX1 | 4.02E-08 | 0.43981334 | 0.952 | 0.982 | 0.000829 | 3 |
| H3F3A | 4.10E-08 | 0.39356804 | 1 | 0.996 | 0.000846 | 3 |

| | | | | | | |
|---|---|---|---|---|---|---|
| RP3-423B2 | 4.38E-08 | 0.52242443 | 0.976 | 0.886 | 0.000903 | 3 |
| PKIB | 4.51E-08 | 0.55103945 | 0.524 | 0.202 | 0.000931 | 3 |
| RP11-219G | 5.13E-08 | 0.5283609 | 0.881 | 0.762 | 0.001059 | 3 |
| RPL36A-HN | 6.10E-08 | 0.4268857 | 0.976 | 0.98 | 0.001259 | 3 |
| RP11-864I4 | 6.66E-08 | 0.40794925 | 1 | 0.838 | 0.001374 | 3 |
| RPL7 | 6.92E-08 | 0.30989336 | 1 | 1 | 0.001428 | 3 |
| RPL3 | 7.47E-08 | 0.38302463 | 1 | 0.994 | 0.001542 | 3 |
| EEF1A1P6 | 7.93E-08 | 0.35538651 | 1 | 0.996 | 0.001637 | 3 |
| RPS18 | 8.44E-08 | 0.33204755 | 1 | 0.998 | 0.001742 | 3 |
| RPS3AP5 | 8.74E-08 | 0.52028734 | 0.881 | 0.864 | 0.001804 | 3 |
| RPL5 | 8.81E-08 | 0.42382514 | 0.976 | 0.974 | 0.001817 | 3 |
| RPL37P6 | 1.31E-07 | 0.52810357 | 0.952 | 0.886 | 0.002697 | 3 |
| S100A16 | 1.43E-07 | 0.61317004 | 0.952 | 0.89 | 0.002954 | 3 |
| AC025750. | 1.58E-07 | 0.40331475 | 0.976 | 0.938 | 0.003264 | 3 |
| RPL35 | 1.66E-07 | 0.4750227 | 1 | 0.988 | 0.003422 | 3 |
| RPS14 | 1.68E-07 | 0.28292271 | 1 | 1 | 0.003461 | 3 |
| RPL7AP6 | 1.77E-07 | 0.40102502 | 1 | 0.982 | 0.003647 | 3 |
| RP11-543P | 1.89E-07 | 0.4607355 | 1 | 0.974 | 0.003897 | 3 |
| RPL39P3 | 2.40E-07 | 0.42813018 | 0.976 | 0.912 | 0.004952 | 3 |
| RP11-12M9 | 2.66E-07 | 0.37104549 | 0.976 | 0.978 | 0.005491 | 3 |
| RP11-175B | 2.91E-07 | 0.3817243 | 0.976 | 0.944 | 0.006012 | 3 |
| GAPDHP40 | 2.95E-07 | 0.49262324 | 0.881 | 0.784 | 0.006087 | 3 |
| AC007161. | 3.04E-07 | 0.44757601 | 0.595 | 0.293 | 0.006277 | 3 |
| GUK1 | 3.10E-07 | 0.54449375 | 0.905 | 0.88 | 0.006392 | 3 |
| TMSB10 | 3.18E-07 | 0.39555923 | 1 | 0.988 | 0.006554 | 3 |
| EEF1A1 | 3.20E-07 | 0.25870617 | 1 | 1 | 0.006607 | 3 |
| AP000350. | 3.40E-07 | 0.48474946 | 1 | 0.928 | 0.007023 | 3 |
| RACK1 | 3.64E-07 | 0.37389215 | 0.976 | 0.994 | 0.007503 | 3 |
| TOMM7 | 3.86E-07 | 0.29453886 | 1 | 0.97 | 0.007959 | 3 |
| RPSA | 4.08E-07 | 0.43000081 | 1 | 0.99 | 0.008424 | 3 |
| CTD-2161E | 4.68E-07 | 0.3807627 | 1 | 0.998 | 0.009658 | 3 |
| AC022210. | 4.96E-07 | 0.42358534 | 0.857 | 0.77 | 0.010229 | 3 |
| RPL15P3 | 5.50E-07 | 0.42908895 | 1 | 0.97 | 0.011361 | 3 |
| TXN | 6.51E-07 | 0.42698599 | 1 | 0.97 | 0.013426 | 3 |
| RPL10L | 6.52E-07 | 0.42160192 | 0.738 | 0.525 | 0.01345 | 3 |
| RPS23 | 6.97E-07 | 0.28512675 | 1 | 1 | 0.014384 | 3 |
| ATP5EP2 | 7.17E-07 | 0.3700044 | 0.976 | 0.954 | 0.014791 | 3 |
| MTRNR2L1 | 8.69E-07 | 0.57752393 | 1 | 0.996 | 0.017934 | 3 |
| RPL34 | 9.75E-07 | 0.2935588 | 1 | 0.998 | 0.020116 | 3 |
| CTB-33G10 | 9.76E-07 | 0.36230958 | 0.976 | 0.954 | 0.020146 | 3 |
| RP11-391L3 | 1.13E-06 | 0.42317471 | 1 | 0.95 | 0.023237 | 3 |
| RPS16 | 1.24E-06 | 0.3363435 | 1 | 1 | 0.025534 | 3 |
| RPL37 | 1.40E-06 | 0.2299675 | 1 | 0.998 | 0.028893 | 3 |
| NHP2 | 1.74E-06 | 0.40892446 | 0.976 | 0.932 | 0.035978 | 3 |
| RP11-122G | 1.74E-06 | 0.53417084 | 0.81 | 0.599 | 0.035985 | 3 |
| RPL36AP48 | 1.93E-06 | 0.45326062 | 0.619 | 0.361 | 0.03986 | 3 |
| RPS20 | 2.03E-06 | 0.36495203 | 1 | 0.976 | 0.041815 | 3 |

| | | | | | | |
|---|---|---|---|---|---|---|
| SEC61G | 2.44E-06 | 0.55103781 | 0.786 | 0.756 | 0.050421 | 3 |
| RPL12P16 | 2.68E-06 | 0.31747657 | 1 | 0.976 | 0.055298 | 3 |
| PRDX6 | 2.72E-06 | 0.42740805 | 0.952 | 0.93 | 0.056079 | 3 |
| PHB | 2.84E-06 | 0.38500303 | 0.929 | 0.87 | 0.058582 | 3 |
| SUMO1 | 2.86E-06 | 0.41718764 | 0.929 | 0.848 | 0.059007 | 3 |
| PPIAP19 | 3.04E-06 | 0.44926786 | 0.929 | 0.758 | 0.062746 | 3 |
| RPS15AP24 | 3.20E-06 | 0.42389061 | 0.762 | 0.577 | 0.065961 | 3 |
| RP11-372E | 3.29E-06 | 0.44425074 | 0.881 | 0.737 | 0.067807 | 3 |
| RPS3A | 3.74E-06 | 0.35351312 | 1 | 0.958 | 0.077128 | 3 |
| UQCRH | 3.88E-06 | 0.34977556 | 1 | 0.992 | 0.080089 | 3 |
| UBBP4 | 3.98E-06 | 0.4429114 | 0.952 | 0.832 | 0.082105 | 3 |
| EDF1 | 4.91E-06 | 0.44339245 | 0.976 | 0.888 | 0.101326 | 3 |
| RP11-25I15 | 5.76E-06 | 0.38764105 | 0.952 | 0.926 | 0.118844 | 3 |
| RPS17P5 | 6.40E-06 | 0.39438763 | 0.762 | 0.553 | 0.132056 | 3 |
| BTF3 | 6.51E-06 | 0.32319872 | 1 | 0.992 | 0.134332 | 3 |
| CTC-451P1 | 6.72E-06 | 0.40075107 | 0.81 | 0.659 | 0.138623 | 3 |
| RPS23P8 | 7.36E-06 | 0.28141254 | 1 | 1 | 0.151873 | 3 |
| RPL5P22 | 8.77E-06 | 0.34487693 | 0.905 | 0.717 | 0.180915 | 3 |
| CAPNS1 | 9.06E-06 | 0.3857014 | 0.929 | 0.886 | 0.187027 | 3 |
| RPS15AP11 | 9.30E-06 | 0.43653929 | 0.786 | 0.619 | 0.191864 | 3 |
| RPS3AP6 | 9.43E-06 | 0.3264564 | 1 | 0.958 | 0.19455 | 3 |
| RPSAP15 | 9.66E-06 | 0.45073216 | 0.857 | 0.772 | 0.199451 | 3 |
| RPL9P28 | 9.96E-06 | 0.40591293 | 0.667 | 0.473 | 0.205631 | 3 |
| EEF1G | 1.07E-05 | 0.39264547 | 0.952 | 0.908 | 0.220181 | 3 |
| AURKAIP1 | 1.12E-05 | 0.42374239 | 0.714 | 0.519 | 0.231061 | 3 |
| AC016700. | 1.22E-05 | 0.42554822 | 0.81 | 0.725 | 0.250914 | 3 |
| RPS5 | 1.22E-05 | 0.25970416 | 1 | 0.998 | 0.252366 | 3 |
| RPL7P1 | 1.24E-05 | 0.30063178 | 1 | 0.994 | 0.255342 | 3 |
| FTH1P8 | 1.25E-05 | 0.25727535 | 0.976 | 0.998 | 0.258959 | 3 |
| PPIAP9 | 1.32E-05 | 0.41149103 | 0.929 | 0.758 | 0.271479 | 3 |
| ATG10 | 1.38E-05 | 0.248955 | 1 | 1 | 0.285679 | 3 |
| RPS15AP1 | 1.85E-05 | 0.41677638 | 0.857 | 0.749 | 0.382273 | 3 |
| RPL23P8 | 1.86E-05 | 0.36016098 | 0.929 | 0.916 | 0.383077 | 3 |
| RPL10P1 | 1.86E-05 | 0.40620176 | 0.881 | 0.826 | 0.383979 | 3 |
| RPS9 | 1.86E-05 | 0.30626502 | 1 | 0.996 | 0.384295 | 3 |
| EIF3E | 2.07E-05 | 0.31768748 | 1 | 0.984 | 0.428047 | 3 |
| EIF5AL1 | 2.22E-05 | 0.4115251 | 0.762 | 0.655 | 0.458316 | 3 |
| C14orf2 | 2.28E-05 | 0.30654213 | 0.952 | 0.934 | 0.469963 | 3 |
| RPUSD3 | 2.38E-05 | 0.52955677 | 0.619 | 0.413 | 0.491459 | 3 |
| RPS13P2 | 2.47E-05 | 0.25730171 | 1 | 0.988 | 0.510173 | 3 |
| ETFB | 2.48E-05 | 0.42536461 | 0.881 | 0.719 | 0.510816 | 3 |
| RPL13A | 2.55E-05 | 0.2492351 | 1 | 1 | 0.526637 | 3 |
| RPL17-C18 | 2.61E-05 | 0.35019642 | 0.952 | 0.916 | 0.539695 | 3 |
| MTRNR2L3 | 2.64E-05 | 0.46280617 | 0.833 | 0.772 | 0.5454 | 3 |
| PFN1 | 2.82E-05 | 0.29297107 | 1 | 0.996 | 0.583024 | 3 |
| NDUFC2-K | 2.86E-05 | 0.38392589 | 0.881 | 0.784 | 0.589747 | 3 |
| ATP5L | 2.92E-05 | 0.31917496 | 0.976 | 0.966 | 0.602862 | 3 |

| | | | | | | |
|---|---|---|---|---|---|---|
| SH3BGRL3 | 2.95E-05 | 0.52609063 | 0.786 | 0.739 | 0.609586 | 3 |
| RPL41P2 | 2.96E-05 | 0.23227944 | 1 | 0.998 | 0.61126 | 3 |
| RPL7AP30 | 3.08E-05 | 0.35691808 | 0.786 | 0.613 | 0.635749 | 3 |
| AC004692. | 3.12E-05 | 0.33300152 | 0.762 | 0.583 | 0.64462 | 3 |
| RPS11 | 3.30E-05 | 0.21664282 | 1 | 0.996 | 0.680759 | 3 |
| S100A11 | 3.35E-05 | 0.37059282 | 1 | 0.978 | 0.69145 | 3 |
| ATP5E | 3.35E-05 | 0.22462119 | 1 | 1 | 0.691502 | 3 |
| EEF1A1P5 | 3.53E-05 | 0.25573968 | 1 | 0.994 | 0.729564 | 3 |
| RPS24 | 3.75E-05 | 0.26070428 | 0.976 | 0.998 | 0.774765 | 3 |
| SLC25A5P6 | 3.98E-05 | 0.3437761 | 0.81 | 0.713 | 0.821698 | 3 |
| RP11-3P17 | 4.05E-05 | 0.39417305 | 0.667 | 0.465 | 0.836042 | 3 |
| RPSAP17 | 4.52E-05 | 0.48465361 | 0.881 | 0.846 | 0.933553 | 3 |
| RPL37AP1 | 4.65E-05 | 0.43656172 | 0.762 | 0.695 | 0.959375 | 3 |
| RPS15AP5 | 5.23E-05 | 0.35753881 | 0.69 | 0.529 | 1 | 3 |
| RPL24P4 | 5.31E-05 | 0.31863668 | 1 | 0.934 | 1 | 3 |
| MAPK3 | 5.32E-05 | 0.33422594 | 0.5 | 0.265 | 1 | 3 |
| CH507-42P | 5.92E-05 | 0.38804112 | 0.595 | 0.387 | 1 | 3 |
| COX8A | 5.93E-05 | 0.36422113 | 0.929 | 0.926 | 1 | 3 |
| AC090804. | 6.72E-05 | 0.33386944 | 0.595 | 0.365 | 1 | 3 |
| FAU | 6.79E-05 | 0.26752885 | 1 | 0.95 | 1 | 3 |
| TSTD1 | 6.94E-05 | 0.45227335 | 0.714 | 0.573 | 1 | 3 |
| AC090602. | 6.99E-05 | 0.38202794 | 0.976 | 0.94 | 1 | 3 |
| RPL12 | 8.27E-05 | 0.21740342 | 1 | 1 | 1 | 3 |
| NDUFB2 | 8.35E-05 | 0.33124332 | 0.881 | 0.884 | 1 | 3 |
| SEC61B | 8.36E-05 | 0.27141534 | 0.976 | 0.974 | 1 | 3 |
| WDR83OS | 8.37E-05 | 0.36635342 | 0.833 | 0.81 | 1 | 3 |
| HMGN1 | 9.05E-05 | 0.29693479 | 0.976 | 0.972 | 1 | 3 |
| RP3-417G1 | 9.55E-05 | 0.32542823 | 0.976 | 0.95 | 1 | 3 |
| RP11-371A | 0.000101 | 0.35349749 | 0.881 | 0.782 | 1 | 3 |
| RP11-152F | 0.000101 | 0.38900701 | 0.929 | 0.782 | 1 | 3 |
| ATP5D | 0.000103 | 0.40530836 | 0.786 | 0.693 | 1 | 3 |
| MMACHC | 0.000105 | 0.3296631 | 0.976 | 0.946 | 1 | 3 |
| COX5A | 0.000108 | 0.3509339 | 0.952 | 0.942 | 1 | 3 |
| UQCR10 | 0.000109 | 0.39566212 | 0.976 | 0.796 | 1 | 3 |
| RP11-435P | 0.000113 | 0.28743672 | 0.524 | 0.305 | 1 | 3 |
| RP5-1106E | 0.000114 | 0.3264248 | 0.714 | 0.527 | 1 | 3 |
| APRT | 0.000118 | 0.32685739 | 0.929 | 0.848 | 1 | 3 |
| LINC01641 | 0.000121 | 0.32947419 | 0.976 | 0.97 | 1 | 3 |
| RPL22 | 0.000122 | 0.3073972 | 1 | 0.962 | 1 | 3 |
| GNAS | 0.000124 | 0.30169089 | 1 | 1 | 1 | 3 |
| CTC-398G3 | 0.000124 | 0.28933889 | 0.738 | 0.559 | 1 | 3 |
| AP000620. | 0.000124 | 0.32118415 | 0.69 | 0.501 | 1 | 3 |
| CH507-513 | 0.000136 | 0.05864858 | 0.69 | 0.497 | 1 | 3 |
| FTH1P2 | 0.000137 | 0.23159657 | 1 | 0.994 | 1 | 3 |
| RPS27 | 0.000143 | 0.31939822 | 0.952 | 0.948 | 1 | 3 |
| PPIAL4F | 0.000143 | 0.37148888 | 0.595 | 0.387 | 1 | 3 |
| RPL39L | 0.000144 | 0.3081864 | 0.976 | 0.94 | 1 | 3 |

| | | | | | | |
|---|---|---|---|---|---|---|
| RPL13 | 0.000149 | 0.23019845 | 1 | 0.998 | 1 | 3 |
| PRDX2 | 0.000156 | 0.3424218 | 0.833 | 0.812 | 1 | 3 |
| NQO1 | 0.000159 | 0.34063737 | 0.976 | 0.954 | 1 | 3 |
| HINT1 | 0.000165 | 0.25129543 | 1 | 0.97 | 1 | 3 |
| RP11-79L9. | 0.000173 | 0.39635646 | 0.714 | 0.563 | 1 | 3 |
| LRRC75A | 0.000175 | 0.38709051 | 0.786 | 0.667 | 1 | 3 |
| PCBP2 | 0.000181 | 0.43134418 | 0.69 | 0.547 | 1 | 3 |
| RPS3 | 0.000189 | 0.17246727 | 1 | 0.998 | 1 | 3 |
| RPL23 | 0.000191 | 0.23956271 | 1 | 0.998 | 1 | 3 |
| FTH1 | 0.000197 | 0.17597635 | 1 | 1 | 1 | 3 |
| AP000936. | 0.000203 | 0.33855562 | 0.905 | 0.806 | 1 | 3 |
| RPS3AP43 | 0.000205 | 0.34989657 | 0.619 | 0.431 | 1 | 3 |
| RPS15 | 0.000209 | 0.25302065 | 0.929 | 0.912 | 1 | 3 |
| RPL29P12 | 0.00021 | 0.38619163 | 0.524 | 0.335 | 1 | 3 |
| ATP5C1 | 0.00022 | 0.31246815 | 0.905 | 0.902 | 1 | 3 |
| NME1-NMI | 0.000222 | 0.24502609 | 1 | 0.996 | 1 | 3 |
| RPL36A | 0.00024 | 0.23255202 | 1 | 1 | 1 | 3 |
| SELENOW | 0.000244 | 0.32140228 | 0.952 | 0.938 | 1 | 3 |
| RP11-69L1( | 0.00025 | 0.23965415 | 1 | 0.992 | 1 | 3 |
| RPS27P15 | 0.000253 | 0.38438141 | 0.643 | 0.507 | 1 | 3 |
| BTF3P1 | 0.000255 | 0.24459524 | 1 | 0.982 | 1 | 3 |
| RPS7P10 | 0.000259 | 0.28270012 | 0.976 | 0.91 | 1 | 3 |
| RPS21 | 0.000265 | 0.20533785 | 1 | 0.992 | 1 | 3 |
| BTF3P9 | 0.000281 | 0.33712498 | 0.952 | 0.892 | 1 | 3 |
| RP11-613M | 0.000286 | 0.36965527 | 0.476 | 0.283 | 1 | 3 |
| RPL18 | 0.000288 | 0.22811196 | 1 | 0.996 | 1 | 3 |
| EIF1P3 | 0.000306 | 0.30762908 | 0.929 | 0.842 | 1 | 3 |
| ATP5J2 | 0.000318 | 0.31228956 | 1 | 0.932 | 1 | 3 |
| COMMD6 | 0.000322 | 0.32247189 | 0.786 | 0.657 | 1 | 3 |
| RPL7AP11 | 0.000357 | 0.31581902 | 0.571 | 0.417 | 1 | 3 |
| RP11-466H | 0.000364 | 0.22727947 | 0.976 | 0.982 | 1 | 3 |
| EEF1A1P12 | 0.000366 | 0.33255768 | 0.905 | 0.834 | 1 | 3 |
| CH507-513 | 0.000379 | 0.11944894 | 0.667 | 0.515 | 1 | 3 |
| PPIAL4G | 0.0004 | 0.31384846 | 0.905 | 0.852 | 1 | 3 |
| KRT8 | 0.000458 | 0.36969117 | 0.905 | 0.838 | 1 | 3 |
| RPL4P4 | 0.000472 | 0.29527894 | 1 | 0.97 | 1 | 3 |
| RP11-360D | 0.000474 | 0.3711058 | 0.643 | 0.543 | 1 | 3 |
| RPL34P18 | 0.000485 | 0.31626867 | 0.905 | 0.868 | 1 | 3 |
| RPSAP46 | 0.000498 | 0.34407529 | 0.452 | 0.267 | 1 | 3 |
| RPL12P17 | 0.0005 | 0.34061258 | 0.81 | 0.727 | 1 | 3 |
| CHURC1-FN | 0.000503 | 0.36639811 | 0.738 | 0.553 | 1 | 3 |
| BTF3P4 | 0.000505 | 0.2638611 | 1 | 0.984 | 1 | 3 |
| MZT2A | 0.000512 | 0.27544926 | 0.881 | 0.886 | 1 | 3 |
| CCDC73 | 0.000513 | 0.18039384 | 0.262 | 0.098 | 1 | 3 |
| EIF1 | 0.000519 | 0.23744801 | 1 | 1 | 1 | 3 |
| AC010733. | 0.000552 | 0.29781251 | 0.929 | 0.81 | 1 | 3 |
| RPL23AP74 | 0.000564 | 0.29188808 | 0.738 | 0.633 | 1 | 3 |

| | | | | | | |
|---|---|---|---|---|---|---|
| RPS4XP17 | 0.000565 | 0.33679476 | 0.524 | 0.353 | 1 | 3 |
| PPIAL4A | 0.000567 | 0.31233879 | 0.714 | 0.557 | 1 | 3 |
| CTB-147C2 | 0.00057 | 0.32213156 | 0.571 | 0.391 | 1 | 3 |
| RP11-571F | 0.000613 | 0.34117368 | 0.524 | 0.347 | 1 | 3 |
| RPL23AP42 | 0.000622 | 0.23694125 | 1 | 0.984 | 1 | 3 |
| ETFA | 0.000648 | 0.39902089 | 0.857 | 0.816 | 1 | 3 |
| PPIAP29 | 0.00065 | 0.26596994 | 0.929 | 0.878 | 1 | 3 |
| RPL21P28 | 0.000652 | 0.38387105 | 0.619 | 0.495 | 1 | 3 |
| GNGT1 | 0.000653 | 0.37958096 | 0.571 | 0.417 | 1 | 3 |
| RPS2P28 | 0.000656 | 0.31948994 | 0.595 | 0.423 | 1 | 3 |
| ZNHIT1 | 0.000672 | 0.3898734 | 0.81 | 0.741 | 1 | 3 |
| PSMB4 | 0.000688 | 0.26994777 | 0.976 | 0.924 | 1 | 3 |
| BTF3P7 | 0.000688 | 0.26431074 | 1 | 0.99 | 1 | 3 |
| RPL4 | 0.000723 | 0.23026189 | 1 | 1 | 1 | 3 |
| EIF5A | 0.000728 | 0.33654979 | 0.714 | 0.541 | 1 | 3 |
| LAMTOR2 | 0.000728 | 0.32443194 | 0.643 | 0.507 | 1 | 3 |
| MTRNR2L8 | 0.000729 | 0.43668683 | 1 | 0.99 | 1 | 3 |
| NDUFC2 | 0.000743 | 0.30418555 | 0.905 | 0.81 | 1 | 3 |
| RPL10AP6 | 0.000771 | 0.28457174 | 0.881 | 0.862 | 1 | 3 |
| SH3YL1 | 0.00079 | 0.27896411 | 0.476 | 0.251 | 1 | 3 |
| CHMP2A | 0.000799 | 0.46395346 | 0.643 | 0.543 | 1 | 3 |
| AC098614. | 0.000816 | 0.3603933 | 0.881 | 0.85 | 1 | 3 |
| JTB | 0.000847 | 0.25795555 | 0.929 | 0.952 | 1 | 3 |
| RP11-553P | 0.000855 | 0.22303529 | 0.976 | 0.922 | 1 | 3 |
| ARPC3 | 0.000855 | 0.27438679 | 0.976 | 0.924 | 1 | 3 |
| RP11-452G | 0.000876 | 0.28811973 | 0.738 | 0.567 | 1 | 3 |
| CCT3 | 0.000882 | 0.30359961 | 0.976 | 0.928 | 1 | 3 |
| RPSAP12 | 0.000919 | 0.30343706 | 0.905 | 0.836 | 1 | 3 |
| CTD-2270N | 0.000922 | 0.30011653 | 0.738 | 0.569 | 1 | 3 |
| FTH1P20 | 0.000948 | 0.21707564 | 1 | 0.998 | 1 | 3 |
| ATPIF1 | 0.000991 | 0.35177512 | 0.881 | 0.745 | 1 | 3 |
| S100A13 | 0.001008 | 0.36719474 | 0.738 | 0.669 | 1 | 3 |
| PTOV1 | 0.001052 | 0.26288526 | 0.429 | 0.24 | 1 | 3 |
| COX4I1 | 0.001057 | 0.26117659 | 1 | 0.952 | 1 | 3 |
| RPL21P134 | 0.001057 | 0.26739759 | 0.452 | 0.257 | 1 | 3 |
| C19orf53 | 0.001082 | 0.31471167 | 0.714 | 0.623 | 1 | 3 |
| RP11-489M | 0.001121 | 0.32355782 | 0.548 | 0.379 | 1 | 3 |
| RPL27A | 0.001145 | 0.21438646 | 1 | 0.994 | 1 | 3 |
| RPL18AP16 | 0.001187 | 0.25359583 | 0.5 | 0.317 | 1 | 3 |
| AC010468. | 0.001193 | 0.35706056 | 0.738 | 0.605 | 1 | 3 |
| CKB | 0.001233 | 0.29762215 | 0.333 | 0.162 | 1 | 3 |
| CTC-448F2. | 0.001238 | 0.20993701 | 0.262 | 0.106 | 1 | 3 |
| RPL7AP66 | 0.001257 | 0.27503782 | 0.952 | 0.898 | 1 | 3 |
| AC140076. | 0.001295 | 0.27245019 | 1 | 0.96 | 1 | 3 |
| AC020550. | 0.001312 | 0.29031674 | 0.714 | 0.601 | 1 | 3 |
| RP11-829H | 0.001365 | 0.29263883 | 0.929 | 0.866 | 1 | 3 |
| CTC-550B1 | 0.001373 | 0.25523076 | 0.571 | 0.411 | 1 | 3 |

| | | | | | | |
|---|---|---|---|---|---|---|
| AC009245. | 0.001406 | 0.2462328 | 1 | 0.98 | 1 | 3 |
| EEF1D | 0.001424 | 0.24628763 | 0.952 | 0.914 | 1 | 3 |
| C1orf122 | 0.001483 | 0.28234848 | 0.81 | 0.697 | 1 | 3 |
| ATP5I | 0.001491 | 0.31249051 | 0.81 | 0.752 | 1 | 3 |
| RPS18P12 | 0.001518 | 0.26894629 | 0.762 | 0.653 | 1 | 3 |
| RP11-144C | 0.001533 | 0.27546332 | 1 | 0.986 | 1 | 3 |
| COX5B | 0.001569 | 0.34335375 | 0.881 | 0.85 | 1 | 3 |
| RP11-183G | 0.001573 | 0.31109834 | 0.69 | 0.627 | 1 | 3 |
| TMSB4X | 0.001639 | 0.32548001 | 1 | 0.996 | 1 | 3 |
| NBEAL1 | 0.001679 | 0.20642216 | 1 | 0.99 | 1 | 3 |
| RPL24 | 0.001691 | 0.22628548 | 1 | 0.99 | 1 | 3 |
| POLR2L | 0.001699 | 0.37584719 | 0.81 | 0.864 | 1 | 3 |
| RP13-258O | 0.0017 | 0.231448 | 0.952 | 0.926 | 1 | 3 |
| COX6C | 0.001702 | 0.23190958 | 1 | 0.958 | 1 | 3 |
| RPL35P1 | 0.001706 | 0.32365876 | 0.643 | 0.539 | 1 | 3 |
| CH507-513 | 0.001707 | 0.16068013 | 0.619 | 0.491 | 1 | 3 |
| RP11-448G | 0.001787 | 0.19662615 | 0.333 | 0.17 | 1 | 3 |
| CHCHD2 | 0.001804 | 0.21334513 | 1 | 0.992 | 1 | 3 |
| OST4 | 0.001819 | 0.28251567 | 1 | 0.952 | 1 | 3 |
| RPL21P120 | 0.001882 | 0.26151039 | 0.405 | 0.232 | 1 | 3 |
| FTH1P12 | 0.001896 | 0.25185849 | 0.976 | 0.952 | 1 | 3 |
| FTH1P7 | 0.001917 | 0.18668176 | 1 | 0.998 | 1 | 3 |
| RPA3 | 0.001921 | 0.27268933 | 0.524 | 0.353 | 1 | 3 |
| BEX3 | 0.001975 | 0.32767466 | 0.81 | 0.707 | 1 | 3 |
| PCBD1 | 0.002086 | 0.28994109 | 0.786 | 0.737 | 1 | 3 |
| AC000089. | 0.002089 | 0.29807654 | 0.929 | 0.886 | 1 | 3 |
| RP5-827C2 | 0.002095 | 0.36354539 | 0.619 | 0.515 | 1 | 3 |
| RP11-534L. | 0.002111 | 0.22252177 | 0.476 | 0.305 | 1 | 3 |
| TXNP4 | 0.002112 | 0.29551669 | 0.524 | 0.375 | 1 | 3 |
| EEF1A1P11 | 0.002151 | 0.23838918 | 1 | 0.986 | 1 | 3 |
| PRDX1P1 | 0.002197 | 0.33085391 | 0.81 | 0.735 | 1 | 3 |
| RPL5P18 | 0.002211 | 0.27564695 | 0.595 | 0.441 | 1 | 3 |
| NDUFA12 | 0.002222 | 0.22687349 | 0.952 | 0.814 | 1 | 3 |
| RP1-249H1 | 0.002226 | 0.29885333 | 0.905 | 0.804 | 1 | 3 |
| RPS3AP33 | 0.002238 | 0.23048763 | 0.405 | 0.232 | 1 | 3 |
| SLC25A5 | 0.002276 | 0.28239658 | 0.952 | 0.974 | 1 | 3 |
| RPS2P6 | 0.002284 | 0.21110303 | 0.69 | 0.499 | 1 | 3 |
| RPSAP45 | 0.002285 | 0.25169409 | 0.595 | 0.467 | 1 | 3 |
| PPP4C | 0.002318 | 0.29923207 | 0.405 | 0.246 | 1 | 3 |
| SLC25A39 | 0.002421 | 0.27890939 | 0.929 | 0.916 | 1 | 3 |
| RPL23AP65 | 0.002464 | 0.19602784 | 0.976 | 0.952 | 1 | 3 |
| RPS24P8 | 0.002516 | 0.24397399 | 0.881 | 0.918 | 1 | 3 |
| LDOC1 | 0.002528 | 0.4367611 | 0.619 | 0.485 | 1 | 3 |
| NHP2P1 | 0.002562 | 0.25446326 | 0.929 | 0.854 | 1 | 3 |
| RPS7 | 0.002593 | 0.18247581 | 1 | 0.992 | 1 | 3 |
| NDUFB3 | 0.002606 | 0.2621298 | 0.81 | 0.683 | 1 | 3 |
| RP11-16L9. | 0.002623 | 0.21952298 | 0.429 | 0.253 | 1 | 3 |

| | | | | | | |
|---|---|---|---|---|---|---|
| NDUFA2 | 0.002668 | 0.2216702 | 0.762 | 0.623 | 1 | 3 |
| RP11-411B | 0.002861 | 0.26387513 | 0.714 | 0.607 | 1 | 3 |
| EID1 | 0.002872 | 0.31221825 | 0.738 | 0.645 | 1 | 3 |
| GNG5 | 0.002912 | 0.26004177 | 0.833 | 0.75 | 1 | 3 |
| UQCRQ | 0.002914 | 0.30776516 | 0.857 | 0.822 | 1 | 3 |
| ISYNA1 | 0.003028 | 0.2527729 | 0.429 | 0.269 | 1 | 3 |
| NDUFS8 | 0.003084 | 0.27454418 | 0.81 | 0.697 | 1 | 3 |
| OAZ1 | 0.003148 | 0.19394578 | 0.952 | 0.98 | 1 | 3 |
| AC009961. | 0.003241 | 0.23905573 | 0.5 | 0.355 | 1 | 3 |
| AP1S1 | 0.003265 | 0.22229651 | 0.786 | 0.675 | 1 | 3 |
| UBC | 0.003266 | 0.27119156 | 0.976 | 0.882 | 1 | 3 |
| CTA-242H1 | 0.003293 | 0.22597535 | 0.714 | 0.589 | 1 | 3 |
| DUSP23 | 0.003307 | 0.27805465 | 0.31 | 0.162 | 1 | 3 |
| RP1-40G4P | 0.003335 | 0.26236348 | 0.905 | 0.896 | 1 | 3 |
| C12orf57 | 0.003339 | 0.29196523 | 0.667 | 0.535 | 1 | 3 |
| NUCKS1 | 0.003579 | 0.29255843 | 0.976 | 0.956 | 1 | 3 |
| RPS7P4 | 0.003581 | 0.32986594 | 0.738 | 0.756 | 1 | 3 |
| RPSAP9 | 0.003586 | 0.35075992 | 0.571 | 0.489 | 1 | 3 |
| SUMO2 | 0.003681 | 0.28759299 | 0.905 | 0.886 | 1 | 3 |
| RPL35A | 0.003829 | 0.16046283 | 1 | 0.998 | 1 | 3 |
| MTX1 | 0.003885 | 0.32952078 | 0.333 | 0.186 | 1 | 3 |
| SC22CB-1E | 0.003928 | 0.29527294 | 0.738 | 0.609 | 1 | 3 |
| NDUFC1 | 0.003929 | 0.2633119 | 0.952 | 0.818 | 1 | 3 |
| DNPH1 | 0.003976 | 0.25198424 | 0.595 | 0.443 | 1 | 3 |
| RPS19 | 0.004007 | 0.13408473 | 1 | 1 | 1 | 3 |
| RPS10-NUD | 0.004013 | 0.24922956 | 1 | 0.974 | 1 | 3 |
| EEF1A2 | 0.004036 | 0.36647041 | 0.548 | 0.431 | 1 | 3 |
| FSCN3 | 0.004045 | 0.33022257 | 0.595 | 0.453 | 1 | 3 |
| HSPB1P1 | 0.004086 | 0.1520971 | 1 | 0.996 | 1 | 3 |
| MRPS21 | 0.004221 | 0.1899941 | 0.905 | 0.878 | 1 | 3 |
| PSMB3 | 0.00426 | 0.24651942 | 0.429 | 0.261 | 1 | 3 |
| RPL12P35 | 0.004284 | 0.25442747 | 0.714 | 0.641 | 1 | 3 |
| RPS3AP47 | 0.004346 | 0.32239047 | 0.643 | 0.553 | 1 | 3 |
| CSNK2B | 0.004348 | 0.30495188 | 0.81 | 0.812 | 1 | 3 |
| PFDN4 | 0.004457 | 0.2122132 | 0.976 | 0.96 | 1 | 3 |
| METRN | 0.004505 | 0.2980152 | 0.595 | 0.475 | 1 | 3 |
| BEST1 | 0.004559 | 0.13370955 | 1 | 1 | 1 | 3 |
| NACA | 0.004618 | 0.23780935 | 0.905 | 0.93 | 1 | 3 |
| NDUFS5 | 0.004759 | 0.23272293 | 1 | 0.92 | 1 | 3 |
| AC097523. | 0.004784 | 0.2935578 | 0.619 | 0.495 | 1 | 3 |
| RPL3P4 | 0.004852 | 0.20946518 | 0.857 | 0.934 | 1 | 3 |
| AC008280. | 0.004853 | 0.24856472 | 0.333 | 0.186 | 1 | 3 |
| NACA2 | 0.004877 | 0.29497266 | 0.667 | 0.637 | 1 | 3 |
| RPL12P1 | 0.004987 | 0.21430279 | 0.714 | 0.657 | 1 | 3 |
| CHURC1 | 0.005007 | 0.32329645 | 0.833 | 0.743 | 1 | 3 |
| COPZ1 | 0.005044 | 0.24924153 | 0.69 | 0.553 | 1 | 3 |
| CTD-2184D | 0.005051 | 0.2333667 | 0.929 | 0.934 | 1 | 3 |

| | | | | | | |
|---|---|---|---|---|---|---|
| PPP2R1A | 0.005142 | 0.20743914 | 0.714 | 0.621 | 1 | 3 |
| RPL15P2 | 0.005166 | 0.24145136 | 0.881 | 0.816 | 1 | 3 |
| RPSAP49 | 0.005331 | 0.17157722 | 0.381 | 0.212 | 1 | 3 |
| FTH1P10 | 0.00556 | 0.15754671 | 1 | 0.998 | 1 | 3 |
| RPSAP19 | 0.005584 | 0.21994417 | 0.619 | 0.477 | 1 | 3 |
| RAB25 | 0.005596 | 0.31340479 | 0.762 | 0.715 | 1 | 3 |
| SUMO3 | 0.005689 | 0.3273778 | 0.929 | 0.902 | 1 | 3 |
| CHCHD1 | 0.005693 | 0.38422499 | 0.738 | 0.643 | 1 | 3 |
| RPL7P32 | 0.005784 | 0.23231993 | 0.952 | 0.93 | 1 | 3 |
| CH17-385C | 0.005837 | 0.22719647 | 0.524 | 0.369 | 1 | 3 |
| CIB1 | 0.005876 | 0.23096104 | 0.452 | 0.293 | 1 | 3 |
| MT2A | 0.005908 | 0.23920787 | 0.5 | 0.317 | 1 | 3 |
| NDUFB1 | 0.005936 | 0.26444485 | 0.833 | 0.735 | 1 | 3 |
| AC092610. | 0.006004 | 0.17738301 | 0.714 | 0.565 | 1 | 3 |
| UQCRC2 | 0.006132 | 0.38787763 | 0.667 | 0.601 | 1 | 3 |
| RPS3AP36 | 0.006207 | 0.23160433 | 0.476 | 0.343 | 1 | 3 |
| RPL21P16 | 0.006298 | 0.27631969 | 0.595 | 0.475 | 1 | 3 |
| RP11-641D | 0.006431 | 0.23111442 | 0.929 | 0.884 | 1 | 3 |
| FTH1P5 | 0.006598 | 0.19776492 | 0.929 | 0.954 | 1 | 3 |
| RP11-425L1 | 0.006626 | 0.1566463 | 0.976 | 0.938 | 1 | 3 |
| RPL10P5 | 0.006694 | 0.3063715 | 0.571 | 0.467 | 1 | 3 |
| SLC25A3 | 0.006776 | 0.16974314 | 1 | 0.976 | 1 | 3 |
| AC007969. | 0.006953 | 0.32358281 | 0.762 | 0.76 | 1 | 3 |
| MCTS1 | 0.007123 | 0.31758442 | 0.595 | 0.493 | 1 | 3 |
| C1QBP | 0.007176 | 0.2394193 | 0.786 | 0.681 | 1 | 3 |
| GSTO1 | 0.007335 | 0.24703878 | 0.667 | 0.537 | 1 | 3 |
| SOD1 | 0.007358 | 0.21742599 | 0.929 | 0.918 | 1 | 3 |
| SMIM14 | 0.007408 | 0.26567832 | 0.643 | 0.513 | 1 | 3 |
| RP11-314A | 0.007415 | 0.21178891 | 0.714 | 0.607 | 1 | 3 |
| AK3 | 0.007531 | 0.28558438 | 0.667 | 0.591 | 1 | 3 |
| RPL37A | 0.007579 | 0.18269997 | 1 | 1 | 1 | 3 |
| EIF3F | 0.007746 | 0.31609511 | 0.714 | 0.629 | 1 | 3 |
| RPL12P4 | 0.007849 | 0.21292901 | 0.976 | 0.976 | 1 | 3 |
| RPL4P5 | 0.007939 | 0.21804251 | 0.952 | 0.94 | 1 | 3 |
| RP11-507E1 | 0.007954 | 0.24873386 | 0.952 | 0.954 | 1 | 3 |
| C1orf198 | 0.008133 | 0.21517666 | 0.262 | 0.134 | 1 | 3 |
| RPL7AP50 | 0.008204 | 0.29572155 | 0.524 | 0.429 | 1 | 3 |
| EEF1A1P4 | 0.00836 | 0.31915986 | 0.69 | 0.645 | 1 | 3 |
| RP11-50D9 | 0.008373 | 0.30665346 | 0.548 | 0.439 | 1 | 3 |
| AP2S1 | 0.008576 | 0.24407877 | 0.81 | 0.703 | 1 | 3 |
| MRPL28 | 0.008866 | 0.23256576 | 0.595 | 0.451 | 1 | 3 |
| PSMD4 | 0.008933 | 0.22177258 | 0.81 | 0.745 | 1 | 3 |
| PABPC1 | 0.00895 | 0.20545922 | 1 | 1 | 1 | 3 |
| COMT | 0.009137 | 0.22269557 | 0.833 | 0.778 | 1 | 3 |
| RP1-273G1 | 0.009309 | 0.18362558 | 0.5 | 0.361 | 1 | 3 |
| H2BFS | 0.009439 | 0.33902966 | 0.452 | 0.335 | 1 | 3 |
| ATP5F1 | 0.009469 | 0.23693612 | 0.976 | 0.952 | 1 | 3 |

| | | | | | | |
|---|---|---|---|---|---|---|
| ENO1 | 0.009831 | 0.11577874 | 1 | 0.986 | 1 | 3 |
| RRM2 | 1.69E-48 | 1.14528587 | 0.719 | 0.035 | 3.49E-44 | 4 |
| MKI67 | 8.81E-46 | 0.62437227 | 0.469 | 0.006 | 1.82E-41 | 4 |
| PBK | 1.15E-45 | 0.59184376 | 0.469 | 0.006 | 2.38E-41 | 4 |
| MCM10 | 1.19E-38 | 0.56055752 | 0.406 | 0.006 | 2.45E-34 | 4 |
| CDK1 | 9.53E-38 | 0.43020474 | 0.375 | 0.004 | 1.97E-33 | 4 |
| DLGAP5 | 2.60E-37 | 0.47242372 | 0.344 | 0.002 | 5.36E-33 | 4 |
| MYBL2 | 6.39E-37 | 0.71691922 | 0.594 | 0.031 | 1.32E-32 | 4 |
| TK1 | 7.57E-37 | 0.67952265 | 0.469 | 0.014 | 1.56E-32 | 4 |
| CDCA5 | 8.50E-34 | 0.52930993 | 0.469 | 0.018 | 1.75E-29 | 4 |
| KIF4A | 9.84E-34 | 0.39602793 | 0.312 | 0.002 | 2.03E-29 | 4 |
| PCLAF | 1.16E-33 | 1.86362783 | 0.938 | 0.157 | 2.40E-29 | 4 |
| NCAPH | 1.17E-33 | 0.35627123 | 0.312 | 0.002 | 2.42E-29 | 4 |
| HMGB2 | 8.16E-32 | 1.12638457 | 0.625 | 0.051 | 1.68E-27 | 4 |
| AURKB | 1.86E-30 | 0.74585888 | 0.406 | 0.014 | 3.85E-26 | 4 |
| TOP2A | 4.29E-29 | 0.68611499 | 0.406 | 0.016 | 8.86E-25 | 4 |
| CTD-2116N | 6.55E-29 | 0.65321068 | 0.469 | 0.025 | 1.35E-24 | 4 |
| TCF19 | 3.74E-26 | 0.36343109 | 0.312 | 0.008 | 7.73E-22 | 4 |
| GINS2 | 5.12E-26 | 0.74209809 | 0.562 | 0.053 | 1.06E-21 | 4 |
| CENPU | 7.27E-26 | 0.44780551 | 0.375 | 0.016 | 1.50E-21 | 4 |
| HMMR | 1.21E-25 | 0.40383599 | 0.344 | 0.012 | 2.50E-21 | 4 |
| UBE2C | 3.18E-25 | 1.02808313 | 0.656 | 0.082 | 6.57E-21 | 4 |
| CDC20 | 8.41E-25 | 0.44173165 | 0.281 | 0.006 | 1.73E-20 | 4 |
| PTTG1 | 1.03E-24 | 0.5915516 | 0.469 | 0.033 | 2.13E-20 | 4 |
| CDKN3 | 1.88E-24 | 0.83779972 | 0.688 | 0.098 | 3.89E-20 | 4 |
| BUB1 | 3.26E-24 | 0.39019816 | 0.312 | 0.01 | 6.72E-20 | 4 |
| ASF1B | 1.39E-20 | 0.47646179 | 0.469 | 0.043 | 2.87E-16 | 4 |
| TYMS | 2.26E-20 | 0.91840645 | 0.75 | 0.157 | 4.67E-16 | 4 |
| HIST1H4C | 3.54E-20 | 1.36409698 | 0.562 | 0.076 | 7.30E-16 | 4 |
| MCM7 | 5.65E-19 | 0.95939068 | 0.906 | 0.309 | 1.17E-14 | 4 |
| RAD51 | 6.67E-19 | 0.38621748 | 0.344 | 0.023 | 1.38E-14 | 4 |
| H2AFZ | 1.07E-18 | 1.18091394 | 1 | 0.798 | 2.21E-14 | 4 |
| PRR11 | 2.02E-17 | 0.73235345 | 0.562 | 0.09 | 4.16E-13 | 4 |
| TPX2 | 1.32E-16 | 0.67427907 | 0.469 | 0.061 | 2.72E-12 | 4 |
| CDC6 | 1.34E-16 | 0.54243857 | 0.469 | 0.061 | 2.77E-12 | 4 |
| BIRC5 | 1.51E-16 | 0.49052476 | 0.406 | 0.045 | 3.12E-12 | 4 |
| CCNB2 | 3.34E-16 | 0.62321974 | 0.438 | 0.055 | 6.89E-12 | 4 |
| MELK | 2.25E-15 | 0.37656716 | 0.312 | 0.025 | 4.64E-11 | 4 |
| HMGB1 | 3.21E-15 | 0.91812645 | 1 | 0.924 | 6.62E-11 | 4 |
| NCAPG2 | 4.05E-15 | 0.52323685 | 0.469 | 0.068 | 8.36E-11 | 4 |
| STMN1 | 1.13E-14 | 0.91545441 | 0.688 | 0.178 | 2.32E-10 | 4 |
| RMI1 | 1.35E-14 | 0.33903762 | 0.375 | 0.041 | 2.79E-10 | 4 |
| RFC3 | 1.65E-14 | 0.65964673 | 0.531 | 0.1 | 3.42E-10 | 4 |
| CENPH | 6.03E-14 | 0.52120862 | 0.406 | 0.055 | 1.24E-09 | 4 |
| FEN1 | 1.52E-13 | 0.55883194 | 0.656 | 0.17 | 3.13E-09 | 4 |
| PRC1 | 2.76E-13 | 0.49297868 | 0.375 | 0.049 | 5.70E-09 | 4 |
| MT2A | 8.38E-13 | 1.3953861 | 0.781 | 0.303 | 1.73E-08 | 4 |

| | | | | | | |
|---|---|---|---|---|---|---|
| MAD2L1 | 1.01E-12 | 0.49226159 | 0.375 | 0.051 | 2.09E-08 | 4 |
| PCNA | 1.34E-12 | 0.97196285 | 0.812 | 0.354 | 2.77E-08 | 4 |
| VRK1 | 1.60E-12 | 0.57867379 | 0.531 | 0.115 | 3.30E-08 | 4 |
| DEK | 1.96E-12 | 0.70447504 | 0.812 | 0.356 | 4.05E-08 | 4 |
| MCM5 | 2.43E-12 | 0.2883374 | 0.312 | 0.033 | 5.01E-08 | 4 |
| ATAD2 | 3.20E-12 | 0.76943372 | 0.562 | 0.139 | 6.61E-08 | 4 |
| SAPCD2 | 6.17E-12 | 0.5023307 | 0.5 | 0.096 | 1.27E-07 | 4 |
| SPDEF | 1.20E-11 | 0.42142861 | 0.344 | 0.045 | 2.47E-07 | 4 |
| PLK1 | 1.24E-11 | 0.44517133 | 0.312 | 0.039 | 2.57E-07 | 4 |
| TRIP13 | 1.57E-11 | 0.48600258 | 0.375 | 0.059 | 3.24E-07 | 4 |
| HNRNPA3 | 2.24E-11 | 0.57965673 | 1 | 0.961 | 4.62E-07 | 4 |
| LMNB1 | 2.49E-11 | 0.45939002 | 0.469 | 0.096 | 5.15E-07 | 4 |
| HNRNPA2E | 2.88E-11 | 0.69519619 | 1 | 0.959 | 5.95E-07 | 4 |
| GMNN | 3.48E-11 | 0.61195267 | 0.5 | 0.114 | 7.18E-07 | 4 |
| EXOSC9 | 4.40E-11 | 0.38908521 | 0.344 | 0.049 | 9.08E-07 | 4 |
| RAD21 | 4.71E-11 | 0.69323923 | 0.844 | 0.431 | 9.72E-07 | 4 |
| SPC25 | 1.23E-10 | 0.44625179 | 0.344 | 0.055 | 2.55E-06 | 4 |
| CKS1B | 1.42E-10 | 1.0601959 | 0.812 | 0.491 | 2.93E-06 | 4 |
| PTMAP5 | 2.31E-10 | 0.64234536 | 1 | 0.961 | 4.78E-06 | 4 |
| RRM1 | 2.51E-10 | 0.48368054 | 0.469 | 0.108 | 5.18E-06 | 4 |
| RAN | 2.84E-10 | 0.56848267 | 1 | 0.998 | 5.86E-06 | 4 |
| TREX2 | 4.02E-10 | 0.23703478 | 0.281 | 0.035 | 8.29E-06 | 4 |
| HSPD1 | 5.34E-10 | 0.67062794 | 1 | 0.969 | 1.10E-05 | 4 |
| NUCKS1 | 5.68E-10 | 0.71389921 | 0.969 | 0.957 | 1.17E-05 | 4 |
| MCM2 | 8.85E-10 | 0.40220412 | 0.375 | 0.068 | 1.83E-05 | 4 |
| GINS1 | 1.15E-09 | 0.68004454 | 0.5 | 0.135 | 2.37E-05 | 4 |
| TMPO | 1.77E-09 | 0.85597492 | 0.688 | 0.315 | 3.65E-05 | 4 |
| HMGB1P5 | 1.83E-09 | 0.74263049 | 0.906 | 0.632 | 3.78E-05 | 4 |
| PTMA | 1.93E-09 | 0.67575479 | 1 | 0.986 | 3.98E-05 | 4 |
| CENPX | 1.95E-09 | 0.77174721 | 0.875 | 0.524 | 4.02E-05 | 4 |
| IGFBP5 | 2.49E-09 | 0.40248153 | 0.375 | 0.07 | 5.14E-05 | 4 |
| AURKA | 3.04E-09 | 0.66666907 | 0.531 | 0.164 | 6.27E-05 | 4 |
| SMC4 | 3.51E-09 | 0.6522245 | 0.531 | 0.164 | 7.25E-05 | 4 |
| TUBA1B | 5.70E-09 | 0.63029512 | 1 | 0.978 | 0.000118 | 4 |
| DHFR | 5.70E-09 | 0.87446227 | 0.719 | 0.384 | 0.000118 | 4 |
| C1orf21 | 5.74E-09 | 0.32879212 | 0.344 | 0.063 | 0.000118 | 4 |
| NUDT1 | 7.66E-09 | 0.41109767 | 0.406 | 0.092 | 0.000158 | 4 |
| H2AFV | 1.27E-08 | 0.61881189 | 1 | 0.853 | 0.000262 | 4 |
| TFDP1 | 1.28E-08 | 0.33387973 | 0.469 | 0.121 | 0.000264 | 4 |
| HMGN2 | 1.36E-08 | 0.76237314 | 0.875 | 0.695 | 0.00028 | 4 |
| SGO2 | 1.48E-08 | 0.34678975 | 0.312 | 0.055 | 0.000306 | 4 |
| HSP90AA1 | 1.69E-08 | 0.45686959 | 1 | 1 | 0.00035 | 4 |
| STIL | 2.45E-08 | 0.21219454 | 0.312 | 0.055 | 0.000506 | 4 |
| CDT1 | 2.53E-08 | 0.36556846 | 0.375 | 0.084 | 0.000522 | 4 |
| USP1 | 2.71E-08 | 0.55463752 | 0.531 | 0.178 | 0.000558 | 4 |
| CHEK1 | 3.25E-08 | 0.48350802 | 0.438 | 0.115 | 0.000671 | 4 |
| UBE2S | 3.54E-08 | 0.63465845 | 0.688 | 0.307 | 0.00073 | 4 |

| | | | | | | |
|---|---|---|---|---|---|---|
| HNRNPA1 | 5.03E-08 | 0.53172833 | 0.938 | 0.888 | 0.001039 | 4 |
| TUBB | 5.42E-08 | 0.57484598 | 1 | 0.975 | 0.001119 | 4 |
| HNRNPA3P | 6.83E-08 | 0.5658078 | 0.875 | 0.63 | 0.00141 | 4 |
| ANP32B | 8.21E-08 | 0.54659304 | 1 | 0.978 | 0.001694 | 4 |
| RANBP1 | 9.03E-08 | 0.70756549 | 0.875 | 0.599 | 0.001864 | 4 |
| DHTKD1 | 1.01E-07 | 0.55917939 | 0.594 | 0.25 | 0.002084 | 4 |
| SMC2 | 1.43E-07 | 0.34145212 | 0.312 | 0.063 | 0.002943 | 4 |
| PHF19 | 1.52E-07 | 0.26195645 | 0.281 | 0.051 | 0.003139 | 4 |
| MTHFD1 | 1.72E-07 | 0.67282899 | 0.781 | 0.464 | 0.003541 | 4 |
| NPM1 | 1.88E-07 | 0.38643135 | 1 | 1 | 0.003878 | 4 |
| HMGN2P17 | 3.16E-07 | 0.30007253 | 0.375 | 0.092 | 0.006517 | 4 |
| DBF4 | 3.59E-07 | 0.57217655 | 0.5 | 0.17 | 0.007413 | 4 |
| MRPS34 | 4.12E-07 | 0.62526105 | 0.875 | 0.607 | 0.008505 | 4 |
| GGCT | 4.35E-07 | 0.56609951 | 0.906 | 0.808 | 0.008978 | 4 |
| ITGB3BP | 4.95E-07 | 0.26630426 | 0.281 | 0.055 | 0.010227 | 4 |
| SP100 | 5.08E-07 | 0.47270408 | 0.688 | 0.348 | 0.010477 | 4 |
| AKR1C2 | 5.12E-07 | 0.6302672 | 0.406 | 0.106 | 0.010573 | 4 |
| XRCC2 | 5.38E-07 | 0.23042892 | 0.281 | 0.055 | 0.011112 | 4 |
| HNRNPA1P | 6.23E-07 | 0.58319118 | 0.844 | 0.575 | 0.012848 | 4 |
| ARF1 | 6.40E-07 | 0.45635615 | 1 | 0.998 | 0.013217 | 4 |
| THAP11 | 6.90E-07 | 0.22877672 | 0.281 | 0.055 | 0.014235 | 4 |
| DTYMK | 7.13E-07 | 0.51330012 | 0.5 | 0.178 | 0.014715 | 4 |
| HNRNPAB | 8.07E-07 | 0.56331843 | 0.938 | 0.869 | 0.01665 | 4 |
| CENPN | 8.27E-07 | 0.64931917 | 0.5 | 0.184 | 0.017063 | 4 |
| EIF1AX | 9.51E-07 | 0.45786058 | 0.969 | 0.886 | 0.019617 | 4 |
| UBTF | 1.34E-06 | 0.34620402 | 0.438 | 0.133 | 0.027675 | 4 |
| SPTSSB | 1.36E-06 | 0.48036719 | 0.969 | 0.746 | 0.027972 | 4 |
| SET | 1.46E-06 | 0.42251834 | 1 | 0.988 | 0.030227 | 4 |
| H2AFZP3 | 1.76E-06 | 0.59873243 | 0.625 | 0.327 | 0.036237 | 4 |
| IRX3 | 1.88E-06 | 0.42170689 | 0.656 | 0.297 | 0.038728 | 4 |
| SNRPA | 2.04E-06 | 0.40661377 | 0.5 | 0.188 | 0.042026 | 4 |
| CCT5 | 2.09E-06 | 0.42782474 | 0.969 | 0.873 | 0.043205 | 4 |
| ZWILCH | 2.37E-06 | 0.22195371 | 0.375 | 0.1 | 0.048873 | 4 |
| BTBD3 | 2.71E-06 | 0.32362459 | 0.281 | 0.061 | 0.055893 | 4 |
| SLC25A5 | 2.77E-06 | 0.43624083 | 1 | 0.971 | 0.057181 | 4 |
| NCAPD2 | 2.91E-06 | 0.37462519 | 0.375 | 0.108 | 0.060065 | 4 |
| RPP25 | 3.23E-06 | 0.5285414 | 0.594 | 0.292 | 0.066688 | 4 |
| NPR3 | 3.24E-06 | 0.51609831 | 0.438 | 0.145 | 0.066841 | 4 |
| SNRNP25 | 3.41E-06 | 0.42385322 | 0.656 | 0.354 | 0.07035 | 4 |
| RP11-111M | 3.51E-06 | 0.30248659 | 0.312 | 0.074 | 0.072364 | 4 |
| NUP54 | 3.63E-06 | 0.46307807 | 0.5 | 0.188 | 0.074918 | 4 |
| PTGES3 | 4.15E-06 | 0.58544814 | 0.906 | 0.779 | 0.085584 | 4 |
| TFF1 | 4.40E-06 | 0.71853681 | 0.969 | 0.908 | 0.090743 | 4 |
| TBL1X | 4.46E-06 | 0.49589208 | 0.594 | 0.284 | 0.092094 | 4 |
| LSM4 | 4.62E-06 | 0.63446347 | 0.875 | 0.742 | 0.095271 | 4 |
| DUT | 5.54E-06 | 0.69607793 | 0.625 | 0.37 | 0.11425 | 4 |
| NCAPD3 | 5.90E-06 | 0.27166735 | 0.344 | 0.094 | 0.121703 | 4 |

| | | | | | | |
|---|---|---|---|---|---|---|
| GINS4 | 6.09E-06 | 0.42352201 | 0.344 | 0.1 | 0.125672 | 4 |
| H1FX | 6.17E-06 | 0.4849405 | 0.406 | 0.137 | 0.127277 | 4 |
| TPI1 | 6.20E-06 | 0.53353679 | 0.906 | 0.869 | 0.127961 | 4 |
| UNG | 6.56E-06 | 0.51831411 | 0.438 | 0.159 | 0.135465 | 4 |
| RFC2 | 7.52E-06 | 0.49996974 | 0.656 | 0.362 | 0.155244 | 4 |
| COPS3 | 7.70E-06 | 0.43505685 | 0.719 | 0.413 | 0.158976 | 4 |
| CACYBP | 7.72E-06 | 0.52195372 | 0.906 | 0.785 | 0.159433 | 4 |
| HELLS | 8.63E-06 | 0.57330794 | 0.312 | 0.084 | 0.178052 | 4 |
| HNRNPA1L | 8.76E-06 | 0.43758835 | 0.781 | 0.581 | 0.180702 | 4 |
| TPM3 | 8.83E-06 | 0.42019963 | 0.938 | 0.836 | 0.182343 | 4 |
| CDCA4 | 8.90E-06 | 0.39838544 | 0.5 | 0.198 | 0.183615 | 4 |
| UBE2T | 8.95E-06 | 0.40722857 | 0.438 | 0.16 | 0.184768 | 4 |
| TPD52L1 | 9.24E-06 | 0.62581961 | 0.938 | 0.824 | 0.190707 | 4 |
| HNRNPA3P | 9.32E-06 | 0.52903272 | 0.781 | 0.571 | 0.192416 | 4 |
| CCNB1 | 9.37E-06 | 0.58511882 | 0.5 | 0.213 | 0.193405 | 4 |
| CENPK | 9.72E-06 | 0.3200107 | 0.375 | 0.114 | 0.200568 | 4 |
| SF3A1 | 1.01E-05 | 0.45590093 | 0.75 | 0.464 | 0.207847 | 4 |
| SNHG15 | 1.06E-05 | 0.22617671 | 0.312 | 0.082 | 0.217911 | 4 |
| PKMYT1 | 1.09E-05 | 0.39856506 | 0.625 | 0.319 | 0.224021 | 4 |
| BANF1 | 1.12E-05 | 0.4219377 | 0.969 | 0.881 | 0.230713 | 4 |
| CYC1 | 1.16E-05 | 0.50233739 | 0.906 | 0.665 | 0.240394 | 4 |
| RPS2P7 | 1.29E-05 | 0.45162876 | 1 | 1 | 0.266141 | 4 |
| MMACHC | 1.35E-05 | 0.35532472 | 1 | 0.945 | 0.278037 | 4 |
| HNRNPA3P | 1.39E-05 | 0.52516771 | 0.781 | 0.64 | 0.286921 | 4 |
| CCT6A | 1.44E-05 | 0.44920612 | 0.938 | 0.863 | 0.296893 | 4 |
| RNASEH2A | 1.49E-05 | 0.25975221 | 0.312 | 0.082 | 0.307271 | 4 |
| C9orf40 | 1.69E-05 | 0.1825914 | 0.312 | 0.08 | 0.349525 | 4 |
| PPP1CC | 1.73E-05 | 0.51676195 | 0.781 | 0.507 | 0.356605 | 4 |
| CSE1L | 1.75E-05 | 0.4237681 | 0.875 | 0.624 | 0.36136 | 4 |
| BAG2 | 1.80E-05 | 0.43080419 | 0.656 | 0.339 | 0.371075 | 4 |
| CMC2 | 1.91E-05 | 0.50708178 | 0.844 | 0.597 | 0.393616 | 4 |
| RP11-498C | 1.95E-05 | 0.26906735 | 0.281 | 0.07 | 0.402137 | 4 |
| LINC02001 | 2.00E-05 | 0.32694167 | 0.344 | 0.106 | 0.413484 | 4 |
| KRT18 | 2.01E-05 | 0.48856687 | 0.875 | 0.8 | 0.414536 | 4 |
| RPS2P46 | 2.04E-05 | 0.47303319 | 1 | 0.996 | 0.420351 | 4 |
| PCBP1 | 2.11E-05 | 0.43707135 | 1 | 0.961 | 0.434577 | 4 |
| CYCS | 2.19E-05 | 0.55270598 | 0.875 | 0.888 | 0.452981 | 4 |
| LSM5 | 2.24E-05 | 0.47790487 | 0.844 | 0.726 | 0.462875 | 4 |
| AKR1C1 | 2.29E-05 | 0.53300807 | 0.406 | 0.131 | 0.473339 | 4 |
| RP11-152C | 2.36E-05 | 0.52855389 | 0.531 | 0.241 | 0.488096 | 4 |
| NHP2 | 2.39E-05 | 0.48719528 | 0.938 | 0.935 | 0.492295 | 4 |
| RBBP7 | 2.41E-05 | 0.52485433 | 0.812 | 0.601 | 0.498092 | 4 |
| FBL | 2.52E-05 | 0.43285344 | 0.75 | 0.62 | 0.520699 | 4 |
| RPS2P4 | 2.56E-05 | 0.54405638 | 1 | 0.973 | 0.528973 | 4 |
| FANCD2 | 2.60E-05 | 0.41804082 | 0.375 | 0.127 | 0.53696 | 4 |
| LRRC59 | 2.89E-05 | 0.51150546 | 0.719 | 0.491 | 0.596897 | 4 |
| PSMG1 | 3.05E-05 | 0.47489444 | 0.562 | 0.286 | 0.629474 | 4 |

| | | | | | | |
|---|---|---|---|---|---|---|
| EIF4H | 3.30E-05 | 0.40063128 | 1 | 0.998 | 0.681257 | 4 |
| RP13-1032 | 3.49E-05 | 0.36823793 | 0.438 | 0.17 | 0.72088 | 4 |
| MIS18A | 4.29E-05 | 0.27329354 | 0.438 | 0.166 | 0.886254 | 4 |
| PAICS | 4.35E-05 | 0.47256153 | 0.875 | 0.714 | 0.897574 | 4 |
| BUB3 | 4.38E-05 | 0.66061588 | 0.688 | 0.384 | 0.903754 | 4 |
| MCM3 | 4.40E-05 | 0.39932855 | 0.438 | 0.17 | 0.907158 | 4 |
| C16orf91 | 4.42E-05 | 0.41046856 | 0.562 | 0.266 | 0.911412 | 4 |
| EIF4EBP2 | 4.46E-05 | 0.4325187 | 0.75 | 0.499 | 0.921126 | 4 |
| DNMT1 | 4.88E-05 | 0.45331254 | 0.562 | 0.288 | 1 | 4 |
| DHFR2 | 4.91E-05 | 0.32470451 | 0.406 | 0.153 | 1 | 4 |
| HSPA9 | 5.04E-05 | 0.48847155 | 0.875 | 0.712 | 1 | 4 |
| CALM1 | 5.22E-05 | 0.4296912 | 1 | 0.932 | 1 | 4 |
| MCM6 | 5.23E-05 | 0.4474828 | 0.438 | 0.186 | 1 | 4 |
| KPNB1 | 5.36E-05 | 0.45857411 | 0.906 | 0.928 | 1 | 4 |
| XRCC5 | 5.47E-05 | 0.39370833 | 1 | 0.759 | 1 | 4 |
| CHCHD2 | 5.65E-05 | 0.30689618 | 1 | 0.992 | 1 | 4 |
| HMGN1 | 5.67E-05 | 0.30691067 | 0.969 | 0.973 | 1 | 4 |
| H3F3B | 5.87E-05 | 0.25045897 | 1 | 0.99 | 1 | 4 |
| DCLRE1B | 6.04E-05 | 0.44784148 | 0.531 | 0.241 | 1 | 4 |
| RP11-669B | 6.05E-05 | 0.42439485 | 0.531 | 0.237 | 1 | 4 |
| TRIM28 | 6.63E-05 | 0.40218923 | 0.844 | 0.718 | 1 | 4 |
| GLRX5 | 6.79E-05 | 0.37330665 | 0.844 | 0.706 | 1 | 4 |
| LSM2 | 7.08E-05 | 0.49950677 | 0.625 | 0.366 | 1 | 4 |
| HMGN2P46 | 7.11E-05 | 0.29600935 | 0.438 | 0.17 | 1 | 4 |
| MRPL35 | 7.35E-05 | 0.44947987 | 0.594 | 0.319 | 1 | 4 |
| TUBAP2 | 7.39E-05 | 0.50679314 | 0.969 | 0.918 | 1 | 4 |
| FARSB | 7.47E-05 | 0.4197435 | 0.562 | 0.29 | 1 | 4 |
| HMGB1P37 | 7.66E-05 | 0.25378525 | 0.312 | 0.096 | 1 | 4 |
| THOC7 | 7.92E-05 | 0.31938487 | 0.969 | 0.982 | 1 | 4 |
| RP11-192C | 8.02E-05 | 0.49032797 | 1 | 0.957 | 1 | 4 |
| PHGDH | 8.08E-05 | 0.45229814 | 0.719 | 0.487 | 1 | 4 |
| DDX39A | 8.33E-05 | 0.40119175 | 0.344 | 0.115 | 1 | 4 |
| AC022210. | 8.72E-05 | 0.34743519 | 0.938 | 0.767 | 1 | 4 |
| CKS2 | 8.83E-05 | 0.38843023 | 0.469 | 0.202 | 1 | 4 |
| RABL6 | 9.09E-05 | 0.4544044 | 0.562 | 0.294 | 1 | 4 |
| EZH2 | 9.13E-05 | 0.33612706 | 0.344 | 0.119 | 1 | 4 |
| NOP56 | 9.63E-05 | 0.43515729 | 0.656 | 0.417 | 1 | 4 |
| RAP2A | 9.78E-05 | 0.29443222 | 0.281 | 0.08 | 1 | 4 |
| ZC3H15 | 9.98E-05 | 0.39491268 | 0.906 | 0.822 | 1 | 4 |
| TUBA1A | 0.00011 | 0.43437515 | 0.969 | 0.935 | 1 | 4 |
| SHMT1 | 0.000116 | 0.30919978 | 0.344 | 0.123 | 1 | 4 |
| TBC1D31 | 0.000119 | 0.23623107 | 0.312 | 0.1 | 1 | 4 |
| RP11-303E | 0.000131 | 0.31512643 | 0.281 | 0.084 | 1 | 4 |
| PRDX1 | 0.000132 | 0.42218516 | 0.969 | 0.98 | 1 | 4 |
| YBX1 | 0.000135 | 0.33051927 | 1 | 0.982 | 1 | 4 |
| MRPL3 | 0.000138 | 0.40481322 | 0.875 | 0.718 | 1 | 4 |
| TMA7 | 0.000146 | 0.33607465 | 0.938 | 0.886 | 1 | 4 |

| | | | | | | |
|---|---|---|---|---|---|---|
| APIP | 0.00015 | 0.37859632 | 0.562 | 0.297 | 1 | 4 |
| ARHGAP4 | 0.000153 | 0.31859512 | 0.469 | 0.207 | 1 | 4 |
| COQ5 | 0.000153 | 0.36441587 | 0.344 | 0.119 | 1 | 4 |
| LBHD1 | 0.000153 | 0.41432499 | 0.781 | 0.573 | 1 | 4 |
| FAM120AC | 0.000159 | 0.41329866 | 0.688 | 0.446 | 1 | 4 |
| RP11-3P17 | 0.000159 | 0.43716528 | 0.75 | 0.464 | 1 | 4 |
| TRMT5 | 0.000162 | 0.4335217 | 0.688 | 0.499 | 1 | 4 |
| RPS2 | 0.000164 | 0.3833795 | 1 | 1 | 1 | 4 |
| IMP4 | 0.000172 | 0.34117596 | 0.75 | 0.523 | 1 | 4 |
| RP11-298J2 | 0.000178 | 0.58029587 | 0.844 | 0.82 | 1 | 4 |
| BOLA3 | 0.000185 | 0.37182439 | 0.875 | 0.796 | 1 | 4 |
| C11orf24 | 0.000189 | 0.2875741 | 0.594 | 0.321 | 1 | 4 |
| AC004069. | 0.000198 | 0.38285112 | 0.688 | 0.448 | 1 | 4 |
| TOMM6 | 0.000208 | 0.50101061 | 0.844 | 0.816 | 1 | 4 |
| RAD51C | 0.000211 | 0.47574303 | 0.844 | 0.722 | 1 | 4 |
| CDC123 | 0.000211 | 0.38115757 | 0.844 | 0.679 | 1 | 4 |
| RPL23AP57 | 0.000211 | 0.3861217 | 0.594 | 0.344 | 1 | 4 |
| NDC1 | 0.000212 | 0.23268075 | 0.438 | 0.172 | 1 | 4 |
| CLEC2D | 0.000218 | 0.31157571 | 1 | 0.949 | 1 | 4 |
| SFPQ | 0.000235 | 0.50856441 | 0.75 | 0.618 | 1 | 4 |
| WDR5 | 0.000242 | 0.34925918 | 0.5 | 0.247 | 1 | 4 |
| HSPA8 | 0.000247 | 0.37496191 | 1 | 0.973 | 1 | 4 |
| SAC3D1 | 0.000252 | 0.44137436 | 0.469 | 0.249 | 1 | 4 |
| POLR3K | 0.000254 | 0.37966462 | 0.781 | 0.558 | 1 | 4 |
| RNF168 | 0.000279 | 0.41477482 | 0.5 | 0.258 | 1 | 4 |
| LSM6 | 0.00028 | 0.32957036 | 0.406 | 0.172 | 1 | 4 |
| GCSH | 0.000282 | 0.43000667 | 0.844 | 0.712 | 1 | 4 |
| NET1 | 0.000288 | 0.49855825 | 0.5 | 0.243 | 1 | 4 |
| GADD45GII | 0.000289 | 0.3176575 | 0.75 | 0.601 | 1 | 4 |
| HNRNPUP1 | 0.000292 | 0.15846141 | 0.281 | 0.088 | 1 | 4 |
| UBE2D1 | 0.000294 | 0.34219677 | 0.344 | 0.129 | 1 | 4 |
| KNSTRN | 0.000303 | 0.2305534 | 0.312 | 0.11 | 1 | 4 |
| COMMD4 | 0.000318 | 0.27981174 | 0.656 | 0.364 | 1 | 4 |
| SNRPGP10 | 0.000341 | 0.426507 | 0.938 | 0.748 | 1 | 4 |
| UQCR10 | 0.000341 | 0.39212864 | 0.875 | 0.806 | 1 | 4 |
| CDC25B | 0.00036 | 0.30891514 | 0.656 | 0.386 | 1 | 4 |
| CIAO1 | 0.000364 | 0.3557562 | 0.719 | 0.477 | 1 | 4 |
| CREM | 0.000367 | 0.40039265 | 0.688 | 0.481 | 1 | 4 |
| BRI3BP | 0.000373 | 0.5978534 | 0.781 | 0.618 | 1 | 4 |
| NTMT1 | 0.000389 | 0.30882441 | 0.375 | 0.16 | 1 | 4 |
| MRM2 | 0.000389 | 0.27130595 | 0.531 | 0.27 | 1 | 4 |
| JTB | 0.00039 | 0.40361903 | 1 | 0.947 | 1 | 4 |
| ATP5G3 | 0.000394 | 0.38026677 | 0.906 | 0.947 | 1 | 4 |
| RP11-12G1 | 0.000411 | 0.17038049 | 0.312 | 0.104 | 1 | 4 |
| SRSF3 | 0.000426 | 0.5704401 | 0.906 | 0.818 | 1 | 4 |
| DPM2 | 0.000442 | 0.4226982 | 0.438 | 0.213 | 1 | 4 |
| BLVRB | 0.000456 | 0.50622837 | 0.812 | 0.669 | 1 | 4 |

| | | | | | | |
|---|---|---|---|---|---|---|
| PMVK | 0.00049 | 0.27588794 | 0.5 | 0.233 | 1 | 4 |
| RPS2P48 | 0.000512 | 0.52884544 | 1 | 0.969 | 1 | 4 |
| COA1 | 0.000513 | 0.36805678 | 0.469 | 0.233 | 1 | 4 |
| FAM120A | 0.000523 | 0.46805152 | 0.906 | 0.836 | 1 | 4 |
| CCDC34 | 0.000565 | 0.40579381 | 0.375 | 0.162 | 1 | 4 |
| ENOPH1 | 0.000572 | 0.29992825 | 0.469 | 0.221 | 1 | 4 |
| MCUB | 0.000591 | 0.28854466 | 0.281 | 0.098 | 1 | 4 |
| YWHAQ | 0.000624 | 0.32643934 | 0.969 | 0.902 | 1 | 4 |
| NLN | 0.000626 | 0.6258161 | 0.594 | 0.362 | 1 | 4 |
| TAGLN2 | 0.000626 | 0.44445402 | 0.875 | 0.718 | 1 | 4 |
| SUV39H2 | 0.000629 | 0.30574059 | 0.375 | 0.157 | 1 | 4 |
| GLO1 | 0.000634 | 0.41772078 | 0.75 | 0.579 | 1 | 4 |
| SRSF2 | 0.00064 | 0.45566765 | 0.844 | 0.683 | 1 | 4 |
| SORD2P | 0.000663 | 0.24690126 | 0.531 | 0.26 | 1 | 4 |
| RP11-296A | 0.000668 | 0.4203365 | 0.969 | 0.977 | 1 | 4 |
| MND1 | 0.000676 | 0.3115511 | 0.438 | 0.204 | 1 | 4 |
| IER5L | 0.000689 | 0.31381748 | 0.406 | 0.19 | 1 | 4 |
| CCDC85B | 0.000692 | 0.35705586 | 0.5 | 0.284 | 1 | 4 |
| CDV3 | 0.000698 | 0.45941913 | 0.844 | 0.81 | 1 | 4 |
| ECE2 | 0.000724 | 0.33361472 | 0.531 | 0.295 | 1 | 4 |
| GON7 | 0.000748 | 0.30636665 | 0.406 | 0.182 | 1 | 4 |
| FAM217B | 0.000753 | 0.41073012 | 0.688 | 0.476 | 1 | 4 |
| DLEU1 | 0.000755 | 0.24811721 | 0.344 | 0.141 | 1 | 4 |
| DNASE1 | 0.000767 | 0.31481237 | 0.625 | 0.407 | 1 | 4 |
| MFSD2B | 0.000779 | 0.26657374 | 0.406 | 0.188 | 1 | 4 |
| NHP2P1 | 0.000835 | 0.37543127 | 0.938 | 0.855 | 1 | 4 |
| SLBP | 0.000851 | 0.30965172 | 0.5 | 0.249 | 1 | 4 |
| CHMP4A | 0.000856 | 0.31902996 | 0.688 | 0.481 | 1 | 4 |
| HNRNPA0 | 0.000918 | 0.41891521 | 0.844 | 0.687 | 1 | 4 |
| RANP1 | 0.000923 | 0.26311915 | 0.812 | 0.599 | 1 | 4 |
| PGAM1 | 0.000931 | 0.4215456 | 0.969 | 0.93 | 1 | 4 |
| ZDHHC12 | 0.000937 | 0.24033381 | 0.344 | 0.133 | 1 | 4 |
| ARRB2 | 0.000961 | 0.27538003 | 0.312 | 0.121 | 1 | 4 |
| KIF23 | 0.000964 | 0.18606099 | 0.312 | 0.115 | 1 | 4 |
| PRDX1P1 | 0.000972 | 0.39348006 | 0.75 | 0.74 | 1 | 4 |
| GNB2 | 0.000976 | 0.38556292 | 0.812 | 0.71 | 1 | 4 |
| GAPDH | 0.000996 | 0.35917257 | 0.969 | 0.99 | 1 | 4 |
| PDSS1 | 0.00103 | 0.37295845 | 0.406 | 0.18 | 1 | 4 |
| MRPL57 | 0.001079 | 0.2893907 | 0.844 | 0.605 | 1 | 4 |
| ALG13 | 0.001096 | 0.32337058 | 0.688 | 0.446 | 1 | 4 |
| SRSF6 | 0.001105 | 0.34777949 | 0.625 | 0.429 | 1 | 4 |
| AK6 | 0.00112 | 0.27325939 | 0.594 | 0.358 | 1 | 4 |
| PAQR4 | 0.001136 | 0.20290038 | 0.344 | 0.135 | 1 | 4 |
| SNRPG | 0.001175 | 0.34803257 | 0.906 | 0.773 | 1 | 4 |
| SLC25A5P1 | 0.001228 | 0.19738817 | 0.344 | 0.147 | 1 | 4 |
| RAB8A | 0.001231 | 0.25449308 | 0.5 | 0.264 | 1 | 4 |
| NASP | 0.001254 | 0.39000253 | 0.531 | 0.311 | 1 | 4 |

| | | | | | | |
|---|---|---|---|---|---|---|
| PGK1 | 0.001259 | 0.27722193 | 1 | 0.932 | 1 | 4 |
| MTFP1 | 0.001261 | 0.19534125 | 0.312 | 0.119 | 1 | 4 |
| EIF5 | 0.001274 | 0.29048064 | 0.969 | 0.937 | 1 | 4 |
| HAT1 | 0.001296 | 0.39132596 | 0.75 | 0.597 | 1 | 4 |
| HNRNPA1P | 0.001344 | 0.24751642 | 0.594 | 0.352 | 1 | 4 |
| KLF5 | 0.001345 | 0.24496997 | 0.469 | 0.243 | 1 | 4 |
| SLC25A11 | 0.001362 | 0.31113618 | 0.406 | 0.188 | 1 | 4 |
| SDHD | 0.001375 | 0.4752824 | 0.625 | 0.407 | 1 | 4 |
| FANCI | 0.00138 | 0.23529279 | 0.312 | 0.125 | 1 | 4 |
| GOT1 | 0.00138 | 0.37527297 | 0.5 | 0.294 | 1 | 4 |
| CENPP | 0.001418 | 0.33673683 | 0.406 | 0.194 | 1 | 4 |
| DNAJC19 | 0.001441 | 0.50526156 | 0.656 | 0.47 | 1 | 4 |
| MPC2 | 0.00148 | 0.26687272 | 0.688 | 0.446 | 1 | 4 |
| RUVBL1 | 0.001508 | 0.37932621 | 0.625 | 0.434 | 1 | 4 |
| ILF2 | 0.001537 | 0.43550571 | 0.844 | 0.71 | 1 | 4 |
| COMT | 0.001548 | 0.33963085 | 0.906 | 0.775 | 1 | 4 |
| SMYD5 | 0.001575 | 0.14469853 | 0.281 | 0.098 | 1 | 4 |
| SRGAP2B | 0.001583 | 0.22015344 | 0.344 | 0.141 | 1 | 4 |
| TSEN34 | 0.001586 | 0.37834404 | 0.844 | 0.705 | 1 | 4 |
| G6PD | 0.00161 | 0.33603012 | 0.906 | 0.873 | 1 | 4 |
| PAPOLA | 0.001668 | 0.39263727 | 0.906 | 0.84 | 1 | 4 |
| UGDH | 0.00167 | 0.3480534 | 0.562 | 0.36 | 1 | 4 |
| FAM122B | 0.001711 | 0.37757389 | 0.344 | 0.151 | 1 | 4 |
| MZT2A | 0.001723 | 0.31029197 | 0.969 | 0.881 | 1 | 4 |
| PSAT1 | 0.001733 | 0.41227131 | 0.719 | 0.552 | 1 | 4 |
| KPNA2 | 0.001738 | 0.3569898 | 0.781 | 0.748 | 1 | 4 |
| RP11-386I2 | 0.001753 | 0.26303646 | 0.375 | 0.168 | 1 | 4 |
| CBX3 | 0.00176 | 0.3685746 | 0.938 | 0.871 | 1 | 4 |
| SLC39A6 | 0.001773 | 0.4874931 | 0.688 | 0.489 | 1 | 4 |
| PTP4A1 | 0.001815 | 0.54798974 | 0.781 | 0.683 | 1 | 4 |
| YWHAH | 0.001869 | 0.27846862 | 0.469 | 0.235 | 1 | 4 |
| RPS2P5 | 0.001872 | 0.33315552 | 1 | 0.992 | 1 | 4 |
| CDK2AP2 | 0.001947 | 0.293485 | 0.844 | 0.695 | 1 | 4 |
| GRHL1 | 0.002013 | 0.14650665 | 0.312 | 0.123 | 1 | 4 |
| SERBP1 | 0.002038 | 0.28070561 | 0.875 | 0.939 | 1 | 4 |
| CPT2 | 0.002055 | 0.33227834 | 0.5 | 0.284 | 1 | 4 |
| AC007969. | 0.002107 | 0.28116277 | 0.906 | 0.751 | 1 | 4 |
| MASTL | 0.002125 | 0.27851002 | 0.312 | 0.131 | 1 | 4 |
| SRM | 0.002126 | 0.32039402 | 0.812 | 0.62 | 1 | 4 |
| SAE1 | 0.002161 | 0.32912706 | 0.562 | 0.354 | 1 | 4 |
| HNRNPF | 0.002183 | 0.62203157 | 0.781 | 0.742 | 1 | 4 |
| PMF1 | 0.002215 | 0.31760922 | 0.594 | 0.403 | 1 | 4 |
| STOML2 | 0.002307 | 0.38359496 | 0.562 | 0.376 | 1 | 4 |
| TCERG1 | 0.002308 | 0.30410363 | 0.531 | 0.288 | 1 | 4 |
| RP11-364L | 0.002315 | 0.39990106 | 1 | 0.988 | 1 | 4 |
| PFN1 | 0.00232 | 0.27180814 | 1 | 0.996 | 1 | 4 |
| SIVA1 | 0.00234 | 0.35063376 | 0.656 | 0.489 | 1 | 4 |

| | | | | | | |
|---|---|---|---|---|---|---|
| GATA3 | 0.00236 | 0.51706882 | 0.812 | 0.775 | 1 | 4 |
| ERH | 0.0024 | 0.29546938 | 0.969 | 0.949 | 1 | 4 |
| BZW1 | 0.00242 | 0.26880391 | 0.906 | 0.82 | 1 | 4 |
| ENO1 | 0.002439 | 0.25895196 | 1 | 0.986 | 1 | 4 |
| RP3-342P2( | 0.002443 | 0.41726339 | 0.969 | 0.973 | 1 | 4 |
| FBXW11 | 0.002482 | 0.21428892 | 0.594 | 0.342 | 1 | 4 |
| MROH7-TT | 0.002487 | 0.23941464 | 0.281 | 0.115 | 1 | 4 |
| TCOF1 | 0.002516 | 0.31787212 | 0.406 | 0.202 | 1 | 4 |
| VKORC1 | 0.00252 | 0.26352681 | 0.5 | 0.29 | 1 | 4 |
| HNRNPU | 0.002521 | 0.39913514 | 0.781 | 0.73 | 1 | 4 |
| AARS | 0.002541 | 0.35345982 | 0.844 | 0.728 | 1 | 4 |
| RBBP8 | 0.002564 | 0.29801385 | 0.406 | 0.198 | 1 | 4 |
| EIF2S1 | 0.002625 | 0.32531761 | 0.812 | 0.728 | 1 | 4 |
| RMND5B | 0.002642 | 0.34599477 | 0.906 | 0.89 | 1 | 4 |
| THAP12 | 0.002651 | 0.37331751 | 0.531 | 0.352 | 1 | 4 |
| DDX46 | 0.00272 | 0.35821676 | 0.656 | 0.472 | 1 | 4 |
| NIP7 | 0.002744 | 0.29126733 | 0.656 | 0.493 | 1 | 4 |
| ZP3 | 0.002821 | 0.24338181 | 0.469 | 0.243 | 1 | 4 |
| ARPC1A | 0.002823 | 0.31161518 | 0.875 | 0.693 | 1 | 4 |
| PAK4 | 0.002838 | 0.34251711 | 0.469 | 0.282 | 1 | 4 |
| DCTN3 | 0.002864 | 0.3022719 | 0.5 | 0.282 | 1 | 4 |
| PSMG3 | 0.002893 | 0.33185884 | 0.562 | 0.352 | 1 | 4 |
| FAM216A | 0.002928 | 0.31561142 | 0.375 | 0.192 | 1 | 4 |
| PSMC6 | 0.002931 | 0.29879974 | 0.875 | 0.683 | 1 | 4 |
| IRS1 | 0.00303 | 0.29108859 | 0.375 | 0.182 | 1 | 4 |
| STRN4 | 0.003034 | 0.36166709 | 0.469 | 0.272 | 1 | 4 |
| PPAT | 0.003043 | 0.36940273 | 0.438 | 0.217 | 1 | 4 |
| TRIM59 | 0.003094 | 0.32672603 | 0.438 | 0.262 | 1 | 4 |
| HNRNPA3P | 0.003222 | 0.31370125 | 0.781 | 0.681 | 1 | 4 |
| ANAPC11 | 0.003304 | 0.38529445 | 0.781 | 0.661 | 1 | 4 |
| SNHG3 | 0.003341 | 0.3577529 | 0.531 | 0.329 | 1 | 4 |
| PRPS1 | 0.003397 | 0.4393598 | 0.656 | 0.493 | 1 | 4 |
| TST | 0.003467 | 0.30156256 | 0.5 | 0.29 | 1 | 4 |
| EXOSC8 | 0.003528 | 0.32998582 | 0.5 | 0.321 | 1 | 4 |
| SVIP | 0.003546 | 0.31814344 | 0.781 | 0.595 | 1 | 4 |
| BCL11B | 0.003687 | 0.27791742 | 0.281 | 0.115 | 1 | 4 |
| PAGR1 | 0.003693 | 0.23485915 | 0.406 | 0.213 | 1 | 4 |
| TOMM34 | 0.00371 | 0.29704241 | 0.625 | 0.419 | 1 | 4 |
| GREB1 | 0.003847 | 0.4255329 | 0.406 | 0.227 | 1 | 4 |
| RP11-824M | 0.003852 | 0.27585936 | 0.906 | 0.806 | 1 | 4 |
| C2orf69 | 0.003928 | 0.31851147 | 0.438 | 0.266 | 1 | 4 |
| KPNA2P3 | 0.003946 | 0.26900785 | 0.406 | 0.205 | 1 | 4 |
| AP2M1 | 0.003953 | 0.24908197 | 0.906 | 0.834 | 1 | 4 |
| HEATR3 | 0.004002 | 0.31192003 | 0.312 | 0.135 | 1 | 4 |
| SRP68 | 0.004068 | 0.28096103 | 0.469 | 0.286 | 1 | 4 |
| CCT8 | 0.004075 | 0.38156301 | 0.781 | 0.669 | 1 | 4 |
| PRPF8 | 0.00408 | 0.28900935 | 0.688 | 0.481 | 1 | 4 |

| | | | | | | |
|---|---|---|---|---|---|---|
| TMED2 | 0.004142 | 0.39099743 | 0.812 | 0.748 | 1 | 4 |
| RPLP0 | 0.004189 | 0.16009489 | 1 | 1 | 1 | 4 |
| MTPN | 0.004214 | 0.2114201 | 0.75 | 0.56 | 1 | 4 |
| SDHB | 0.004228 | 0.36434774 | 0.594 | 0.421 | 1 | 4 |
| MED8 | 0.004259 | 0.22856041 | 0.406 | 0.205 | 1 | 4 |
| LARS | 0.004263 | 0.28992492 | 0.562 | 0.382 | 1 | 4 |
| MRPS12 | 0.004273 | 0.46040601 | 0.688 | 0.599 | 1 | 4 |
| VTI1B | 0.004334 | 0.29410486 | 0.844 | 0.654 | 1 | 4 |
| NABP2 | 0.00438 | 0.18893418 | 0.344 | 0.157 | 1 | 4 |
| PHTF2 | 0.004403 | 0.30636176 | 0.438 | 0.241 | 1 | 4 |
| MORF4L2 | 0.004435 | 0.35798489 | 0.875 | 0.81 | 1 | 4 |
| FLOT1 | 0.004465 | 0.2554481 | 0.375 | 0.182 | 1 | 4 |
| SNRPGP15 | 0.004469 | 0.35822572 | 0.688 | 0.519 | 1 | 4 |
| RP11-298C | 0.004525 | 0.32899161 | 0.438 | 0.262 | 1 | 4 |
| RCC2 | 0.00456 | 0.27363228 | 0.75 | 0.552 | 1 | 4 |
| RP11-446E! | 0.004607 | 0.29089588 | 0.406 | 0.213 | 1 | 4 |
| BDH1 | 0.004712 | 0.34577579 | 0.469 | 0.276 | 1 | 4 |
| LSM3 | 0.004748 | 0.44625975 | 0.781 | 0.765 | 1 | 4 |
| AGAP3 | 0.00475 | 0.17363473 | 0.375 | 0.176 | 1 | 4 |
| NXT1 | 0.004753 | 0.28097361 | 0.562 | 0.366 | 1 | 4 |
| PRDX2P4 | 0.004772 | 0.2521245 | 0.562 | 0.362 | 1 | 4 |
| BRIX1 | 0.004885 | 0.30355701 | 0.562 | 0.376 | 1 | 4 |
| SYNE2 | 0.004887 | 0.24929311 | 0.5 | 0.288 | 1 | 4 |
| POLE3 | 0.004937 | 0.46743937 | 0.656 | 0.505 | 1 | 4 |
| SSRP1 | 0.00495 | 0.31125382 | 0.625 | 0.415 | 1 | 4 |
| PHB2 | 0.004958 | 0.27477176 | 0.719 | 0.534 | 1 | 4 |
| SYS1-DBND | 0.005041 | 0.31200328 | 0.406 | 0.231 | 1 | 4 |
| PPIAL4G | 0.005049 | 0.31629214 | 0.938 | 0.851 | 1 | 4 |
| HTATSF1 | 0.005055 | 0.35464811 | 0.438 | 0.26 | 1 | 4 |
| SLC25A39 | 0.005066 | 0.28132495 | 0.969 | 0.914 | 1 | 4 |
| C7orf50 | 0.005129 | 0.43897647 | 0.562 | 0.378 | 1 | 4 |
| ASS1 | 0.005151 | 0.24631919 | 1 | 0.969 | 1 | 4 |
| HN1 | 0.005157 | 0.29620741 | 0.906 | 0.867 | 1 | 4 |
| ARL5A | 0.005299 | 0.41508938 | 0.719 | 0.679 | 1 | 4 |
| ANP32E | 0.005314 | 0.3724699 | 0.625 | 0.495 | 1 | 4 |
| SLC25A1 | 0.005415 | 0.25934691 | 0.781 | 0.622 | 1 | 4 |
| BRD7 | 0.005552 | 0.29076232 | 0.469 | 0.295 | 1 | 4 |
| MDH2 | 0.005563 | 0.35211552 | 0.969 | 0.967 | 1 | 4 |
| NONO | 0.005592 | 0.24839965 | 0.969 | 0.885 | 1 | 4 |
| AC007161.: | 0.005598 | 0.2619392 | 0.5 | 0.305 | 1 | 4 |
| FBRSL1 | 0.005656 | 0.23044614 | 0.5 | 0.286 | 1 | 4 |
| H2AFX | 0.005658 | 0.36035784 | 0.406 | 0.247 | 1 | 4 |
| RP11-452G | 0.005741 | 0.34431229 | 0.688 | 0.573 | 1 | 4 |
| RP11-567G | 0.005776 | 0.19896687 | 0.406 | 0.204 | 1 | 4 |
| MCM4 | 0.005837 | 0.29383151 | 0.375 | 0.19 | 1 | 4 |
| MRPS26 | 0.005888 | 0.24155988 | 0.312 | 0.147 | 1 | 4 |
| GTF2A1 | 0.005954 | 0.32583823 | 0.438 | 0.264 | 1 | 4 |

| | | | | | | |
|---|---|---|---|---|---|---|
| PRMT5 | 0.005964 | 0.36701857 | 0.375 | 0.205 | 1 | 4 |
| SEPHS1 | 0.00597 | 0.34647202 | 0.688 | 0.511 | 1 | 4 |
| PDS5A | 0.005995 | 0.40239368 | 0.562 | 0.399 | 1 | 4 |
| COPS9 | 0.006005 | 0.29920993 | 0.844 | 0.695 | 1 | 4 |
| HSP90AB1 | 0.006017 | 0.21079396 | 1 | 0.994 | 1 | 4 |
| UBE2I | 0.006072 | 0.2749859 | 0.906 | 0.847 | 1 | 4 |
| CAT | 0.006079 | 0.33948161 | 0.5 | 0.307 | 1 | 4 |
| INTS13 | 0.006231 | 0.30121635 | 0.281 | 0.127 | 1 | 4 |
| MRPS10 | 0.006357 | 0.28940756 | 0.625 | 0.45 | 1 | 4 |
| TOMM5 | 0.006385 | 0.26834002 | 0.781 | 0.703 | 1 | 4 |
| ATP5A1 | 0.006472 | 0.35417336 | 0.969 | 0.879 | 1 | 4 |
| PREX1 | 0.00658 | 0.31681713 | 0.438 | 0.26 | 1 | 4 |
| EIF5A | 0.006584 | 0.37709482 | 0.688 | 0.546 | 1 | 4 |
| MRFAP1 | 0.006624 | 0.24458416 | 1 | 0.937 | 1 | 4 |
| RP11-423H | 0.006663 | 0.36709436 | 0.594 | 0.438 | 1 | 4 |
| VAPA | 0.00672 | 0.30783374 | 0.625 | 0.501 | 1 | 4 |
| EIF1AD | 0.0068 | 0.24557298 | 0.344 | 0.168 | 1 | 4 |
| IPPK | 0.006812 | 0.26781968 | 0.375 | 0.194 | 1 | 4 |
| MIF | 0.00685 | 0.31260735 | 0.969 | 0.947 | 1 | 4 |
| HNRNPD | 0.006896 | 0.28391957 | 0.75 | 0.652 | 1 | 4 |
| TSEN15 | 0.006953 | 0.27419979 | 0.562 | 0.387 | 1 | 4 |
| RP3-337H4 | 0.006959 | 0.30690282 | 0.844 | 0.75 | 1 | 4 |
| TMEM97 | 0.007006 | 0.47318687 | 0.469 | 0.301 | 1 | 4 |
| TMEM14A | 0.007013 | 0.36182157 | 0.469 | 0.295 | 1 | 4 |
| AC009133. | 0.007029 | 0.2283279 | 0.281 | 0.129 | 1 | 4 |
| SRSF4 | 0.007062 | 0.24507426 | 0.438 | 0.25 | 1 | 4 |
| MCMBP | 0.007173 | 0.29899038 | 0.406 | 0.235 | 1 | 4 |
| H1F0 | 0.007278 | 0.27508783 | 0.281 | 0.129 | 1 | 4 |
| RP11-543B | 0.007407 | 0.29021149 | 0.375 | 0.227 | 1 | 4 |
| TUBGCP2 | 0.007476 | 0.32594588 | 0.469 | 0.295 | 1 | 4 |
| EXOSC2 | 0.007485 | 0.27943472 | 0.281 | 0.121 | 1 | 4 |
| UBL4A | 0.007508 | 0.23740848 | 0.344 | 0.168 | 1 | 4 |
| CHTF8 | 0.007559 | 0.26459062 | 0.406 | 0.213 | 1 | 4 |
| UTP18 | 0.007755 | 0.26370107 | 0.562 | 0.341 | 1 | 4 |
| STIP1 | 0.007837 | 0.45261579 | 0.656 | 0.575 | 1 | 4 |
| DCTN1 | 0.007845 | 0.32884814 | 0.594 | 0.409 | 1 | 4 |
| PPP2R2C | 0.007854 | 0.18121163 | 0.312 | 0.149 | 1 | 4 |
| ZNF814 | 0.007923 | 0.20090094 | 0.5 | 0.295 | 1 | 4 |
| TSPAN13 | 0.008078 | 0.38034646 | 0.75 | 0.658 | 1 | 4 |
| RCC1 | 0.00811 | 0.21407653 | 0.406 | 0.223 | 1 | 4 |
| TRA2A | 0.008178 | 0.18866119 | 0.531 | 0.337 | 1 | 4 |
| FUS | 0.008225 | 0.39428498 | 0.688 | 0.667 | 1 | 4 |
| CA12 | 0.008245 | 0.40442285 | 0.406 | 0.231 | 1 | 4 |
| XBP1 | 0.008329 | 0.39054687 | 0.781 | 0.705 | 1 | 4 |
| CSDE1 | 0.008477 | 0.1521663 | 1 | 1 | 1 | 4 |
| IMPDH1 | 0.0085 | 0.31295135 | 0.312 | 0.155 | 1 | 4 |
| TRAPPC12 | 0.008593 | 0.16517393 | 0.281 | 0.123 | 1 | 4 |

| | | | | | | |
|---|---|---|---|---|---|---|
| DNLZ | 0.008636 | 0.27604859 | 0.344 | 0.174 | 1 | 4 |
| RPL10 | 0.008741 | 0.30777053 | 1 | 1 | 1 | 4 |
| ARF6 | 0.008807 | 0.24778923 | 0.656 | 0.481 | 1 | 4 |
| RPL21 | 0.008884 | 0.27460564 | 0.656 | 0.497 | 1 | 4 |
| PRPF19 | 0.008951 | 0.24550035 | 0.5 | 0.339 | 1 | 4 |
| RNPS1 | 0.00902 | 0.24404848 | 0.781 | 0.681 | 1 | 4 |
| CDK2AP1 | 0.009037 | 0.38812252 | 0.781 | 0.748 | 1 | 4 |
| ANXA5 | 0.009041 | 0.34964568 | 0.531 | 0.337 | 1 | 4 |
| MTCP1 | 0.009079 | 0.21292356 | 0.375 | 0.19 | 1 | 4 |
| PLP2 | 0.009192 | 0.23059384 | 0.562 | 0.374 | 1 | 4 |
| NQO2 | 0.009383 | 0.34959541 | 0.469 | 0.297 | 1 | 4 |
| HNRNPH3 | 0.009475 | 0.37773726 | 0.688 | 0.624 | 1 | 4 |
| TRA2B | 0.009557 | 0.38398698 | 0.812 | 0.658 | 1 | 4 |
| FBXO45 | 0.009573 | 0.37076606 | 0.562 | 0.421 | 1 | 4 |
| MPRIP | 0.009815 | 0.25316342 | 0.375 | 0.194 | 1 | 4 |
| C19orf48 | 0.009915 | 0.31139167 | 0.438 | 0.266 | 1 | 4 |
