# Supplementary material for: Estrogen Receptor-Regulated Gene Signatures in Invasive Breast Cancer Cells and Aggressive Breast Tumors
Source: Cancers (Basel). 2022 Jun 9;14(12):2848. doi: 10.3390/cancers14122848 (PMC9221274; doi:10.3390/cancers14122848)
Supplement: Supplementary file 1 [file cancers-14-02848-s001.zip › Table S6.pdf]

Table S6: Statistical analysis of clinical parameters associated with signature 1.

Clinical Attribute	Attribute Type	Statistical Test	p-Value	q-Value	
Integrative Cluster	Patient	Chi-squared Test	0	0	
ER Status	Sample	Chi-squared Test	0	0	(Fig. 6A)
PR Status	Sample	Chi-squared Test	0	0	
Pam50 + Claudin-low subtype	Patient	Chi-squared Test	0	0	
Neoplasm Histologic Grade	Sample	Chi-squared Test	0.00E+00	0.00E+00	(Fig. 6B)
3-Gene classifier subtype	Patient	Chi-squared Test	0.00E+00	0.00E+00	
ER status measured by IHC	Patient	Chi-squared Test	0.00E+00	0.00E+00	
Nottingham prognostic index	Patient	Wilcoxon Test	0.00E+00	0.00E+00	
Chemotherapy	Patient	Chi-squared Test	8.18E-11	2.91E-10	
Cellularity	Patient	Chi-squared Test	6.00E-10	1.92E-09	
HER2 status measured by SNP6	Patient	Chi-squared Test	6.98E-10	2.03E-09	
Tumor Other Histologic Subtype	Patient	Chi-squared Test	1.49E-09	3.98E-09	
Oncotree Code	Sample	Chi-squared Test	1.05E-06	2.40E-06	
Cancer Type Detailed	Sample	Chi-squared Test	1.05E-06	2.40E-06	
HER2 Status	Sample	Chi-squared Test	3.43E-05	7.32E-05	
Hormone Therapy	Patient	Chi-squared Test	4.61E-04	8.20E-04	
Patient's Vital Status	Patient	Chi-squared Test	9.43E-04	1.59E-03	(Fig. 6C)
Tumor Size	Sample	Chi-squared Test	4.33E-03	6.93E-03	
Tumor Stage	Sample	Chi-squared Test	0.0149	0.0226	
Radio Therapy	Patient	Chi-squared Test	0.0741	0.0988	
Cohort	Patient	Chi-squared Test	0.234	0.299	
Inferred Menopausal State	Patient	Chi-squared Test	0.284	0.35	
Age at Diagnosis	Patient	Wilcoxon Test	0.382	0.453	
Lymph nodes examined positive	Patient	Chi-squared Test	0.715	0.817	
Type of Breast Surgery	Patient	Chi-squared Test	0.773	0.853	
Cancer Type	Sample	Chi-squared Test	0.84	0.896	
Mutation Count	Sample	Wilcoxon Test	0.95	0.963	
Primary Tumor Laterality	Patient	Chi-squared Test	0.963	0.963	

Survival Type	Number of Patients	# in Altered group	# in Unaltered group	Median months survival in Altered group (95% CI)	Median months survival in Unaltered group (95% CI)	p-Value	q-Value	
Relapse Free	1903	1140	763	210.39 (171.88 - NA)	248.95 (196.38 - NA)	3.47E-03	6.48E-03	(Fig. 6E)
Overall	1904	1140	764	145.73 (131.30 - 159.07)	171.63 (153.90 - 187.03)	6.48E-03	6.48E-03	(Fig. 6D)
