# Supplementary material for: Estrogen Receptor-Regulated Gene Signatures in Invasive Breast Cancer Cells and Aggressive Breast Tumors
Source: Cancers (Basel). 2022 Jun 9;14(12):2848. doi: 10.3390/cancers14122848 (PMC9221274; doi:10.3390/cancers14122848)
Supplement: Supplementary file 1 [file cancers-14-02848-s001.zip › Table S7.pdf]

Table S7: Statistical analysis of clinical parameters associated with signature 2.

Clinical Attribute	Attribute Type	Statistical Test	p-Value	q-Value	
Integrative Cluster	Patient	Chi-squared Test	0	0	
ER Status	Sample	Chi-squared Test	0	0	(Fig. 6A)
PR Status	Sample	Chi-squared Test	0	0	
Pam50 + Claudin-low subtype	Patient	Chi-squared Test	0	0	
Neoplasm Histologic Grade	Sample	Chi-squared Test	0.00E+00	0.00E+00	
3-Gene classifier subtype	Patient	Chi-squared Test	0.00E+00	0.00E+00	
ER status measured by IHC	Patient	Chi-squared Test	0.00E+00	0.00E+00	(Fig. 6B)
Nottingham prognostic index	Patient	Wilcoxon Test	2.22E-16	8.88E-16	
HER2 status measured by SNP6	Patient	Chi-squared Test	4.72E-13	1.68E-12	
Chemotherapy	Patient	Chi-squared Test	1.29E-09	4.14E-09	
Tumor Other Histologic Subtype	Patient	Chi-squared Test	2.79E-09	8.10E-09	
HER2 Status	Sample	Chi-squared Test	4.82E-08	1.29E-07	
Oncotree Code	Sample	Chi-squared Test	1.55E-07	3.55E-07	
Cancer Type Detailed	Sample	Chi-squared Test	1.55E-07	3.55E-07	
Cellularity	Patient	Chi-squared Test	9.71E-06	2.07E-05	
Hormone Therapy	Patient	Chi-squared Test	4.05E-04	7.63E-04	(Fig. 6C)
Patient's Vital Status	Patient	Chi-squared Test	9.17E-04	1.55E-03	
Tumor Stage	Sample	Chi-squared Test	0.0614	0.0893	
Tumor Size	Sample	Chi-squared Test	0.117	0.163	
Radio Therapy	Patient	Chi-squared Test	0.203	0.271	
Age at Diagnosis	Patient	Wilcoxon Test	0.321	0.4	
Inferred Menopausal State	Patient	Chi-squared Test	0.325	0.4	
Mutation Count	Sample	Wilcoxon Test	0.507	0.601	
Primary Tumor Laterality	Patient	Chi-squared Test	0.572	0.654	
Cancer Type	Sample	Chi-squared Test	0.64	0.706	
Lymph nodes examined positive	Patient	Chi-squared Test	0.721	0.769	
Type of Breast Surgery	Patient	Chi-squared Test	0.767	0.791	
Cohort	Patient	Chi-squared Test	0.903	0.903	

Survival Type	Number of Patients	# in Altered group	# in Unaltered group	Median months survival in Altered group (95% CI)	Median months survival in Unaltered group (95% CI)	p-Value	q-Value	
Relapse Free	1903	1354	549	248.95 (182.43 - NA)	221.97 (187.47 - NA)	5.03E-03	5.03E-03	(Fig. 6D)
Overall	1904	1355	549	147.77 (139.30 - 162.83)	172.90 (153.90 - 194.70)	3.46E-03	5.03E-03	
