# Supplementary material for: Estrogen Receptor-Regulated Gene Signatures in Invasive Breast Cancer Cells and Aggressive Breast Tumors
Source: Cancers (Basel). 2022 Jun 9;14(12):2848. doi: 10.3390/cancers14122848 (PMC9221274; doi:10.3390/cancers14122848)
Supplement: Supplementary file 1 [file cancers-14-02848-s001.zip › Table S8.pdf]

**Table S8: Statistical analysis of clinical parameters associated with signature 1, where the genes overlapping with signature 2 were removed.**

**Signature 1 without overlap**

| Clinical Attribute | Attribute Type | Statistical Test | p-Value | q-Value | |
|---|---|---|---|---|---|
| Integrative Cluster | Patient | Chi-squared Test | 0 | 0 | |
| ER Status | Sample | Chi-squared Test | 0 | 0 | |
| PR Status | Sample | Chi-squared Test | 0 | 0 | |
| Pam50 + Claudin-low subtype | Patient | Chi-squared Test | 0 | 0 | Figure S2A |
| Neoplasm Histologic Grade | Sample | Chi-squared Test | 0 | 0 | Figure S2B |
| 3-Gene classifier subtype | Patient | Chi-squared Test | 0 | 0 | |
| ER status measured by IHC | Patient | Chi-squared Test | 0 | 0 | |
| Nottingham prognostic index | Patient | Wilcoxon Test | 0 | 0 | |
| Chemotherapy | Patient | Chi-squared Test | 1.41E-14 | 5.01E-14 | |
| Tumor Other Histologic Subtype | Patient | Chi-squared Test | 6.24E-12 | 2.00E-11 | |
| Cellularity | Patient | Chi-squared Test | 4.55E-09 | 1.32E-08 | |
| Hormone Therapy | Patient | Chi-squared Test | 6.24E-08 | 1.66E-07 | |
| Oncotree Code | Sample | Chi-squared Test | 1.32E-06 | 3.03E-06 | |
| Cancer Type Detailed | Sample | Chi-squared Test | 1.32E-06 | 3.03E-06 | |
| HER2 status measured by SNP6 | Patient | Chi-squared Test | 4.78E-06 | 9.56E-06 | |
| HER2 Status | Sample | Chi-squared Test | 2.92E-05 | 5.18E-05 | |
| Patient's Vital Status | Patient | Chi-squared Test | 2.13E-04 | 3.44E-04 | Figure S2C |
| Tumor Stage | Sample | Chi-squared Test | 2.15E-04 | 3.44E-04 | |
| Cohort | Patient | Chi-squared Test | 7.47E-03 | 0.0114 | |
| Radio Therapy | Patient | Chi-squared Test | 8.16E-03 | 0.0116 | |
| Tumor Size | Sample | Chi-squared Test | 8.34E-03 | 0.0116 | |

| | | | | |
|---|---|---|---|---|
| Inferred Menopausal State | Patient | Chi-squared Test | 0.0323 | 0.0414 |
| Mutation Count | Sample | Wilcoxon Test | 0.252 | 0.296 |
| Age at Diagnosis | Patient | Wilcoxon Test | 0.259 | 0.296 |
| Lymph nodes examined positive | Patient | Chi-squared Test | 0.435 | 0.48 |
| Primary Tumor Laterality | Patient | Chi-squared Test | 0.795 | 0.848 |
| Type of Breast Surgery | Patient | Chi-squared Test | 0.822 | 0.848 |
| Cancer Type | Sample | Chi-squared Test | 0.883 | 0.883 |

| **Survival Type** | **Number of Patients** | **# in Altered group** | **# in Unaltered group** | **Median months survival in Altered group (95% CI)** | **Median months survival in Unaltered group (95% CI)** | **p-Value** | **q-Value** | |
|---|---|---|---|---|---|---|---|---|
| Relapse Free | 1903 | 813 | 1090 | 195.23 (165.07 - NA) | 273.29 (206.22 - NA) | 4.30E-04 | 8.60E-04 | Figure S2E |
| Overall | 1904 | 813 | 1091 | 137.93 (120.13 - 151.20) | 168.97 (153.90 - 182.60) | 2.13E-03 | 2.13E-03 | Figure S2D |
