# Supplementary material for: Estrogen Receptor-Regulated Gene Signatures in Invasive Breast Cancer Cells and Aggressive Breast Tumors
Source: Cancers (Basel). 2022 Jun 9;14(12):2848. doi: 10.3390/cancers14122848 (PMC9221274; doi:10.3390/cancers14122848)
Supplement: Supplementary file 1 [file cancers-14-02848-s001.zip › Table S9.pdf]

**Table S9: Statistical analysis of clinical parameters associated with signature 2, where the genes overlapping with signature 1 were removed.**
**Siganture 2 without overlap**

| Clinical Attribute | Attribute Type | Statistical Test | p-Value | q-Value | |
|---|---|---|---|---|---|
| Integrative Cluster | Patient | Chi-squared Test | 0 | 0 | |
| ER Status | Sample | Chi-squared Test | 0 | 0 | |
| PR Status | Sample | Chi-squared Test | 0 | 0 | |
| Pam50 + Claudin-low subtype | Patient | Chi-squared Test | 0 | 0 | Figure S2F |
| Neoplasm Histologic Grade | Sample | Chi-squared Test | 0 | 0 | Figure S2G |
| 3-Gene classifier subtype | Patient | Chi-squared Test | 0 | 0 | |
| ER status measured by IHC | Patient | Chi-squared Test | 0 | 0 | |
| Nottingham prognostic index | Patient | Wilcoxon Test | 1.11E-16 | 4.44E-16 | |
| Chemotherapy | Patient | Chi-squared Test | 5.62E-12 | 2.00E-11 | |
| Hormone Therapy | Patient | Chi-squared Test | 1.51E-09 | 4.84E-09 | |
| HER2 Status | Sample | Chi-squared Test | 2.23E-09 | 6.50E-09 | |
| Tumor Other Histologic Subtype | Patient | Chi-squared Test | 3.36E-07 | 8.95E-07 | |
| HER2 status measured by SNP6 | Patient | Chi-squared Test | 4.35E-07 | 1.07E-06 | |
| Oncotree Code | Sample | Chi-squared Test | 6.09E-07 | 1.30E-06 | |
| Cancer Type Detailed | Sample | Chi-squared Test | 6.09E-07 | 1.30E-06 | |
| Cellularity | Patient | Chi-squared Test | 6.32E-06 | 1.26E-05 | |
| Inferred Menopausal State | Patient | Chi-squared Test | 0.0118 | 0.0223 | |
| Radio Therapy | Patient | Chi-squared Test | 0.0187 | 0.0299 | |
| Tumor Size | Sample | Chi-squared Test | 0.049 | 0.0747 | |
| Age at Diagnosis | Patient | Wilcoxon Test | 0.0556 | 0.0808 | |
| Tumor Stage | Sample | Chi-squared Test | 0.0612 | 0.0851 | |
| Patient's Vital Status | Patient | Chi-squared Test | 0.0931 | 0.124 | Figure S2H |

| | | | | |
|---|---|---|---|---|
| Lymph nodes examined positive | Patient | Chi-squared Test | 0.154 | 0.197 |
| Cohort | Patient | Chi-squared Test | 0.294 | 0.349 |
| Mutation Count | Sample | Wilcoxon Test | 0.579 | 0.639 |
| Type of Breast Surgery | Patient | Chi-squared Test | 0.734 | 0.783 |
| Primary Tumor Laterality | Patient | Chi-squared Test | 0.943 | 0.973 |
| Cancer Type | Sample | Chi-squared Test | 0.99 | 0.99 |

| Survival Type | Number of Patients | # in Altered group | # in Unaltered group | Median months survival in Altered group (95% CI) | Median months survival in Unaltered group | p-Value | q-Value | |
|---|---|---|---|---|---|---|---|---|
| Relapse Free | 1903 | 939 | 964 | 248.95 (182.76 - NA) | 215.79 (183.16 - | 0.0536 | 0.107 | Figure S2I |
| Overall | 1904 | 940 | 964 | 148.87 (131.33 - | 161.67 (149.70 - | 0.195 | 0.195 | Figure S2J |
